# Bioengineered exosome-mRNA hybrids: a breakthrough in targeted miRNA delivery for diabetic kidney fibrosis therapy

**DOI:** 10.3389/fbioe.2026.1709588

**Published:** 2026-03-02

**Authors:** Jing Ke, Lei Cao, Shaochun Zhang, Jili Xing

**Affiliations:** 1 Department of Endocrinology, The Central Hospital of Ezhou, Ezhou, China; 2 Department of Geriatrics, The Central Hospital of Ezhou, Ezhou, China; 3 Department of Orthopedics, The Central Hospital of Ezhou, Ezhou, China; 4 Department of Gastroenterology, The Central Hospital of Ezhou, Ezhou, China

**Keywords:** biomimetic red blood cell membrane, diabetic nephropathy, podocyte targeting, poly(ADP-ribose) polymerase 1, small interfering RNA delivery, TGFβ/smads pathway

## Abstract

**Introduction:**

Diabetic nephropathy (DN) is characterized by progressive podocyte injury, yet actionable upstream regulators and precise targeted delivery strategies remain limited. This study investigated the role of poly (ADP-ribose) polymerase 1 (PARP1) in hyperglycemia-induced podocyte damage and developed a biomimetic targeted siRNA delivery system to silence PARP1.

**Methods:**

Transcriptomic profiling was performed in MPC5 podocytes exposed to high glucose. Functional validation was conducted using the PARP1 inhibitor PJ-34 and PARP1 gene silencing *in vitro* and in a streptozotocin-induced type 1 diabetic mouse model. A PLGA-core nanoparticle system loaded with PARP1 siRNA and coated with red blood cell membrane (RBCm), further functionalized with the podocyte-targeting ligand BMS-α, was engineered and evaluated for targeting efficiency and therapeutic efficacy.

**Results:**

PARP1 was significantly upregulated under high-glucose conditions and associated with activation of the TGFβ/Smads signaling pathway. Pharmacological inhibition and gene silencing of PARP1 attenuated pathway activation, restored autophagic flux, and reduced apoptosis, inflammation, and profibrotic responses *in vitro*, while alleviating glomerular injury in diabetic mice. The siPARP1-NPs@RBCm-BMS-α system demonstrated favorable physicochemical stability, effective siRNA encapsulation, enhanced podocyte targeting, and improved renal structure and function *in vivo*.

**Discussion:**

These findings identify PARP1 as a key regulator of podocyte injury in DN via the TGFβ/Smads pathway and support the biomimetic receptor-relevant siRNA platform as a promising targeted therapeutic strategy for diabetic nephropathy.

## Introduction

Diabetic nephropathy (DN) is one of the most severe microvascular complications of diabetes and represents the leading cause of end-stage renal disease (ESRD) worldwide (Zoja et al., 2020; Liu et al., 2023). Current clinical management primarily relies on conventional approaches, including glycemic control, blood pressure regulation, and renin-angiotensin-aldosterone system (RAAS) blockade. However, these strategies often fail to halt disease progression and exert only limited restorative effects on early structural damage to the kidney (Samsu, 2021; Sulaiman, 2024). Podocytes are pivotal for maintaining the integrity of the glomerular filtration barrier, and their structural and functional impairment plays a central role in DN pathogenesis (Rao et al., 2024; Barutta et al., 2022). Exposure to a hyperglycemic milieu directly injures podocytes, inducing apoptosis, functional dysregulation, and foot process effacement, ultimately leading to proteinuria and glomerulosclerosis (Nakamichi et al., 2021; Wolf and Ziyadeh, 2007). Elucidating the molecular mechanisms underlying hyperglycemia-induced podocyte injury and identifying effective therapeutic targets remain pressing challenges in DN research (Zhou et al., 2022). The disease’s pathogenesis is highly complex, encompassing inflammation, oxidative stress, apoptosis, and fibrosis, yet the key regulatory molecules have not been fully defined (Feng et al., 2021).

Poly (ADP-ribose) polymerase 1 (PARP1) is a DNA damage sensor enzyme that orchestrates multiple cellular stress responses, including inflammation, energy metabolism, and apoptosis (Langelier et al., 2018; Qin et al., 2016). In recent years, its aberrant upregulation in metabolic disorders has attracted increasing attention, with accumulating evidence indicating a pivotal role in the pathophysiology of diabetes and its complications (Brady et al., 2019; Arruri et al., 2021). Hyperactivation of PARP1 exacerbates oxidative stress, promotes pro-inflammatory cytokine release, and depletes intracellular NAD^+^ and ATP, culminating in cellular dysfunction and death (Martín-Guerrero et al., 2020; Abdellatif et al., 2023). In the kidney, elevated PARP1 expression correlates with tubulointerstitial fibrosis and glomerulosclerosis (Ke et al., 2025; Xu et al., 2021). Nevertheless, its podocyte-specific actions—particularly whether it drives injury and dysfunction under hyperglycemic stress via defined signaling cascades—remain insufficiently characterized (Shevalye et al., 2010). Moreover, the evidence linking PARP1 to canonical fibrotic pathways is fragmentary, necessitating transcriptomic and mechanistic studies to clarify its pathogenic role in podocyte injury (Liu et al., 2022; Lin et al., 2021).

Transforming growth factor-β (TGF-β) and its downstream Smads cascade constitute a central signaling axis in DN, mediating extracellular matrix accumulation, inflammation, and fibrosis (Chen et al., 2023). Activation of this pathway induces overexpression of fibrosis-related genes such as Alpha-Smooth Muscle Actin (α-SMA) and collagen I, thereby driving structural damage and functional decline in the glomerulus (Ji et al., 2018; You et al., 2019). Existing studies have reported interactions between the PARP family and the TGFβ/Smads signaling pathway in various disease contexts; however, the specific mechanisms involved in podocytes remain unclear (Le et al., 2020; Chen et al., 2024). We hypothesize that in DN, PARP1 may act as an upstream regulator that aggravates podocyte injury and fibrotic transformation through activation of the TGF-β/Smads pathway. Based on this hypothesis, this study focuses on the molecular alterations of podocytes under high-glucose (HG) conditions, integrating transcriptomic analysis with functional validation to investigate the potential link between PARP1 and the TGFβ/Smads pathway, and to construct a theoretical model of its role in podocyte injury, thereby providing new insights for targeted interventions in DN.

Although small-molecule inhibitors such as PJ-34 can partially suppress PARP1 activity and improve podocyte function, their clinical translation remains hindered by challenges, including poor plasma stability, limited target specificity, and adverse side effects (Zhu et al., 2021; Szabó et al., 2006). In recent years, RNA interference (RNAi) has emerged as a powerful strategy for silencing disease-associated genes, with small interfering RNA (siRNA) offering precise suppression of pathogenic targets along with superior specificity and biocompatibility (Redhwan et al., 2024; Moqbel Redhwan et al., 2024). Nevertheless, rapid degradation *in vivo*, low cellular uptake, and insufficient tissue selectivity continue to restrict the therapeutic potential of siRNA (Bidar et al., 2022; Morales-Becerril et al., 2022). To address these limitations, biomimetic nanocarrier systems have been developed, with red blood cell membrane (RBCm)-coated poly (lactic-co-glycolic acid) (PLGA) nanoparticles (NPs) attracting particular attention due to their inherent immune evasion, prolonged circulation, and favorable biocompatibility (Huang et al., 2023; Miele et al., 2021). In this study, we integrated the podocyte-targeting lipid ligand Bone Morphogenetic Protein-derived Podocyte-Targeting Peptide α (BMS-α) into the surface of RBCm-coated NPs to create a novel biomimetic delivery platform—siPARP1-NPs@RBCm-BMS-α—capable of encapsulating PARP1-specific siRNA. This design simultaneously optimizes delivery efficiency, safety, and targeting capacity, offering substantial promise for renal disease intervention.

This study aims to elucidate the molecular mechanisms by which PARP1 contributes to podocyte injury in DN, with particular emphasis on its pathogenic role mediated through the TGFβ/Smads signaling axis. In parallel, we developed and validated small interfering RNA targeting PARP1–loaded nanoparticles coated with red blood cell membrane and functionalized with the podocyte-targeting ligand BMS-α (sGiPARP1-NPs@RBCm-BMS-α) for efficient silencing of PARP1 and targeted functional intervention. The findings may offer new insights into the molecular pathology of DN and provide theoretical and technological groundwork for advancing particular and potentially translatable nucleic acid–based podocyte-targeted therapeutic strategies.

## Materials and methods

### Cell culture and experimental grouping

The mouse podocyte cell line MPC5 was obtained from the Cell Bank of the Chinese Academy of Sciences (Shanghai, China) and cultured in McCoy’s 5A medium (16600082, Thermo Fisher Scientific) supplemented with 10% fetal bovine serum (FBS; 10099141, Thermo Fisher Scientific) at 37 °C in a humidified incubator containing 5% CO_2_.

For experiments involving the PARP1 inhibitor PJ-34, cells were seeded into six-well plates (3516, Corning) at a density of 5 × 10^5^ cells/well and cultured for 24 h until reaching ∼70% confluence. The medium was then replaced, and cells were allocated to the following groups: NC: normal control, 5.5 mM glucose (G7021, Sigma); NC + PJ-34: 5.5 mM glucose supplemented with 10 μM PJ-34 (S1192, Selleckchem; final DMSO concentration <0.1%); HG: HG injury induced by 30 mM glucose; HG + PJ-34: 30 mM glucose supplemented with 10 μM PJ-34. All groups were incubated for 48 h before cells were harvested for further analyses.

For siPARP1-NPs@RBCm-BMS-α experiments, cells were seeded under the same conditions and, after 24 h, the medium was replaced with McCoy’s 5A containing 30 mM glucose. The treatment groups were as follows: HG: 30 mM glucose only; NPs@RBCm-BMS-α: 50 μM empty NPs; siPARP1-NPs@RBCm: 50 μM siPARP1-loaded NPs lacking BMS-α modification; siPARP1-NPs@RBCm-BMS-α: 50 μM fully functionalized NPs; siPARP1: 50 μM siPARP1 + the commercial transfection reagent riboFECT™ CP (RiboBio, China). After 48 h of incubation, cells from all groups were collected for subsequent analyses.

### RNA extraction and library preparation

Following Phosphate-Buffered Saline (PBS) washes, cells were lysed in 1 mL TRIzol reagent (Invitrogen, Cat#15596018), and total RNA was extracted according to the manufacturer’s protocol. RNA concentration and purity were determined using a NanoDrop 2000 spectrophotometer (Thermo Fisher Scientific). Samples with an A260/A280 ratio between 1.8 and 2.1 and acceptable A260/A230 values (indicating minimal organic contamination) were retained. RNA integrity was assessed using an Agilent 2100 Bioanalyzer (Agilent Technologies), and only samples with an RNA Integrity Number (RIN) ≥ 8.0 were used for library construction.

mRNA libraries were prepared following the Illumina TruSeq Stranded mRNA Library Prep Kit protocol (Illumina, Cat#20020594). Briefly, polyadenylated mRNA was enriched from 1 μg of total RNA using oligo (dT) magnetic beads, followed by high-temperature fragmentation to yield 200–300 bp fragments. First-strand cDNA was synthesized using SuperScript II reverse transcriptase and random primers, and second-strand synthesis generated double-stranded cDNA. Ends were repaired, A-tailed, and ligated to indexed adapters. Libraries were amplified by 12–15 PCR cycles to obtain sufficient yield, and amplicons were purified using AMPure XP magnetic beads.

Final libraries were quantified with a Qubit 3.0 fluorometer (Invitrogen) and assessed on an Agilent 2100 Bioanalyzer to confirm an insert size distribution of 250–300 bp with a single, distinct peak. Qualified libraries were pooled and sequenced on an Illumina NovaSeq 6000 platform using paired-end 150 bp reads, generating an average of 6 Gbp per sample to ensure adequate transcriptome coverage and quantitative reliability. All procedures were performed under RNase-free conditions, and two technicians independently verified library quality control and labeling to ensure reproducibility and accuracy.

### Sequencing data processing and differential expression analysis

Raw sequencing reads in FASTQ format were first quality-checked using FastQC (v0.11.9), assessing metrics such as base quality distribution, sequence duplication levels, GC content, and adapter contamination. To remove adapters and low-quality reads, Trimmomatic (v0.39) was applied, retaining reads with an average quality score ≥20 and a minimum length of 36 bp (clean reads). After filtering, clean reads were aligned to the mouse reference genome GRCm39 (GENCODE Release M30) using HISAT2 (v2.1.0) with default parameters and the “-rna-strandness RF” option enabled to preserve strand specificity. Alignment outputs in BAM format were subsequently used for downstream quantification.

Gene-level read counts for each sample were obtained using featureCounts (v2.0.1, Subread package) with the GENCODE M30 GTF annotation. The resulting count matrix was transformed into both TPM (Transcripts Per Million) values for expression visualization and raw counts for statistical analysis. Before differential expression analysis, the count matrix was normalized using the median of ratios method to correct for systematic variation among samples. DEGs were identified using DESeq2 (v1.34.0) with the Wald test under a negative binomial model.

Genes with a *p*-value <0.05 and |log_2_FoldChange| > 1 were considered significant, with multiple testing correction performed using the Benjamini–Hochberg procedure to control the false discovery rate (FDR). Volcano plots and heatmaps were generated in R using ggplot2 (v3.3.5) and pheatmap (v1.0.12).

### Gene ontology (GO) and kyoto encyclopedia of genes and genomes (KEGG) pathway enrichment analysis

GO and KEGG enrichment analyses of the 59 identified DEGs were conducted using the clusterProfiler package (v4.2.2) in R. GO annotations were obtained from the GO database (https://geneontology.org), and KEGG pathway information from the KEGG database (https://www.kegg.jp). All genes were standardized to Ensembl gene IDs, with the species set to *Mus musculus* and the background defined as all expressed genes in the reference transcriptome to ensure analytical consistency.

GO enrichment was calculated separately for the Biological Process (BP), Molecular Function (MF), and Cellular Component (CC) categories using a two-tailed hypergeometric test, with FDR control via the Benjamini–Hochberg method. KEGG pathway enrichment followed identical significance criteria (FDR <0.05), and pathways were ranked by enrichment factor (GeneRatio).

### PPI network construction and visualization

To explore potential protein-level regulatory relationships among the DEGs, PPI network prediction was performed using the STRING database (v11.5, https://string-db.org). The input comprised the 59 DEGs identified by DESeq2 (|log_2_FC| > 1, *p* < 0.05), restricted to *Mus musculus*. The minimum interaction confidence score was set at 0.4 (high confidence) to enhance reliability.

### Reverse transcription quantitative polymerase chain reaction (RT-qPCR)

Total RNA was extracted using TRIzol reagent (15596026, Invitrogen, United States), and RNA concentration and purity were measured with a NanoDrop 2000 spectrophotometer (ND-2000, Thermo Scientific, United States). One microgram of RNA was reverse-transcribed into cDNA using the HiScript III RT SuperMix kit (R323-01, Vazyme, China). Quantitative real-time PCR was performed with ChamQ Universal SYBR qPCR Master Mix (Q711-02, Vazyme, China) on an ABI StepOnePlus system (4376600, Applied Biosystems, United States). Relative expression levels were calculated using the 2^−ΔΔCt^ method. Each sample was analyzed in triplicate (The specific primer information is listed in Supplementary Table S1).

### Western blot (WB) analysis

Total protein was extracted using Radio-Immunoprecipitation Assay (RIPA) lysis buffer (P0013B, Beyotime, China) supplemented with a protease inhibitor cocktail (04693132001, Roche, Switzerland), phenylmethylsulfonyl fluoride (PMSF; ST506, Beyotime), and a phosphatase inhibitor (78420, Thermo Fisher, United States). After lysis on ice for 30 min, lysates were centrifuged, and the supernatants were collected. Protein concentrations were determined using the Bicinchoninic Acid (BCA) assay (23225, Thermo Fisher, United States). Equal amounts of protein (30 μg per lane) were separated by sodium dodecyl sulfate-polyacrylamide gel electrophoresis (SDS-PAGE) and transferred onto polyvinylidene difluoride (PVDF) membranes (IPVH00010, Millipore, United States). Membranes were blocked with 5% non-fat milk (232100, BD Biosciences, United States) for 1 h at room temperature and incubated overnight at 4 °C with the following primary antibodies: PARP1 (ab191217, Abcam, United Kingdom; 1:1000), TGFβ1 (ab92486, Abcam, United Kingdom; 1:1000), Smad3 (ab40854, Abcam, United Kingdom; 1:1000), LC3B (ab192890, Abcam, United Kingdom; 1:2000), P62 (ab109012, Abcam; 1:10000), α-SMA (ab5694, Abcam; 1 μg/mL), Collagen I (ab270993, Abcam, United Kingdom; 1:1000), and GAPDH (#5174, CST; 1:1000) as the loading control. The following day, membranes were incubated with Horseradish peroxidase (HRP)-conjugated secondary antibodies (111–035-003, Jackson ImmunoResearch, United States; 1:5000) for 1 h, and signals were visualized using an Enhanced Chemiluminescence (ECL) detection kit (32106, Thermo Scientific, United States) and captured with the ChemiDoc XRS imaging system (1708265, Bio-Rad, United States).

### Cell counting Kit-8 (CCK-8) assay

Cell viability was assessed using a CCK-8 kit (CK04, Dojindo, Japan). MPC5 cells were seeded in 96-well plates (3599, Corning, United States) at a density of 5 × 10^4^ cells per well. Following treatment, 10 μL of CCK-8 solution was added to each well and incubated at 37 °C for 2 h. Absorbance was measured at 450 nm using a microplate reader. Each condition was tested in triplicate, and experiments were independently repeated three times to ensure statistical reliability.

### Cytotoxicity assay

Cytotoxicity was evaluated using a lactate dehydrogenase (LDH) release assay kit (C0016, Beyotime, China), following the manufacturer’s instructions. Briefly, 100 μL of culture supernatant from each well was mixed with 10 μL of LDH working solution and incubated in the dark for 30 min. Absorbance was recorded at 490 nm using a microplate reader. All assays were performed in triplicate and repeated three times independently.

### Flow cytometry analysis

Apoptosis was quantified using an Annexin V-Fluorescein Isothiocyanate (FITC)/PI apoptosis detection kit (556547, BD Biosciences, United States). After treatment, cells were washed with PBS (SH30256.01, HyClone, United States), digested with trypsin (25200056, Gibco, United States), and collected by centrifugation. Pellets were resuspended in 100 μL binding buffer, followed by the addition of 5 μL Annexin V-FITC and 5 μL PI. Samples were incubated in the dark at room temperature for 15 min, and fluorescence was measured using a flow cytometer (653118, BD Accuri C6, United States). Data were analyzed with FlowJo software (v10.6.2, FlowJo LLC, United States) to determine the proportions of early and late apoptotic cells. Each experiment was performed in triplicate with three independent repeats.

### Enzyme-linked immunosorbent assay (ELISA)

Following treatment, cell culture supernatants were collected and centrifuged at 1,000 × g for 10 min at room temperature. The resulting supernatants were analyzed for proinflammatory cytokines using commercial ELISA kits: TGF-α (ab277719, Abcam, United Kingdom), IL-6 (ab222503, Abcam, United Kingdom), and IL-1β (ab197742, Abcam, United Kingdom). Assays were performed strictly according to the manufacturers’ protocols. Quantification was based on standard curves, and absorbance was measured at 450 nm using a microplate reader (S1H1RAD, Synergy H1, BioTek, United States). Each experimental group included three technical replicates, and the experiment was independently repeated three times.

### Establishment and grouping of the diabetic mouse model

Eight-week-old male C57BL/6J mice (18–20 g) were obtained from Beijing Vital River Laboratory Animal Technology Co., Ltd. (Beijing, China). Mice were housed in a specific pathogen-free facility under controlled conditions (22 °C ± 2 °C, 50%–60% humidity, 12-h light/dark cycle) with free access to food and water. Animals were acclimatized for 7 days before experimentation. Type 1 diabetes mellitus (T1DM) was induced by intraperitoneal injection of streptozotocin (STZ; 572201, Sigma-Aldrich) at 55 mg/kg for five consecutive days. STZ was freshly dissolved in sterile 0.01 mol/L citrate-sodium citrate buffer (pH 4.5). Seventy-two hours after the final injection, fasting blood glucose was measured; mice with glucose ≥16.7 mmol/L and positive urine glucose were considered diabetic. Control mice received equal volumes of sterile citrate buffer. One week after diabetes induction, the DN cohort received either PJ-34 (10 mg/kg) or saline intraperitoneally once daily for 4 weeks. For nanoparticle treatment, diabetic mice were administered NPs@RBCm-BMS-α, siPARP1-NPs@RBCm, or siPARP1-NPs@RBCm-BMS-α or siPARP1 (delivered according to the riboFECT™ CP kit instructions) at a dose of 20 mg/kg for three consecutive days. After 4 weeks, mice were subjected to further analyses (Ke et al., 2025; Qu et al., 2024).

The experimental groups (n = 6 per group) were: Control (citrate buffer), T1DM (STZ), T1DM + Saline (STZ + saline), T1DM + PJ-34 (STZ + PJ-34), NPs@RBCm-BMS-α, siPARP1-NPs@RBCm, siPARP1-NPs@RBCm-BMS-α.

### Isolation of glomeruli

Mice were anesthetized via intraperitoneal injection of pentobarbital sodium (50 mg/kg), and the heart was perfused with 8 × 10^7^ Dynabeads suspended in PBS. The renal medulla and cortex were dissected, and the cortex was minced into 1 mm^3^ fragments, digested with 2% collagenase, and passed through a 100-µm cell strainer. The filtrate was centrifuged (1,500 rpm, 4 °C, 5 min), and the pellet was resuspended in PBS. Glomeruli containing Dynabeads were isolated using a magnetic particle concentrator and washed three times with PBS.

### Assessment of renal function and organ index

At the end of the 4-week treatment, mice were euthanized by an intraperitoneal injection of an overdose of pentobarbital sodium (150 mg/kg), and death was confirmed by the cessation of heartbeat and respiration. Subsequently, the kidneys were excised and weighed. The kidney weight-to-body weight ratio (mg/g) was calculated. Twenty-four-hour urine samples were collected for urinary albumin-to-creatinine ratio (UACR) analysis. Urinary albumin was quantified using a commercial ELISA kit (ab108792, Abcam, United Kingdom) and creatinine with a colorimetric assay (DICT-500, Bioassay Systems, CA, United States). UACR was calculated as:

$$\text{UACR}=\frac{\text{urinary}\;\text{albumin}}{\text{urinary}\;\text{creatinine}}\times 1.73$$

### Periodic acid-schiff (PAS) staining and semi-quantitative analysis of glomerular injury

Histopathological evaluation was performed on paraffin-embedded kidney sections (3–5 µm). Sections were deparaffinized, rehydrated, and stained with a PAS kit (Solarbio, Beijing, China) according to the manufacturer’s instructions. Glomerular injury was scored blindly based on mesangial matrix expansion and glomerular hypertrophy: 0 = no detectable expansion or hypertrophy; 1 = 0–25% involvement; 2 = 25–50%; 3 = 50–75%; 4 = 75–100%. For each sample, at least 30 glomeruli were examined under a 40× objective lens. The renal injury index was calculated as:

$$\frac{N1\times 1+N2\times 2+N3\times 3+N4\times 4}{n}$$

where N1-N4 represent the number of glomeruli with injury scores of 1-4, and n is the total number of glomeruli assessed.

### TEM

Kidney tissues were sectioned into 1–3 cm fragments and rapidly fixed in 2.5% glutaraldehyde at 4 °C. Samples were dehydrated through a graded ethanol series (50%, 70%, 80%, 90%, and 100%), embedded in epoxy resin with a curing agent, sectioned, stained, and examined under TEM. ImageJ software (National Institutes of Health, Bethesda, MD, United States) was used to quantify the number of foot processes per micrometer of the glomerular basement membrane (GBM), the average foot process width, and GBM thickness from TEM images.

### Immunohistochemistry (IHC)

Kidney specimens were fixed in 4% paraformaldehyde, paraffin-embedded, and sectioned at a thickness of 3 μm. After deparaffinization and rehydration, endogenous peroxidase activity was quenched with 3% hydrogen peroxide for 15 min, followed by blocking of nonspecific binding sites with 5% bovine serum at room temperature for 1 h (after antigen retrieval). Sections were incubated overnight at 4 °C with primary antibodies against PARP1 (ab191217, Abcam, United Kingdom; 1:1000) and TGFβ1 (ab215715, Abcam, United Kingdom; 1:1000). This was followed by incubation with secondary antibodies at room temperature for 1 h, then with HRP-conjugated streptavidin for 45 min. Visualization was achieved using 3,3′-diaminobenzidine (DAB), and nuclei were counterstained with hematoxylin. Stained sections were imaged, and the positive staining area within each glomerulus was quantified using ImageJ.

### Immunofluorescence (IF)

Paraffin-embedded kidney sections were deparaffinized, subjected to antigen retrieval in citrate buffer (pH 6.0), and blocked at room temperature for 1 h. Sections were incubated overnight at 4 °C with anti-PARP1 (ab191217, Abcam, United Kingdom; 1:500) and anti-synaptopodin (sc-515842, Santa Cruz Biotechnology, United States; 1:1000). The next day, Alexa Fluor® 647-conjugated goat anti-mouse IgG H&L (ab150115, Abcam, United Kingdom; 1:1000) and Alexa Fluor® 488-conjugated donkey anti-rabbit IgG H&L (ab150061, Abcam, United Kingdom; 1:1000) were applied for 1 h, followed by nuclear counterstaining with DAPI. Images were captured using a Zeiss LSM 880 confocal laser scanning microscope (Germany), and merged channels were analyzed for PARP1-synaptopodin colocalization.

### TUNEL staining

Podocyte apoptosis in glomeruli was assessed using an *In Situ* Cell Death Detection Kit, Fluorescein (11684817910, Roche, Switzerland). Following deparaffinization and proteinase K treatment, sections were incubated with the TUNEL reaction mixture at 37 °C for 1 h in the dark. Anti-synaptopodin staining (Abcam, United Kingdom) was then performed, followed by Alexa Fluor 594-labeled secondary antibody. Nuclei were counterstained with DAPI. Green TUNEL-positive signals and red synaptopodin labeling were visualized using a Zeiss LSM 880 microscope to assess colocalization.

### Isolation and purification of RBCm

RBCm were isolated from the peripheral blood of 8-week-old C57BL/6J mice by differential centrifugation. Whole blood (500 μL) was collected via retro-orbital puncture into EDTA-coated tubes and centrifuged at 1,000 rpm for 5 min at 4 °C to remove plasma and the buffy coat. Red blood cells were washed three times with ice-cold PBS to eliminate residual serum proteins, then resuspended in an equal volume of 25% PBS and incubated on ice for 1 h to induce hypotonic lysis. Lysed membranes were pelleted by centrifugation at 20,000 rpm for 20 min at 4 °C, yielding a pale pink precipitate (RBCm). The membranes were further processed by intermittent sonication on ice (100 W, 2 min, 2 s on/5 s off) and sequential extrusion through 400 nm and 200 nm polycarbonate membranes using a mini-extruder to produce uniform vesicles. The resulting RBCm suspension was resuspended in PBS and stored at 4 °C until use.

### BMS-α functionalization of the RBC membrane and binding efficiency assessment

To endow the RBCm with active podocyte-targeting capability, the membrane surface was modified using the disulfide-linked lipid-monomer ligand BMS-α. A lipid insertion strategy was employed following previously published methods (Fang et al., 2013) with minor modifications. Briefly, BMS-α was dissolved in anhydrous DMSO to prepare a 1 mg/mL stock solution. The BMS-α solution (1 mg/mL) was resuspended in buffer containing 10 mM Tris (pH 8.0), 0.1 mM EDTA, and a 100-fold molar excess of TCEP, followed by incubation at room temperature for 4 h to complete reduction. After washing and purification, the reduced BMS-α was mixed with an equimolar amount of maleimide-lipid and incubated overnight for conjugation. RBCm proteins (approximately 500 μg total) were resuspended in PBS, and the conjugated BMS-α solution was added to reach a final concentration of 50 μg/mL. The reaction was carried out at 4 °C for 45 min under light-protected conditions to avoid photodegradation. Afterward, unbound BMS-α was removed by centrifugation at 20,000 rpm for 20 min and washed twice with PBS. The resulting BMS-RBCm was used for subsequent nanoparticle coating. To determine the binding efficiency of BMS-α to RBCm, BMS-α was labeled with Cy5 (HY-D0821, MedChemExpress) according to the manufacturer’s instructions prior to membrane modification. Specifically, Cy5 dye (5 mg/mL) was added to the 1 mg/mL BMS-α stock solution and incubated for 60 min at 4 °C with gentle shaking in the dark. After incubation, the Cy5–BMS-α conjugate was purified using a Sephadex G-25 column and washed twice with PBS. The labeled Cy5–BMS-α was then conjugated to RBCm following the aforementioned procedure to generate Cy5–BMS-α–RBCm. Unlabeled BMS-RBCm served as the negative control. Flow cytometry was used to detect the percentage of Cy5-positive RBCm to quantify binding efficiency.

### Preparation of small interfering RNA targeting PARP1-Loaded PLGA nanoparticles (siPARP1-NPs)

siPARP1-NPs were prepared using a modified double emulsion (W/O/W) solvent evaporation method. To improve siRNA encapsulation efficiency and stability, a charge-complexation strategy was employed to form siRNA-polyethyleneimine (PEI) complexes. Briefly, 5 µg of chemically modified PARP1 siRNA was dissolved in 100 µL RNase-free water containing 1 mM EDTA and RNase inhibitor (SUPERase•In, 1 U/µL). Linear PEI (25 kDa) was then added to achieve an N/P ratio of 10, followed by incubation on ice for 15 min to form the inner aqueous phase (W1).

The oil phase (O) was prepared by dissolving 10 mg of PLGA in 1 mL of ethyl acetate and stirring at room temperature for 30 min. Under ice-bath conditions, W1 was slowly added dropwise to the oil phase and emulsified using a probe sonicator (20% amplitude) for 30 s to generate the primary emulsion (W1/O). This emulsion was then added to 2 mL of 1% PVA (W2) and emulsified again for 60 s to form the W1/O/W2 double emulsion. The mixture was stirred at room temperature for 4 h to allow complete solvent evaporation and nanoparticle solidification. NPs were collected by centrifugation at 10,000 rpm for 10 min (4 °C) and washed three times with PBS to remove residual PVA and unencapsulated siRNA. For long-term storage, 5% trehalose was added as a cryoprotectant before lyophilization, and samples were stored at −80 °C.

### Coating of siPARP1-NPs with BMS-RBCm

A 2 mL suspension of siPARP1-NPs (1 mg/mL) was mixed with pre-functionalized BMS-RBCm at a mass ratio of 1:1 (NPs:membrane protein) and stirred at 4 °C for 45 min to promote membrane-nanoparticle fusion. The mixture was then subjected to intermittent sonication (100 W, 5 min, 2 s on/5 s off) in an ice bath to enhance coating efficiency. The resulting hybrid particles were repeatedly extruded (≥10 passes) through a 200 nm polycarbonate membrane to ensure uniform size distribution and complete membrane coverage. The final targeted NPs, designated siPARP1-NPs@RBCm-BMS-α, were stored at 4 °C until use. For extended preservation, 5% trehalose was added before lyophilization and storage at −80 °C.

### Particle size distribution and surface charge analysis

To evaluate the stability of the nanoparticle system under different temperatures, siPARP1-NPs@RBCm-BMS-α was resuspended in PBS and stored at 4 °C or room temperature for 2 weeks. To assess stability in biological media, serum stability assays were performed. Unmodified siPARP1, siPARP1-NPs, and siPARP1-NPs@RBCm-BMS-α were incubated in McCoy’s 5A medium containing 10% FBS at 37 °C for 0, 1, 2, 4, 6, and 24 h. Samples were centrifuged at 20,000 g for 20 min at 4 °C, and the pellets were analyzed by 1% (w/v) agarose gel electrophoresis (Raval et al., 2020). The hydrodynamic diameter and zeta potential of NPs were measured using dynamic light scattering (DLS). Before analysis, samples were diluted to an appropriate concentration, placed in clean quartz cuvettes, and measured at 25 °C. Each sample was tested in triplicate, and results are reported as mean ± standard deviation.

### Morphological characterization

Nanoparticle morphology and membrane coating were visualized using TEM. Samples were applied to carbon-coated copper grids, allowed to adsorb for 5 min, and blotted with filter paper. Negative staining was performed with 2% phosphotungstic acid (pH 7.0), followed by air-drying before imaging on a TEM instrument (e.g., JEOL JEM-2100).

### Evaluation of siRNA encapsulation efficiency

The siPARP1-NPs@RBCm-BMS-α solution obtained after complex formation was centrifuged at 12,000 rpm for 15 min. The supernatant was carefully collected, and the concentration of unbound siRNA was determined at 260 nm using a UV-visible spectrophotometer (Nanodrop 1000, Thermo Scientific, United States). For each nanoparticle subtype, NPs@RBCm-BMS-α without siRNA served as the blank control. The amount of siRNA bound to the NPs was calculated as follows:

siRNA loaded on NPs = initial siRNA amount−free siRNA in supernatant.

The encapsulation efficiency was then determined using the formula:

$$\text{Encapsulation efficiency }\left(\%\right)=\frac{\text{siRNA}\;\text{loaded}\;\text{on}\;\text{nanoparticles}}{\text{initial}\;\text{siRNA}\;\text{amount}}\times 100\%$$

### Assessment of targeting capability (cellular uptake and colocalization)

To investigate whether BMS-α modification enhanced podocyte targeting, both unmodified and BMS-α-modified siPARP1-RBCm/NPs were fluorescently labeled (e.g., Cy5 for siRNA or DiD for membrane labeling) and incubated with cultured podocytes (MPC5 cell line). After 4 h of treatment, cells were rinsed with PBS, fixed, and stained with DAPI to visualize nuclei. Fluorescence distribution and colocalization were examined using laser scanning confocal microscopy, and fluorescence intensity was quantified with ImageJ software.

To verify the specific binding of BMS-α to the MC-1R receptor on podocytes, MPC5 cells were pre-incubated with MC-1R–blocking antibody (2 μg/mL) at 37 °C for 12 h. Then, DiD-labeled siPARP1-NPs@RBCm or siPARP1-NPs@RBCm-BMS-α were added and incubated for 30, 60, or 120 min, followed by flow cytometry analysis of cellular fluorescence intensity.

To assess *in vivo* MC-1R binding capability, siPARP1-NPs@RBCm-BMS-α were labeled with DiI. Normal and DN mice received a tail-vein injection of 100 μL DiI-labeled siPARP1-NPs@RBCm-BMS-α (dissolved in 5% sterile sucrose solution) at a dose of 20 mg/kg. Two hours after injection, mice were sacrificed, kidneys were collected, sectioned, and subjected to immunofluorescence staining. Primary antibody MC-1R (ab236734, Abcam) was used at 1:150, and secondary antibody goat anti-rabbit IgG H&L (Alexa Fluor® 488) was used at 1:1000. After incubation at room temperature for 1 h and PBS washing, fluorescence distribution and colocalization were visualized by confocal laser scanning microscopy (CLSM).

### Statistical analysis

Data are presented as mean ± standard deviation (SD). Statistical significance between groups was assessed using a two-tailed unpaired Student’s t-test or analysis of variance (ANOVA). A *p*-value <0.05 was considered statistically significant. Graphs and statistical analyses were performed using GraphPad Prism 9.0 (GraphPad Software, United States). All experiments were repeated at least three times.

## Results

### Transcriptomic Analysis Reveals PARP1-Mediated regulation of the TGFβ/Smads pathway in HG-Induced injury of MPC5 podocytes

**FIGURE 1:**
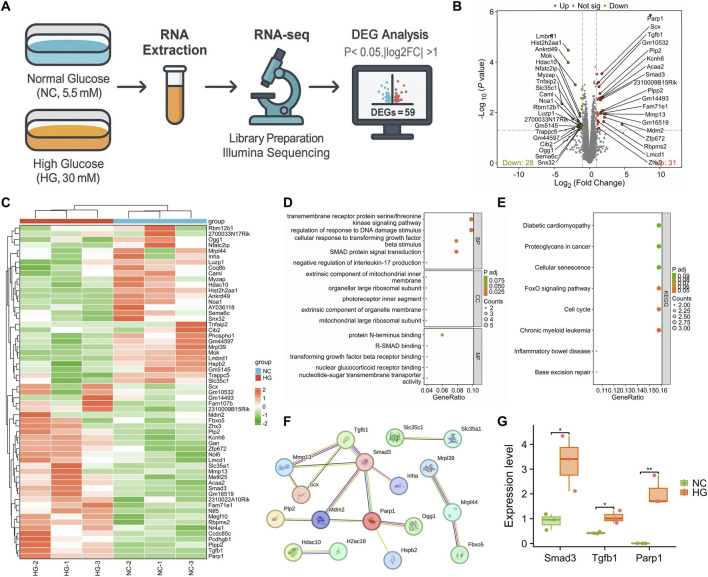
Transcriptomic Analysis Reveals that PARP1 Regulates the TGFβ/Smads Signaling Pathway in HG-Induced MPC5 Podocyte Injury. Note: **(A)** Schematic workflow of RNA sequencing analysis in MPC5 podocytes cultured under normal glucose (NC, 5.5 mM) or HG (HG, 30 mM) conditions (N = 3); **(B)** Volcano plot illustrating the distribution of DEGs (criteria: *p* < 0.05, |log_2_FC| > 1). Green dots represent genes with log_2_FC > 1 and *p* < 0.05, yellow dots represent genes with log_2_FC < −1 and *p* < 0.05, and gray dots represent non-significant genes; **(C)** Heatmap showing the expression profiles of 59 DEGs between the NC and HG groups; **(D,E)** GO and KEGGenrichment analyses of DEGs; **(F)** PPI network constructed using the STRING database; **(G)** Expression levels of PARP1, TGFB1, and Smad3 from transcriptomic data. *p < 0.05; **p < 0.01.

An HG injury model was established using MPC5 podocytes, with cells allocated into a normal glucose group (NC; 5.5 mM glucose) and an HG group (HG; 30 mM glucose). Subsequently, transcriptomic sequencing analysis was performed on both groups (Figure 1A). Differential expression analysis identified 59 genes (*p* < 0.05, |log_2_FC| > 1), including 31 upregulated and 28 downregulated transcripts (Figures 1B,C). GO and KEGG enrichment analyses revealed that these genes were significantly enriched in the TGF-β/Smads signaling pathway, transmembrane receptor serine/threonine kinase signaling, cellular responses to TGF-β stimulus, and R-SMAD binding, among other BP (Figure 1D). KEGG analysis further indicated their involvement in cellular senescence, FoxO signaling, and cell cycle regulation (Figure 1E), suggesting that these signaling axes may play pivotal roles in HG-induced podocyte injury. PPI network analysis demonstrated strong interconnections among PARP1, Smad3, and TGFB1 (Figure 1F). Consistently, transcriptomic data showed significantly increased expression of PARP1, TGFB1, and Smad3 in HG-treated podocytes (Figure 1G).

Collectively, these findings indicate that PARP1 may contribute to HG-induced podocyte injury by modulating the TGFβ/Smads signaling cascade.

### PARP1 inhibition attenuates cytotoxicity, inflammation, and fibrosis in HG-Treated MPC5 podocytes

**FIGURE 2:**
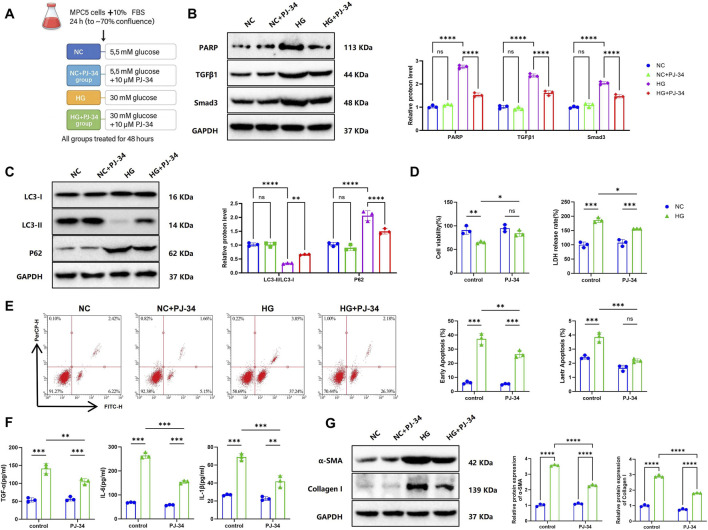
The PARP1 Inhibitor PJ-34 Alleviates HG-Induced Cytotoxicity, Inflammation, and Fibrosis in MPC5 Cells. Note: **(A)** Experimental design and treatment scheme; **(B)** RT-qPCR and WB analysis of PARP1, TGFβ1, and Smad3 expression in MPC5 cells; **(C)** WB analysis of autophagy-related proteins LC3-II/I ratio and P62 expression; **(D)** CCK-8 assay and LDH release assay for cell proliferation and cytotoxicity; **(E)** Flow cytometric analysis of apoptosis; **(F)** ELISA quantification of TGF-α, IL-6, and IL-1β expression; **(G)** WB analysis of fibrotic markers α-SMA and Collagen I. All experiments were performed in triplicate. ns, not significant; *p < 0.05; **p < 0.01; ***p < 0.001; ****p < 0.0001.

To further elucidate the role of PARP1 inhibition, MPC5 cells were divided into four groups: NC, NC + PJ-34, HG, and HG + PJ-34 (Figure 2A). RT-qPCR and WB analyses revealed that HG treatment markedly increased PARP1, TGFβ1, and Smad3 expression compared with NC cells, whereas PJ-34 treatment significantly reduced their expression relative to the HG group, indicating that PJ-34 suppresses activation of the TGFβ/Smads pathway by inhibiting PARP1 (Figure 2B). WB analysis also showed that PJ-34 increased the LC3-II/LC3-I ratio and reduced P62 levels under HG conditions, suggesting restoration of autophagic activity (Figure 2C).

Functional assays demonstrated that HG treatment impaired cell proliferation and increased cytotoxicity, as assessed by CCK-8 and LDH release assays, whereas PJ-34 partially restored proliferation and reduced cytotoxicity (Figure 2D). Flow Cytometry indicated a significant rise in apoptosis in the HG group compared with NC, which was substantially reduced by PJ-34 (Figure 2E). ELISA revealed that PJ-34 lowered TGF-α, IL-6, and IL-1β levels compared with HG (Figure 2F). Furthermore, WB analysis demonstrated that PJ-34 suppressed HG-induced upregulation of α-SMA and collagen I, indicating attenuation of fibrotic responses (Figure 2G).

In summary, the PARP1 inhibitor PJ-34 mitigates HG-induced podocyte toxicity, dampens inflammatory responses, and inhibits fibrosis.

### PARP1 inhibitor PJ-34 attenuates podocyte and glomerular injury in diabetic mice

**FIGURE 3:**
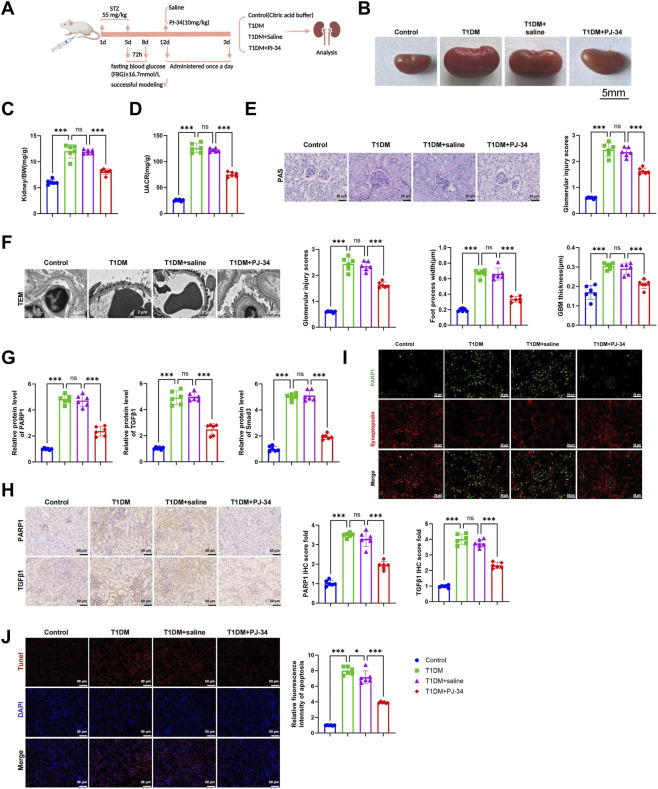
The PARP1 Inhibitor PJ-34 Ameliorates Glomerular Abnormalities and Podocyte Injury in Diabetic Mice. Note: **(A)** Experimental workflow for T1DM model induction and PJ-34 treatment; **(B)** Representative images of mouse kidneys, scale bar: 5 mm; **(C)** Kidney-to-body weight ratio; **(D)** UACR; **(E)** Periodic acid-Schiff (PAS) staining of glomerular morphology, scale bar: 25 μm; **(F)** TEM of GBM thickness and podocyte foot processes, scale bar: 2 μm; **(G)** WB analysis of PARP1, TGFβ1, and Smad3 expression in renal tissues; **(H)** Immunohistochemical staining of PARP1 and TGFβ1 distribution in renal tissues, scale bar: 50 μm; **(I)** IF double staining showing colocalization of PARP1 with the podocyte marker Synaptopodin, scale bar: 25 μm; **(J)** TUNEL staining for podocyte apoptosis and quantification, scale bar: 50 μm. Each group included six mice. ns, not significant; *p < 0.05; ***p < 0.001.

A STZ-induced T1DM mouse model was established and treated with the PARP1 inhibitor PJ-34. Animals were allocated into four groups: Control, T1DM, T1DM + saline, and T1DM + PJ-34 (Figure 3A). Gross examination revealed renal hypertrophy in the T1DM and T1DM + saline groups compared with Controls, whereas kidney morphology improved in the T1DM + PJ-34 group (Figure 3B). Both kidney weight-to-body weight ratio and UACR were markedly elevated in the T1DM and T1DM + saline groups relative to Controls, but significantly reduced following PJ-34 treatment (Figures 3C,D). Periodic acid-Schiff (PAS) staining demonstrated pronounced glomerulosclerosis and podocyte injury in T1DM and T1DM + saline mice, which were alleviated by PJ-34 administration (Figure 3E). TEM revealed marked thickening of the GBM and widespread podocyte foot process effacement, broadening, and blurring of inter-podocyte spaces in T1DM and T1DM + saline mice. In contrast, PJ-34 treatment reduced GBM thickening, partially restored foot process architecture, narrowed foot process width, and diminished fusion, with more clearly delineated structures (Figure 3F).

At the molecular level, WB and immunohistochemistry indicated significant upregulation of PARP1, TGFβ1, and Smad3 in T1DM and T1DM + saline mice compared with Controls, whereas PJ-34 markedly suppressed their expression (Figures 3G,H). IF analysis showed strong colocalization of PARP1 with the podocyte marker synaptopodin in diabetic mice, indicating pronounced PARP1 activation in podocytes under hyperglycemic conditions. PJ-34 reduced PARP1 fluorescence intensity and colocalization with synaptopodin, suggesting inhibition of its expression and activity in podocytes. Moreover, synaptopodin expression in T1DM and T1DM + saline mice was attenuated and diffusely distributed, indicative of cytoskeletal disruption, while PJ-34 partially restored its intensity and continuous linear distribution, reflecting preservation of podocyte structural integrity (Figure 3I). To assess the effect of PJ-34 on podocyte apoptosis, TUNEL staining was performed in combination with synaptopodin IF. T1DM and T1DM + saline mice exhibited a marked increase in TUNEL-positive cells within glomeruli, many colocalizing with synaptopodin, indicating enhanced podocyte apoptosis during diabetic injury. PJ-34 treatment significantly reduced the number of TUNEL-positive podocytes and increased the proportion of synaptopodin-positive cells, suggesting both attenuation of apoptosis and partial recovery of podocyte number (Figure 3J).

Collectively, these findings demonstrate that PJ-34 markedly mitigates glomerular structural abnormalities and podocyte injury in diabetic mice, conferring a protective effect *in vivo*.

### Preparation and characterization of siPARP1-NPs@RBCm-BMS-α

**FIGURE 4:**
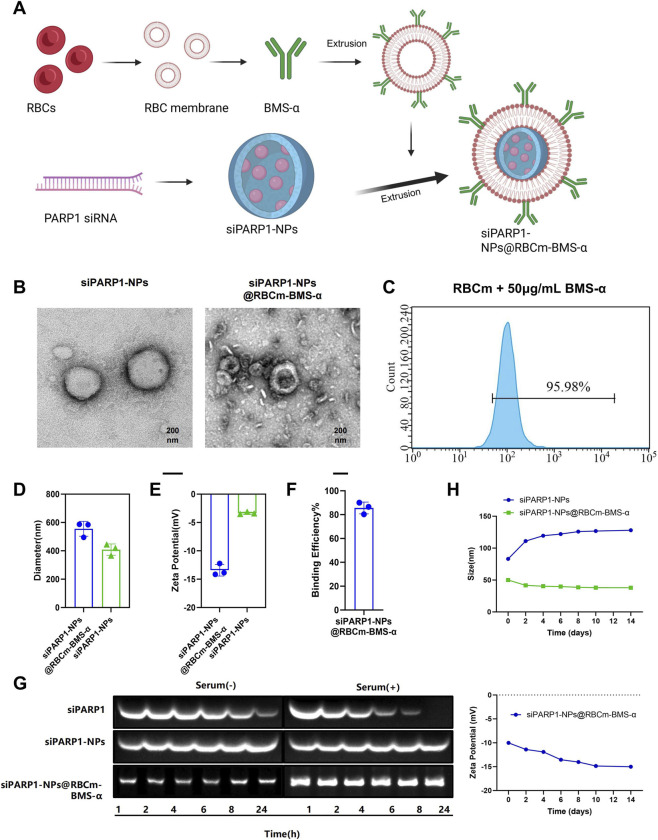
Construction and Physicochemical Characterization of the Targeted siRNA Delivery System siPARP1-NPs@RBCm-BMS-α. Note: **(A)** Schematic illustration of the preparation process of siPARP1-NPs@RBCm-BMS-α, created in BioRender; **(B)** TEM images showing particle morphology and membrane coating of siPARP1-NPs@RBCm-BMS-α (scale bar: 200 nm); **(C)** Flow cytometry results showing the binding efficiency of Cy5-labeled BMS-α to RBCm; **(D)** DLS analysis of particle size distribution for siPARP1-NPs@RBCm-BMS-α and siPARP1-NPs; **(E)** Zeta potential measurements comparing surface charge between siPARP1-NPs@RBCm-BMS-α and siPARP1-NPs; **(F)** Quantitative fluorescence assay of PARP1 siRNA encapsulation efficiency in siPARP1-NPs@RBCm-BMS-α; **(G)** Serum stability of siPARP1-NPs@RBCm-BMS-α assessed by agarose gel electrophoresis; **(H)** Changes in particle size and zeta potential of siPARP1-NPs@RBCm-BMS-α under different storage conditions (4 °C and room temperature). All experiments were performed in triplicate.

We developed a novel podocyte-targeted siRNA delivery platform, designated siPARP1-NPs@RBCm-BMS-α, through a three-step process. First, mouse RBCm were isolated via differential centrifugation and functionalized with the disulfide-bond-containing podocyte-targeting small molecule BMS-α using a lipid insertion strategy. Second, siPARP1-NPs were fabricated by a double-emulsion solvent evaporation method. Finally, the BMS-α-modified RBC membranes were coated onto the PLGA NPs, forming a core-shell nanostructure (Figure 4A). TEM revealed that siPARP1-NPs@RBCm-BMS-α exhibited a near-spherical morphology with a uniformly distributed thin membrane coating and well-defined edges. Compared with uncoated siPARP1-NPs, the coated NPs displayed a distinct low-electron-density corona, confirming successful RBC membrane encapsulation. The NPs were well dispersed without noticeable aggregation or collapse, indicating excellent structural stability and membrane integrity (Figure 4B). Flow cytometry analysis demonstrated that the binding efficiency of BMS-α to the RBCm reached 95.98% (Figure 4C). DLS analysis showed an average particle size of 556.2 ± 7.6 nm and a PDI of 0.19, reflecting a narrow size distribution and good dispersity. In comparison, uncoated siPARP1-NPs measured 408.3 ± 5.4 nm, suggesting an increase in size due to membrane coating (Figure 4D). Zeta potential measurements demonstrated a surface charge of −13.4 ± 1.8 mV for coated NPs versus −3.44 ± 0.6 mV for uncoated particles, further supporting successful coating and indicating favorable stability and biocompatibility (Figure 4E).

Fluorescence quantification revealed that, out of the initial 5 μg PARP1 siRNA, only 0.72 μg remained unencapsulated during PLGA core fabrication, corresponding to an initial siRNA loading efficiency of 85.6% ± 3.2%. This result indicates the high loading capacity of the PLGA core. Following RBC membrane coating and BMS-α functionalization, the final siPARP1-NPs@RBCm-BMS-α retained stable physicochemical properties and effective biological activity, supporting the delivery capability of the nanoplatform (Figure 4F). Serum stability testing showed that encapsulated siPARP1 remained structurally intact after 24 h of incubation in serum (Figure 4G). Temperature stability studies demonstrated that siPARP1-NPs@RBCm-BMS-α maintained good stability at both 4 °C and room temperature (Figure 4H).

Collectively, siPARP1-NPs@RBCm-BMS-α demonstrated robust structural stability and high PARP1 siRNA loading efficiency.

### *In Vitro* and *In Vivo* Safety Evaluation of siPARP1-NPs@RBCm-BMS-α

**FIGURE 5:**
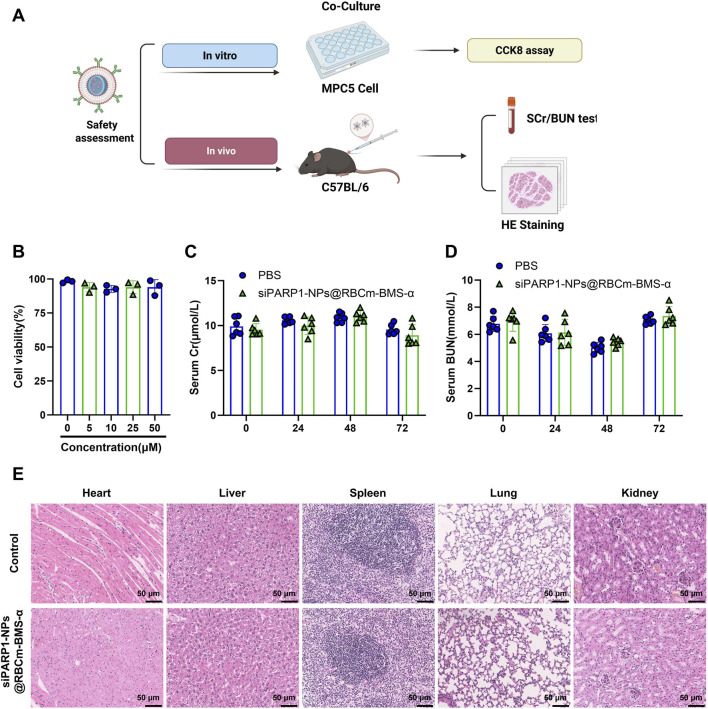
*In Vitro* and *In Vivo* Safety Evaluation of siPARP1-NPs@RBCm-BMS-α. Note: **(A)** Workflow for safety assessment of the siPARP1-NPs@RBCm-BMS-α nanoplatform, created in BioRender; **(B)** CCK-8 assay of MPC5 podocyte viability following treatment with varying concentrations of siPARP1-NPs@RBCm-BMS-α; **(C,D)** ELISA quantification of SCr and BUN levels at different time points after intravenous injection of siPARP1-NPs@RBCm-BMS-α in C57BL/6 mice; **(E)** H&E staining of heart, liver, spleen, lung, and kidney tissues from each group (scale bar: 50 μm). Cellular experiments were performed in triplicate; animal experiments included six mice per group.

A comprehensive safety assessment of siPARP1-NPs@RBCm-BMS-α was performed both *in vitro* and *in vivo* (Figure 5A). *In vitro*, MPC5 podocytes were incubated with various concentrations (0, 5, 10, 25, and 50 μM) of siPARP1-NPs@RBCm-BMS-α. CCK-8 assays showed no significant reduction in cell viability at any concentration compared with controls, indicating high biocompatibility and low cytotoxicity in podocytes (Figure 5B). Even at 50 μM, cell viability remained stable without evident toxic effects, suggesting that the nanoplatform is safe within the effective dose range.

For *in vivo* evaluation, C57BL/6 mice received intraperitoneal injections of siPARP1-NPs@RBCm-BMS-α (20 mg/kg). Serum creatinine (SCr) and blood urea nitrogen (BUN) levels measured at 24, 48, and 72 h post-injection did not differ significantly from saline-treated controls, indicating no acute renal injury (Figures 5C,D). Histopathological examination of the heart, liver, spleen, lung, and kidney via hematoxylin and eosin (H&E) staining revealed intact tissue architecture without necrosis, edema, hemorrhage, or inflammatory infiltration (Figure 5E). Collectively, these findings demonstrate that siPARP1-NPs@RBCm-BMS-α exhibits excellent biosafety and organ tolerance both *in vitro* and *in vivo*, supporting its suitability for therapeutic application in DN.

### Targeting efficiency and biodistribution of siPARP1-NPs@RBCm-BMS-α

**FIGURE 6:**
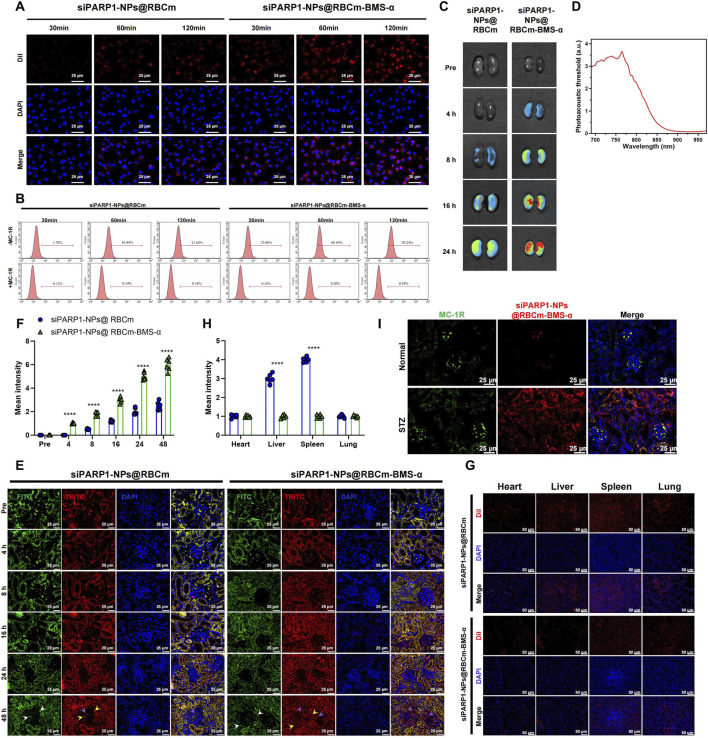
*In Vitro* and *In Vivo* Targeting Capability of siPARP1-NPs@RBCm-BMS-α Towards Podocytes and Renal Tissue. Note: **(A)** CLSM images of MPC5 podocytes after co-incubation with DiI-labeled siPARP1-NPs@RBCm or siPARP1-NPs@RBCm-BMS-α (scale bar: 25 μm); **(B)** Flow Cytometry analysis of intracellular fluorescence levels in MPC5 podocytes at different incubation times. The first row shows cells without MC-1R–blocking antibody treatment; the second row shows cells pre-treated with the MC-1R–blocking antibody; **(C,D)** PAI assessment of renal distribution intensity of DiR-labeled NPs in T1DM mice; **(E,F)** CLSM images of kidney sections from T1DM mice showing localization of DiI-labeled NPs, with merged channels distinguishing autofluorescence from accurate red signal (scale bar: 25 μm); **(G,H)** CLSM analysis of nanoparticle distribution in major organs, evaluating biodistribution and clearance by the liver and spleen (scale bar: 50 μm); **(I)** Representative *in vivo* colocalization images showing siPARP1-NPs@RBCm-BMS-α (DiI-labeled, red) with MC-1R (Alexa Fluor® 488-labeled, green). Scale bar: 25 μm. Six mice per group; experiments were conducted in triplicate. ****p < 0.0001, between-group comparison.

The targeting capacity of siPARP1-NPs@RBCm-BMS-α was first assessed in the mouse podocyte line MPC5. Following 120 min of co-incubation with DiI-labeled siPARP1-NPs@RBCm, punctate red fluorescence was observed on the podocyte membrane, and flow cytometry showed that only 21.4% of cells were fluorescence-positive. In contrast, after 60 min of co-incubation with DiI-labeled siPARP1-NPs@RBCm-BMS-α, 67.3% of cells exhibited membrane-associated ring-like fluorescence. After 120 min, strong intracellular fluorescence was observed, with 85.04% fluorescence-positive cells. Upon addition of the MC-1R–neutralizing antibody, the proportion of fluorescence-positive cells decreased significantly (Figures 6A,B). These results confirm that BMS-α functionalization confers specific podocyte-recognition capability.

The targeting performance was further validated in T1DM mice. siPARP1-NPs@RBCm-BMS-α were labeled with the photosensitizer DiR, and renal targeting was monitored using a photoacoustic imaging (PAI) system under anesthesia (maintained with 1.5% isoflurane in oxygen). Mice receiving siPARP1-NPs@RBCm exhibited only faint renal photoacoustic signals at 8 h, with gradual increases at 16 h and 24 h. In contrast, siPARP1-NPs@RBCm-BMS-α generated a robust signal as early as 8 h, which further intensified at 16 h and persisted to 24 h (Figures 6C,D), indicating rapid and sustained renal accumulation.

CLSM provided further insight into renal cellular localization. Frozen kidney sections from T1DM mice injected with DiI-labeled siPARP1-NPs@RBCm or siPARP1-NPs@RBCm-BMS-α were examined. To distinguish tissue autofluorescence (FITC channel, green; white arrows) and DiI red fluorescence (TRITC channel), merged channels were used to filter out autofluorescence (yellow arrows indicate red autofluorescence). In the siPARP1-NPs@RBCm group, only sparse punctate DiI signals were detected in glomeruli at 4 h and 8 h, with minimal increase over 48 h. Conversely, siPARP1-NPs@RBCm-BMS-α treatment resulted in markedly enhanced glomerular fluorescence by 8 h, which intensified at 16 h and was sustained without decay to 48 h (Figures 6E,F), demonstrating efficient glomerular targeting. Biodistribution analysis revealed low and comparable DiI fluorescence in the heart and lungs across both groups. However, the siPARP1-NPs@RBCm group showed stronger fluorescence in the liver and spleen compared with siPARP1-NPs@RBCm-BMS-α (Figures 6G,H), indicating that BMS-α modification reduces hepatic and splenic sequestration while enhancing renal specificity.

Collectively, these results demonstrate that siPARP1-NPs@RBCm-BMS-α achieves efficient and selective targeting of podocytes in the kidney.

### siPARP1-NPs@RBCm-BMS-α Attenuates HG-Induced Cytotoxicity, Inflammation, and Fibrosis in MPC5 Cells

**FIGURE 7:**
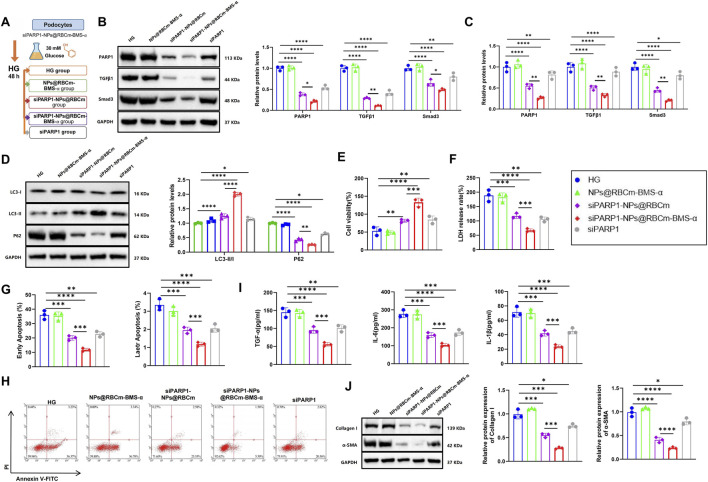
siPARP1-NPs@RBCm-BMS-α Attenuates HG-Induced Toxicity, Inflammation, and Fibrosis in MPC5 Podocytes. Note: **(A)** Schematic of the experimental workflow for establishing the HG-induced podocyte injury model and subsequent siPARP1-NPs@RBCm-BMS-α treatment; **(B,C)** RT-qPCR and WB analysis of PARP1, TGFβ1, and Smad3 expression in MPC5 cells; **(D)** WB analysis of autophagy-related proteins, including the LC3-II/I ratio and P62 expression; **(E,F)** Assessment of cell viability and cytotoxicity using the CCK-8 assay and LDH release assay; **(G,H)** Flow cytometric analysis of podocyte apoptosis rates; **(I)** ELISA quantification of TGF-α, IL-6, and IL-1β levels; **(J)** WB analysis of fibrosis markers α-SMA and Collagen I. Data are presented as mean ± SD from three independent experiments. *p < 0.05, **p < 0.01, ***p < 0.001, ****p < 0.0001 versus the indicated group.

The therapeutic potential of siPARP1-NPs@RBCm-BMS-α in HG-induced podocyte injury was subsequently evaluated. HG-treated podocytes were exposed to 50 μM siPARP1-NPs@RBCm-BMS-α and assigned to four groups: HG, NPs@RBCm-BMS-α, siPARP1-NPs@RBCm, siPARP1-NPs@RBCm-BMS-α, and siPARP1 (Figure 7A). RT-qPCR and WB analyses showed no significant changes in PARP1, TGFβ1, or Smad3 expression in the NPs@RBCm-BMS-α group compared with the HG group. siPARP1, siPARP1-NPs@RBCm, and siPARP1-NPs@RBCm-BMS-α markedly reduced the expression of these genes, with the most pronounced suppression observed in the siPARP1-NPs@RBCm-BMS-α group (Figures 7B,C). WB of autophagy markers revealed that LC3-II/LC3-I ratios and P62 levels remained unchanged in the NPs@RBCm-BMS-α group relative to HG. In contrast, siPARP1, siPARP1-NPs@RBCm, and siPARP1-NPs@RBCm-BMS-α significantly increased LC3-II/LC3-I ratios and reduced P62 expression, with siPARP1-NPs@RBCm-BMS-α again producing the most potent effects, suggesting restored autophagic activity (Figure 7D).

Functionally, CCK-8 and LDH release assays demonstrated that cell viability and cytotoxicity were unaffected in the NPs@RBCm-BMS-α group compared with HG, whereas siPARP1, siPARP1-NPs@RBCm, and siPARP1-NPs@RBCm-BMS-α significantly increased viability and reduced cytotoxicity, with the latter showing superior protection (Figures 7E,F). Flow Cytometry confirmed that apoptosis levels remained unchanged in the NPs@RBCm-BMS-α group but were markedly reduced in siPARP1, siPARP1-NPs@RBCm, and siPARP1-NPs@RBCm-BMS-α groups, with concomitant increases in cell survival, again most notable in the BMS-α-modified group (Figures 7G,H). ELISA assays showed no significant differences in TGF-α, IL-6, or IL-1β levels between the NPs@RBCm-BMS-α and HG groups. However, siPARP1, siPARP1-NPs@RBCm, and siPARP1-NPs@RBCm-BMS-α groups exhibited significant reductions in these inflammatory mediators, with siPARP1-NPs@RBCm-BMS-α producing the steepest decline (Figure 7I). Similarly, WB revealed no change in α-SMA and Collagen I levels in the NPs@RBCm-BMS-α group compared with HG, but siPARP1, siPARP1-NPs@RBCm, and siPARP1-NPs@RBCm-BMS-α significantly downregulated these fibrotic markers, with the most pronounced effect again observed in the latter (Figure 7J).

In summary, siPARP1-NPs@RBCm-BMS-α effectively mitigates cytotoxicity, suppresses inflammation, and inhibits fibrosis in HG-induced podocyte injury, offering a promising molecular intervention strategy for DN.

### siPARP1-NPs@RBCm-BMS-α attenuates podocyte and glomerular injury in DN mice

**FIGURE 8:**
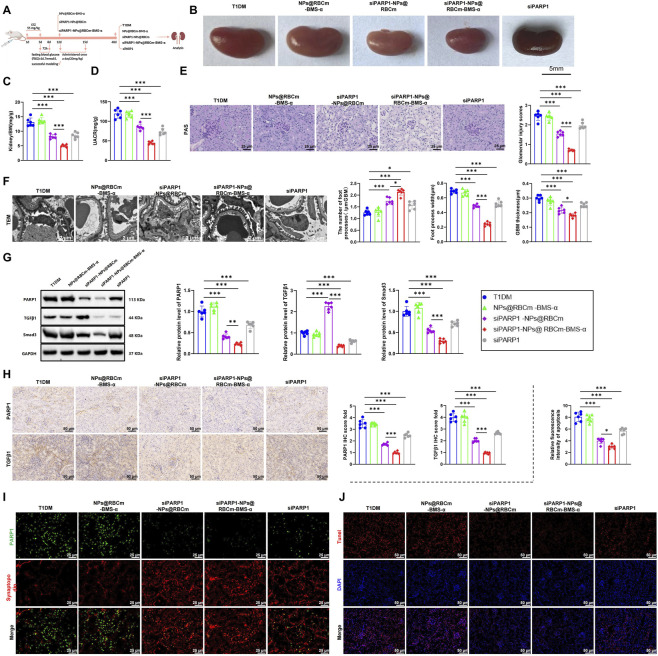
siPARP1-NPs@RBCm-BMS-α Alleviates Glomerular and Podocyte Injury in a DN Mouse Model. Note: **(A)** Experimental design showing STZ-induced T1DM mouse model and subsequent treatment with different nanoparticle systems; **(B)** Representative images of kidney gross morphology (scale bar: 5 mm); **(C,D)** Kidney-to-body weight ratio and UACR for renal function evaluation; **(E)** PAS staining to assess glomerular structural alterations (scale bar: 25 μm); **(F)** TEM analysis of GBM thickness and podocyte foot process morphology (scale bar: 2 μm); **(G,H)** WB and immunohistochemistry for PARP1, TGFβ1, and Smad3 expression and localization in kidney tissue (scale bar: 50 μm); **(I)** Double IF staining for colocalization of PARP1 and Synaptopodin (scale bar: 25 μm); **(J)** TUNEL staining to evaluate podocyte apoptosis and quantify apoptotic cells (scale bar: 50 μm). Data are expressed as mean ± SD from three independent experiments, with n = 6 mice per group. *p < 0.05, **p < 0.01, ***p < 0.001 versus the indicated group.

We next evaluated the therapeutic efficacy of siPARP1-NPs@RBCm-BMS-α in mitigating podocyte and glomerular damage in a murine model of DN. T1DM was induced in mice via STZ injection, and animals were assigned to four groups: T1DM, NPs@RBCm-BMS-α, siPARP1-NPs@RBCm, and siPARP1-NPs@RBCm-BMS-α. Treatments were administered intraperitoneally at 20 mg/kg, as illustrated in the experimental scheme (Figure 8A). Macroscopic kidney examination revealed no significant change in kidney size in the NPs@RBCm-BMS-α group compared with the T1DM group, whereas both siPARP1-containing formulations improved kidney size, with siPARP1-NPs@RBCm-BMS-α showing the most significant reduction (Figure 8B). Consistently, kidney weight-to-body weight ratios and UACR were markedly reduced in the siPARP1-NPs@RBCm and siPARP1-NPs@RBCm-BMS-α groups, with the latter achieving the most pronounced effect, while the NPs@RBCm-BMS-α group showed no significant improvement (Figures 8C,D). Histological assessment using periodic acid-Schiff (PAS) staining demonstrated severe mesangial expansion and glomerular hypertrophy in T1DM mice. No appreciable improvement was observed in the NPs@RBCm-BMS-α group, whereas siPARP1-NPs@RBCm partially alleviated these abnormalities, and siPARP1-NPs@RBCm-BMS-α achieved the most substantial amelioration (Figure 8E). TEM further revealed that both siPARP1-containing formulations attenuated GBM thickening, reduced foot process width, and increased foot process number, with siPARP1-NPs@RBCm-BMS-α eliciting the most marked restoration (Figure 8F).

At the mechanistic level, WB and immunohistochemistry indicated that NPs@RBCm-BMS-α had no significant effect on renal expression of PARP1, TGFβ1, or Smad3. In contrast, both siPARP1-NPs@RBCm and siPARP1-NPs@RBCm-BMS-α significantly downregulated these molecules, with the latter producing the most potent suppression (Figures 8G,H). IF analysis showed robust colocalization of PARP1 with the podocyte marker synaptopodin in T1DM kidneys, which was markedly diminished following siPARP1-NPs@RBCm-BMS-α treatment, indicating effective inhibition of aberrant PARP1 activation in podocytes (Figure 8I). TUNEL staining further demonstrated that siPARP1-containing formulations significantly reduced podocyte apoptosis and increased synaptopodin-positive cell numbers, again with the most significant improvement observed in the siPARP1-NPs@RBCm-BMS-α group, underscoring its potent anti-apoptotic and podocyte-protective effects (Figure 8J).

Collectively, these findings demonstrate that siPARP1-NPs@RBCm-BMS-α selectively targets podocytes to suppress PARP1 expression and modulate the TGFβ/Smads pathway, thereby markedly alleviating podocyte injury and glomerular structural damage in DN mice. This targeted delivery strategy shows strong promise for therapeutic intervention in kidney diseases.

## Discussion

**FIGURE 9:**
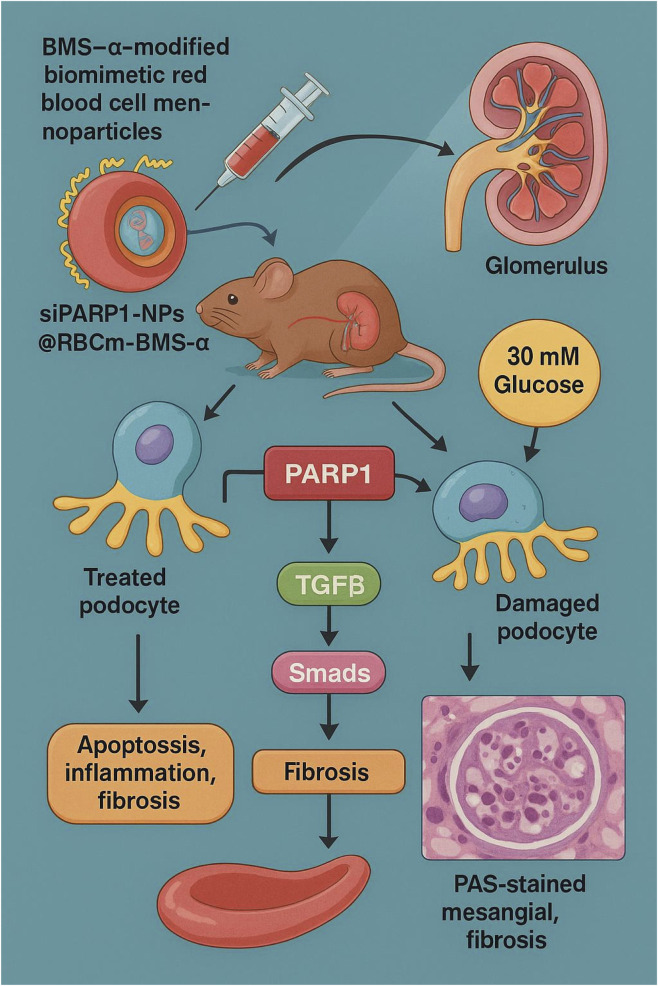
Schematic Representation of the Molecular Mechanism by which siPARP1-NPs@RBCm-BMS-α Mitigates Podocyte Injury and Improves DN Outcomes.

This study systematically elucidates the pivotal role of PARP1 in mediating podocyte injury under hyperglycemic conditions through activation of the TGFβ/Smads signaling pathway. We further report, for the first time, the development of a siPARP1-NPs@RBCm-BMS-α specifically targeting podocytes, enabling efficient delivery of PARP1 siRNA. From mechanistic investigations to *in vivo* validation, our findings substantiate the therapeutic potential of this strategy in DN. Transcriptomic profiling, combined with enrichment analysis of DEGs, revealed marked upregulation of PARP1 and a strong correlation with TGFβ1/Smad3 signaling. Functional inhibition—achieved via both a selective pharmacological inhibitor and siRNA silencing—confirmed its pathogenic role and supported the rationale for our delivery platform. The integration of *in vitro* and *in vivo* evidence establishes PARP1 as a compelling therapeutic target in DN (Figure 9).

As a key DNA damage sensor and repair enzyme, PARP1 has historically been studied in oncology and neurodegenerative disorders (van Beek et al., 2021; Morone and Grimaldi, 2024). More recent work has implicated its pathogenic activity in diabetic retinopathy and cardiomyopathy (Sun et al., 2021; Qin et al., 2016). However, its role in DN—particularly within podocytes, a cell type central to glomerular filtration barrier integrity—remains poorly defined (Szabó et al., 2006). Here, transcriptomic and colocalization analyses demonstrated significant hyperglycemia-induced PARP1 overexpression localized to Synaptopodin-positive regions, providing direct spatial evidence for its involvement. This finding expands the known pathological repertoire of PARP1 in metabolic disease and implicates it as a potential contributor to glomerular barrier disruption.

TGFβ/Smads signaling is a canonical driver of fibrotic remodeling in DN, and identification of its upstream modulators has long been a focus of research (Wang et al., 2021). Through GO and KEGG enrichment analyses and PPI network construction, we place PARP1 within the regulatory hierarchy of the TGFβ/Smads axis for the first time. PARP1 markedly influenced the expression and activation of TGFβ1 and Smad3, and both PJ-34 treatment and siRNA knockdown effectively suppressed pathway activation. These findings suggest that PARP1 may regulate TGFβ transcription factor activity in the nucleus or indirectly amplify upstream signaling through oxidative stress. This work extends the TGFβ regulatory network and introduces a novel intervention node within this classical axis.

We further delineated the involvement of PARP1 in podocyte autophagy, inflammation, and apoptosis. Under hyperglycemic stress, PARP1 activation coincided with suppressed autophagic flux (LC3-II/I↓, P62↑), elevated expression of pro-inflammatory cytokines (IL-6, IL-1β↑), and increased apoptotic cell fractions. Inhibition of PARP1 reversed these changes, underscoring the breadth and integrative nature of its deleterious effects. These outcomes may be mediated through NAD^+^ depletion, Nuclear Factor kappa B (NF-κB) activation, or Sirtuin 1 (SIRT1) suppression, warranting further investigation using metabolomics coupled with chromatin immunoprecipitation assays.

Through comparative pharmacological and genetic interventions, this study established the causal role of PARP1 in podocyte injury. Although PJ-34 demonstrated efficacy, its systemic action predisposes to adverse effects. In contrast, the siPARP1-NPs@RBCm-BMS-α delivery platform exhibited high target specificity, low toxicity, and efficient siRNA encapsulation, producing superior biological outcomes in both cultured podocytes and T1DM mouse models compared with conventional non-targeted delivery. This multi-pronged validation approach strengthened the robustness of the findings and offers a conceptual framework for future therapeutic design.

This study introduces several technical innovations in the design of a nanodrug delivery system. Using RBCm as a biomimetic scaffold combined with the podocyte-targeting molecule BMS-α, we constructed a nanosystem that integrates active targeting, immune evasion, and high delivery efficiency. In both *in vitro* and *in vivo* biodistribution experiments, this system demonstrated stable glomerular targeting while avoiding nonspecific hepatic and splenic accumulation, thereby addressing long-standing challenges of poor siRNA delivery efficiency and limited tissue retention. The encapsulation efficiency of siRNA initially loaded into the PLGA core reached 85.6%, and the system maintained persistent renal fluorescence during prolonged circulation, indicating excellent delivery stability and bioavailability. Previous studies have shown that BMS-α binds specifically to the melanocortin-1 receptor (MC-1R) on podocytes (Fan et al., 2023). Here, we further validated BMS-α–mediated targeting through *in vitro* antibody-blocking assays and *in vivo* colocalization studies in T1DM mice. The interaction between nanoparticles and their cellular receptors is critical for achieving receptor-mediated targeting and subsequent therapeutic efficacy *in vivo*. For example, Bai Q et al. developed a scavenger receptor class A (SR-A)–targeted spherical nucleic acid nanoparticle system and demonstrated enhanced *in vivo* plaque delivery with receptor-relevant cellular colocalization and mechanistic validation (Bai et al., 2022). Similarly, Liu et al. systematically elucidated dopamine receptor (D2DR)-mediated binding and cellular uptake of polydopamine-coated nanoparticles, providing both *in vitro* and *in vivo* evidence supporting receptor-dependent biodistribution and cell-specific internalization (Liu et al., 2021). In line with these precedents, colocalization analysis between the putative podocyte receptor MC-1R and siPARP1-NPs@RBCm-BMS-α in kidney sections would further strengthen the *in vivo* targeting claim, and receptor-blocking/competition experiments could provide additional specificity evidence.

Generally, particles larger than 200 nm are rapidly cleared by the liver and spleen, whereas the slit diaphragm of the glomerular filtration barrier is approximately 40 nm, and the barrier itself permits the passage of nanoparticles smaller than 10 nm (Chan et al., 2023). DLS analysis (Figure 4C) showed that the average size of the entire nanoparticle was 556 nm, which is theoretically too large for systemic delivery to podocytes. However, both *in vitro* and *in vivo* experimental results in the present study demonstrate that this nanodrug delivery system is capable of crossing the glomerular filtration barrier and reaching podocytes. One possible explanation is that the system is modified with the podocyte-targeting ligand BMS-α. Another contributing factor may be the enlargement of filtration barrier gaps in diseased kidneys, which reduces steric hindrance to nanoparticle transport (Shang et al., 2024). In addition, previous studies have reported that relatively large nanoparticles (approximately 300–400 nm in diameter) can be systemically transported to the kidney and subsequently internalized by podocytes via endocytosis (Williams et al., 2018; Guo et al., 2022).

Previous studies have demonstrated that relatively large nanoparticles (>100 nm) can also enter the kidney via secretion from peritubular capillaries through renal tubular epithelial cells. Specifically, after nanoparticles larger than 100 nm pass through the efferent arteriole of the glomerulus and enter the peritubular capillaries, they are driven by intranephron pressure and the strong absorptive forces of the capillary network. These nanoparticles are first transported into the tubular system via exocytosis across peritubular capillary endothelial cells and subsequently undergo transcellular transport through endocytosis by proximal tubular epithelial cells (Huang et al., 2021; Deng et al., 2019). In the present study, the coated nanoparticles, owing to their relatively large diameter and surface modification with the podocyte-targeting ligand BMS-α that recognizes the MC-1R receptor, may account for the delayed renal accumulation observed *in vivo*. Specifically, coated nanoparticles began to accumulate in the renal pelvis approximately 16 h after injection, with enhanced signal intensity and widespread distribution throughout the entire mouse kidney observed at 24 h post-injection.

In terms of structural and functional protection, siPARP1-NPs@RBCm-BMS-α effectively restored podocyte synaptopodin expression, alleviated glomerular basement membrane thickening and foot process effacement, and reduced the proportion of TUNEL-positive cells, thereby markedly improving glomerular architecture in T1DM mice. Notably, the reduced colocalization of synaptopodin and PARP1 indicates a well-defined molecular target and a stable protective effect, suggesting that this delivery system exhibits good adaptability within the complex renal microenvironment. Compared with existing therapeutic approaches such as interferon-based therapies or inhibitors of the renin–angiotensin system, this strategy demonstrates distinct advantages in terms of molecular target specificity and delivery precision.

From a safety perspective, siPARP1-NPs@RBCm-BMS-α did not affect MPC5 podocyte viability across a range of concentrations. Following administration in mice, SCr and BUN levels remained unchanged, and H&E staining of major organs showed no histopathological abnormalities. These results indicate excellent biocompatibility and *in vivo* tolerability, supporting the platform’s suitability for preclinical development. Researchers have previously designed the podocyte-targeted DTsiANp/HDTC4 delivery system to transport HDAC4 siRNA into DKD rat models, producing significant reductions in blood glucose, urinary albumin excretion (UAER), mesangial cell proliferation, and glomerulosclerosis (Raval et al., 2019). Another study developed folate-modified PLGA nanoparticles (FA-PLGA NPs) for targeted delivery of TLR4 siRNA via the folate receptor (FR), effectively improving mesangial matrix expansion, proteinuria, serum creatinine, BUN, and albumin levels in DN mice (Wang et al., 2025). Compared with existing siRNA delivery platforms, this work is the first to integrate podocyte pathophysiology, transcriptomic profiling, and targeted delivery technology to precisely align the molecular target with the delivery route. Current siRNA research in nephrology remains largely dependent on AAV vectors or cationic polymers, which suffer from low efficiency, high cytotoxicity, and poor tissue specificity. In contrast, our siPARP1-NPs@RBCm-BMS-α system demonstrates clear advantages in encapsulation efficiency, targeting specificity, toxicity mitigation, and *in vivo* therapeutic efficacy. Furthermore, its favorable biodistribution profile suggests potential applicability to other kidney disease subtypes—such as mesangial proliferative glomerulonephritis, focal segmental glomerulosclerosis, and minimal change disease—as well as RNA-based modulation of additional renal signaling pathways including NF-κB, PI3K/Akt, and JAK–STAT. These features underscore the strong translational potential of the platform.

In summary, this study provides the first systematic demonstration of the pathogenic role of PARP1 in DN-associated podocyte injury, revealing that PARP1 promotes inflammation, autophagy impairment, and apoptosis via activation of the TGFβ/Smads signaling pathway. We further devised an innovative delivery platform—siPARP1-NPs@RBCm-BMS-α—that enables efficient, precise, and low-toxicity silencing of PARP1, thereby markedly improving glomerular function and podocyte integrity in diabetic mice. This strategy holds substantial value for both basic research and clinical translation and could be extended to other kidney diseases and RNA-based therapeutics. Nevertheless, certain limitations remain, including the need for long-term toxicity assessment, refinement of the safety window, and validation in clinical samples. Furthermore, the direct molecular interplay between PARP1 and TGFβ signaling warrants deeper investigation. Future work employing transgenic animal models and kidney organoids may advance mechanistic understanding and provide a stronger theoretical and technical foundation for the precise RNA-based treatment of DN.

## Data Availability

The original contributions presented in the study are included in the article/Supplementary Material, further inquiries can be directed to the corresponding author.

## Glossary

α-SMA: Alpha-Smooth Muscle Actin
ANOVA: Analysis of Variance
BCA: Bicinchoninic Acid
BP: Biological Process
BMS-α: Bone Morphogenetic Protein-derived Podocyte-Targeting Peptide α
BUN: Blood Urea Nitrogen
CC: Cellular Component
CCK-8: Cell Counting Kit-8
CLSM: Confocal Laser Scanning Microscopy
DAB: 3,3′-Diaminobenzidine
DEGs: Differentially Expressed Genes
DLS: Dynamic Light Scattering
DN: Diabetic Nephropathy
ECL: Enhanced Chemiluminescence
ELISA: Enzyme-Linked Immunosorbent Assay
ESRD: End-Stage Renal Disease
FBS: Fetal Bovine Serum
FITC: Fluorescein Isothiocyanate
GO: Gene Ontology
GBM: Glomerular Basement Membrane
HG: High-Glucose
H&E: Hematoxylin and Eosin
HRP: Horseradish peroxidase
IF: Immunofluorescence
KEGG: Kyoto Encyclopedia of Genes and Genomes
LDH: Lactate Dehydrogenase
MF: Molecular Function
MPC5: Mouse Podocyte Clone 5
NF-κB: Nuclear Factor Kappa B
NPs: Nanoparticles
PAI: Photoacoustic Imaging
PARP1: Poly(ADP-Ribose) Polymerase 1
PAS: Periodic Acid-Schiff
PBS: Phosphate-Buffered Saline
PEI: Polyethyleneimine
PLGA: Poly(Lactic-Co-Glycolic Acid)
PPI: Protein-Protein Interaction
PVDF: Polyvinylidene Difluoride
RBCm: Red Blood Cell Membrane
RNAi: RNA Interference
RT-qPCR: Reverse Transcription Quantitative Polymerase Chain Reaction
scRNA-seq: Single-cell RNA Sequencing
SCr: Serum Creatinine
SDS-PAGE: Sodium Dodecyl Sulfate-Polyacrylamide Gel Electrophoresis
siPARP1-NPs: Small Interfering RNA Targeting PARP1-Loaded PLGA Nanoparticles
siPARP1-NPs@RBCm-BMS-α: Small Interfering RNA Targeting PARP1-Loaded Nanoparticles Coated with Red Blood Cell Membrane and Functionalized with the Podocyte-Targeting Ligand BMS-α
siRNA: Small Interfering RNA
SIRT1: Sirtuin 1
STZ: Streptozotocin
T1DM: Type 1 Diabetes Mellitus
TEM: Transmission Electron Microscopy
TGF-β: Transforming Growth Factor-β
UACR: Urinary Albumin-to-Creatinine Ratio
WB: Western blot.
