# Supplementary figures and images for: Bioengineered exosome-mRNA hybrids: a breakthrough in targeted miRNA delivery for diabetic kidney fibrosis therapy (part 1 of 3)
Source: Front Bioeng Biotechnol. 2026 Mar 2;14:1709588. doi: 10.3389/fbioe.2026.1709588 (PMC12989603; doi:10.3389/fbioe.2026.1709588)

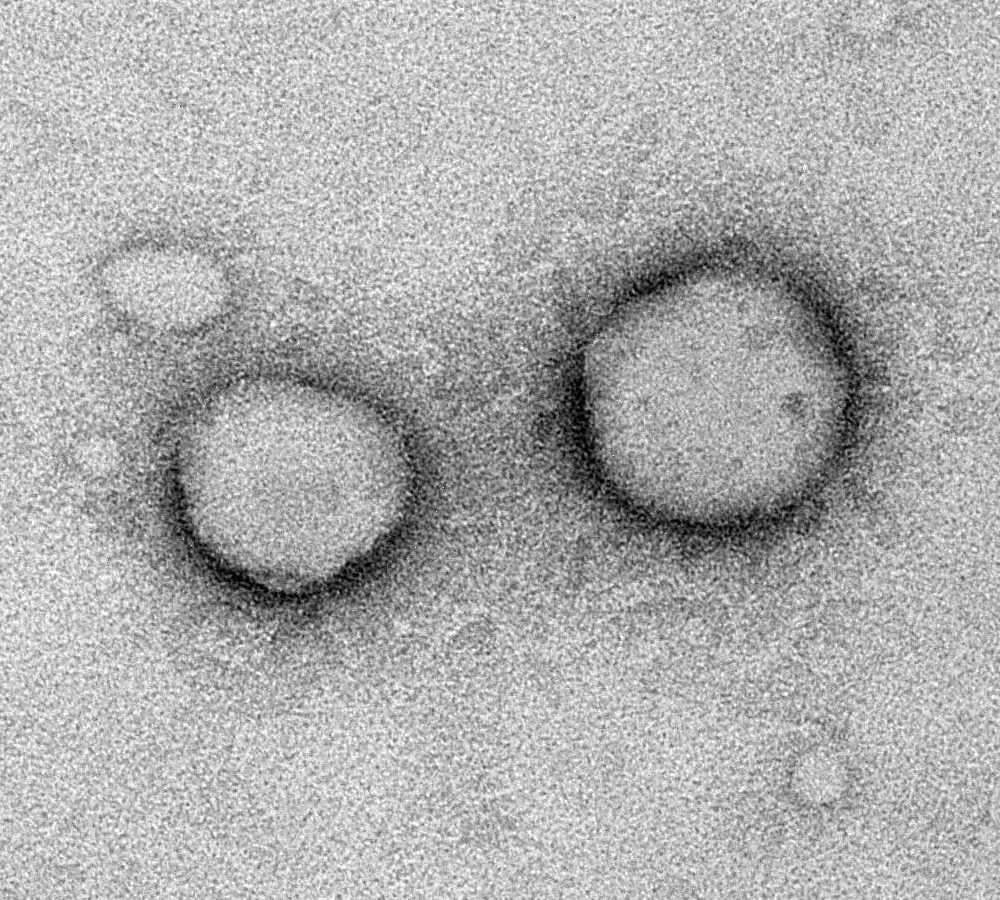

Supplement: Supplementary file 1 [file DataSheet3.zip › original images of figure 4/图4B-1.png]

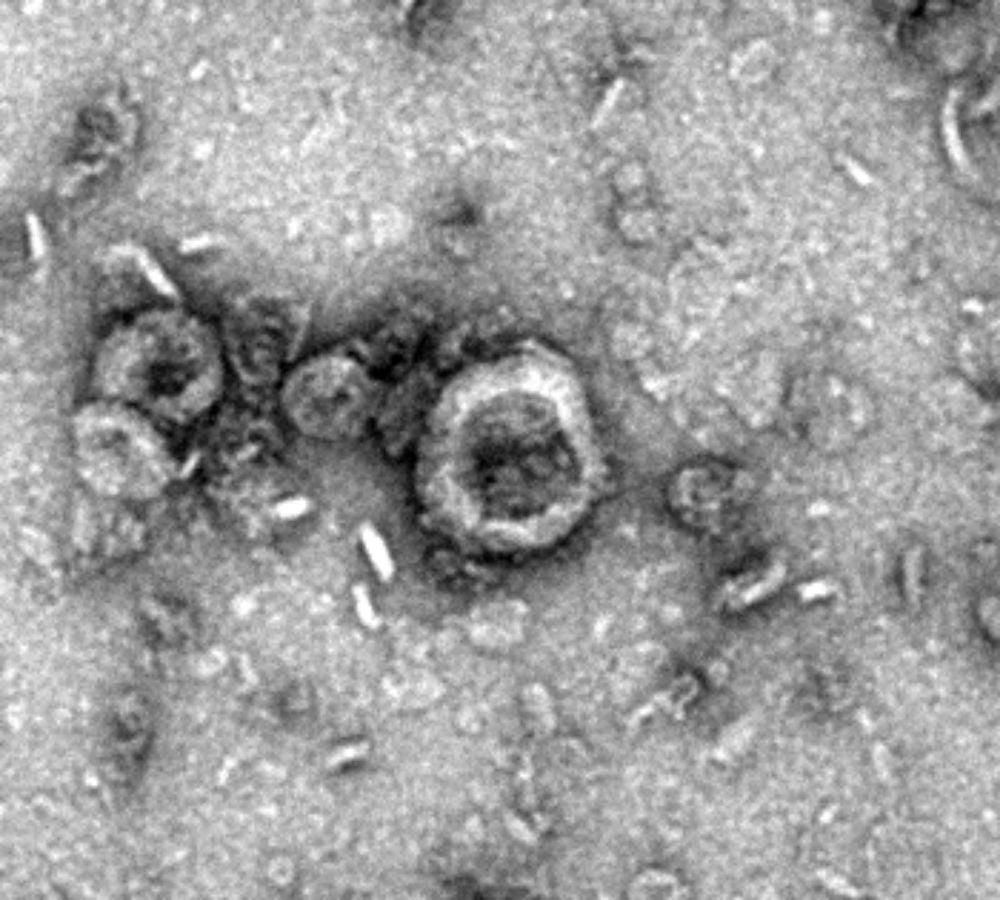

Supplement: Supplementary file 1 [file DataSheet3.zip › original images of figure 4/图4B-2.png]

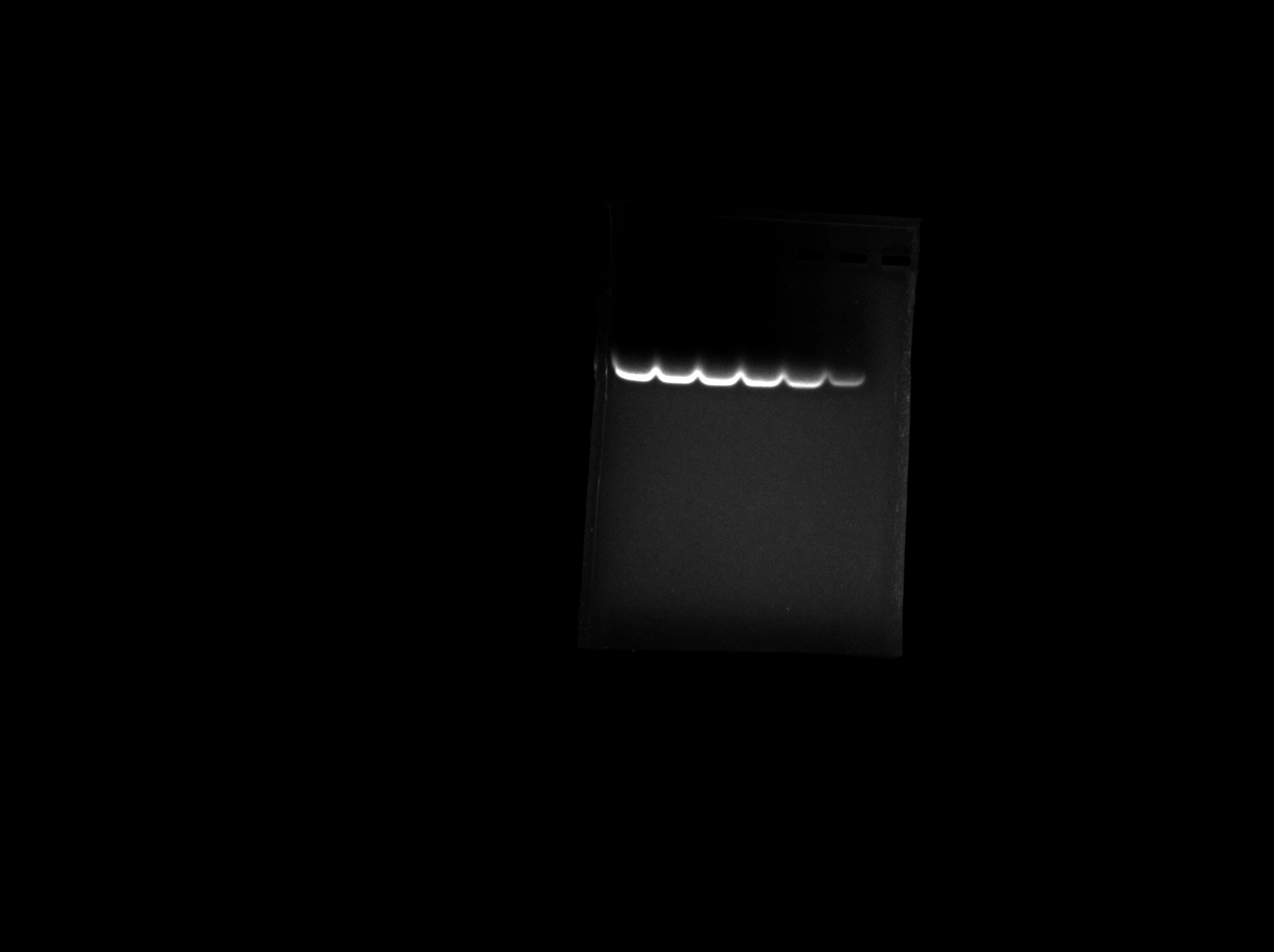

Supplement: Supplementary file 1 [file DataSheet3.zip › original images of figure 4/图4G-1 (1).jpg]

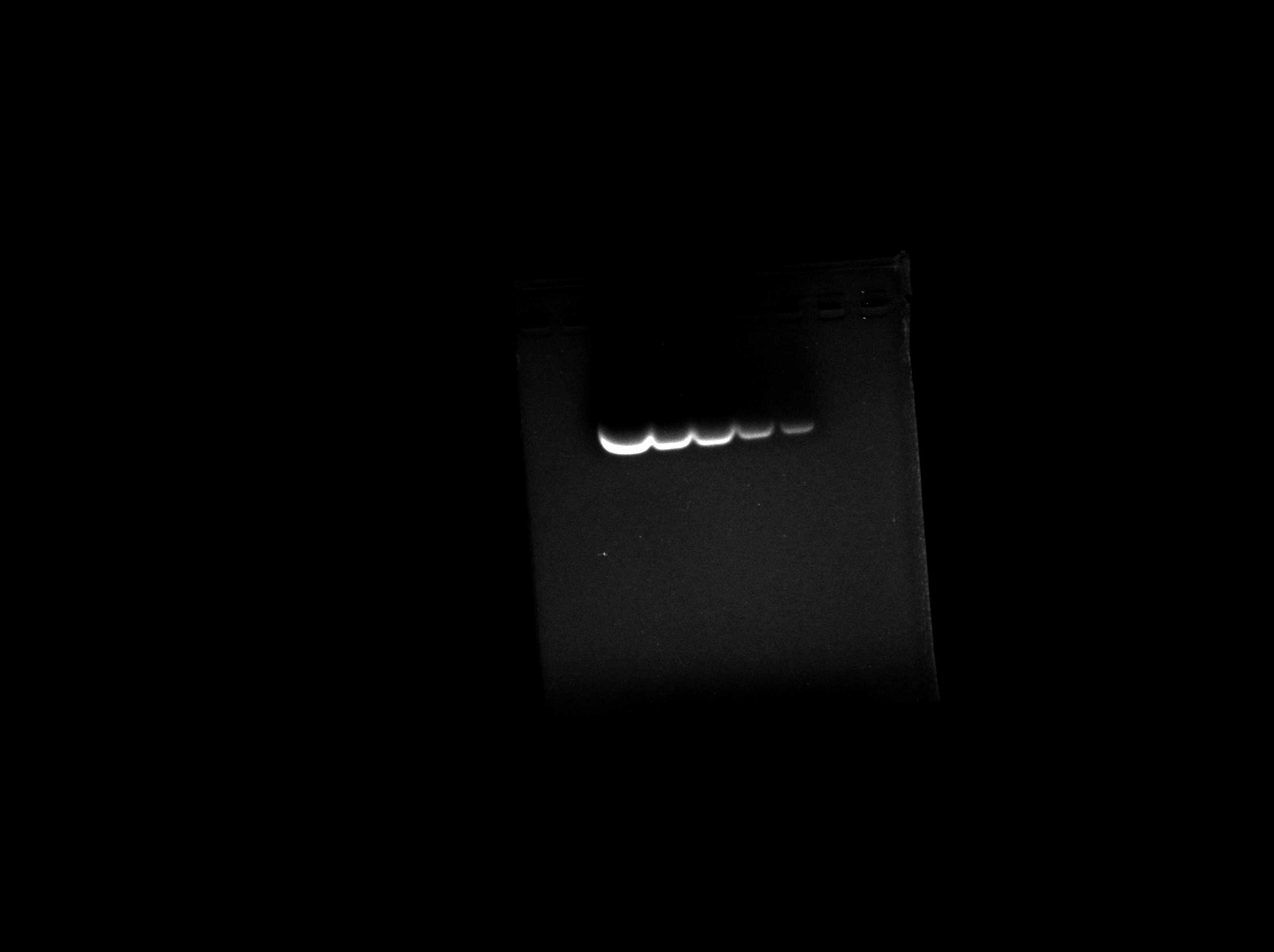

Supplement: Supplementary file 1 [file DataSheet3.zip › original images of figure 4/图4G-1 (2).jpg]

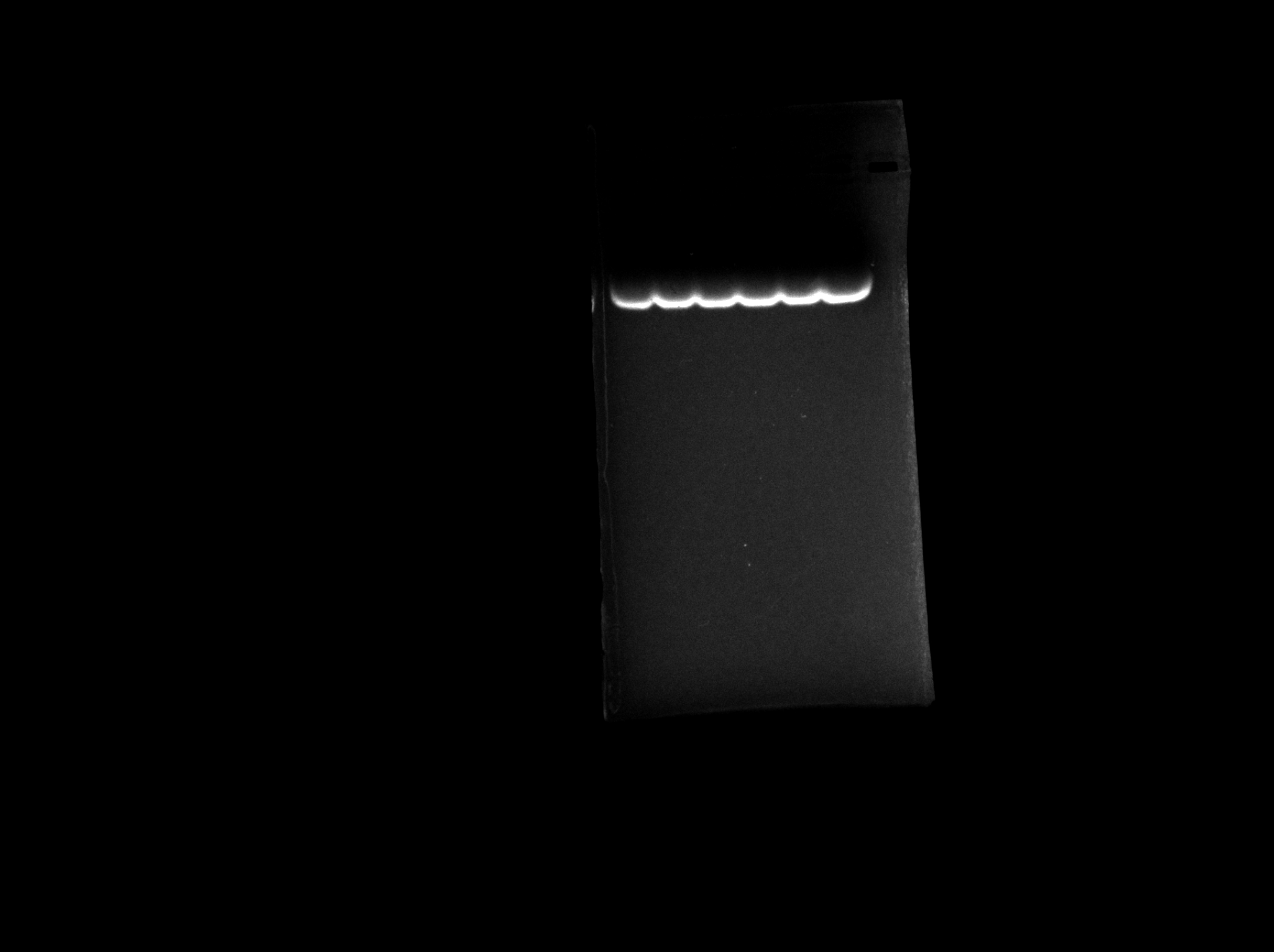

Supplement: Supplementary file 1 [file DataSheet3.zip › original images of figure 4/图4G-2 (1).jpg]

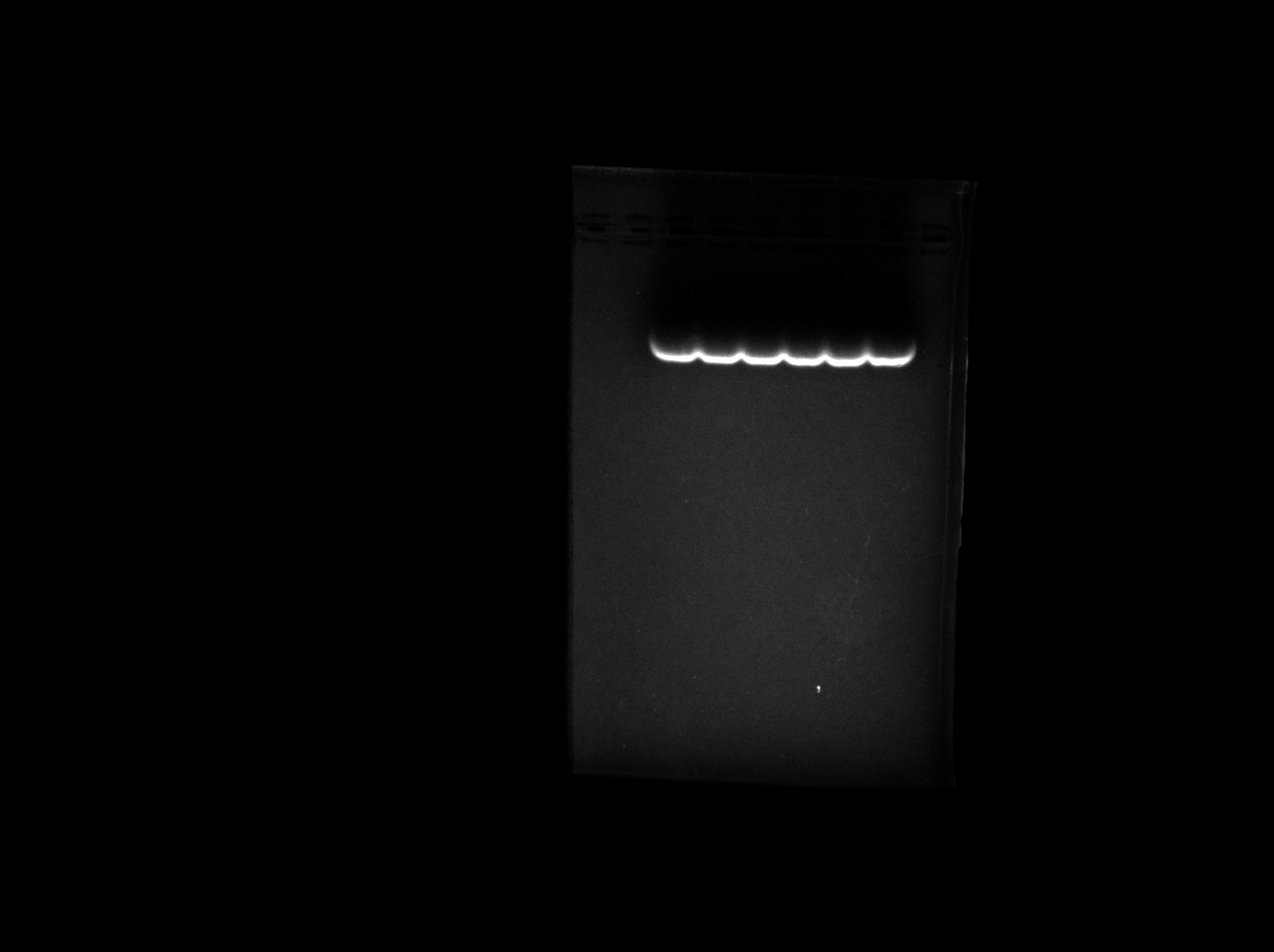

Supplement: Supplementary file 1 [file DataSheet3.zip › original images of figure 4/图4G-2 (2).jpg]

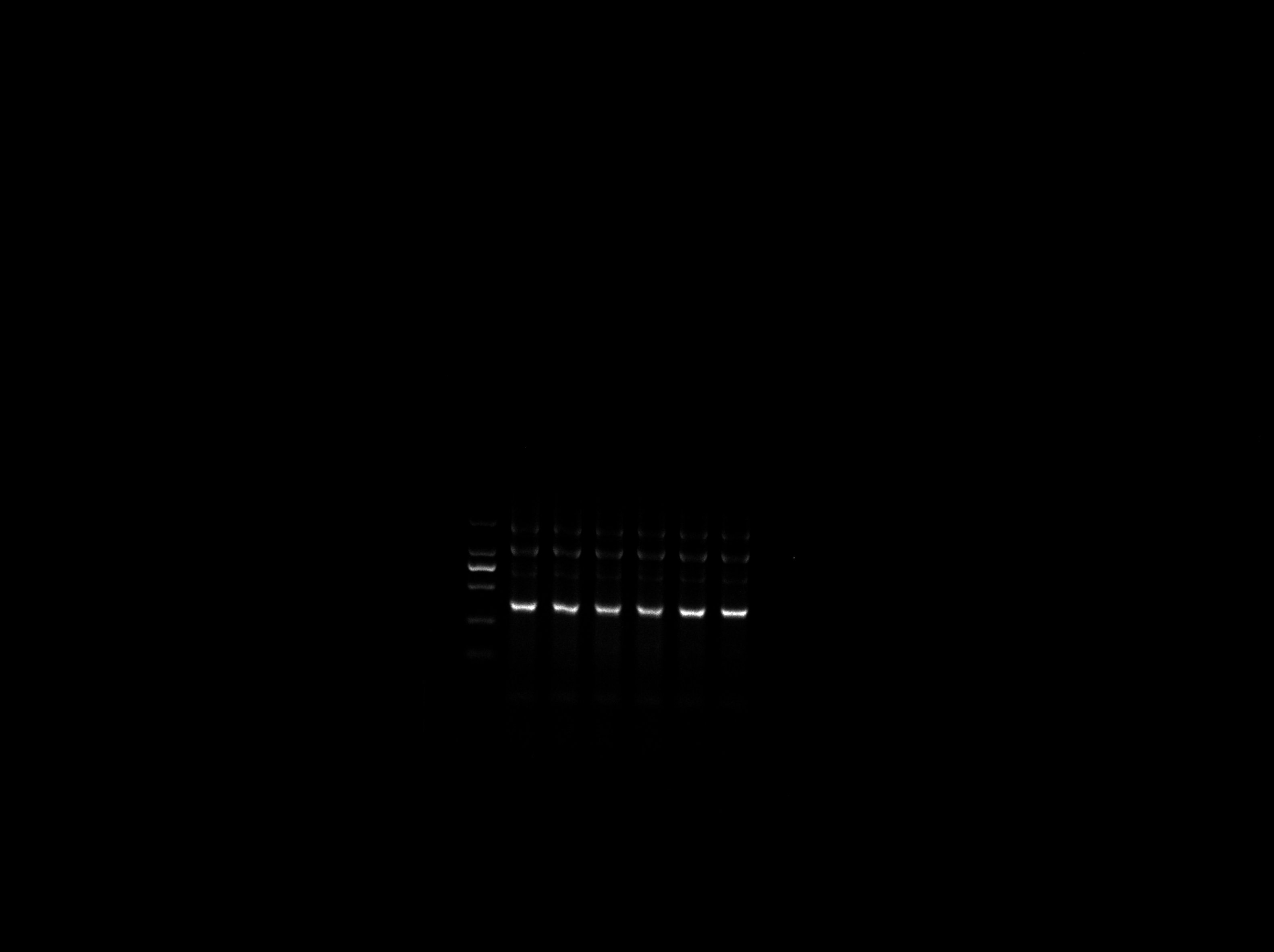

Supplement: Supplementary file 1 [file DataSheet3.zip › original images of figure 4/图4G-3 (1).jpg]

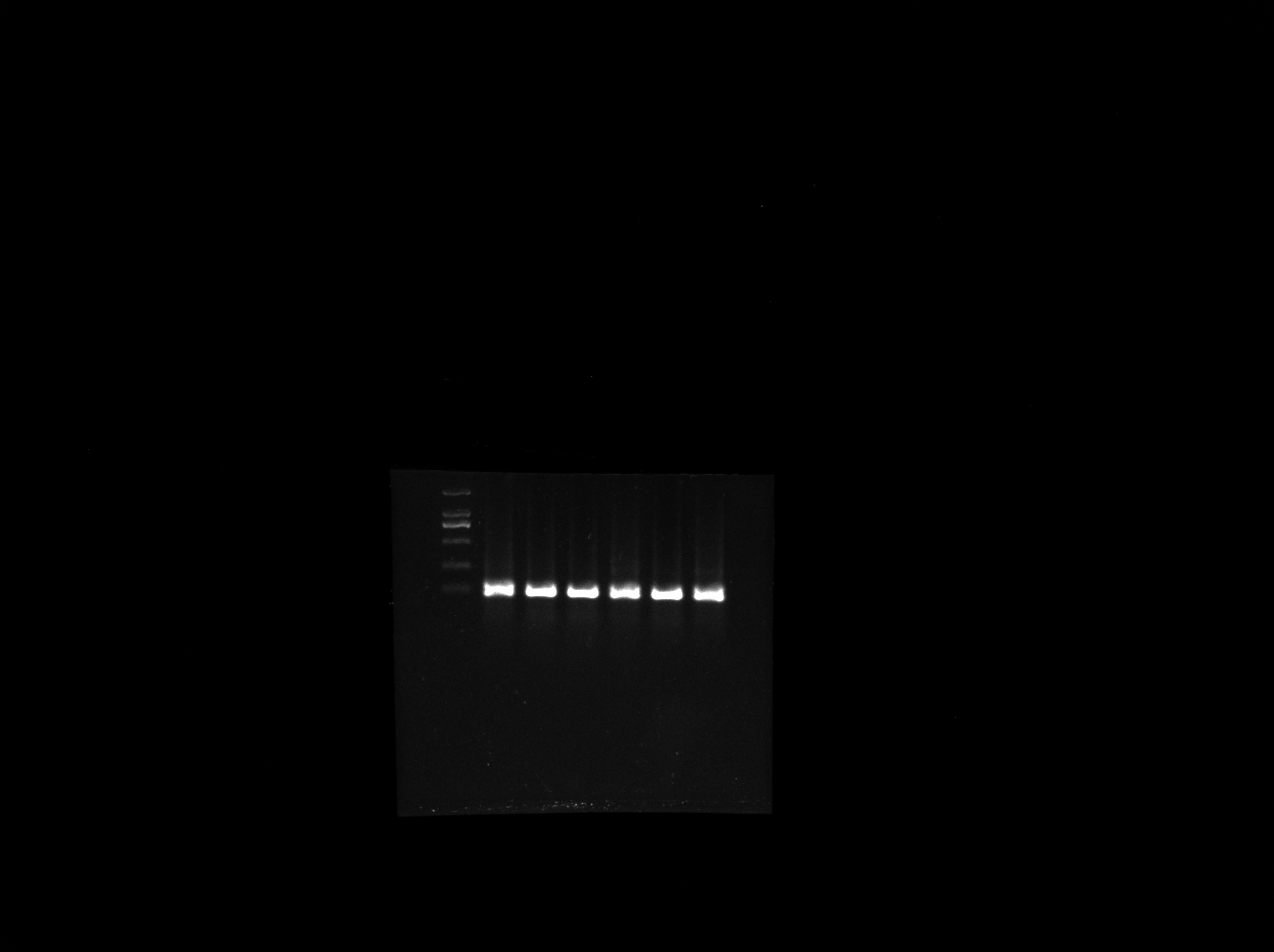

Supplement: Supplementary file 1 [file DataSheet3.zip › original images of figure 4/图4G-3 (2).jpg]

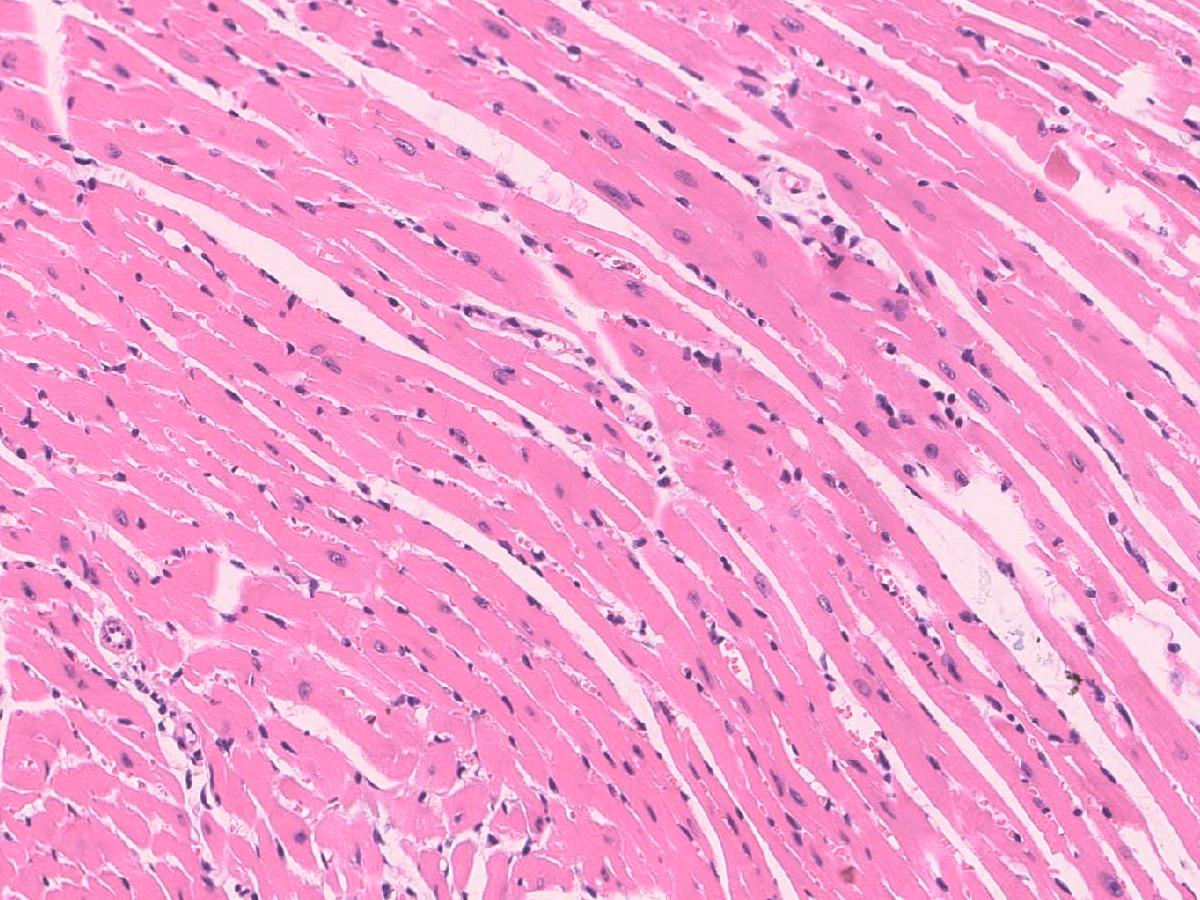

Supplement: Supplementary file 3 [file DataSheet4.zip › original images of figure 5/图5E-1-1(Heart).jpg]

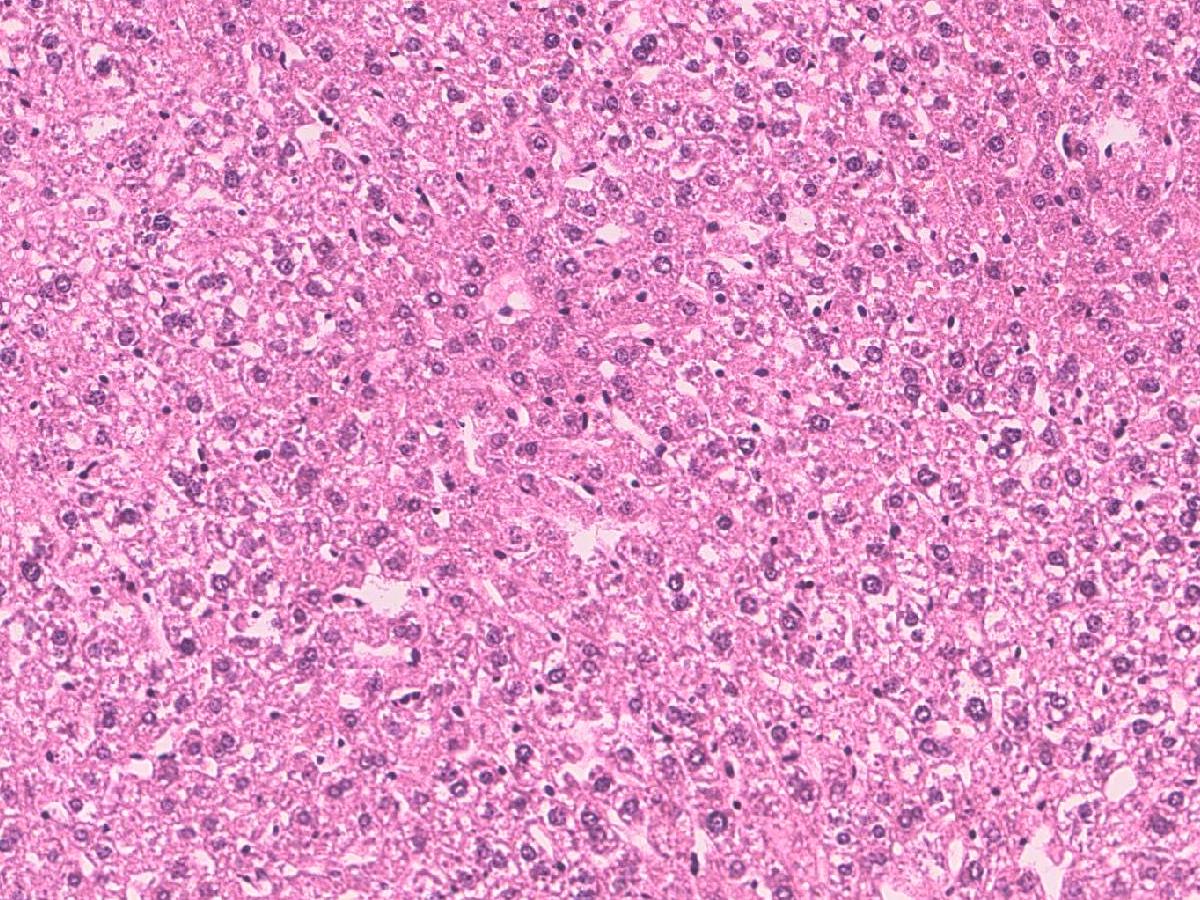

Supplement: Supplementary file 3 [file DataSheet4.zip › original images of figure 5/图5E-1-2(Liver).jpg]

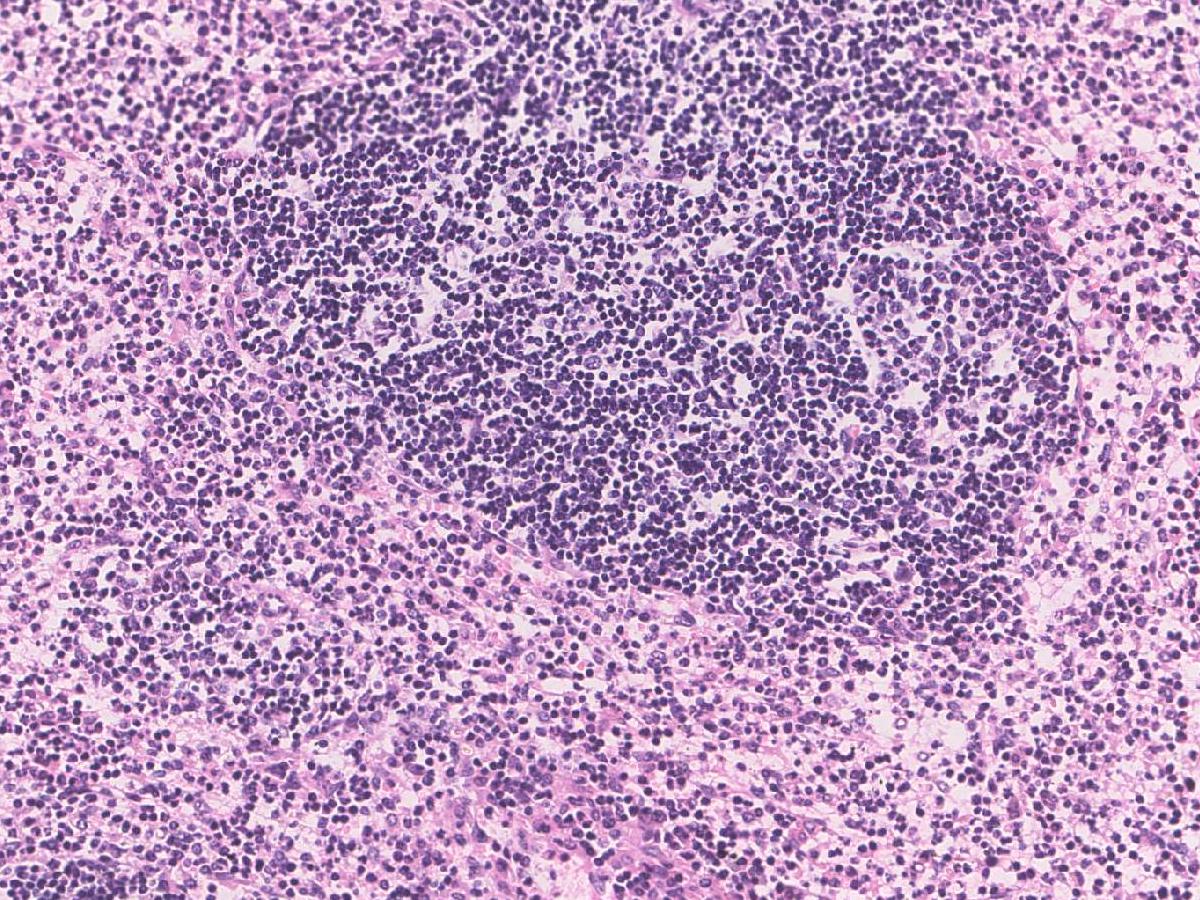

Supplement: Supplementary file 3 [file DataSheet4.zip › original images of figure 5/图5E-1-3(Spleen).jpg]

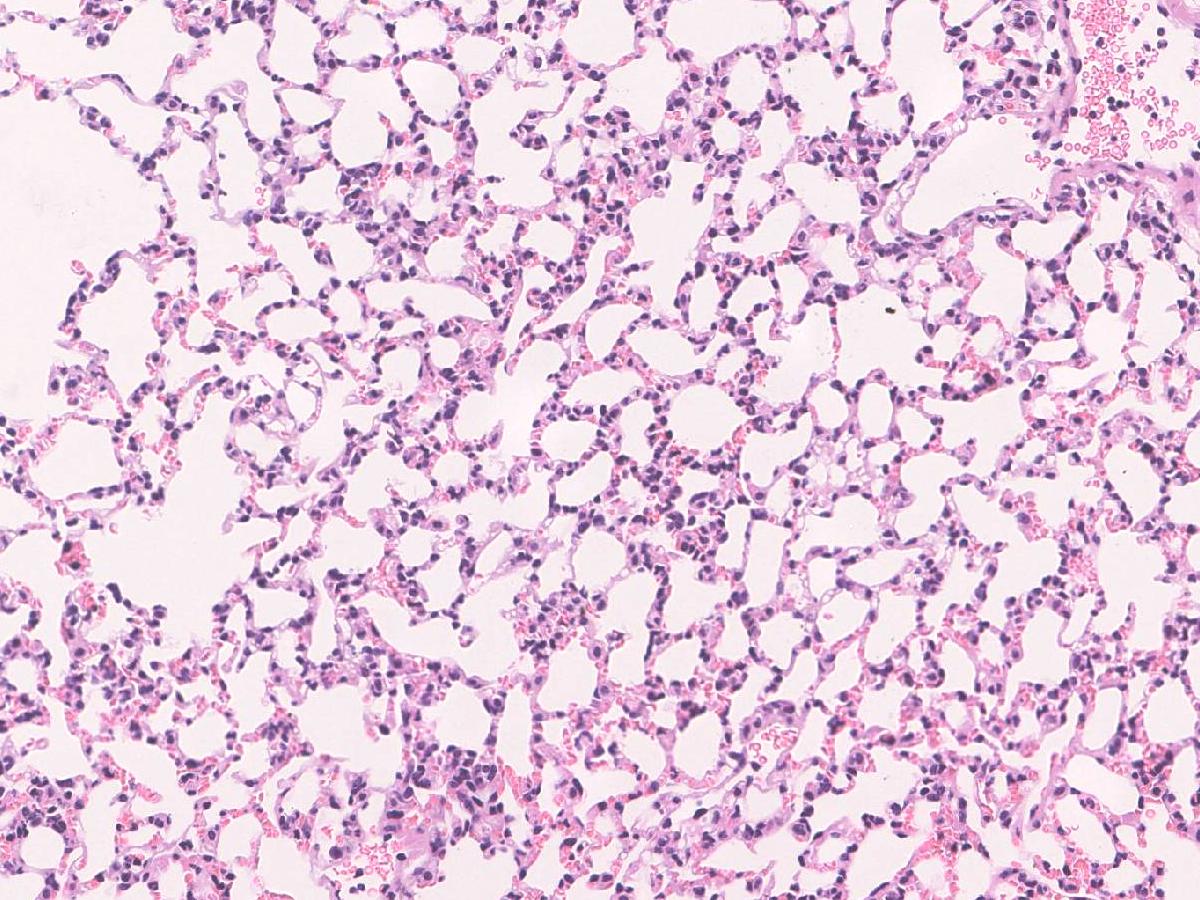

Supplement: Supplementary file 3 [file DataSheet4.zip › original images of figure 5/图5E-1-4(Lung).jpg]

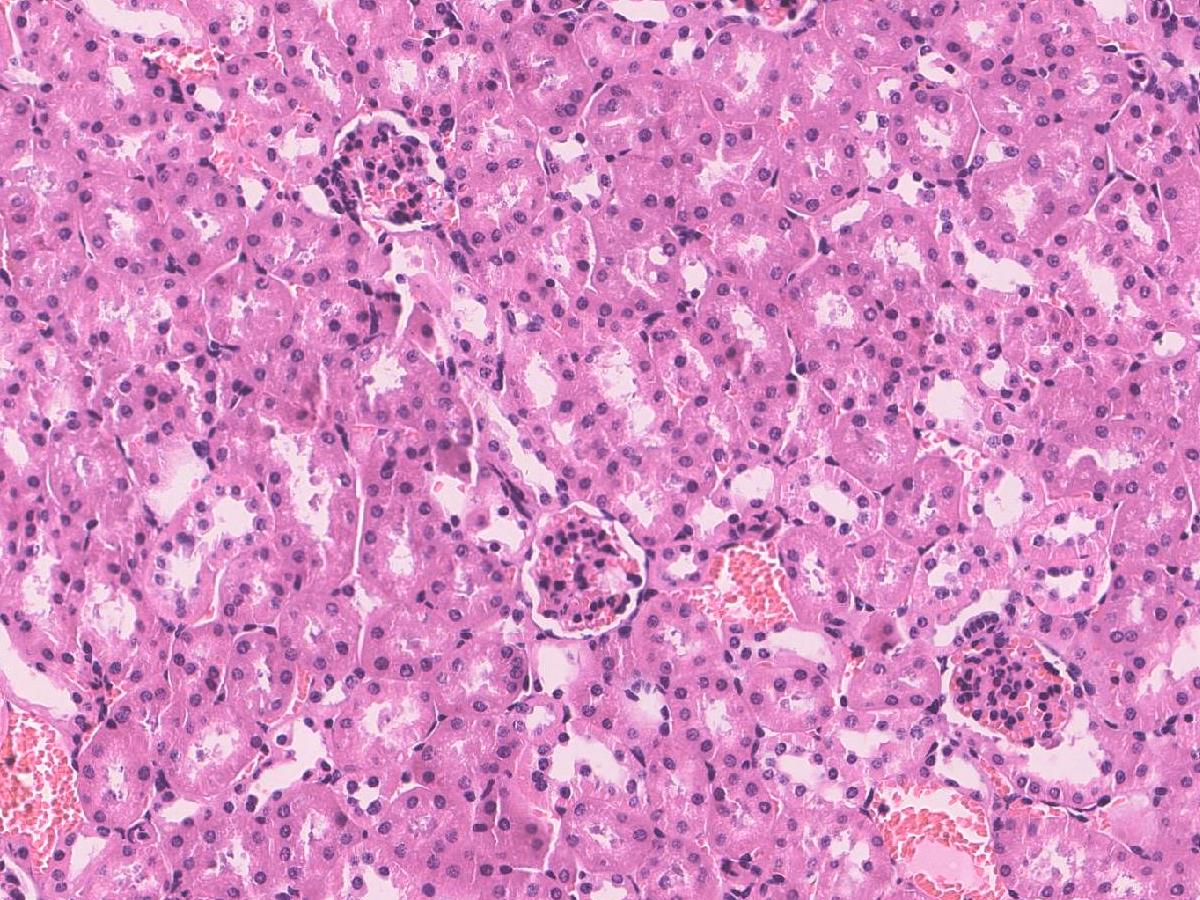

Supplement: Supplementary file 3 [file DataSheet4.zip › original images of figure 5/图5E-1-5(Kidney).jpg]

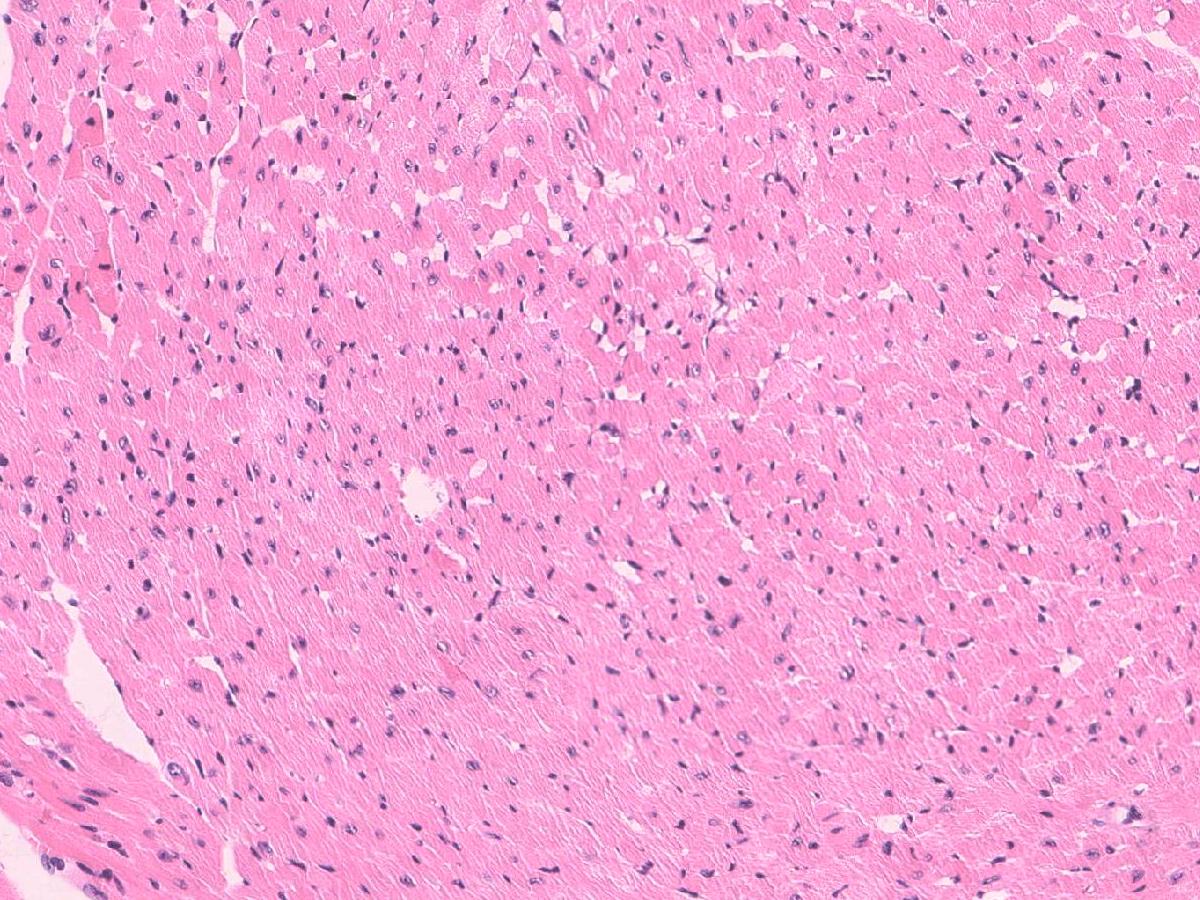

Supplement: Supplementary file 3 [file DataSheet4.zip › original images of figure 5/图5E-2-1(Heart).jpg]

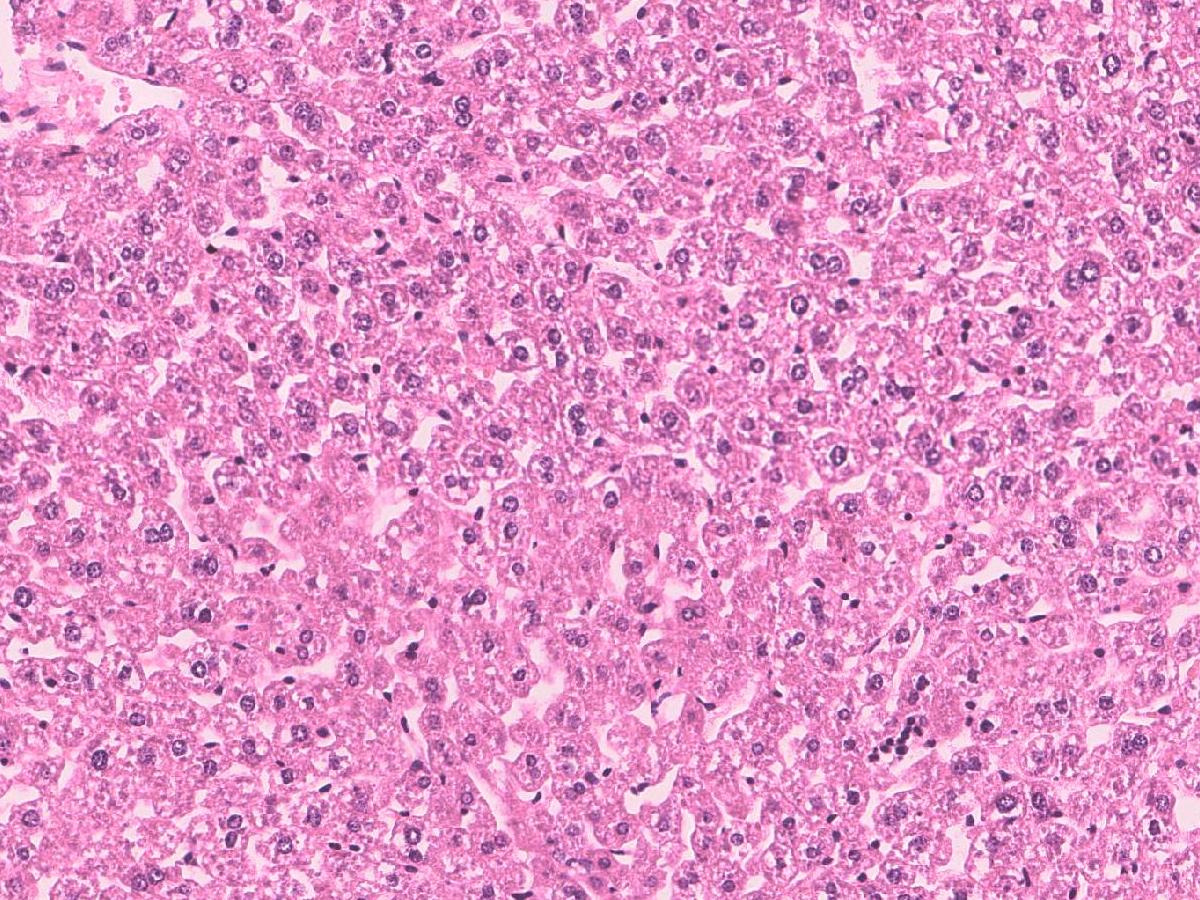

Supplement: Supplementary file 3 [file DataSheet4.zip › original images of figure 5/图5E-2-2(Liver).jpg]

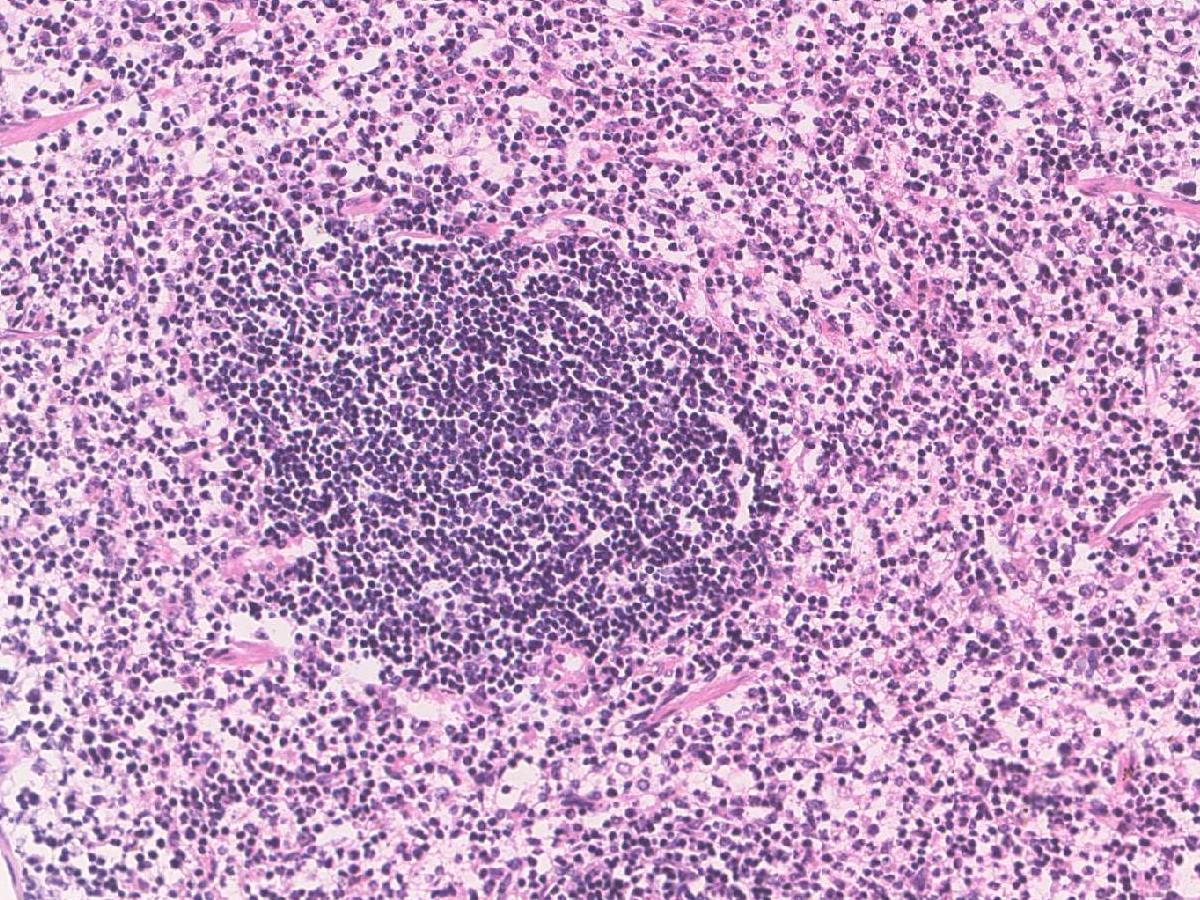

Supplement: Supplementary file 3 [file DataSheet4.zip › original images of figure 5/图5E-2-3(Spleen).jpg]

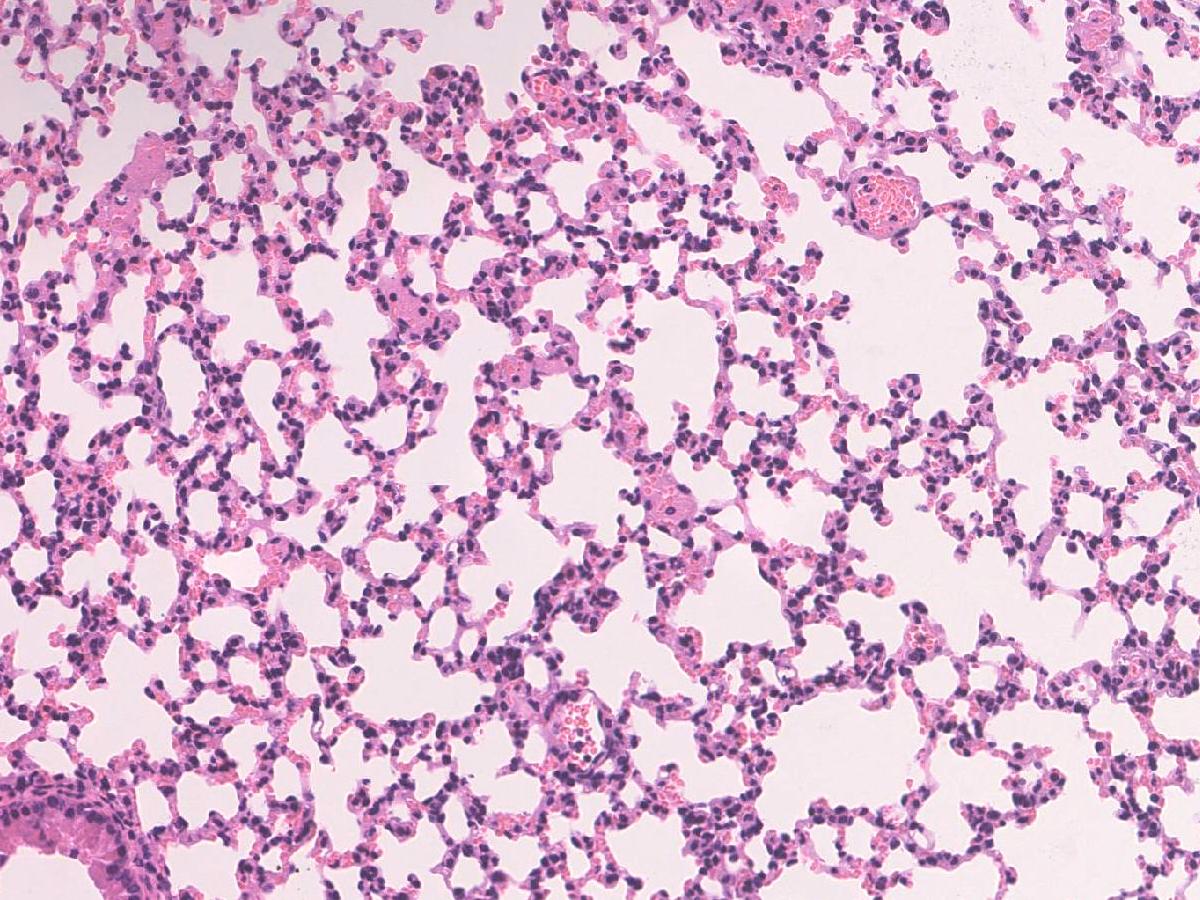

Supplement: Supplementary file 3 [file DataSheet4.zip › original images of figure 5/图5E-2-4(Lung).jpg]

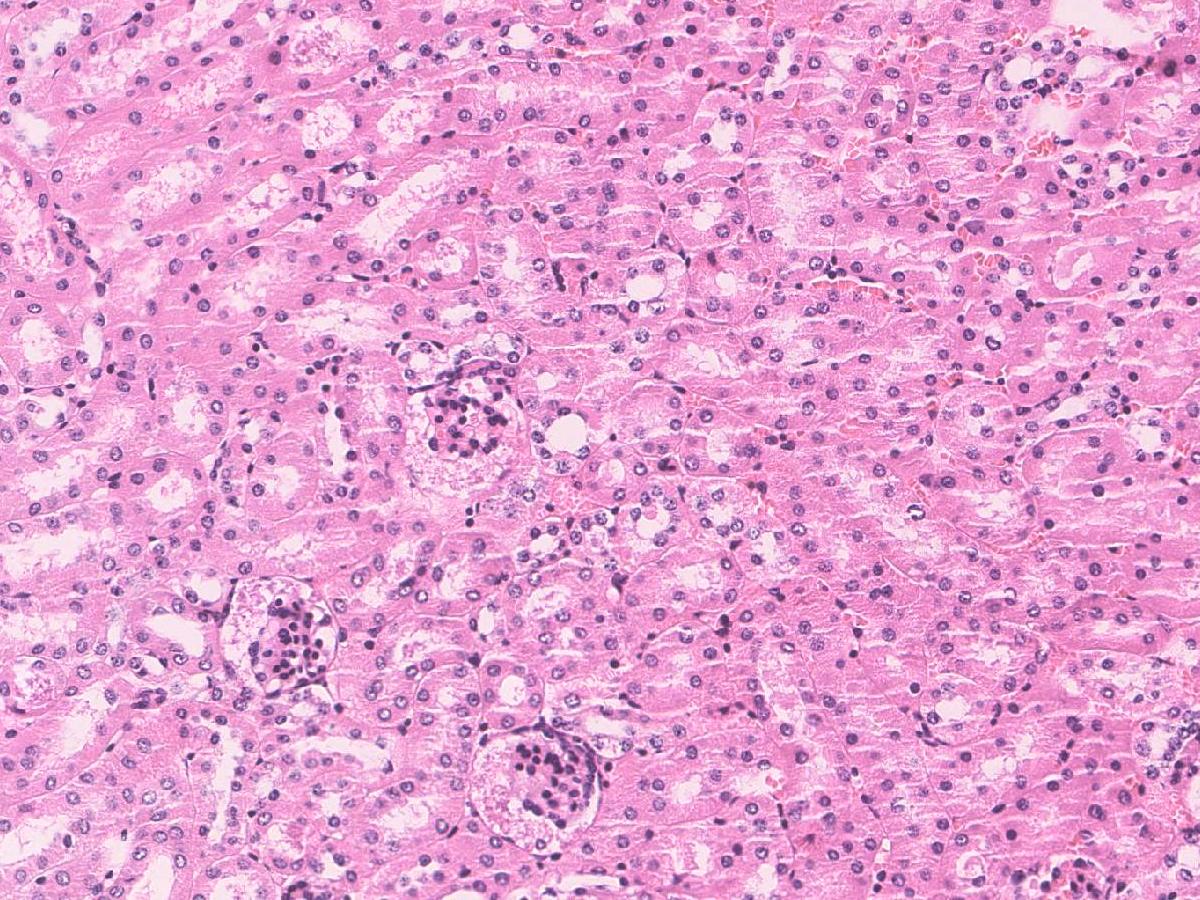

Supplement: Supplementary file 3 [file DataSheet4.zip › original images of figure 5/图5E-2-5(Kidney).jpg]

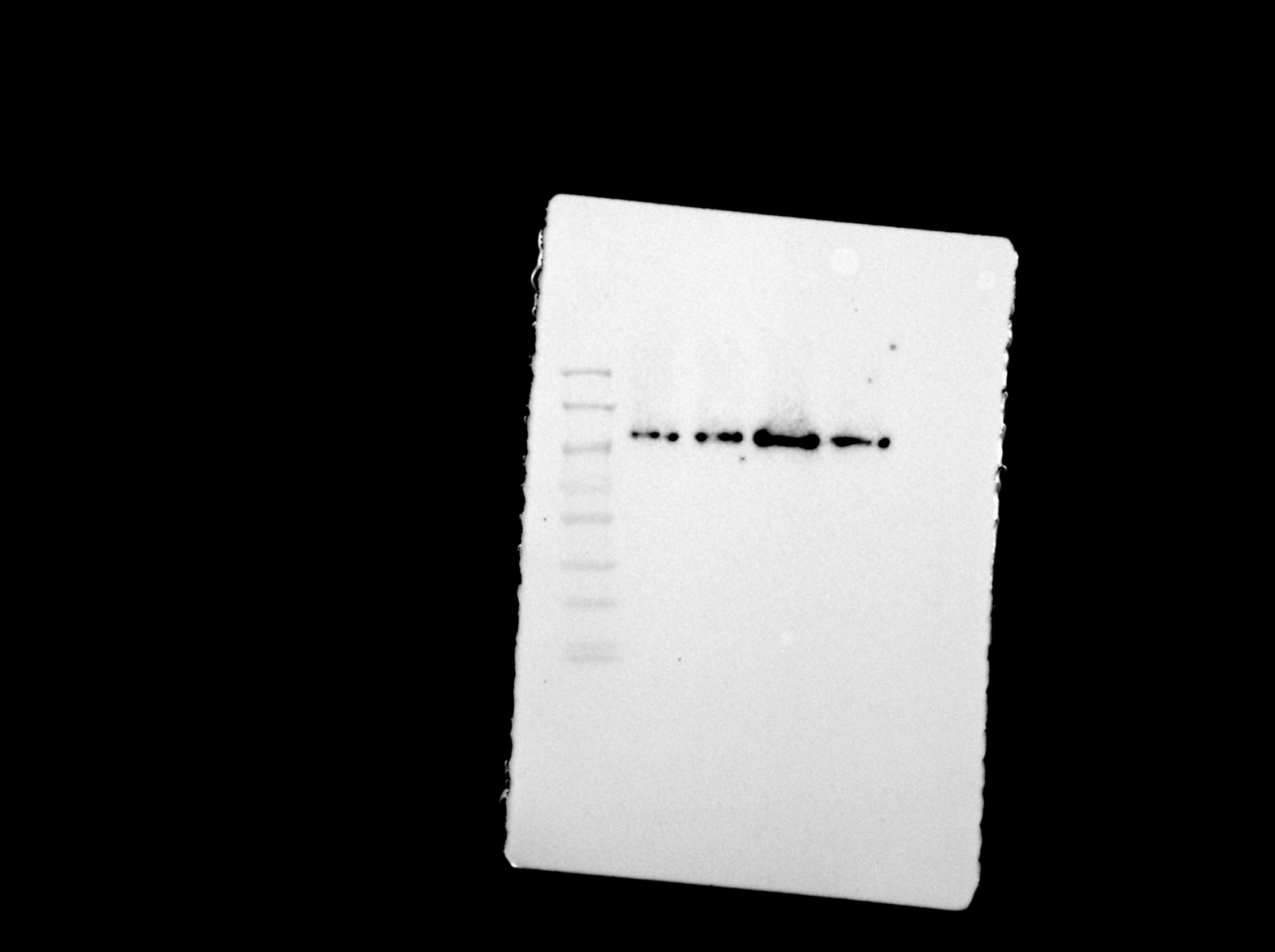

Supplement: Supplementary file 4 [file DataSheet1.zip › original images of figure 2/图2B-1.jpg]

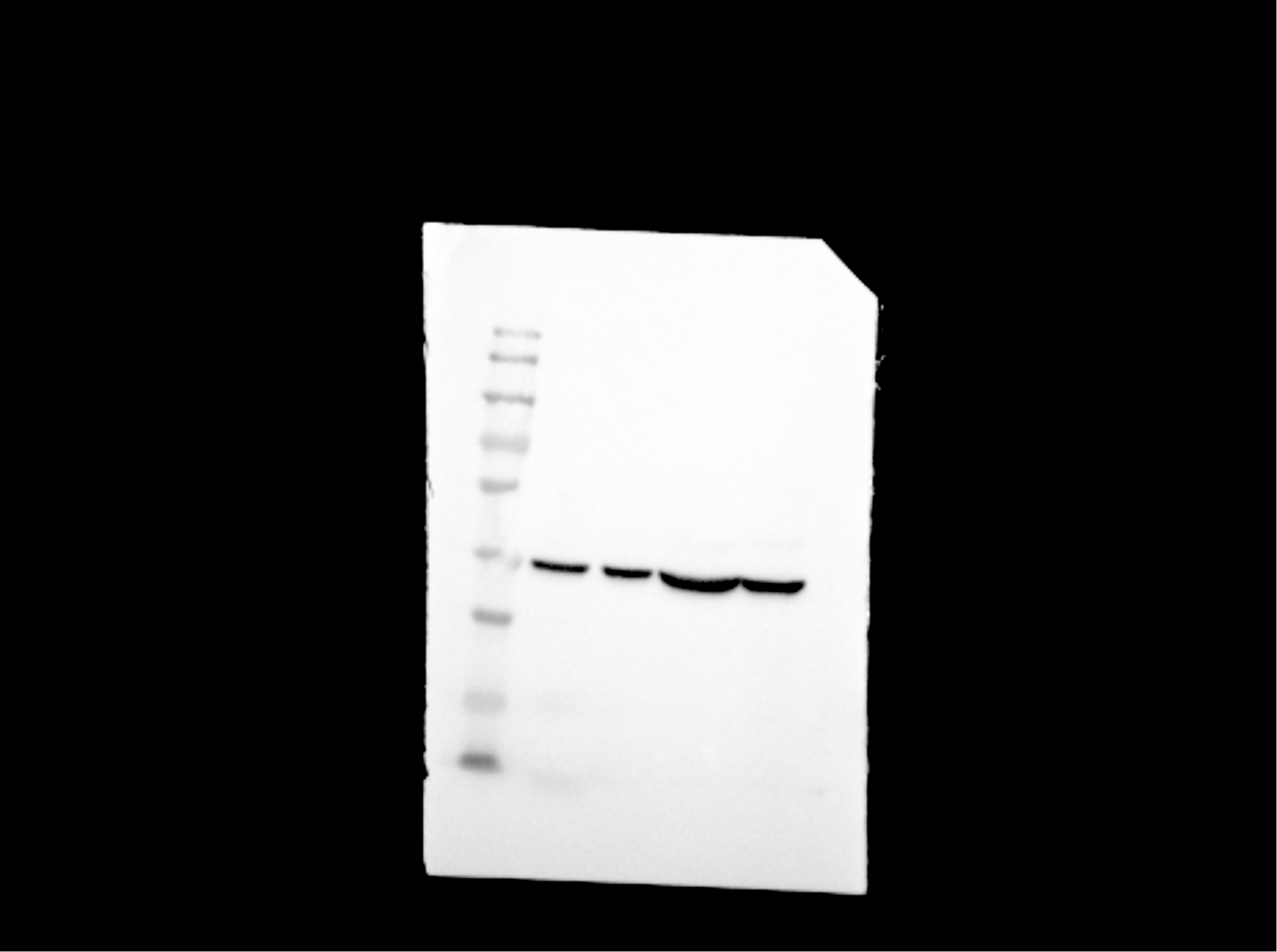

Supplement: Supplementary file 4 [file DataSheet1.zip › original images of figure 2/图2B-2.jpg]

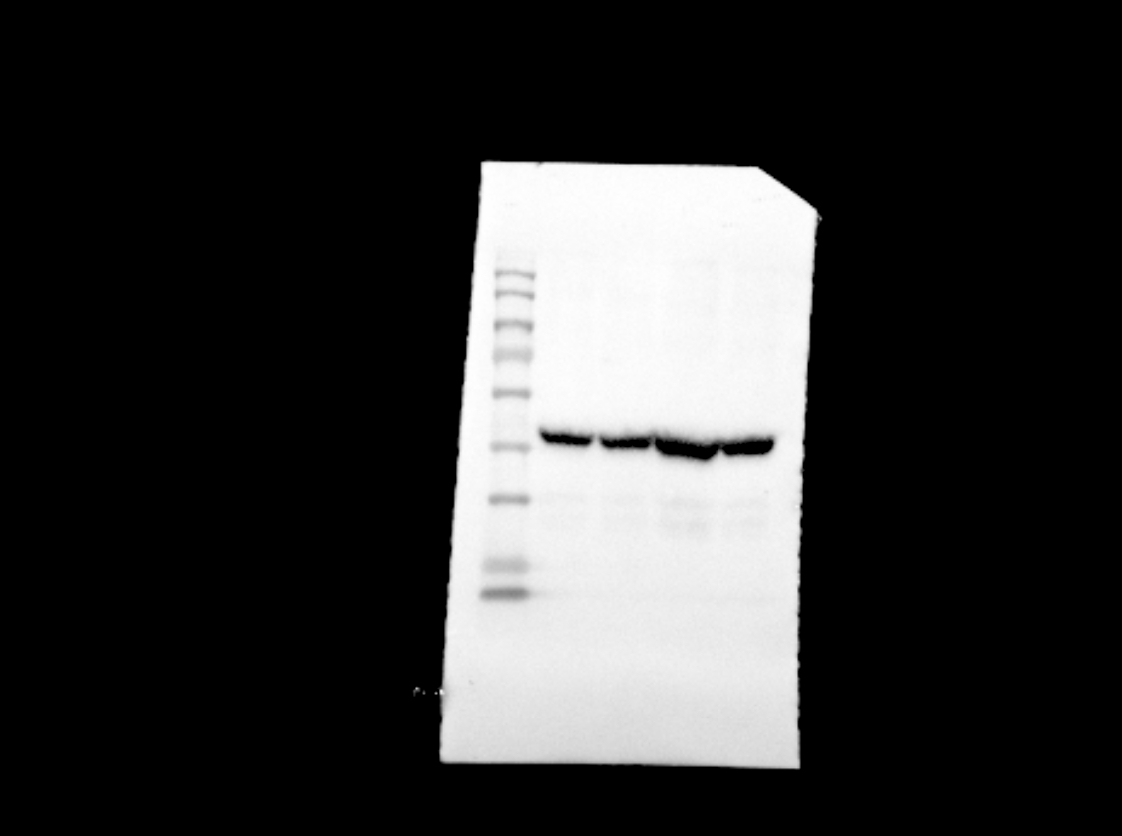

Supplement: Supplementary file 4 [file DataSheet1.zip › original images of figure 2/图2B-3.jpg]

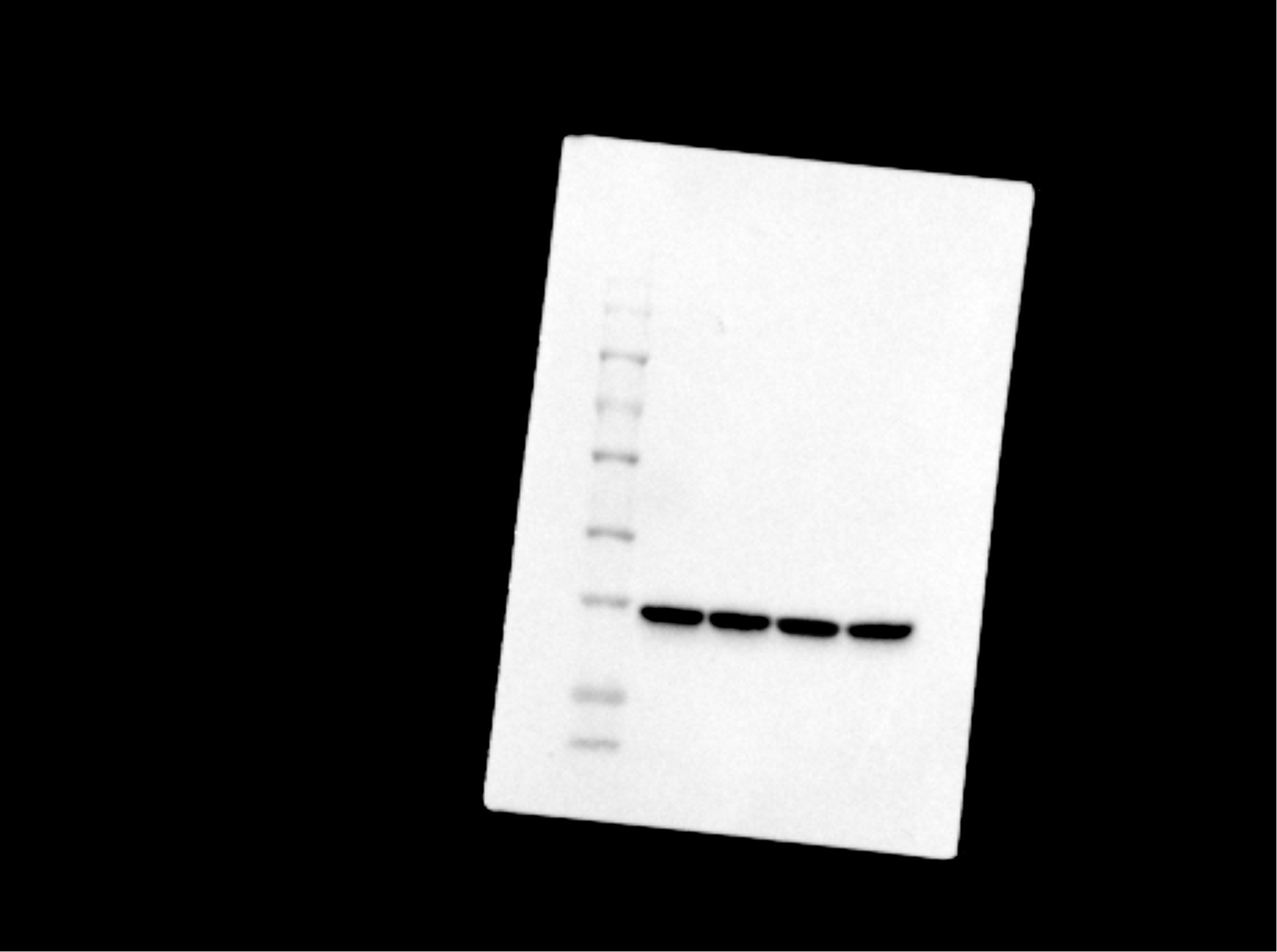

Supplement: Supplementary file 4 [file DataSheet1.zip › original images of figure 2/图2B-4.jpg]

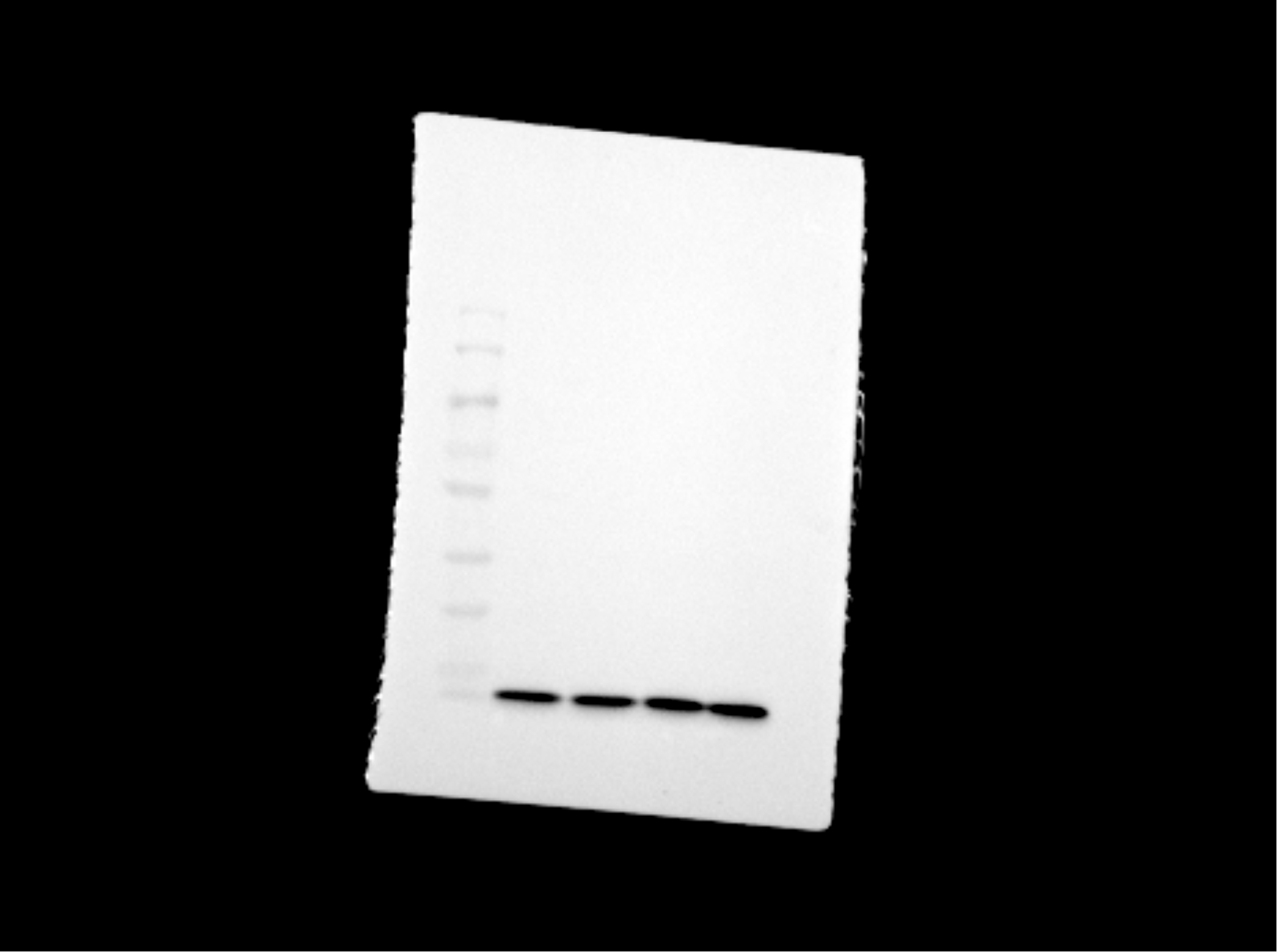

Supplement: Supplementary file 4 [file DataSheet1.zip › original images of figure 2/图2C-1.jpg]

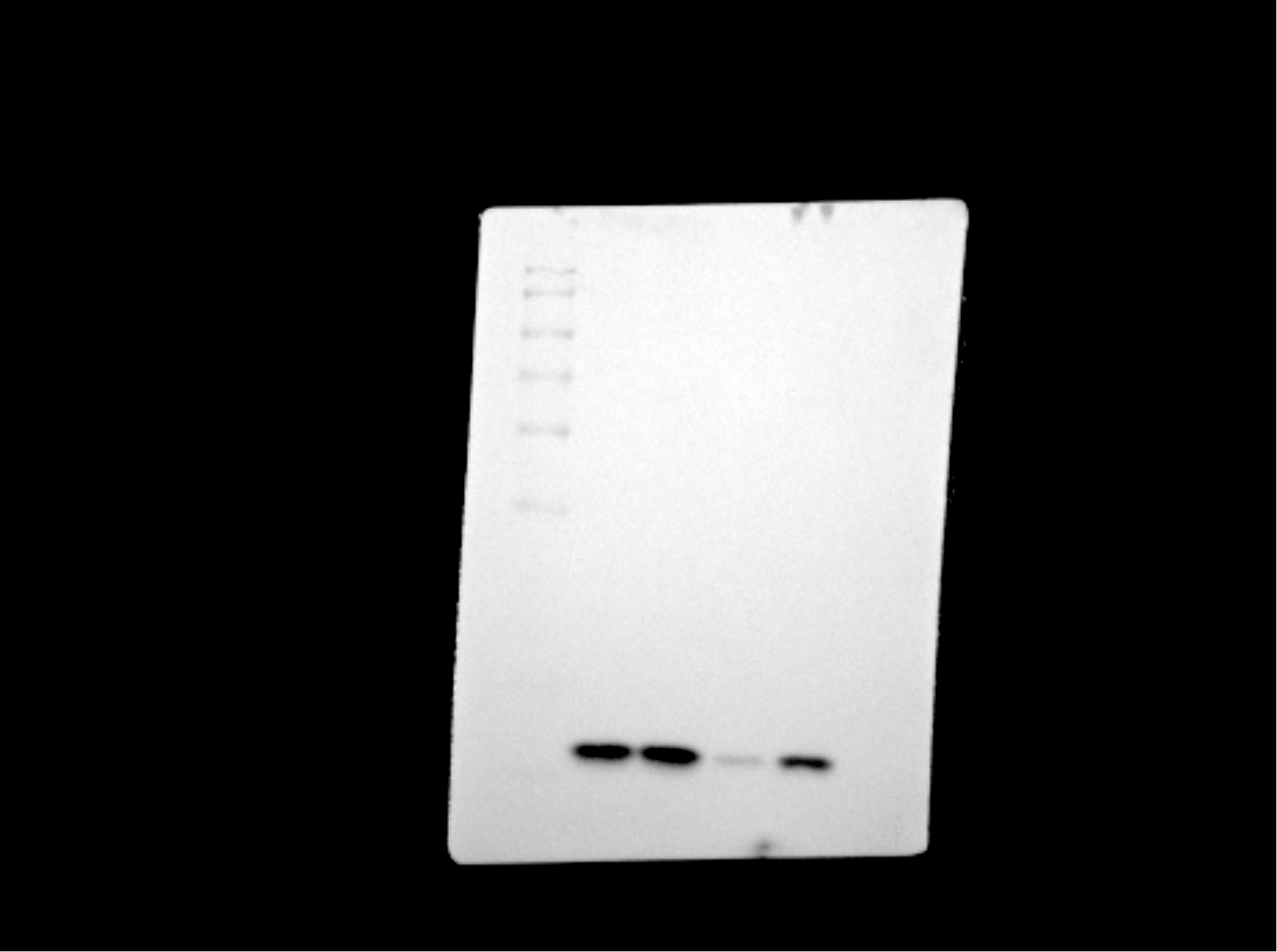

Supplement: Supplementary file 4 [file DataSheet1.zip › original images of figure 2/图2C-2.jpg]

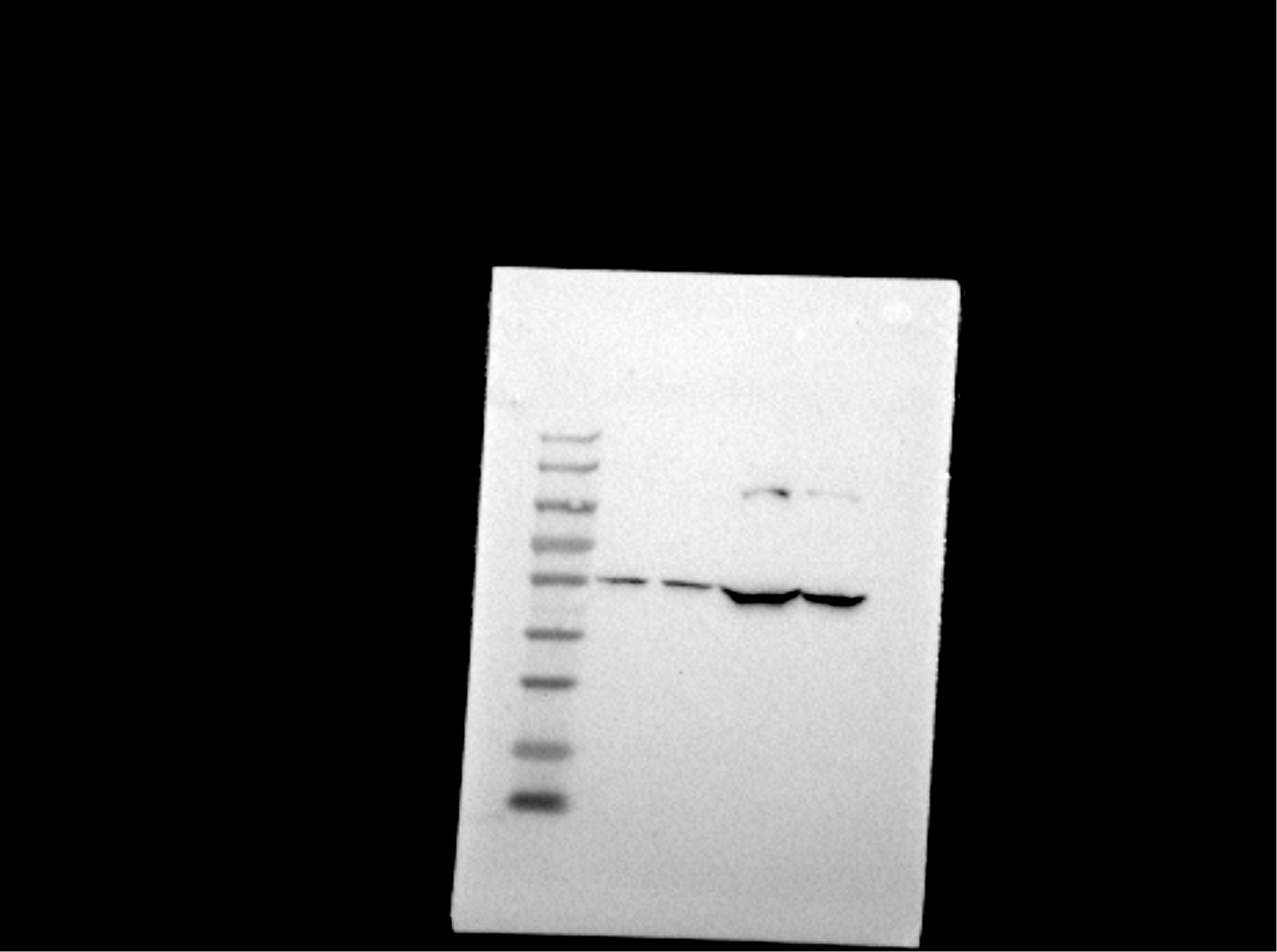

Supplement: Supplementary file 4 [file DataSheet1.zip › original images of figure 2/图2C-3.jpg]

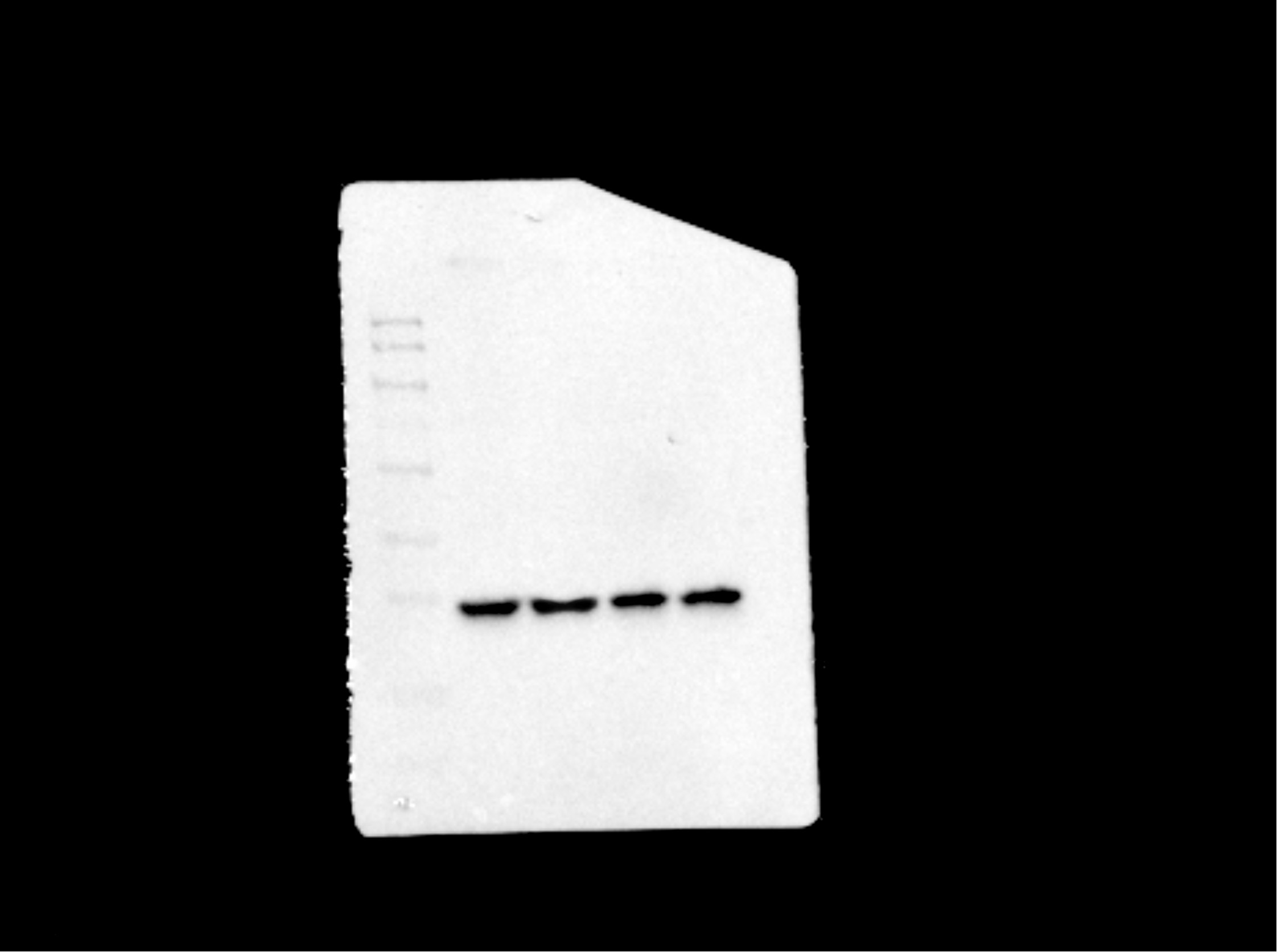

Supplement: Supplementary file 4 [file DataSheet1.zip › original images of figure 2/图2C-4.jpg]

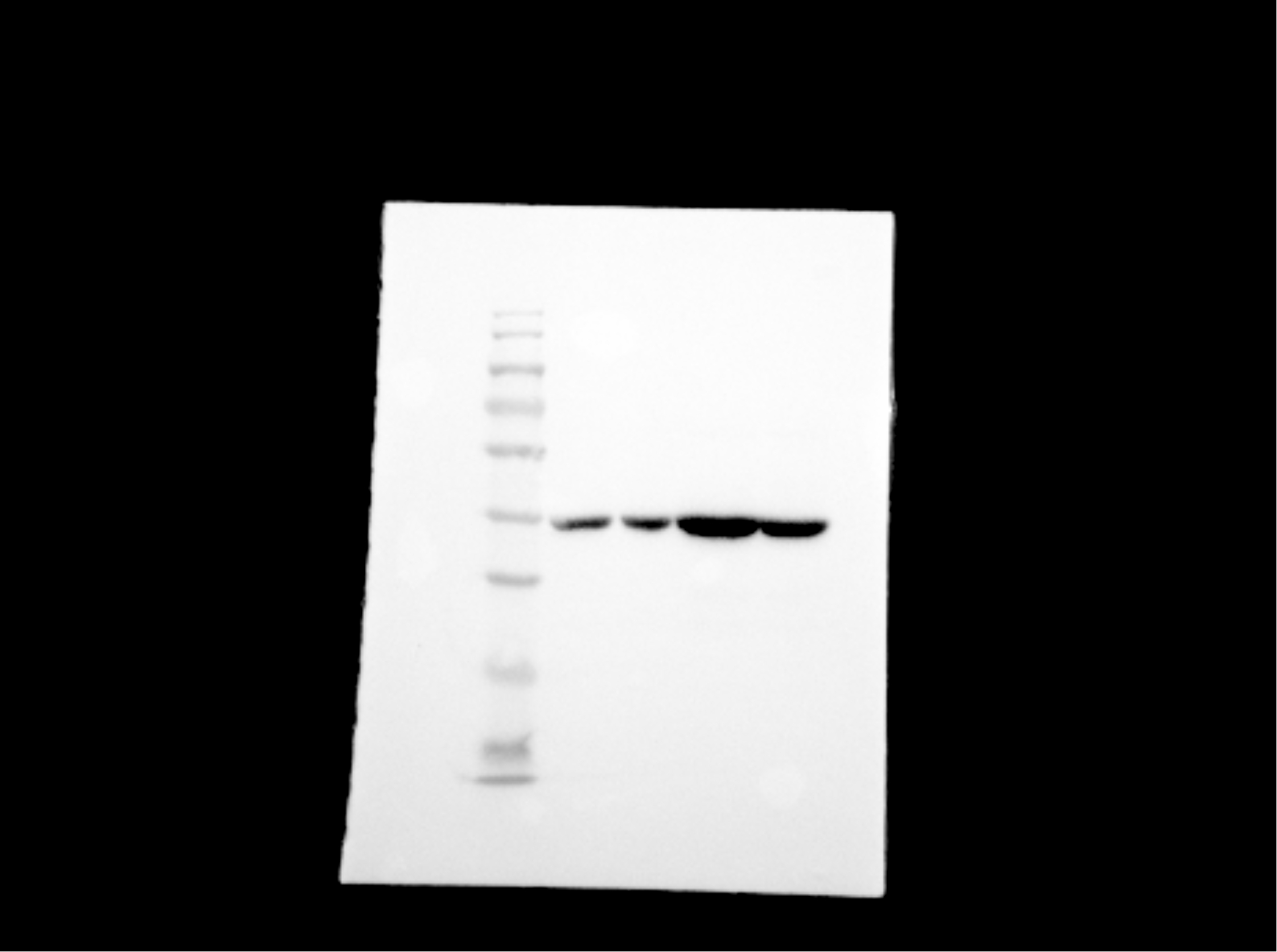

Supplement: Supplementary file 4 [file DataSheet1.zip › original images of figure 2/图2G-1.jpg]

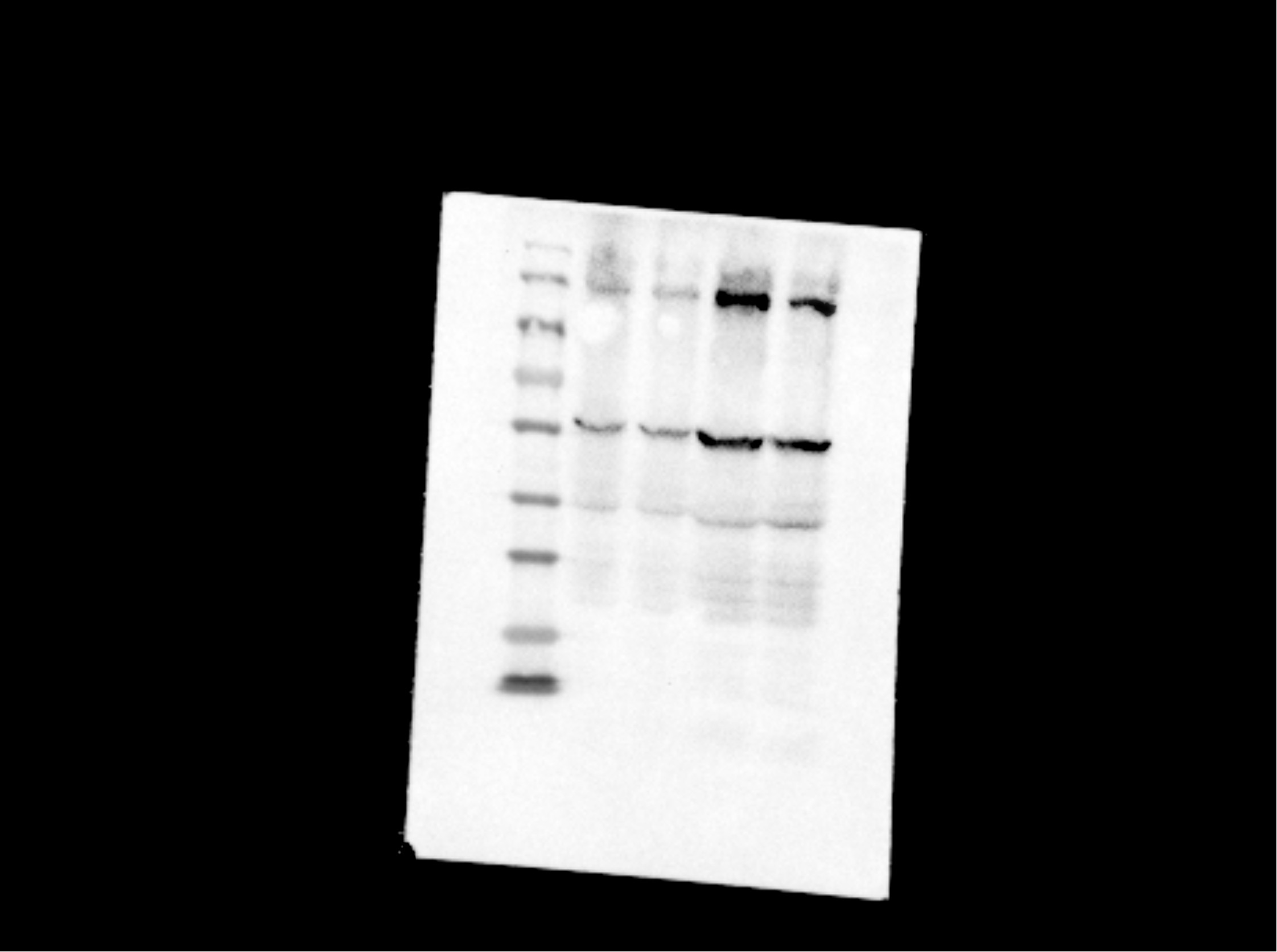

Supplement: Supplementary file 4 [file DataSheet1.zip › original images of figure 2/图2G-2.jpg]

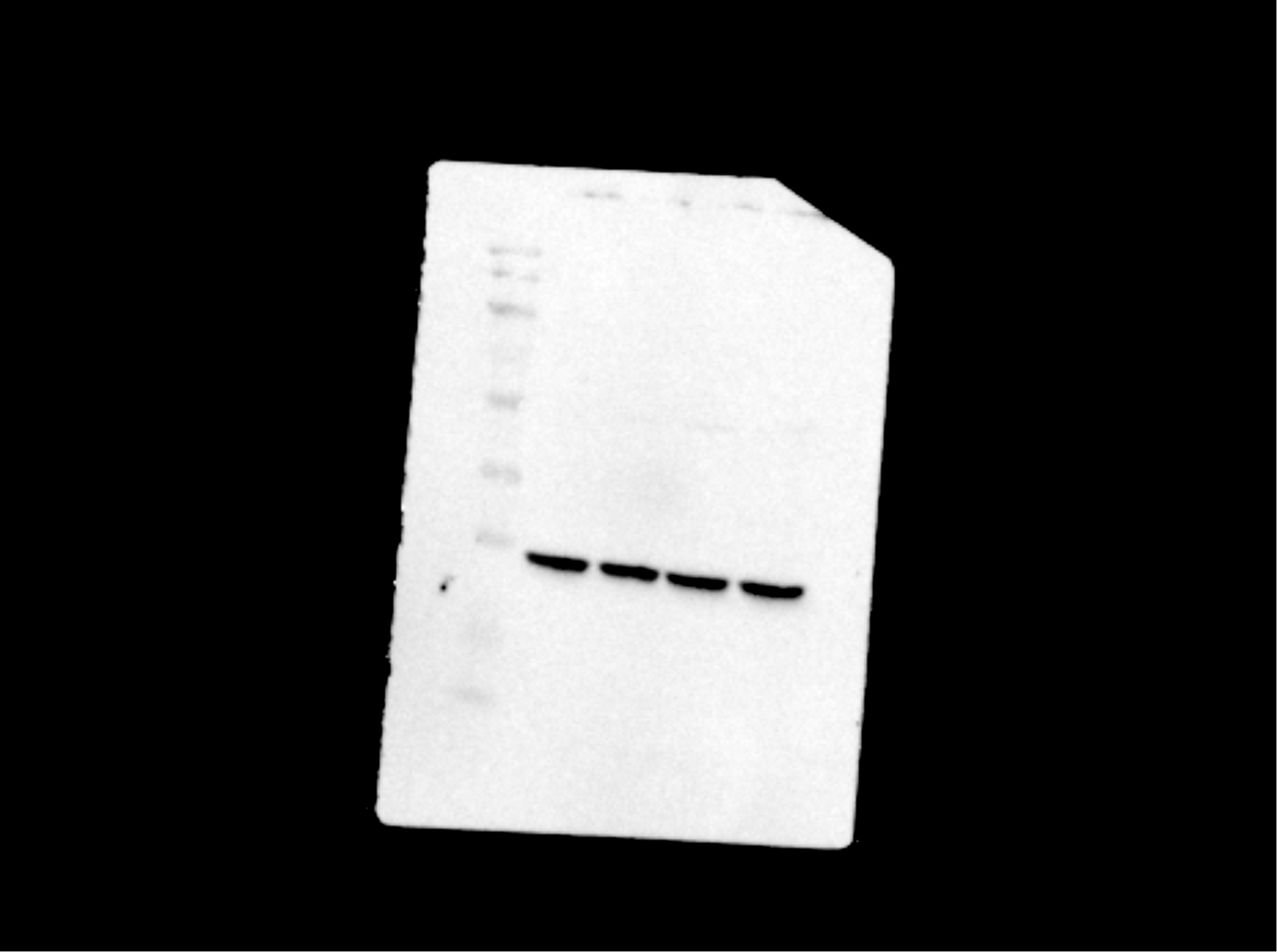

Supplement: Supplementary file 4 [file DataSheet1.zip › original images of figure 2/图2G-3.jpg]

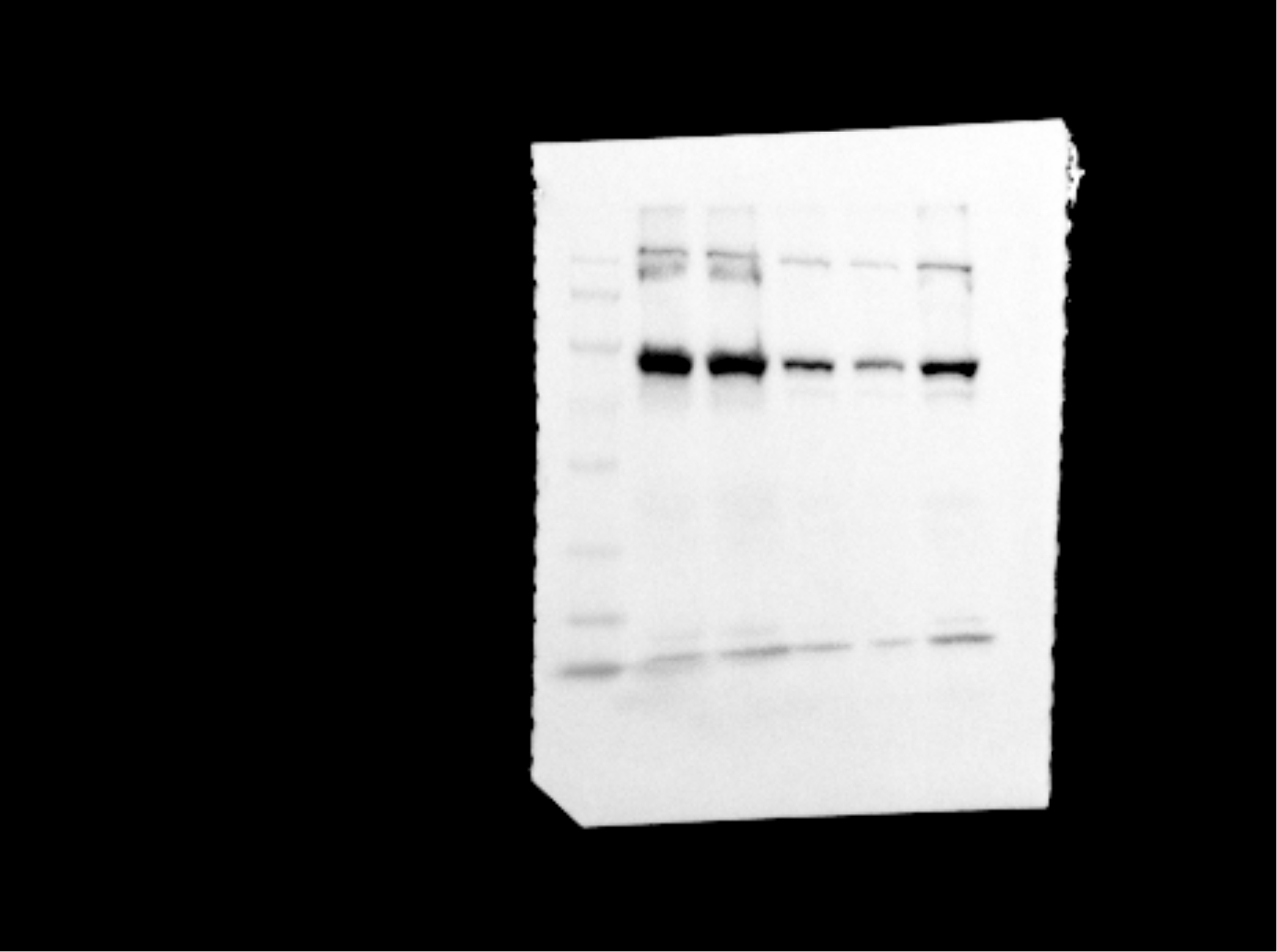

Supplement: Supplementary file 5 [file DataSheet6.zip › original images of figure 7/图7B-1.jpg]

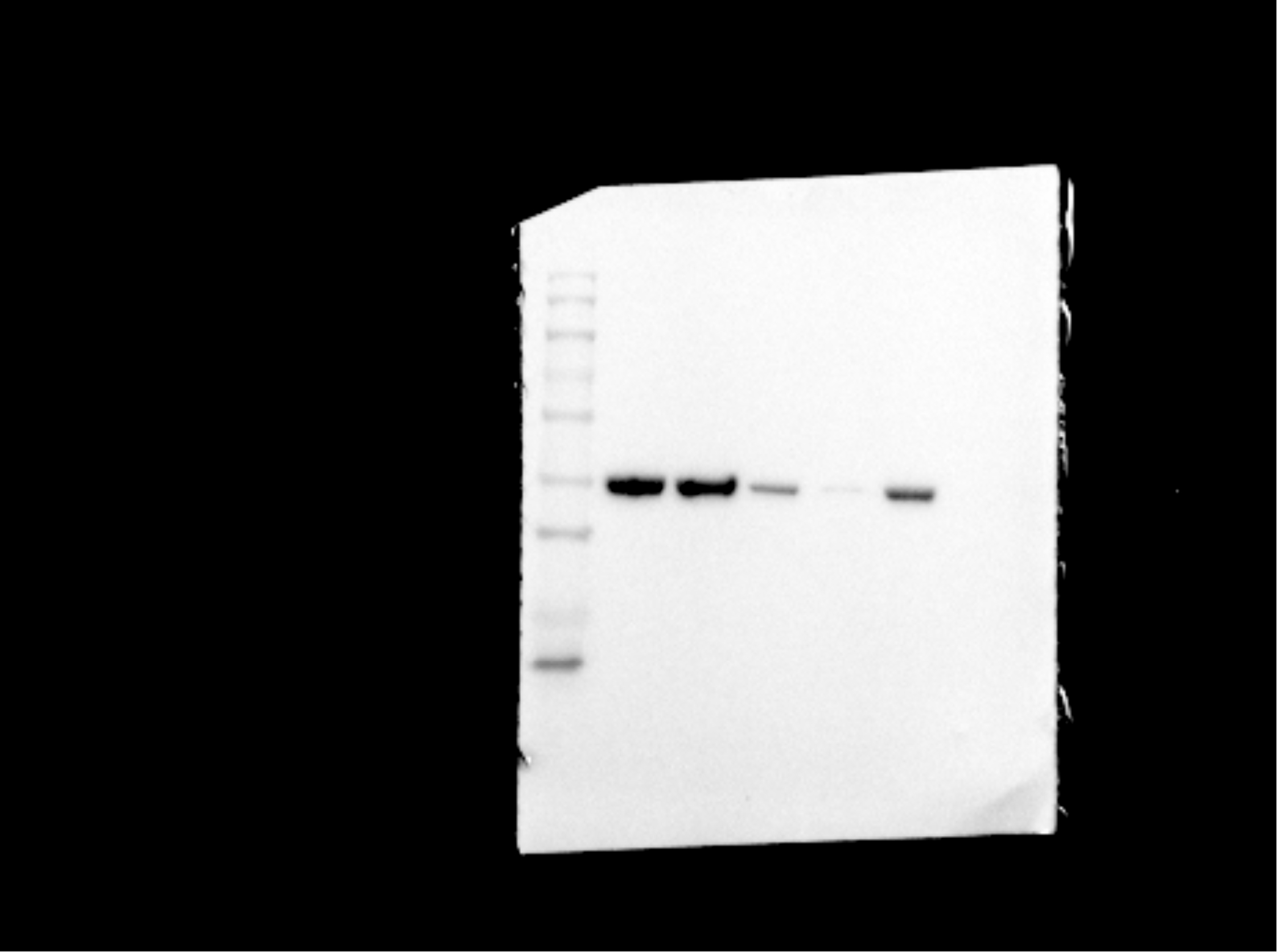

Supplement: Supplementary file 5 [file DataSheet6.zip › original images of figure 7/图7B-2.jpg]

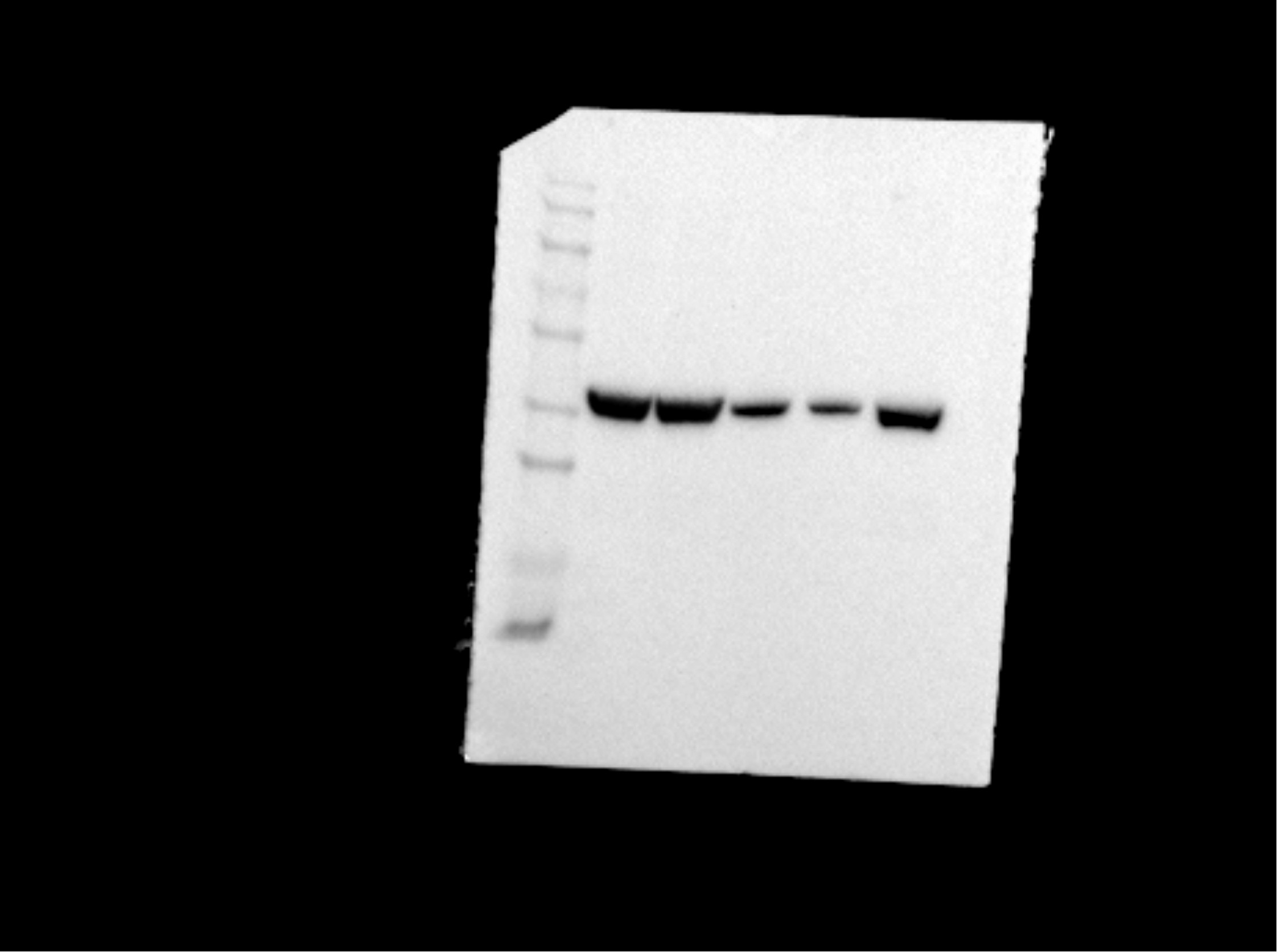

Supplement: Supplementary file 5 [file DataSheet6.zip › original images of figure 7/图7B-3.jpg]

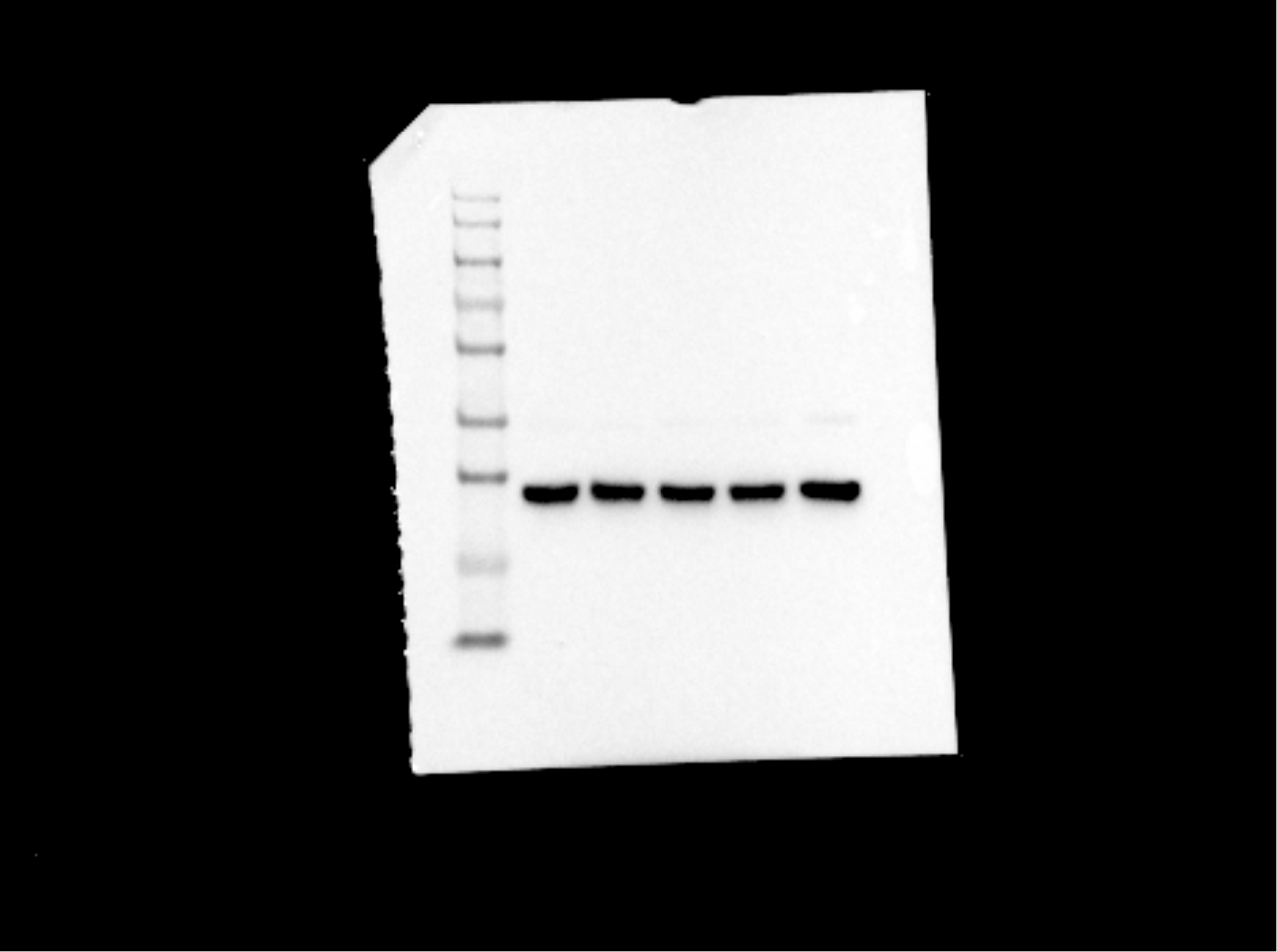

Supplement: Supplementary file 5 [file DataSheet6.zip › original images of figure 7/图7B-4.jpg]

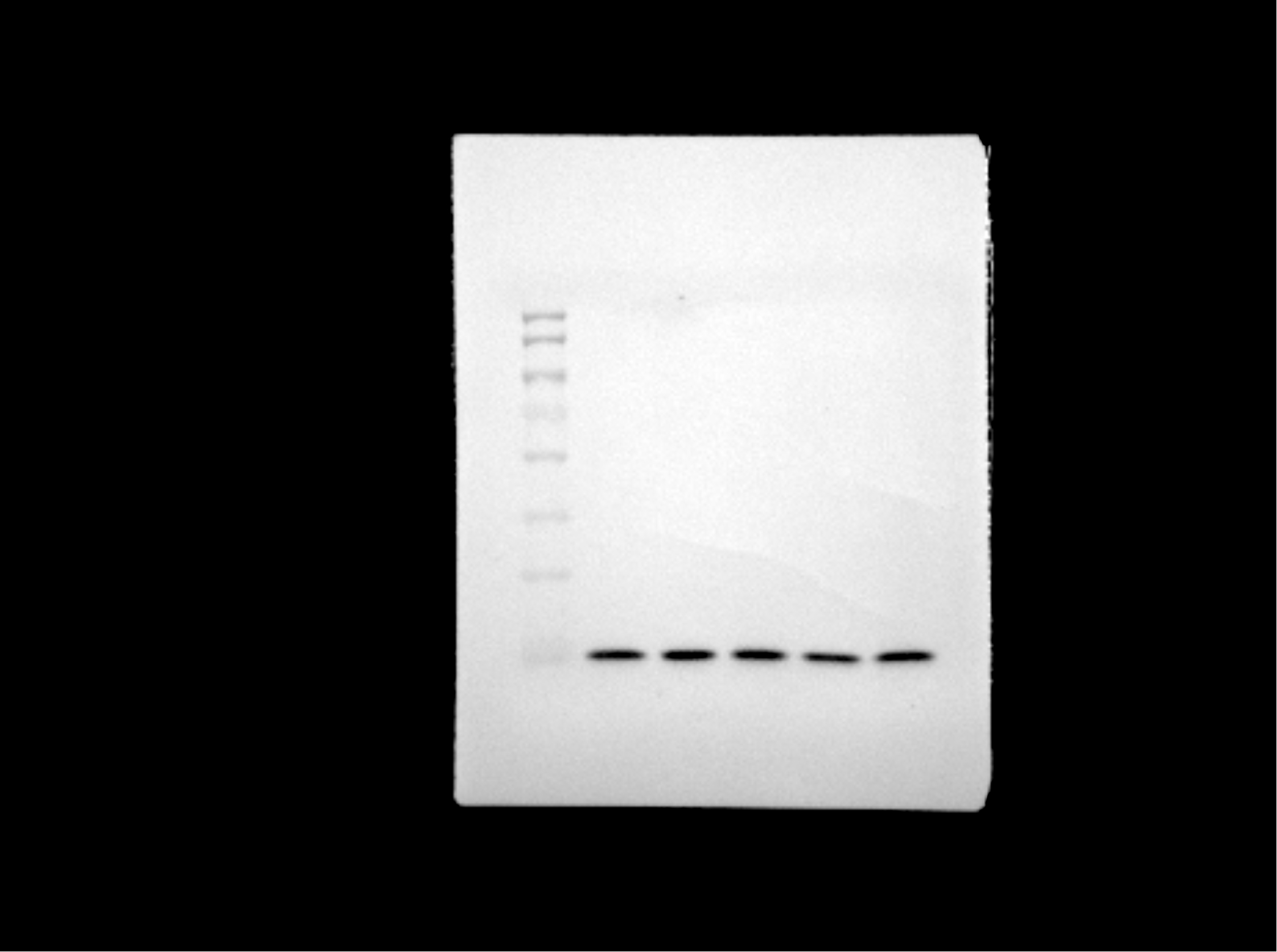

Supplement: Supplementary file 5 [file DataSheet6.zip › original images of figure 7/图7D-1.jpg]

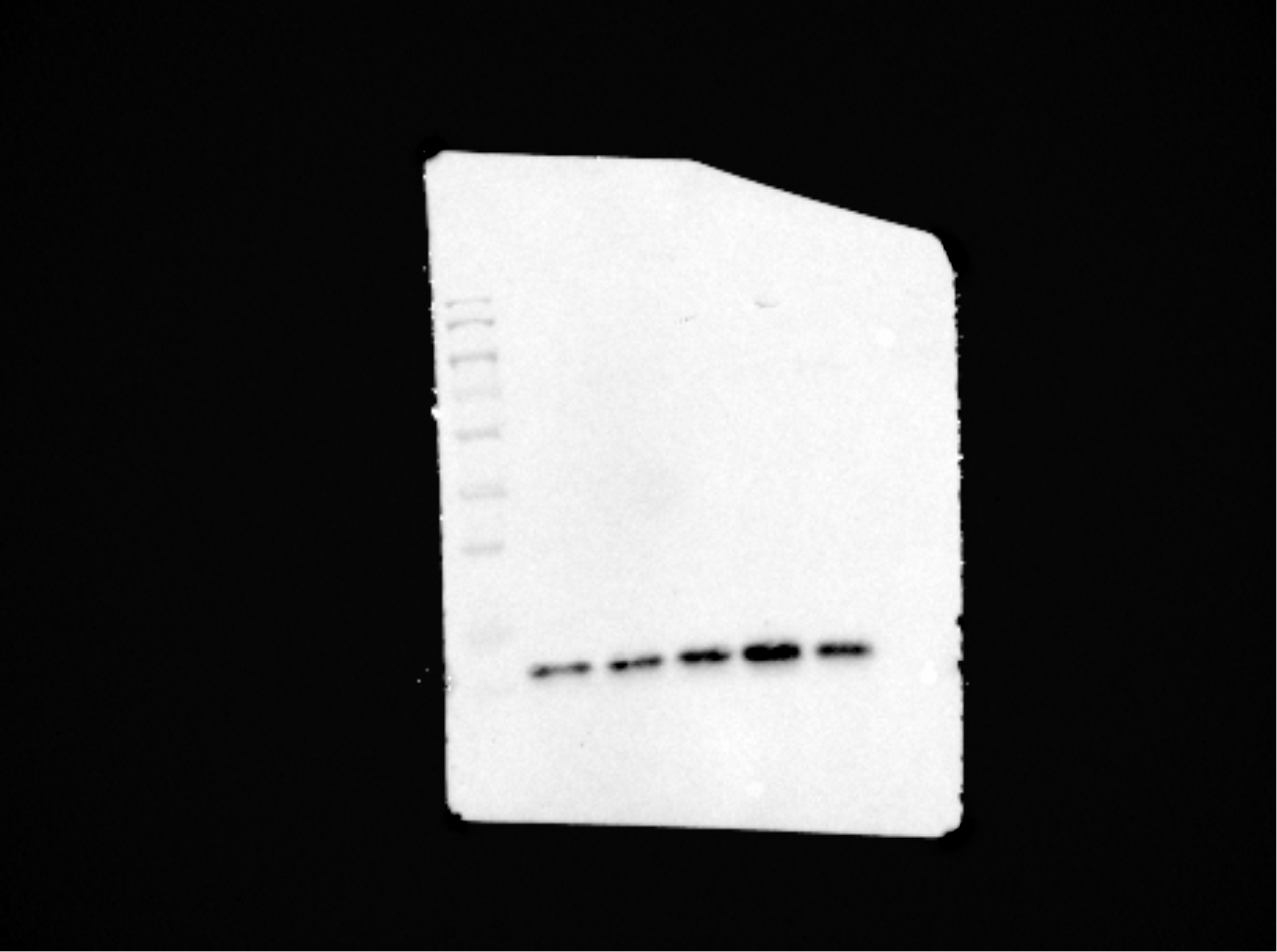

Supplement: Supplementary file 5 [file DataSheet6.zip › original images of figure 7/图7D-2.jpg]

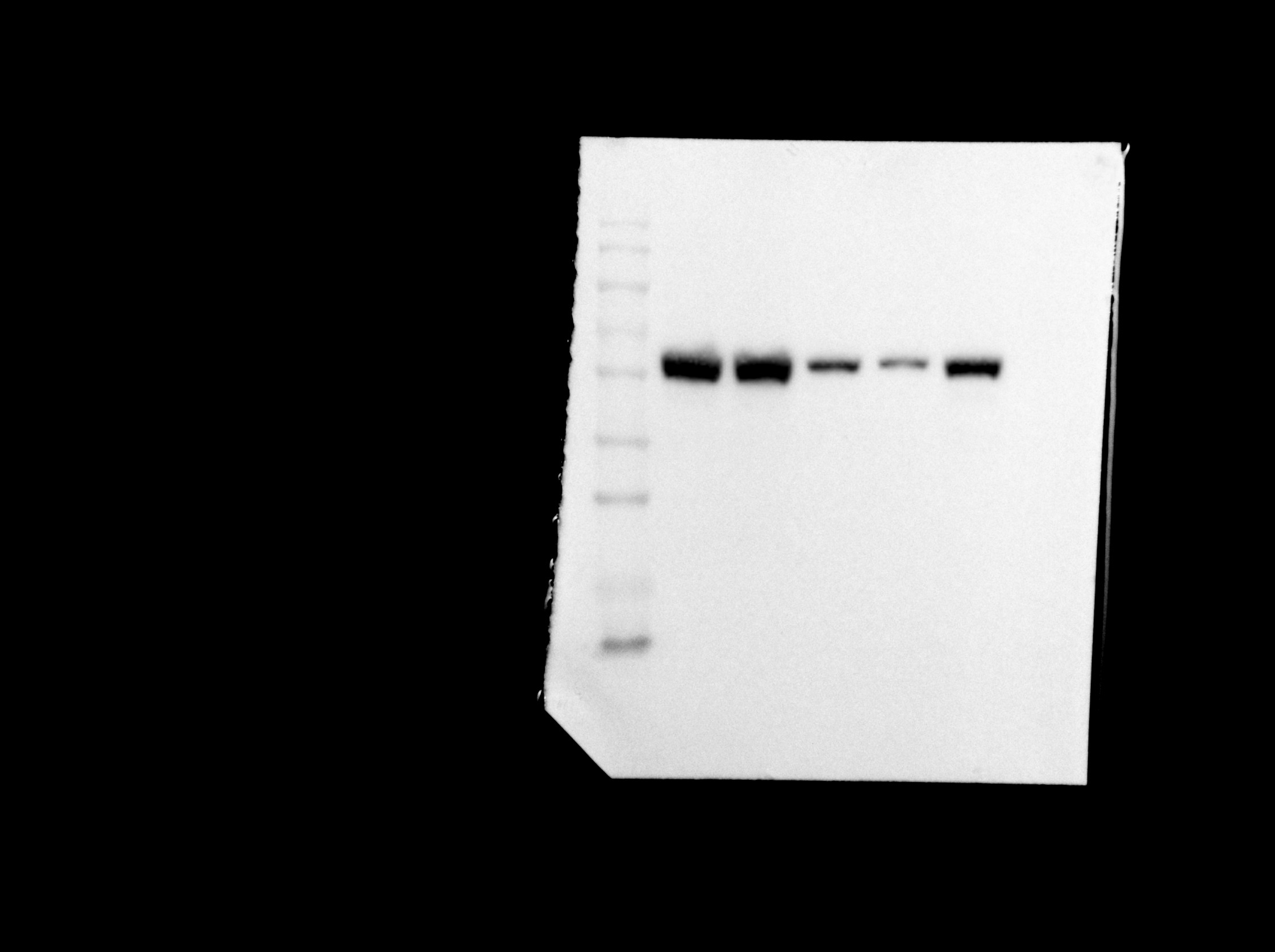

Supplement: Supplementary file 5 [file DataSheet6.zip › original images of figure 7/图7D-3.jpg]

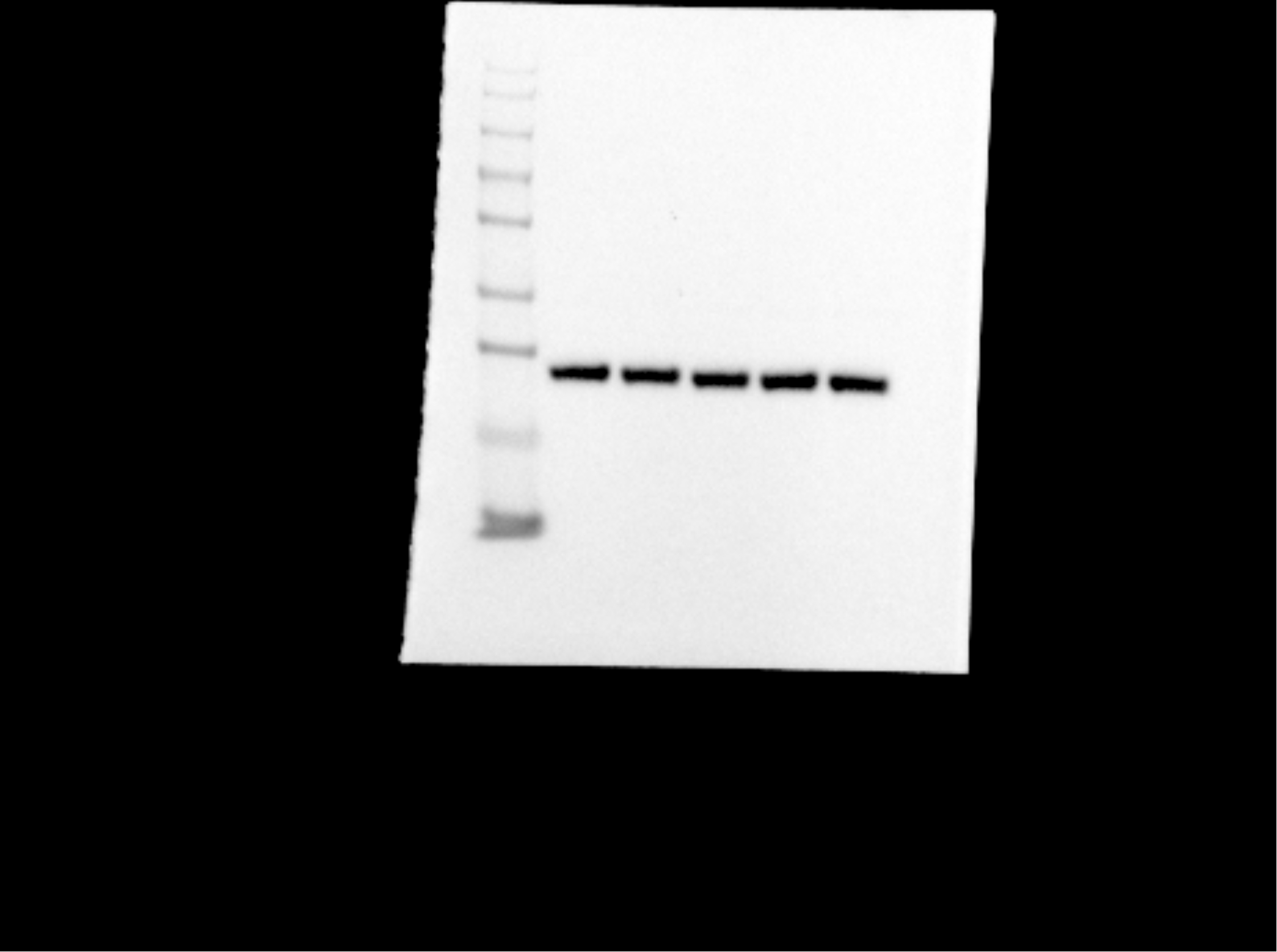

Supplement: Supplementary file 5 [file DataSheet6.zip › original images of figure 7/图7D-4.jpg]

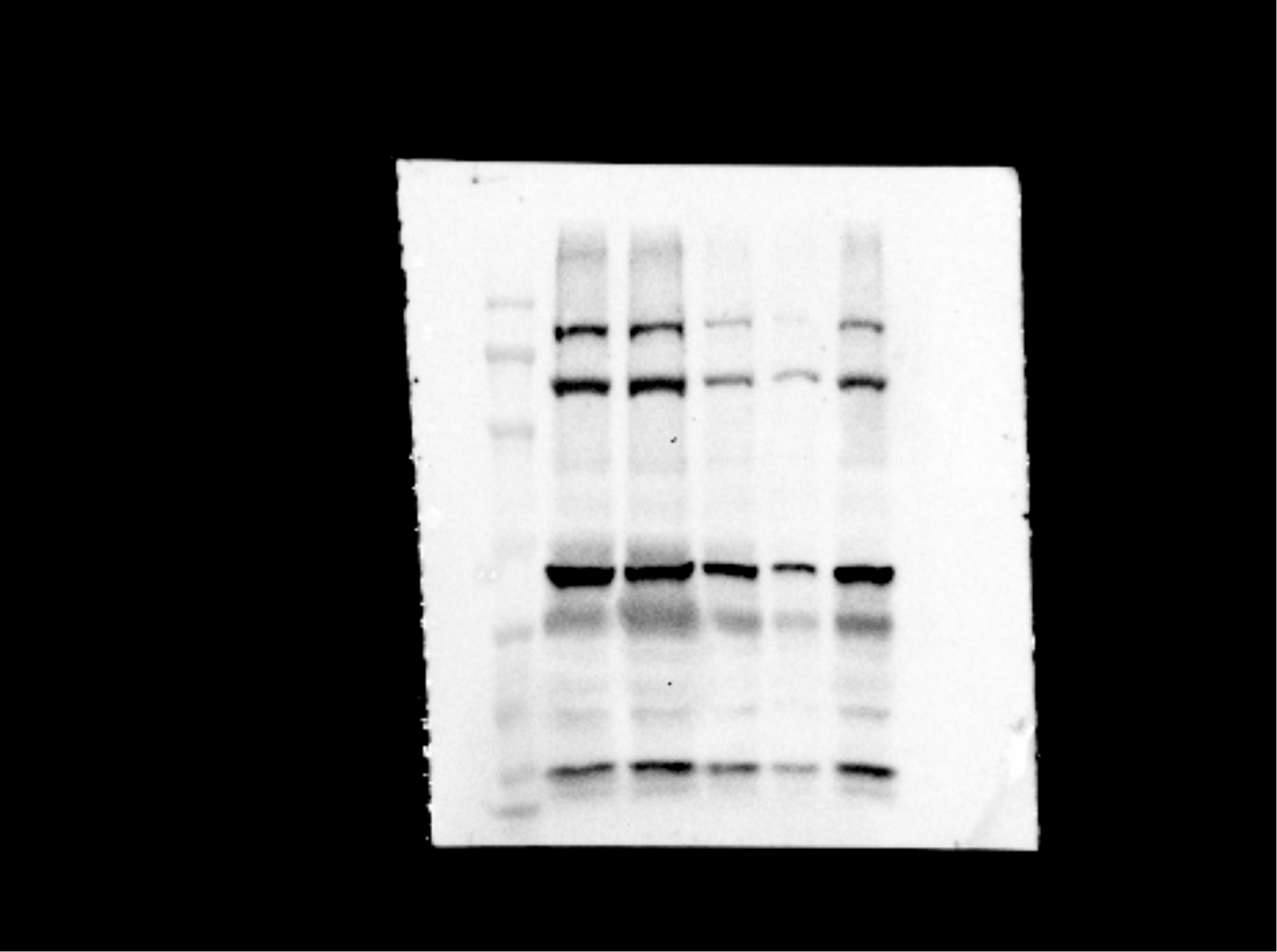

Supplement: Supplementary file 5 [file DataSheet6.zip › original images of figure 7/图7J-1.jpg]

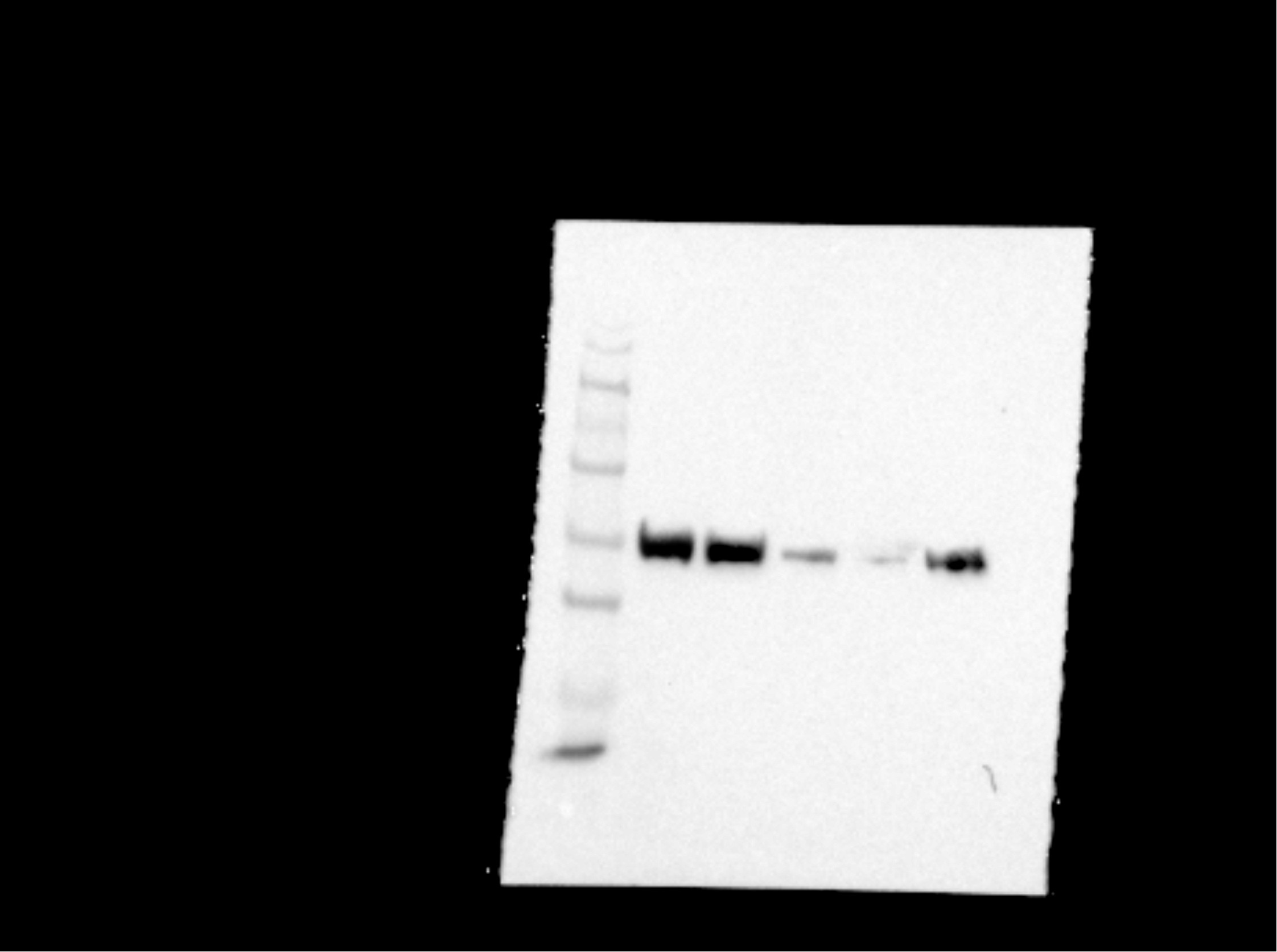

Supplement: Supplementary file 5 [file DataSheet6.zip › original images of figure 7/图7J-2.jpg]

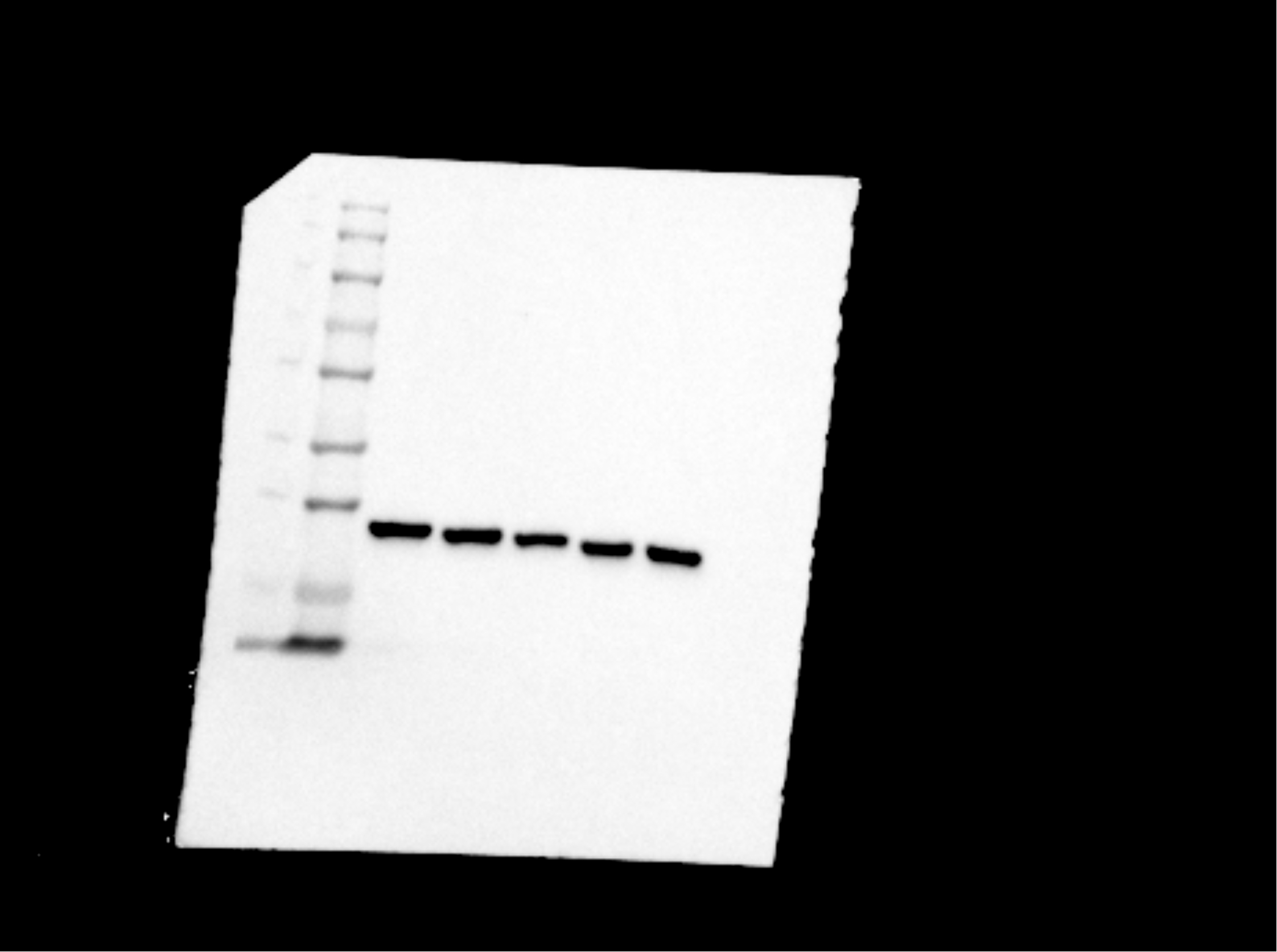

Supplement: Supplementary file 5 [file DataSheet6.zip › original images of figure 7/图7J-3.jpg]

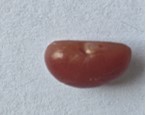

Supplement: Supplementary file 6 [file DataSheet2.zip › original images of figure 3/图3B-1.jpg]

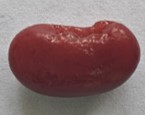

Supplement: Supplementary file 6 [file DataSheet2.zip › original images of figure 3/图3B-2.jpg]

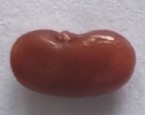

Supplement: Supplementary file 6 [file DataSheet2.zip › original images of figure 3/图3B-3.jpg]

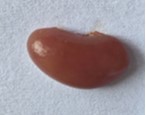

Supplement: Supplementary file 6 [file DataSheet2.zip › original images of figure 3/图3B-4.jpg]

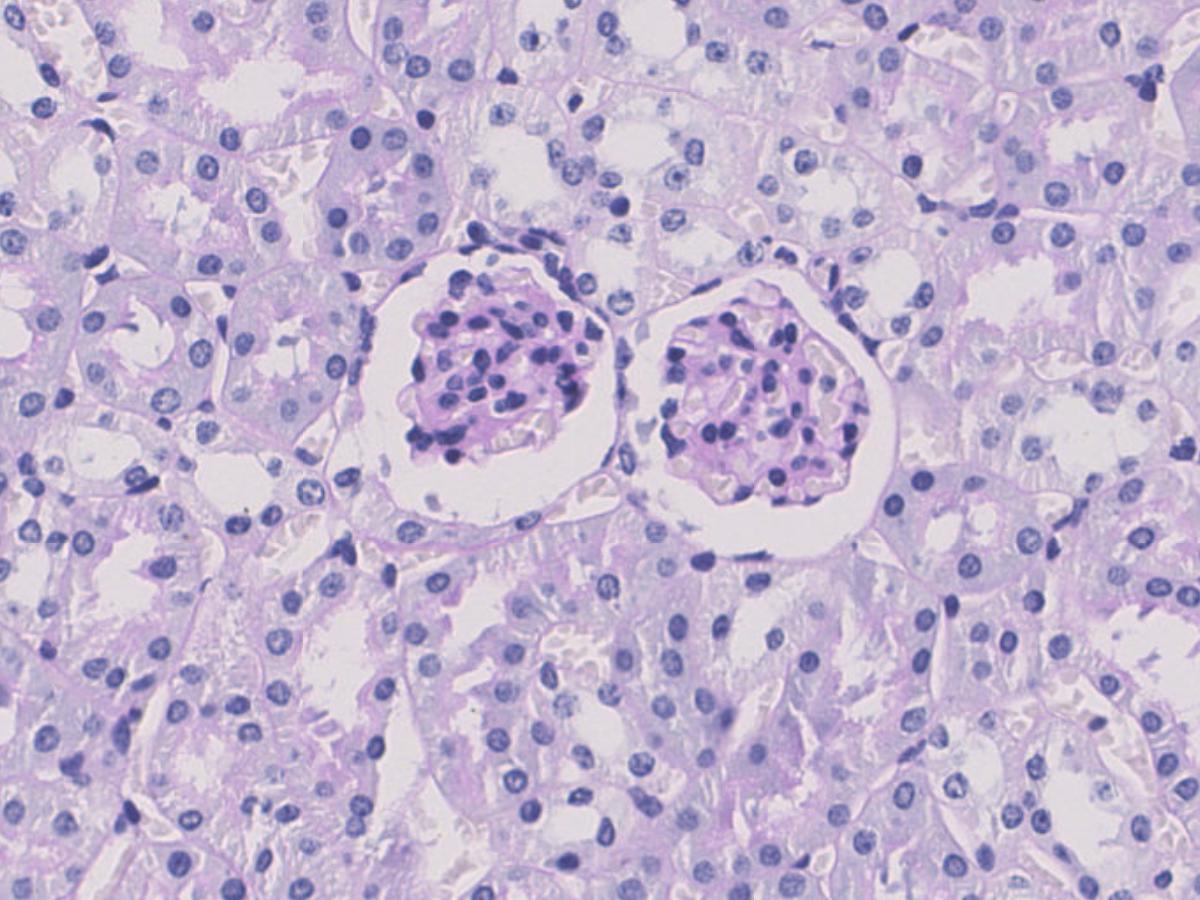

Supplement: Supplementary file 6 [file DataSheet2.zip › original images of figure 3/图3E-1.jpg]

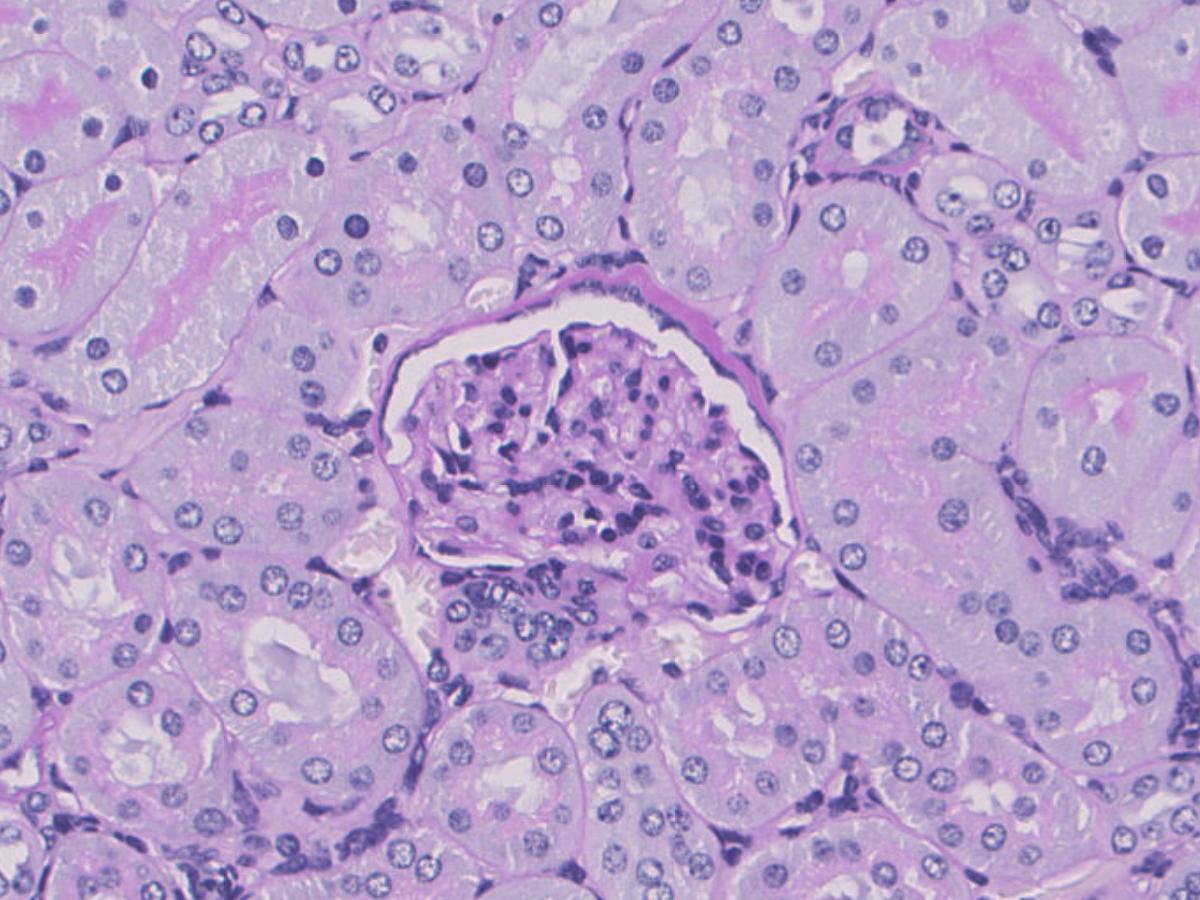

Supplement: Supplementary file 6 [file DataSheet2.zip › original images of figure 3/图3E-2.jpg]

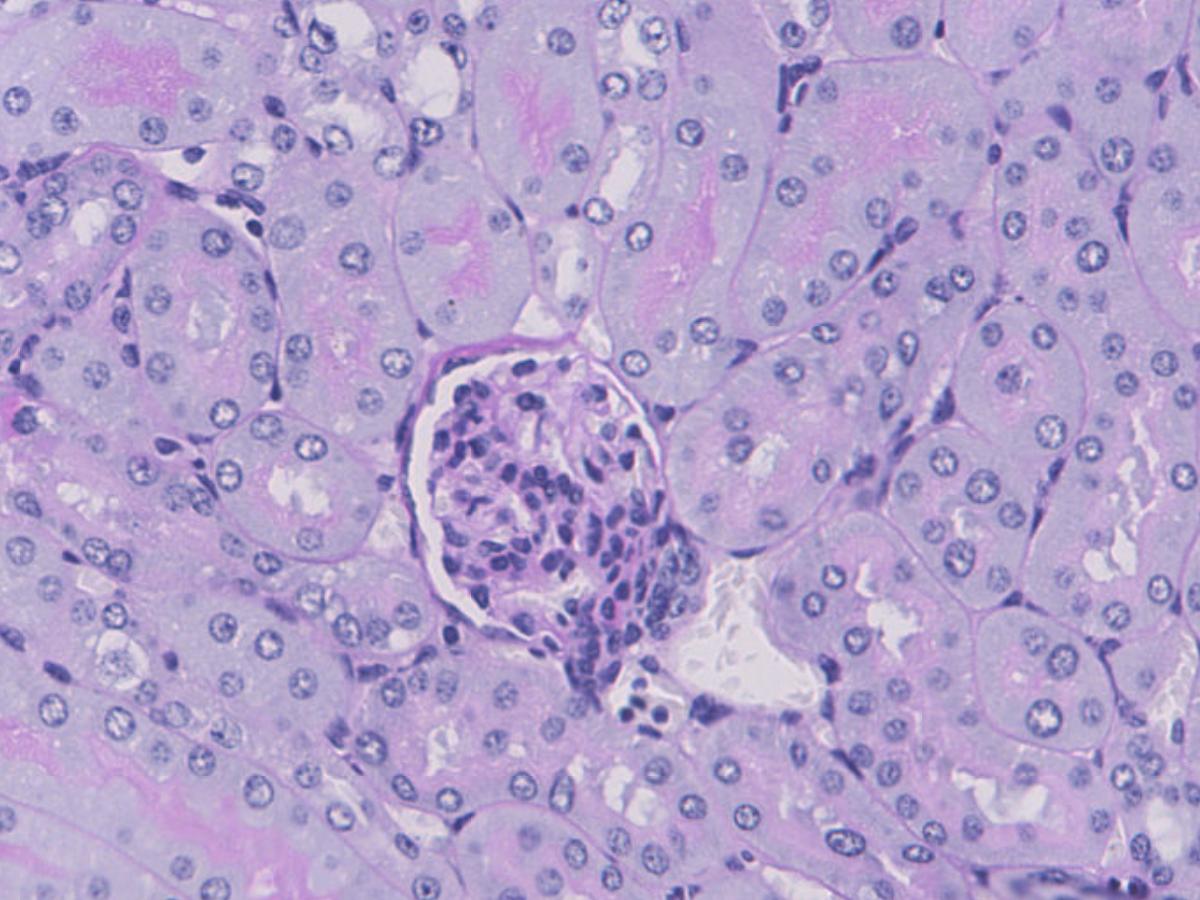

Supplement: Supplementary file 6 [file DataSheet2.zip › original images of figure 3/图3E-3.jpg]

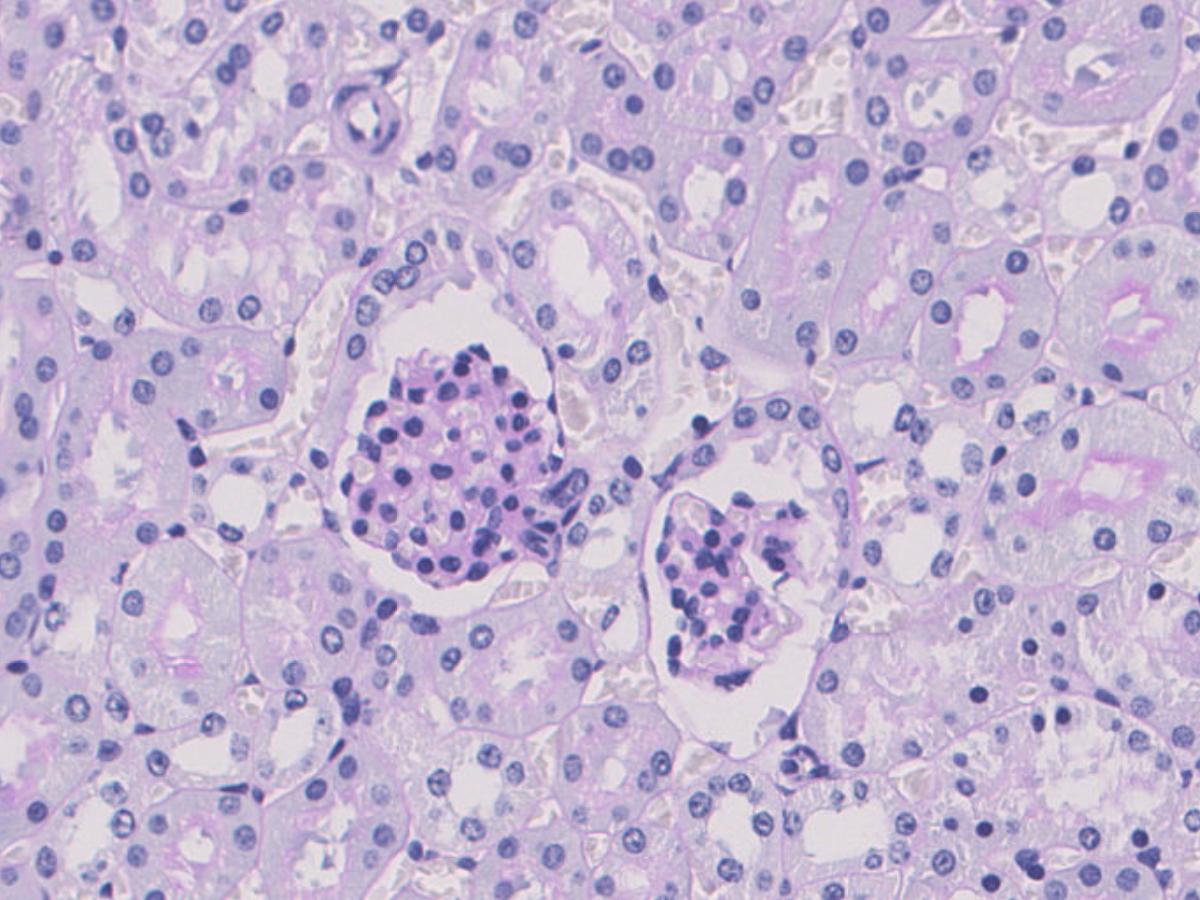

Supplement: Supplementary file 6 [file DataSheet2.zip › original images of figure 3/图3E-4.jpg]

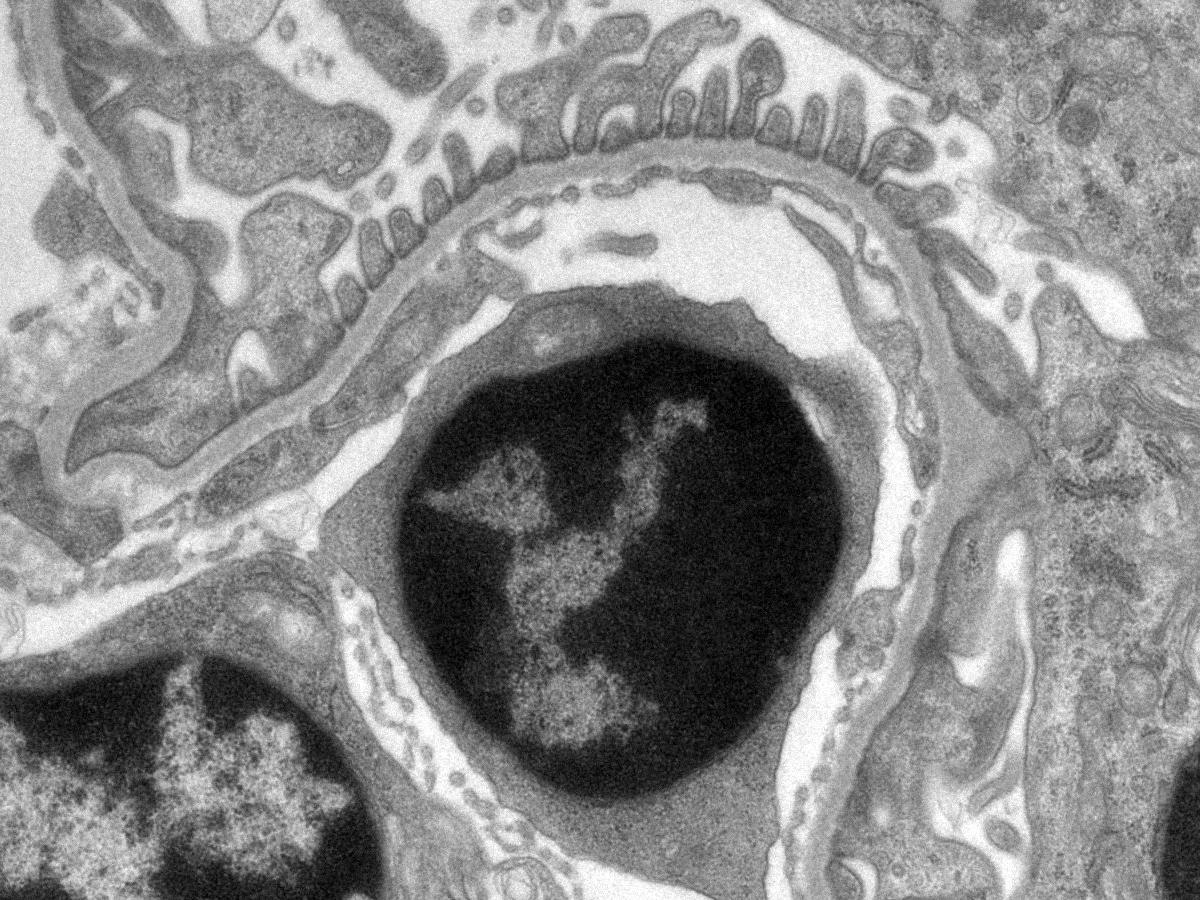

Supplement: Supplementary file 6 [file DataSheet2.zip › original images of figure 3/图3F-1.jpg]

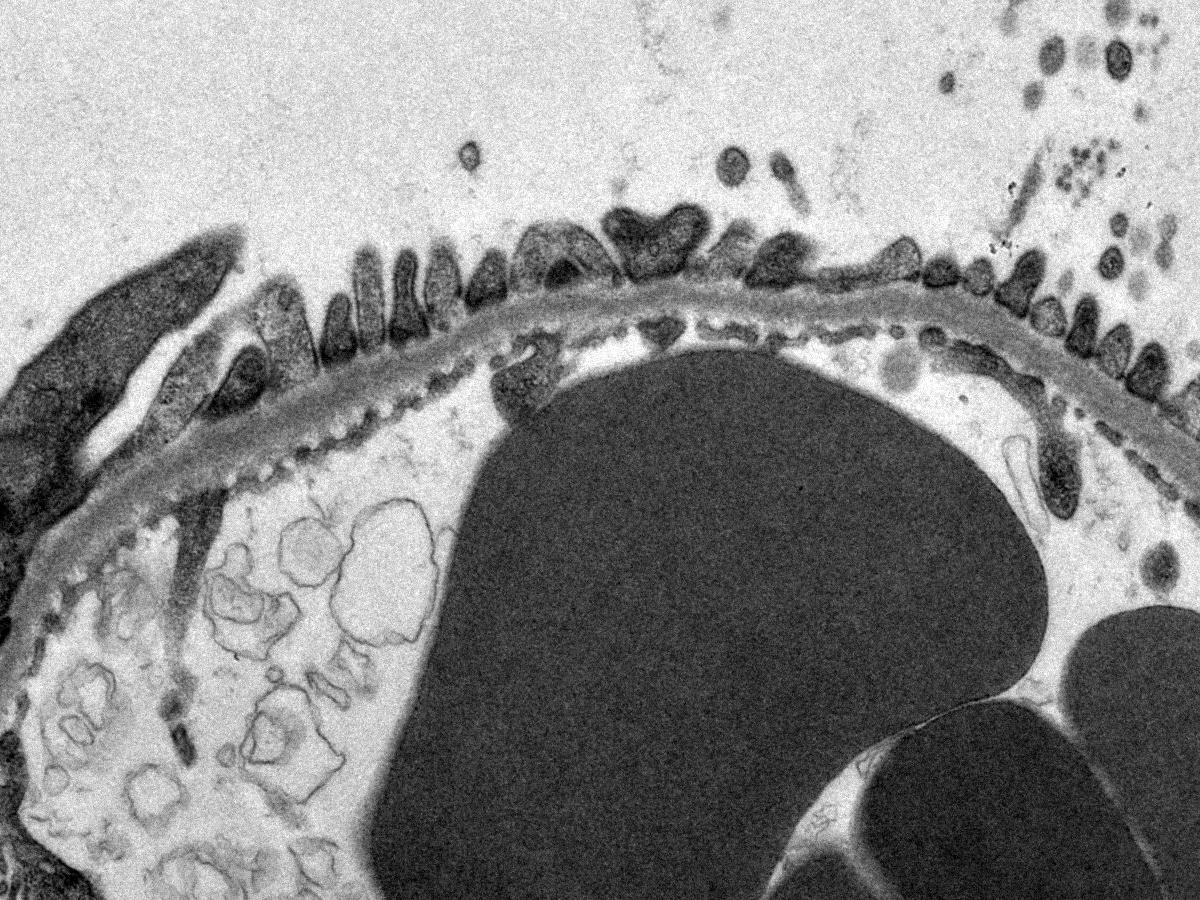

Supplement: Supplementary file 6 [file DataSheet2.zip › original images of figure 3/图3F-2.jpg]

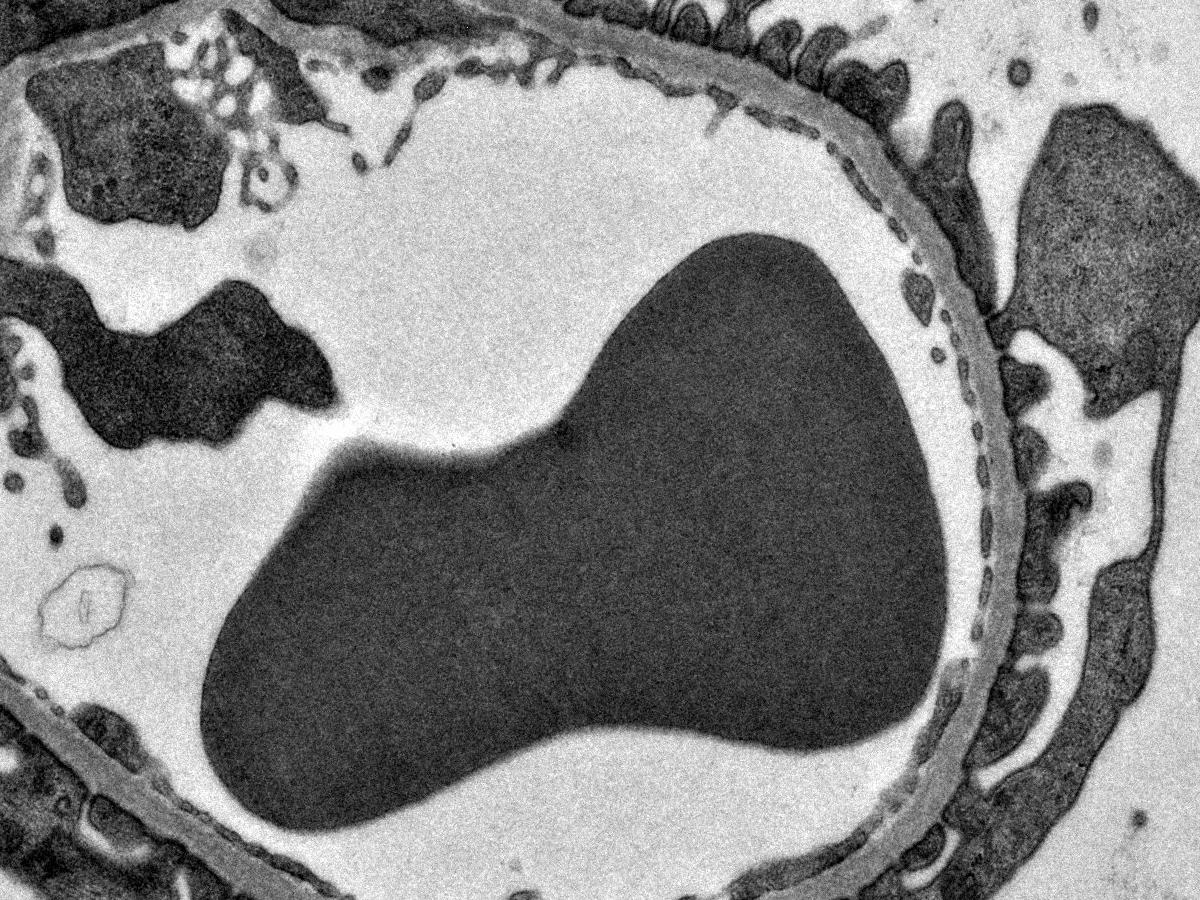

Supplement: Supplementary file 6 [file DataSheet2.zip › original images of figure 3/图3F-3.jpg]

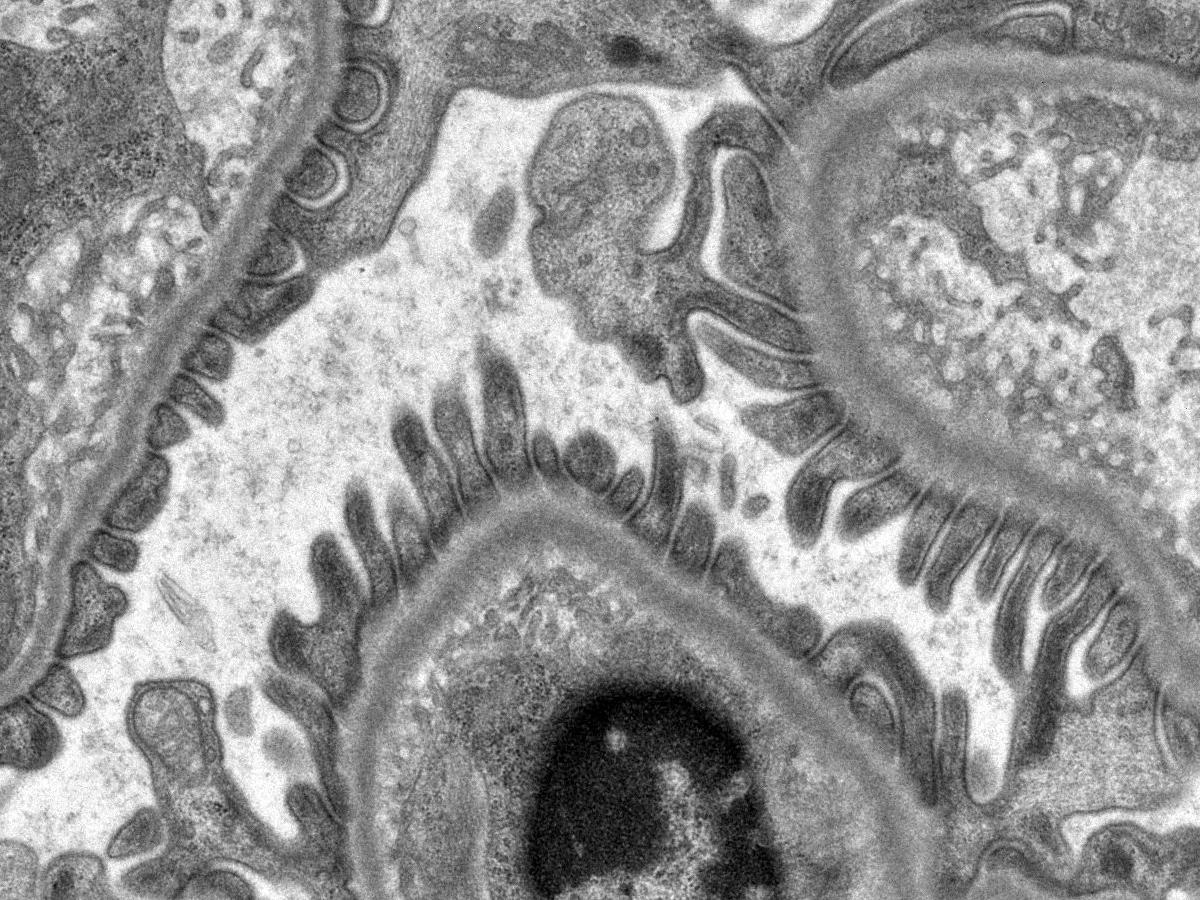

Supplement: Supplementary file 6 [file DataSheet2.zip › original images of figure 3/图3F-4.jpg]

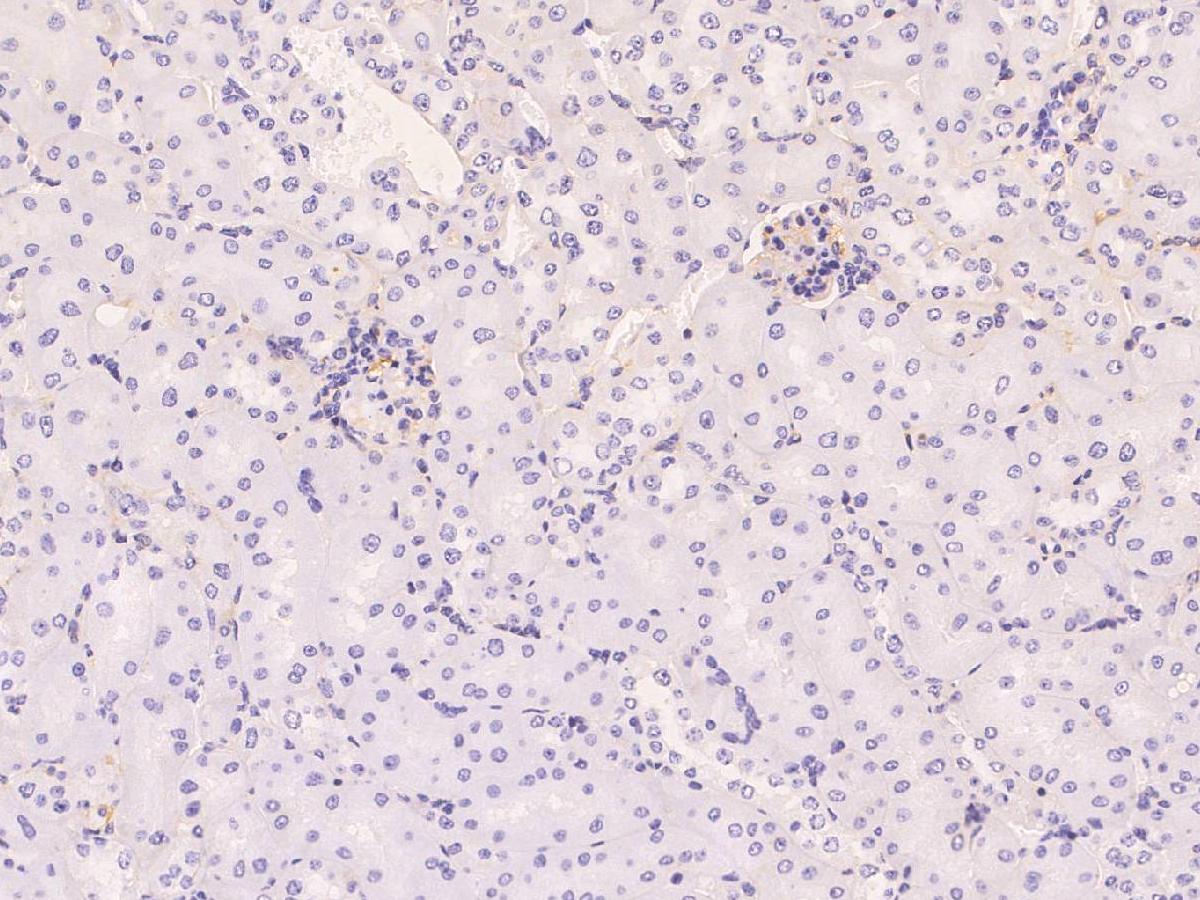

Supplement: Supplementary file 6 [file DataSheet2.zip › original images of figure 3/图3H-1-1.jpg]

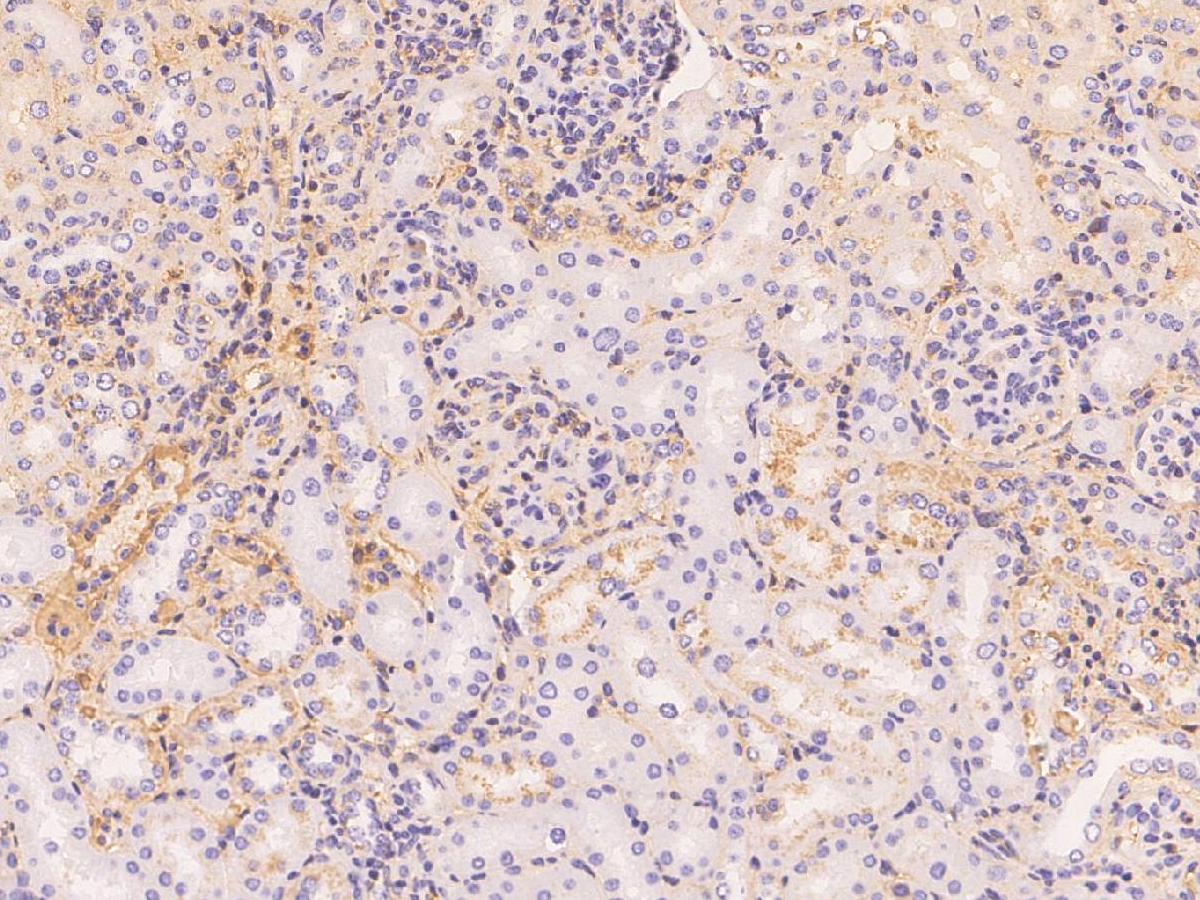

Supplement: Supplementary file 6 [file DataSheet2.zip › original images of figure 3/图3H-1-2.jpg]

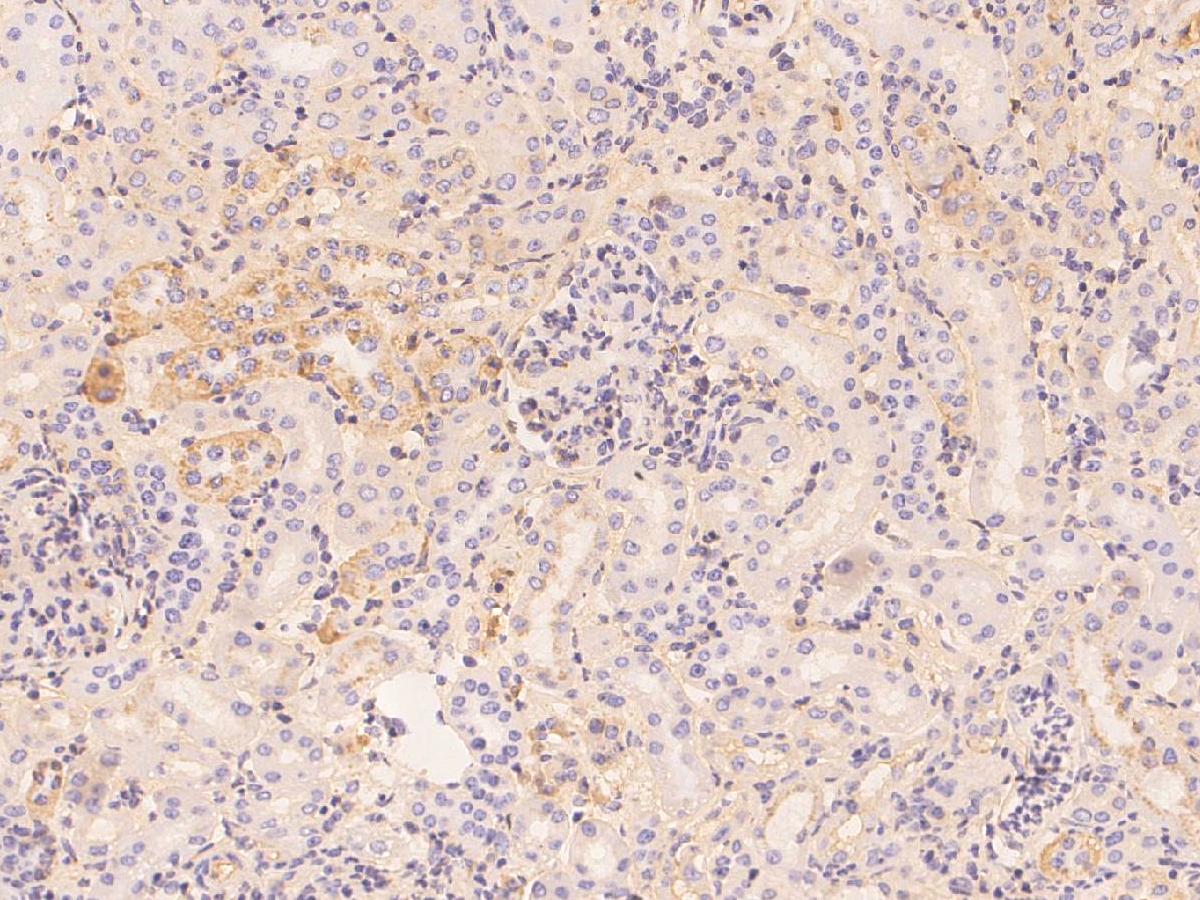

Supplement: Supplementary file 6 [file DataSheet2.zip › original images of figure 3/图3H-1-3.jpg]

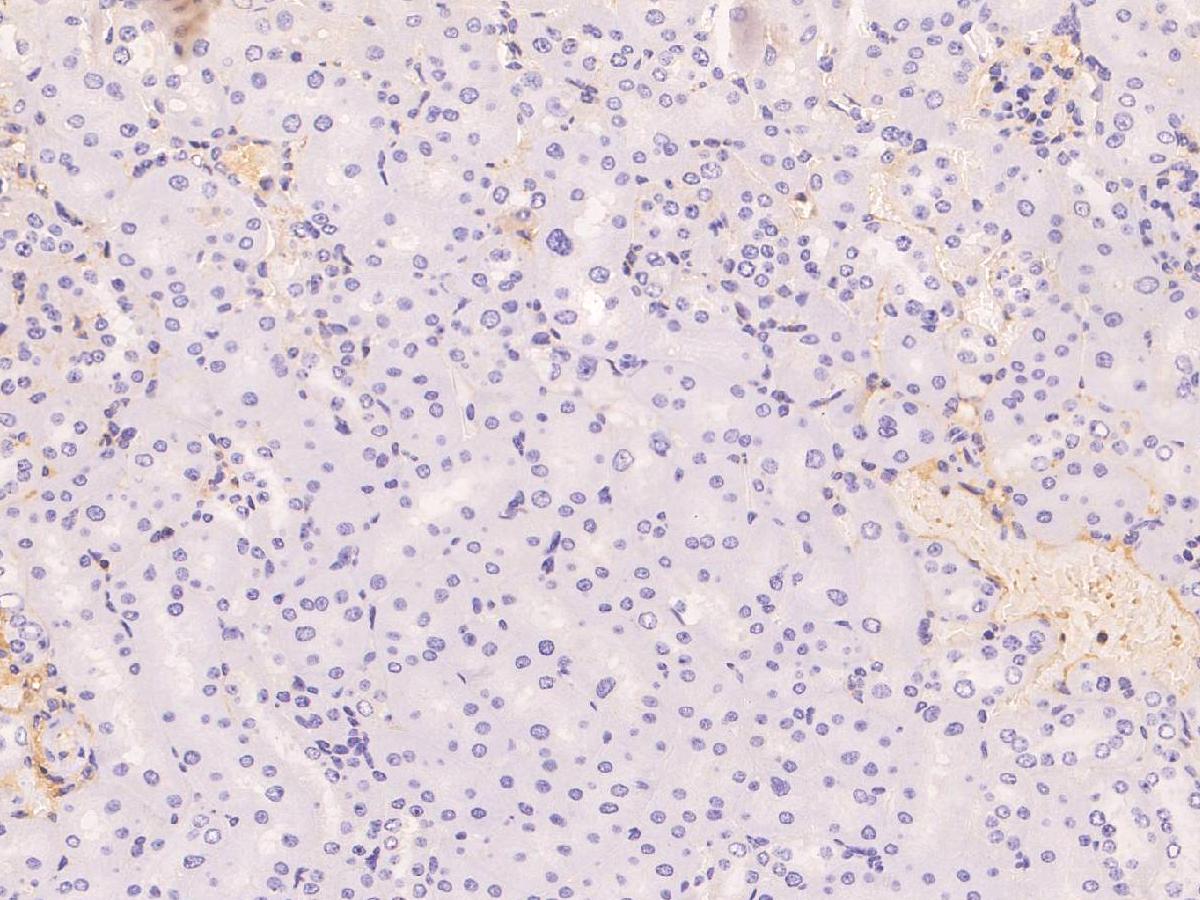

Supplement: Supplementary file 6 [file DataSheet2.zip › original images of figure 3/图3H-1-4.jpg]

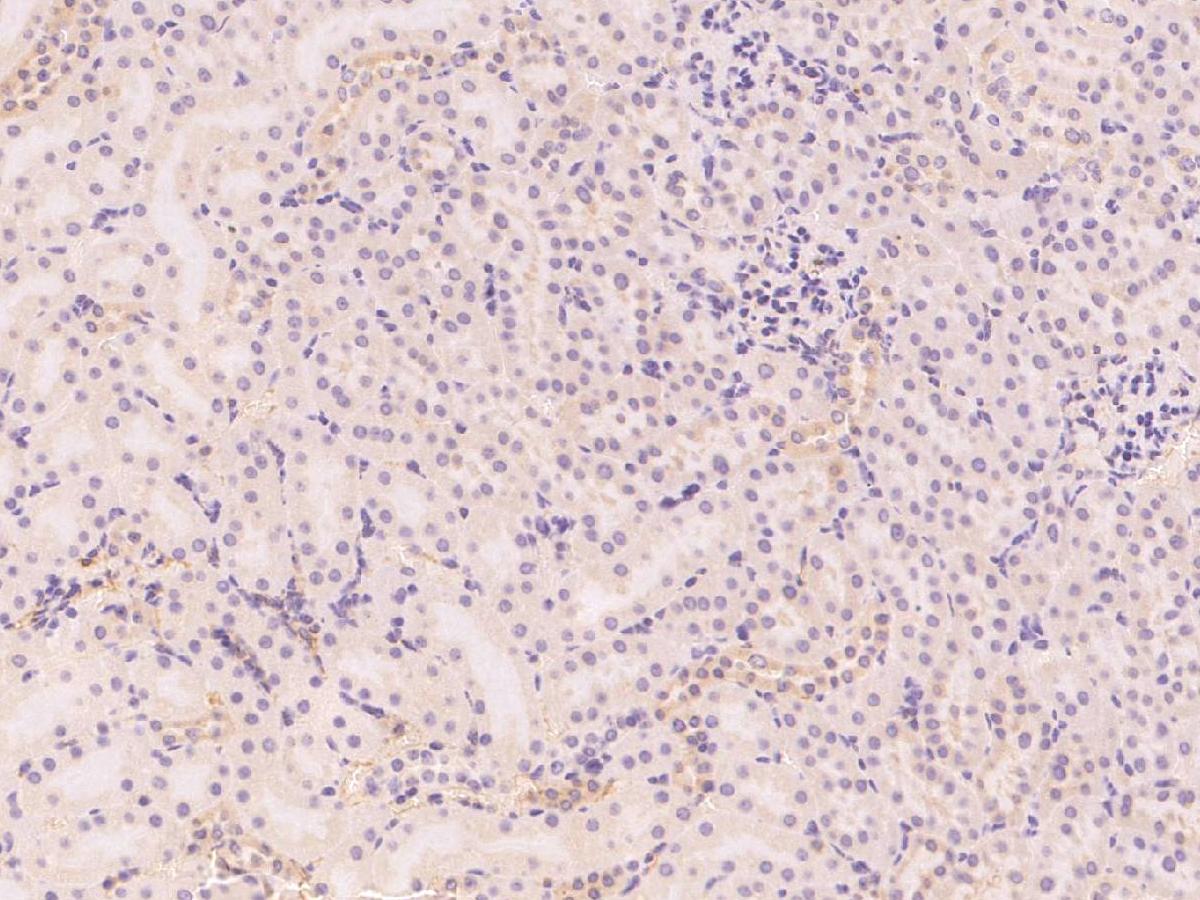

Supplement: Supplementary file 6 [file DataSheet2.zip › original images of figure 3/图3H-2-1.jpg]

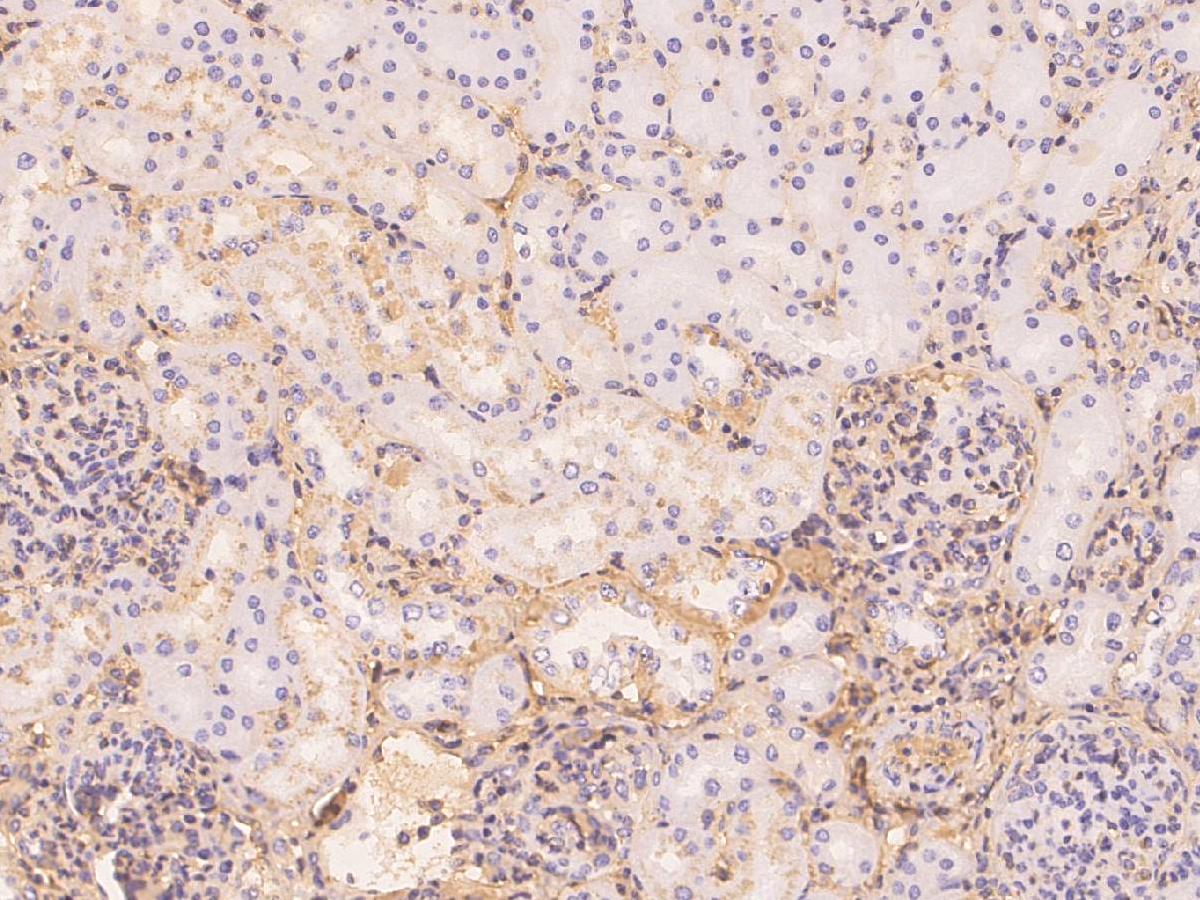

Supplement: Supplementary file 6 [file DataSheet2.zip › original images of figure 3/图3H-2-2.jpg]

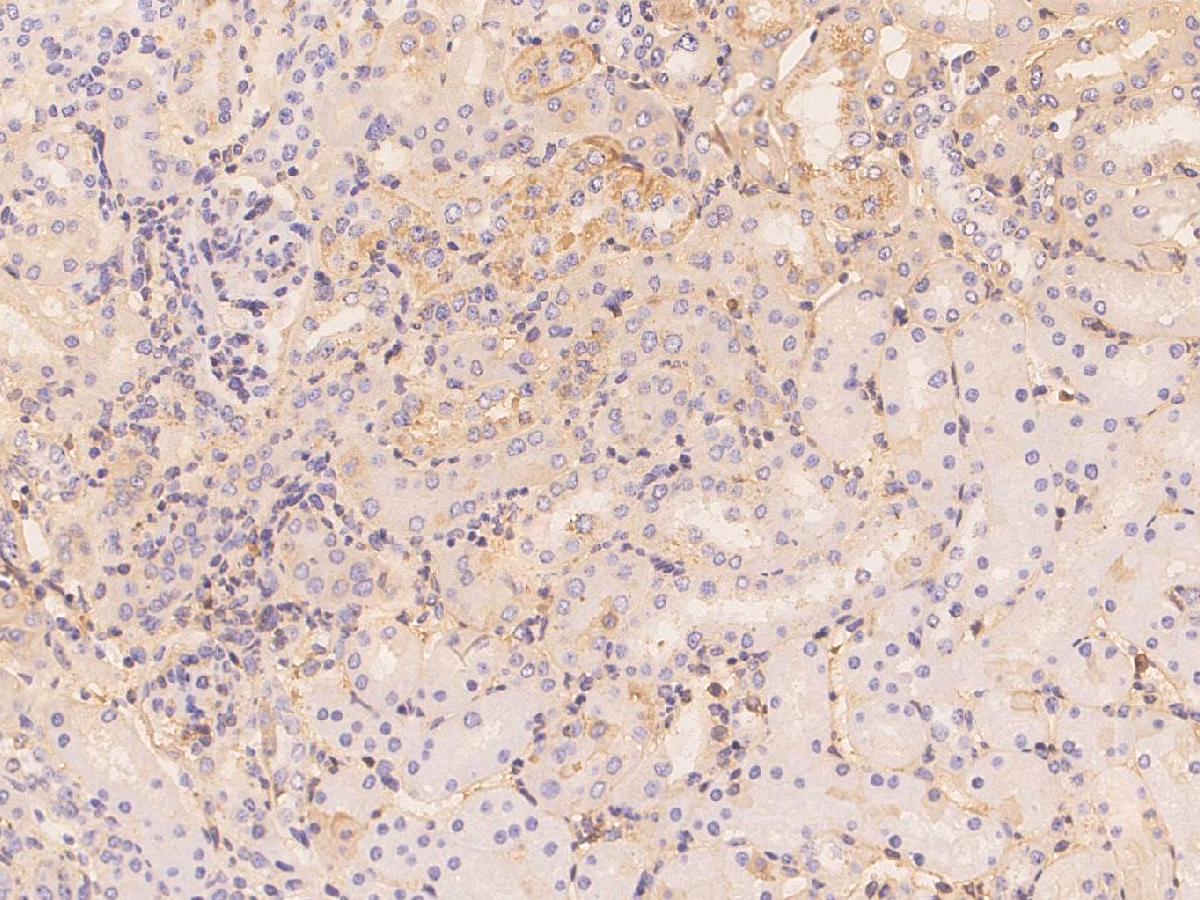

Supplement: Supplementary file 6 [file DataSheet2.zip › original images of figure 3/图3H-2-3.jpg]

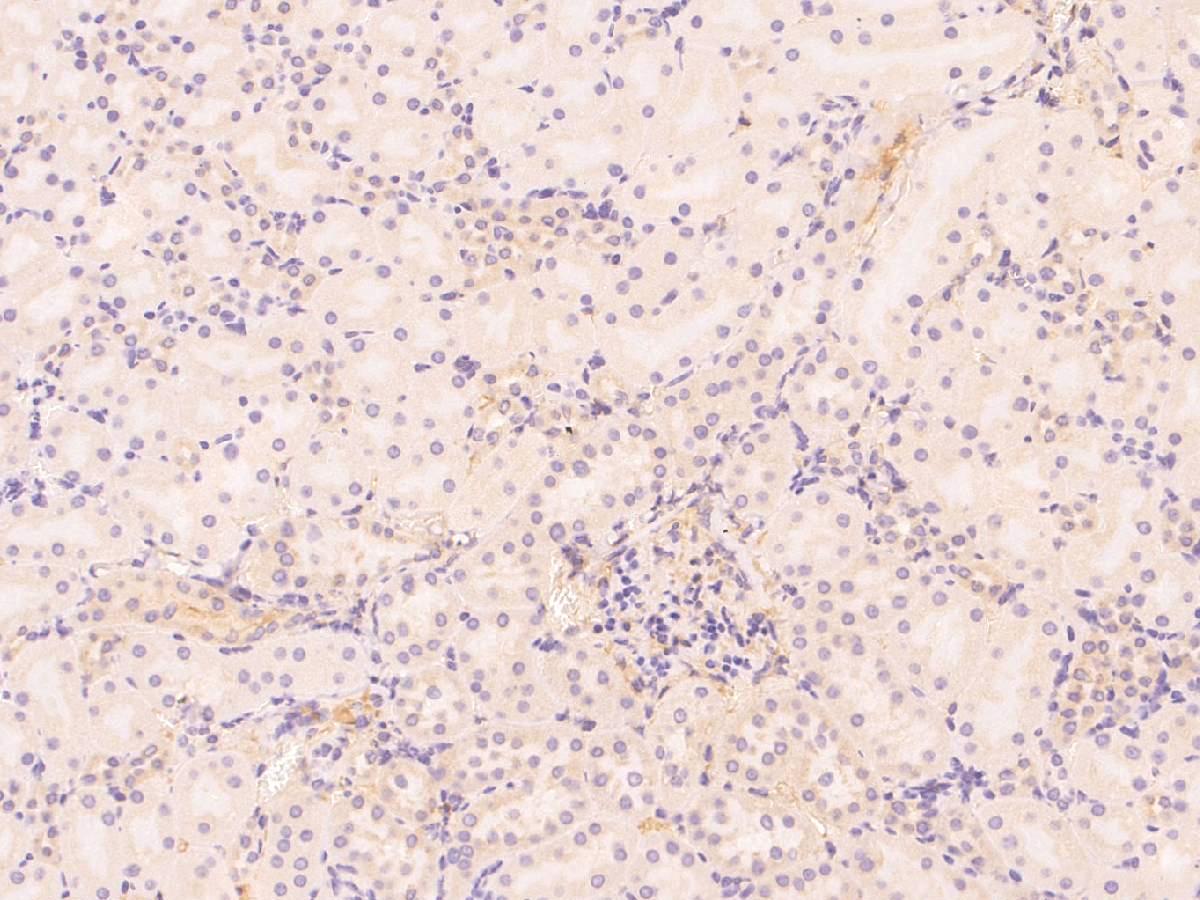

Supplement: Supplementary file 6 [file DataSheet2.zip › original images of figure 3/图3H-2-4.jpg]

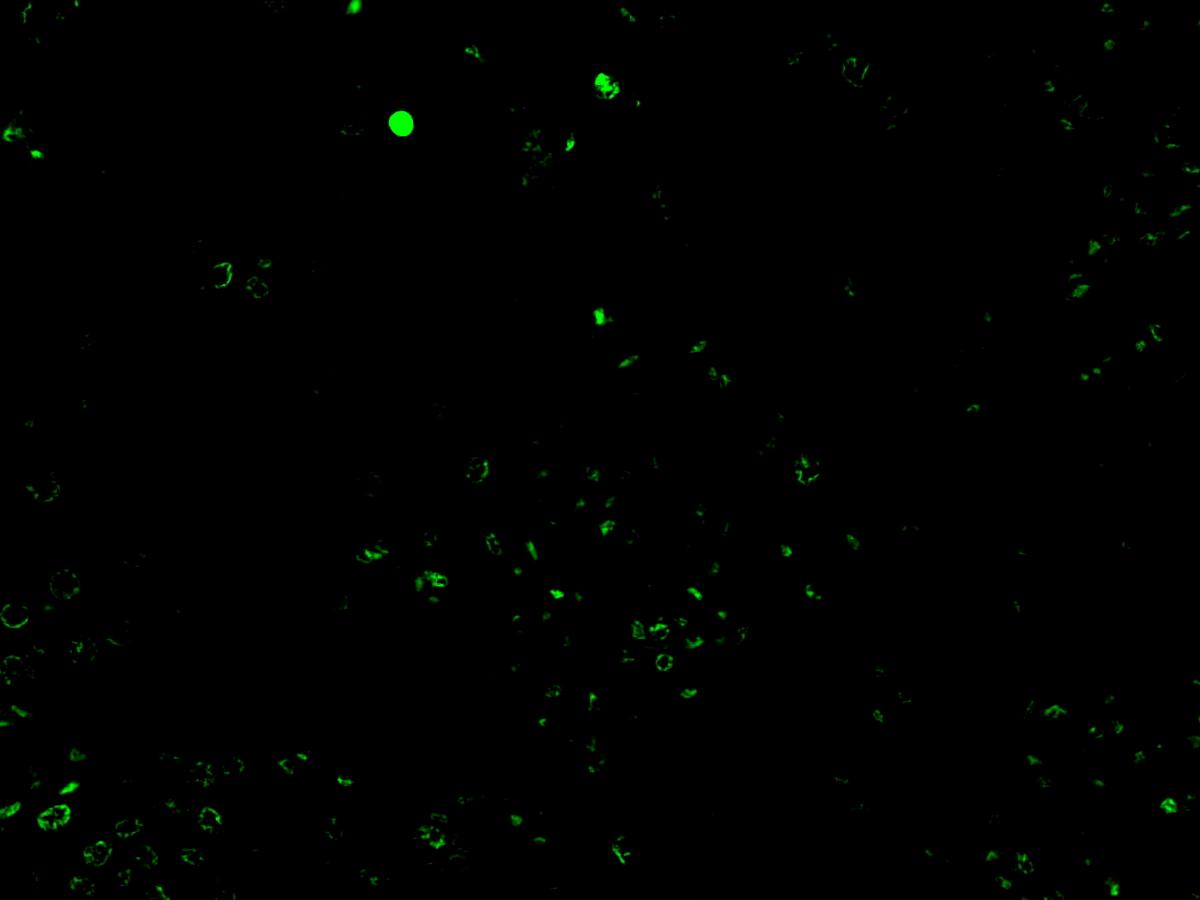

Supplement: Supplementary file 6 [file DataSheet2.zip › original images of figure 3/图3I-1-1(PARP1).jpg]

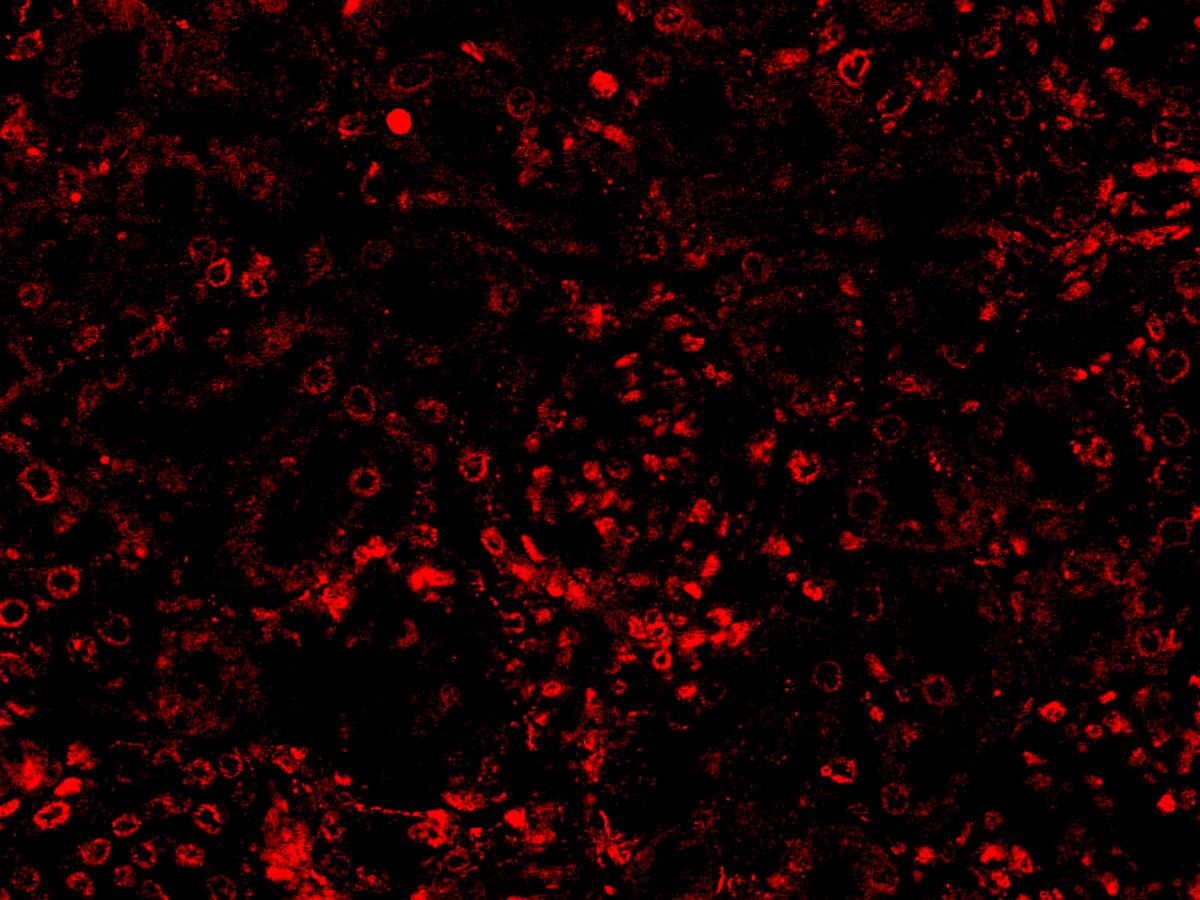

Supplement: Supplementary file 6 [file DataSheet2.zip › original images of figure 3/图3I-1-2(Synaptopodin).jpg]

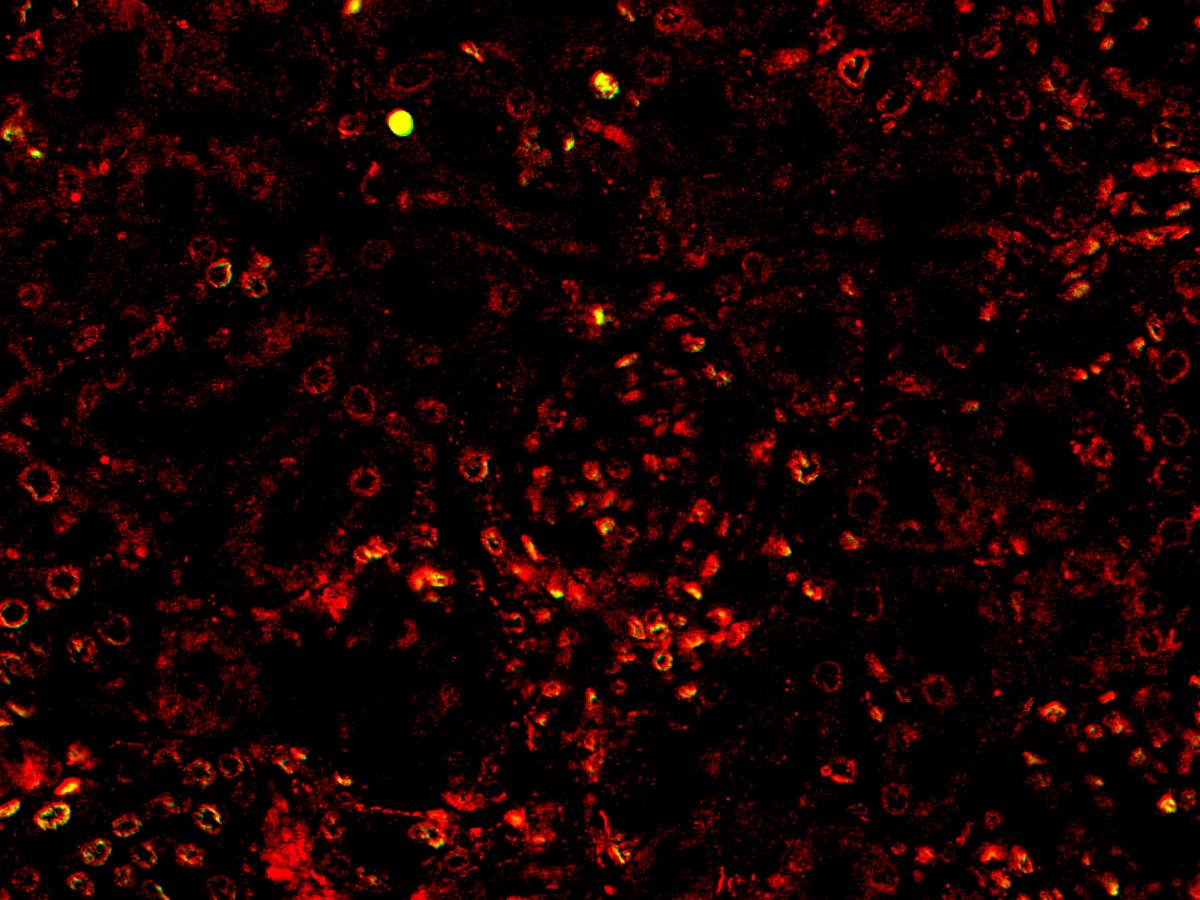

Supplement: Supplementary file 6 [file DataSheet2.zip › original images of figure 3/图3I-1-3(Merge).jpg]

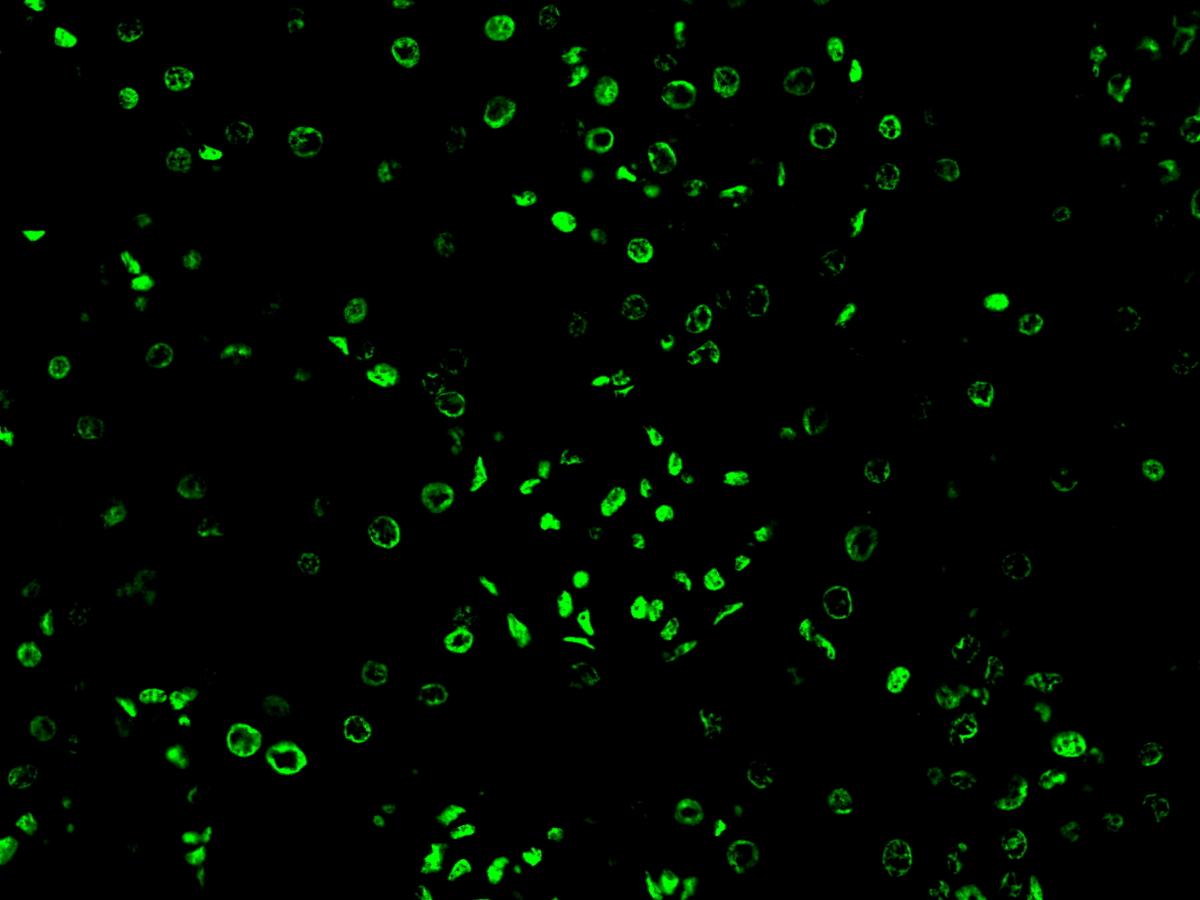

Supplement: Supplementary file 6 [file DataSheet2.zip › original images of figure 3/图3I-2-1(PARP1).jpg]

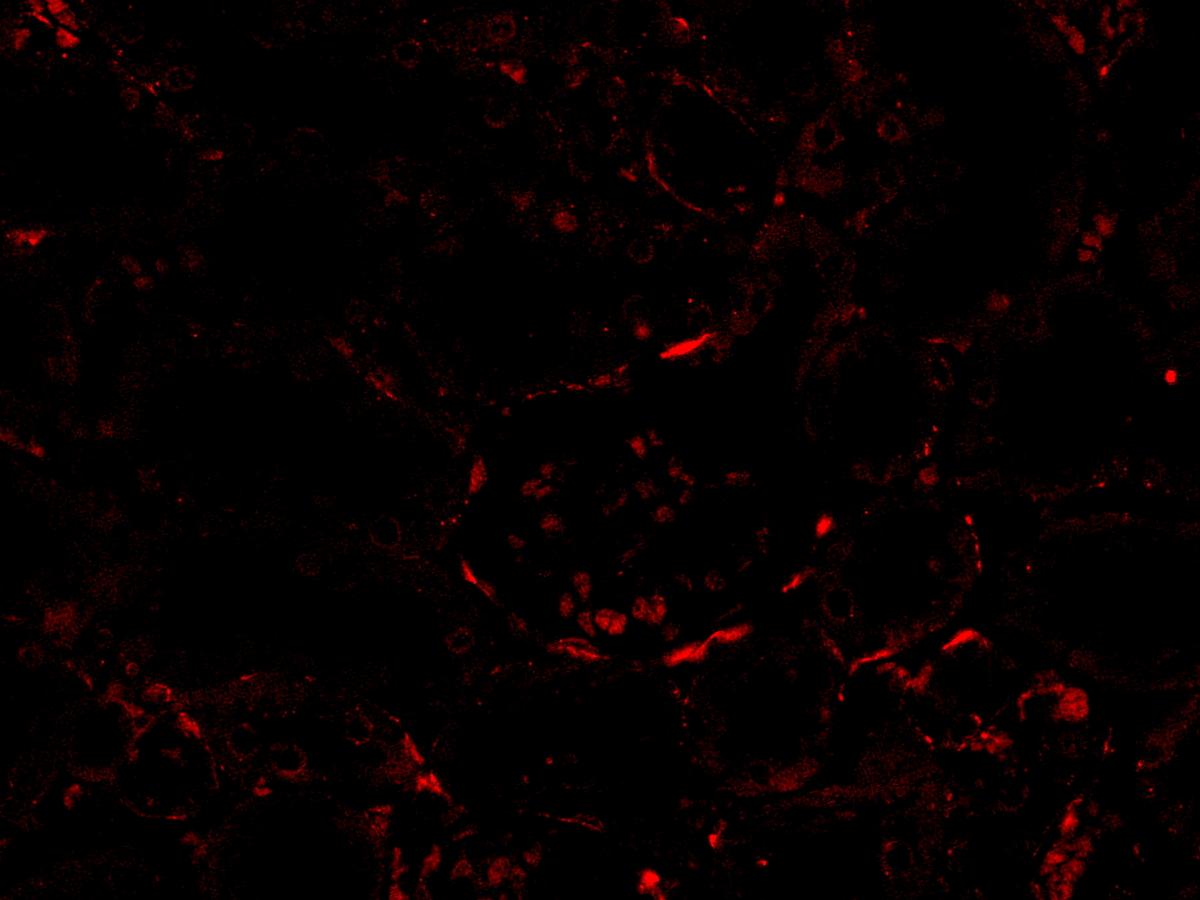

Supplement: Supplementary file 6 [file DataSheet2.zip › original images of figure 3/图3I-2-2(Synaptopodin).jpg]

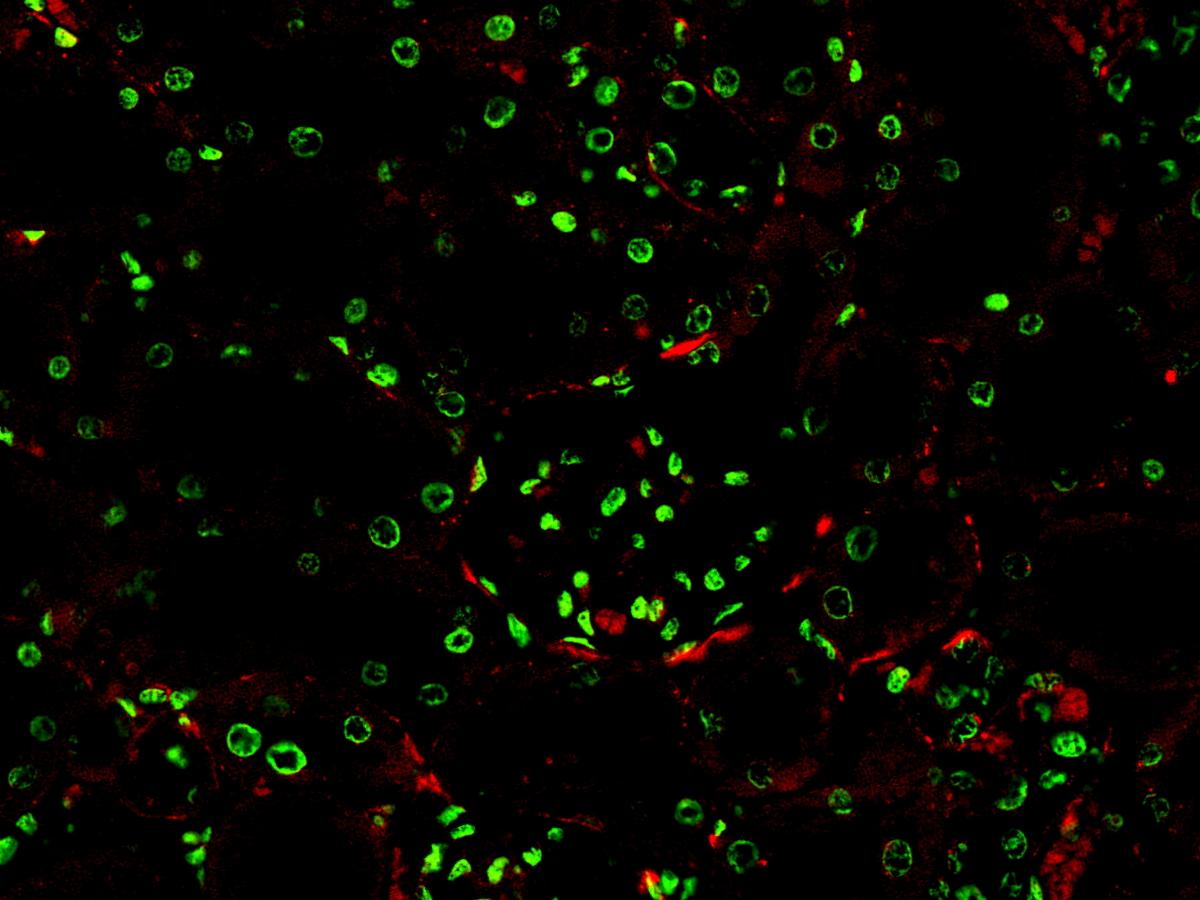

Supplement: Supplementary file 6 [file DataSheet2.zip › original images of figure 3/图3I-2-3(Merge).jpg]

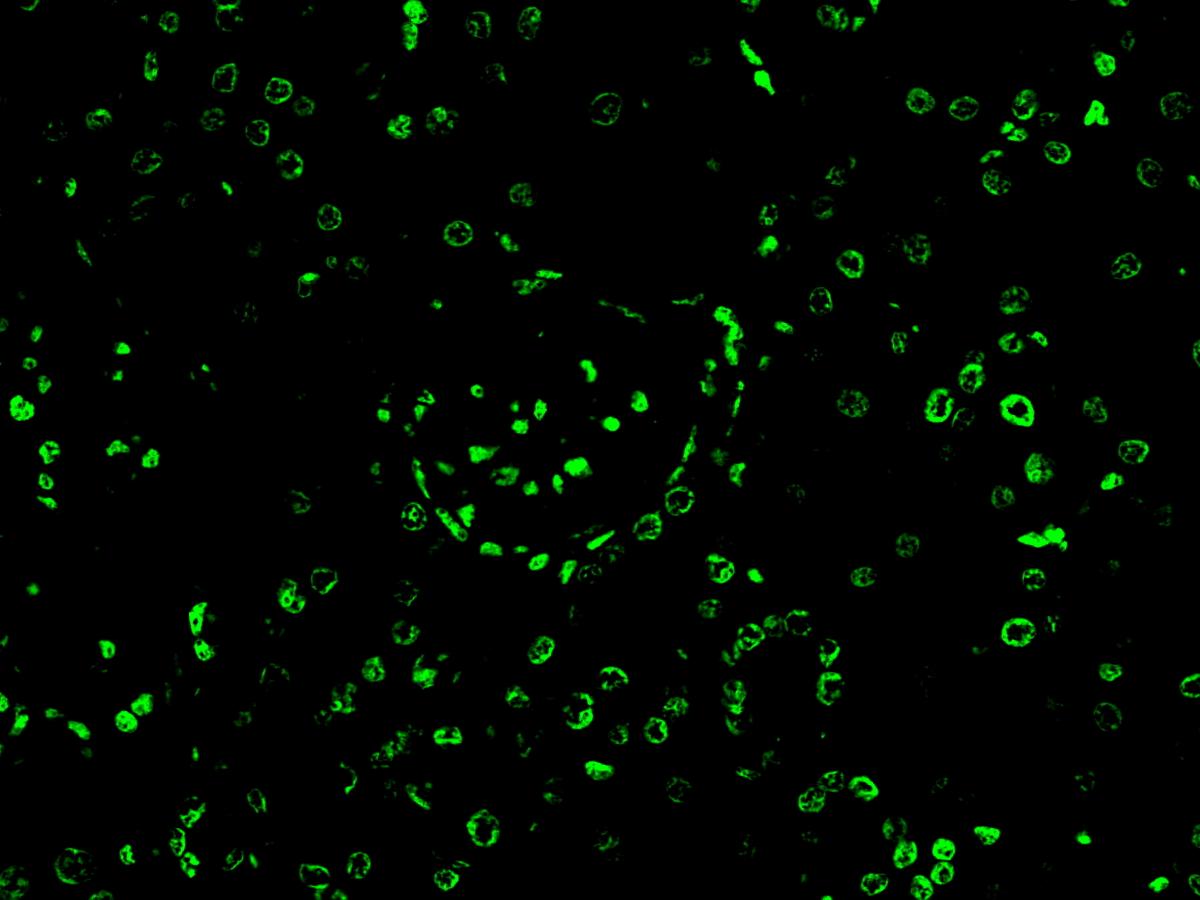

Supplement: Supplementary file 6 [file DataSheet2.zip › original images of figure 3/图3I-3-1(PARP1).jpg]

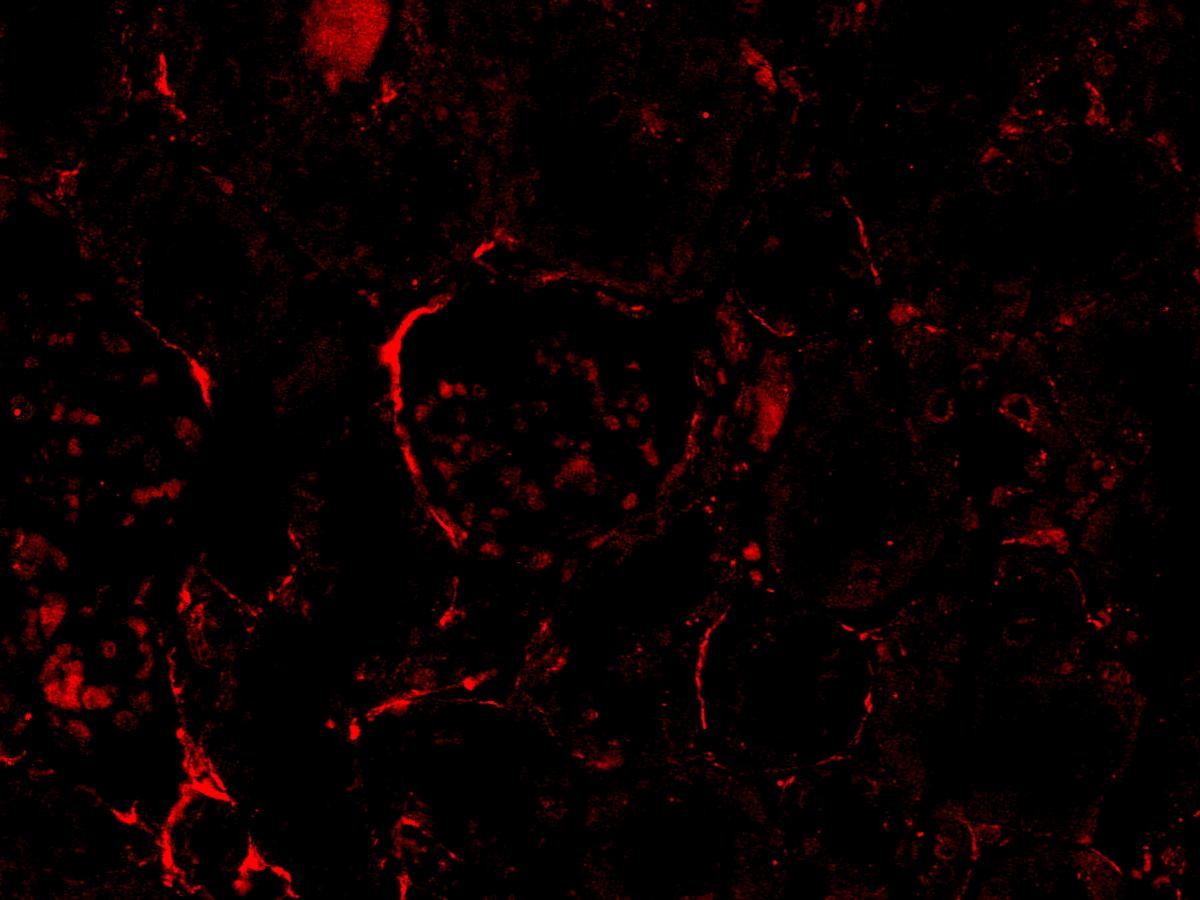

Supplement: Supplementary file 6 [file DataSheet2.zip › original images of figure 3/图3I-3-2(Synaptopodin).jpg]

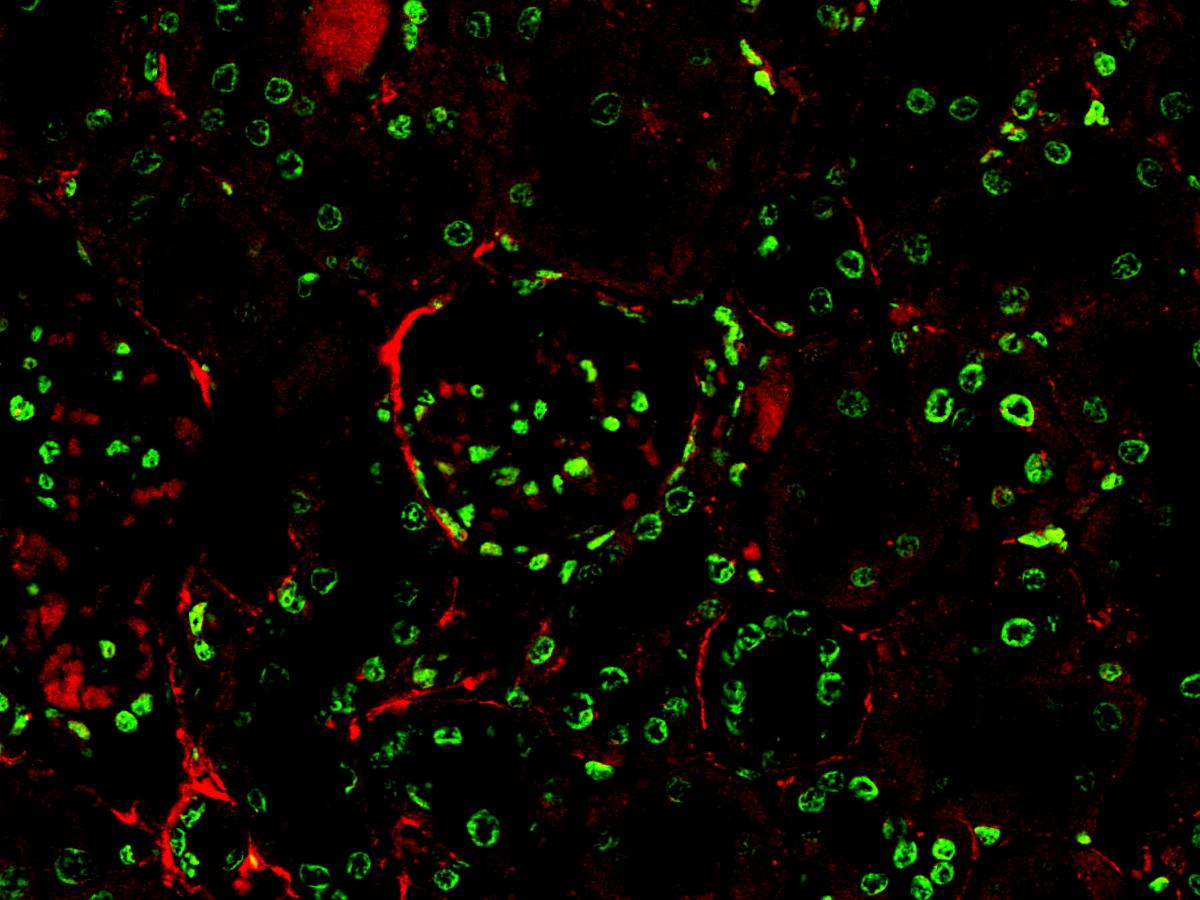

Supplement: Supplementary file 6 [file DataSheet2.zip › original images of figure 3/图3I-3-3(Merge).jpg]

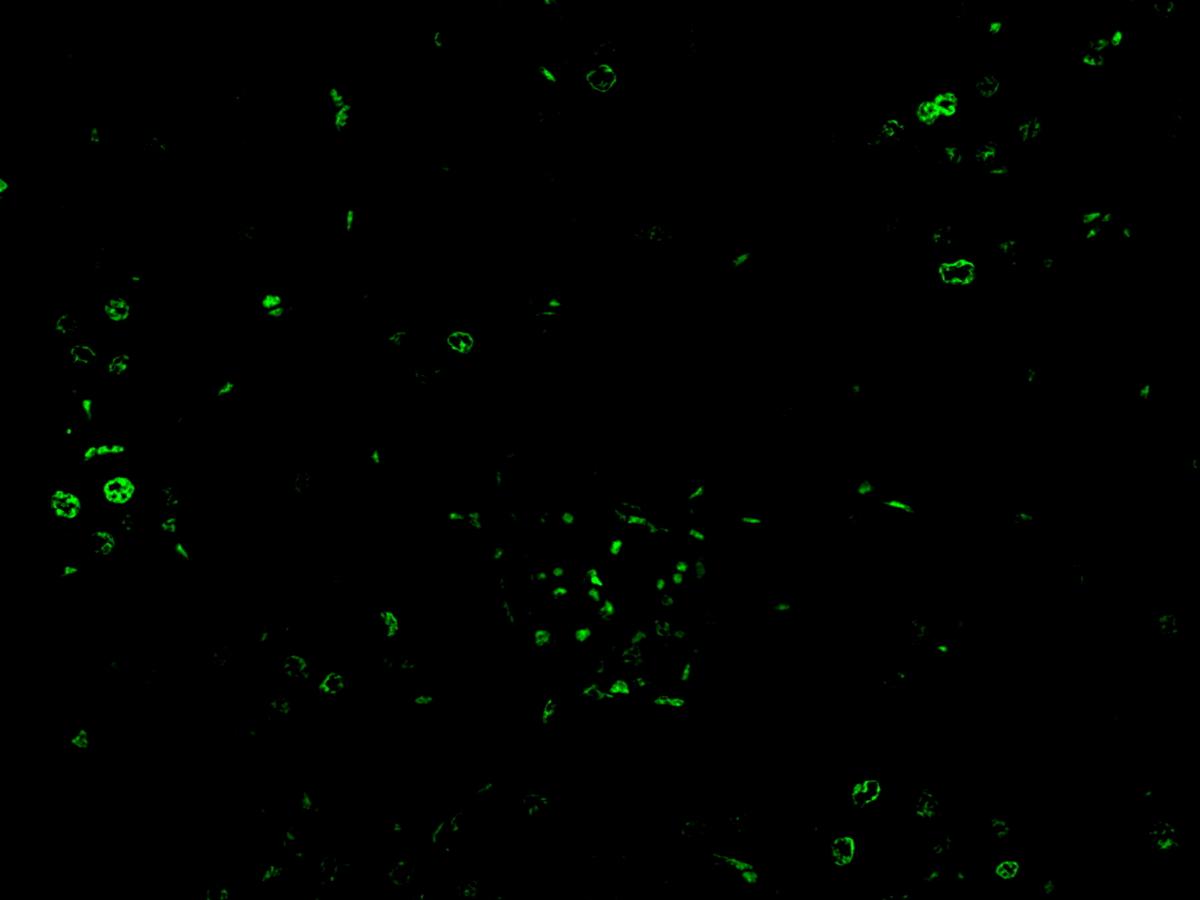

Supplement: Supplementary file 6 [file DataSheet2.zip › original images of figure 3/图3I-4-1(PARP1).jpg]

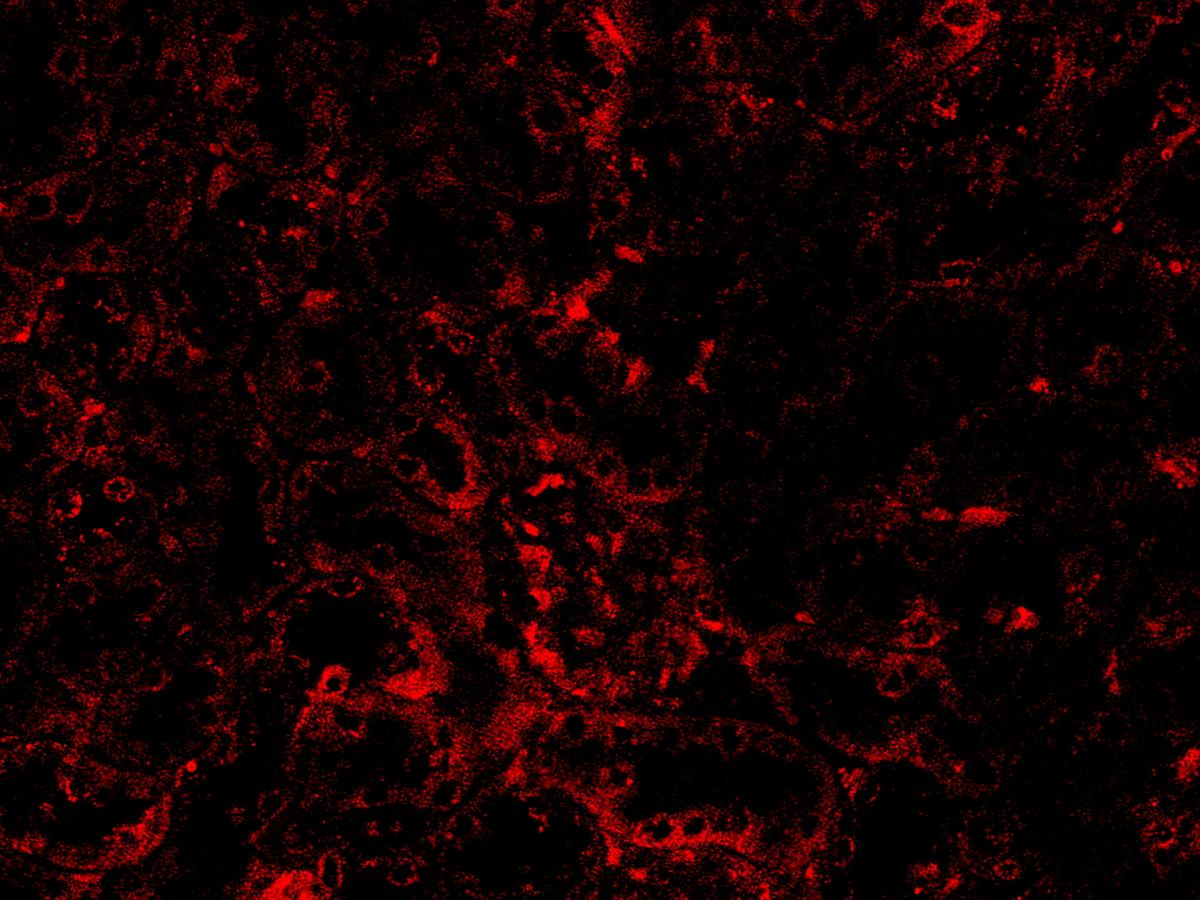

Supplement: Supplementary file 6 [file DataSheet2.zip › original images of figure 3/图3I-4-2(Synaptopodin).jpg]

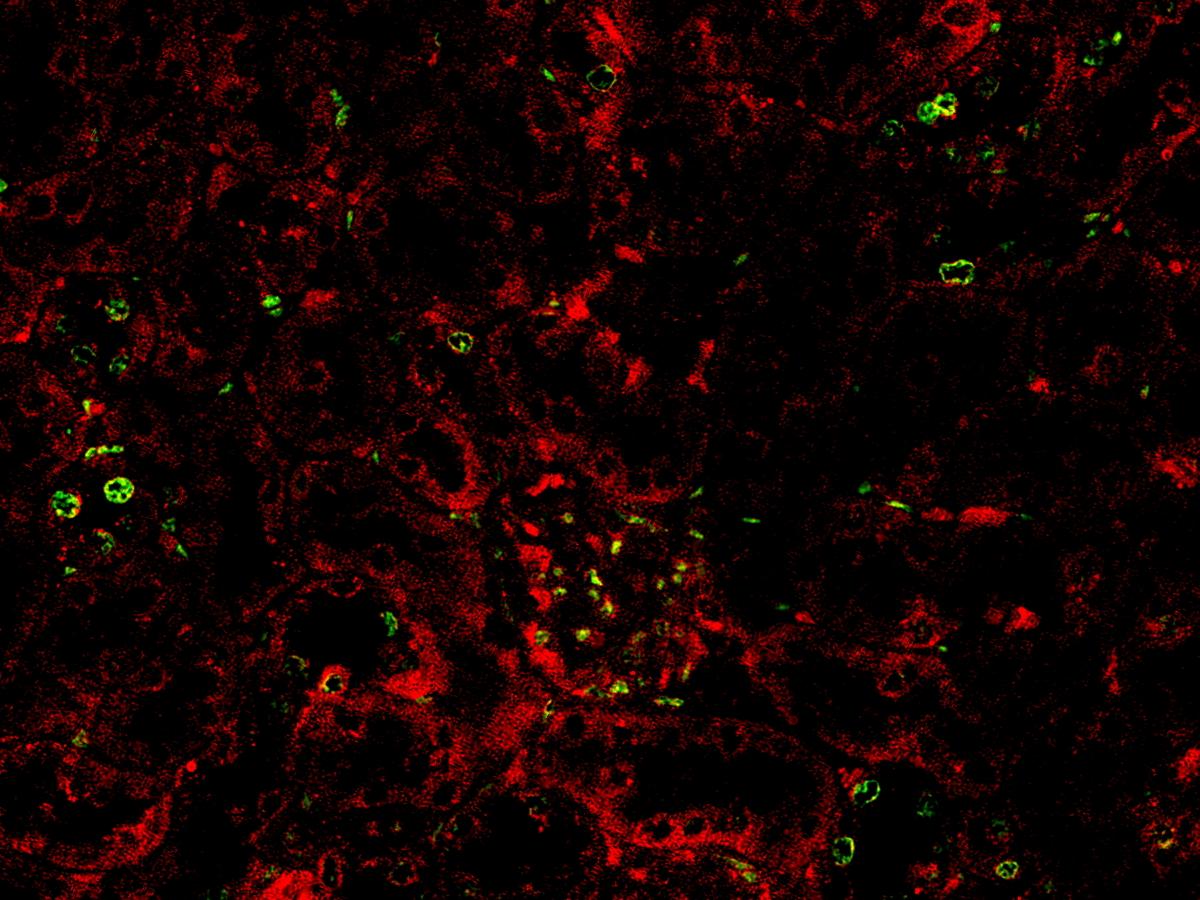

Supplement: Supplementary file 6 [file DataSheet2.zip › original images of figure 3/图3I-4-3(Merge).jpg]

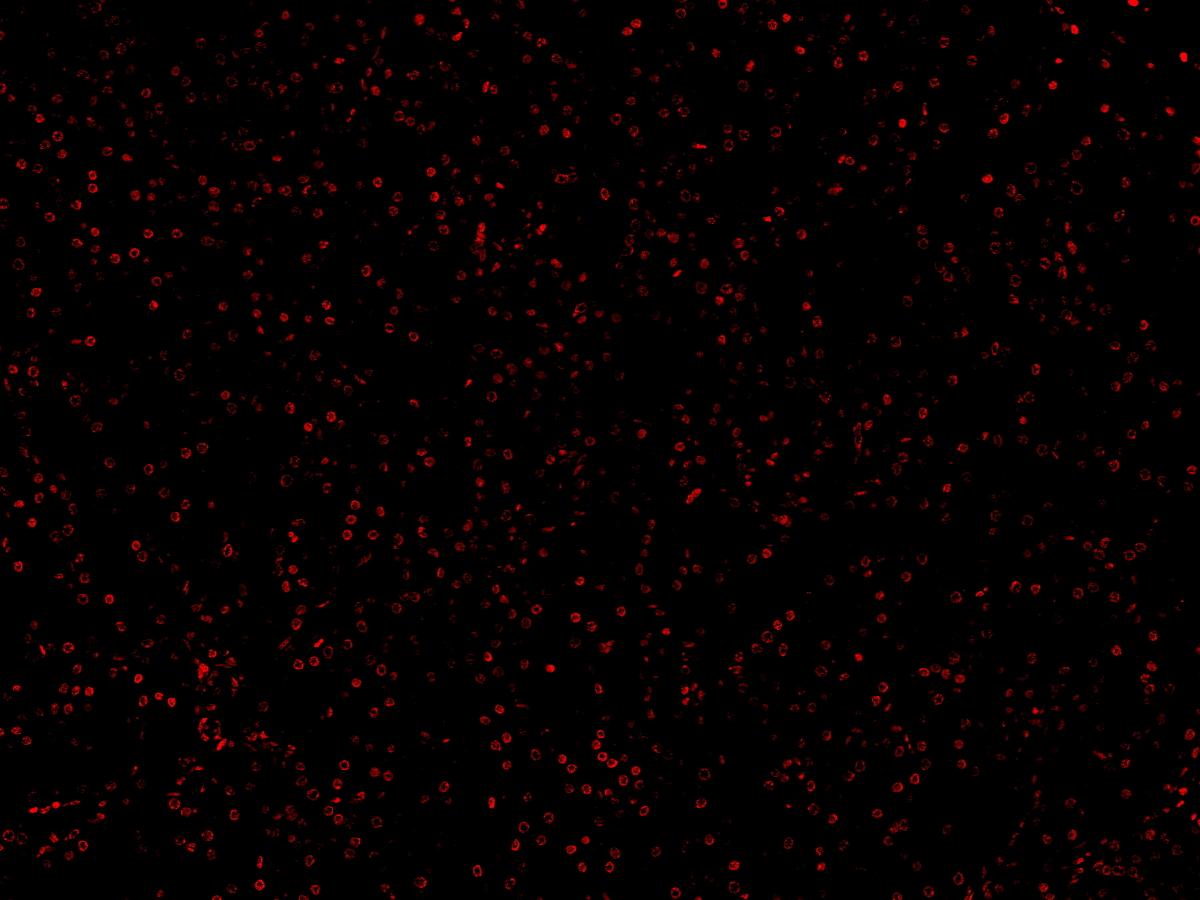

Supplement: Supplementary file 6 [file DataSheet2.zip › original images of figure 3/图3J-1-1(Tunel).jpg]

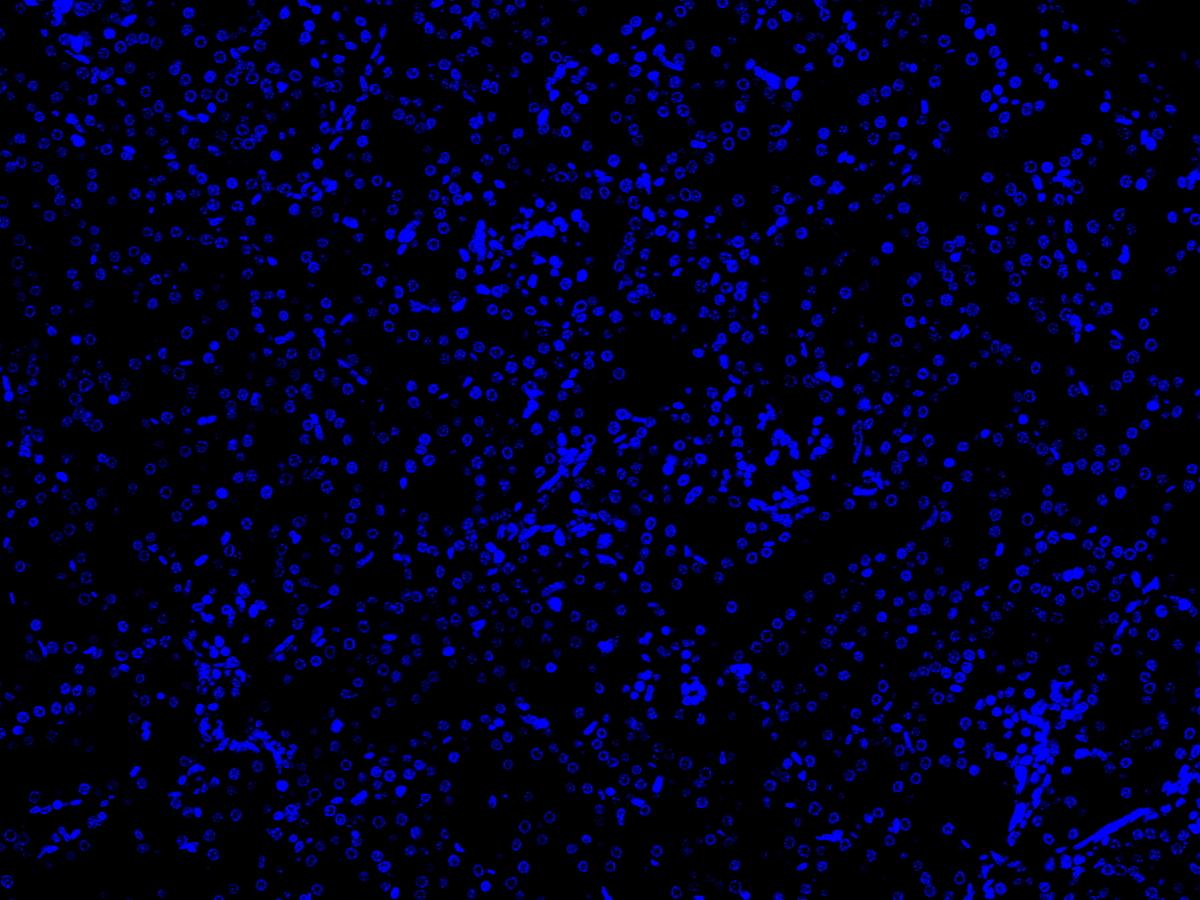

Supplement: Supplementary file 6 [file DataSheet2.zip › original images of figure 3/图3J-1-2(DAPI).jpg]

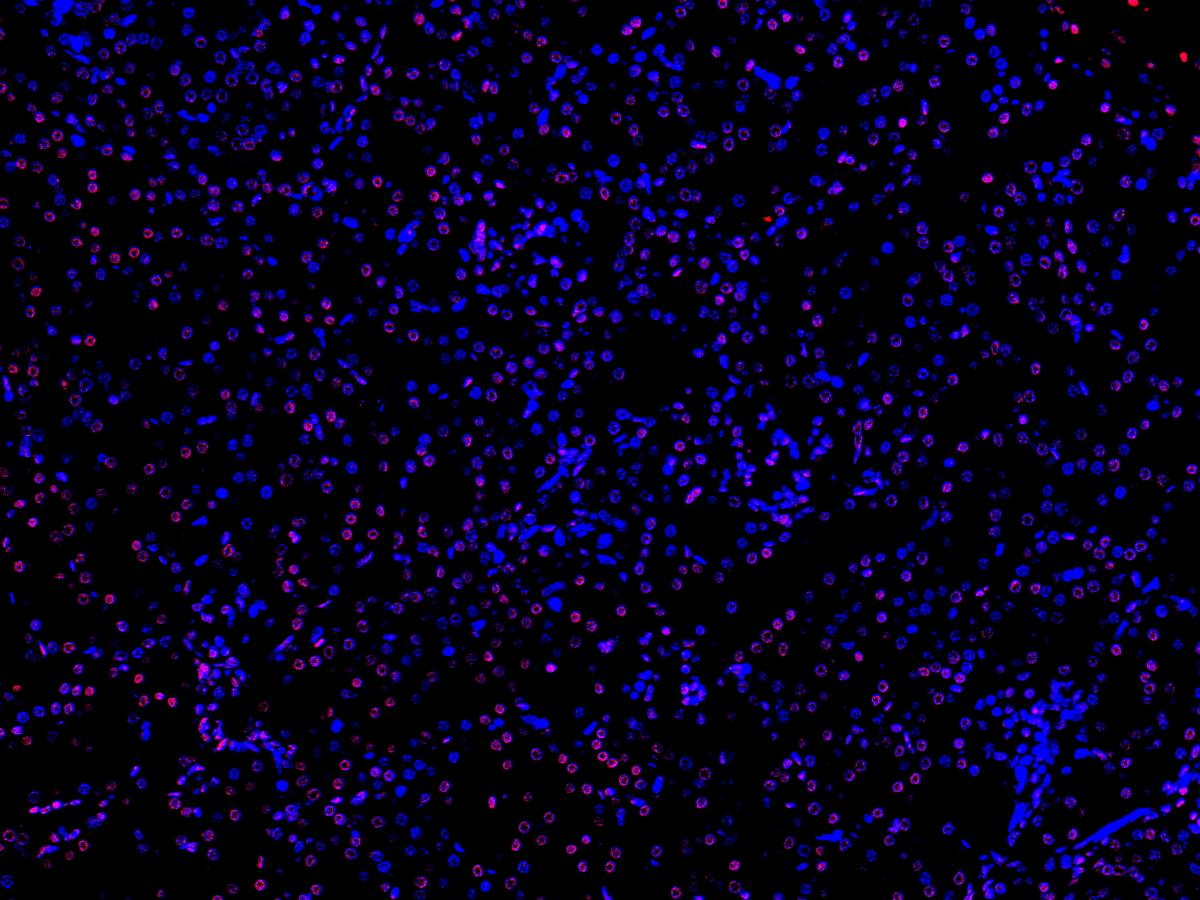

Supplement: Supplementary file 6 [file DataSheet2.zip › original images of figure 3/图3J-1-3(Merge).jpg]

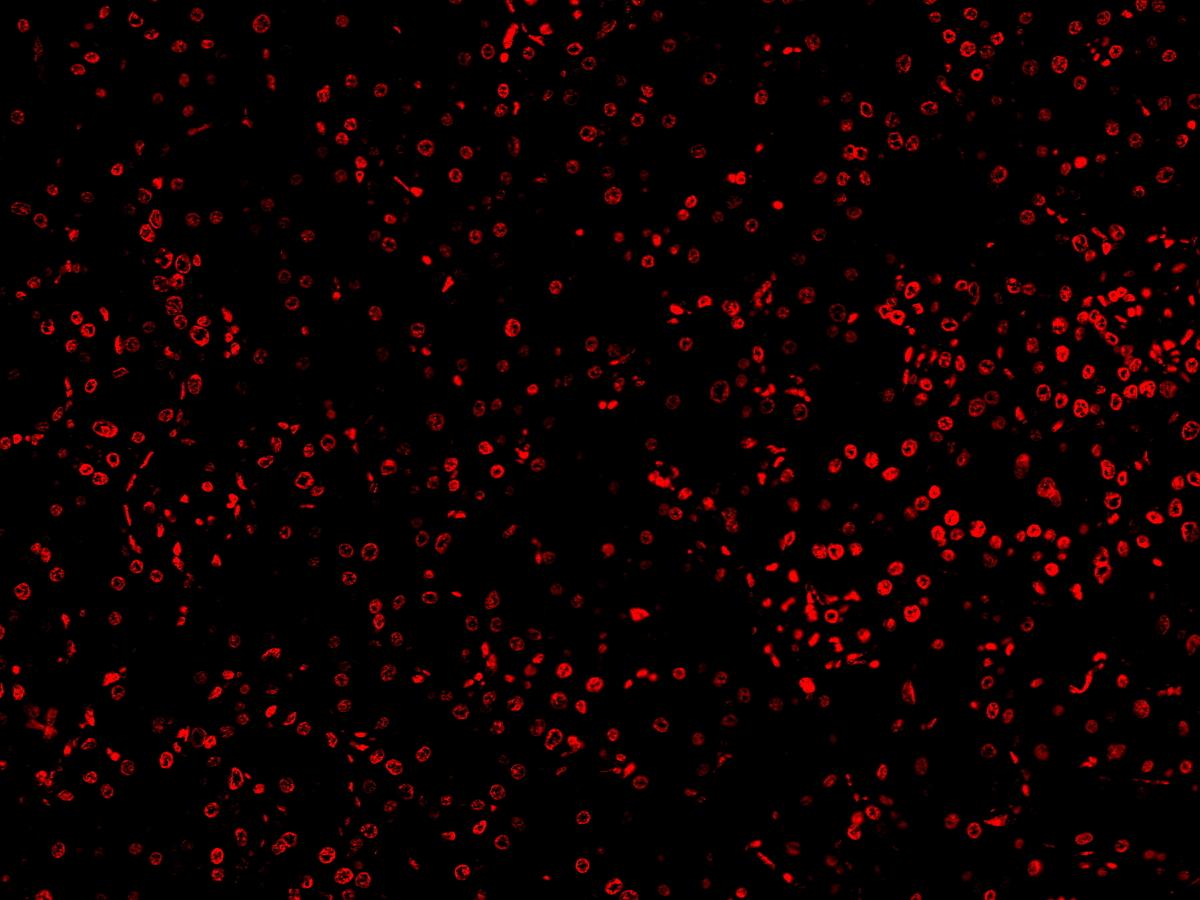

Supplement: Supplementary file 6 [file DataSheet2.zip › original images of figure 3/图3J-2-1(Tunel).jpg]

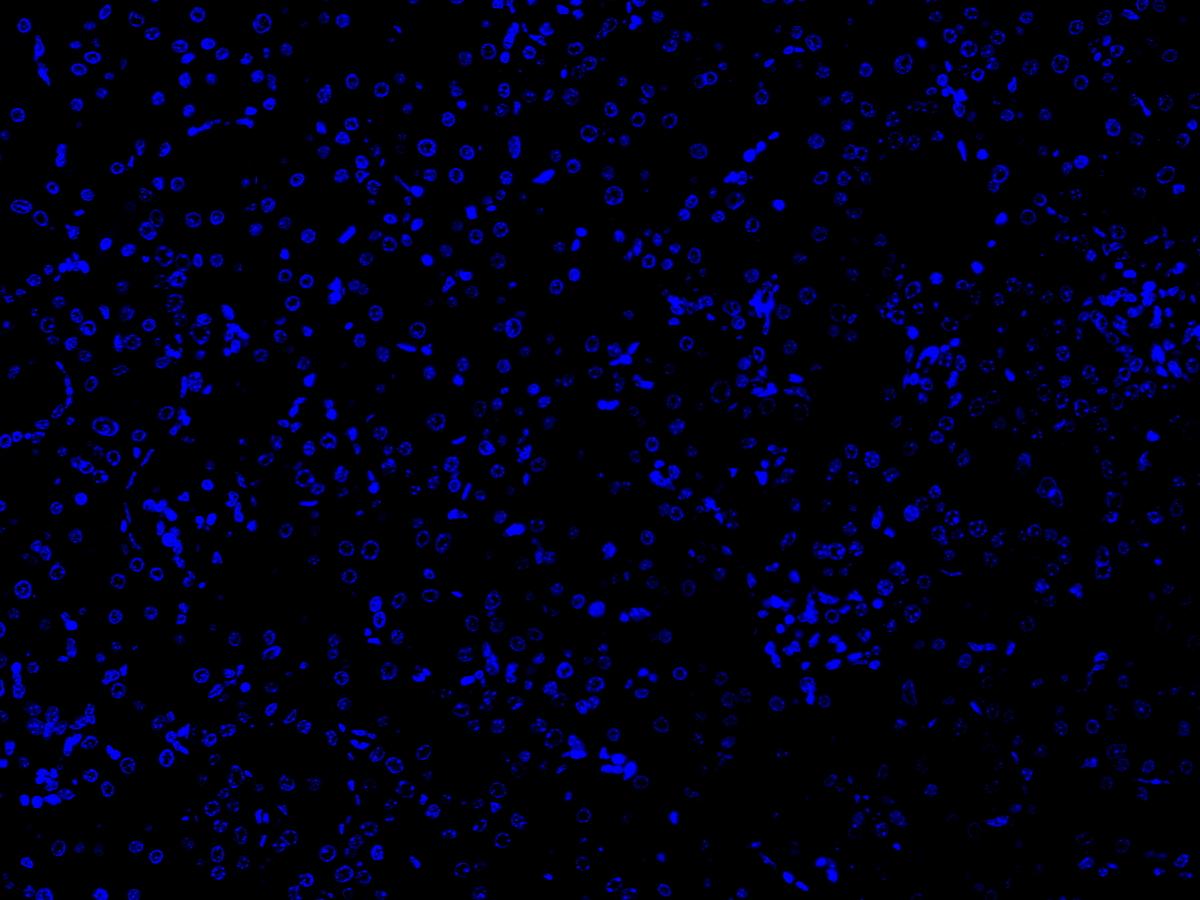

Supplement: Supplementary file 6 [file DataSheet2.zip › original images of figure 3/图3J-2-2(DAPI).jpg]

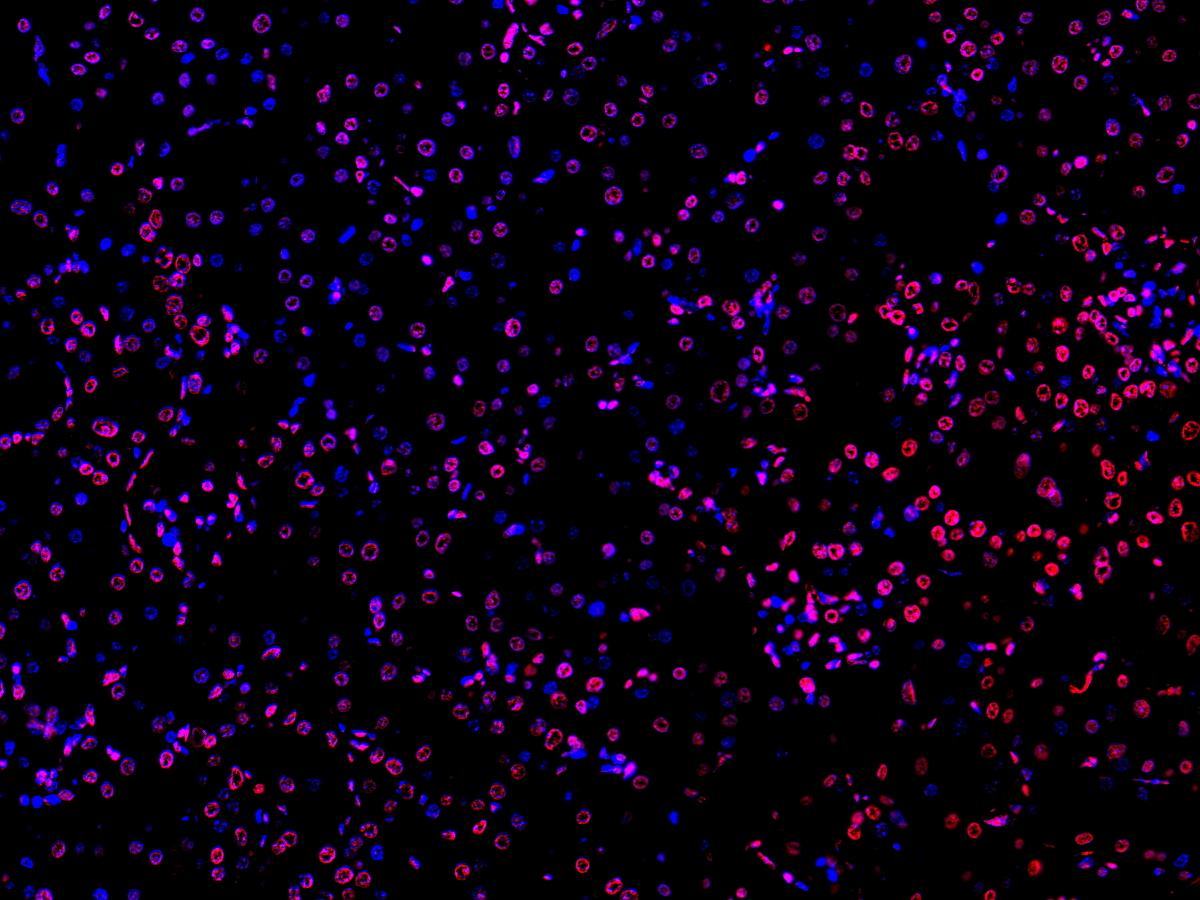

Supplement: Supplementary file 6 [file DataSheet2.zip › original images of figure 3/图3J-2-3(Merge).jpg]

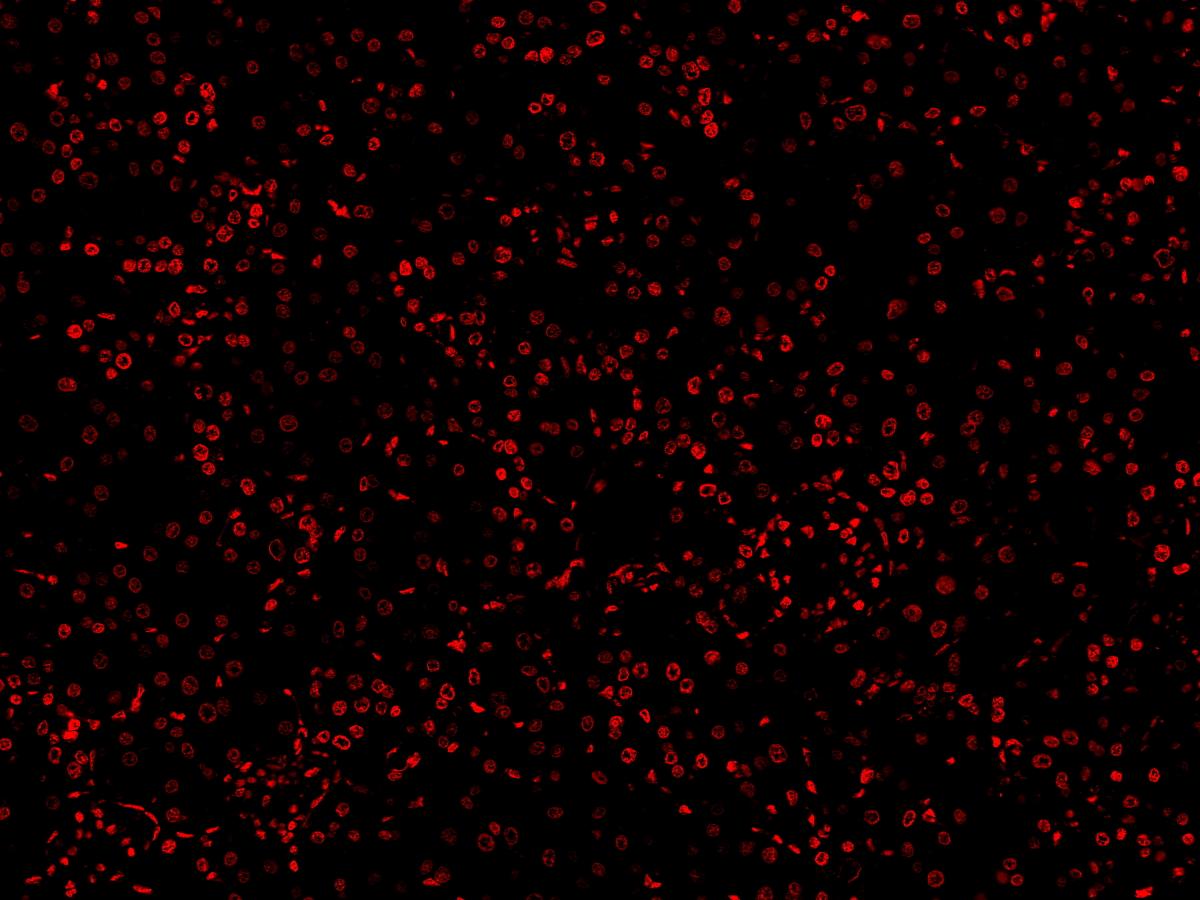

Supplement: Supplementary file 6 [file DataSheet2.zip › original images of figure 3/图3J-3-1(Tunel).jpg]

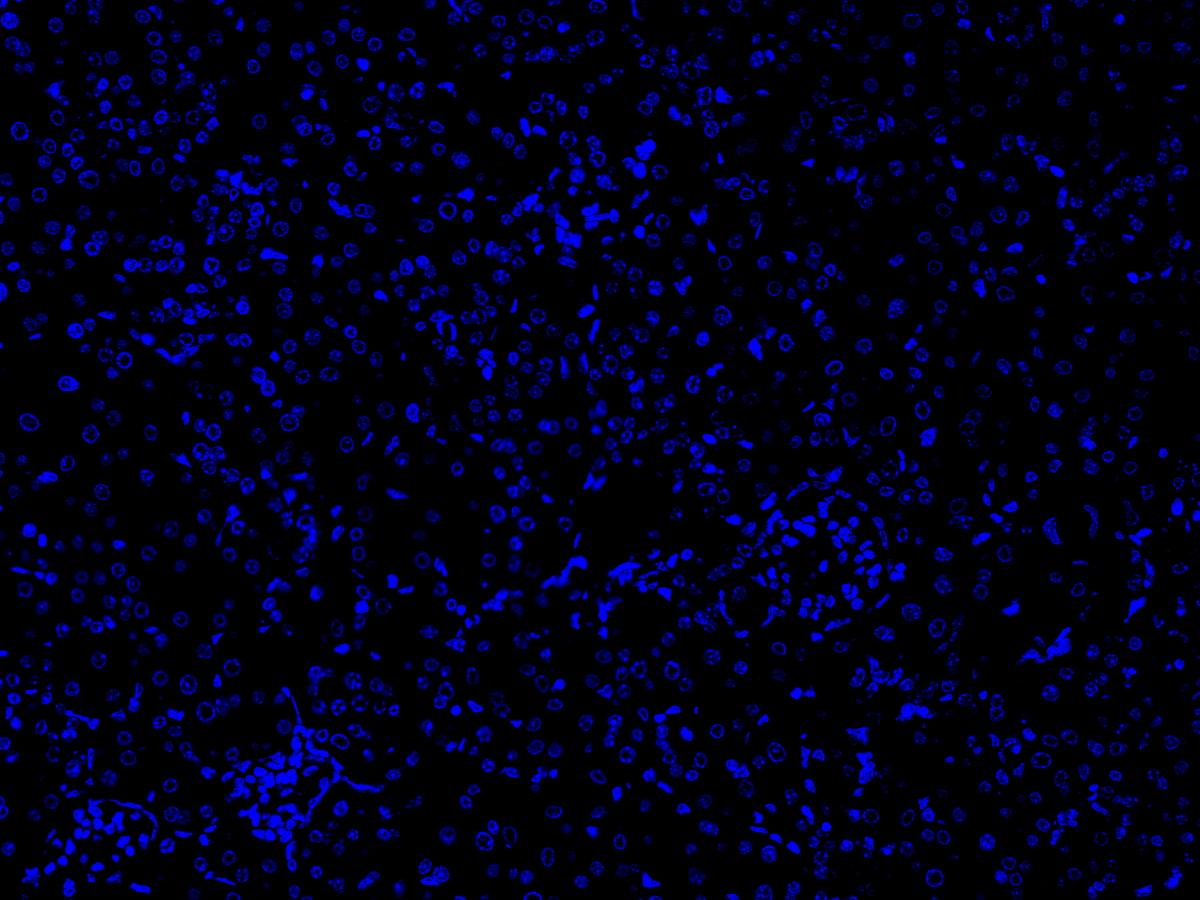

Supplement: Supplementary file 6 [file DataSheet2.zip › original images of figure 3/图3J-3-2(DAPI).jpg]

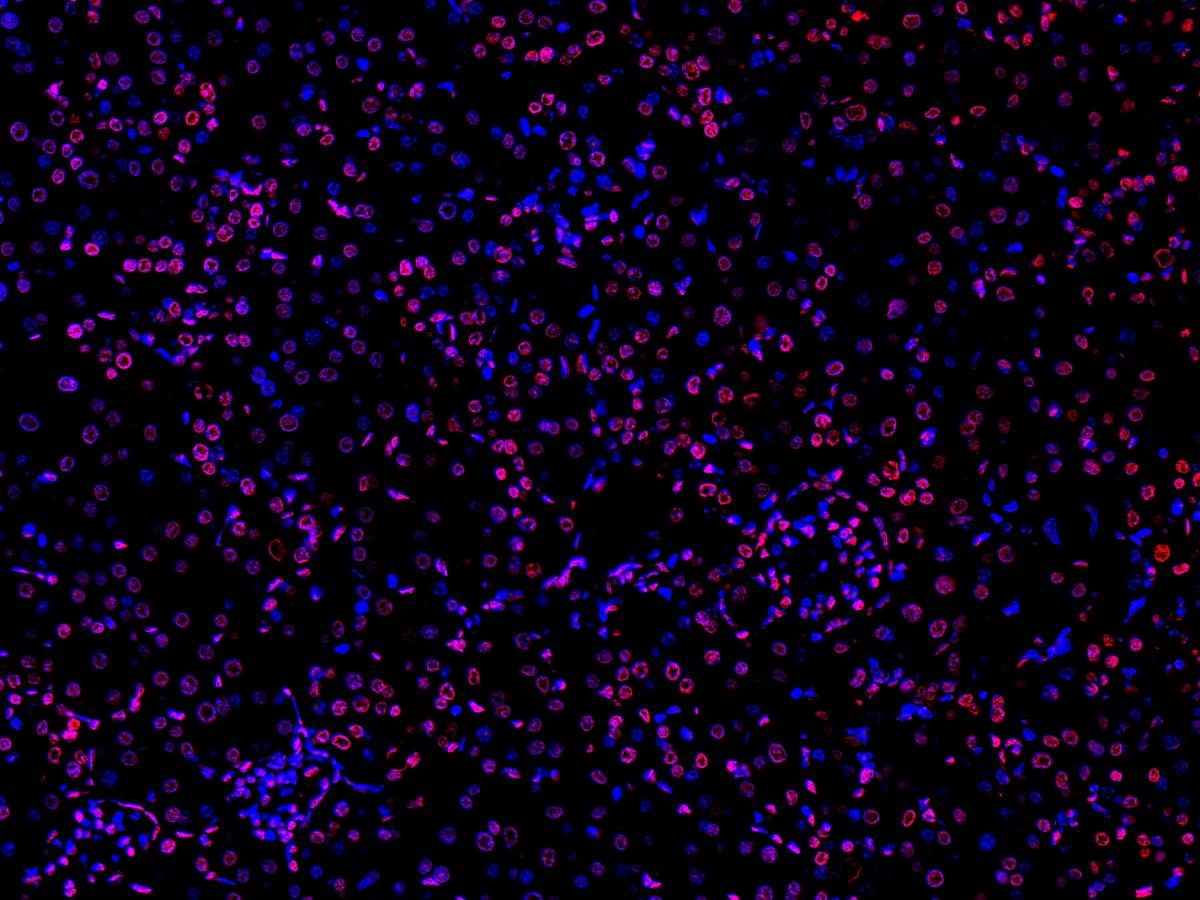

Supplement: Supplementary file 6 [file DataSheet2.zip › original images of figure 3/图3J-3-3(Merge).jpg]

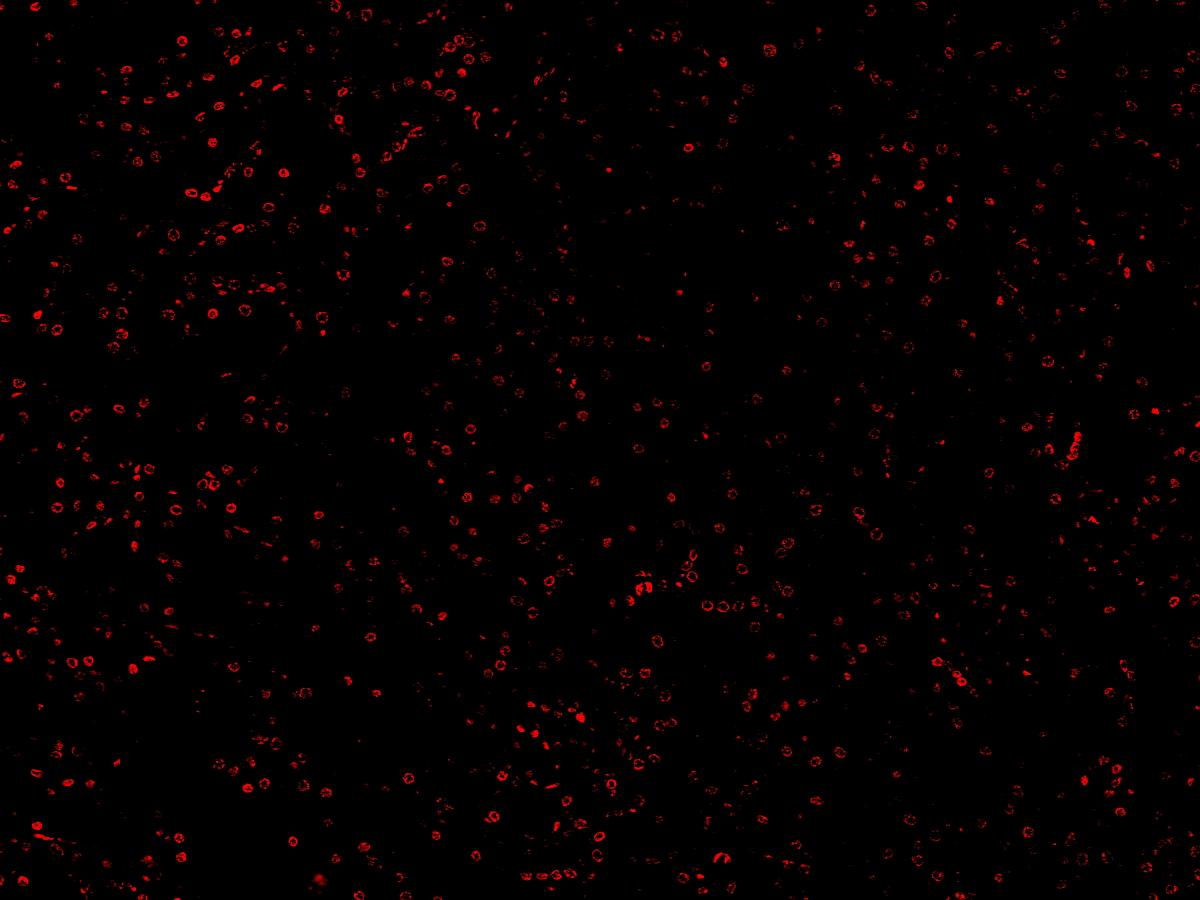

Supplement: Supplementary file 6 [file DataSheet2.zip › original images of figure 3/图3J-4-1(Tunel).jpg]

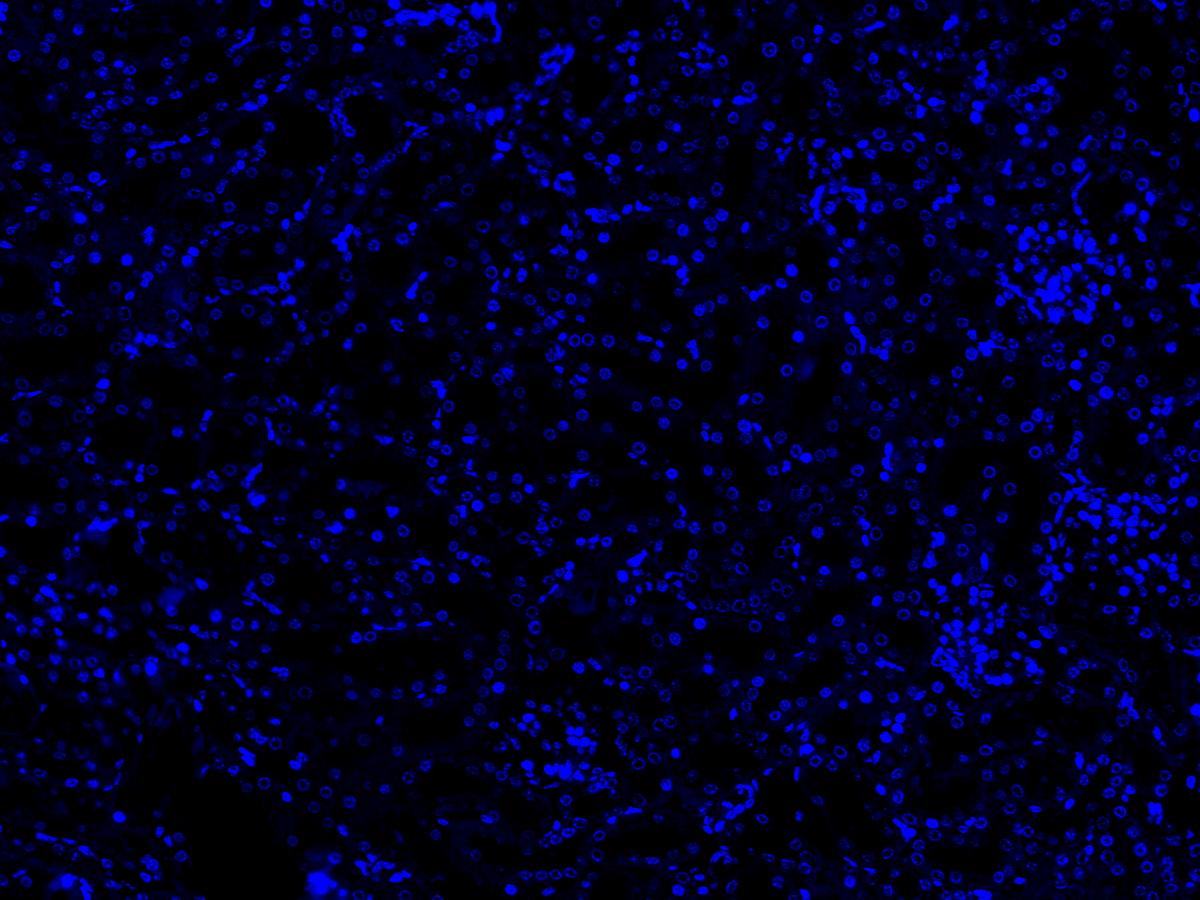

Supplement: Supplementary file 6 [file DataSheet2.zip › original images of figure 3/图3J-4-2(DAPI).jpg]

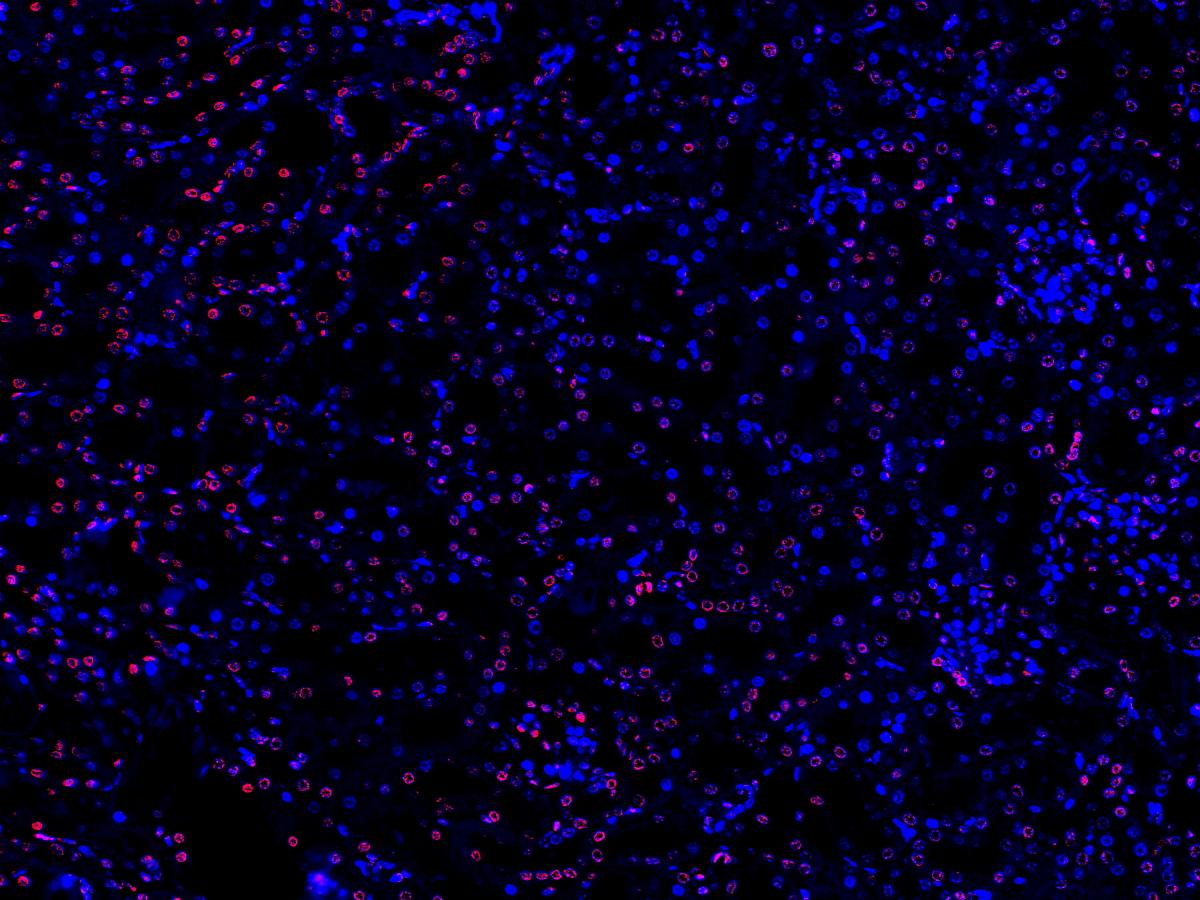

Supplement: Supplementary file 6 [file DataSheet2.zip › original images of figure 3/图3J-4-3(Merge).jpg]

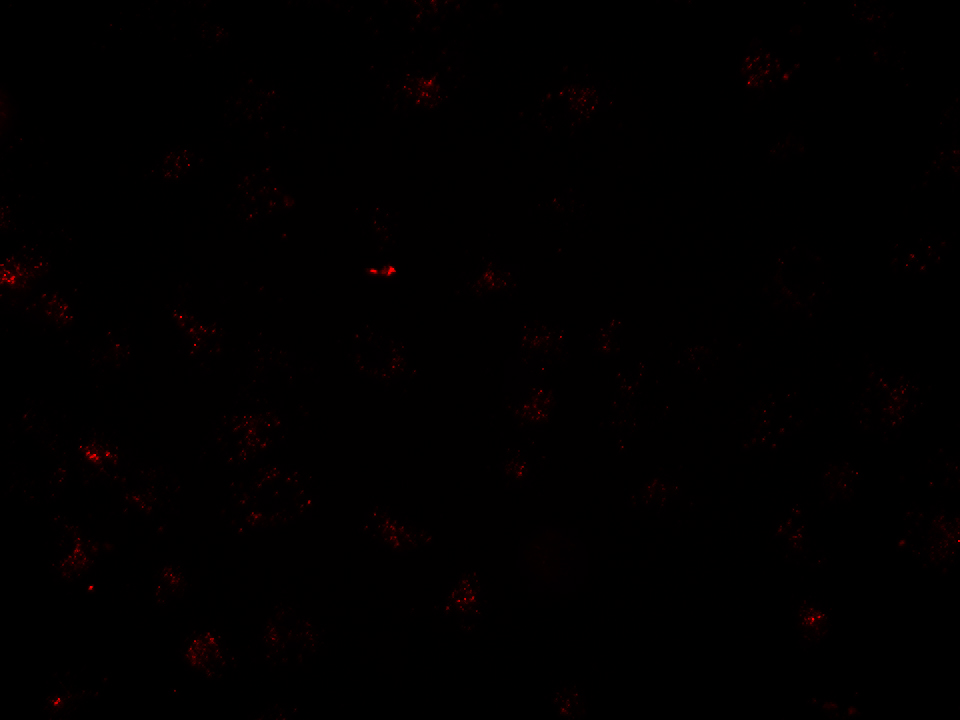

Supplement: Supplementary file 7 [file DataSheet5.zip › original images of figure 6/图6A-1-1.jpg]

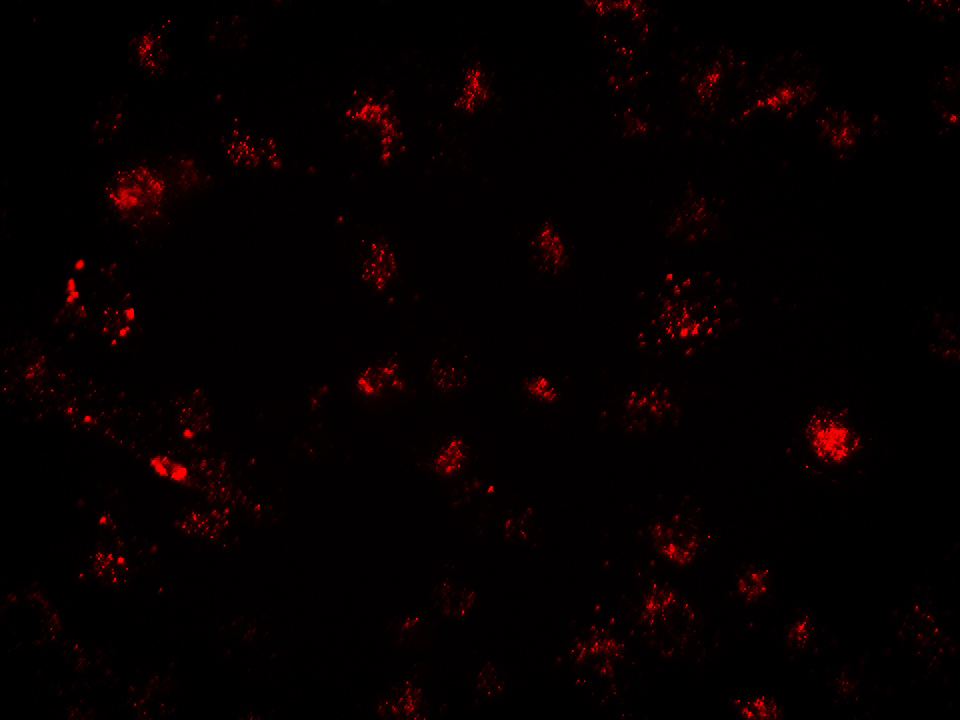

Supplement: Supplementary file 7 [file DataSheet5.zip › original images of figure 6/图6A-1-2.jpg]

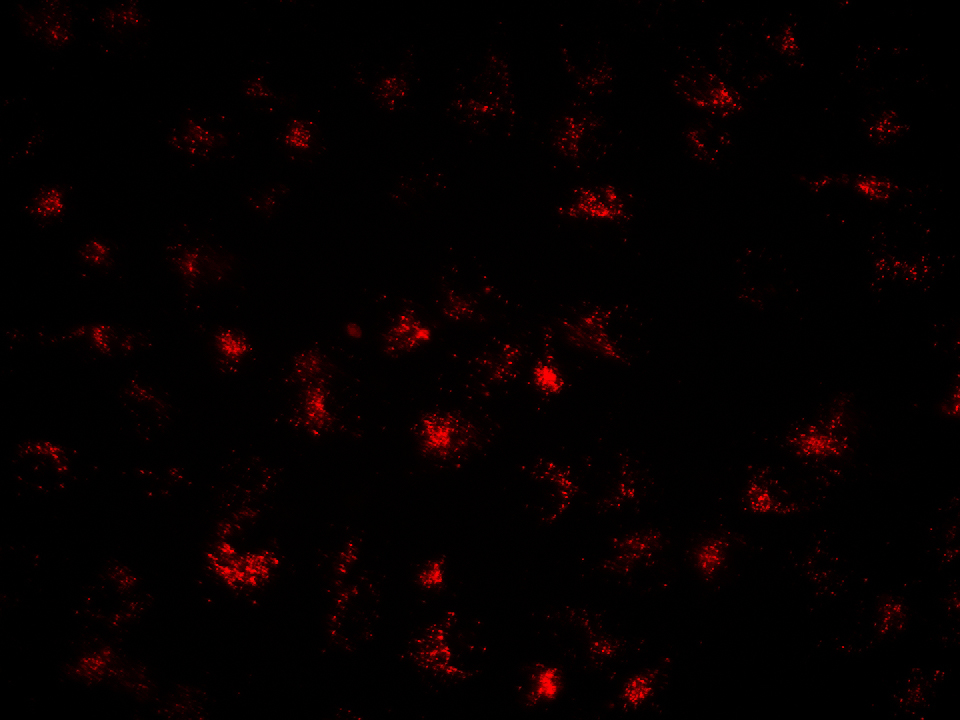

Supplement: Supplementary file 7 [file DataSheet5.zip › original images of figure 6/图6A-1-3.jpg]

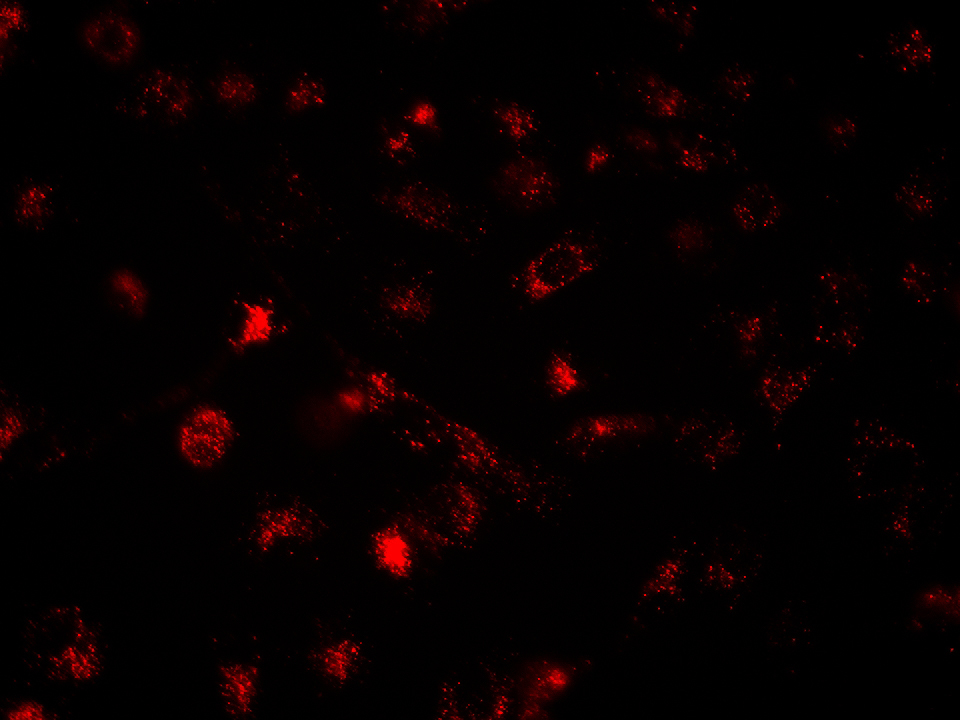

Supplement: Supplementary file 7 [file DataSheet5.zip › original images of figure 6/图6A-1-4.jpg]

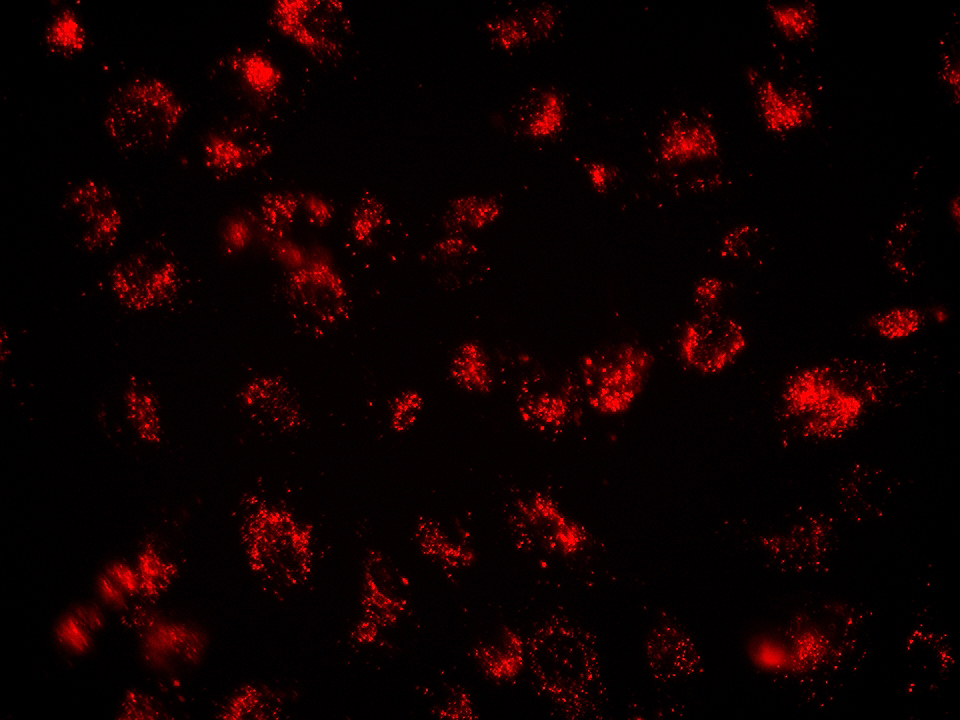

Supplement: Supplementary file 7 [file DataSheet5.zip › original images of figure 6/图6A-1-5.jpg]

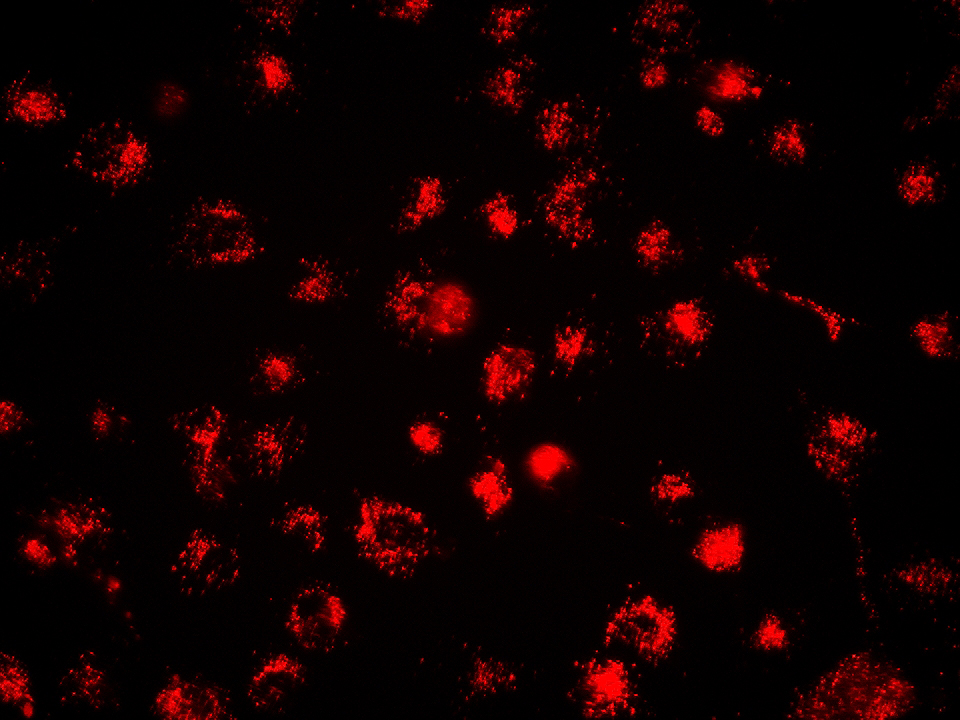

Supplement: Supplementary file 7 [file DataSheet5.zip › original images of figure 6/图6A-1-6.jpg]

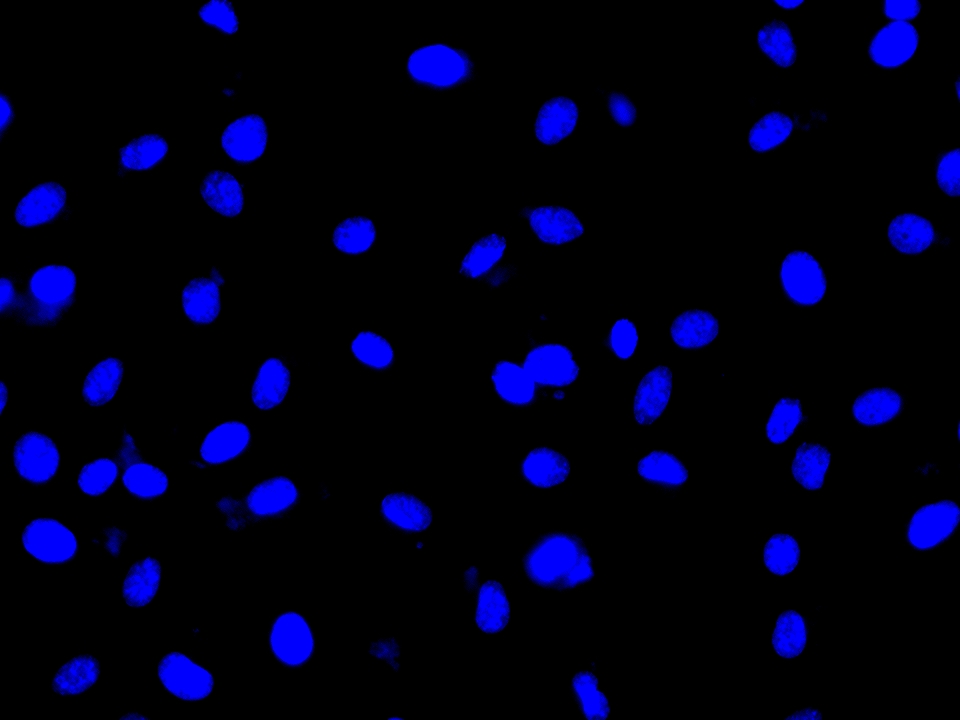

Supplement: Supplementary file 7 [file DataSheet5.zip › original images of figure 6/图6A-2-1.jpg]

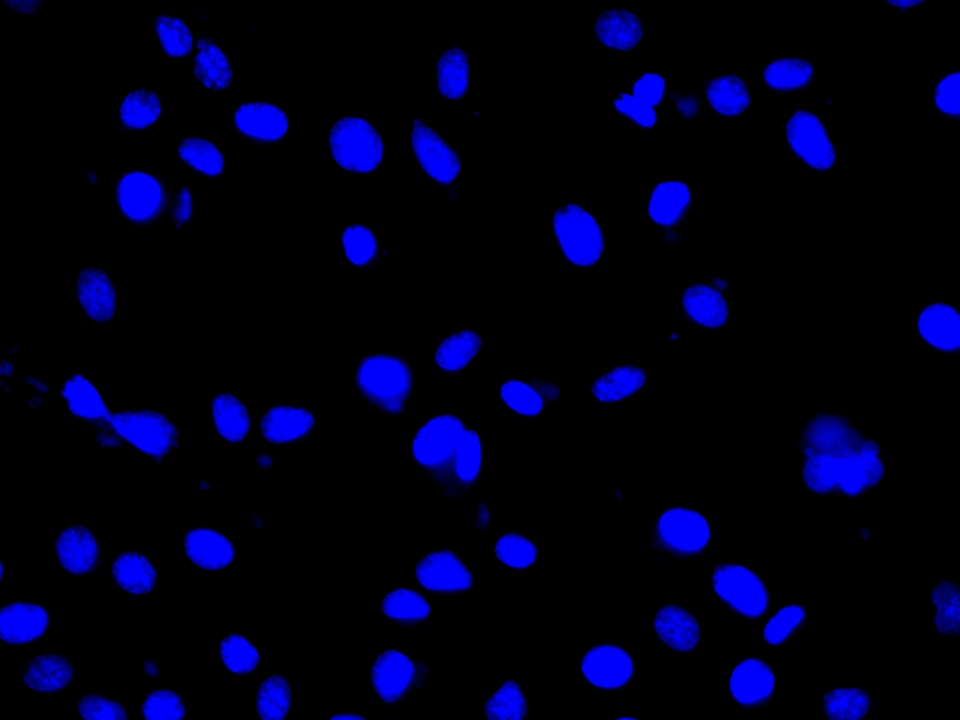

Supplement: Supplementary file 7 [file DataSheet5.zip › original images of figure 6/图6A-2-2.jpg]

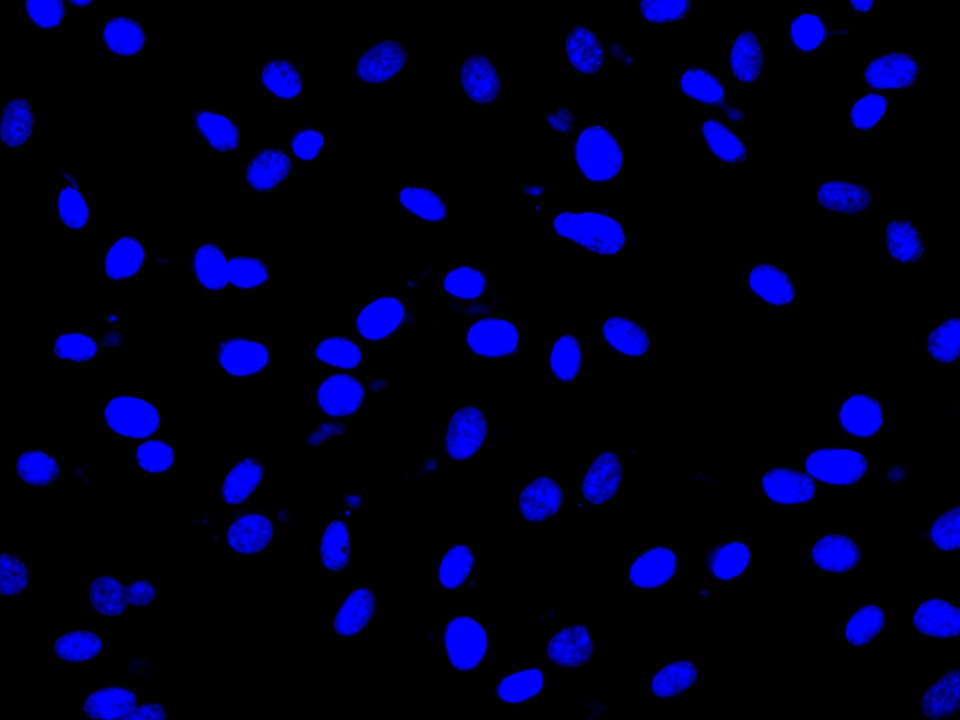

Supplement: Supplementary file 7 [file DataSheet5.zip › original images of figure 6/图6A-2-3.jpg]

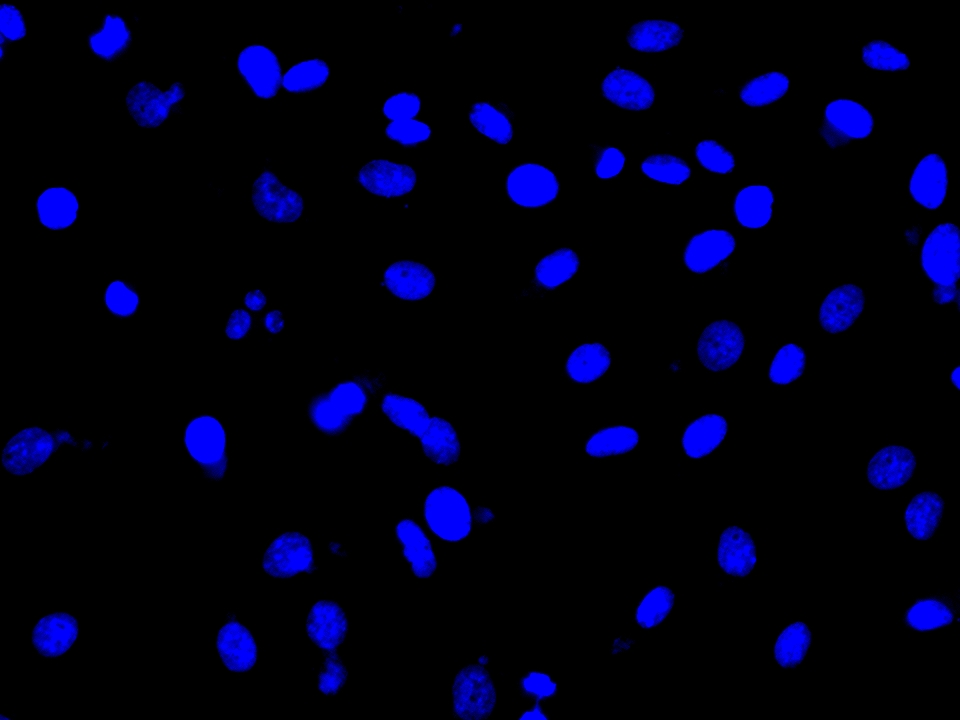

Supplement: Supplementary file 7 [file DataSheet5.zip › original images of figure 6/图6A-2-4.jpg]

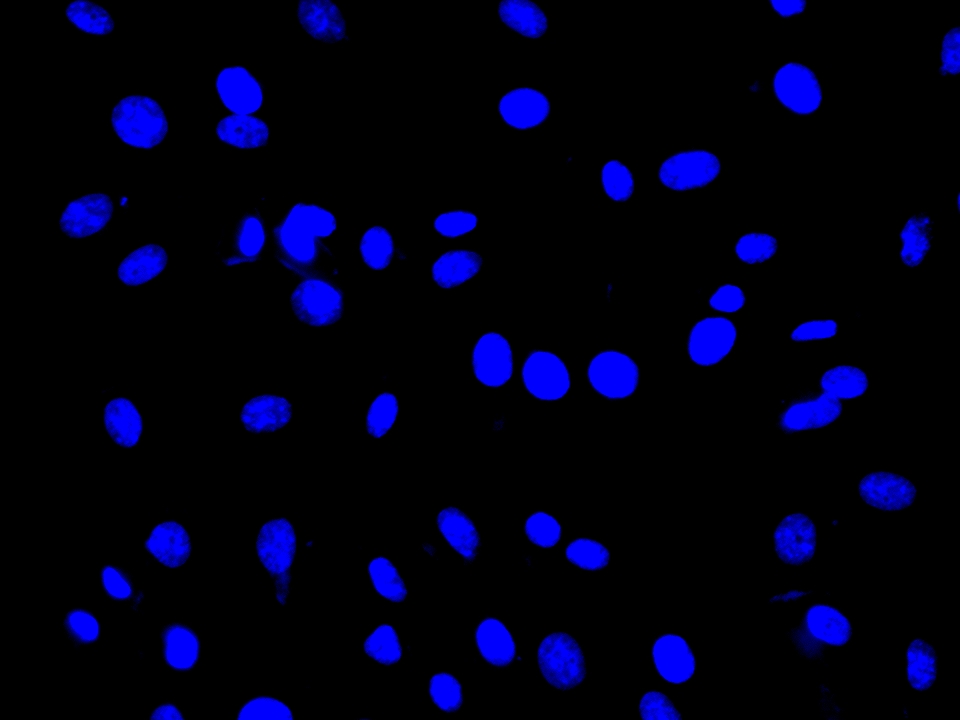

Supplement: Supplementary file 7 [file DataSheet5.zip › original images of figure 6/图6A-2-5.jpg]

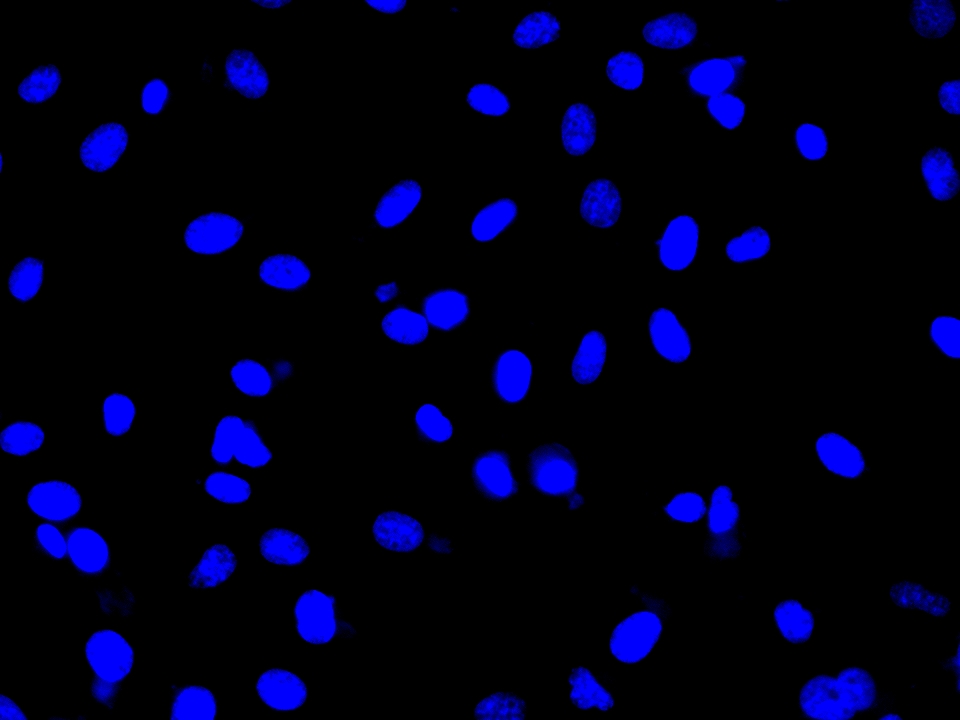

Supplement: Supplementary file 7 [file DataSheet5.zip › original images of figure 6/图6A-2-6.jpg]

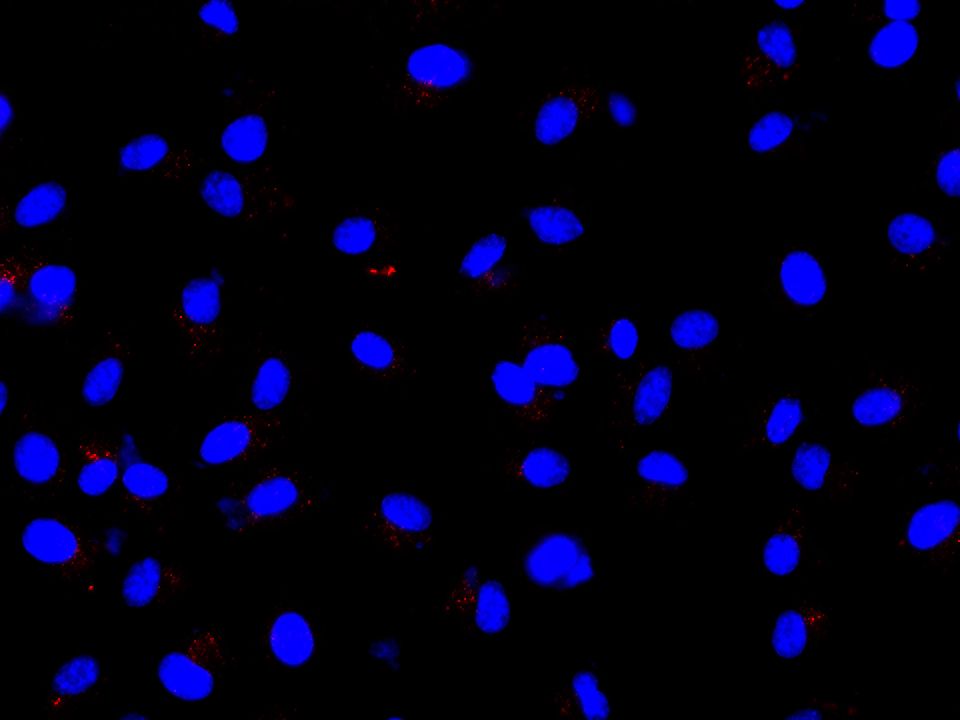

Supplement: Supplementary file 7 [file DataSheet5.zip › original images of figure 6/图6A-3-1.jpg]

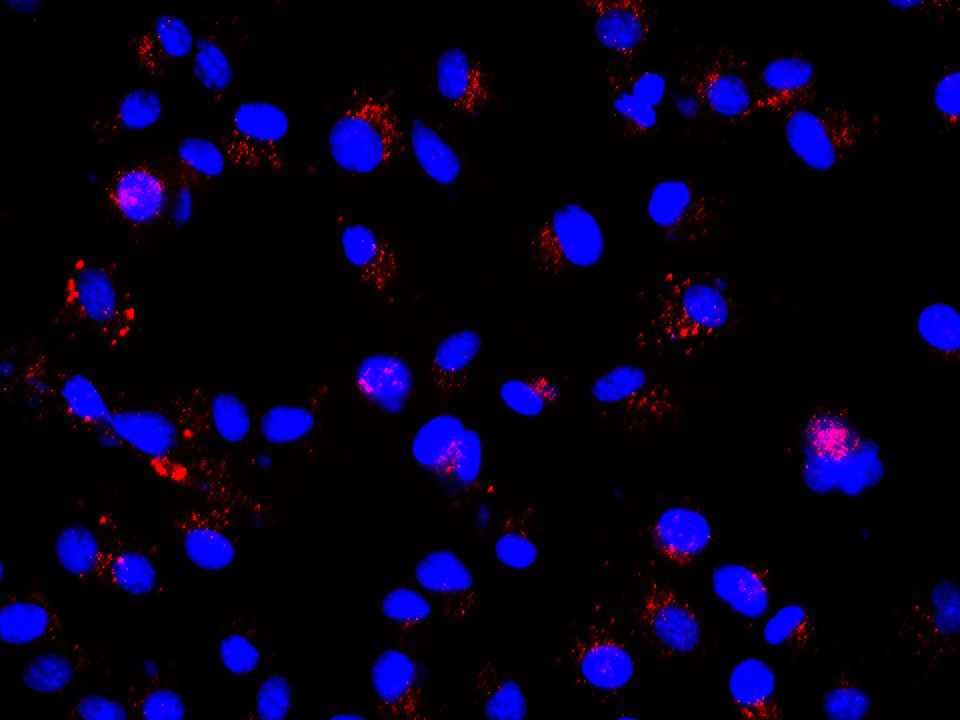

Supplement: Supplementary file 7 [file DataSheet5.zip › original images of figure 6/图6A-3-2.jpg]

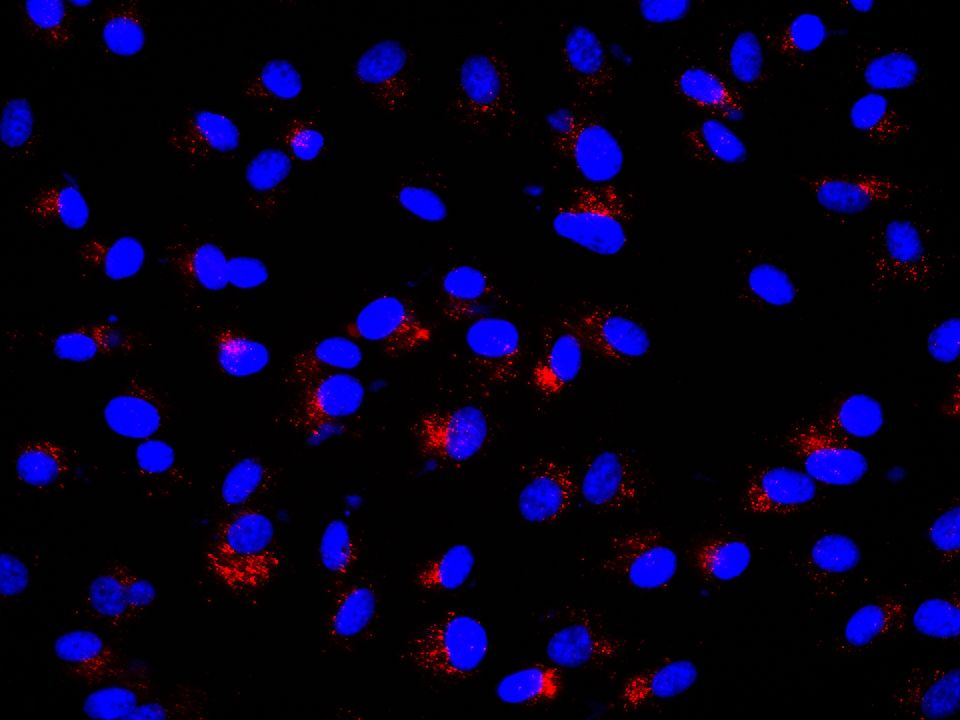

Supplement: Supplementary file 7 [file DataSheet5.zip › original images of figure 6/图6A-3-3.jpg]

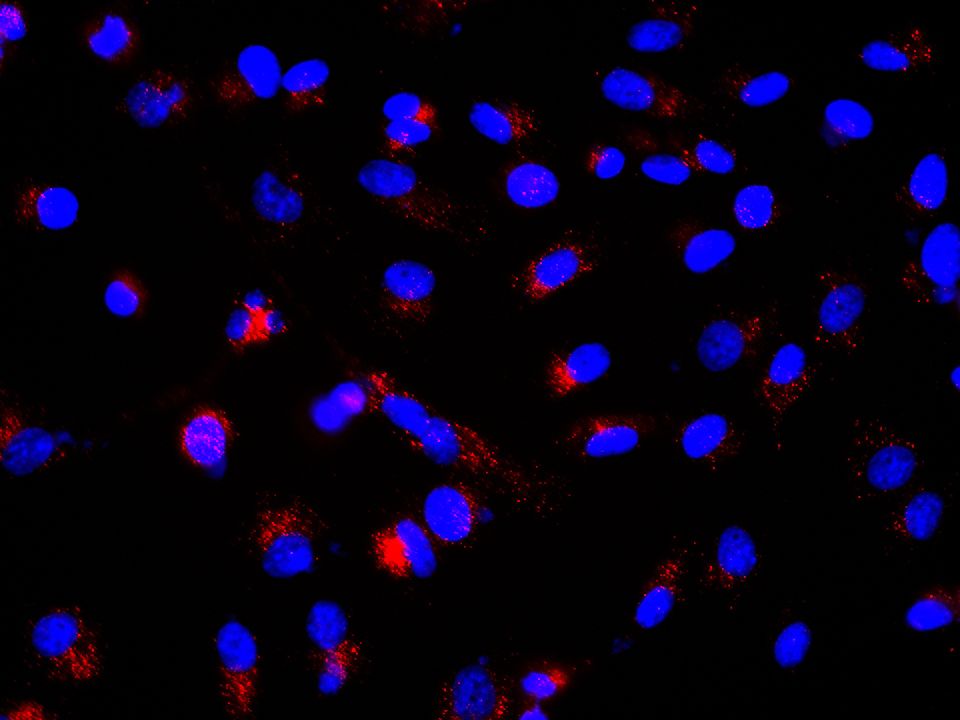

Supplement: Supplementary file 7 [file DataSheet5.zip › original images of figure 6/图6A-3-4.jpg]
