# Supplementary material for: Bioengineered exosome-mRNA hybrids: a breakthrough in targeted miRNA delivery for diabetic kidney fibrosis therapy
Source: Front Bioeng Biotechnol. 2026 Mar 2;14:1709588. doi: 10.3389/fbioe.2026.1709588 (PMC12989603; doi:10.3389/fbioe.2026.1709588)
Supplement: Supplementary file 2 [file Table1.docx]

**Table S1. RT-qPCR Primer Sequence.**

| **Gene** | **Primer Sequence** |
| --- | --- |
| PARP1 | Forward: 5′-CTCTCCCAGAACAAGGACGAAG-3′ |
|  | Reverse: 5′-CCGCTTTCACTTCCTCCATCTTC-3′ |
| TGFβ1 | Forward: 5′-TGATACGCCTGAGTGGCTGTCT-3′ |
|  | Reverse: 5′-CACAAGAGCAGTGAGCGCTGAA-3′ |
| GAPDH | Forward: 5′-CATCACTGCCACCCAGAAGACTG-3′ |
|  | Reverse: 5′-ATGCCAGTGAGCTTCCCGTTCAG-3′ |
