# Supplementary figures and images for: Bioengineered exosome-mRNA hybrids: a breakthrough in targeted miRNA delivery for diabetic kidney fibrosis therapy (part 2 of 3)
Source: Front Bioeng Biotechnol. 2026 Mar 2;14:1709588. doi: 10.3389/fbioe.2026.1709588 (PMC12989603; doi:10.3389/fbioe.2026.1709588)

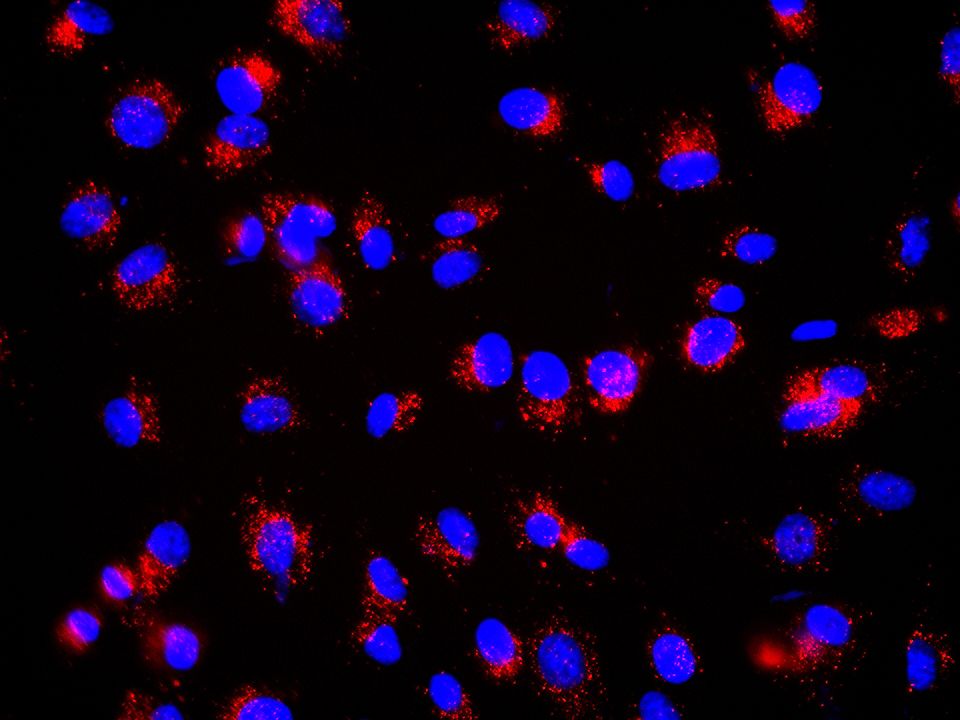

Supplement: Supplementary file 7 [file DataSheet5.zip › original images of figure 6/图6A-3-5.jpg]

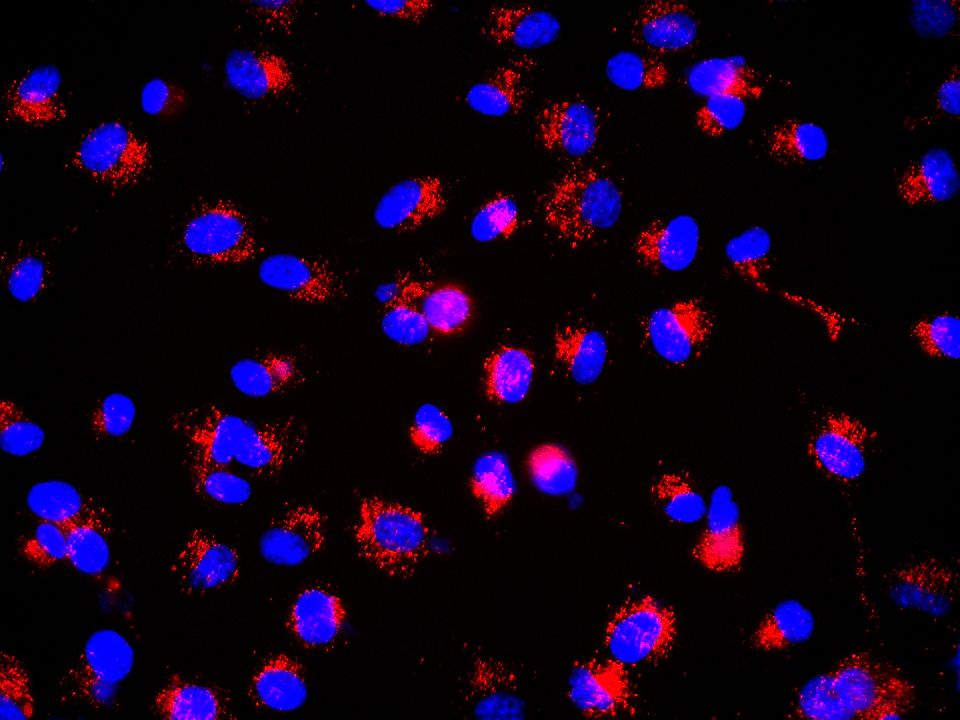

Supplement: Supplementary file 7 [file DataSheet5.zip › original images of figure 6/图6A-3-6.jpg]

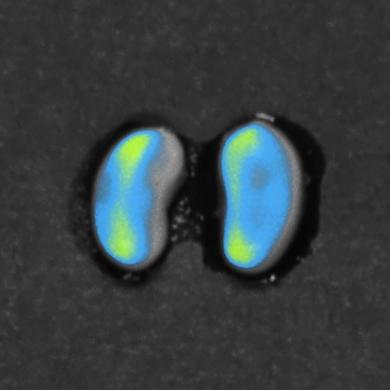

Supplement: Supplementary file 7 [file DataSheet5.zip › original images of figure 6/图6C-1-16.jpg]

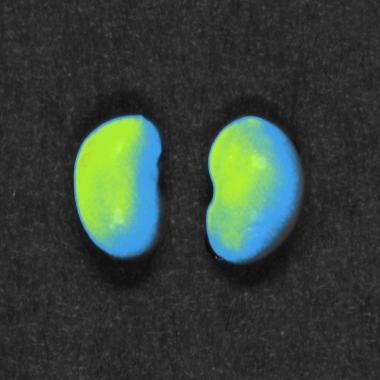

Supplement: Supplementary file 7 [file DataSheet5.zip › original images of figure 6/图6C-1-24.jpg]

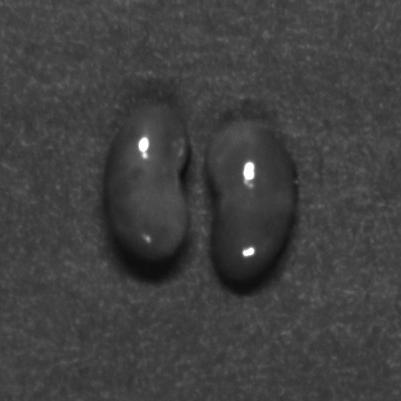

Supplement: Supplementary file 7 [file DataSheet5.zip › original images of figure 6/图6C-1-4.jpg]

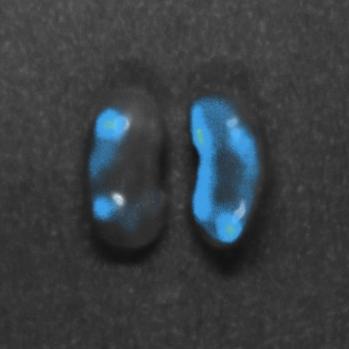

Supplement: Supplementary file 7 [file DataSheet5.zip › original images of figure 6/图6C-1-8.jpg]

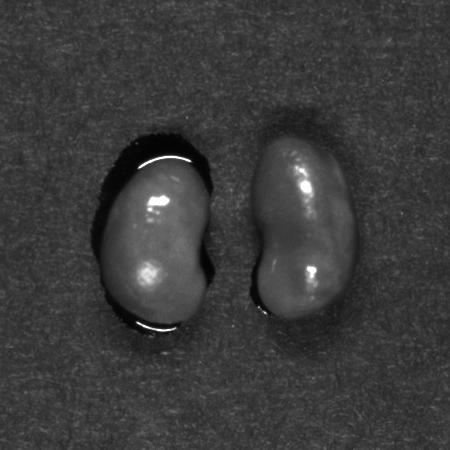

Supplement: Supplementary file 7 [file DataSheet5.zip › original images of figure 6/图6C-1-P.jpg]

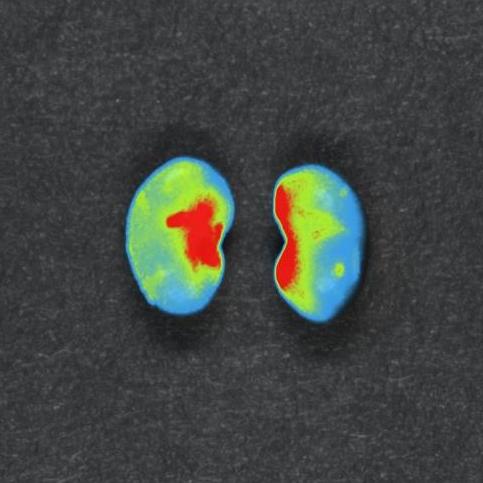

Supplement: Supplementary file 7 [file DataSheet5.zip › original images of figure 6/图6C-2-16.jpg]

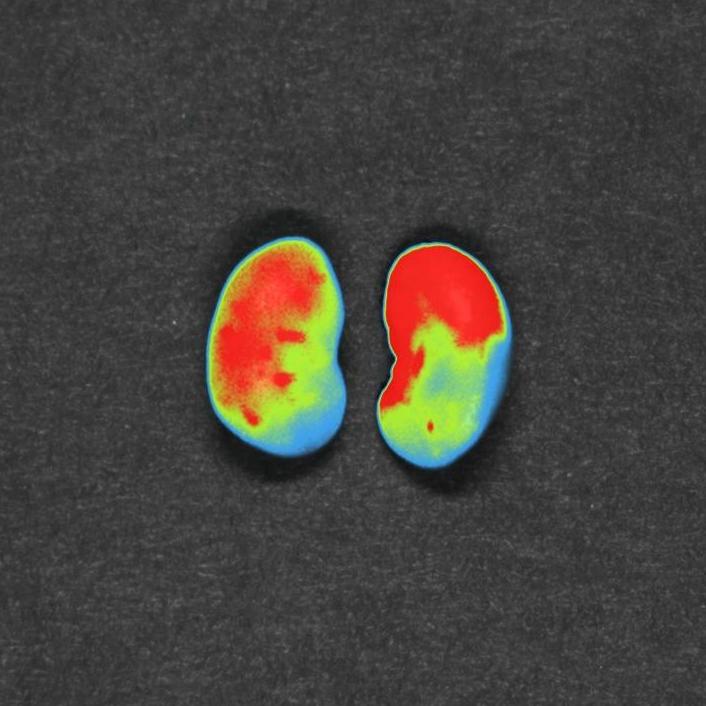

Supplement: Supplementary file 7 [file DataSheet5.zip › original images of figure 6/图6C-2-24.jpg]

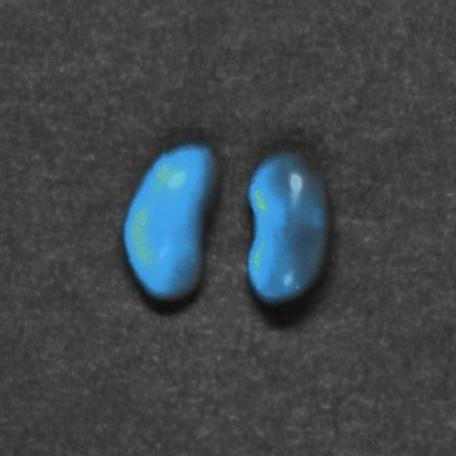

Supplement: Supplementary file 7 [file DataSheet5.zip › original images of figure 6/图6C-2-4.jpg]

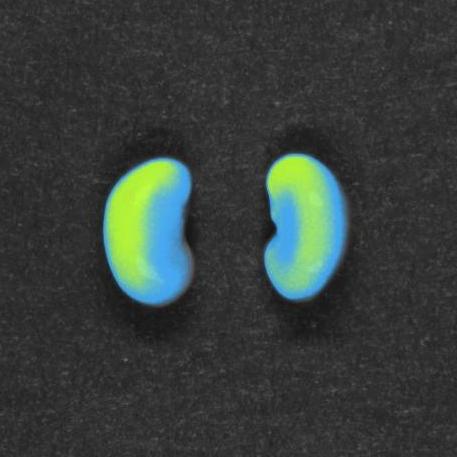

Supplement: Supplementary file 7 [file DataSheet5.zip › original images of figure 6/图6C-2-8.jpg]

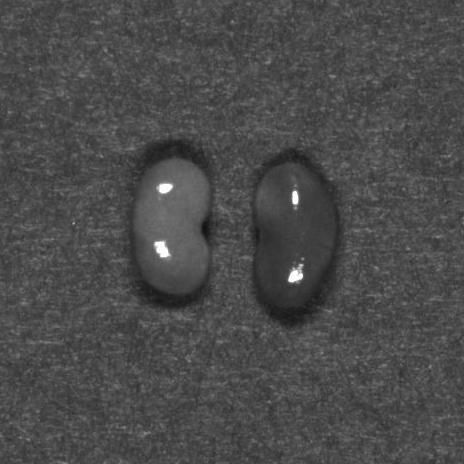

Supplement: Supplementary file 7 [file DataSheet5.zip › original images of figure 6/图6C-2-P.jpg]

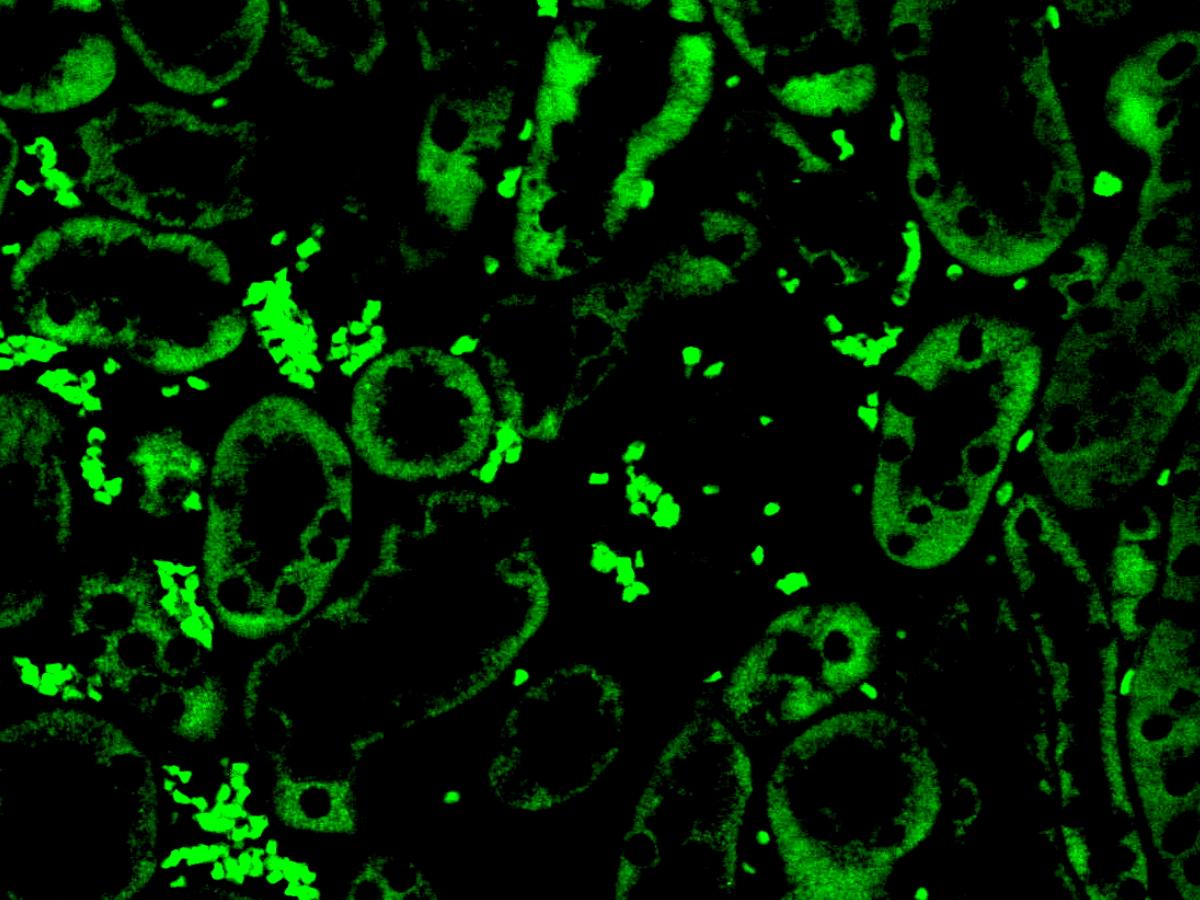

Supplement: Supplementary file 7 [file DataSheet5.zip › original images of figure 6/图6E-1-1-1(Pre-FITC).jpg]

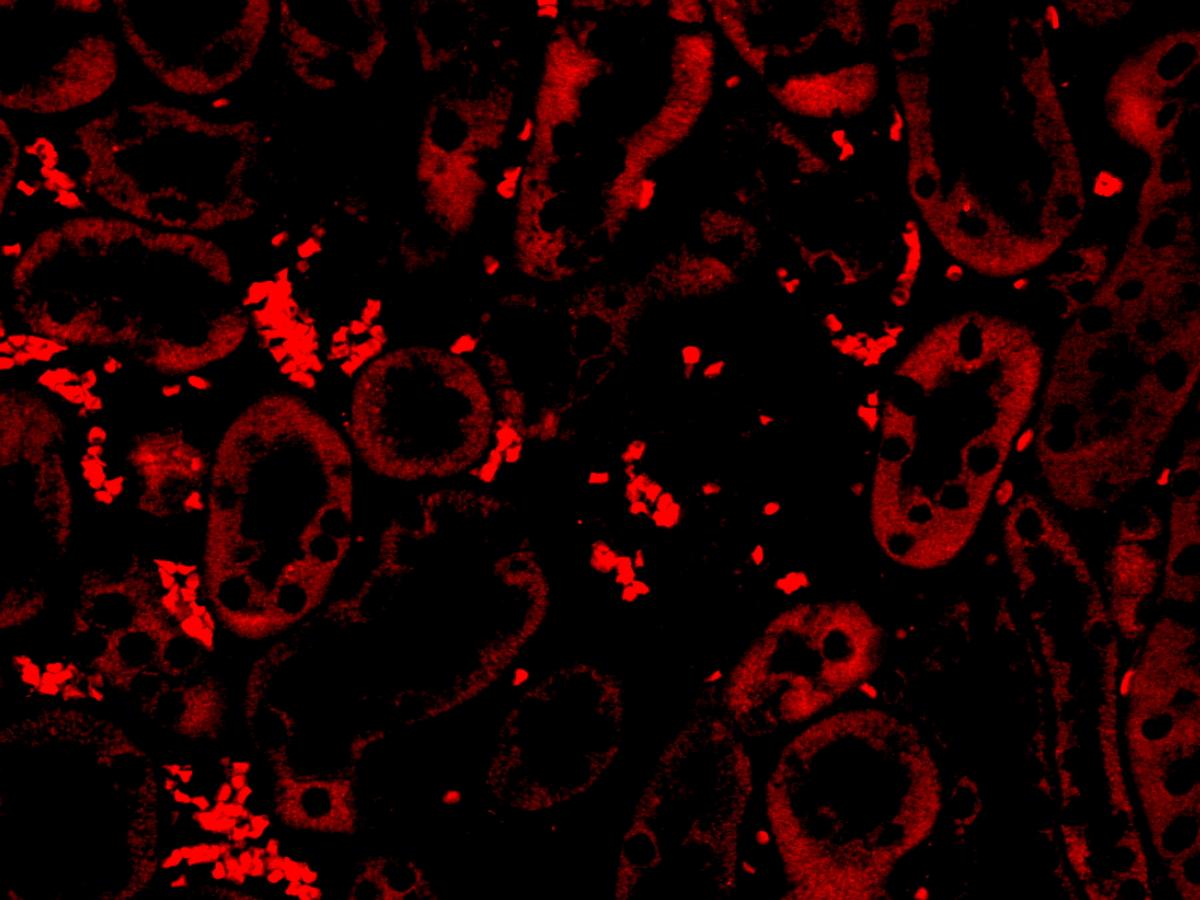

Supplement: Supplementary file 7 [file DataSheet5.zip › original images of figure 6/图6E-1-1-2(Pre-TRITC).jpg]

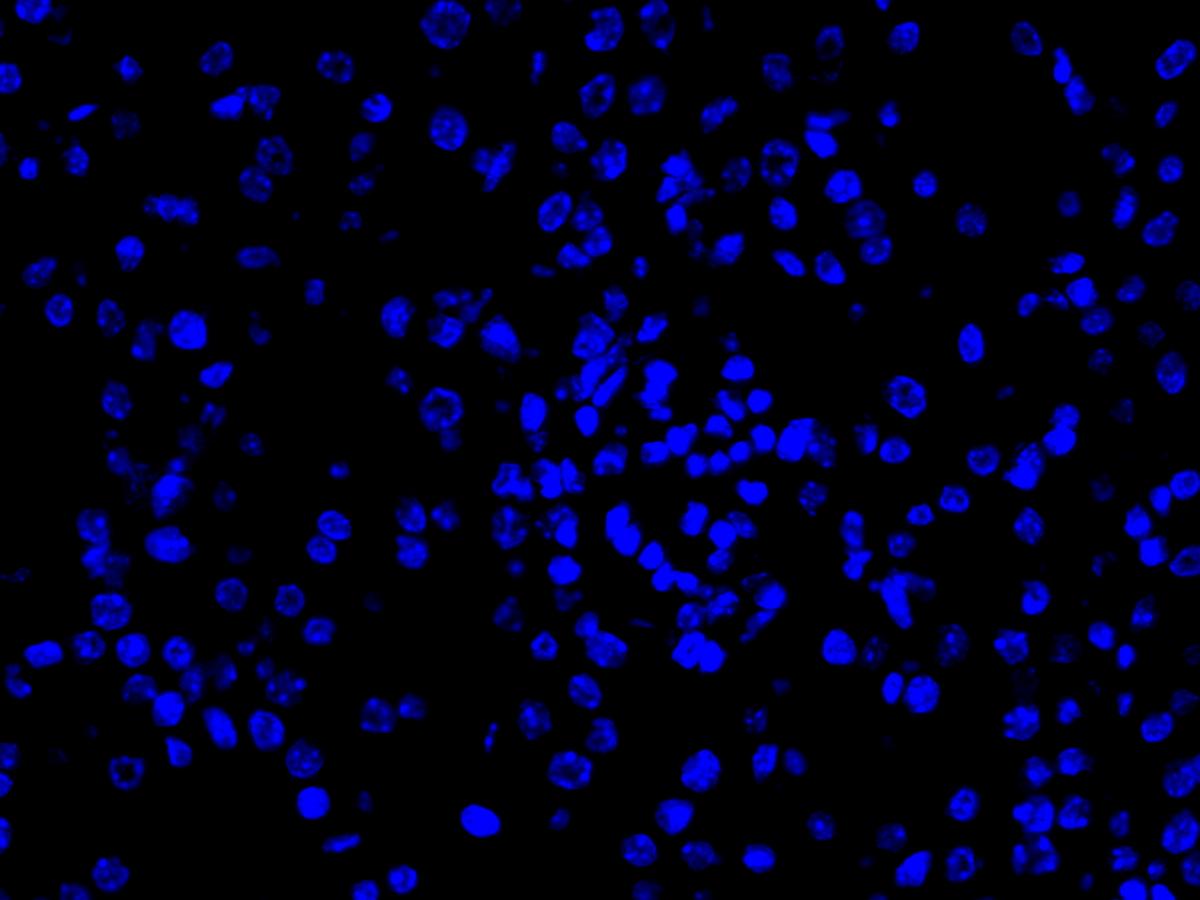

Supplement: Supplementary file 7 [file DataSheet5.zip › original images of figure 6/图6E-1-1-3(Pre-DAPI).jpg]

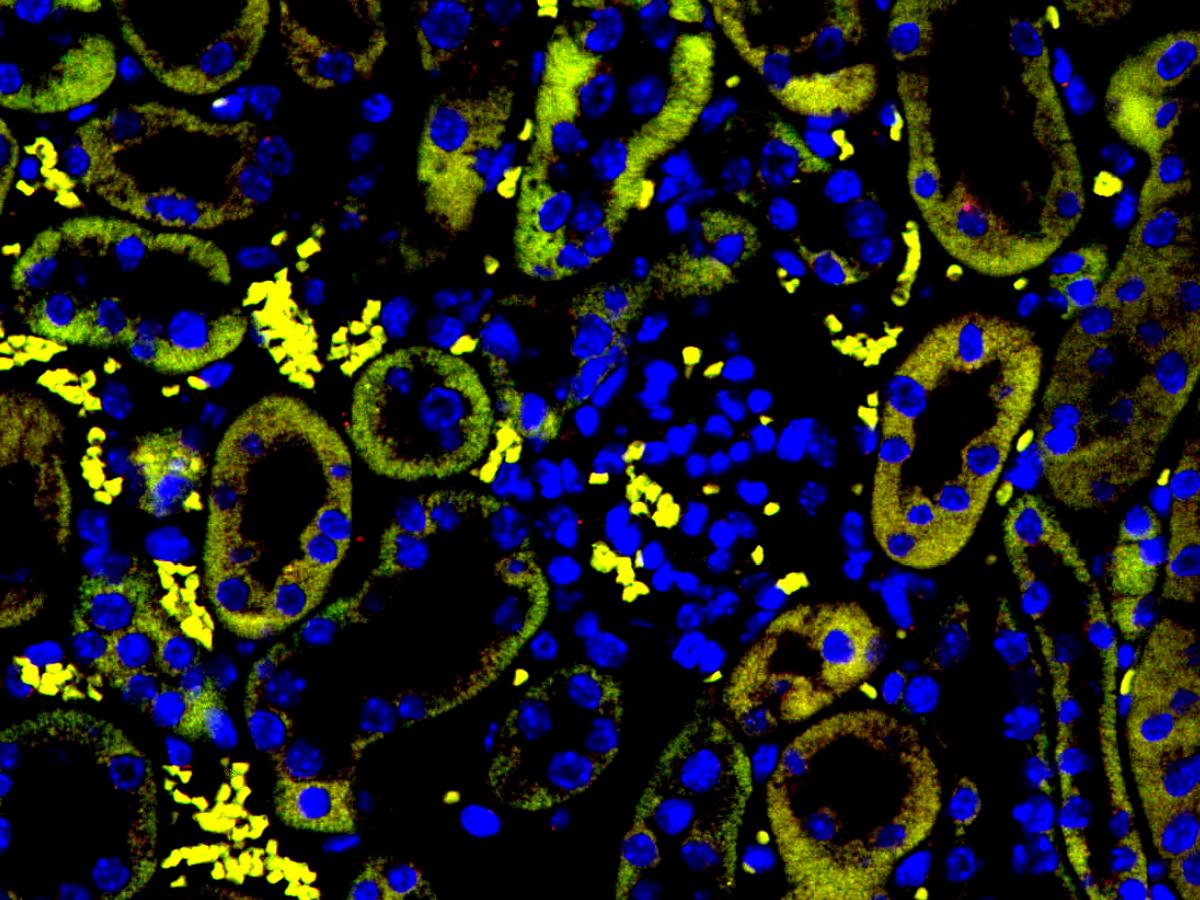

Supplement: Supplementary file 7 [file DataSheet5.zip › original images of figure 6/图6E-1-1-4(Pre-Merge).jpg]

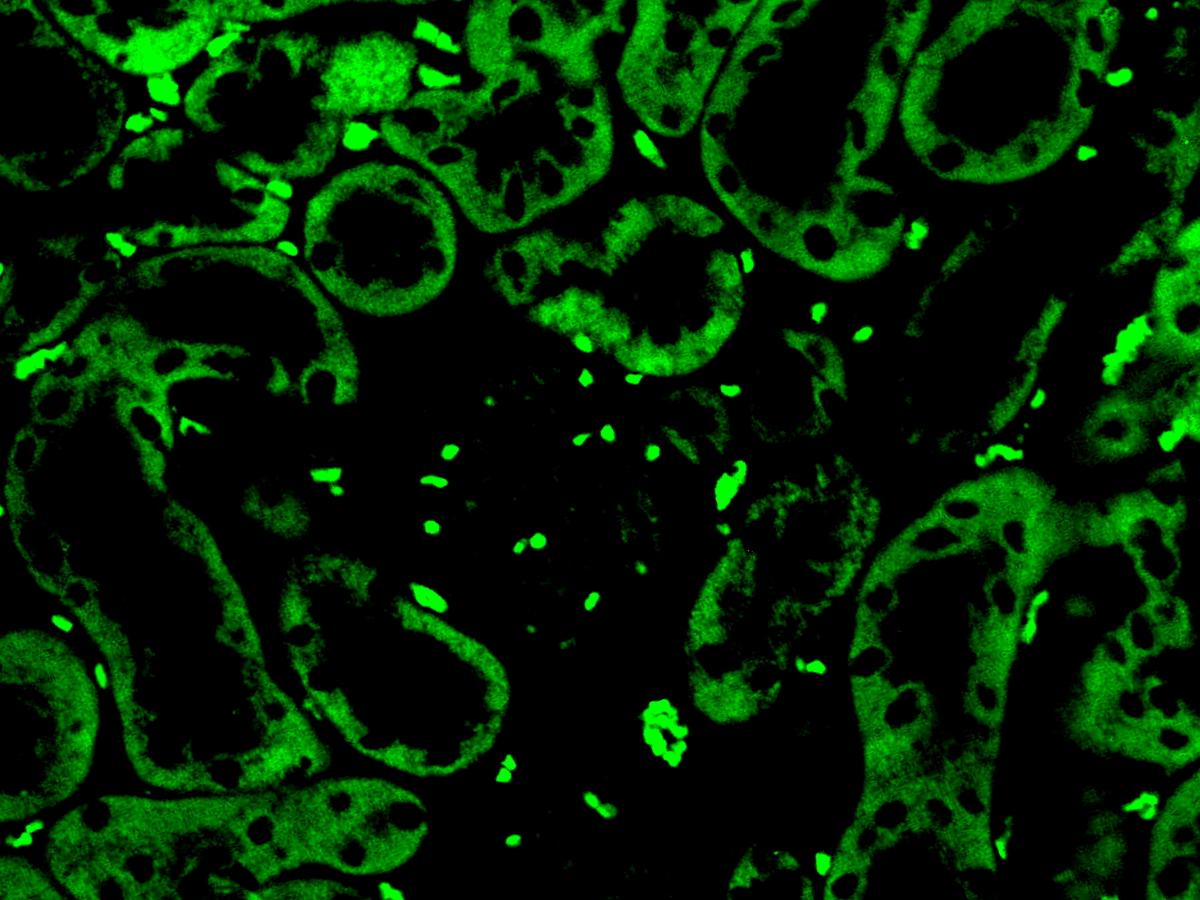

Supplement: Supplementary file 7 [file DataSheet5.zip › original images of figure 6/图6E-1-2-1(4h-FITC).jpg]

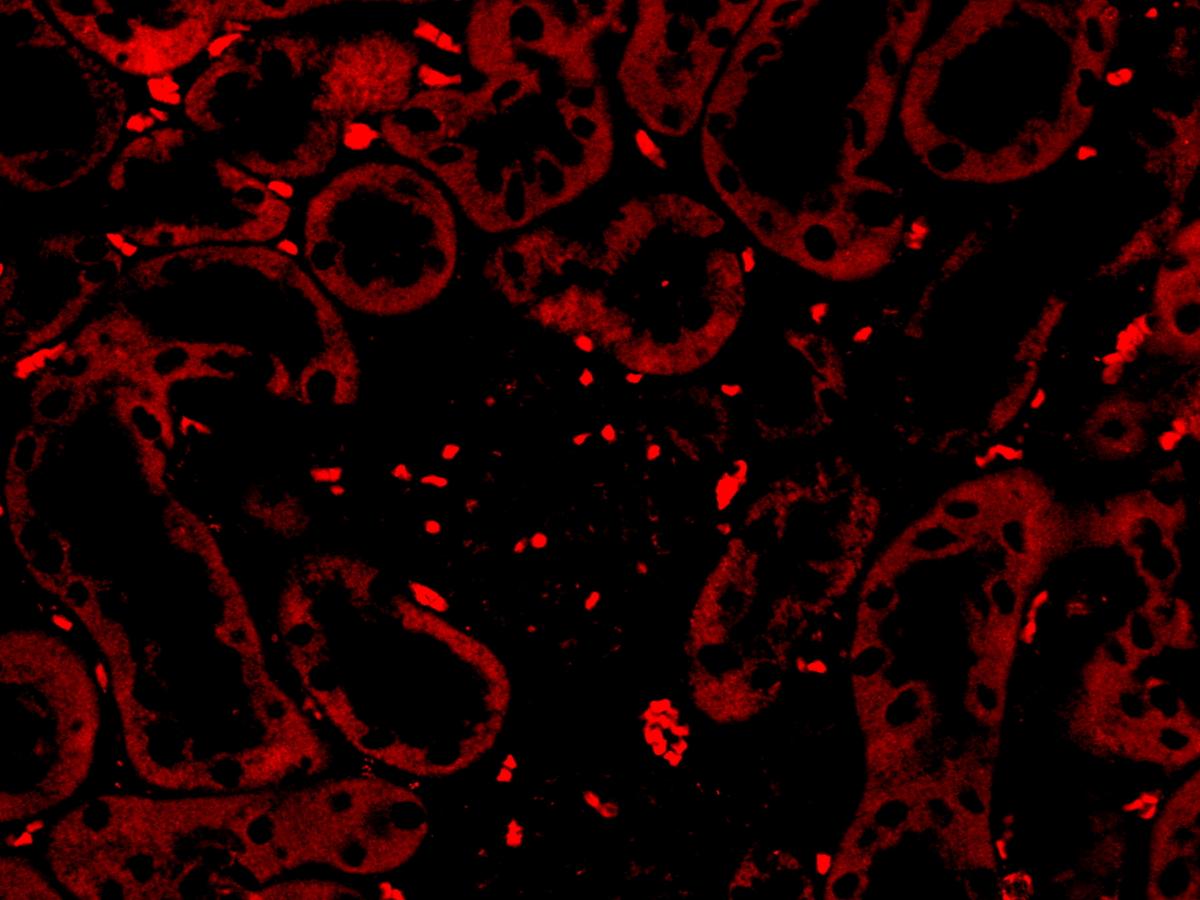

Supplement: Supplementary file 7 [file DataSheet5.zip › original images of figure 6/图6E-1-2-2(4h-TRITC).jpg]

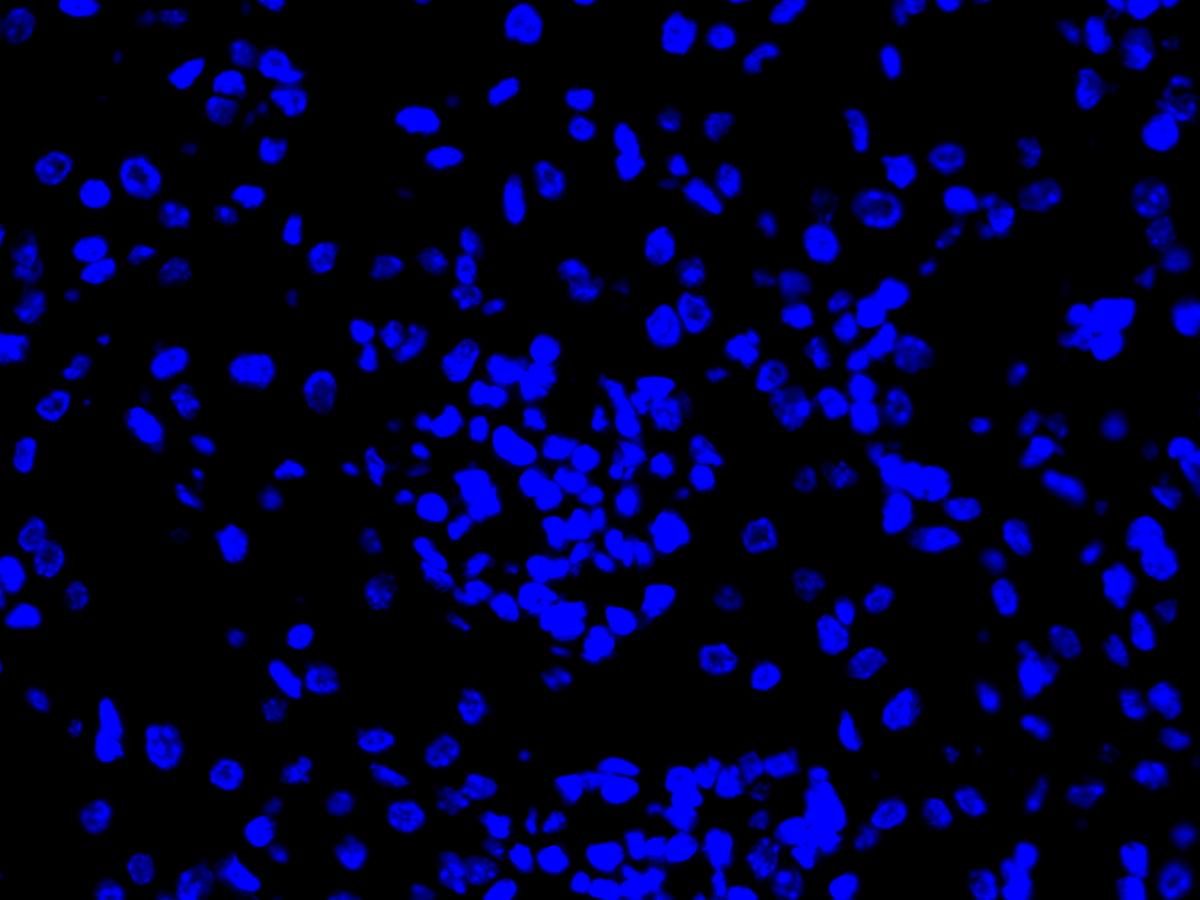

Supplement: Supplementary file 7 [file DataSheet5.zip › original images of figure 6/图6E-1-2-3(4h-DAPI).jpg]

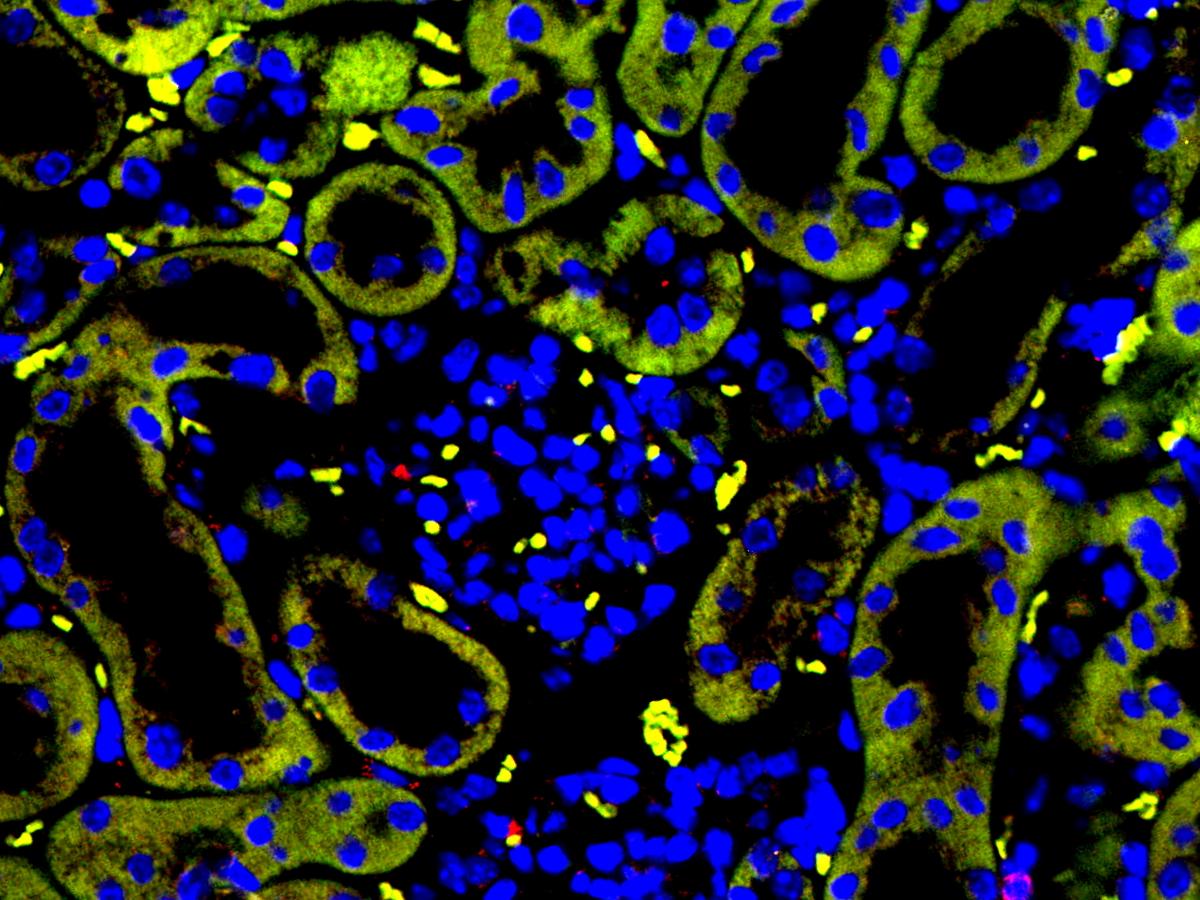

Supplement: Supplementary file 7 [file DataSheet5.zip › original images of figure 6/图6E-1-2-4(4h-Merge).jpg]

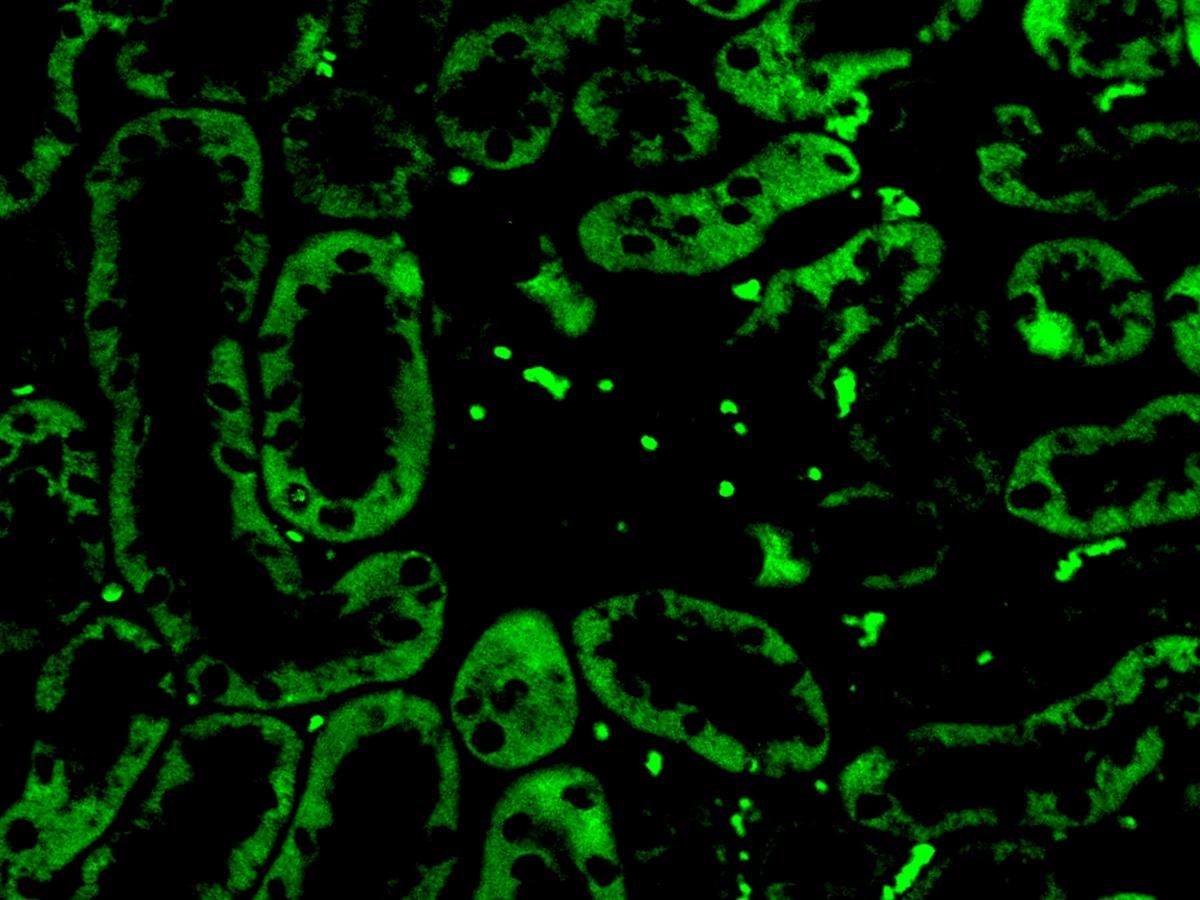

Supplement: Supplementary file 7 [file DataSheet5.zip › original images of figure 6/图6E-1-3-1(8h-FITC).jpg]

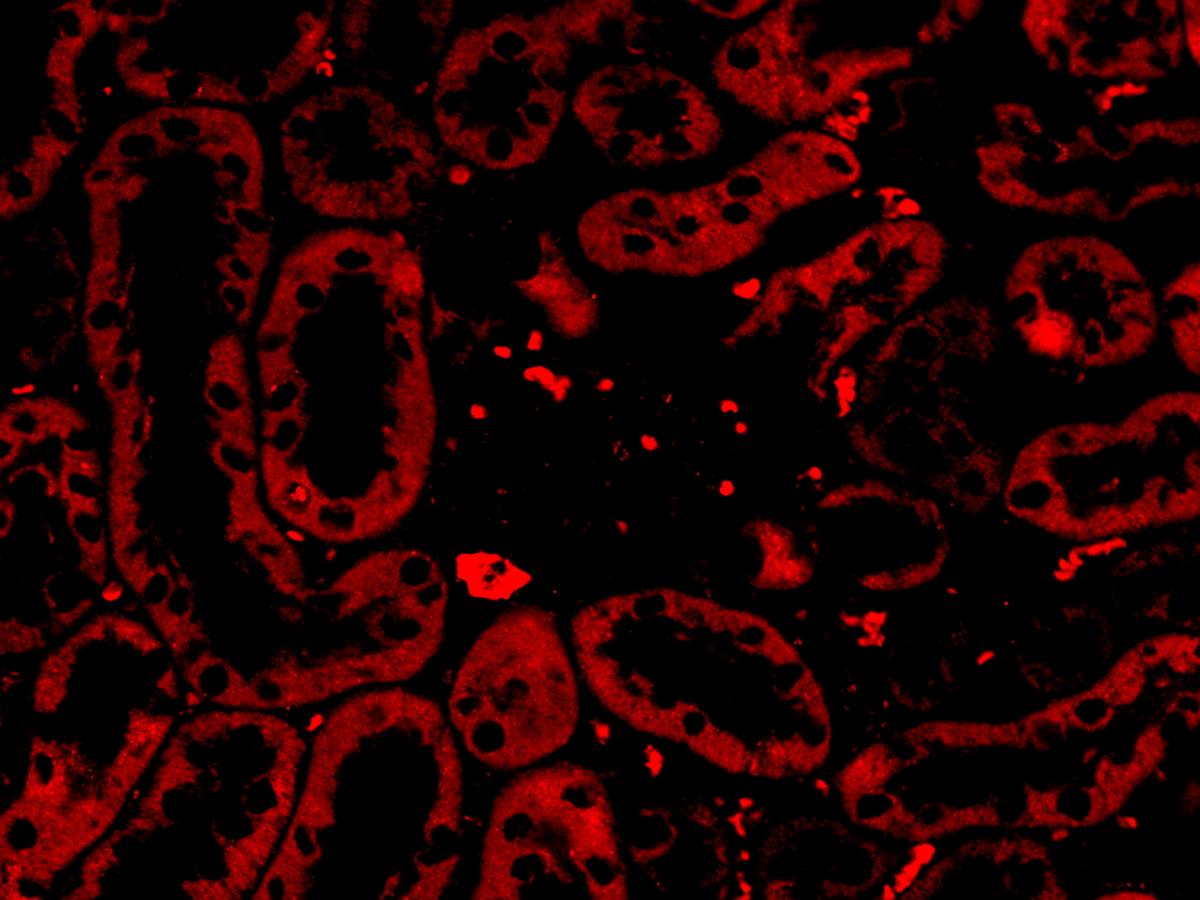

Supplement: Supplementary file 7 [file DataSheet5.zip › original images of figure 6/图6E-1-3-2(8h-TRITC).jpg]

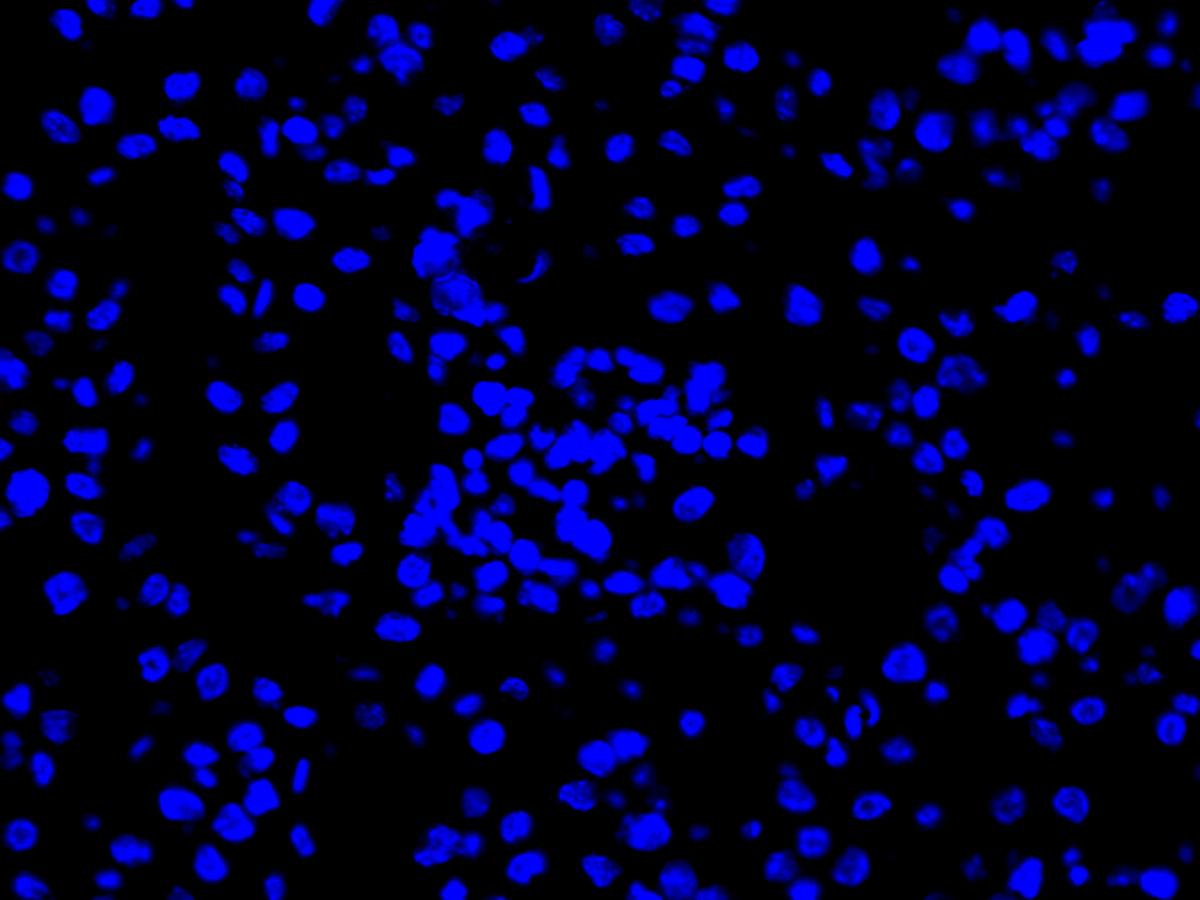

Supplement: Supplementary file 7 [file DataSheet5.zip › original images of figure 6/图6E-1-3-3(8h-DAPI).jpg]

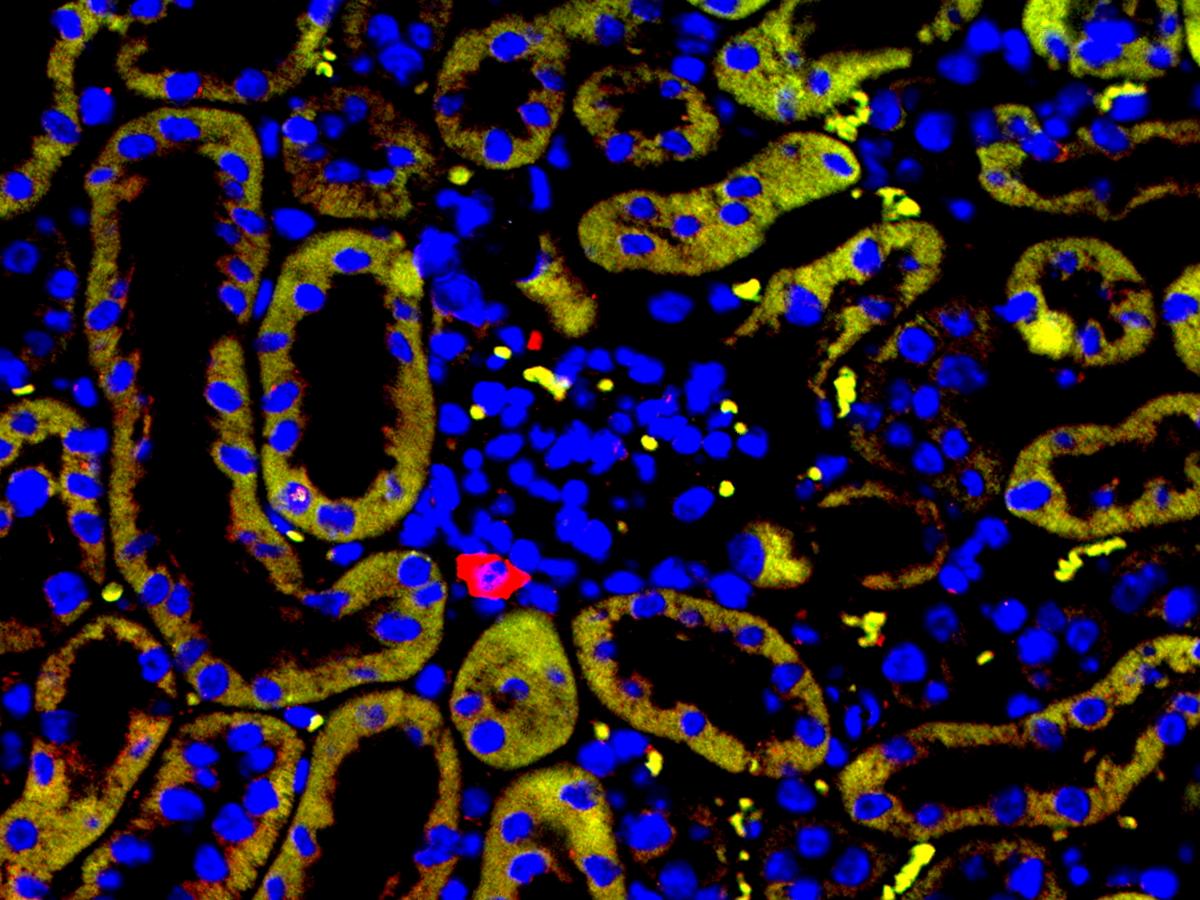

Supplement: Supplementary file 7 [file DataSheet5.zip › original images of figure 6/图6E-1-3-4(8h-Merge).jpg]

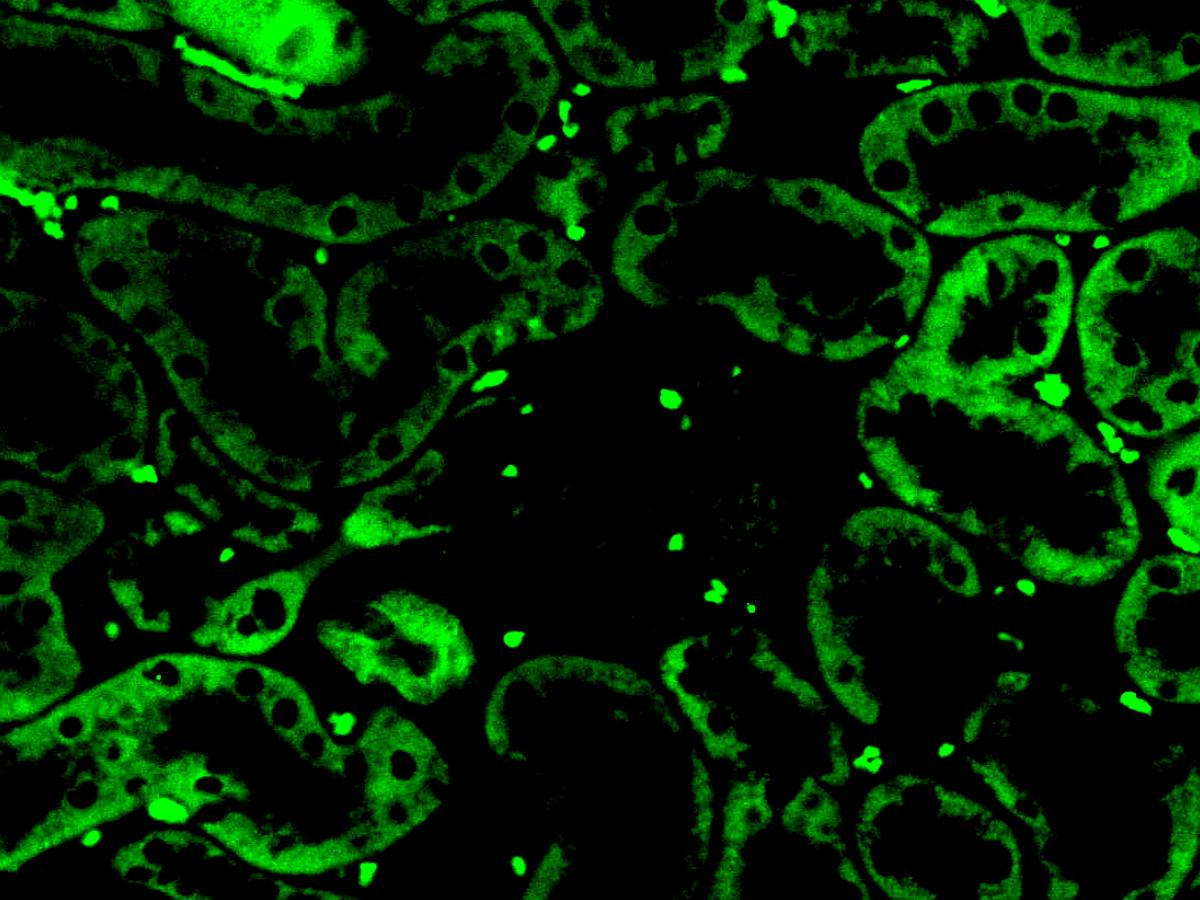

Supplement: Supplementary file 7 [file DataSheet5.zip › original images of figure 6/图6E-1-4-1(16h-FITC).jpg]

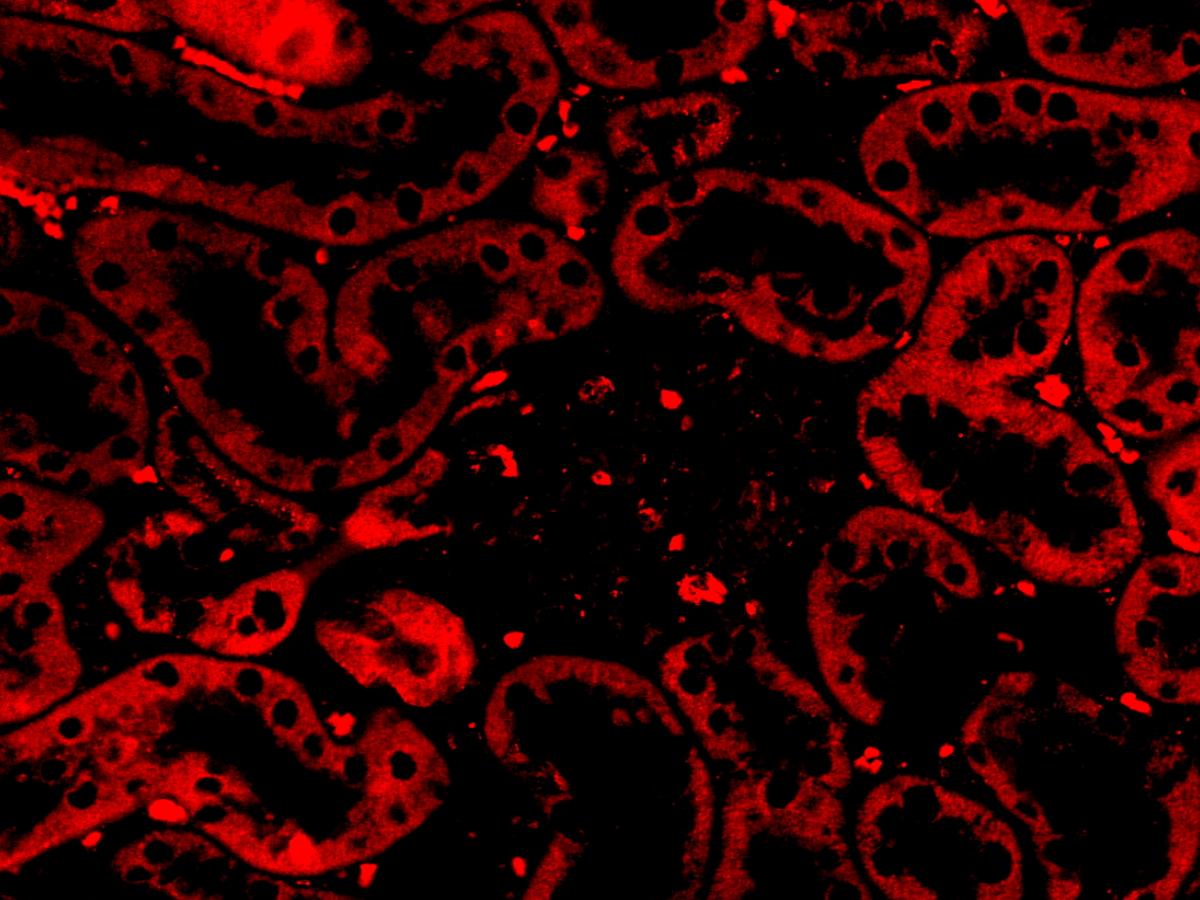

Supplement: Supplementary file 7 [file DataSheet5.zip › original images of figure 6/图6E-1-4-2(16h-TRITC).jpg]

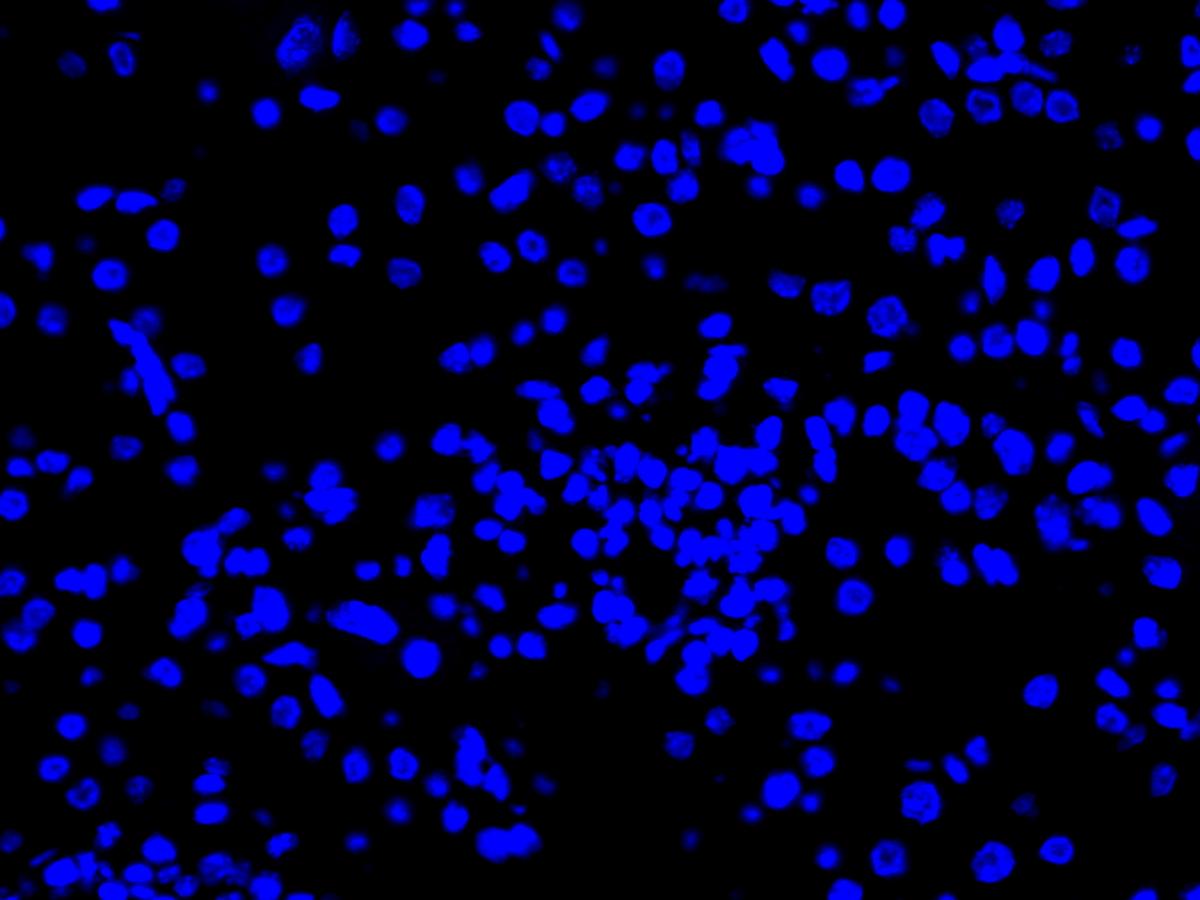

Supplement: Supplementary file 7 [file DataSheet5.zip › original images of figure 6/图6E-1-4-3(16h-DAPI).jpg]

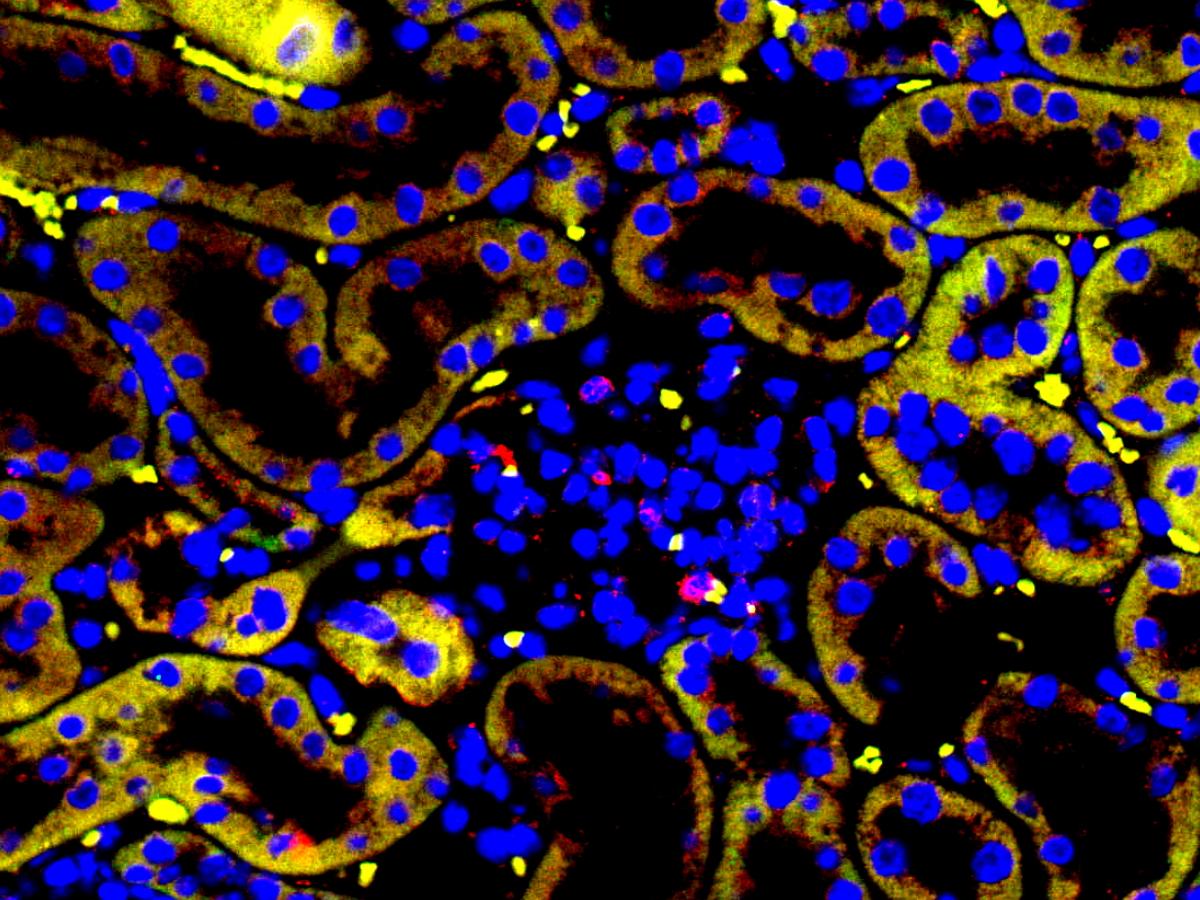

Supplement: Supplementary file 7 [file DataSheet5.zip › original images of figure 6/图6E-1-4-4(16h-Merge).jpg]

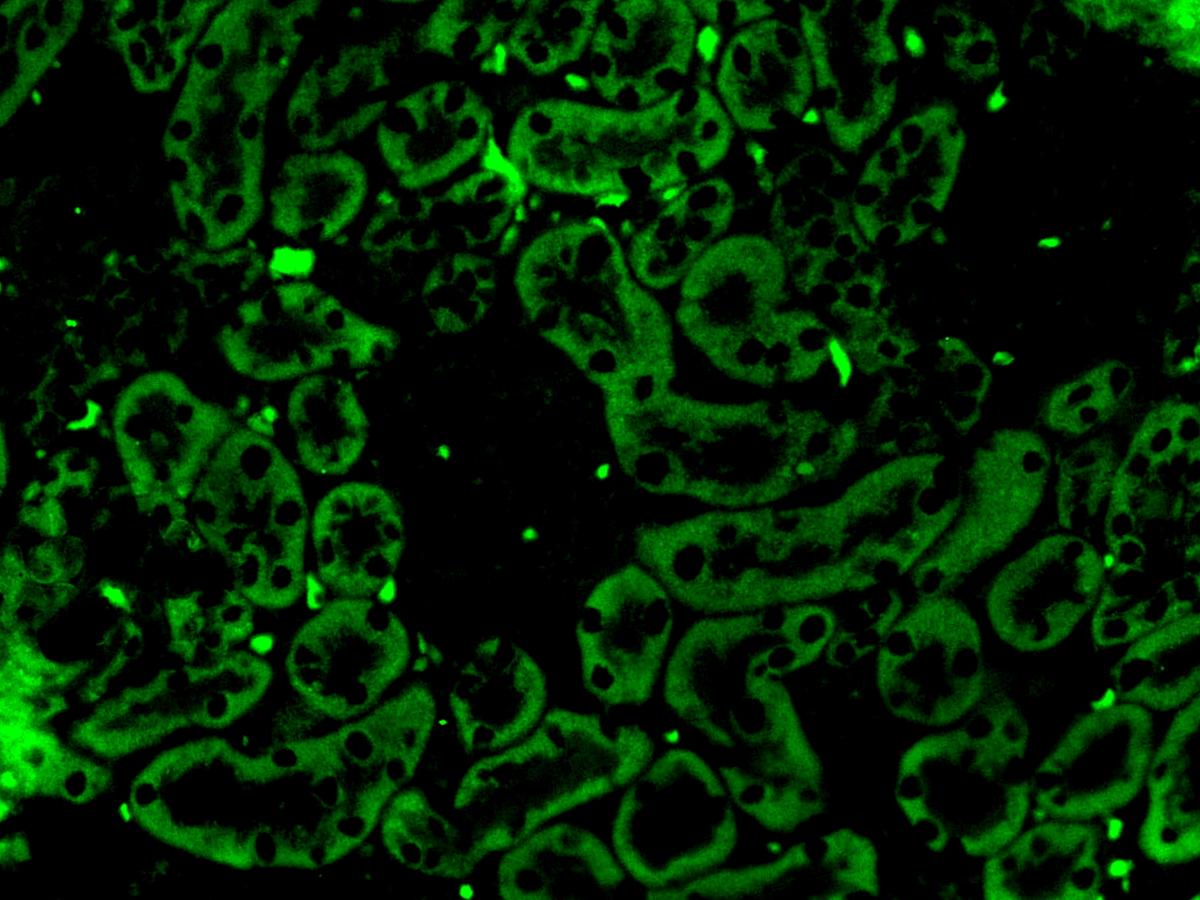

Supplement: Supplementary file 7 [file DataSheet5.zip › original images of figure 6/图6E-1-5-1(24h-FITC).jpg]

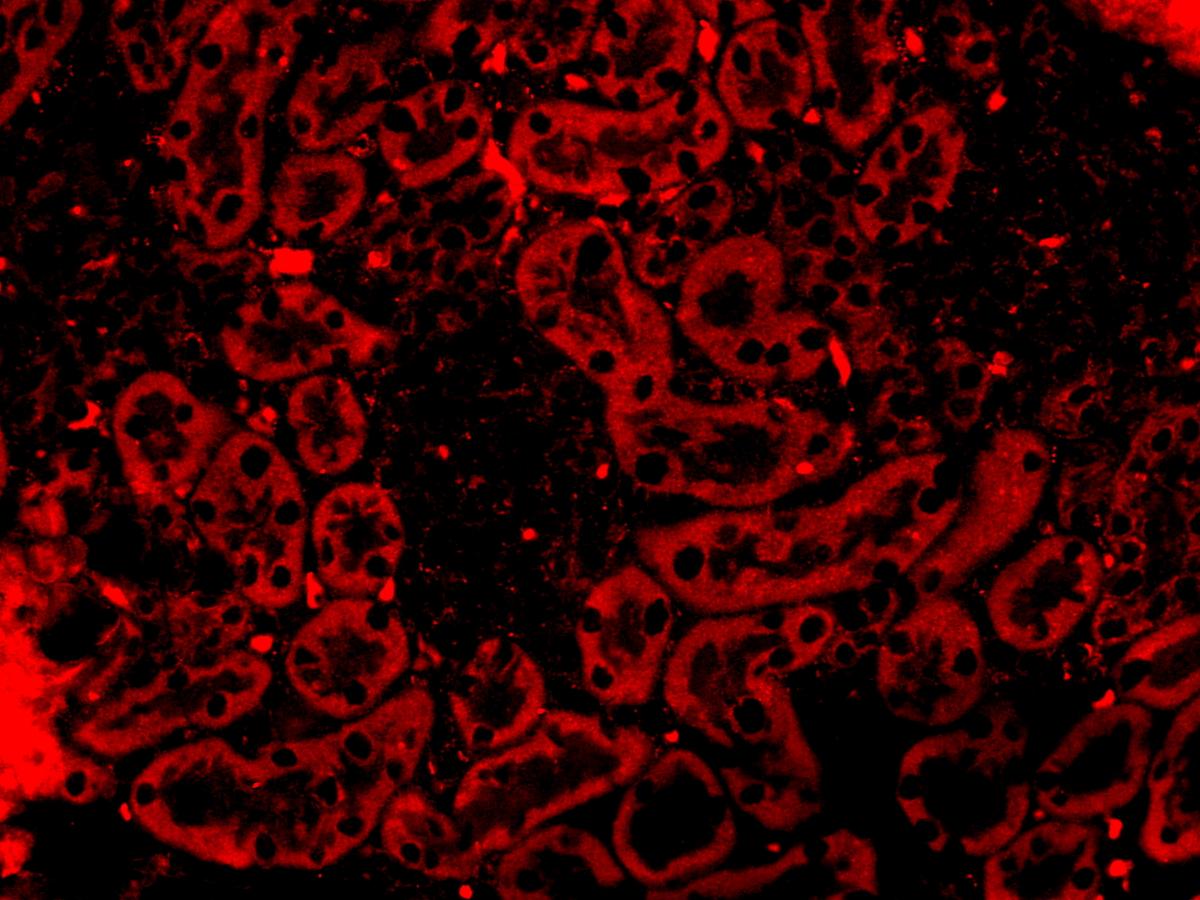

Supplement: Supplementary file 7 [file DataSheet5.zip › original images of figure 6/图6E-1-5-2(24h-TRITC).jpg]

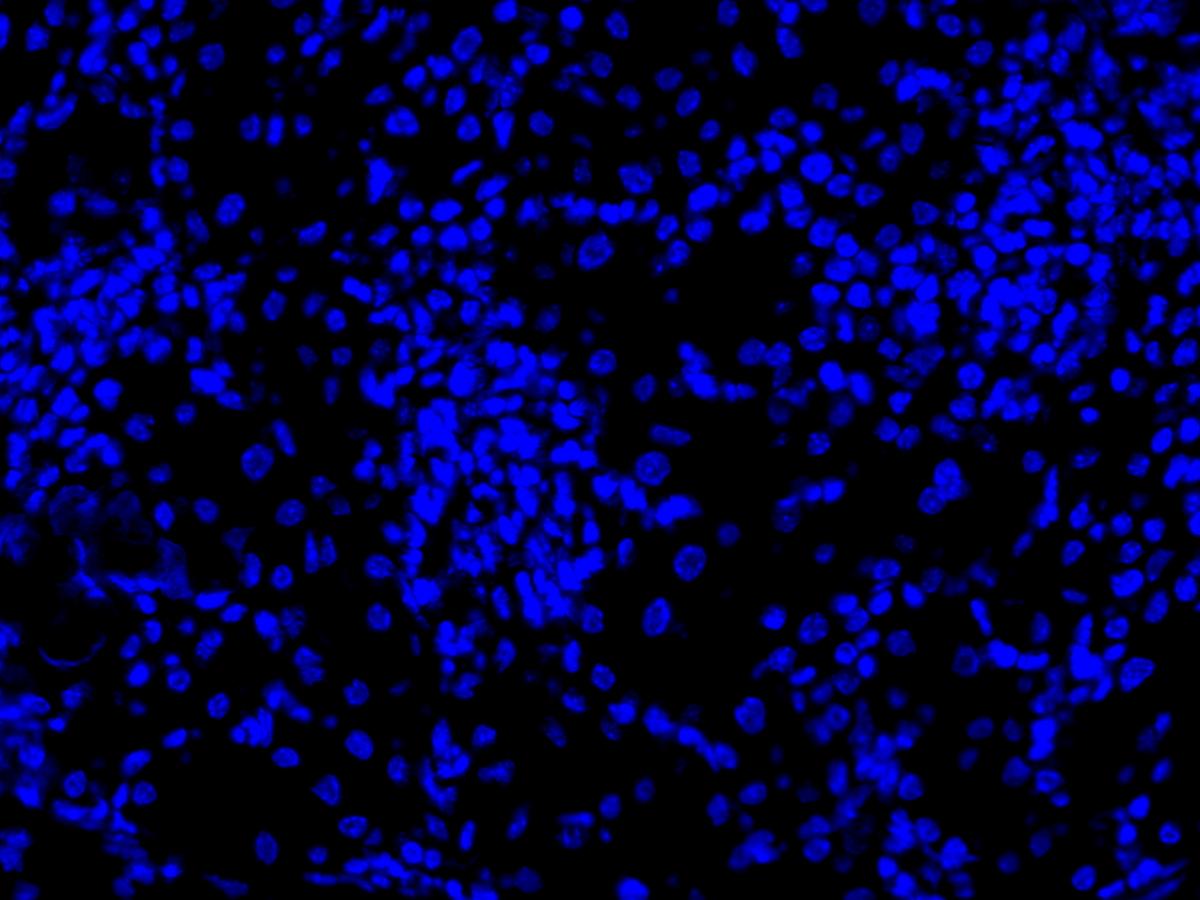

Supplement: Supplementary file 7 [file DataSheet5.zip › original images of figure 6/图6E-1-5-3(24h-DAPI).jpg]

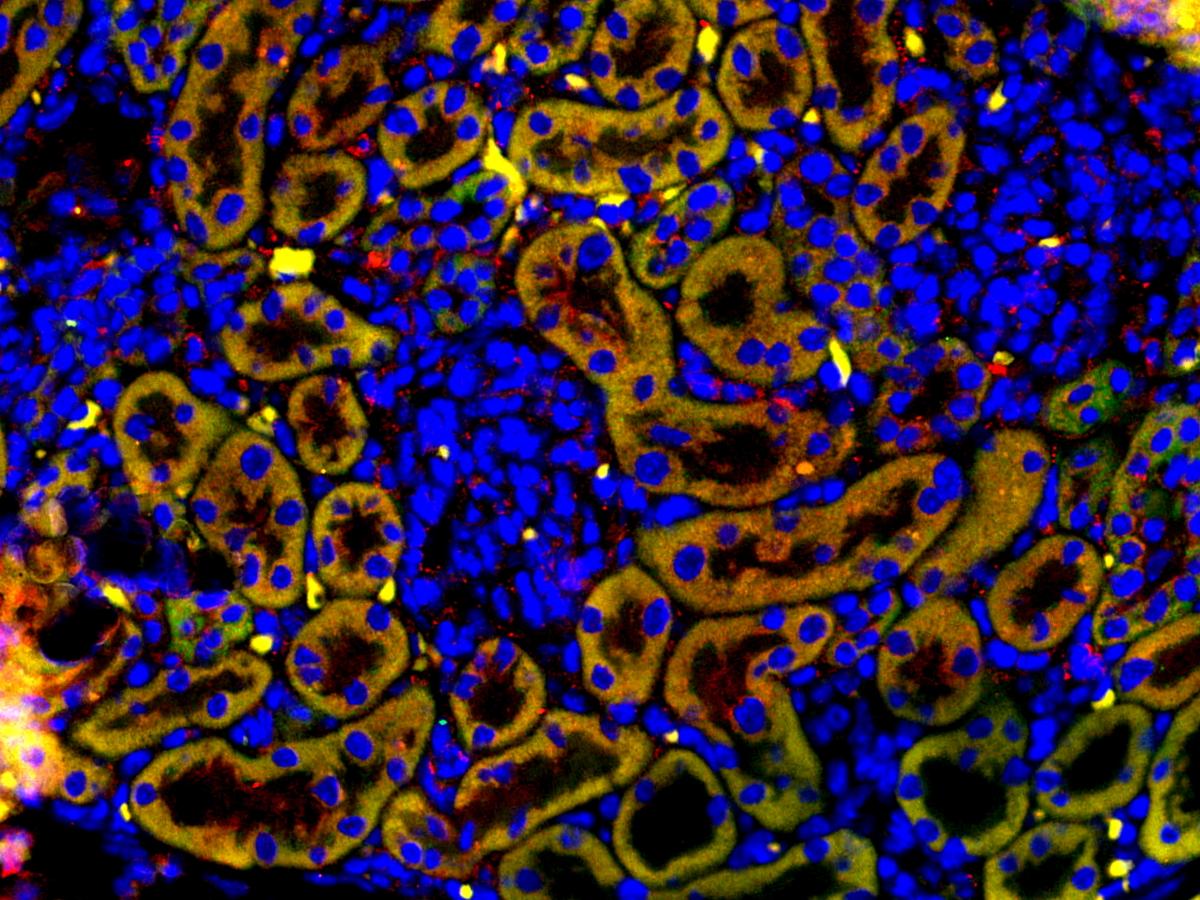

Supplement: Supplementary file 7 [file DataSheet5.zip › original images of figure 6/图6E-1-5-4(24h-Merge).jpg]

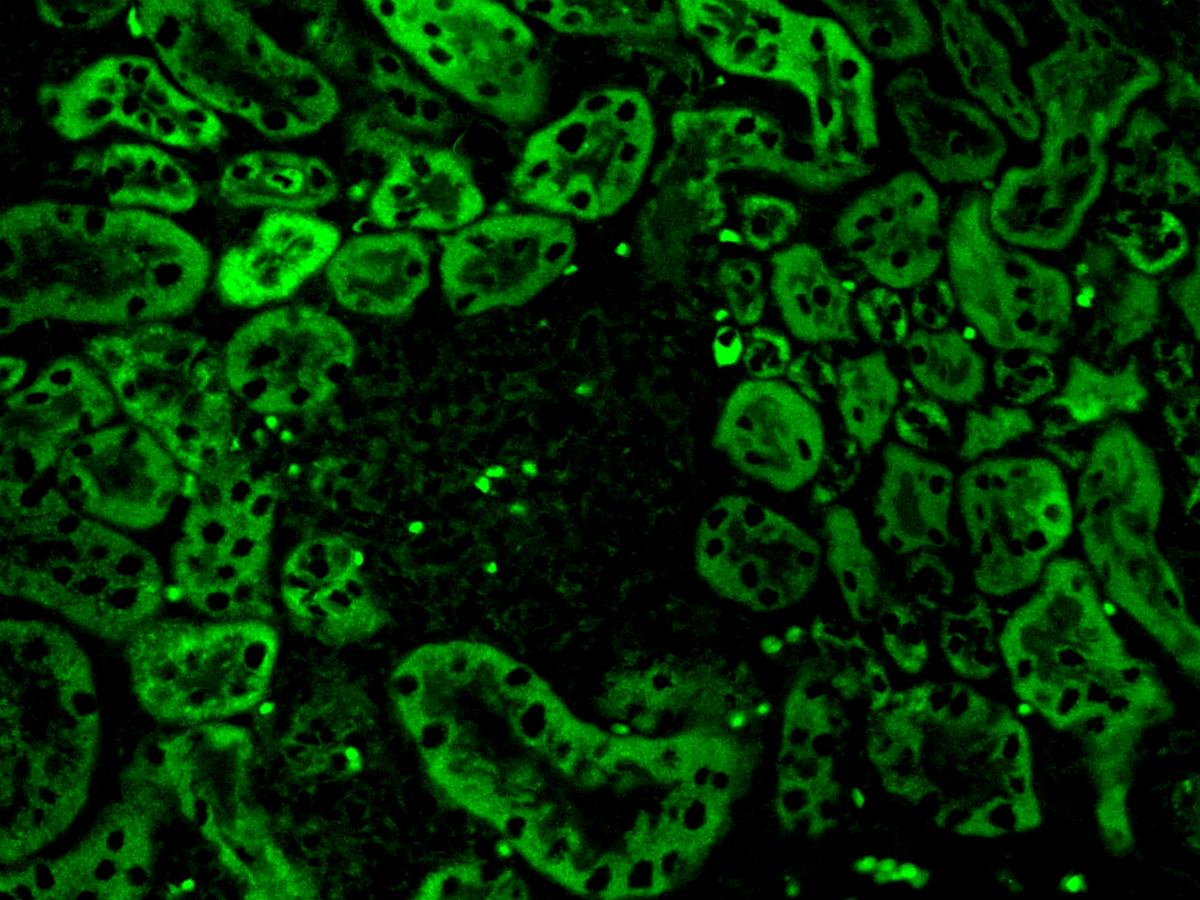

Supplement: Supplementary file 7 [file DataSheet5.zip › original images of figure 6/图6E-1-6-1(48h-FITC).jpg]

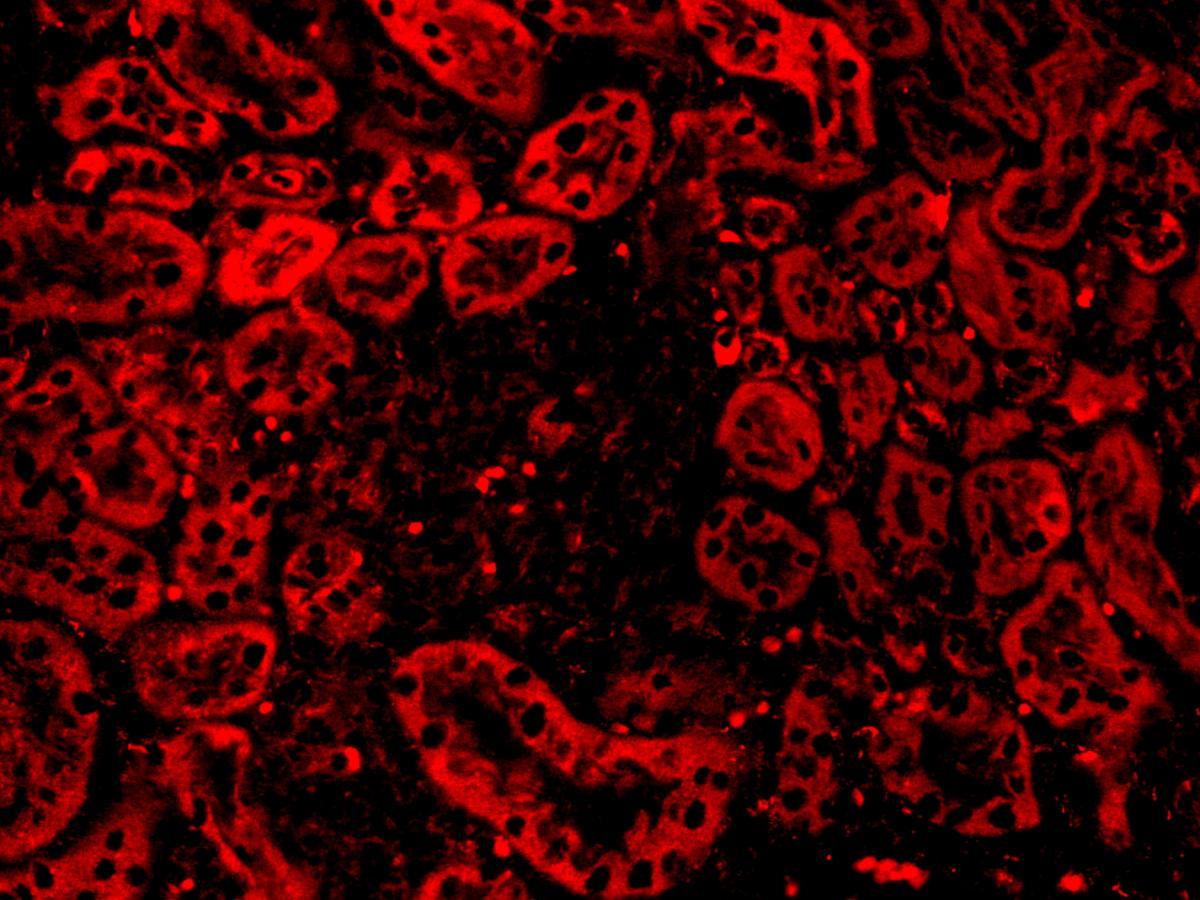

Supplement: Supplementary file 7 [file DataSheet5.zip › original images of figure 6/图6E-1-6-2(48h-TRITC).jpg]

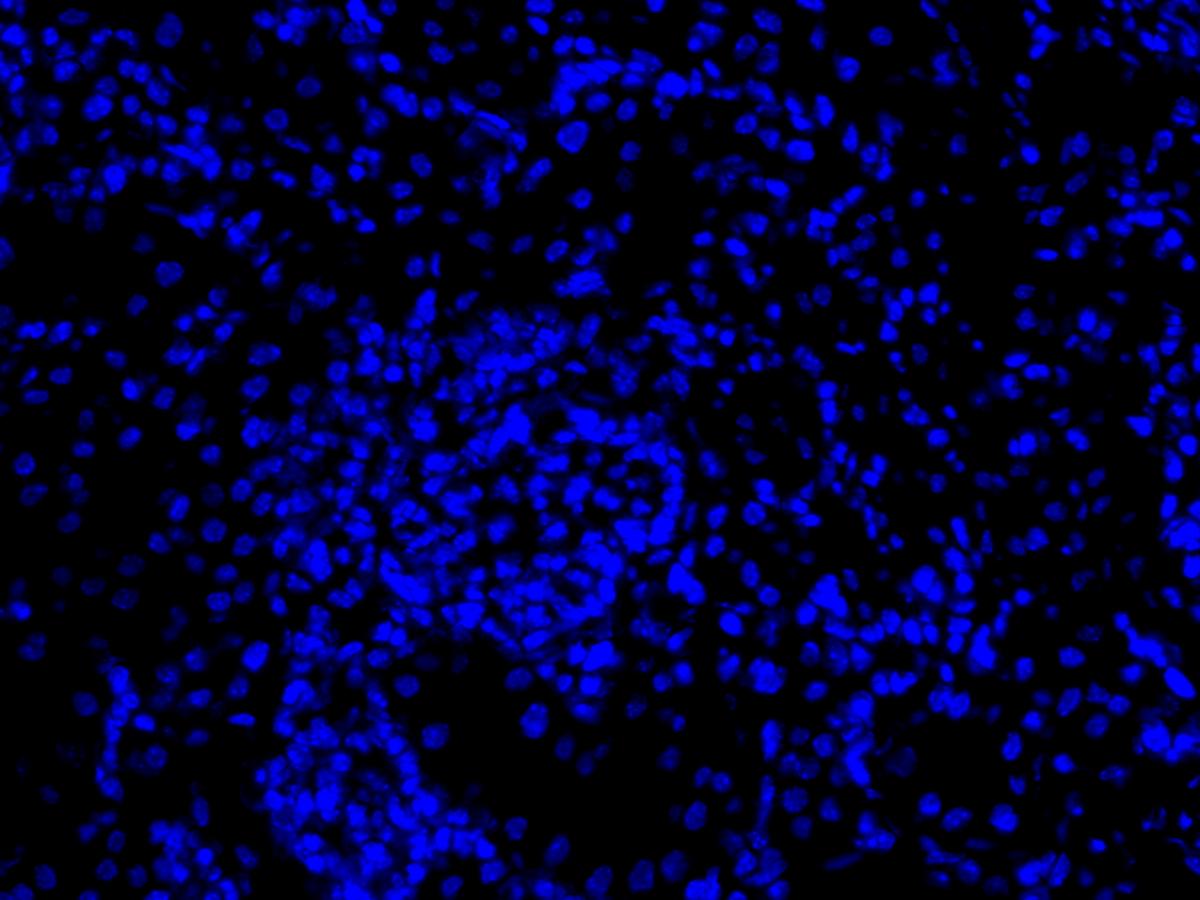

Supplement: Supplementary file 7 [file DataSheet5.zip › original images of figure 6/图6E-1-6-3(48h-DAPI).jpg]

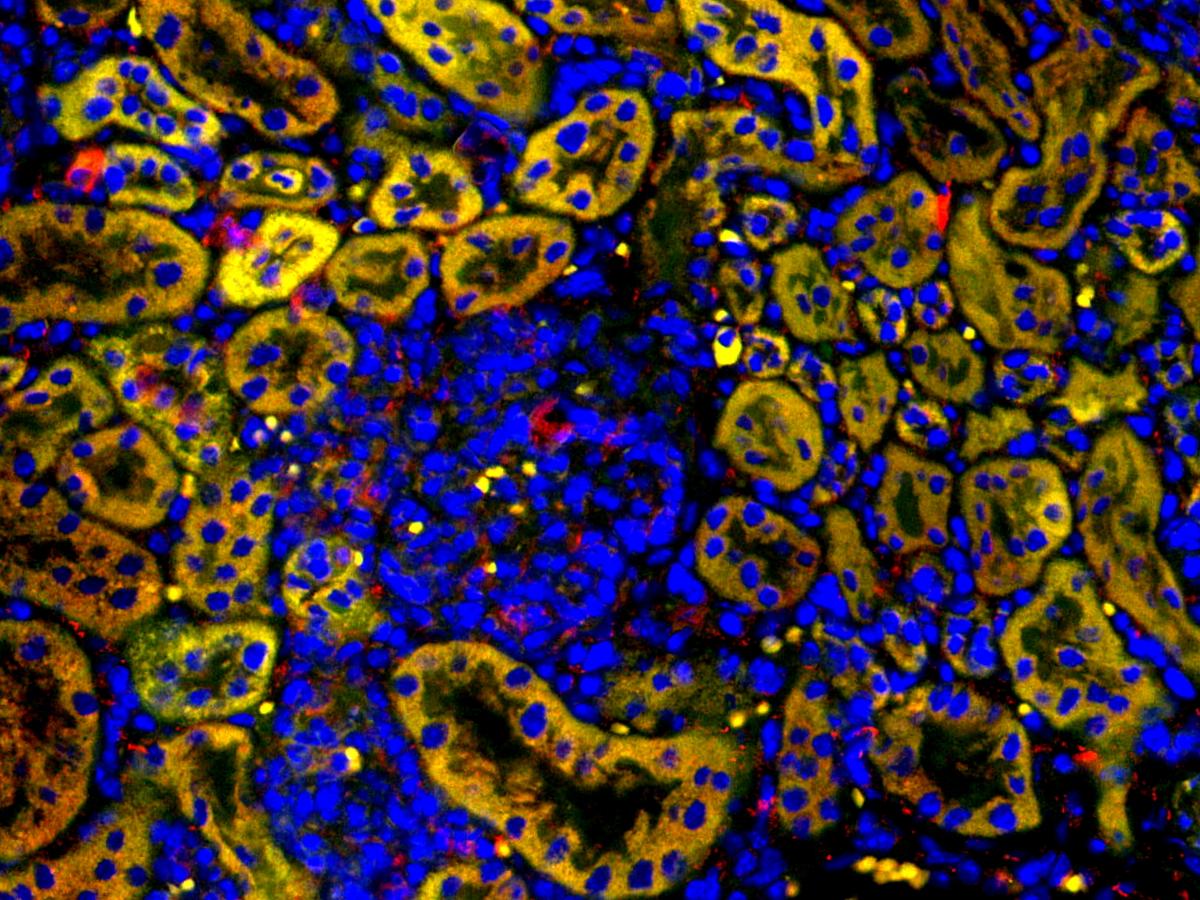

Supplement: Supplementary file 7 [file DataSheet5.zip › original images of figure 6/图6E-1-6-4(48h-Merge).jpg]

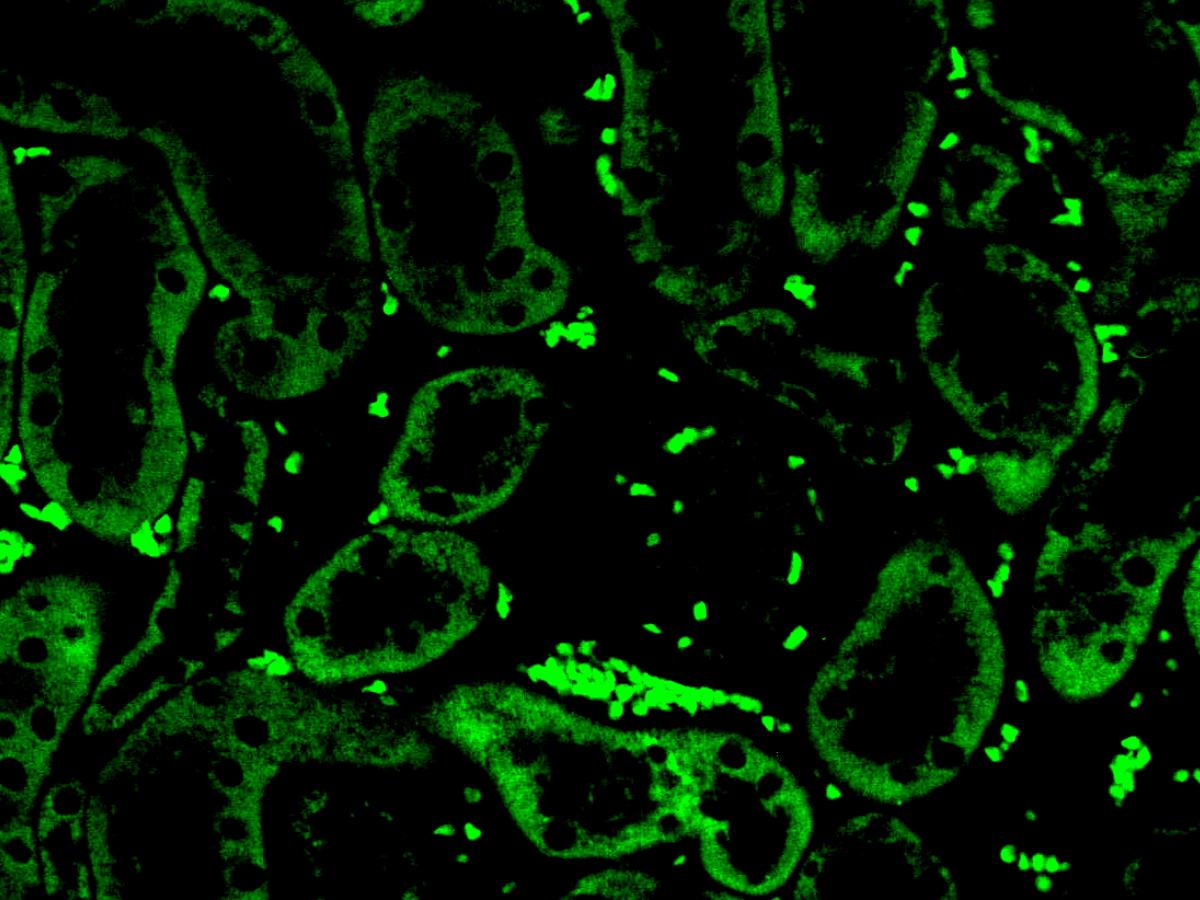

Supplement: Supplementary file 7 [file DataSheet5.zip › original images of figure 6/图6E-2-1-1(Pre-FITC).jpg]

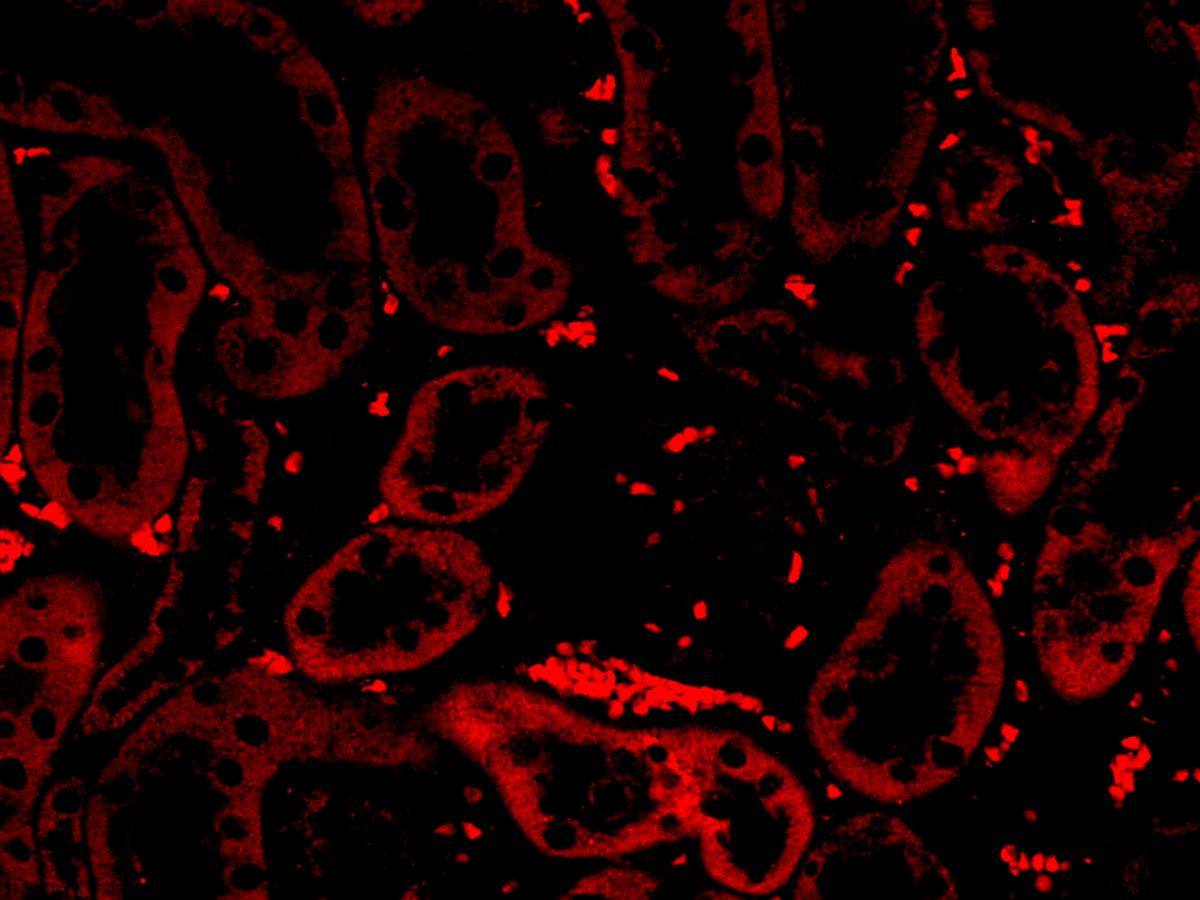

Supplement: Supplementary file 7 [file DataSheet5.zip › original images of figure 6/图6E-2-1-2(Pre-TRITC).jpg]

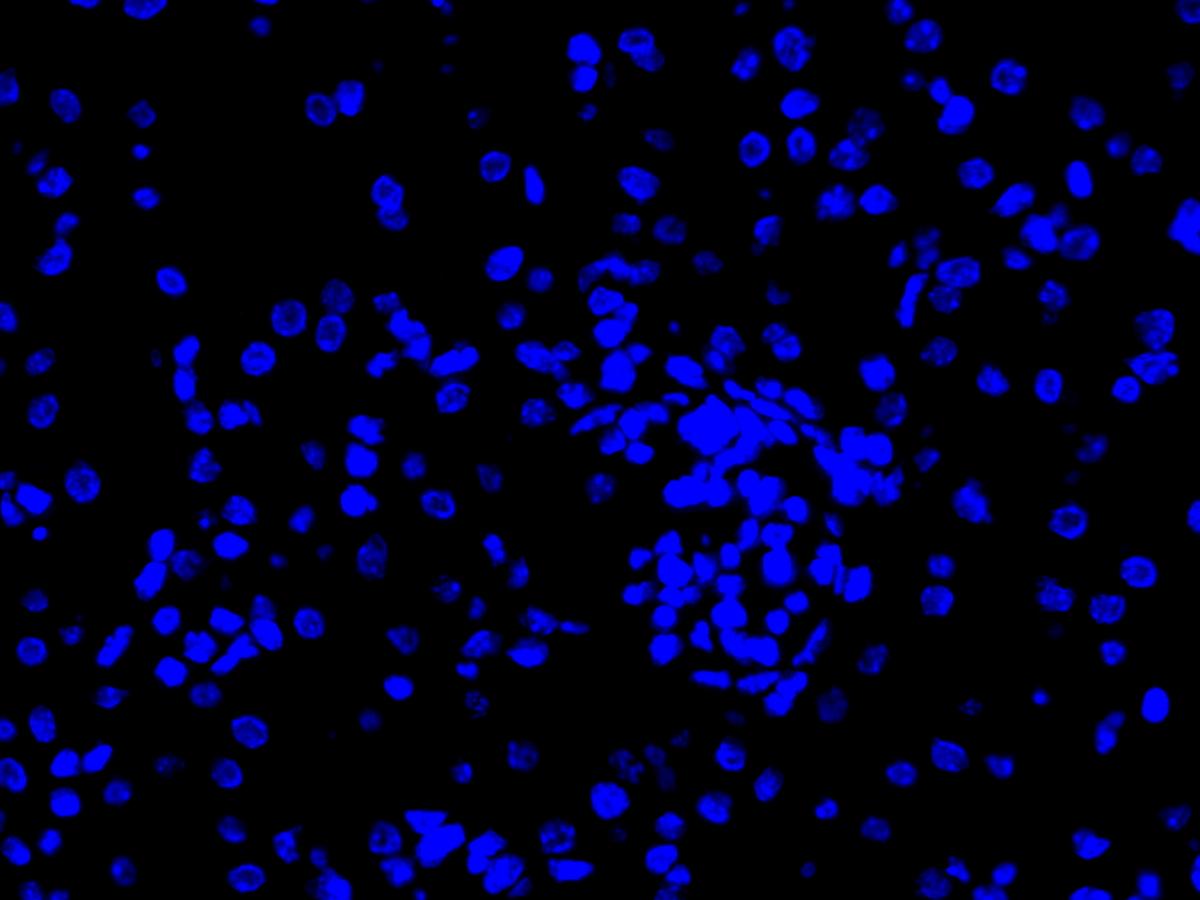

Supplement: Supplementary file 7 [file DataSheet5.zip › original images of figure 6/图6E-2-1-3(Pre-DAPI).jpg]

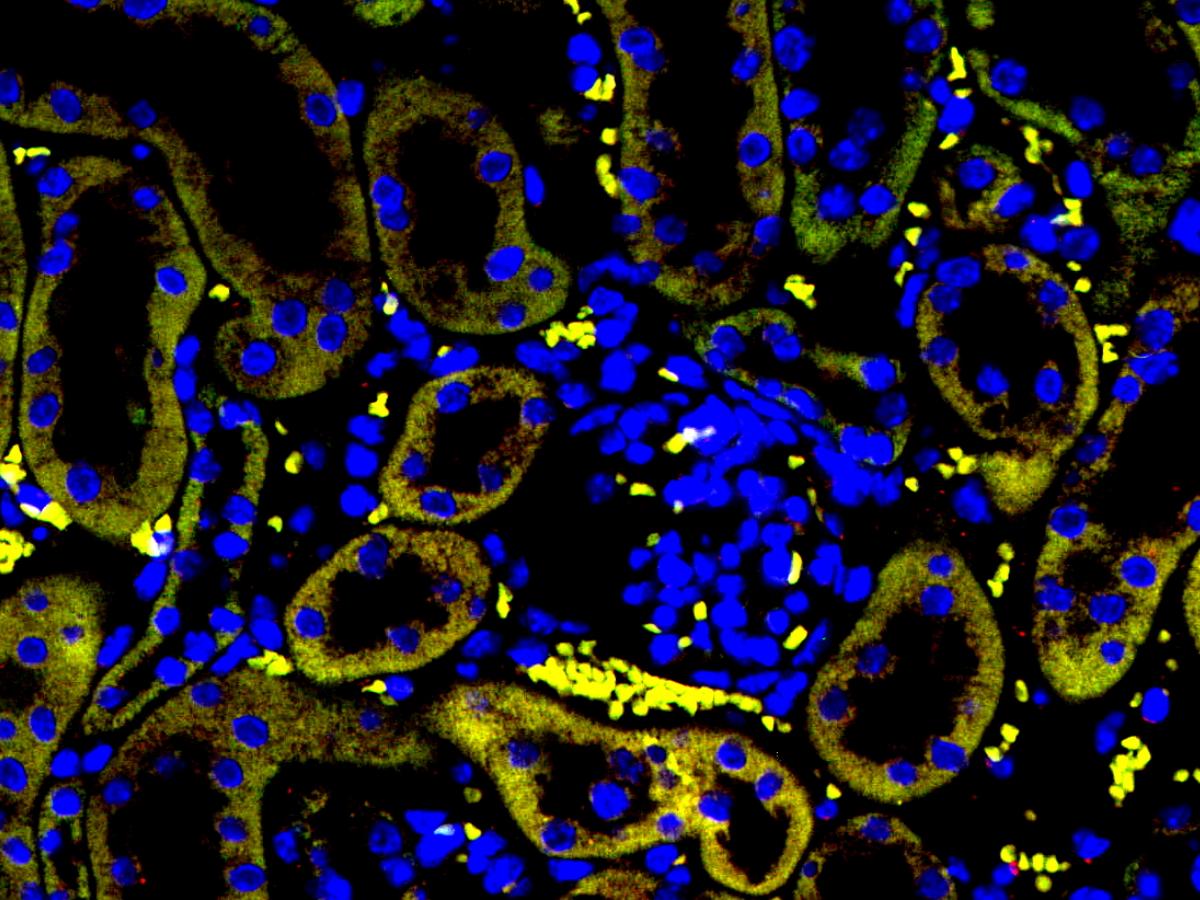

Supplement: Supplementary file 7 [file DataSheet5.zip › original images of figure 6/图6E-2-1-4(Pre-Merge).jpg]

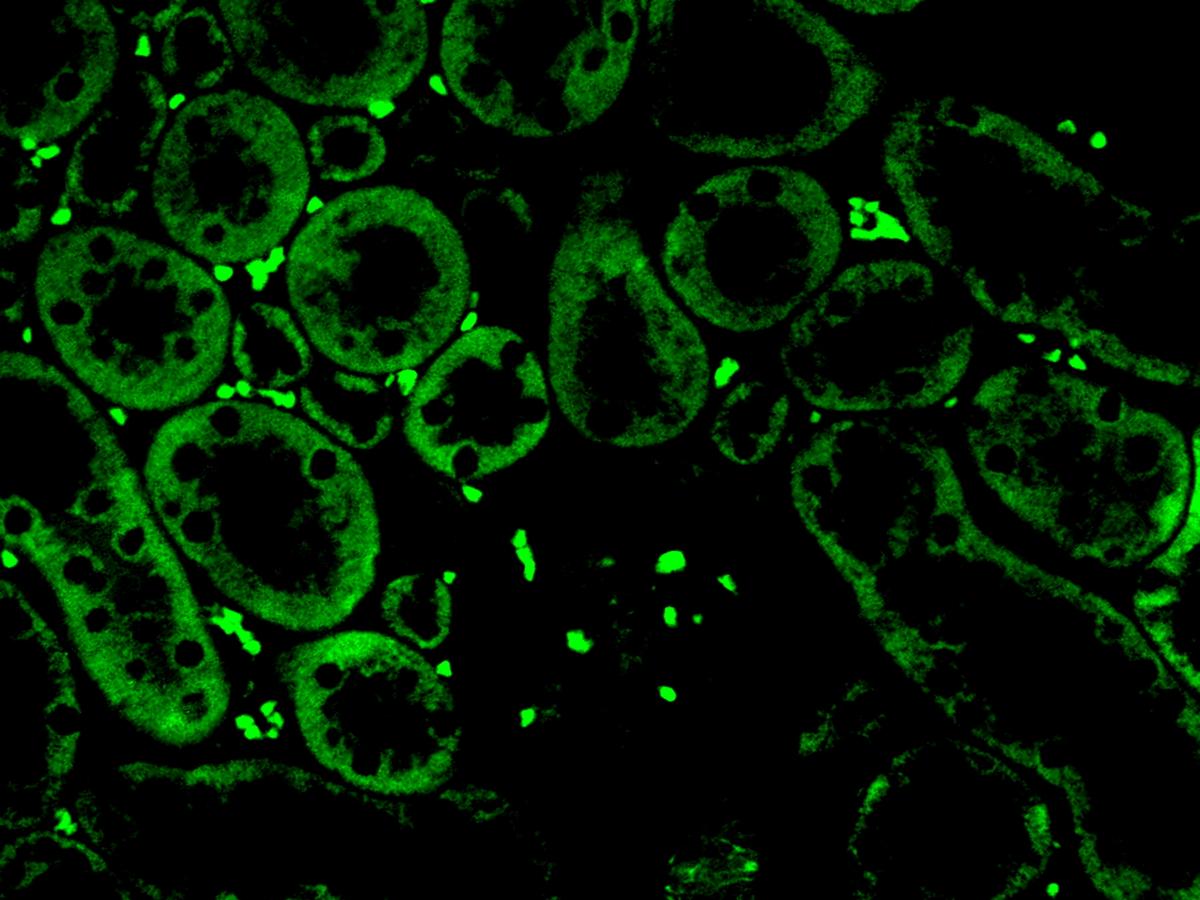

Supplement: Supplementary file 7 [file DataSheet5.zip › original images of figure 6/图6E-2-2-1(4h-FITC).jpg]

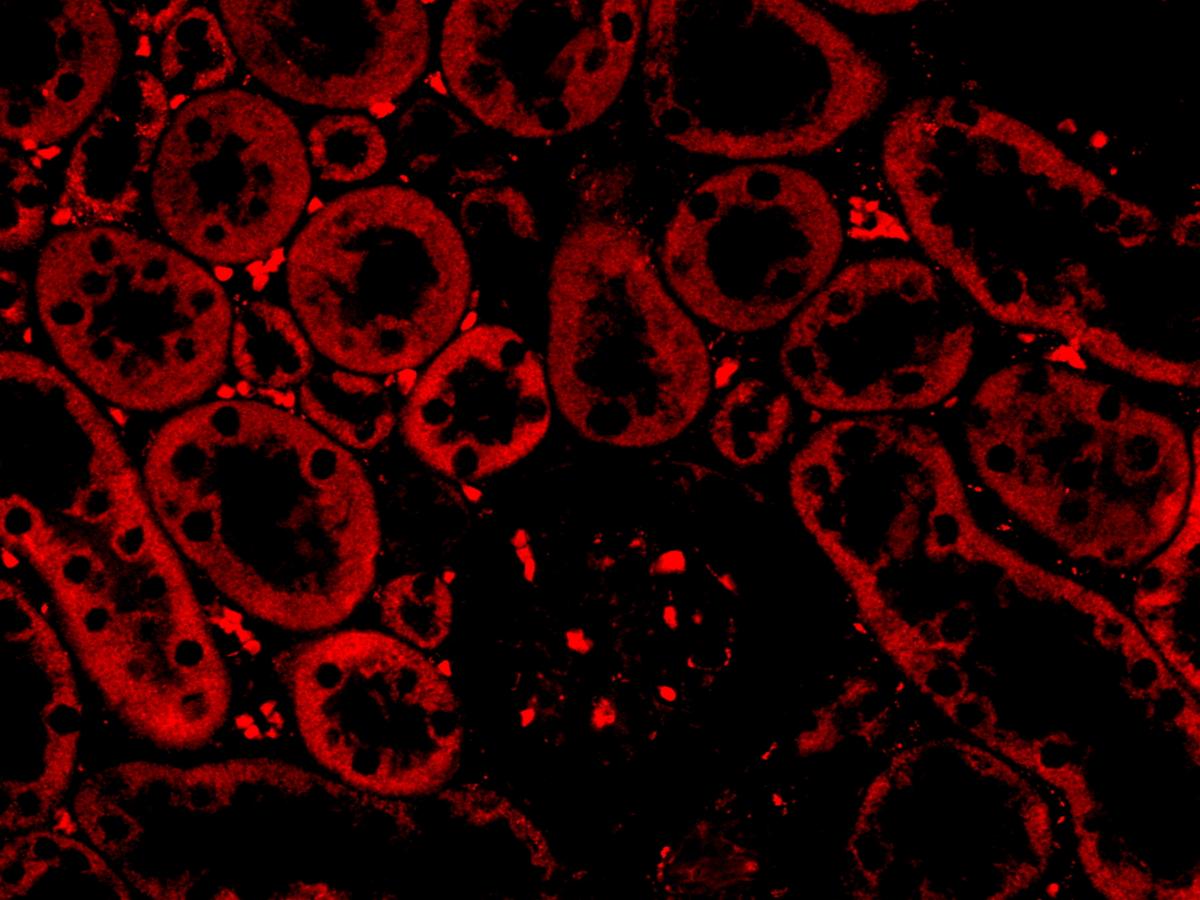

Supplement: Supplementary file 7 [file DataSheet5.zip › original images of figure 6/图6E-2-2-2(4h-TRITC).jpg]

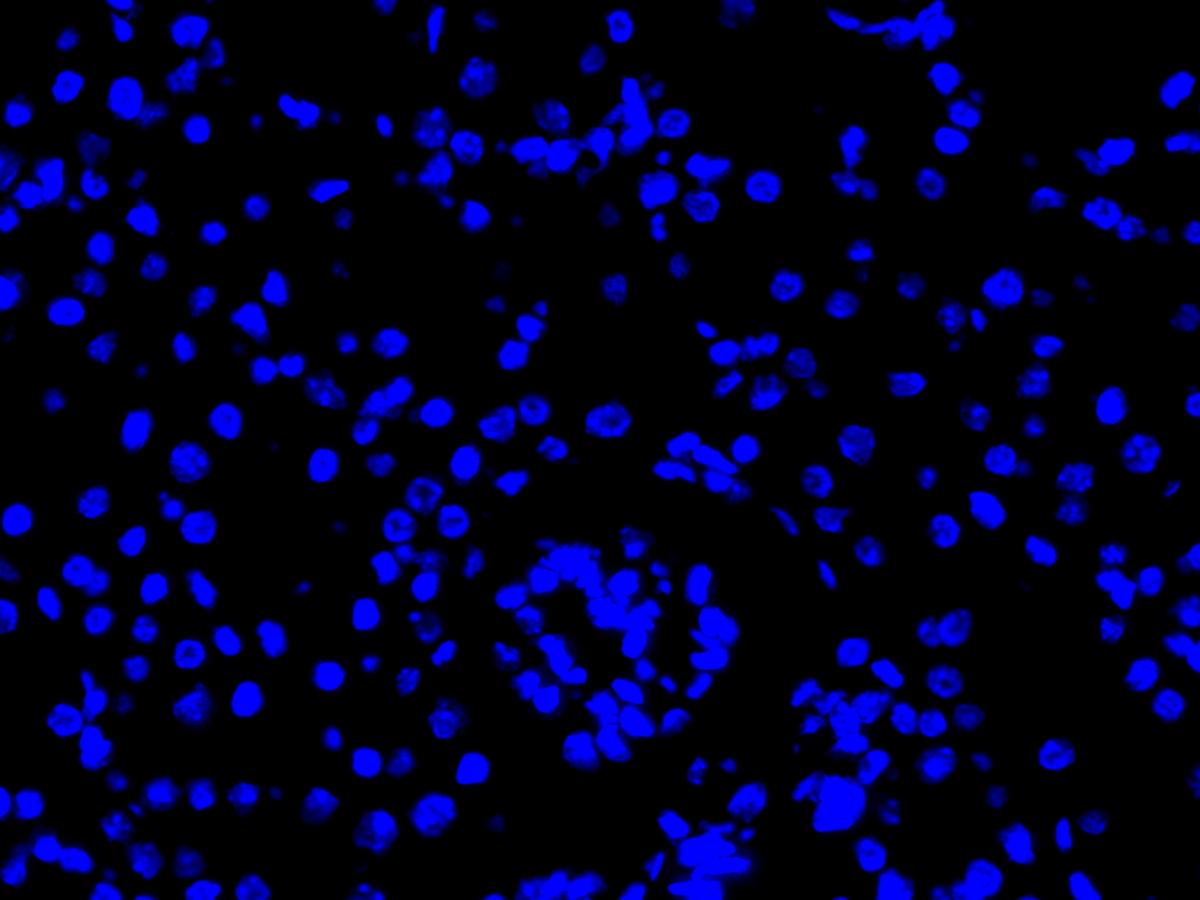

Supplement: Supplementary file 7 [file DataSheet5.zip › original images of figure 6/图6E-2-2-3(4h-DAPI).jpg]

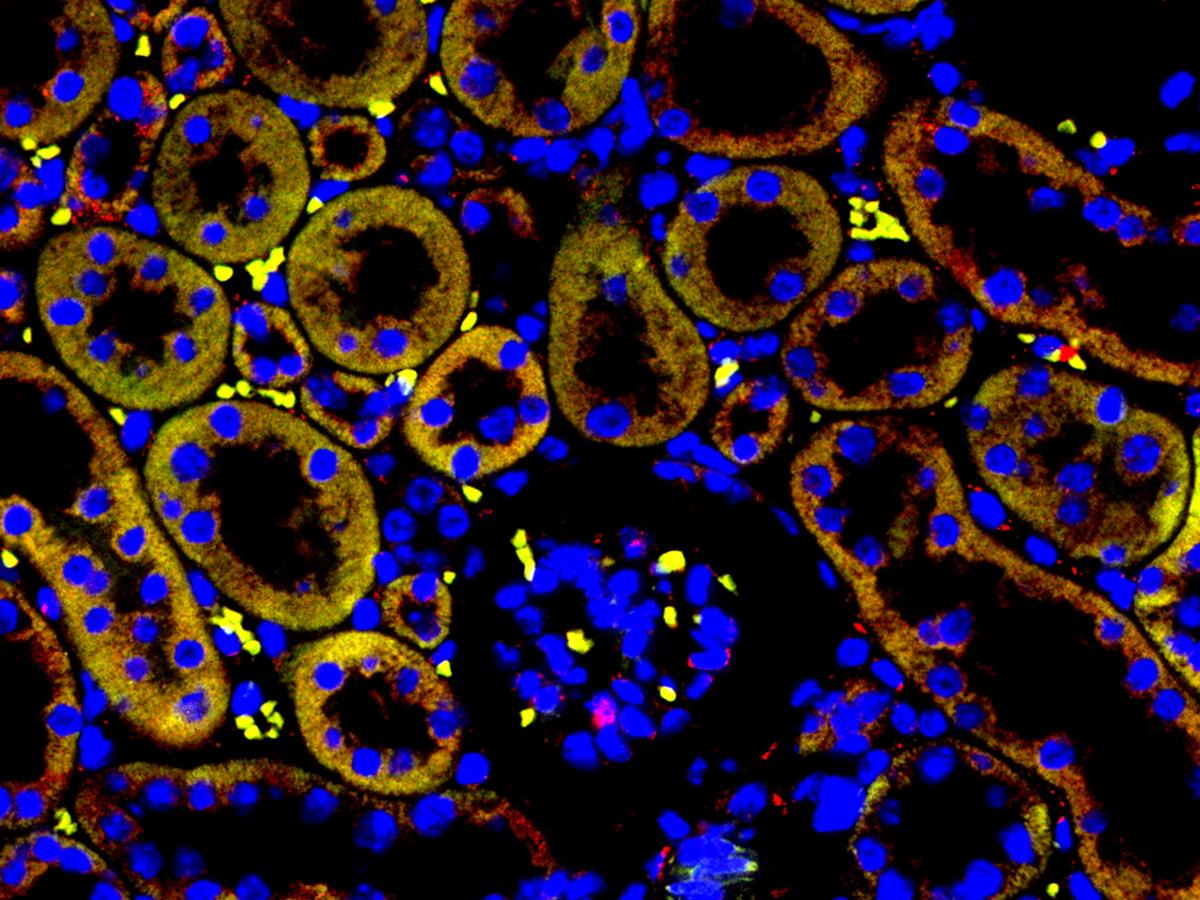

Supplement: Supplementary file 7 [file DataSheet5.zip › original images of figure 6/图6E-2-2-4(4h-Merge).jpg]

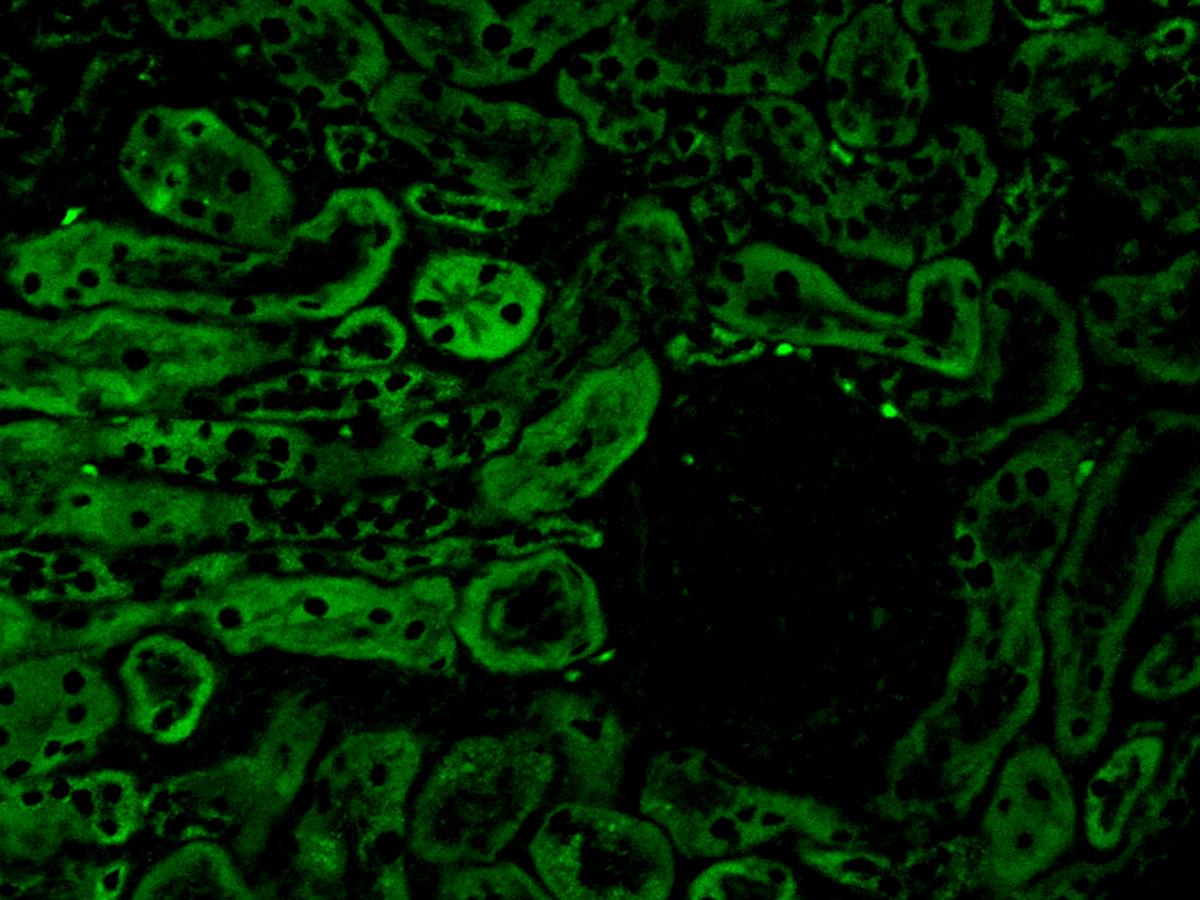

Supplement: Supplementary file 7 [file DataSheet5.zip › original images of figure 6/图6E-2-3-1(8h-FITC).jpg]

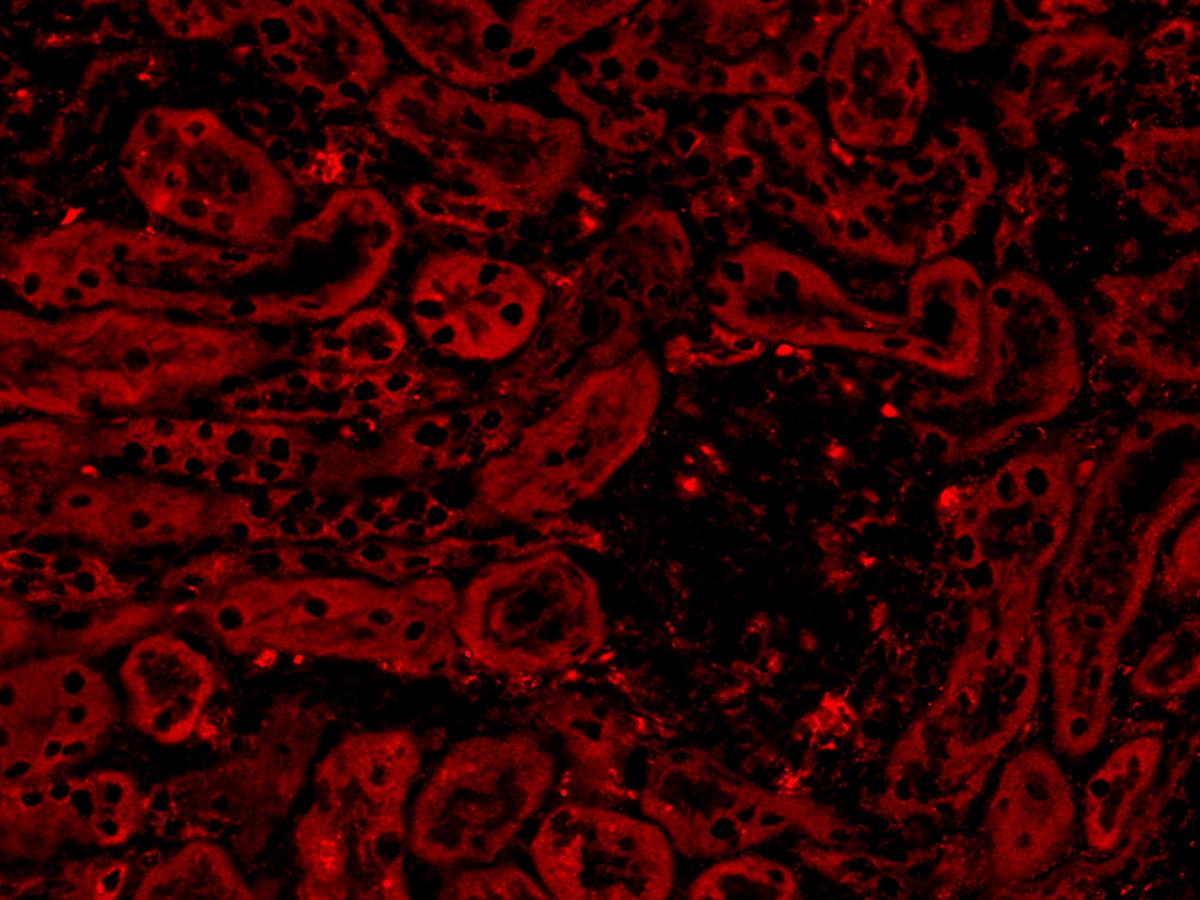

Supplement: Supplementary file 7 [file DataSheet5.zip › original images of figure 6/图6E-2-3-2(8h-TRITC).jpg]

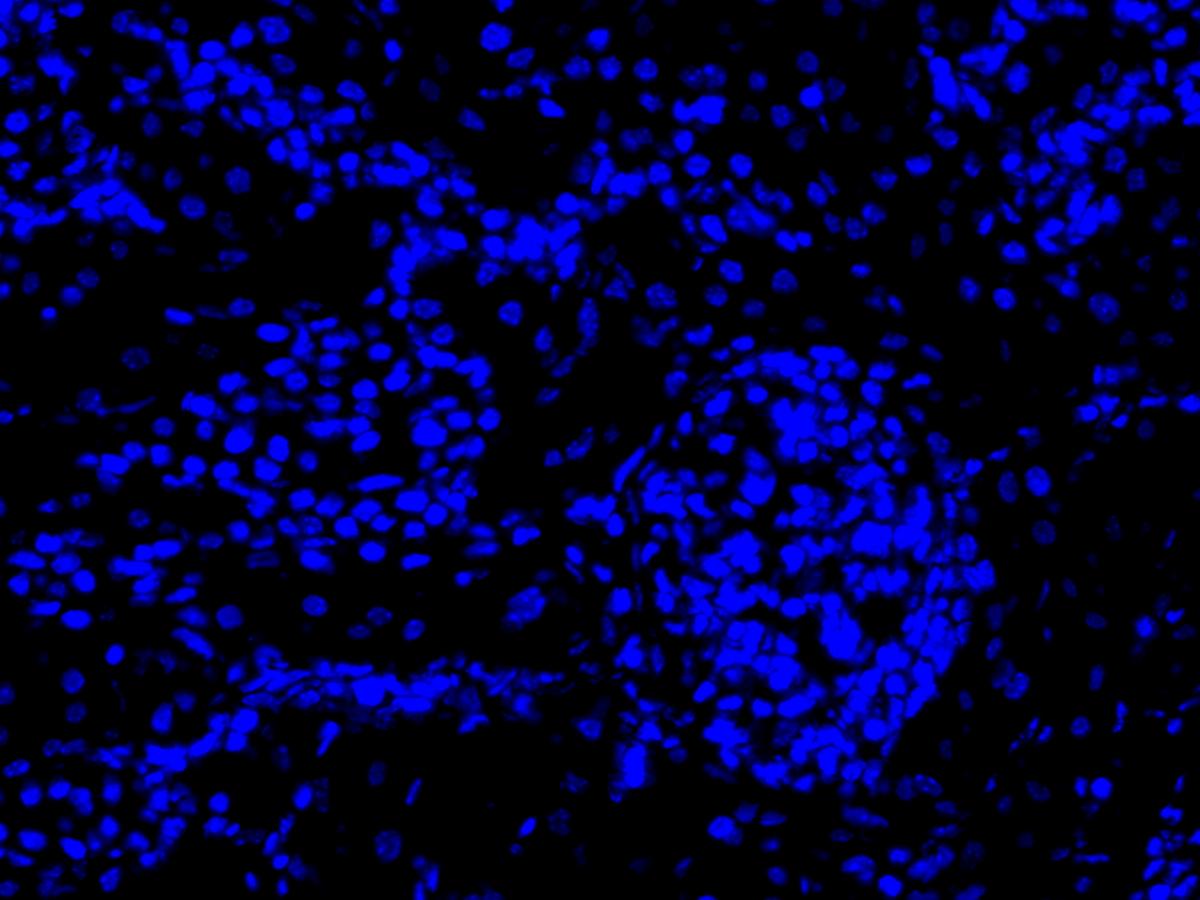

Supplement: Supplementary file 7 [file DataSheet5.zip › original images of figure 6/图6E-2-3-3(8h-DAPI).jpg]

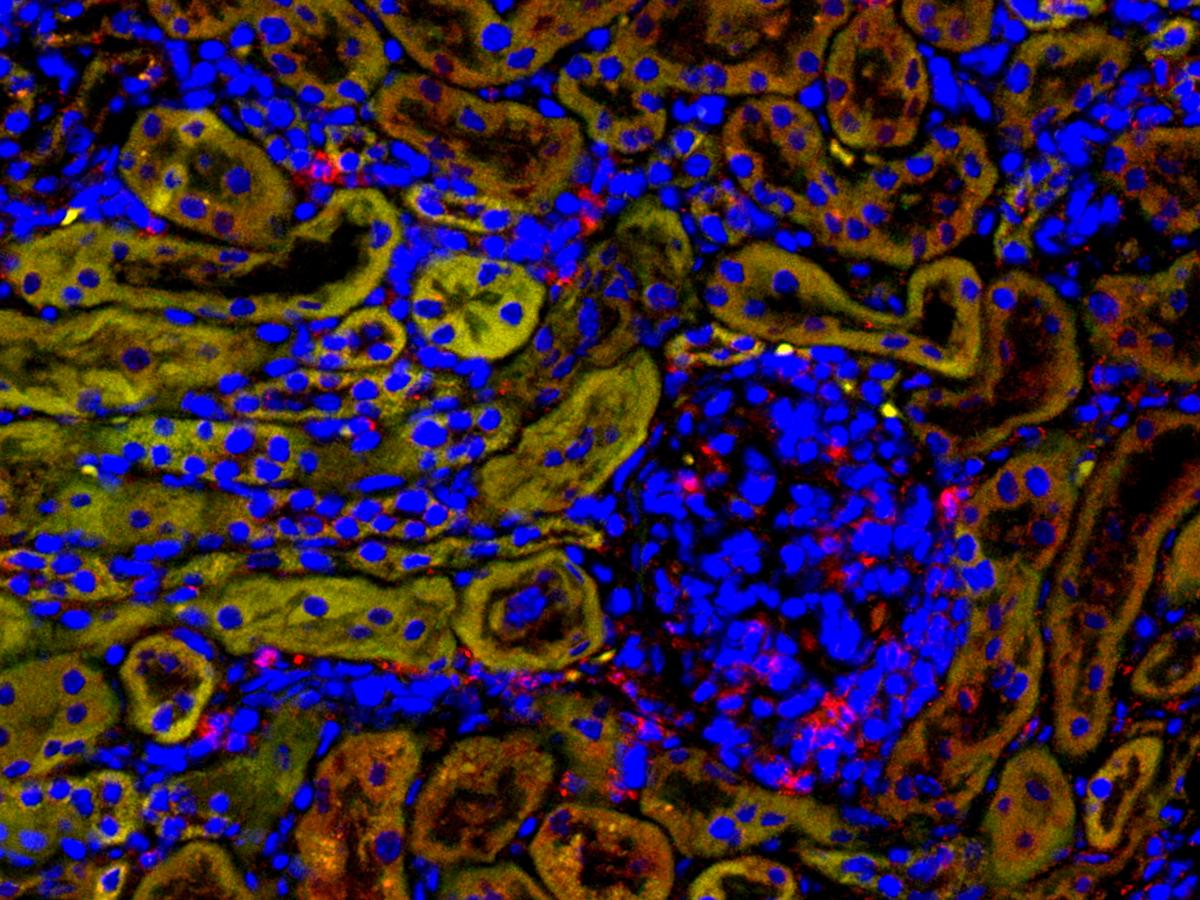

Supplement: Supplementary file 7 [file DataSheet5.zip › original images of figure 6/图6E-2-3-4(8h-Merge).jpg]

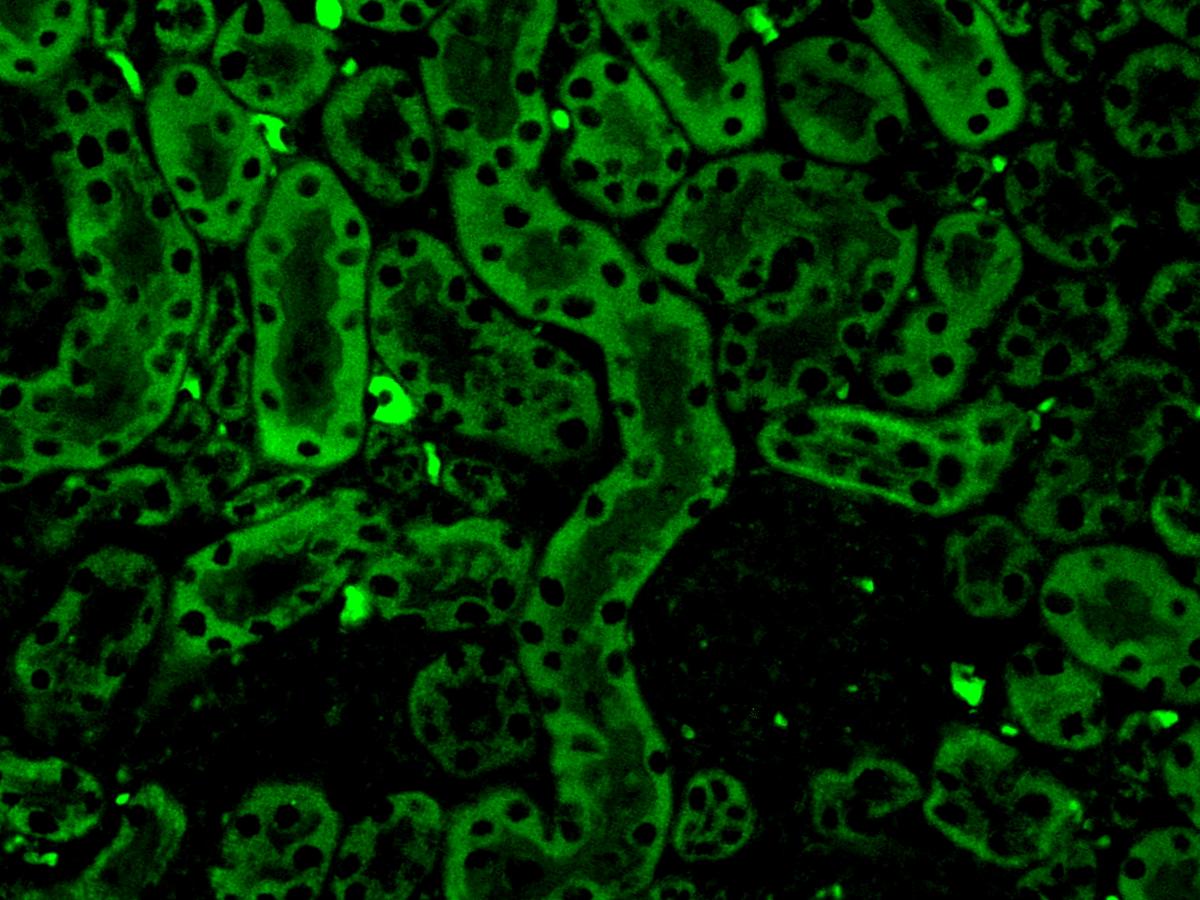

Supplement: Supplementary file 7 [file DataSheet5.zip › original images of figure 6/图6E-2-4-1(16h-FITC).jpg]

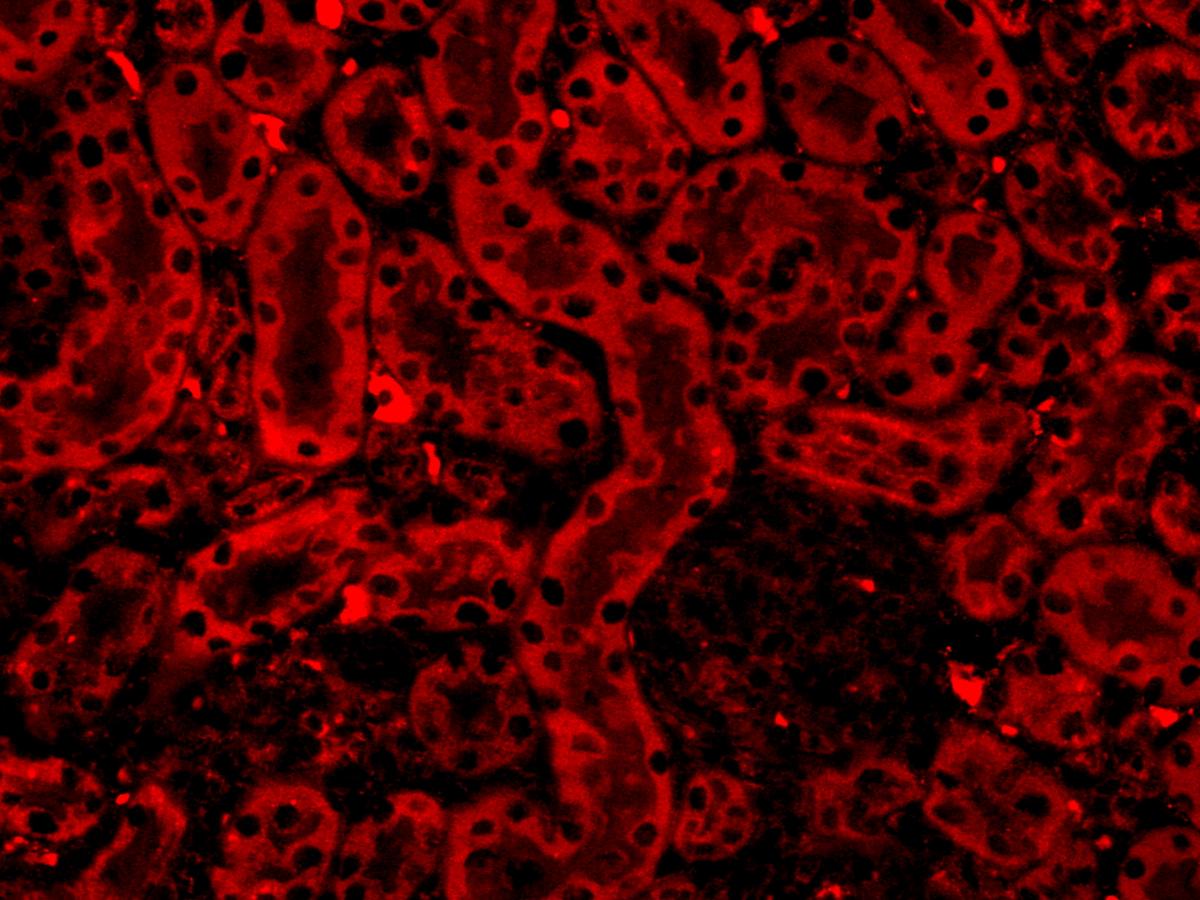

Supplement: Supplementary file 7 [file DataSheet5.zip › original images of figure 6/图6E-2-4-2(16h-TRITC).jpg]

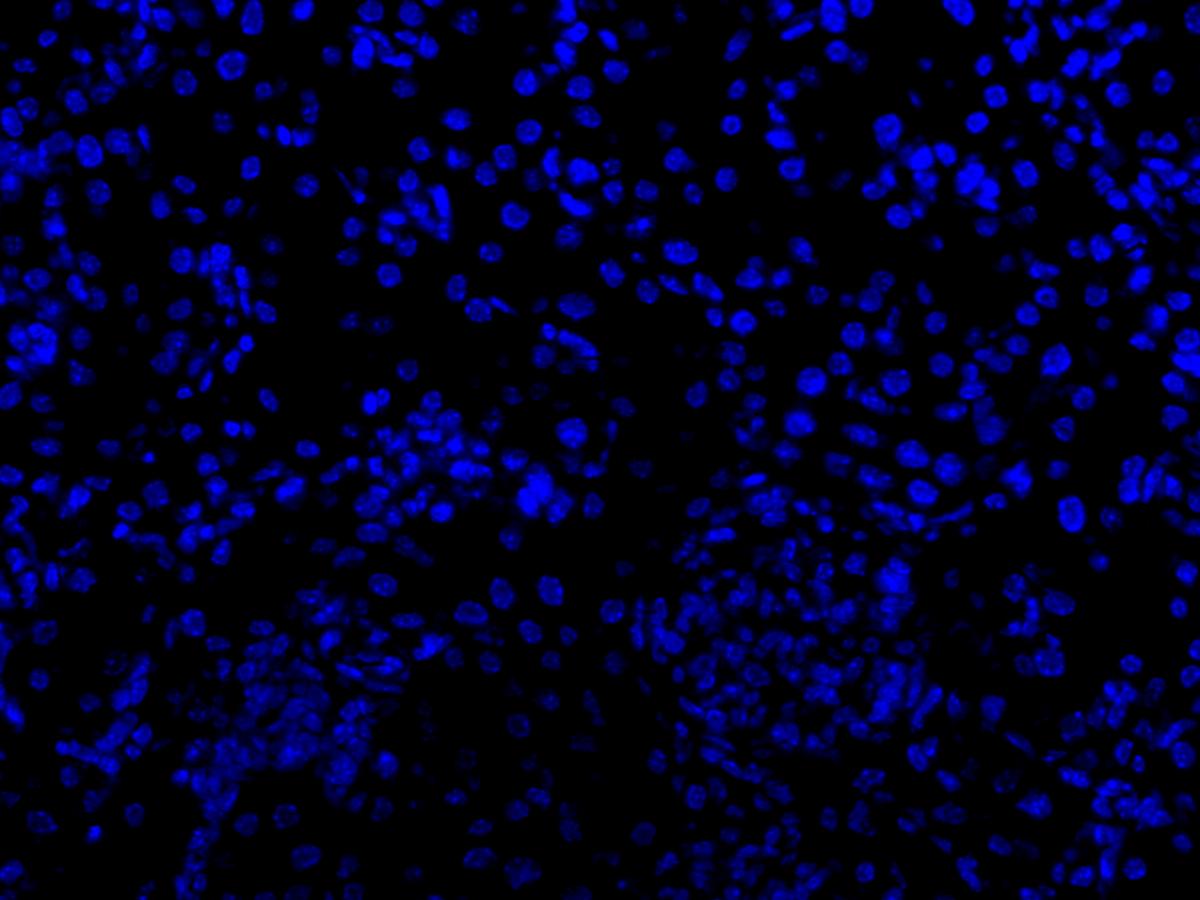

Supplement: Supplementary file 7 [file DataSheet5.zip › original images of figure 6/图6E-2-4-3(16h-DAPI).jpg]

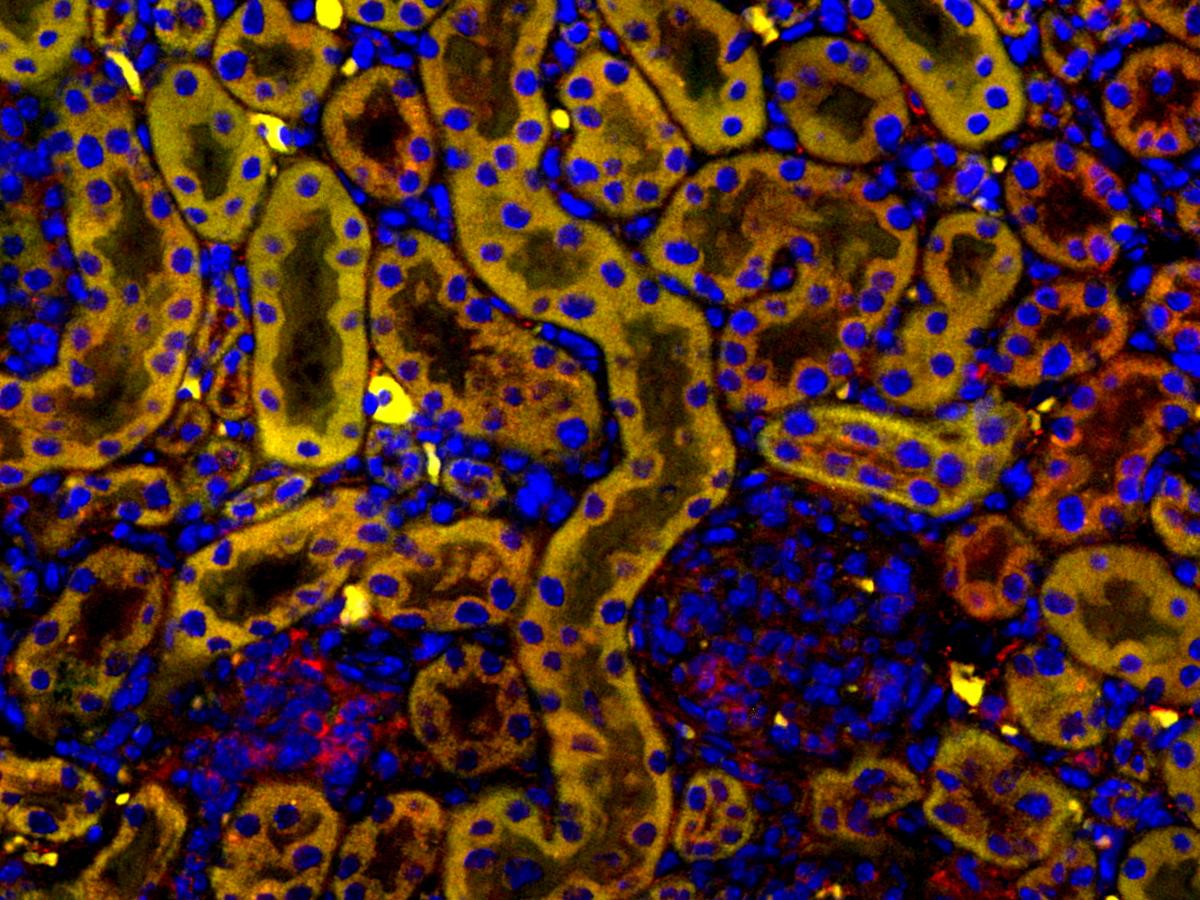

Supplement: Supplementary file 7 [file DataSheet5.zip › original images of figure 6/图6E-2-4-4(16h-Merge).jpg]

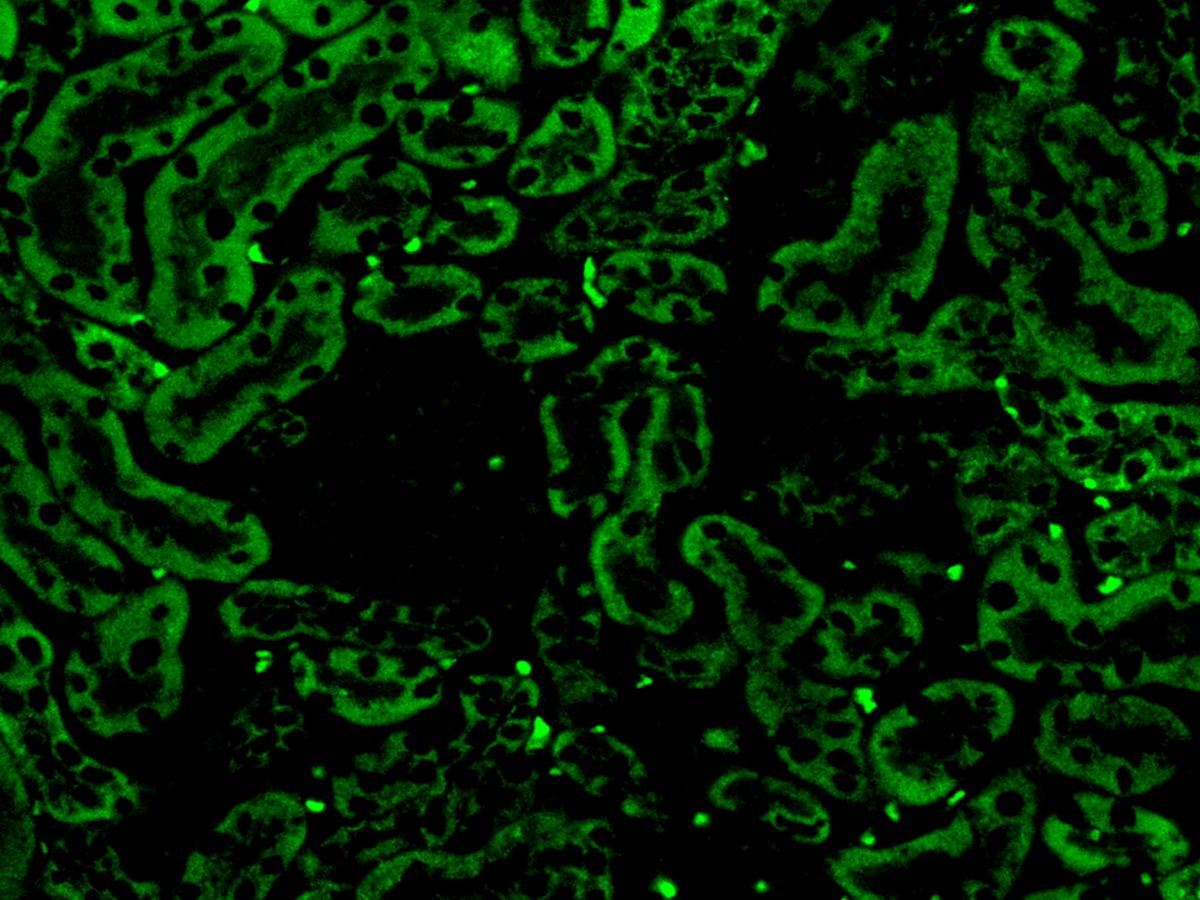

Supplement: Supplementary file 7 [file DataSheet5.zip › original images of figure 6/图6E-2-5-1(24h-FITC).jpg]

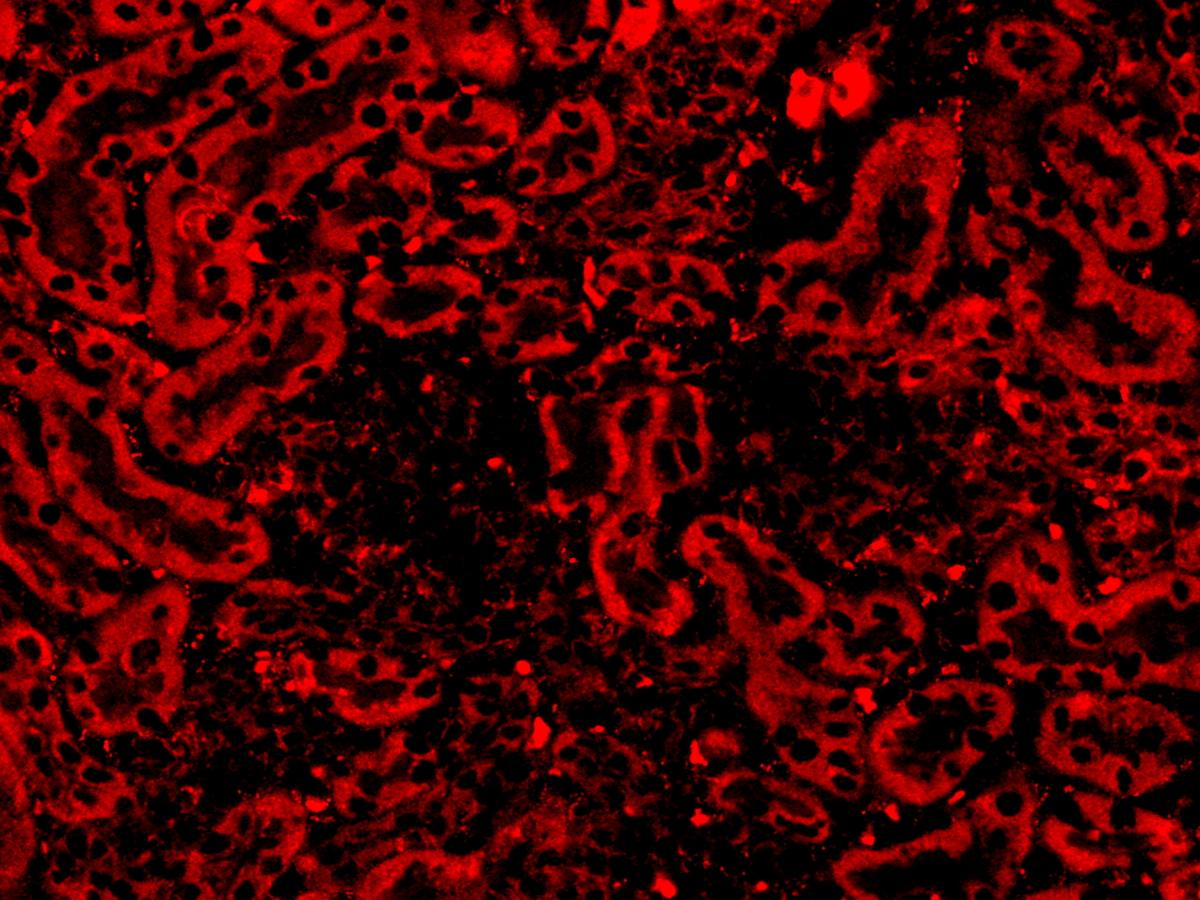

Supplement: Supplementary file 7 [file DataSheet5.zip › original images of figure 6/图6E-2-5-2(24h-TRITC).jpg]

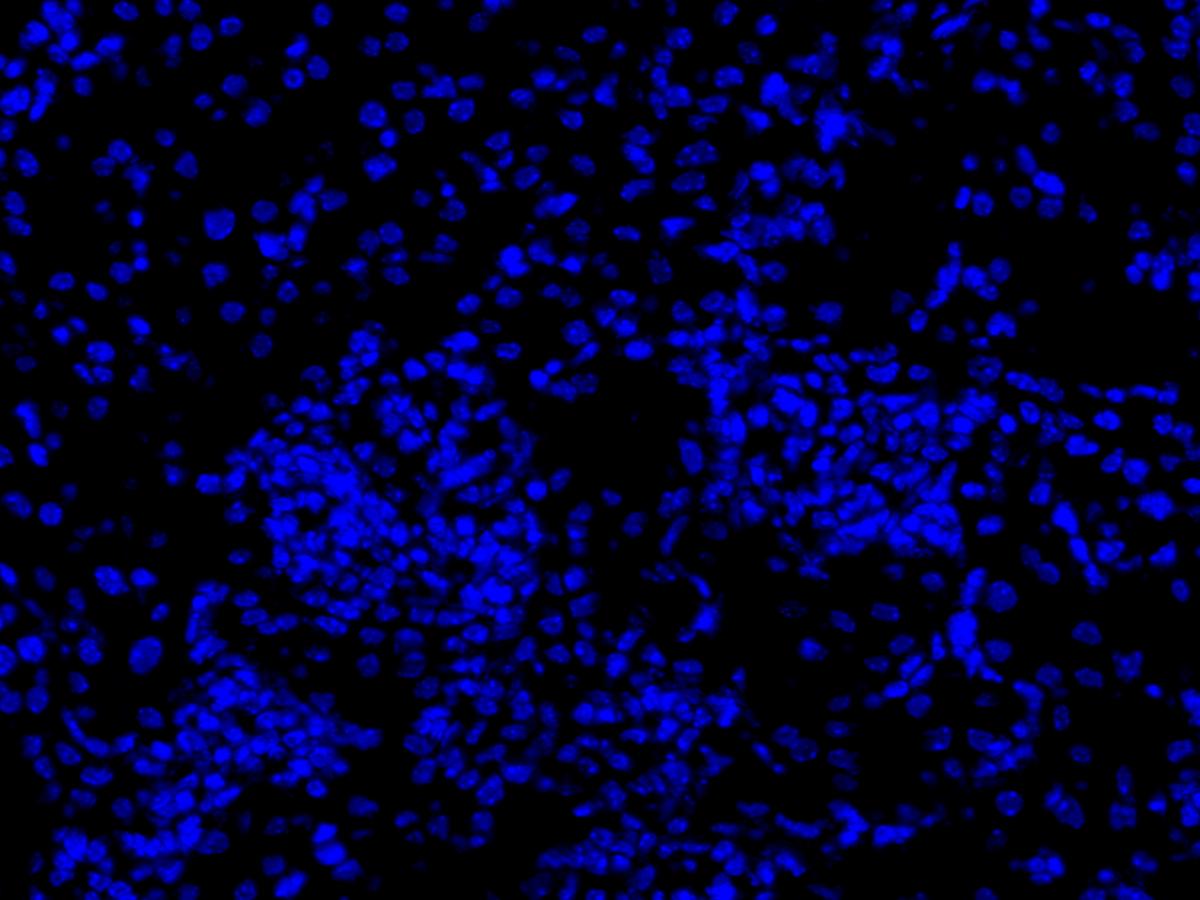

Supplement: Supplementary file 7 [file DataSheet5.zip › original images of figure 6/图6E-2-5-3(24h-DAPI).jpg]

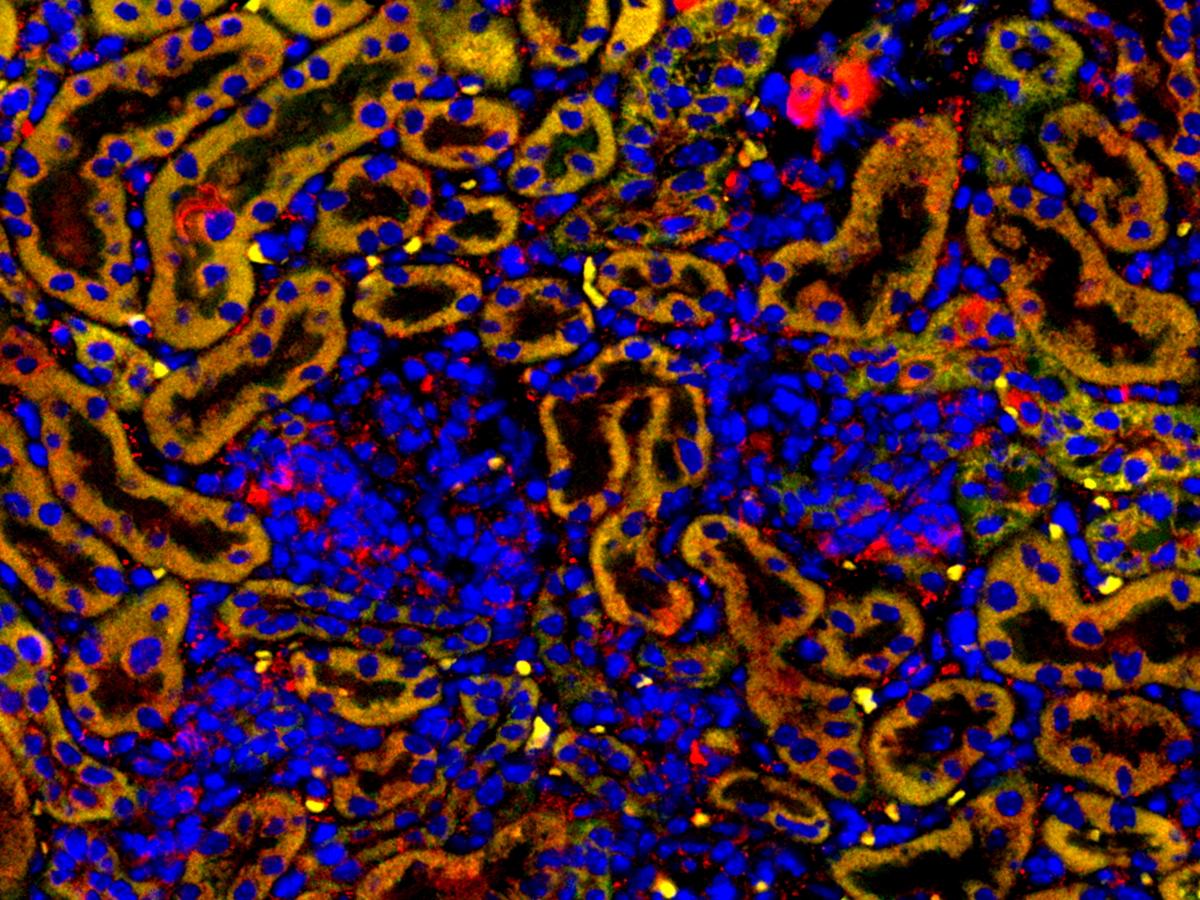

Supplement: Supplementary file 7 [file DataSheet5.zip › original images of figure 6/图6E-2-5-4(24h-Merge).jpg]

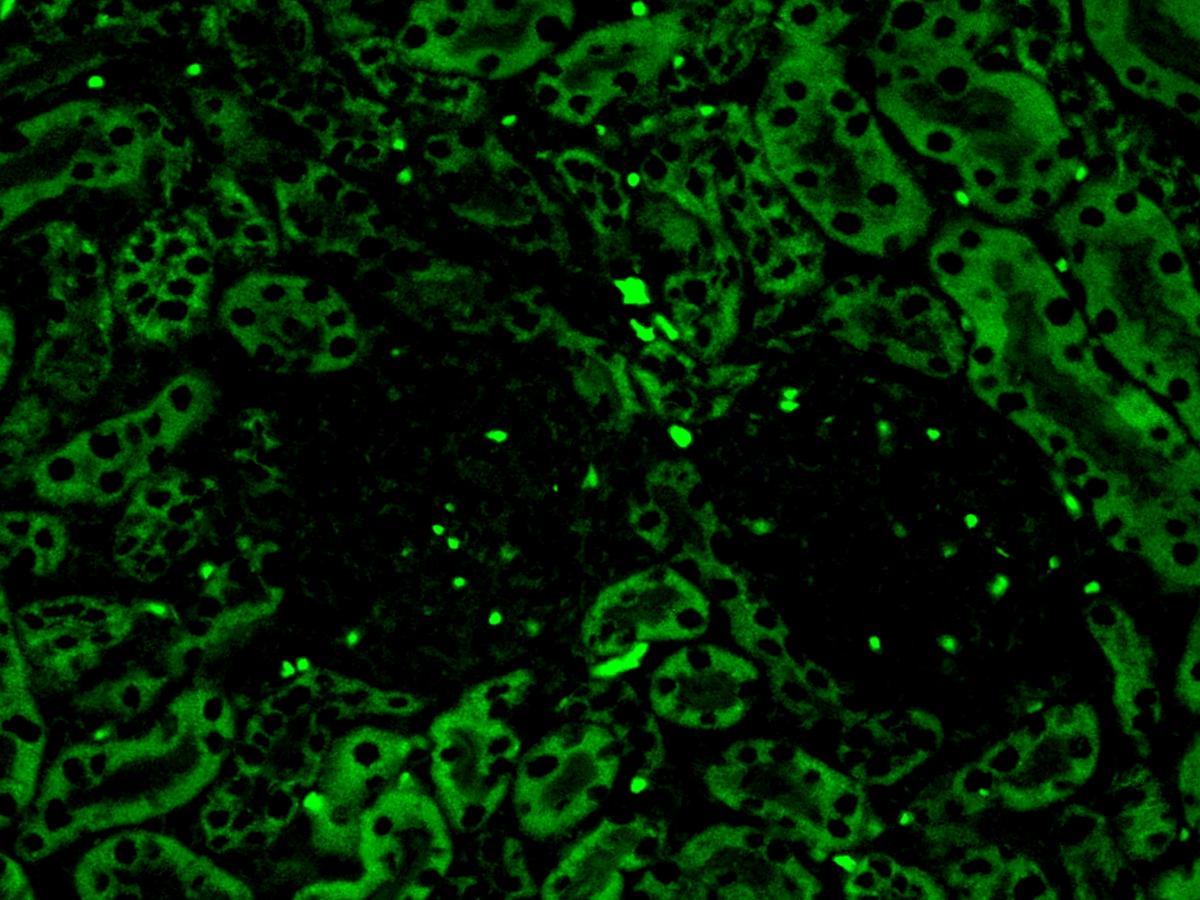

Supplement: Supplementary file 7 [file DataSheet5.zip › original images of figure 6/图6E-2-6-1(48h-FITC).jpg]

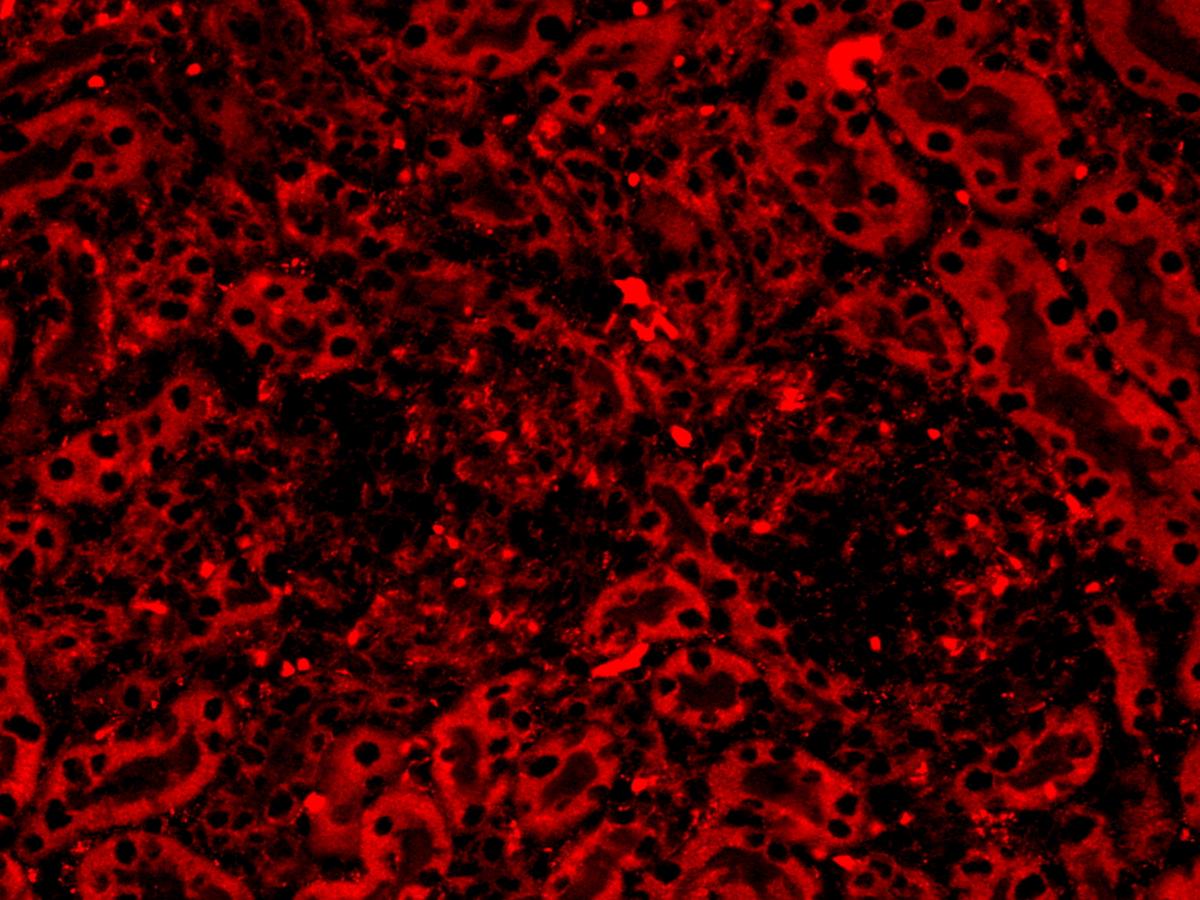

Supplement: Supplementary file 7 [file DataSheet5.zip › original images of figure 6/图6E-2-6-2(48h-TRITC).jpg]

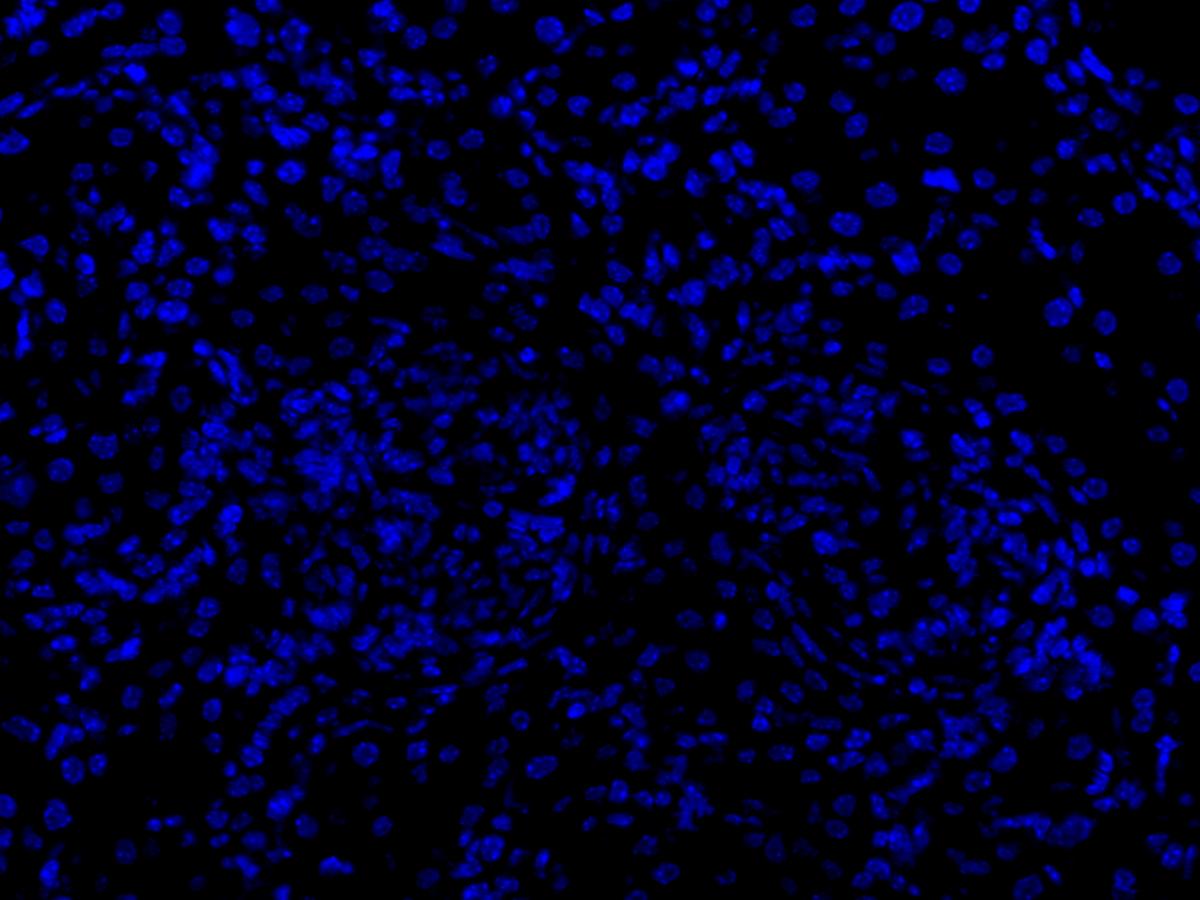

Supplement: Supplementary file 7 [file DataSheet5.zip › original images of figure 6/图6E-2-6-3(48h-DAPI).jpg]

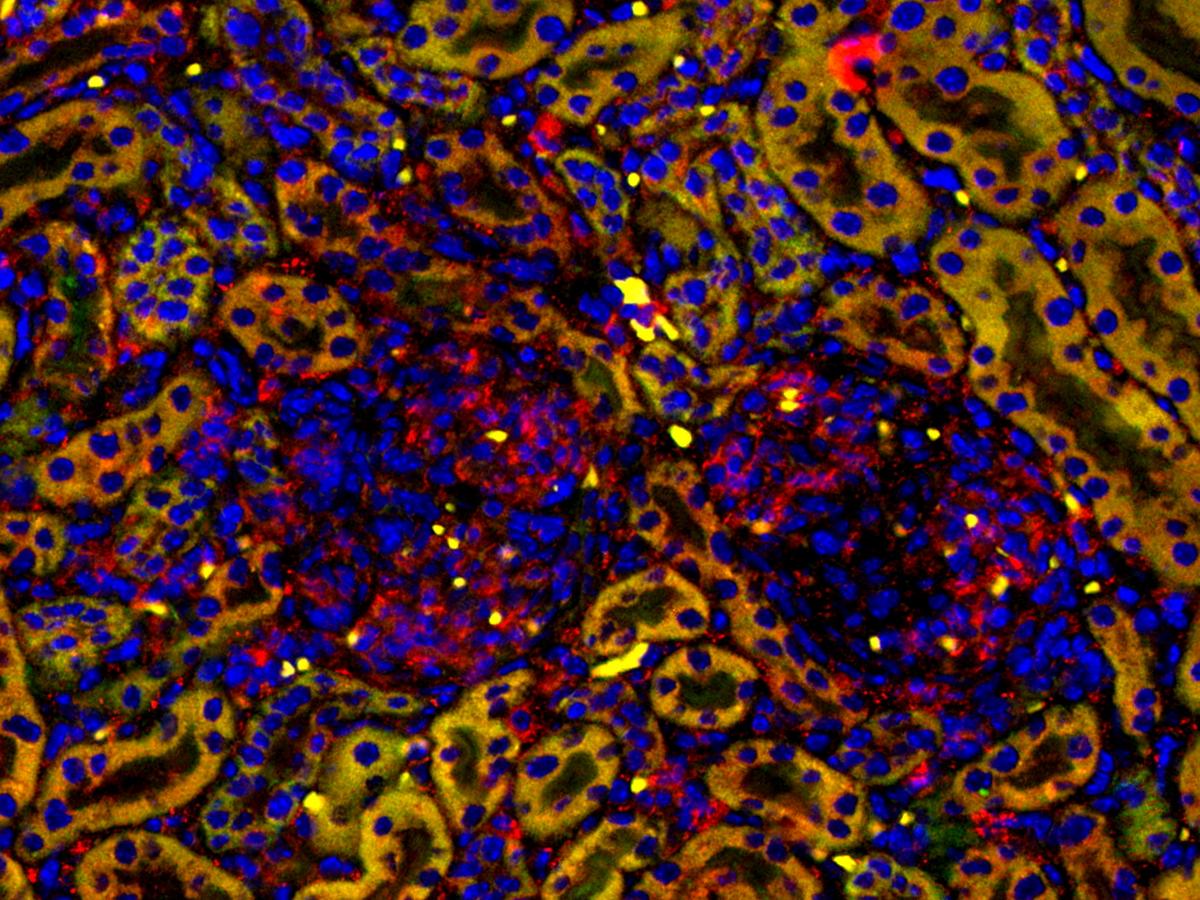

Supplement: Supplementary file 7 [file DataSheet5.zip › original images of figure 6/图6E-2-6-4(48h-Merge).jpg]

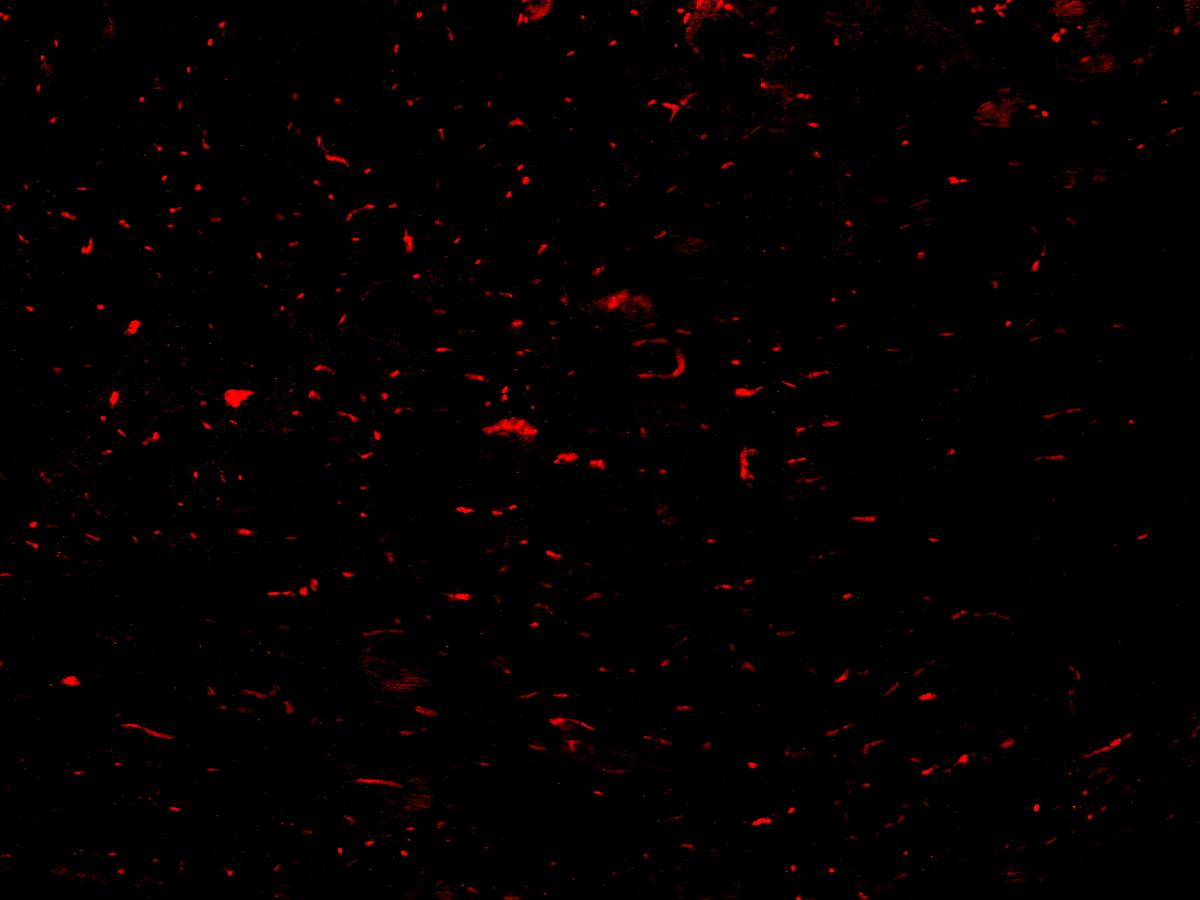

Supplement: Supplementary file 7 [file DataSheet5.zip › original images of figure 6/图6G-1-1-1(Dil).jpg]

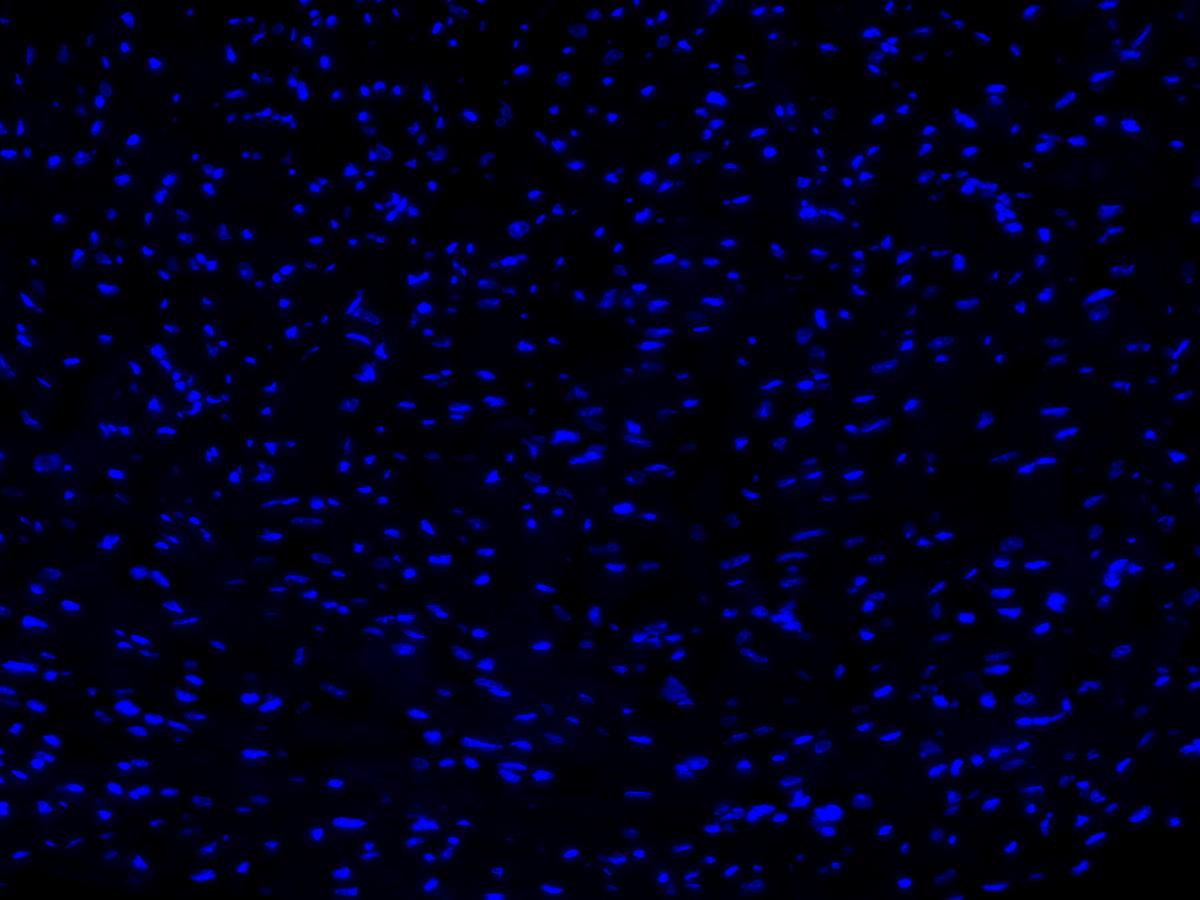

Supplement: Supplementary file 7 [file DataSheet5.zip › original images of figure 6/图6G-1-1-2(DAPI).jpg]

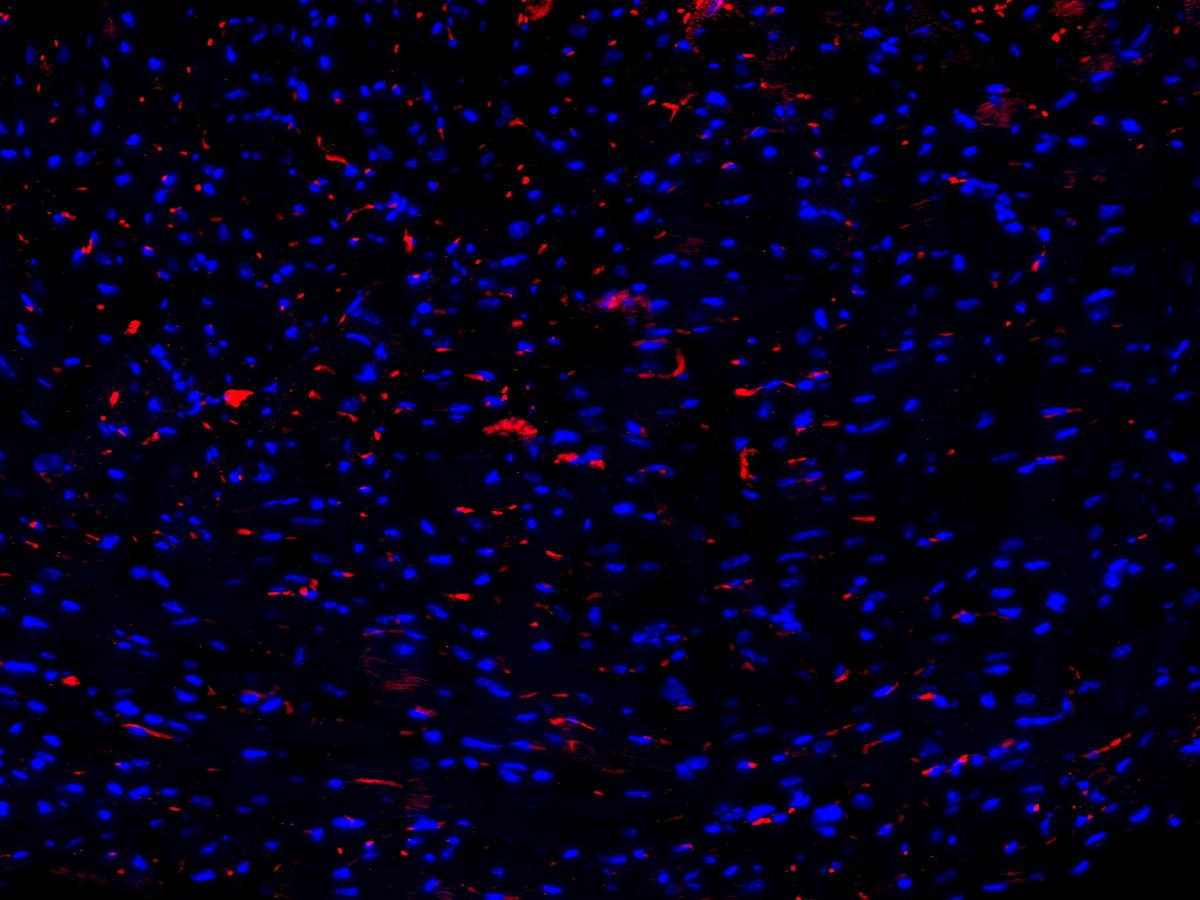

Supplement: Supplementary file 7 [file DataSheet5.zip › original images of figure 6/图6G-1-1-3(Merge).jpg]

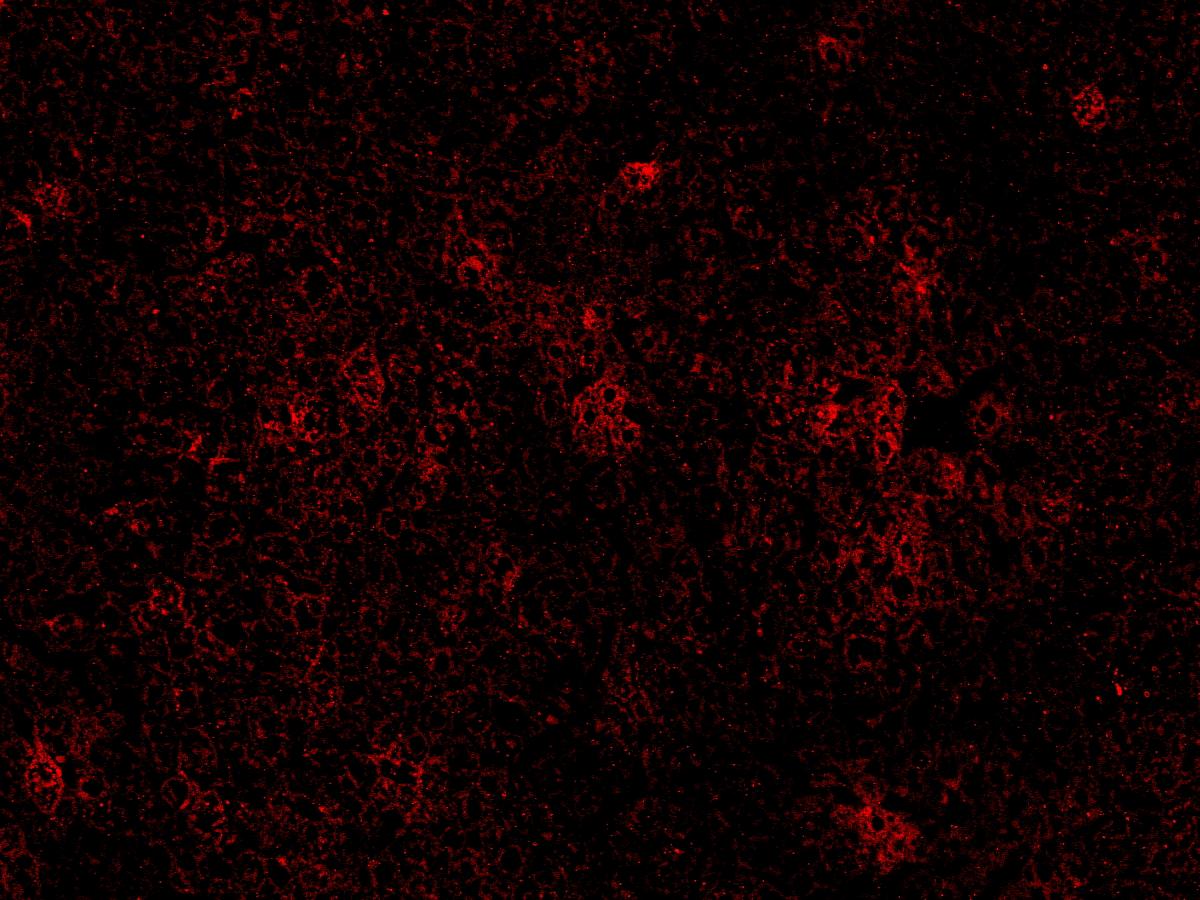

Supplement: Supplementary file 7 [file DataSheet5.zip › original images of figure 6/图6G-1-2-1(Dil).jpg]

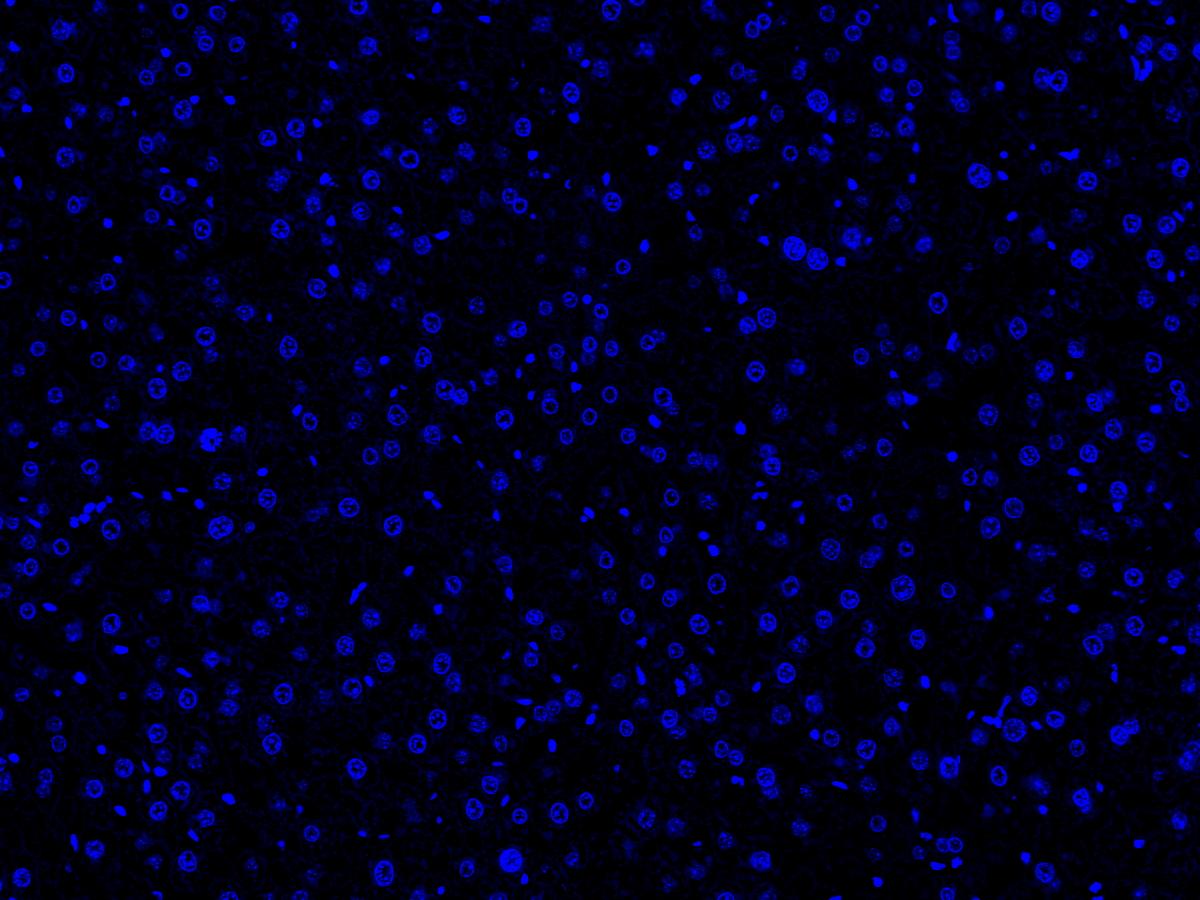

Supplement: Supplementary file 7 [file DataSheet5.zip › original images of figure 6/图6G-1-2-2(DAPI).jpg]

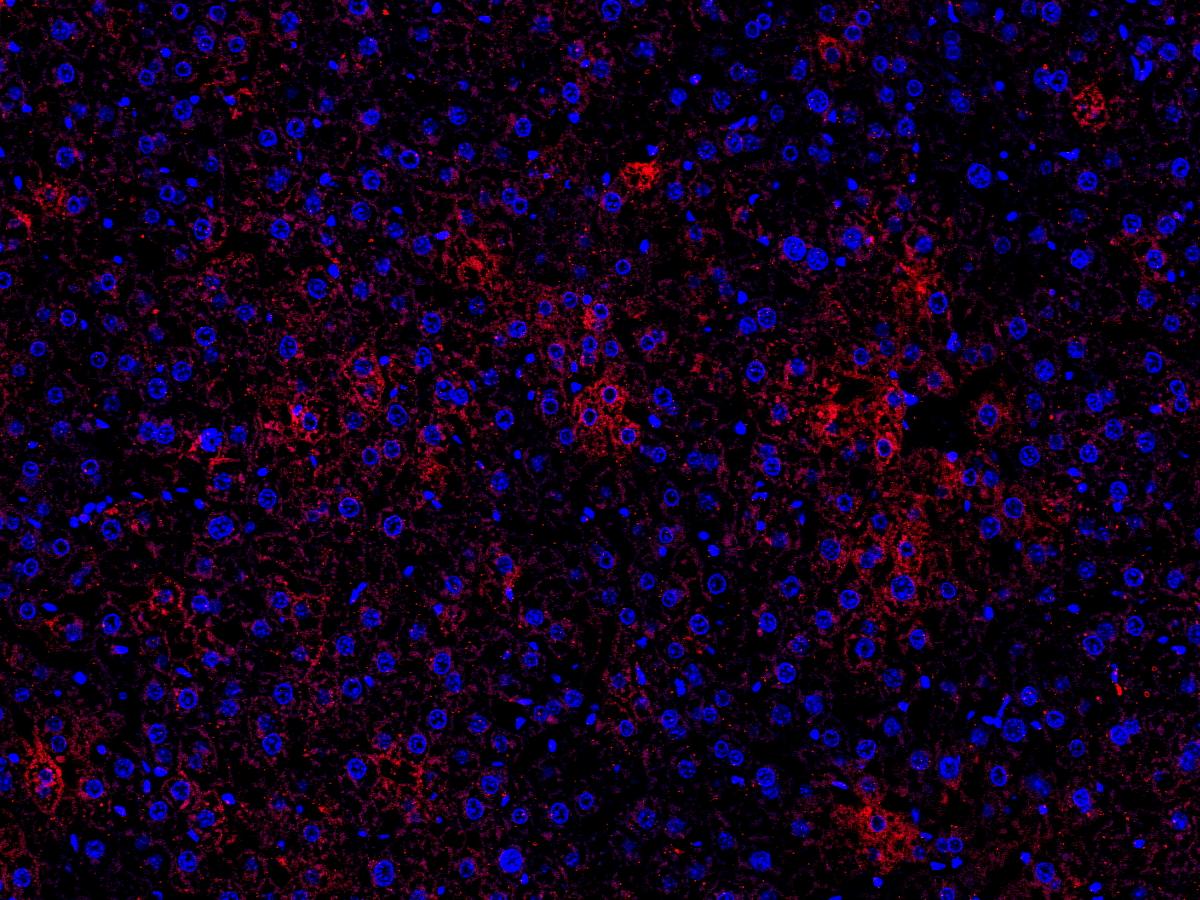

Supplement: Supplementary file 7 [file DataSheet5.zip › original images of figure 6/图6G-1-2-3(Merge).jpg]

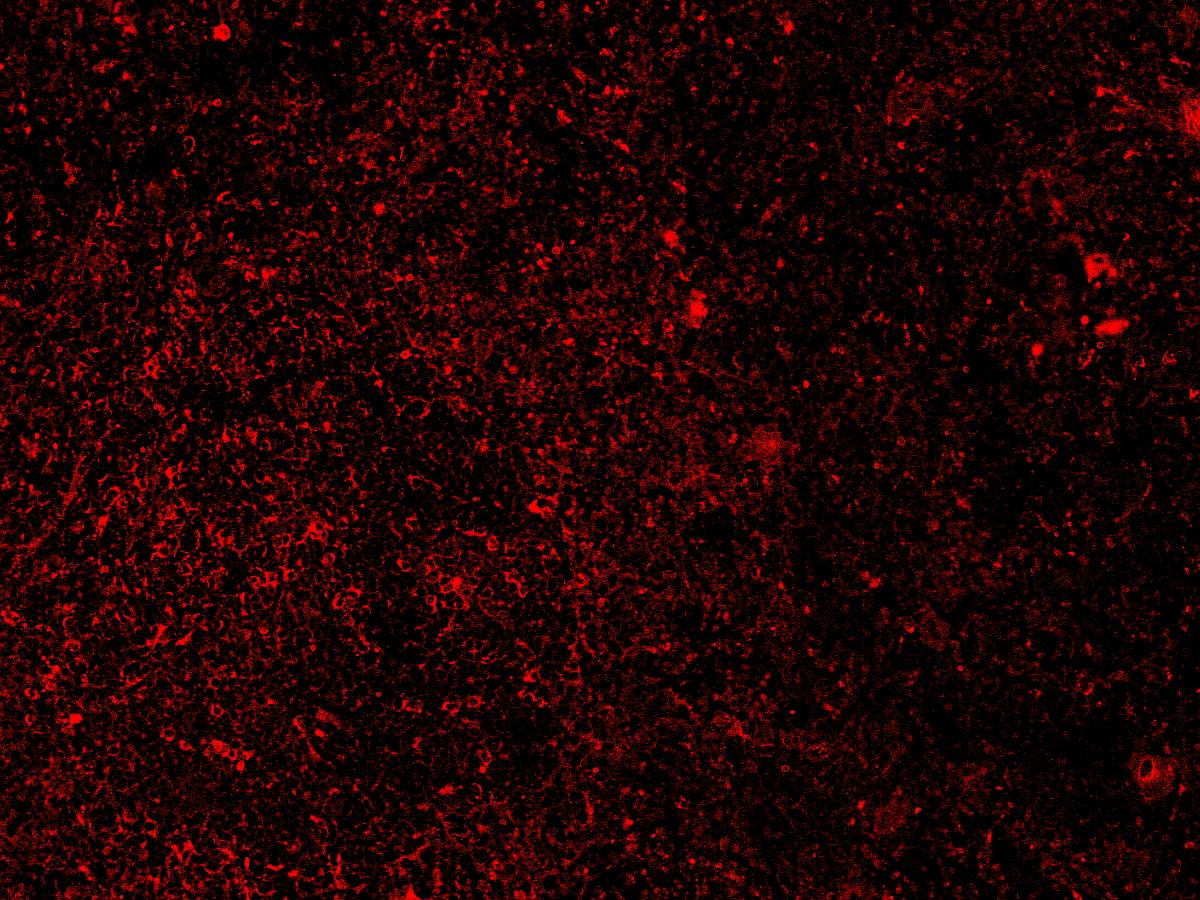

Supplement: Supplementary file 7 [file DataSheet5.zip › original images of figure 6/图6G-1-3-1(Dil).jpg]

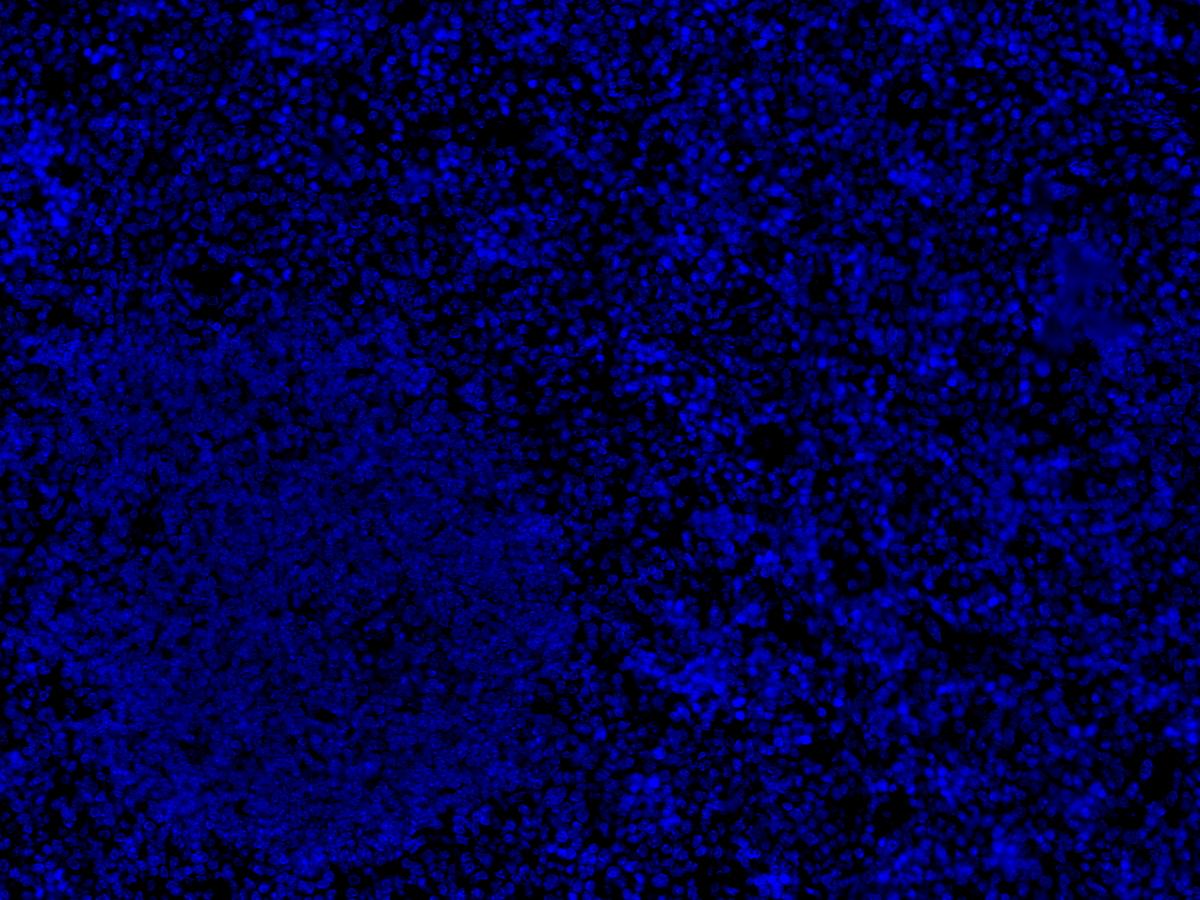

Supplement: Supplementary file 7 [file DataSheet5.zip › original images of figure 6/图6G-1-3-2(DAPI).jpg]

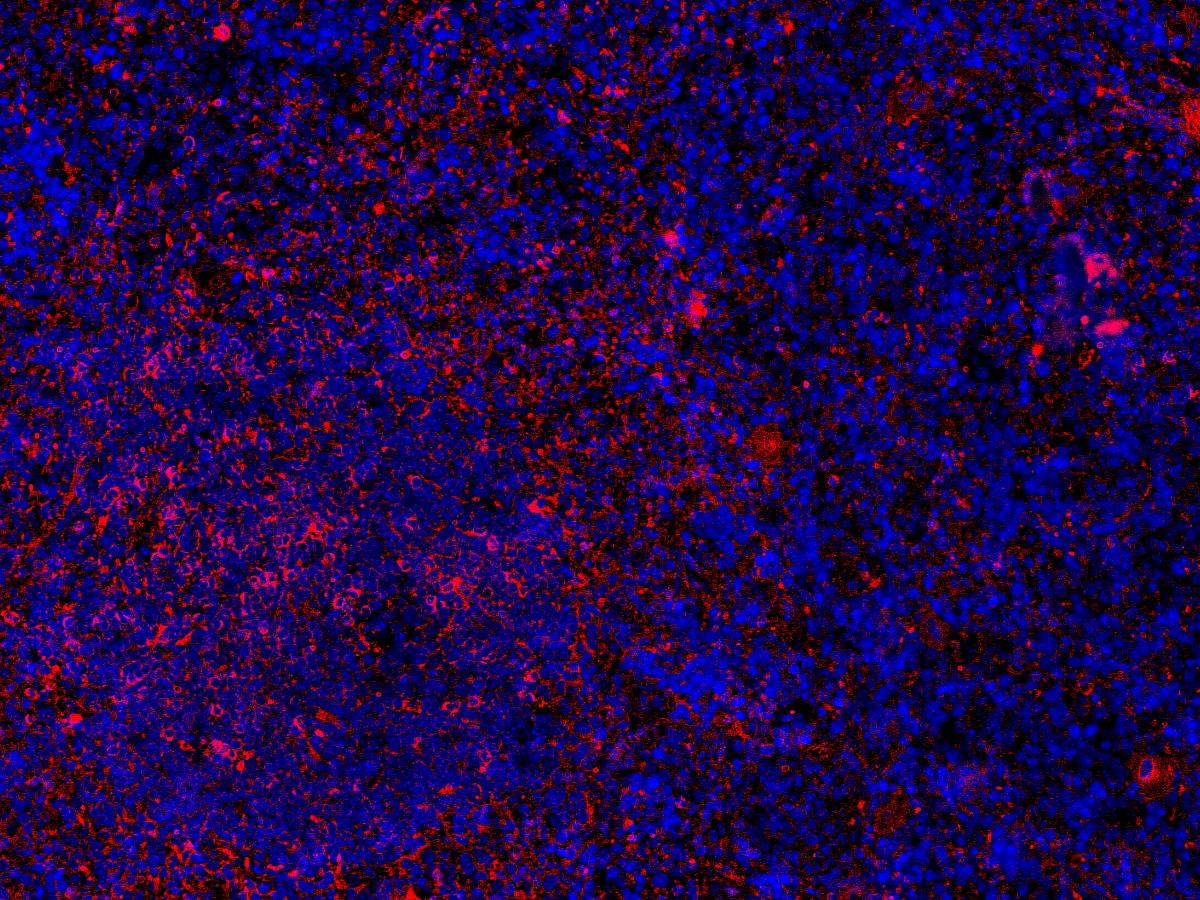

Supplement: Supplementary file 7 [file DataSheet5.zip › original images of figure 6/图6G-1-3-3(Merge).jpg]

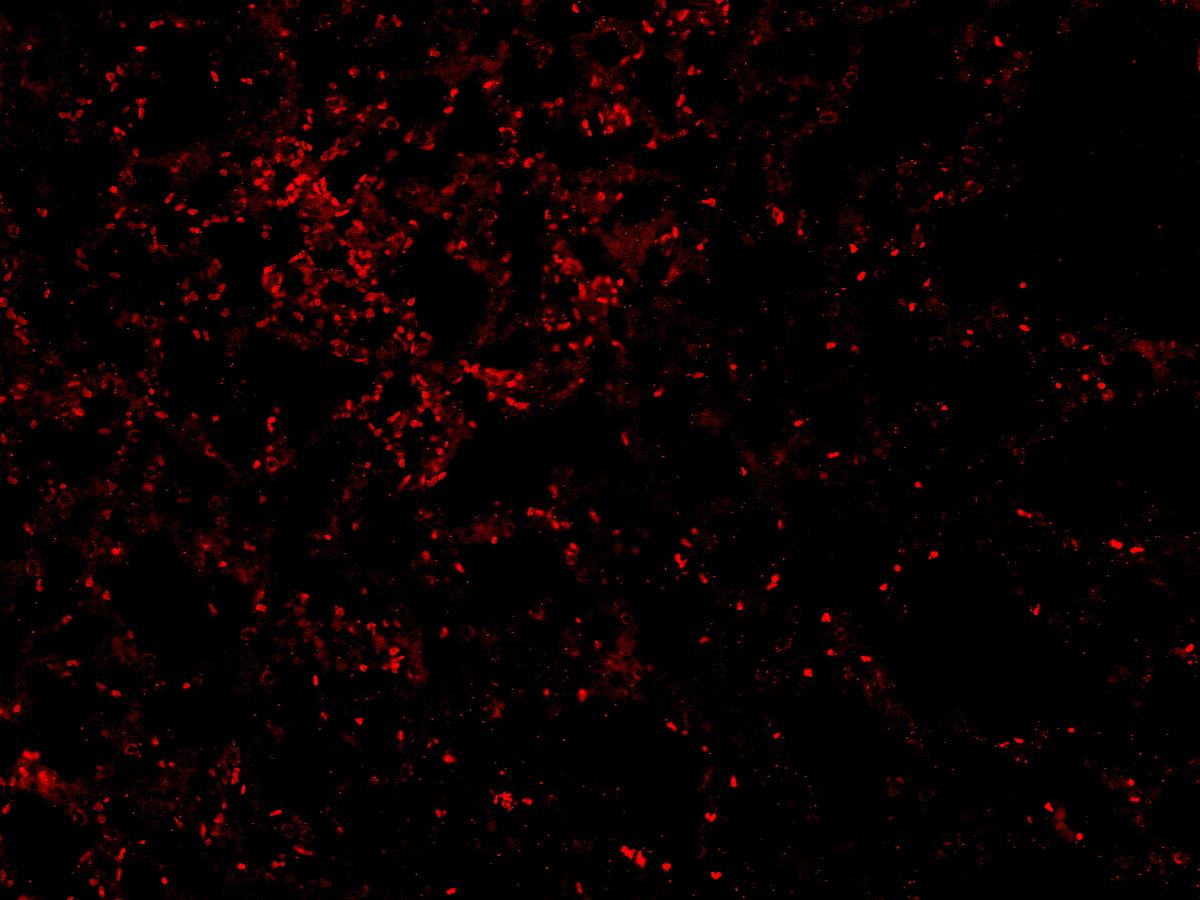

Supplement: Supplementary file 7 [file DataSheet5.zip › original images of figure 6/图6G-1-4-1(Dil).jpg]

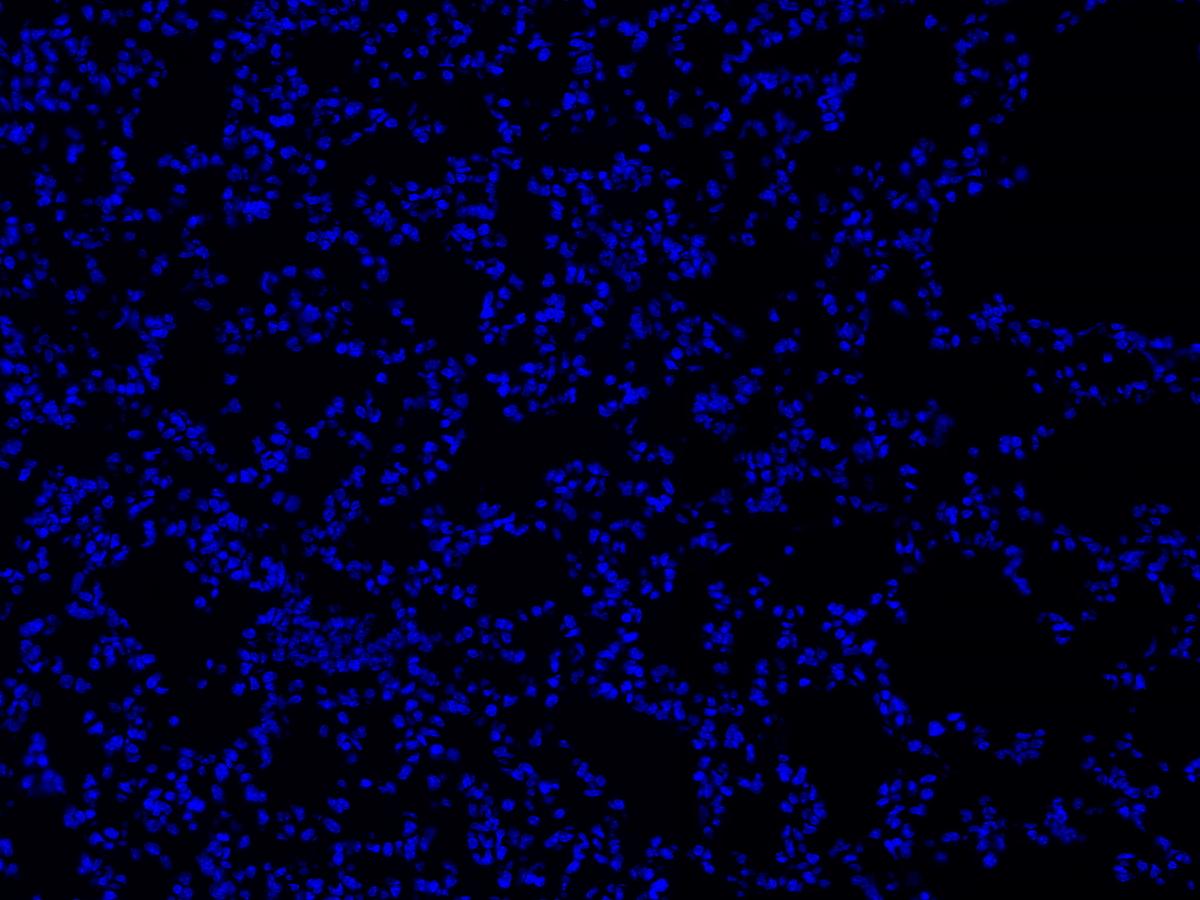

Supplement: Supplementary file 7 [file DataSheet5.zip › original images of figure 6/图6G-1-4-2(DAPI).jpg]

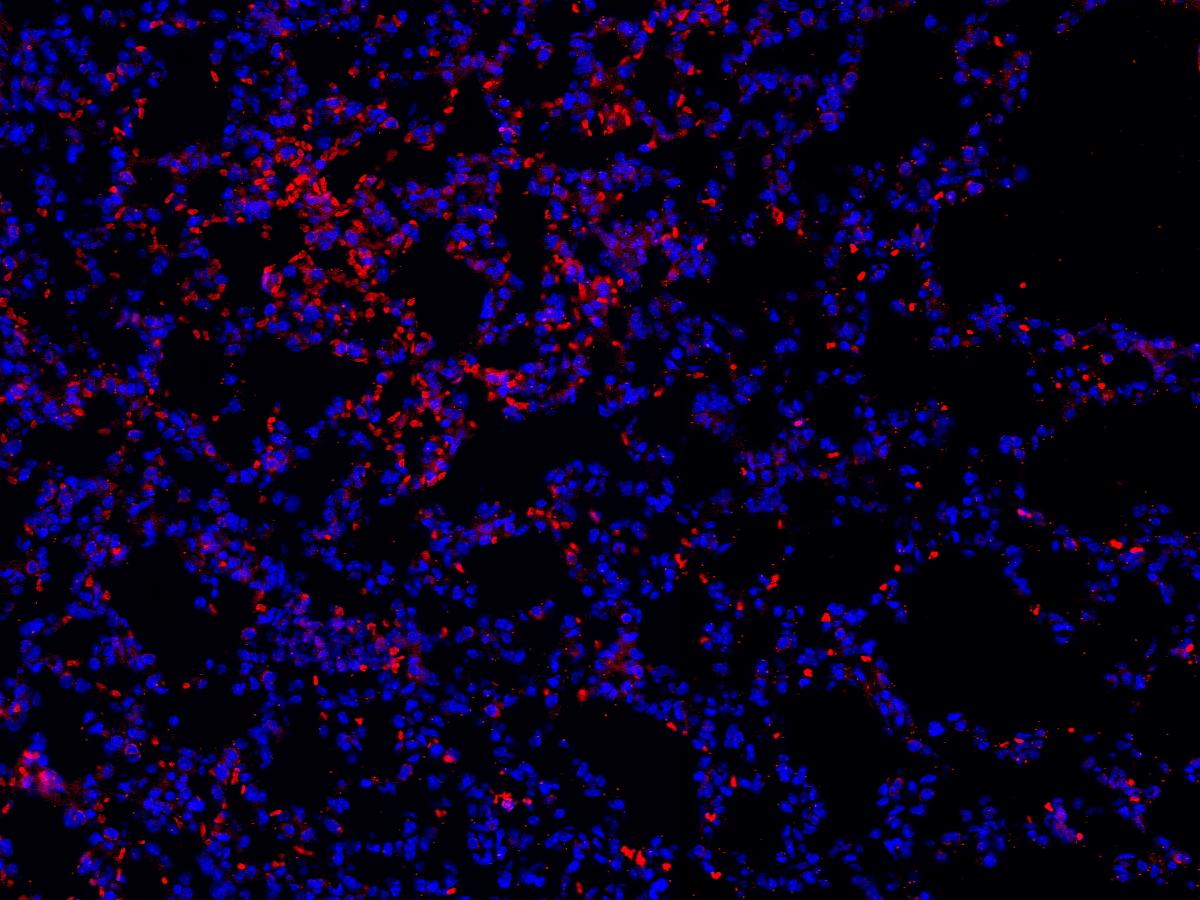

Supplement: Supplementary file 7 [file DataSheet5.zip › original images of figure 6/图6G-1-4-3(Merge).jpg]

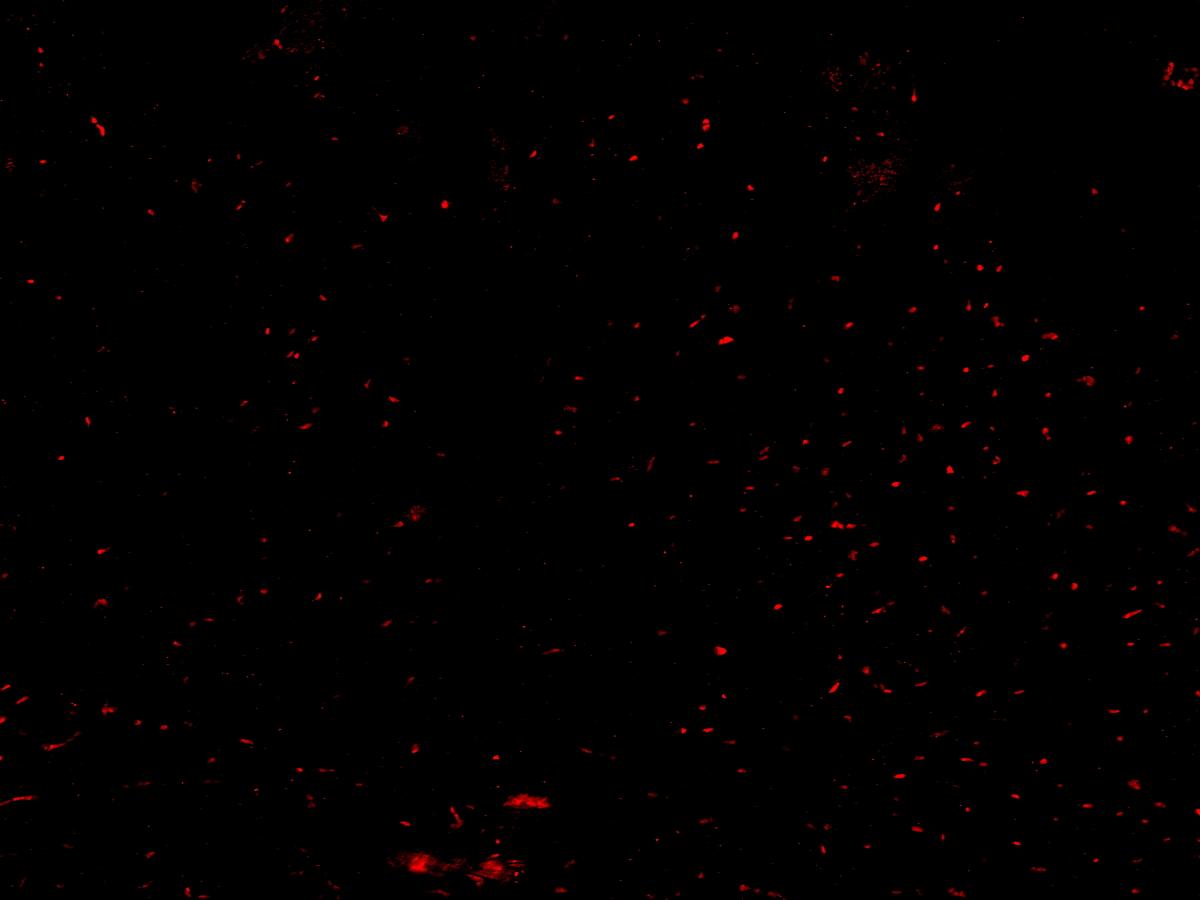

Supplement: Supplementary file 7 [file DataSheet5.zip › original images of figure 6/图6G-2-1-1(Dil).jpg]

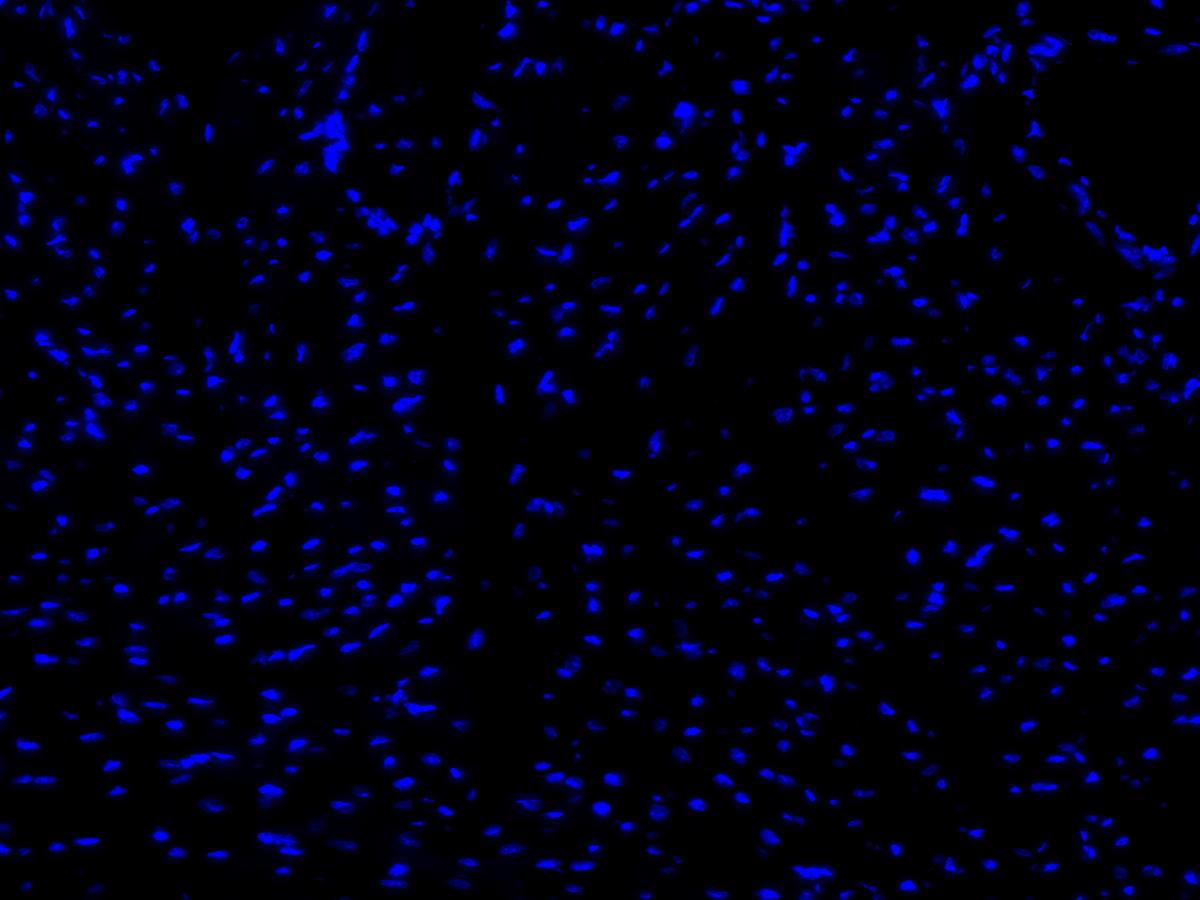

Supplement: Supplementary file 7 [file DataSheet5.zip › original images of figure 6/图6G-2-1-2(DAPI).jpg]

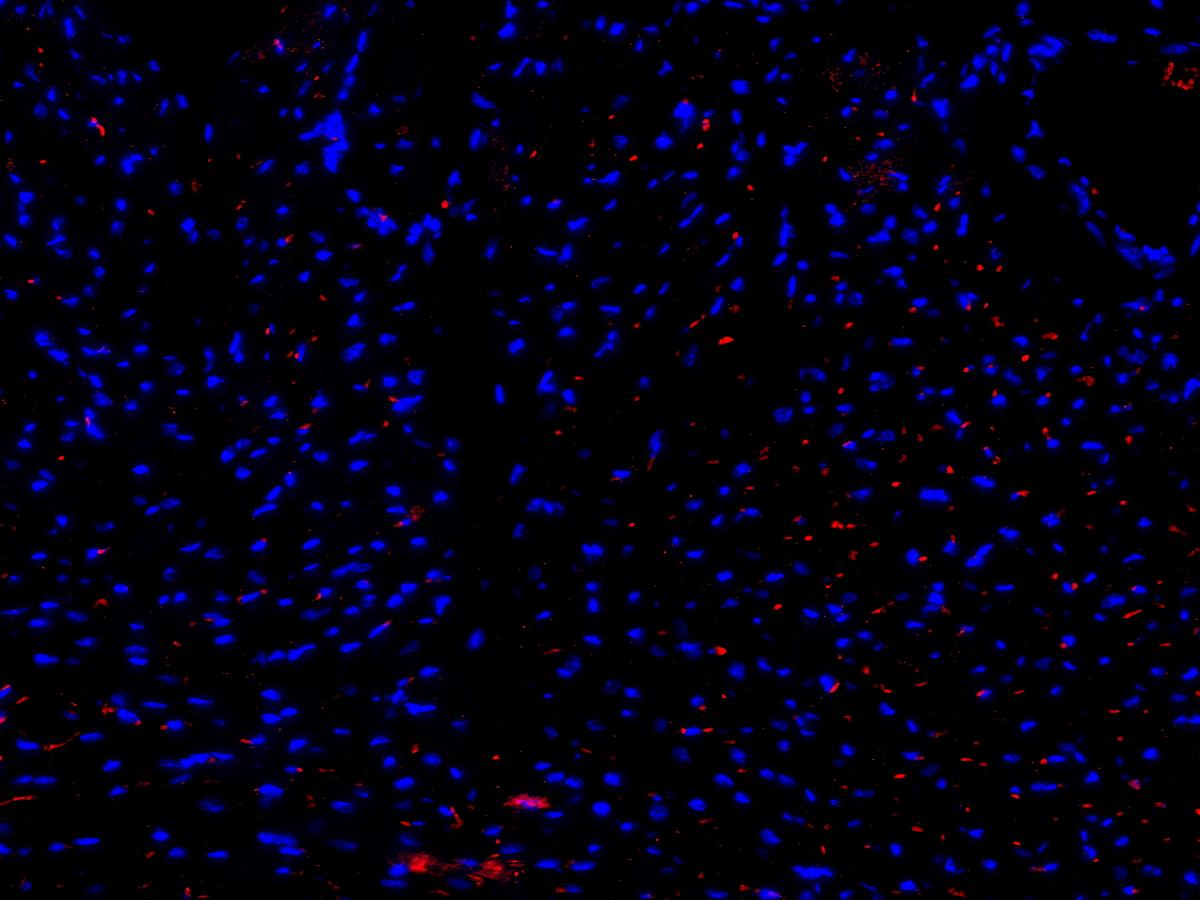

Supplement: Supplementary file 7 [file DataSheet5.zip › original images of figure 6/图6G-2-1-3(Merge).jpg]

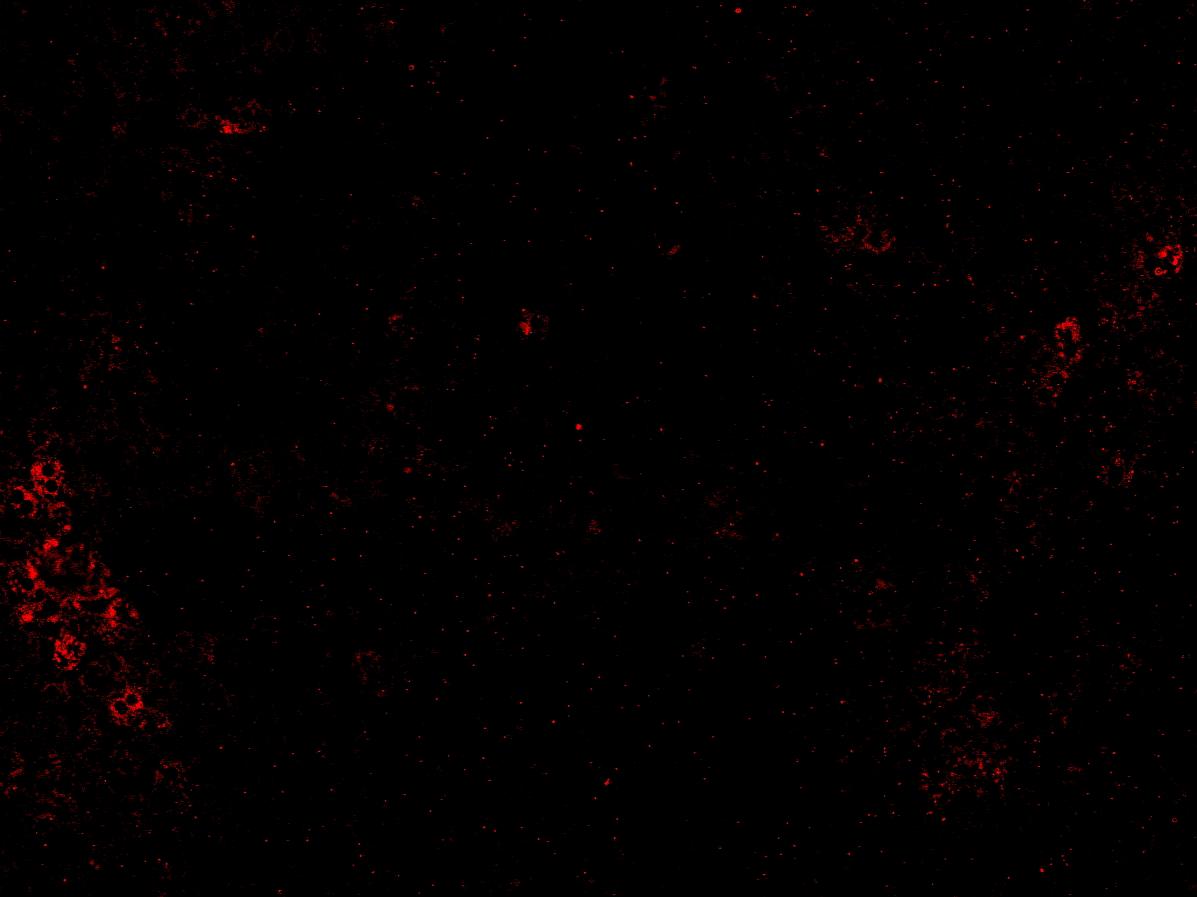

Supplement: Supplementary file 7 [file DataSheet5.zip › original images of figure 6/图6G-2-2-1(Dil).jpg]

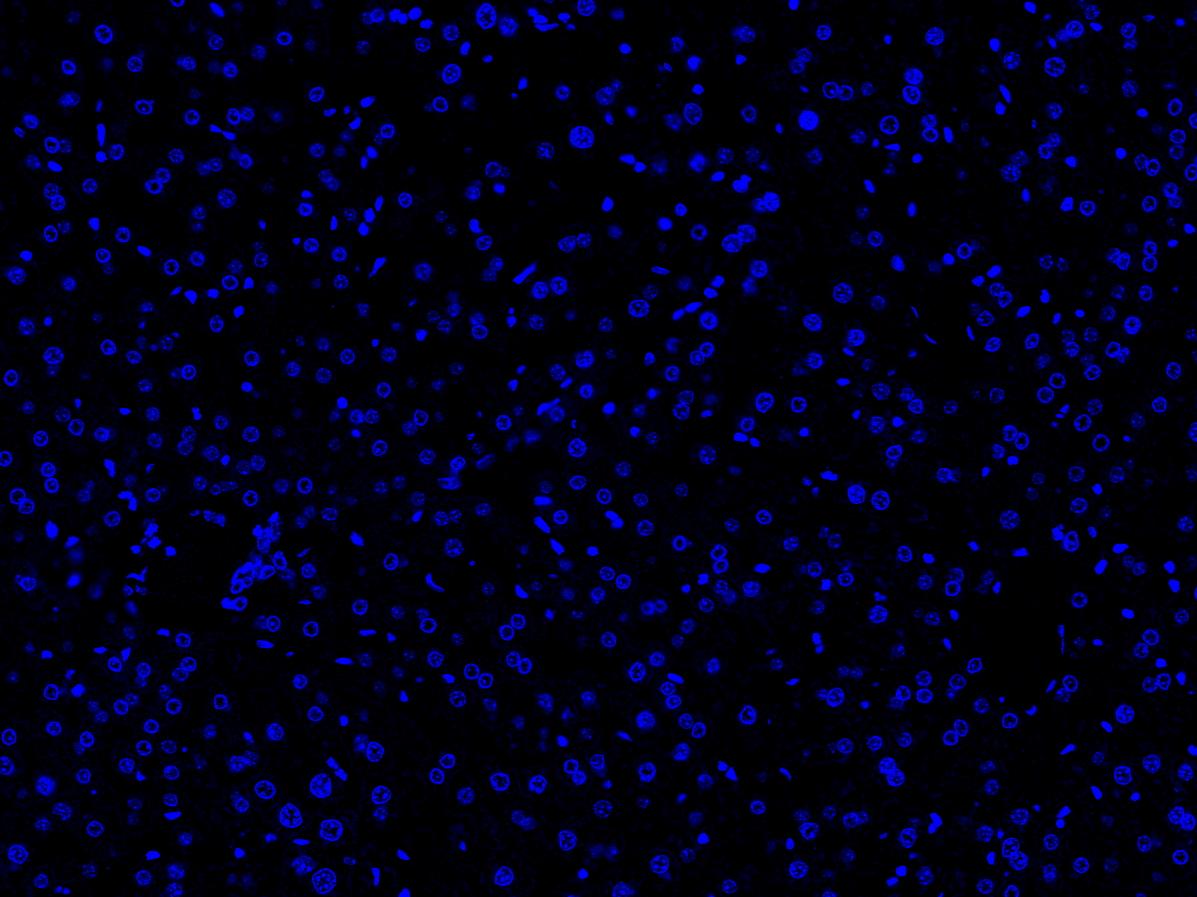

Supplement: Supplementary file 7 [file DataSheet5.zip › original images of figure 6/图6G-2-2-2(DAPI).jpg]

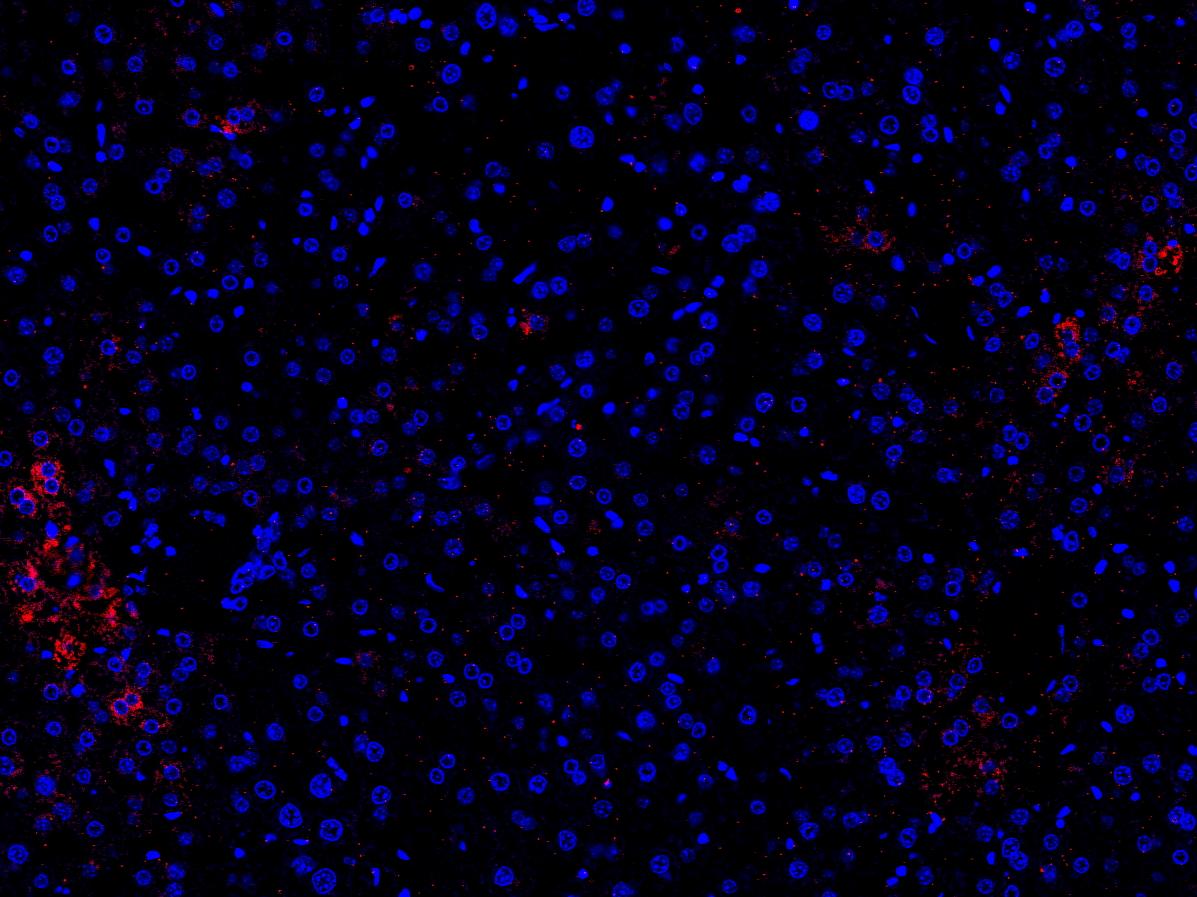

Supplement: Supplementary file 7 [file DataSheet5.zip › original images of figure 6/图6G-2-2-3(Merge).jpg]

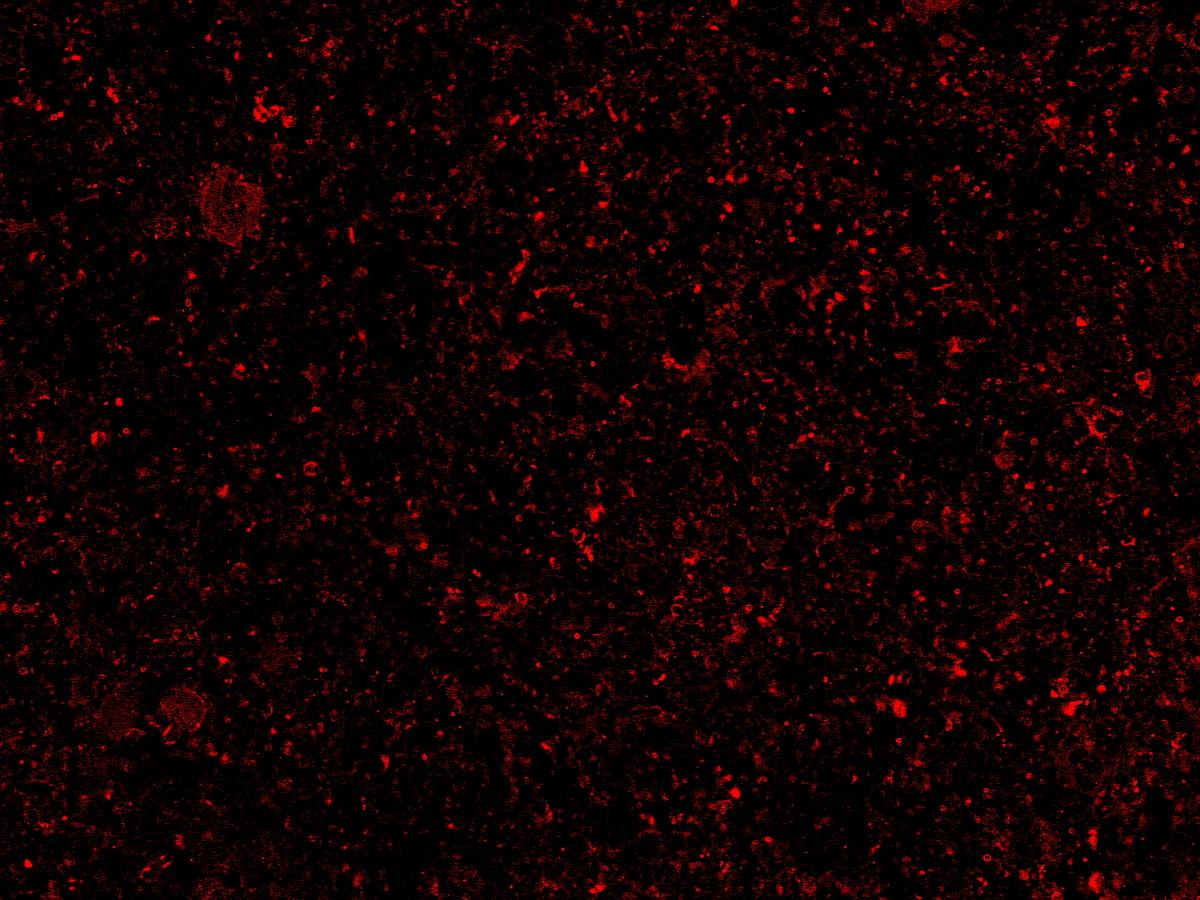

Supplement: Supplementary file 7 [file DataSheet5.zip › original images of figure 6/图6G-2-3-1(Dil).jpg]

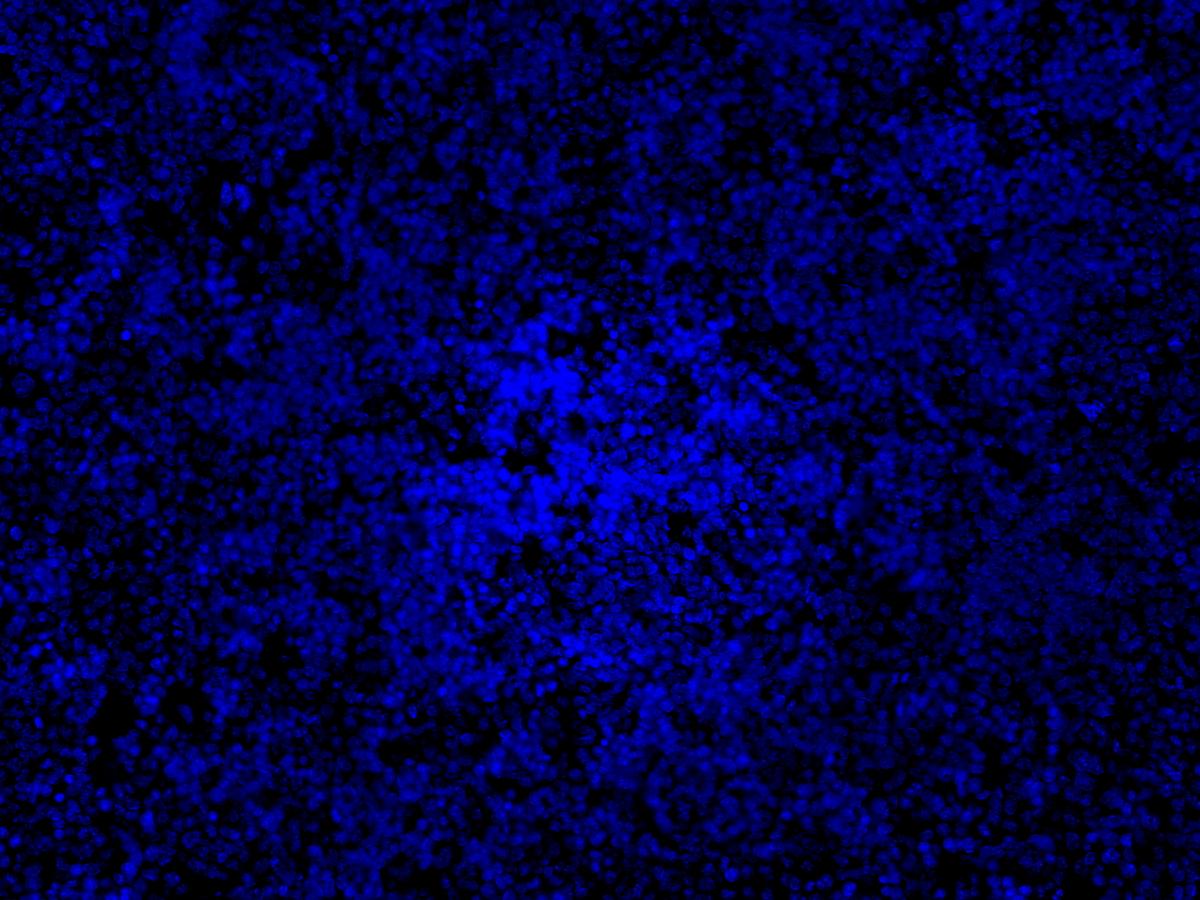

Supplement: Supplementary file 7 [file DataSheet5.zip › original images of figure 6/图6G-2-3-2(DAPI).jpg]

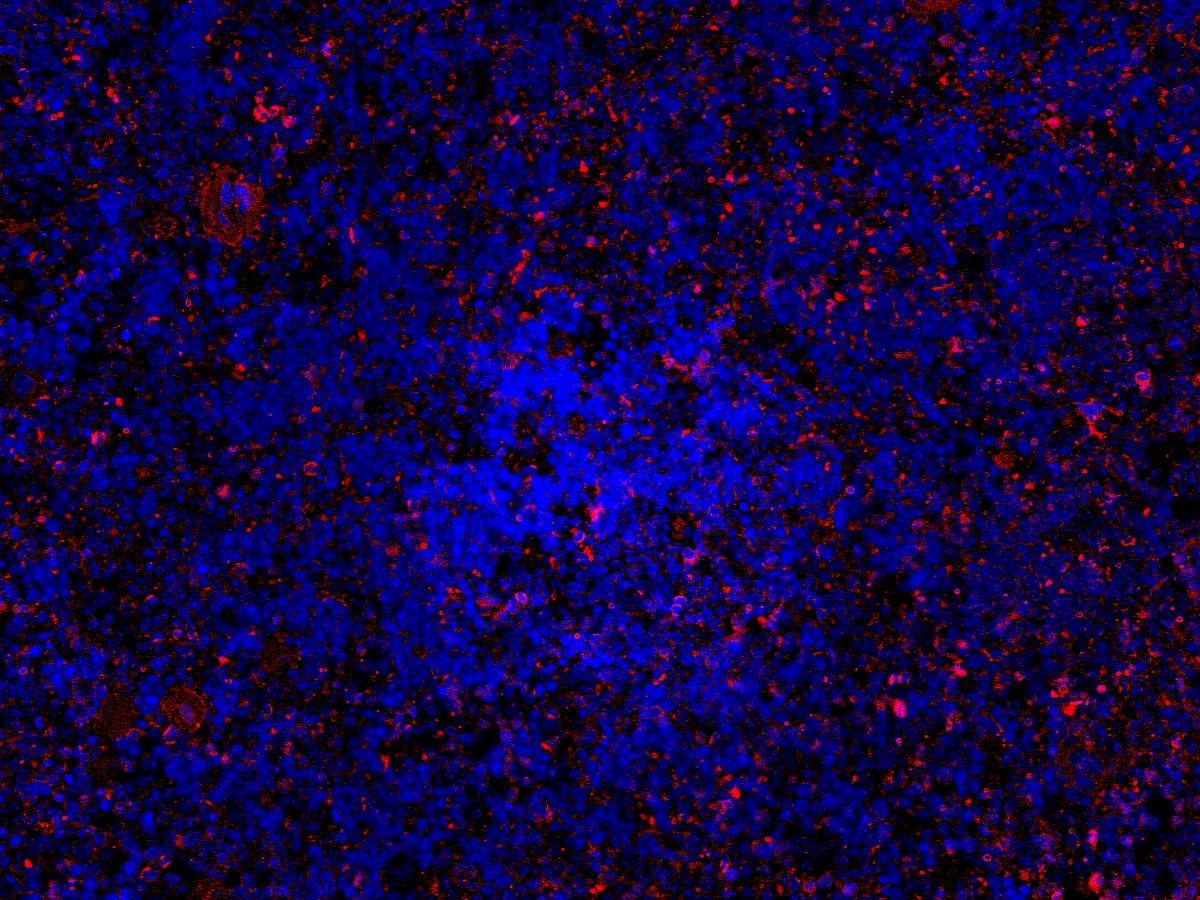

Supplement: Supplementary file 7 [file DataSheet5.zip › original images of figure 6/图6G-2-3-3(Merge).jpg]

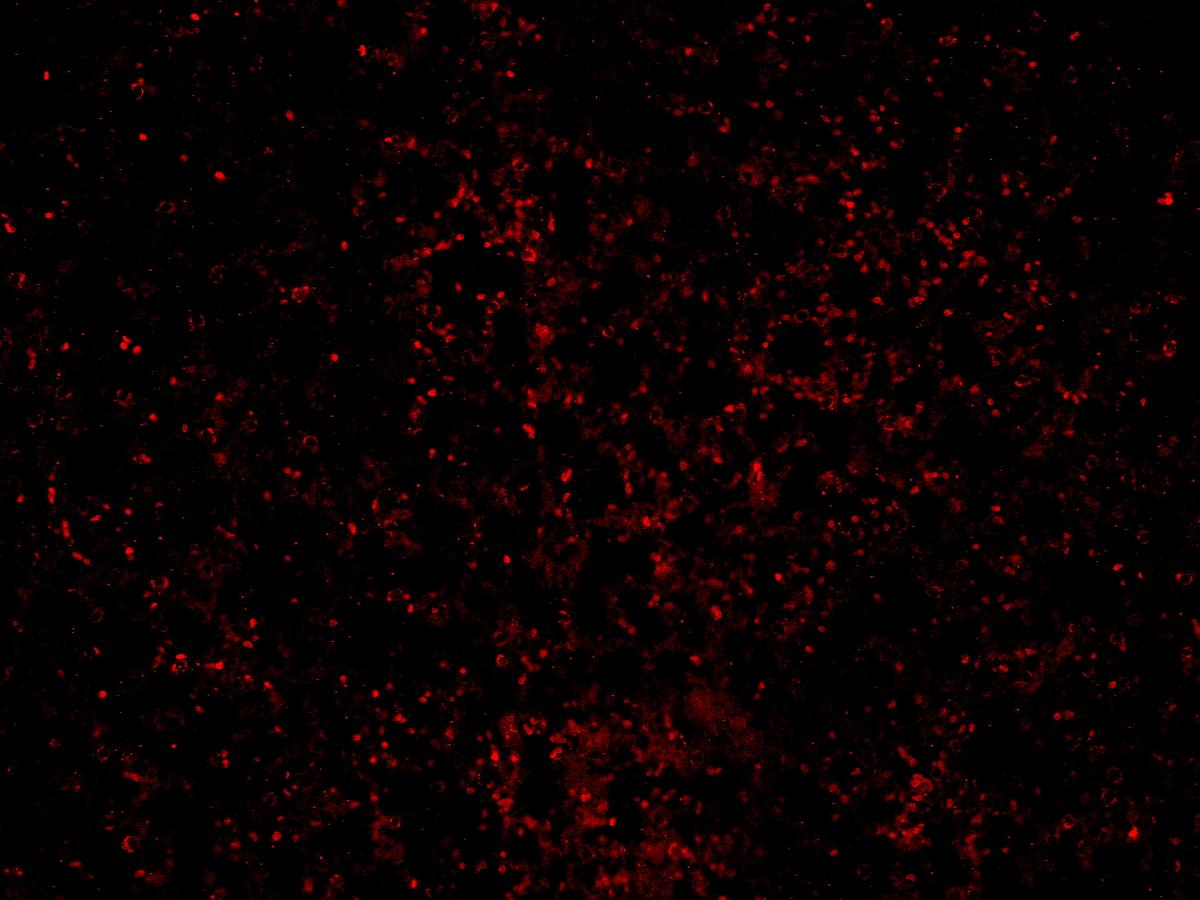

Supplement: Supplementary file 7 [file DataSheet5.zip › original images of figure 6/图6G-2-4-1(Dil).jpg]

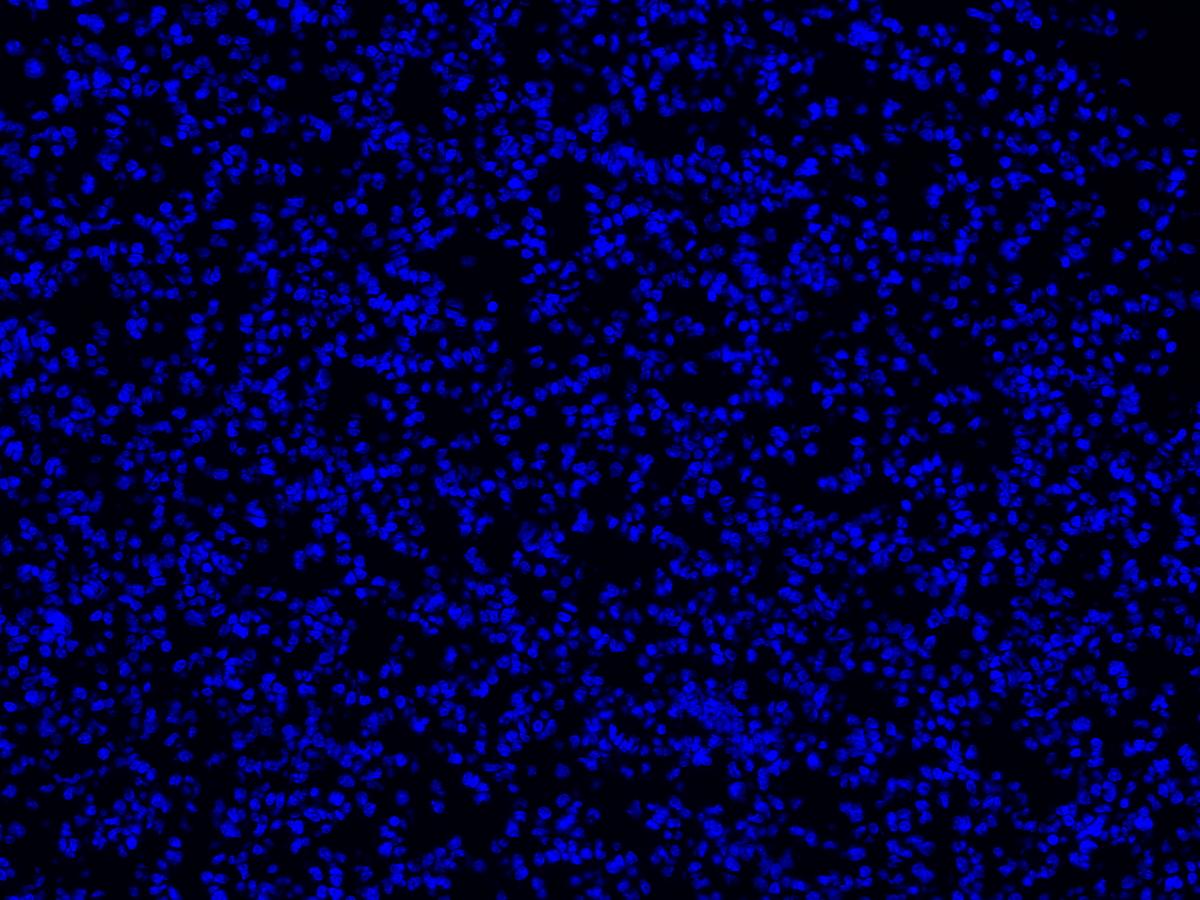

Supplement: Supplementary file 7 [file DataSheet5.zip › original images of figure 6/图6G-2-4-2(DAPI).jpg]

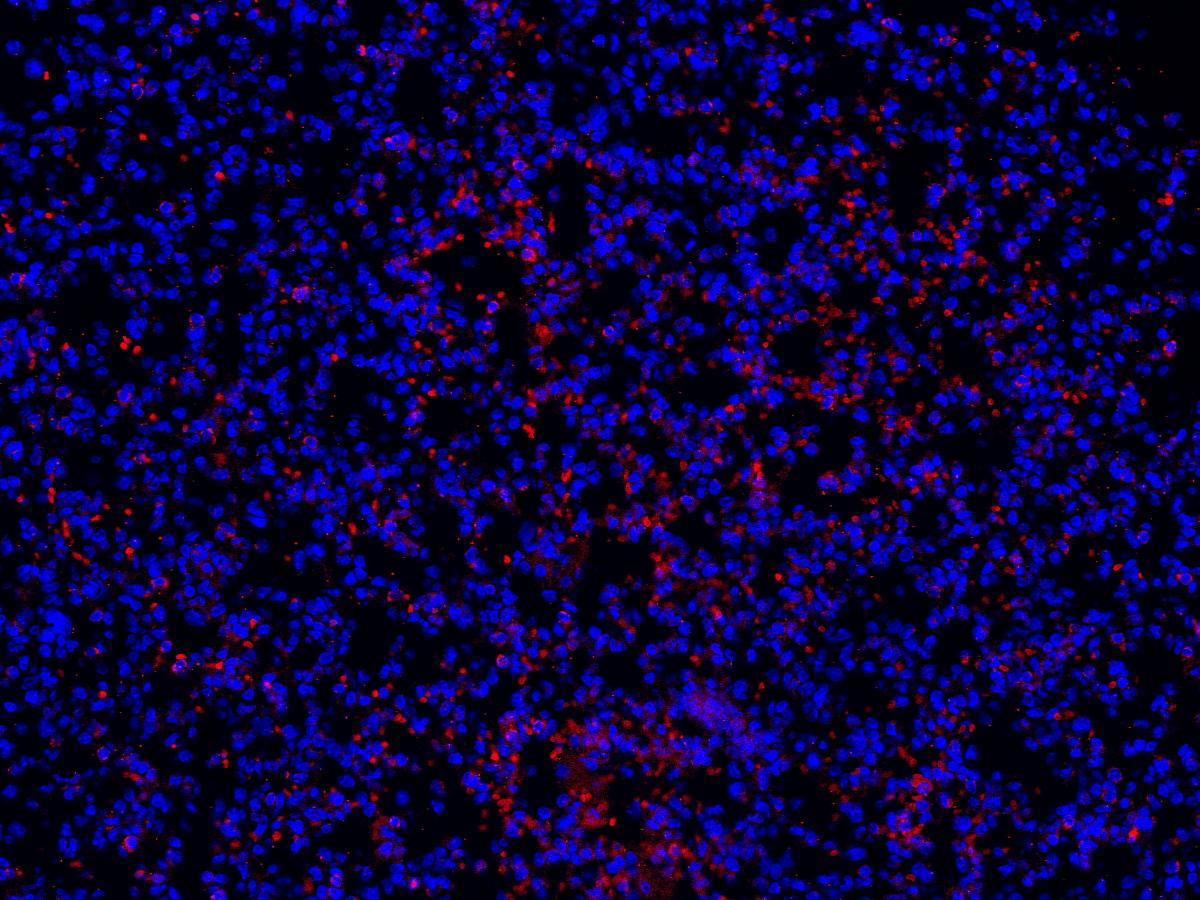

Supplement: Supplementary file 7 [file DataSheet5.zip › original images of figure 6/图6G-2-4-3(Merge).jpg]

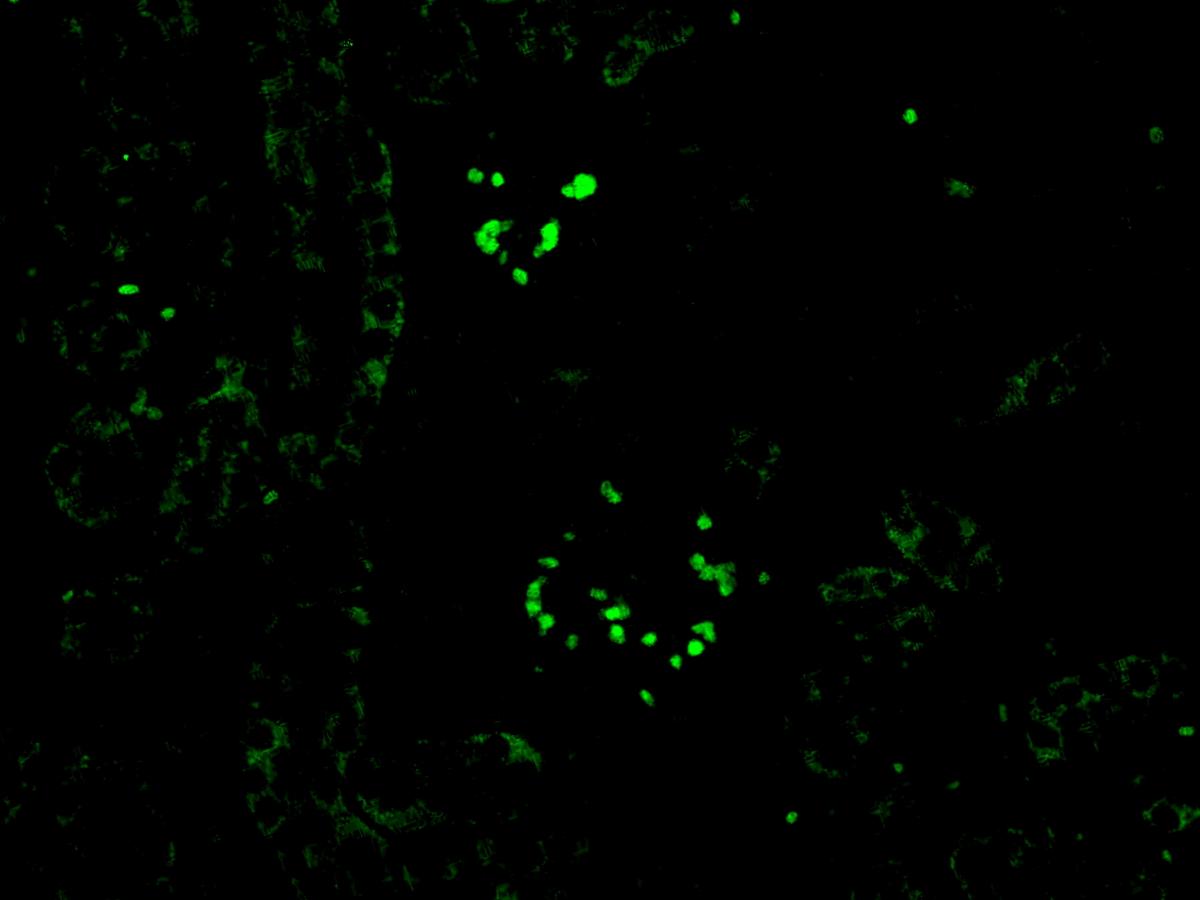

Supplement: Supplementary file 7 [file DataSheet5.zip › original images of figure 6/图6I-1-1.jpg]

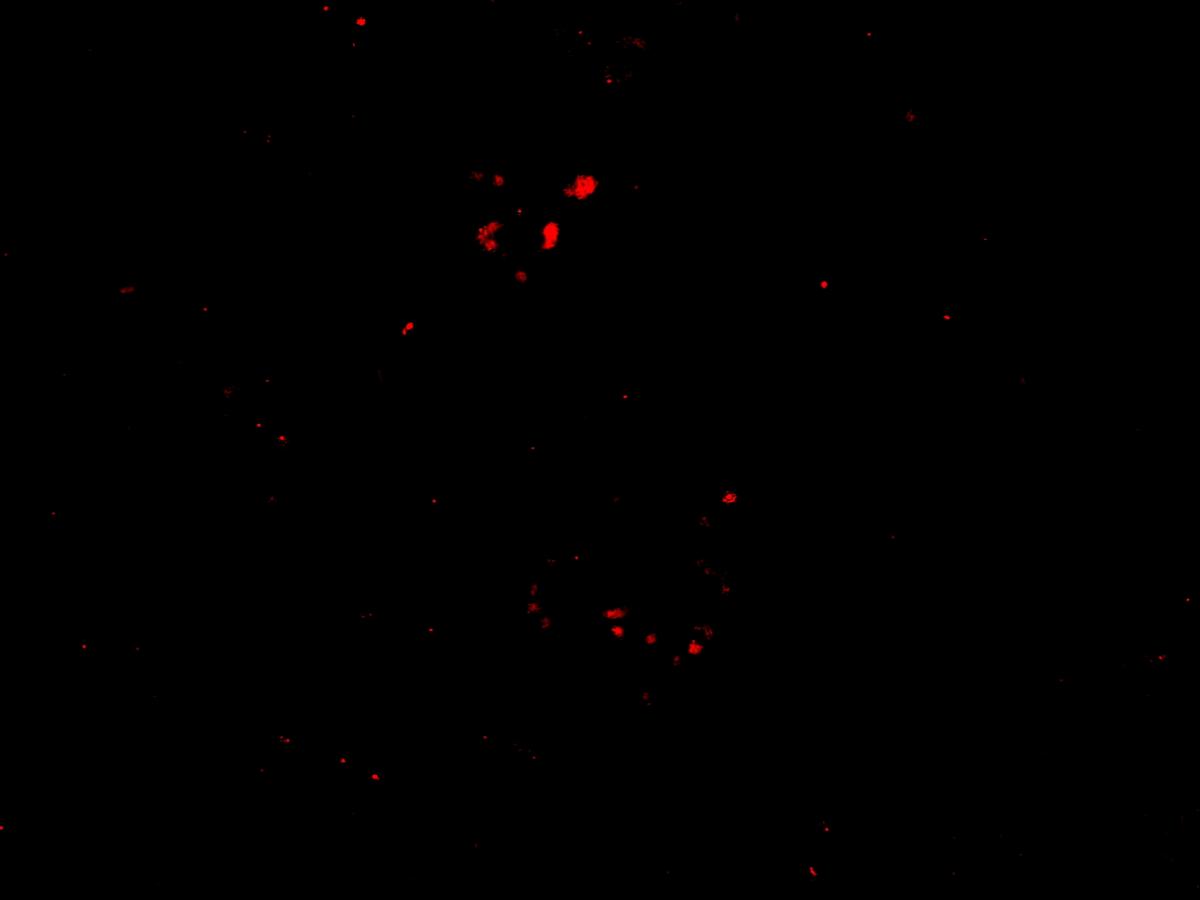

Supplement: Supplementary file 7 [file DataSheet5.zip › original images of figure 6/图6I-1-2.jpg]

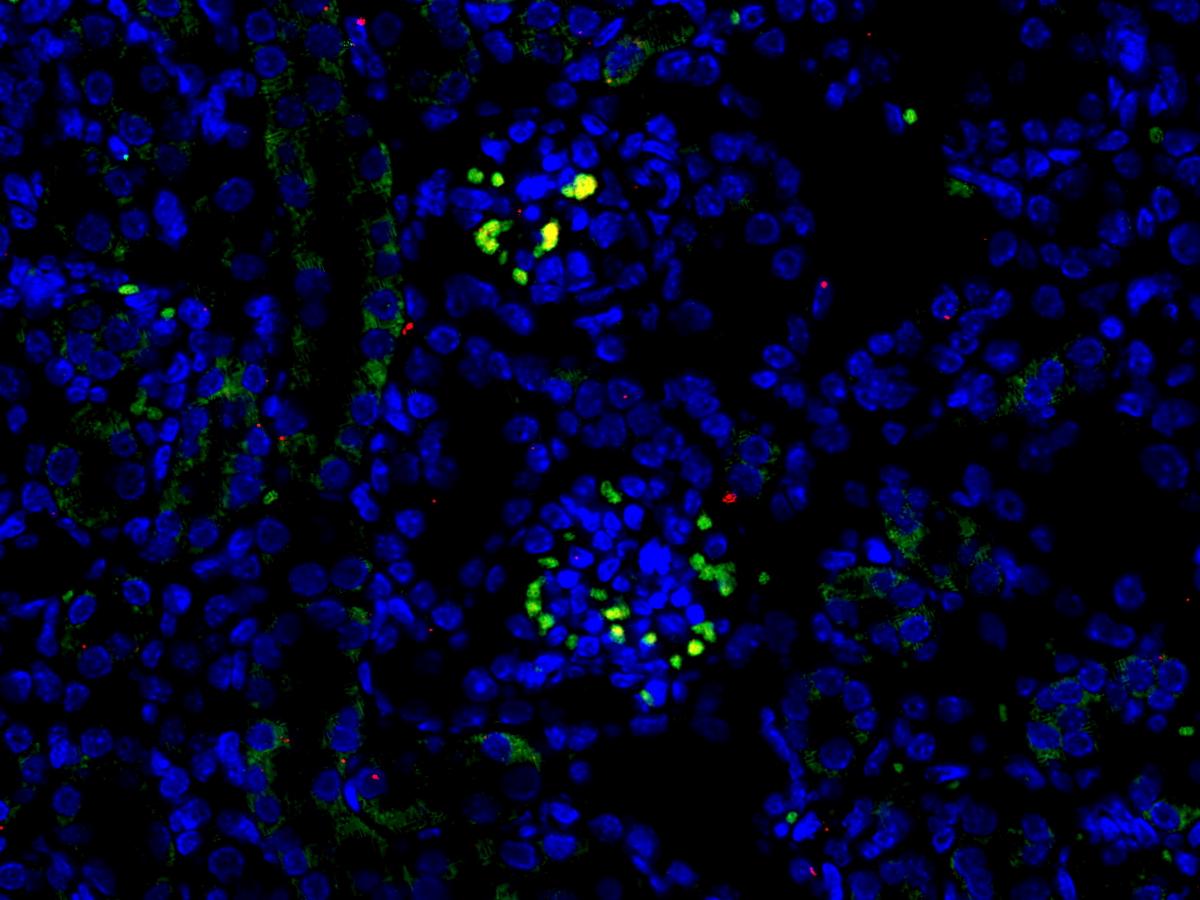

Supplement: Supplementary file 7 [file DataSheet5.zip › original images of figure 6/图6I-1-4.jpg]

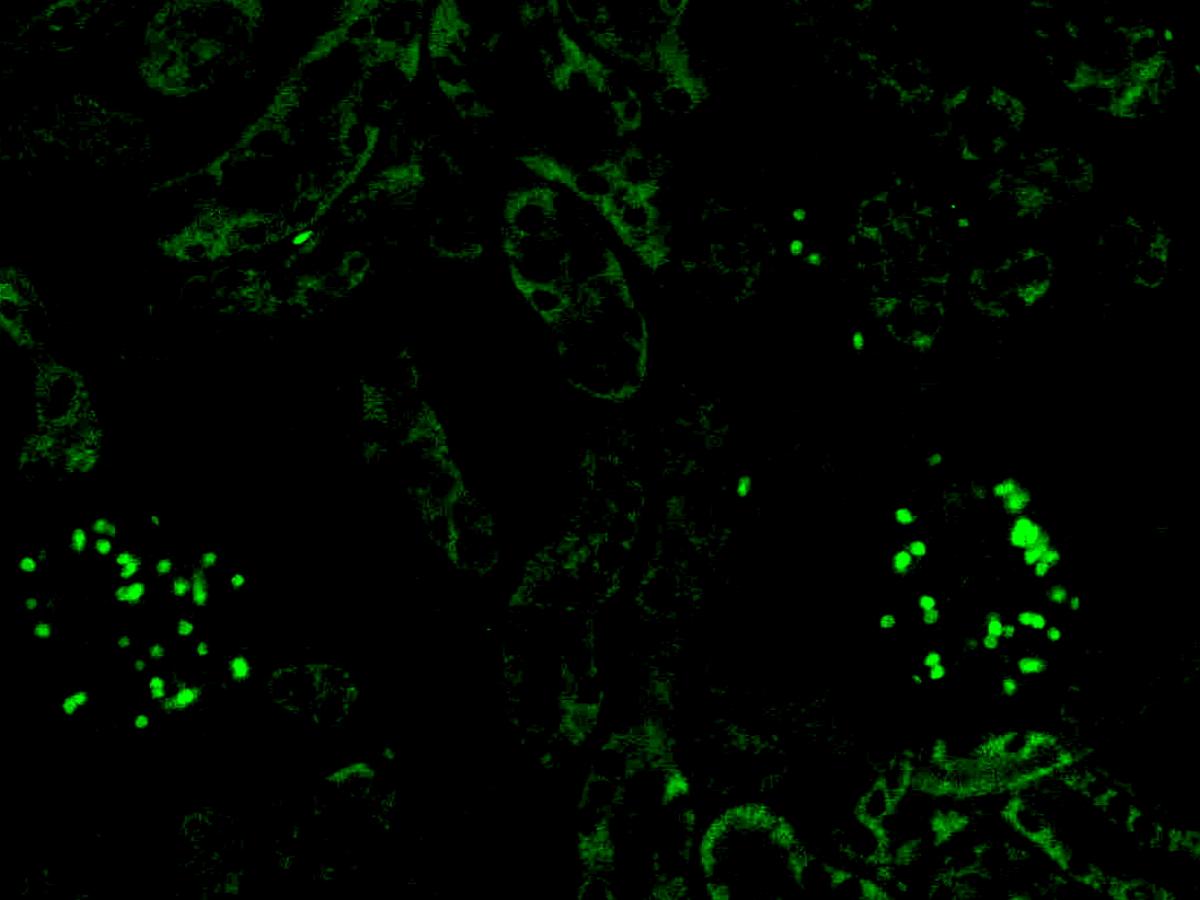

Supplement: Supplementary file 7 [file DataSheet5.zip › original images of figure 6/图6I-2-1.jpg]

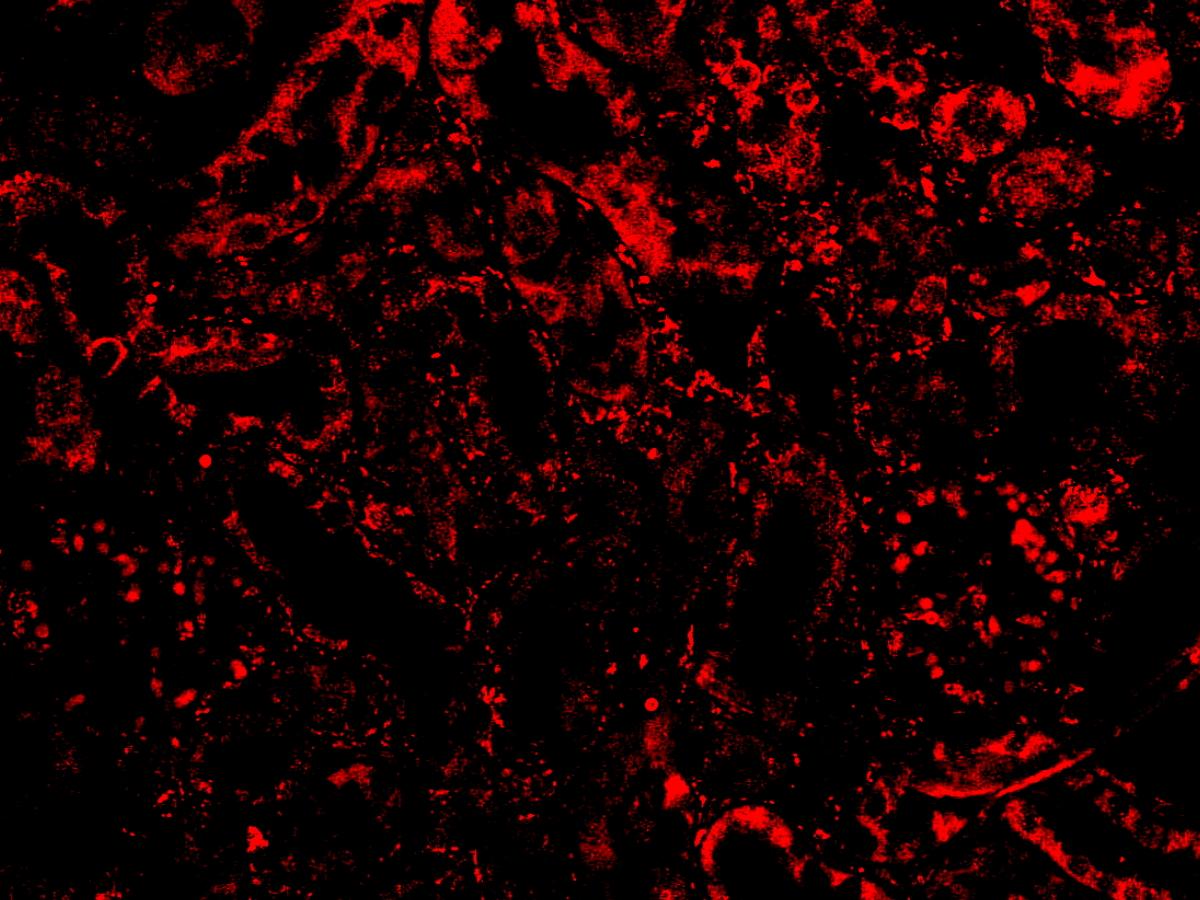

Supplement: Supplementary file 7 [file DataSheet5.zip › original images of figure 6/图6I-2-2.jpg]

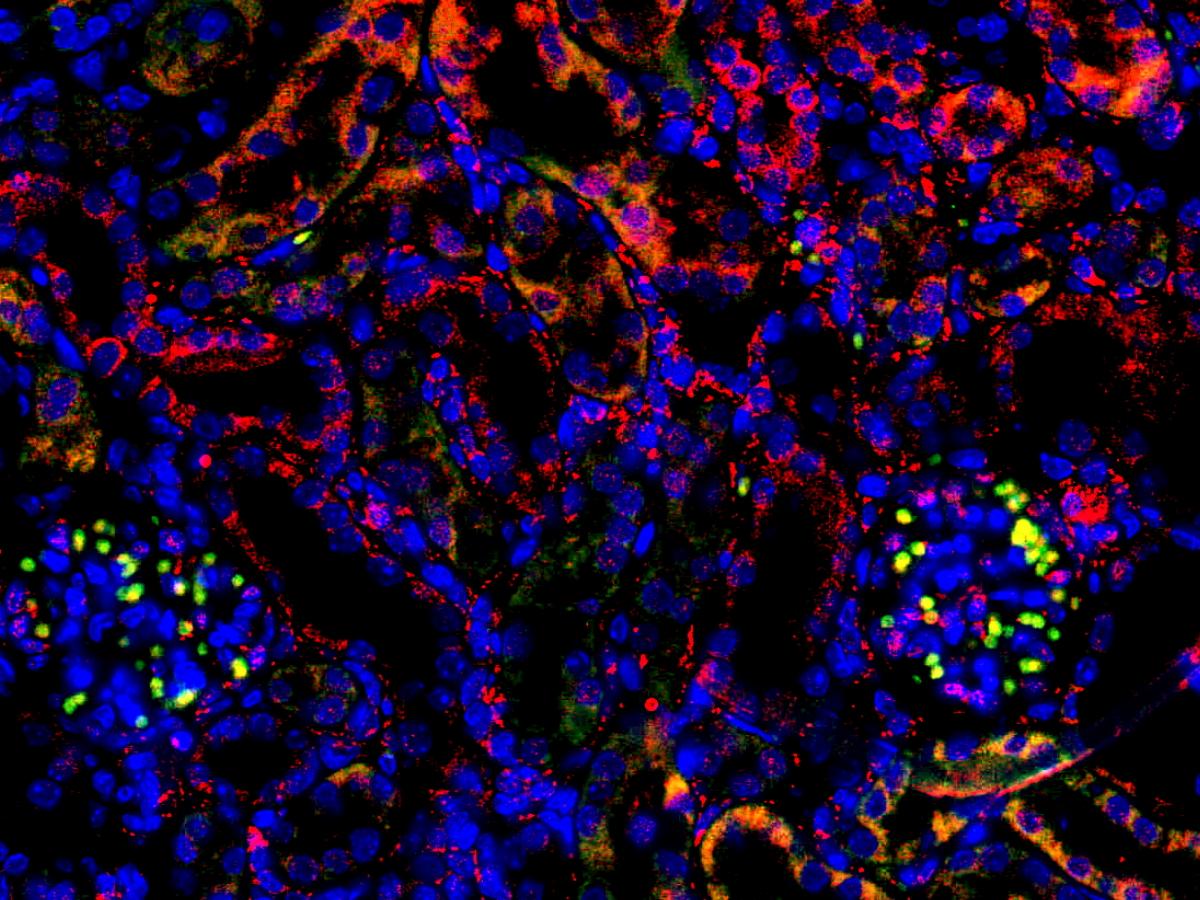

Supplement: Supplementary file 7 [file DataSheet5.zip › original images of figure 6/图6I-2-4.jpg]

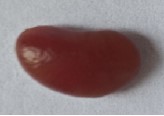

Supplement: Supplementary file 8 [file DataSheet7.zip › original images of figure 8/图8B-1.jpg]

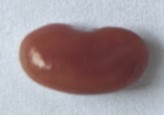

Supplement: Supplementary file 8 [file DataSheet7.zip › original images of figure 8/图8B-2.jpg]

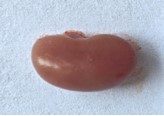

Supplement: Supplementary file 8 [file DataSheet7.zip › original images of figure 8/图8B-3.jpg]

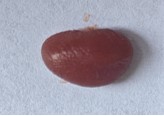

Supplement: Supplementary file 8 [file DataSheet7.zip › original images of figure 8/图8B-4.jpg]

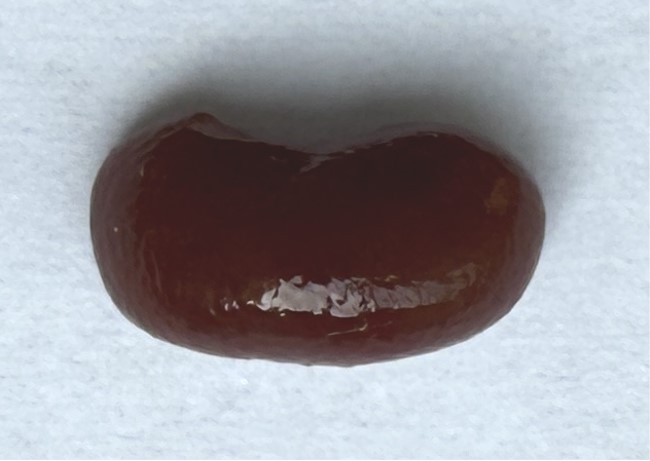

Supplement: Supplementary file 8 [file DataSheet7.zip › original images of figure 8/图8B-5.jpg]

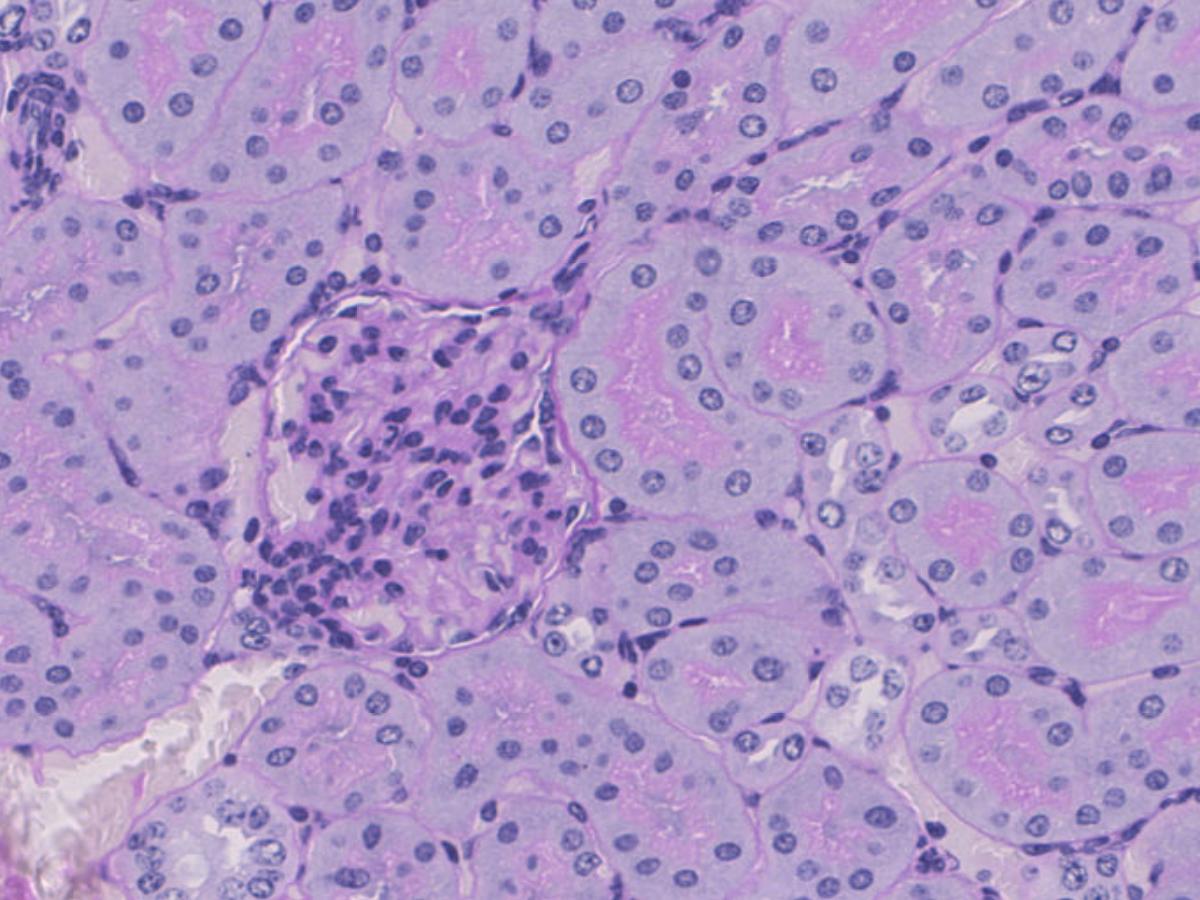

Supplement: Supplementary file 8 [file DataSheet7.zip › original images of figure 8/图8E-1.jpg]

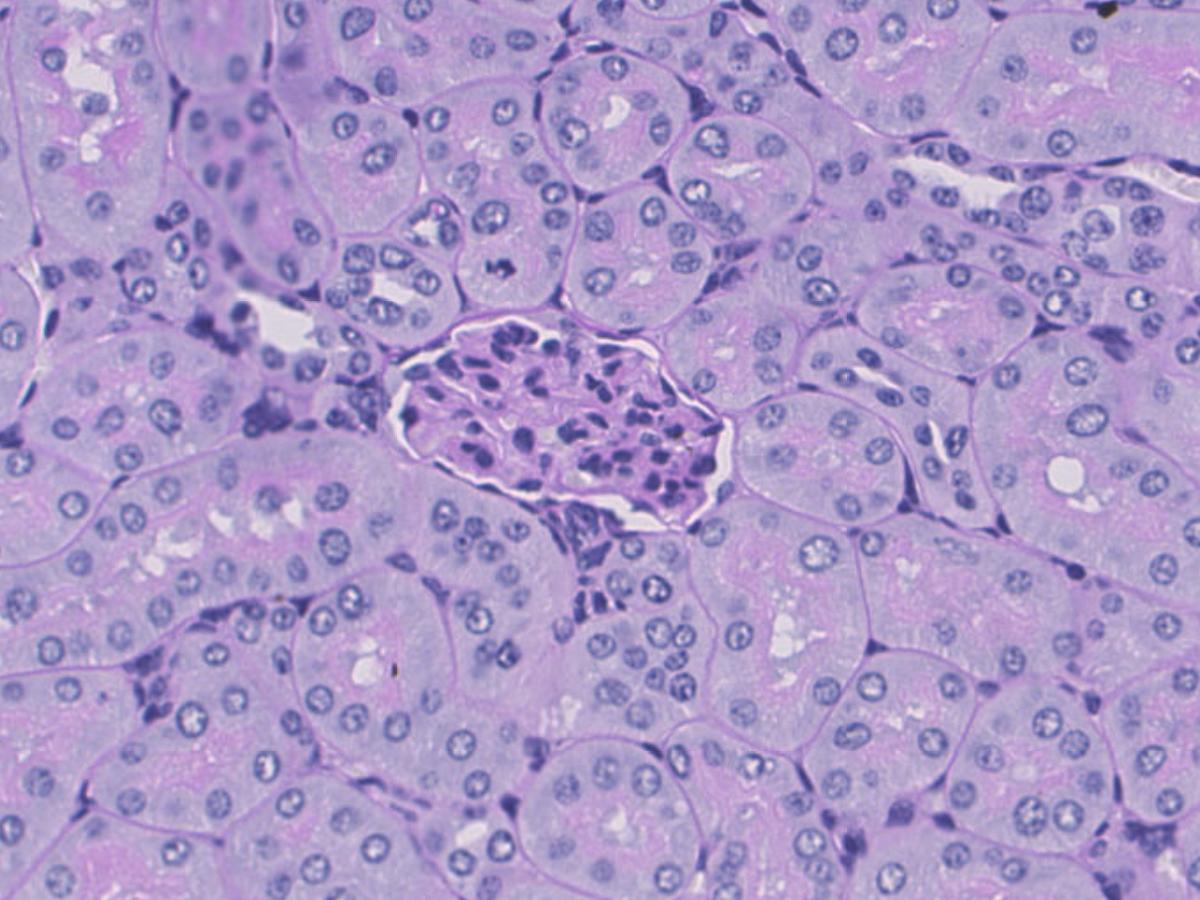

Supplement: Supplementary file 8 [file DataSheet7.zip › original images of figure 8/图8E-2.jpg]

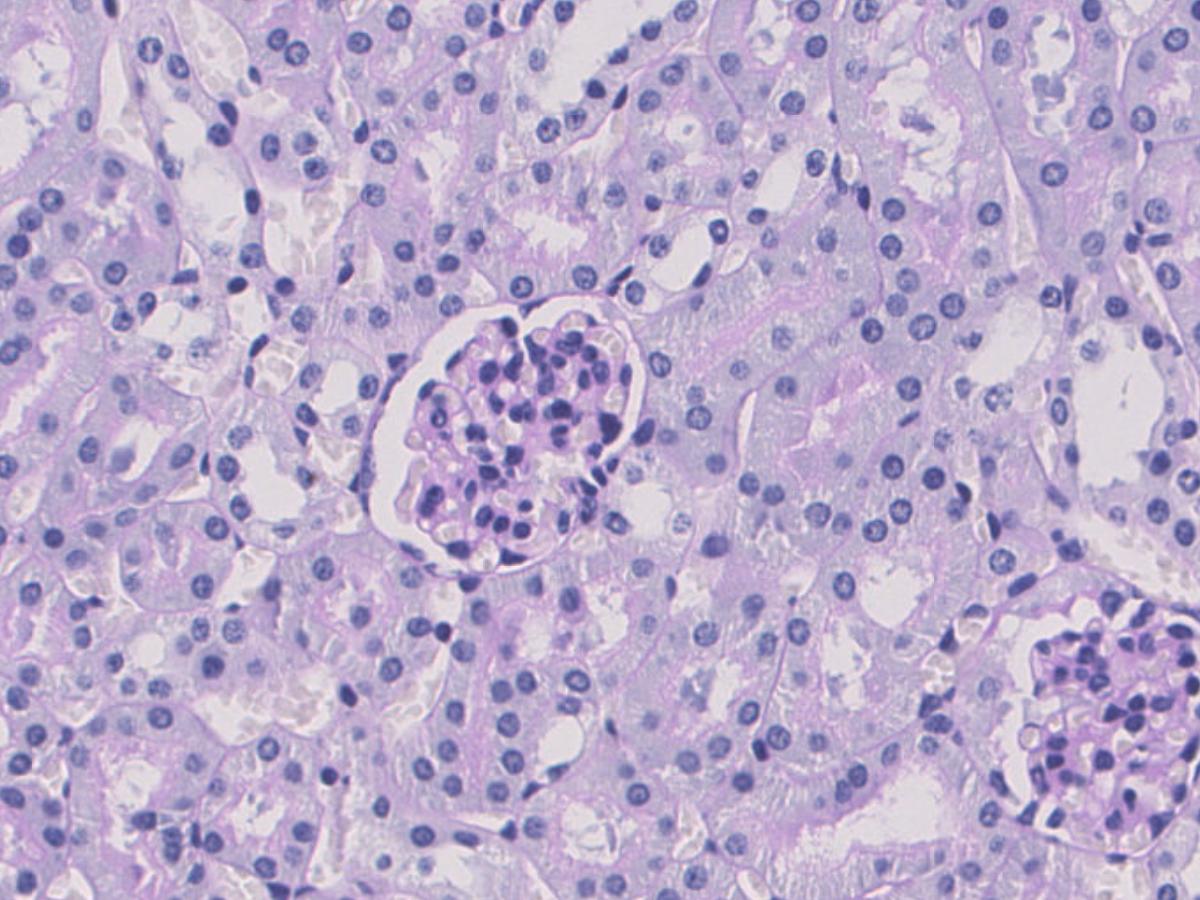

Supplement: Supplementary file 8 [file DataSheet7.zip › original images of figure 8/图8E-3.jpg]

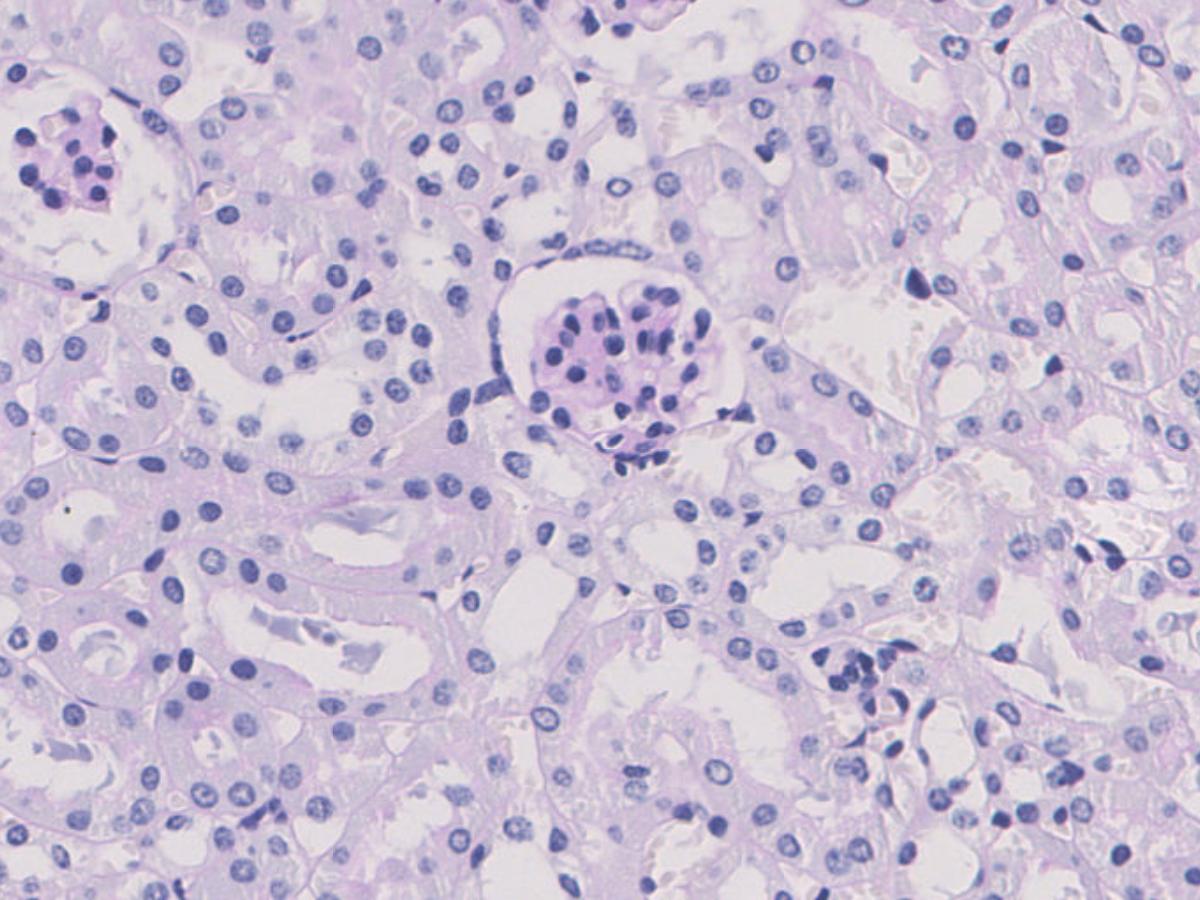

Supplement: Supplementary file 8 [file DataSheet7.zip › original images of figure 8/图8E-4.jpg]

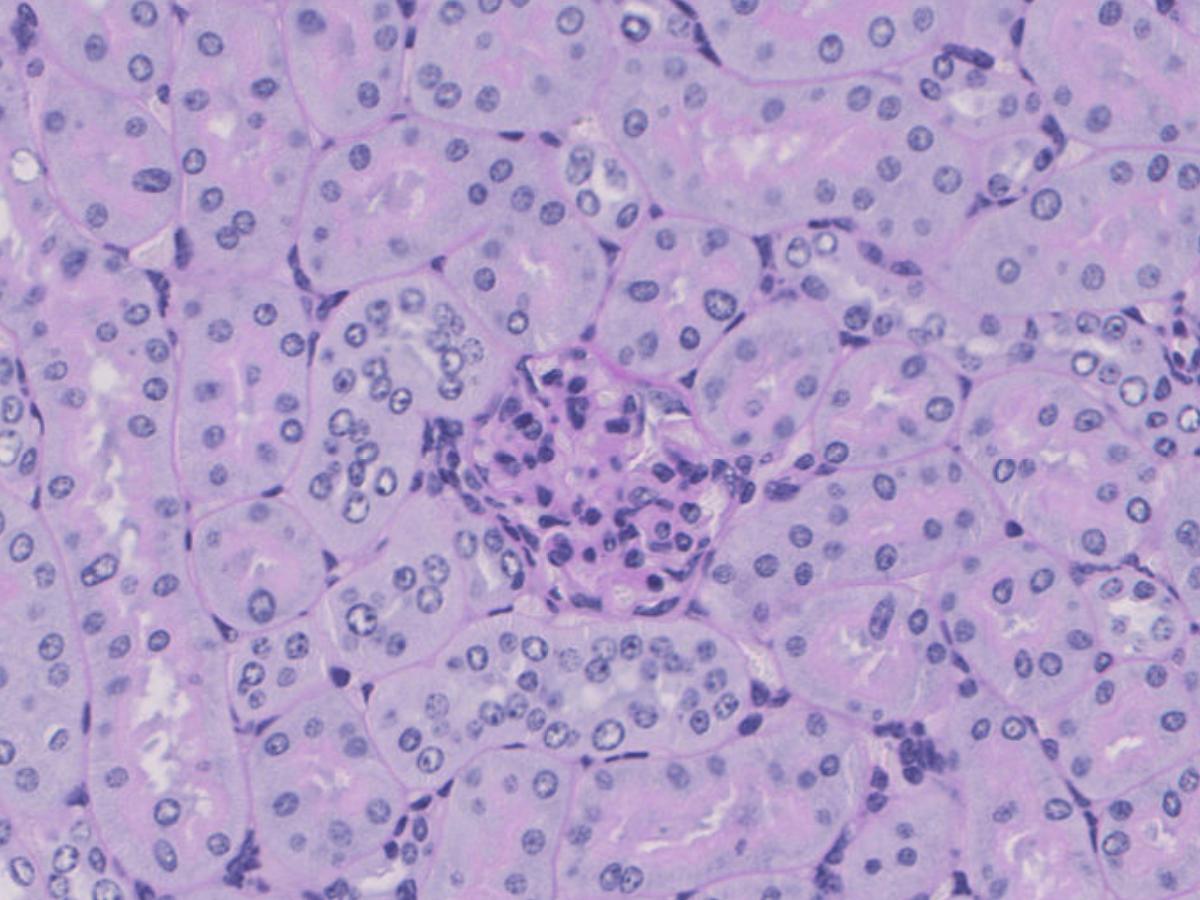

Supplement: Supplementary file 8 [file DataSheet7.zip › original images of figure 8/图8E-5.jpg]
