# Supplementary figures and images for: Bioengineered exosome-mRNA hybrids: a breakthrough in targeted miRNA delivery for diabetic kidney fibrosis therapy (part 3 of 3)
Source: Front Bioeng Biotechnol. 2026 Mar 2;14:1709588. doi: 10.3389/fbioe.2026.1709588 (PMC12989603; doi:10.3389/fbioe.2026.1709588)

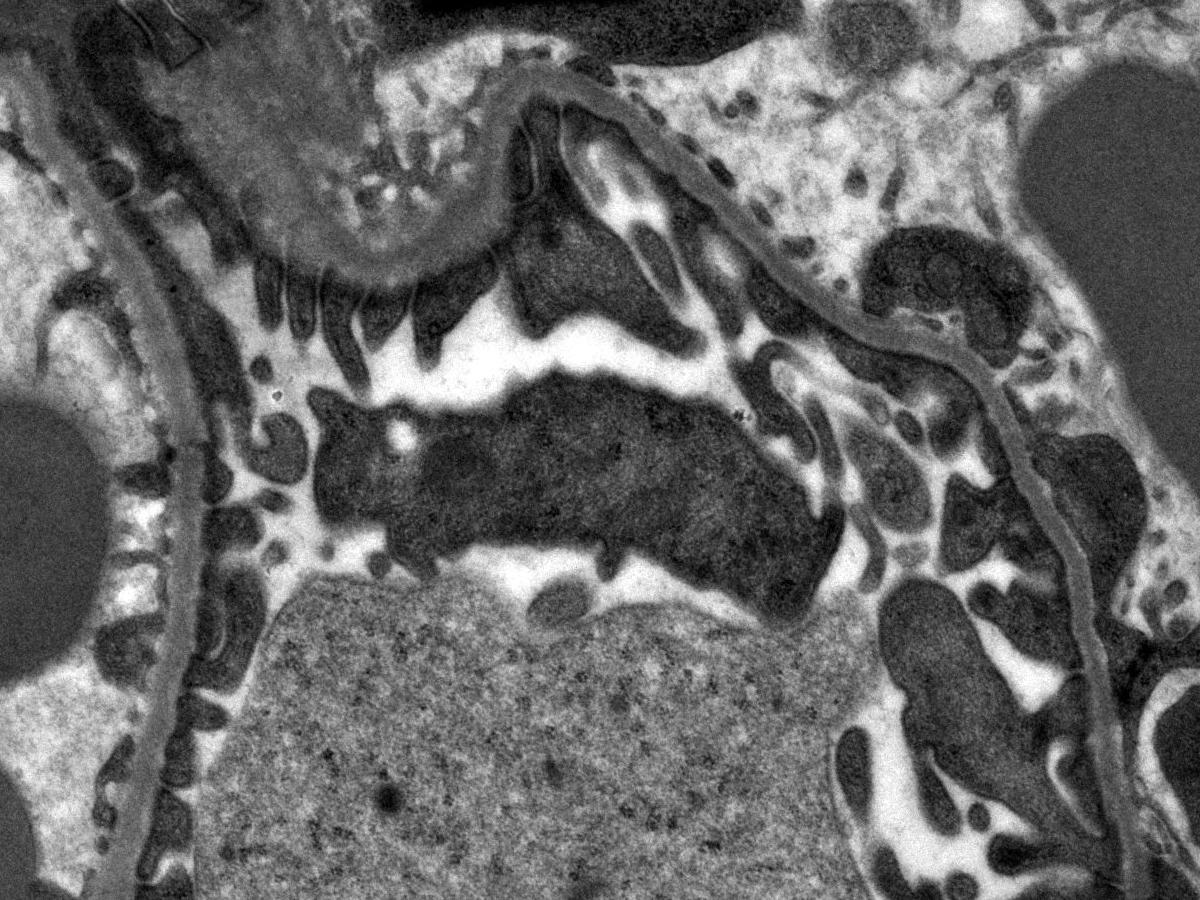

Supplement: Supplementary file 8 [file DataSheet7.zip › original images of figure 8/图8F-1.jpg]

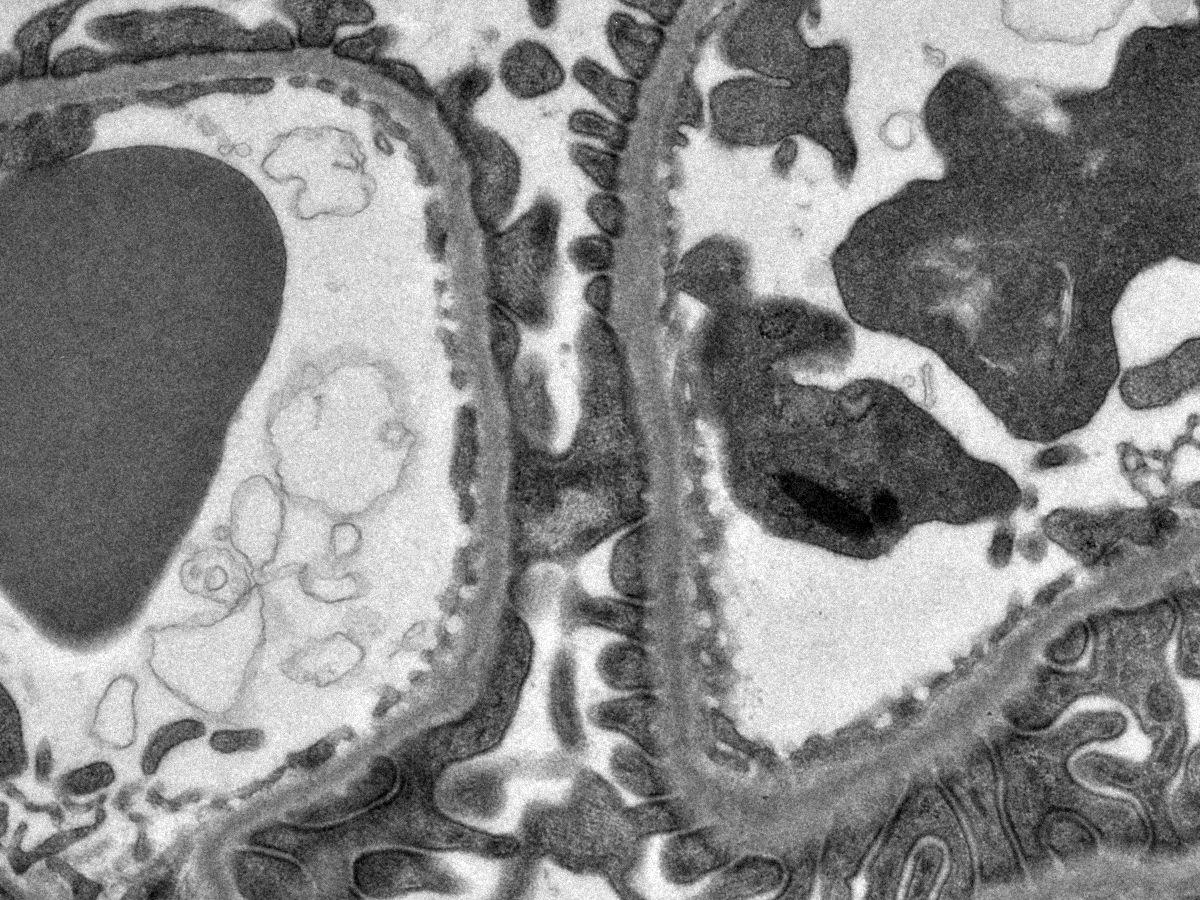

Supplement: Supplementary file 8 [file DataSheet7.zip › original images of figure 8/图8F-2.jpg]

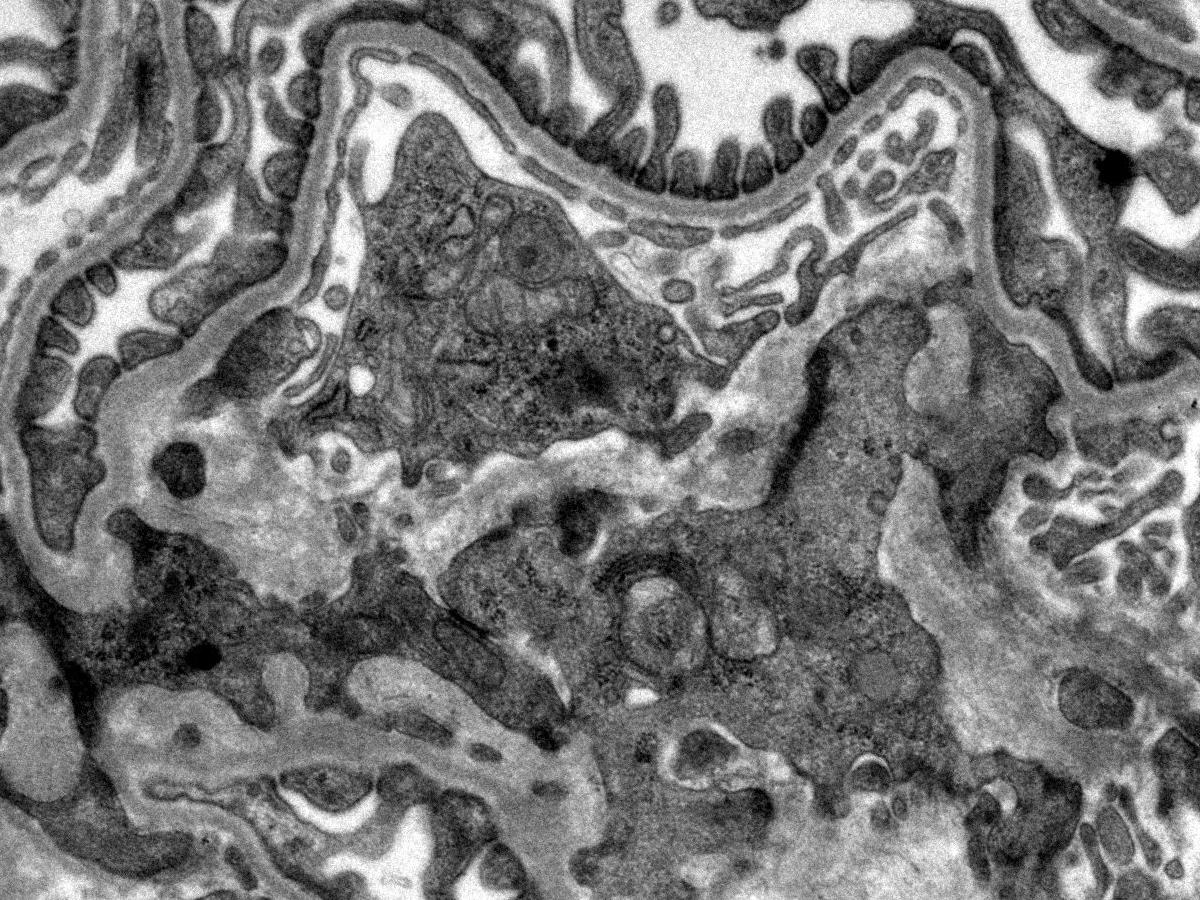

Supplement: Supplementary file 8 [file DataSheet7.zip › original images of figure 8/图8F-3.jpg]

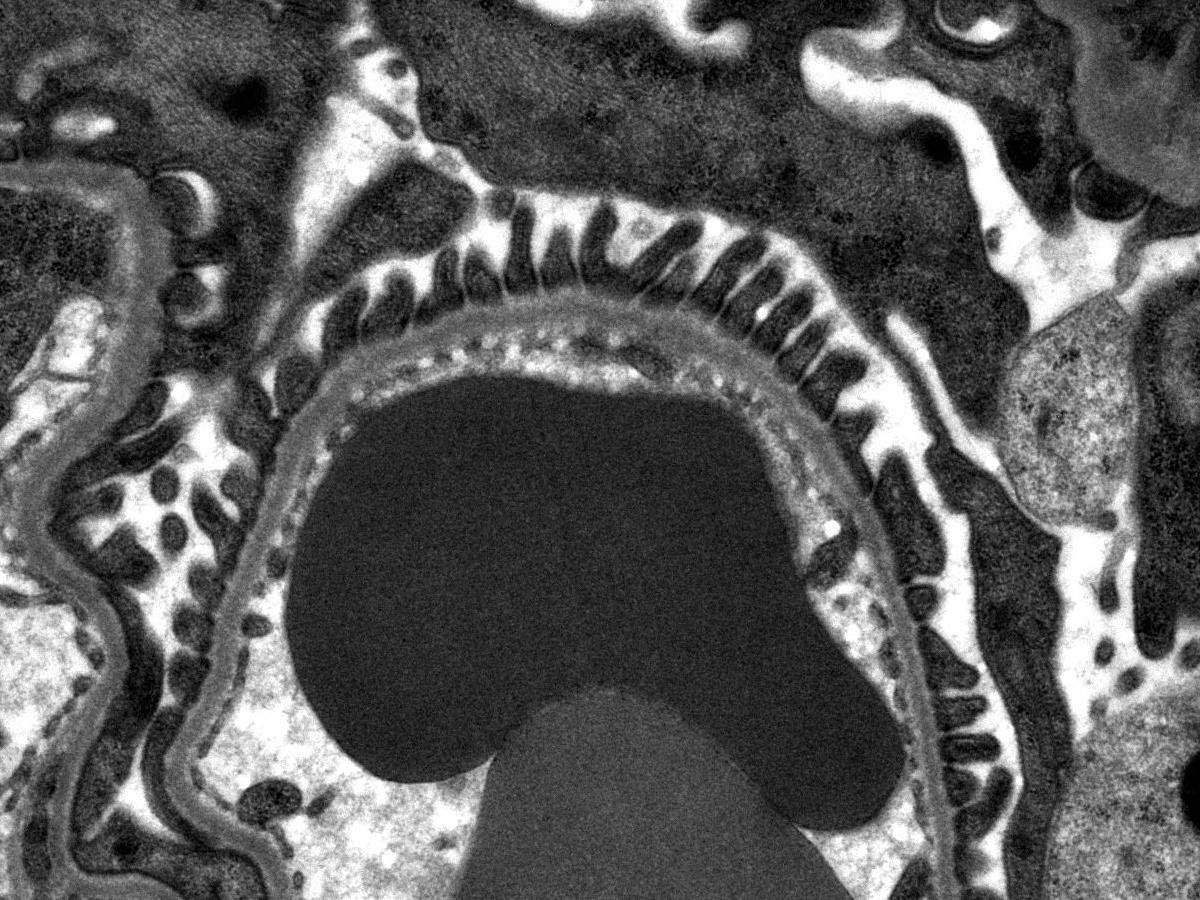

Supplement: Supplementary file 8 [file DataSheet7.zip › original images of figure 8/图8F-4.jpg]

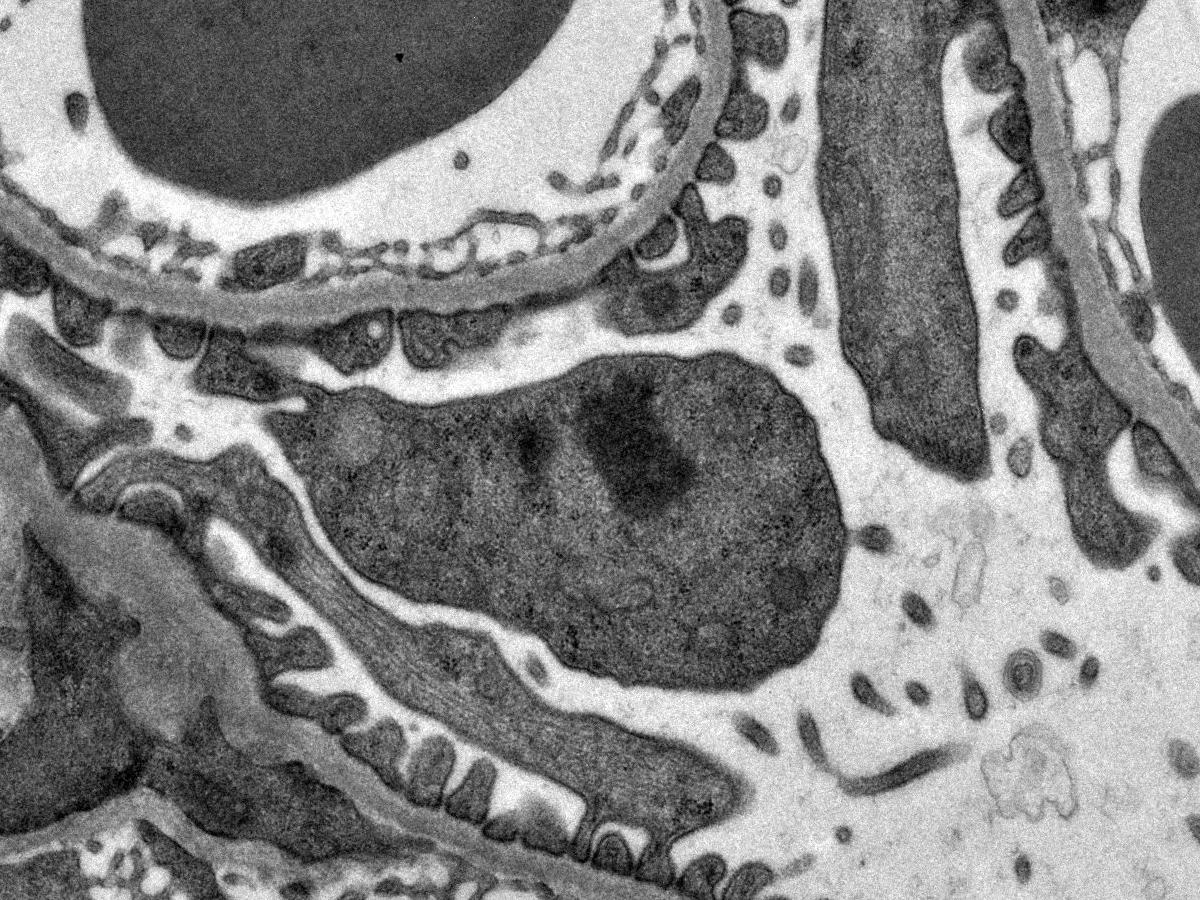

Supplement: Supplementary file 8 [file DataSheet7.zip › original images of figure 8/图8F-5.jpg]

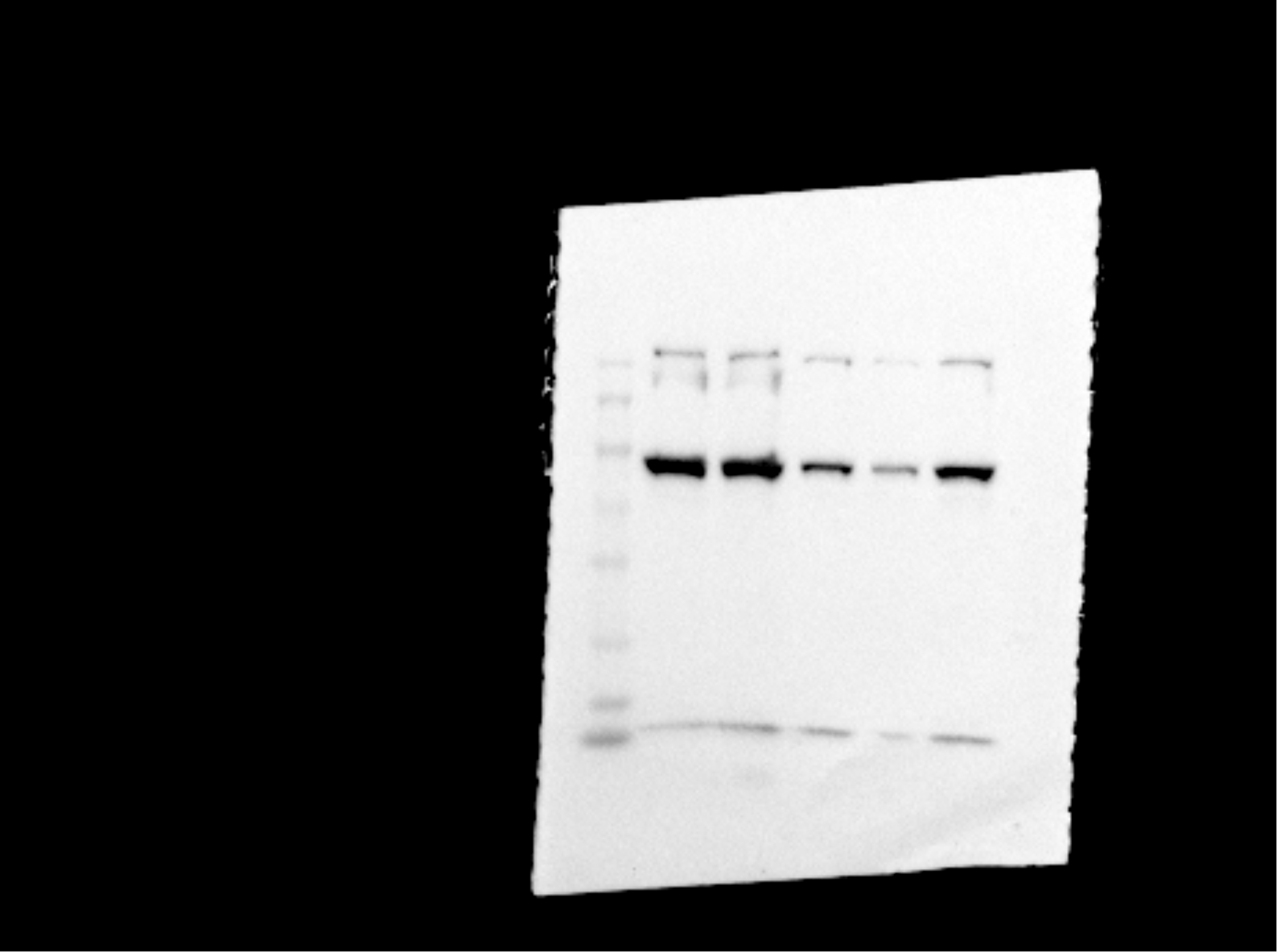

Supplement: Supplementary file 8 [file DataSheet7.zip › original images of figure 8/图8G-1.jpg]

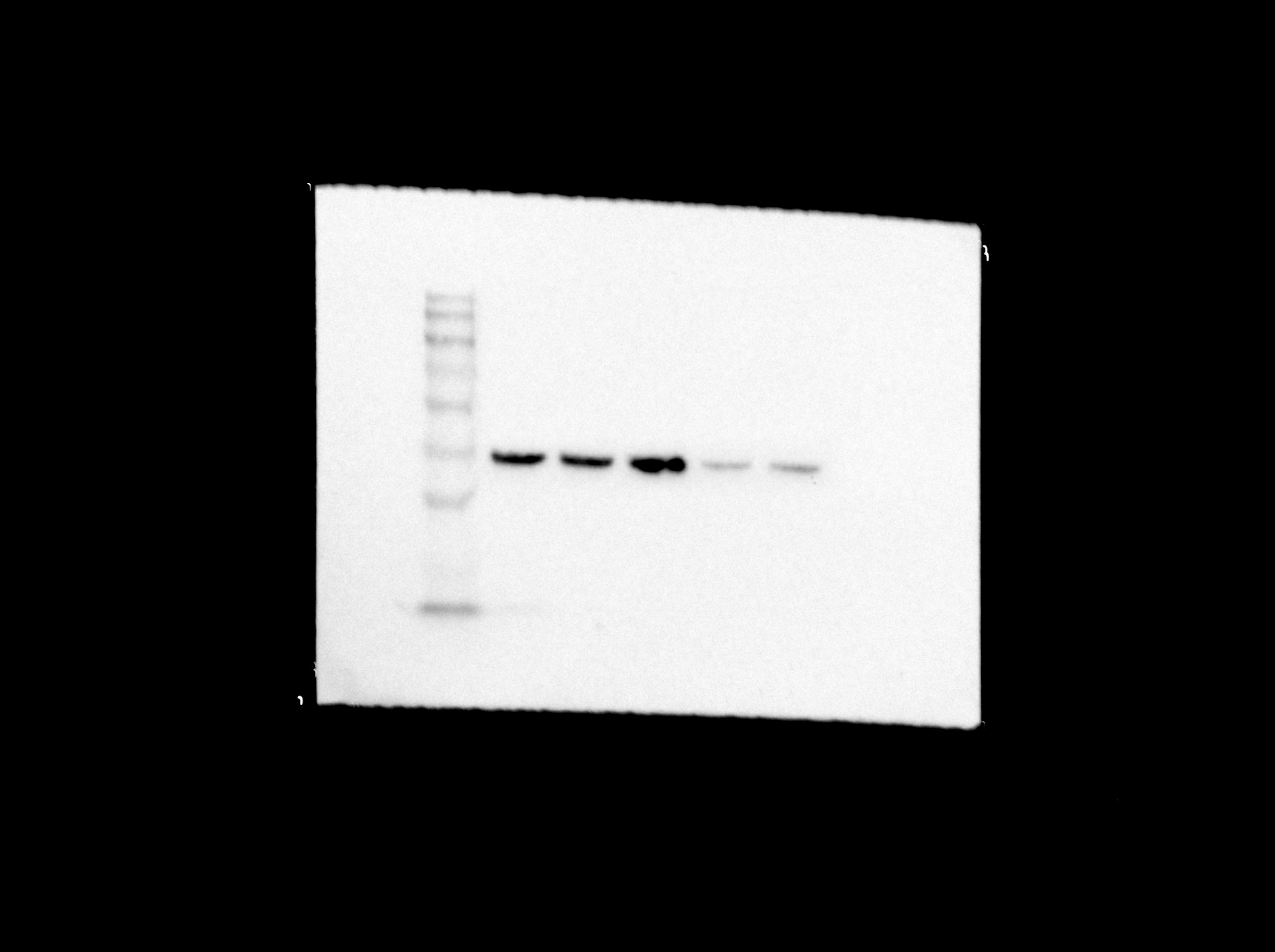

Supplement: Supplementary file 8 [file DataSheet7.zip › original images of figure 8/图8G-2.jpg]

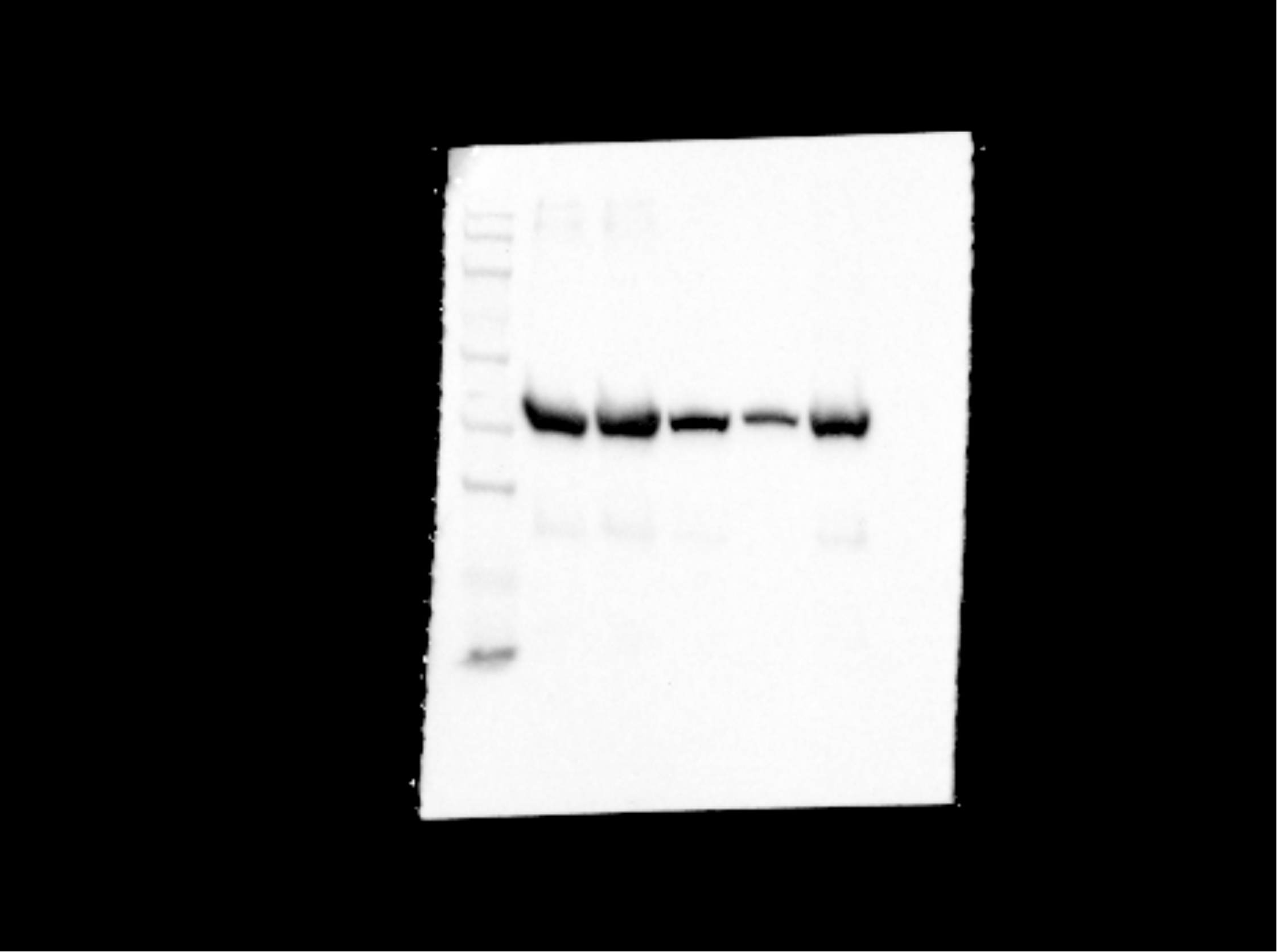

Supplement: Supplementary file 8 [file DataSheet7.zip › original images of figure 8/图8G-3.jpg]

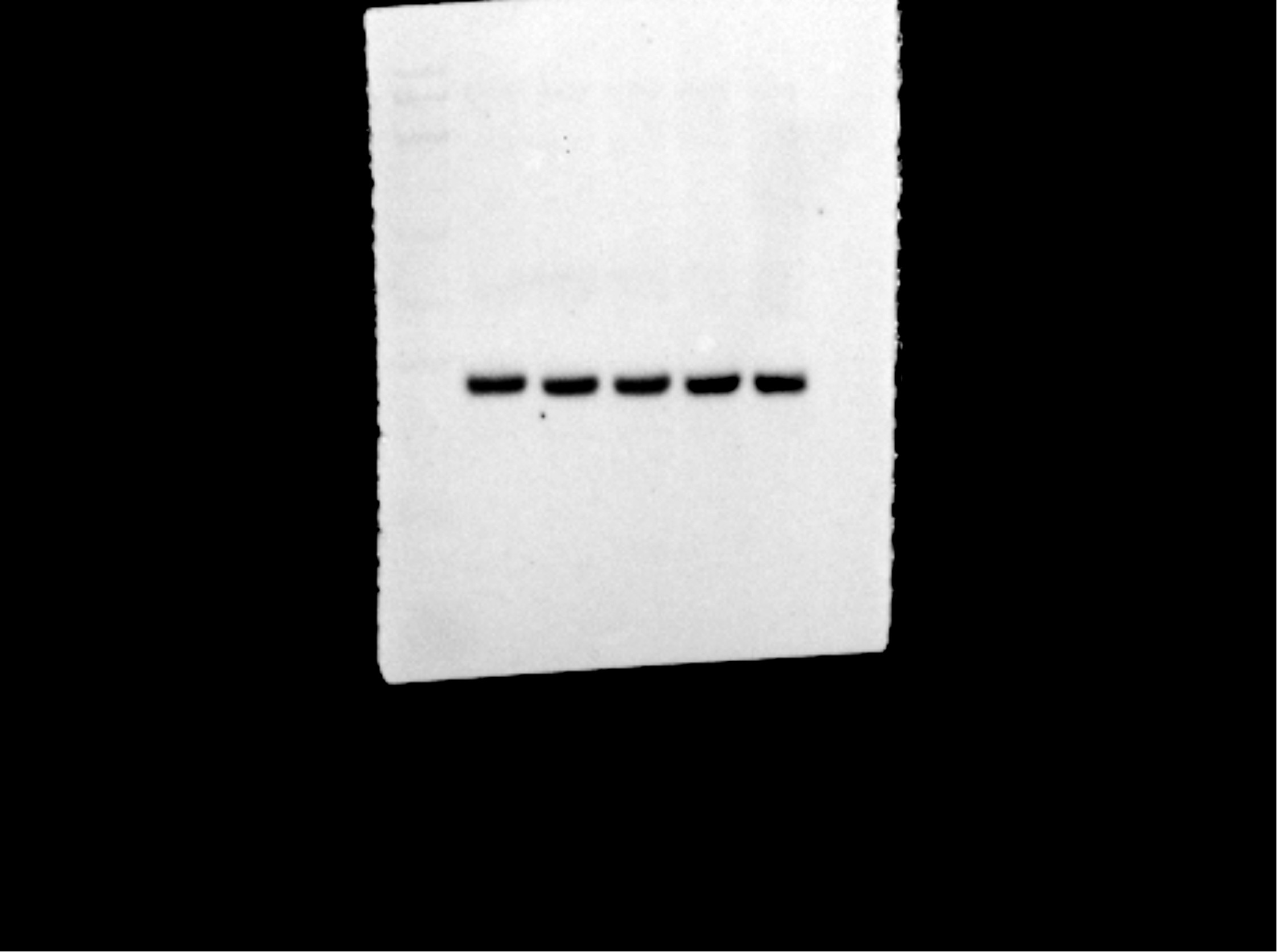

Supplement: Supplementary file 8 [file DataSheet7.zip › original images of figure 8/图8G-4.jpg]

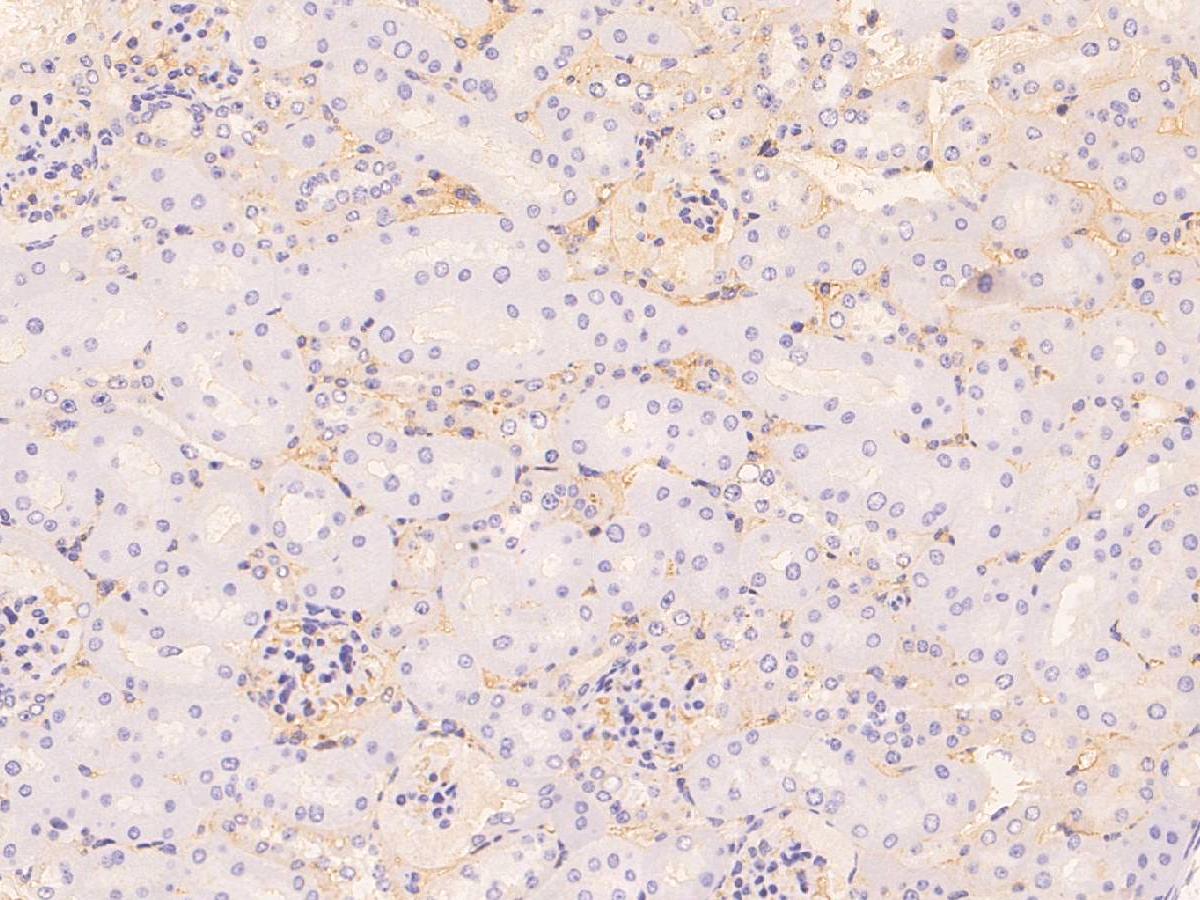

Supplement: Supplementary file 8 [file DataSheet7.zip › original images of figure 8/图8H-1-1.jpg]

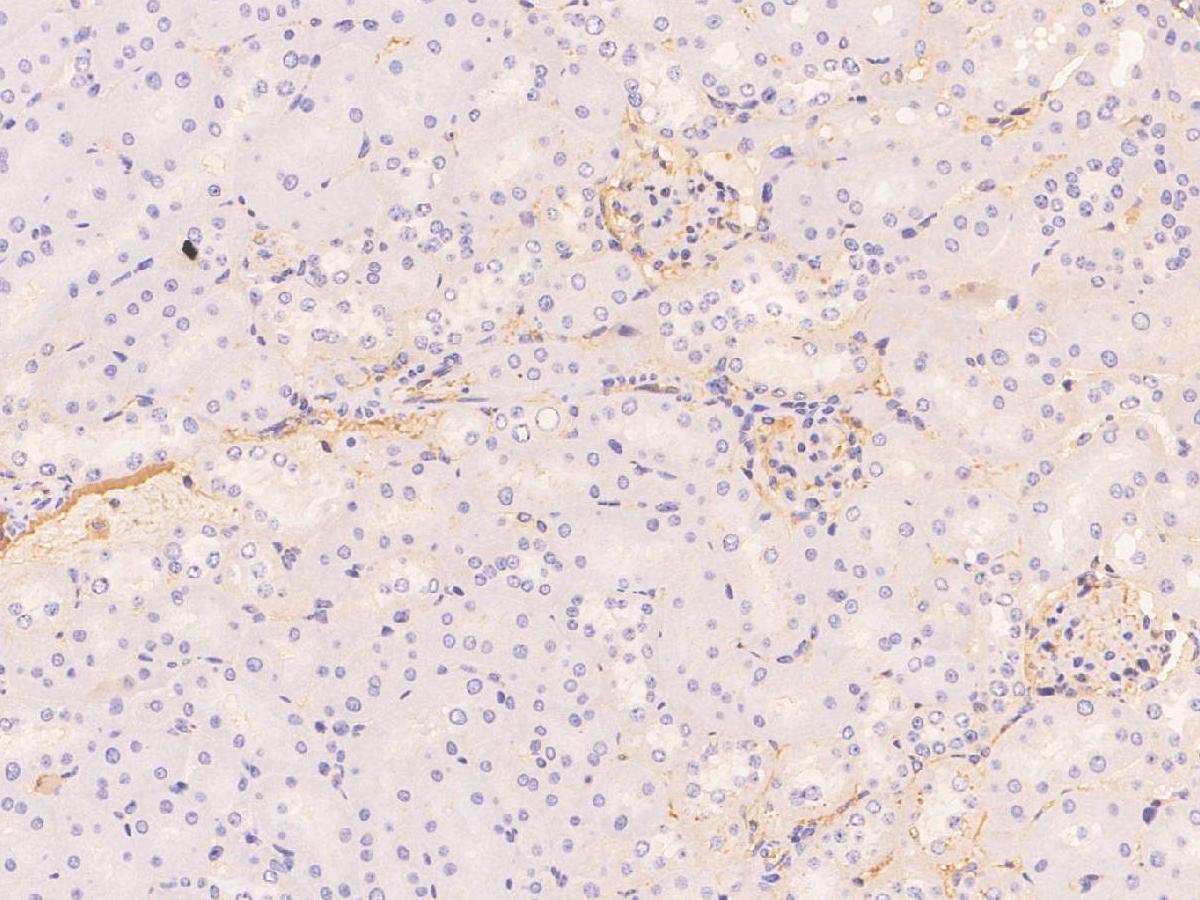

Supplement: Supplementary file 8 [file DataSheet7.zip › original images of figure 8/图8H-1-2 - 副本.jpg]

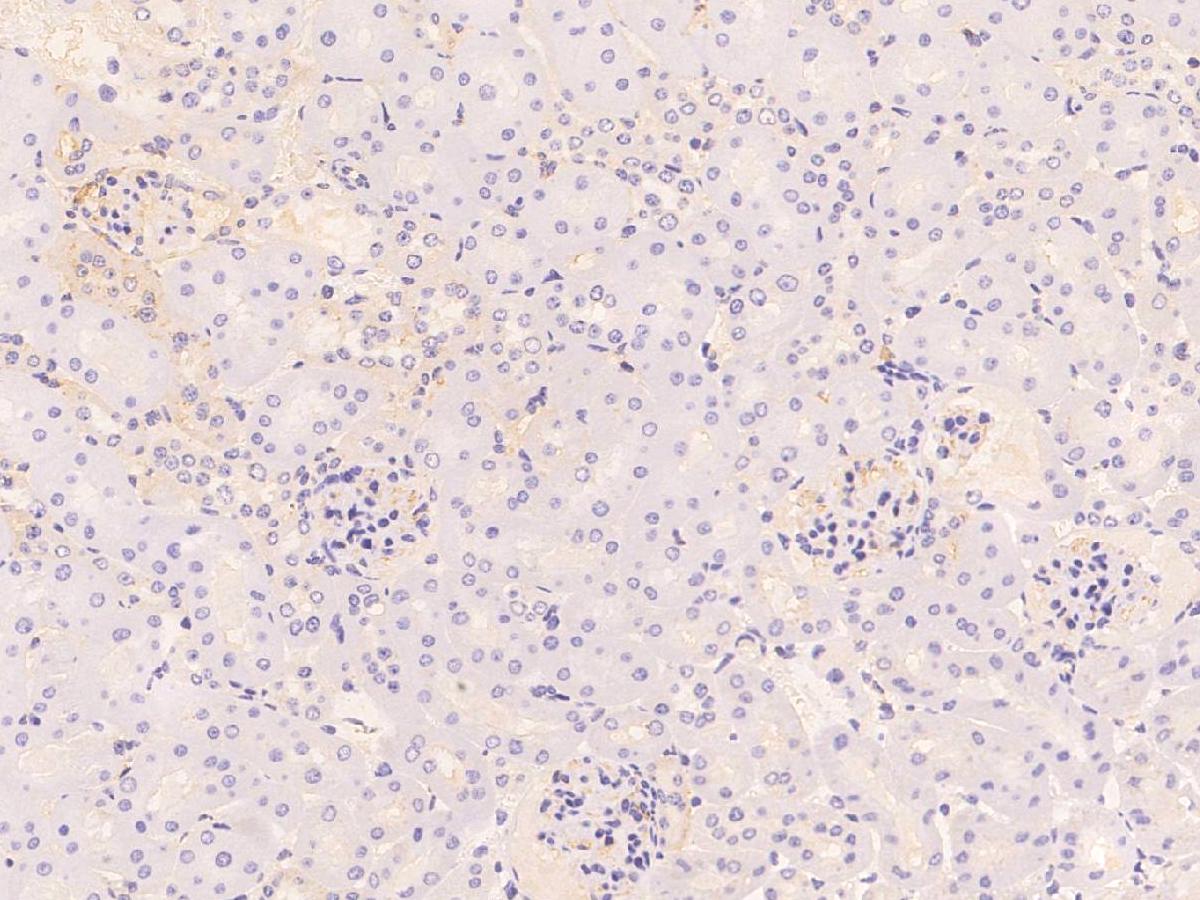

Supplement: Supplementary file 8 [file DataSheet7.zip › original images of figure 8/图8H-1-3.jpg]

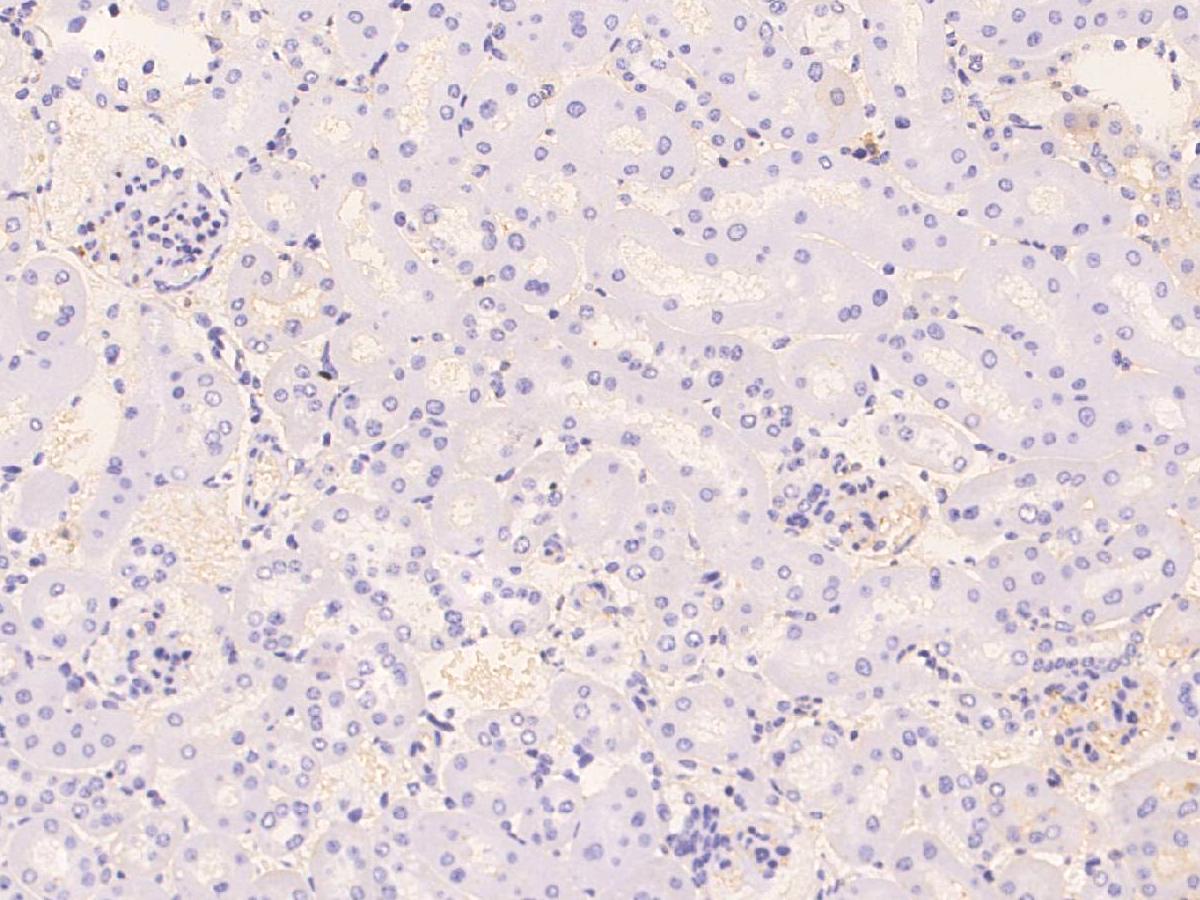

Supplement: Supplementary file 8 [file DataSheet7.zip › original images of figure 8/图8H-1-4.jpg]

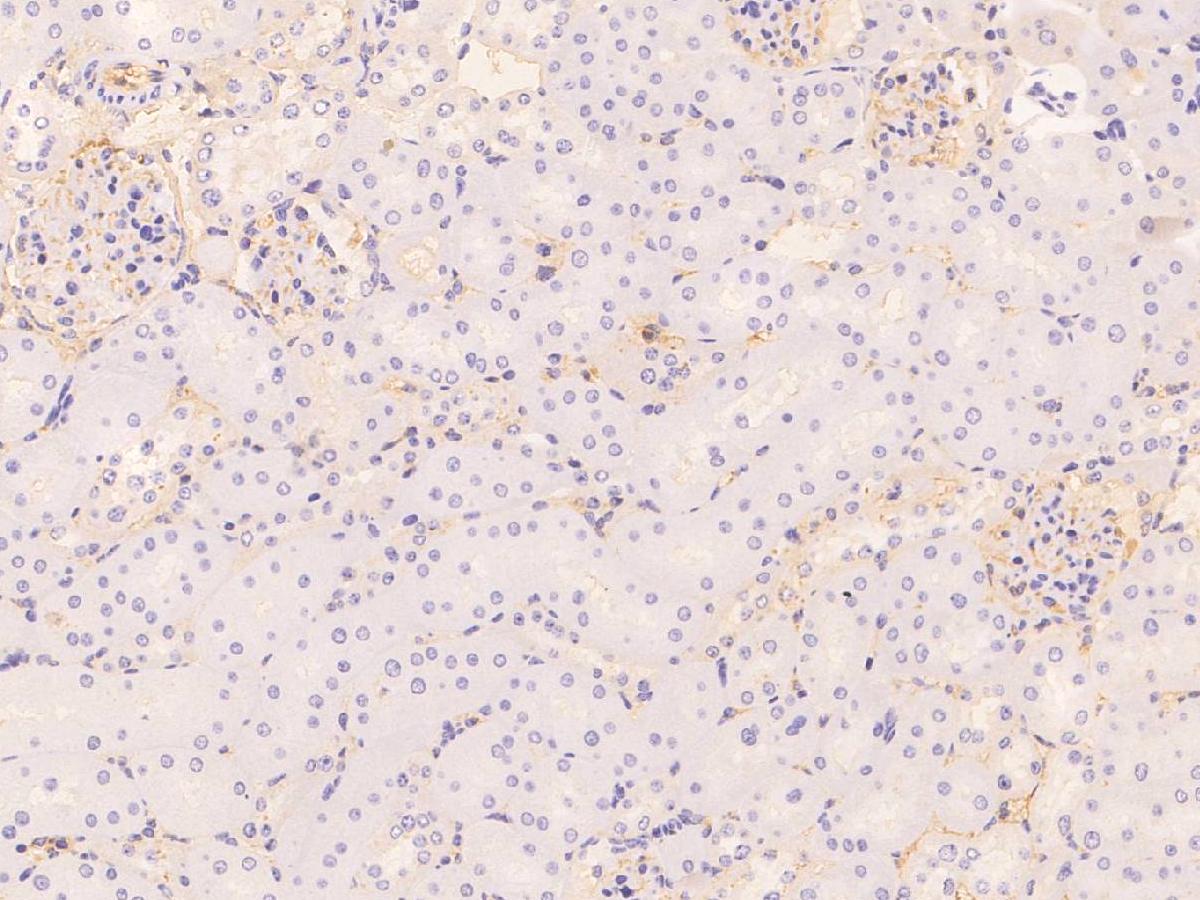

Supplement: Supplementary file 8 [file DataSheet7.zip › original images of figure 8/图8H-1-5.jpg]

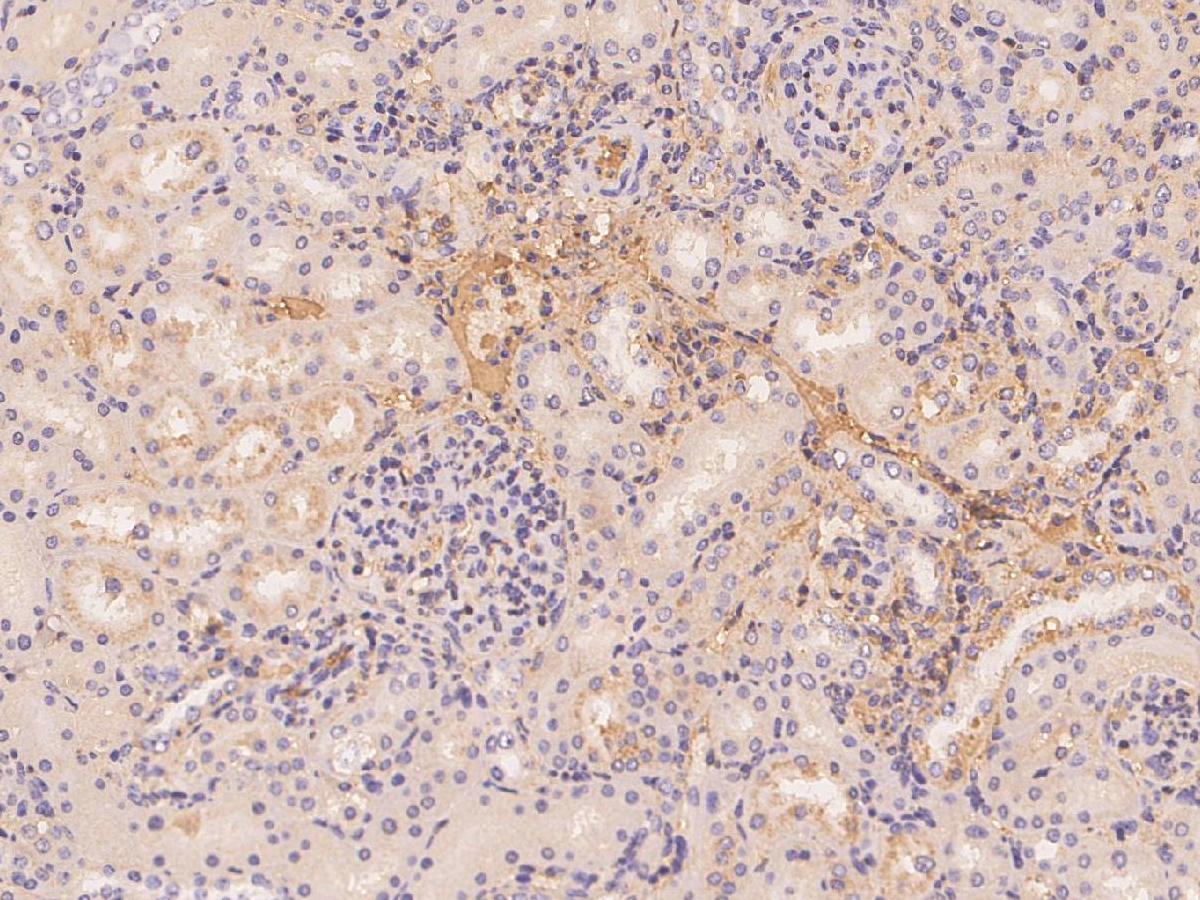

Supplement: Supplementary file 8 [file DataSheet7.zip › original images of figure 8/图8H-2-1.jpg]

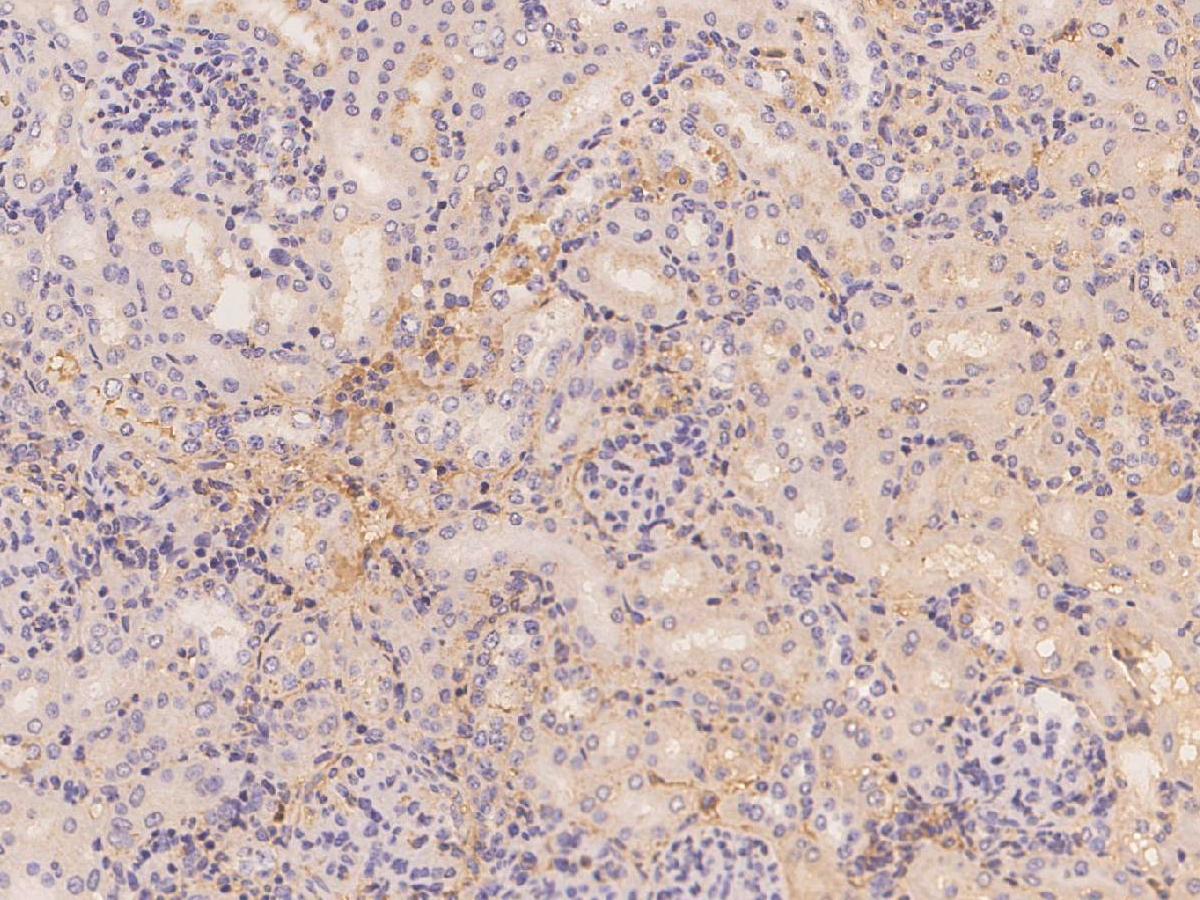

Supplement: Supplementary file 8 [file DataSheet7.zip › original images of figure 8/图8H-2-2.jpg]

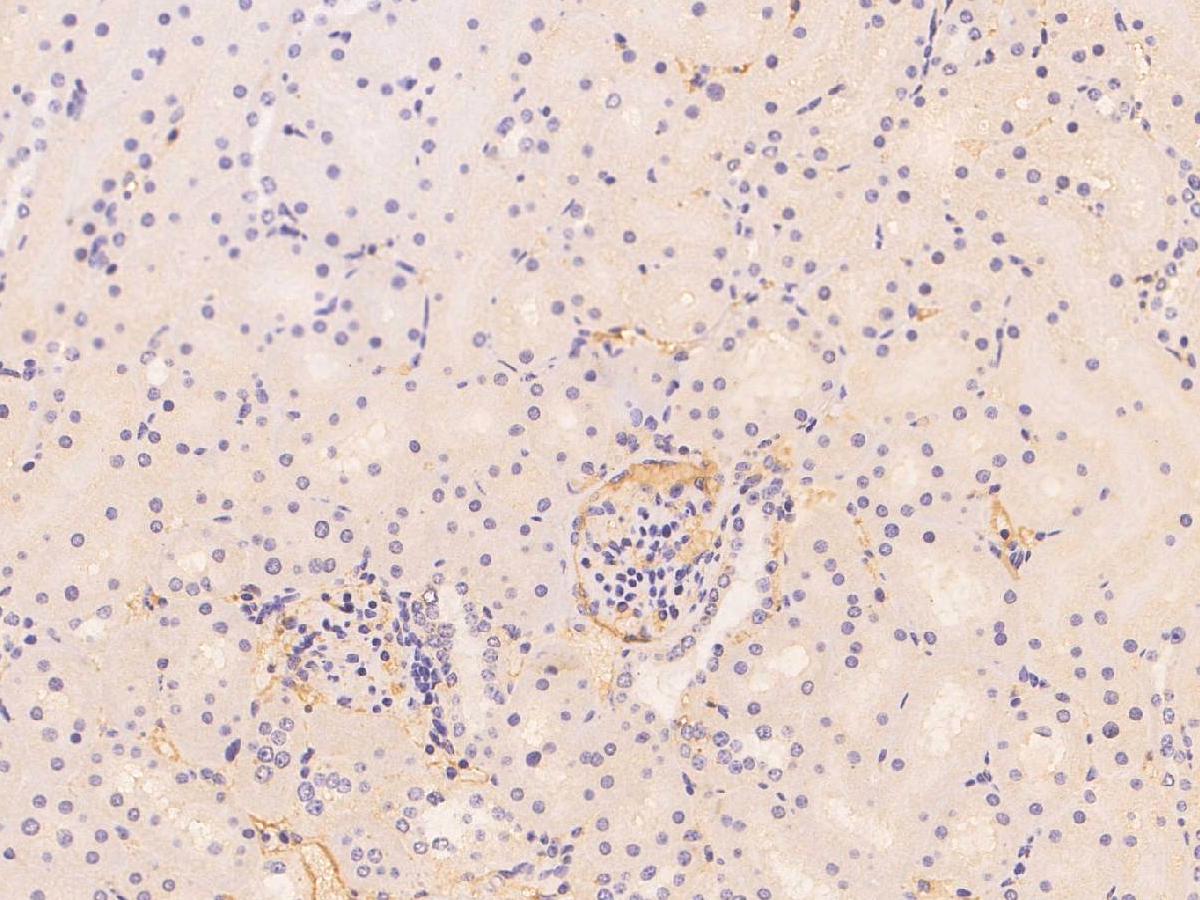

Supplement: Supplementary file 8 [file DataSheet7.zip › original images of figure 8/图8H-2-3.jpg]

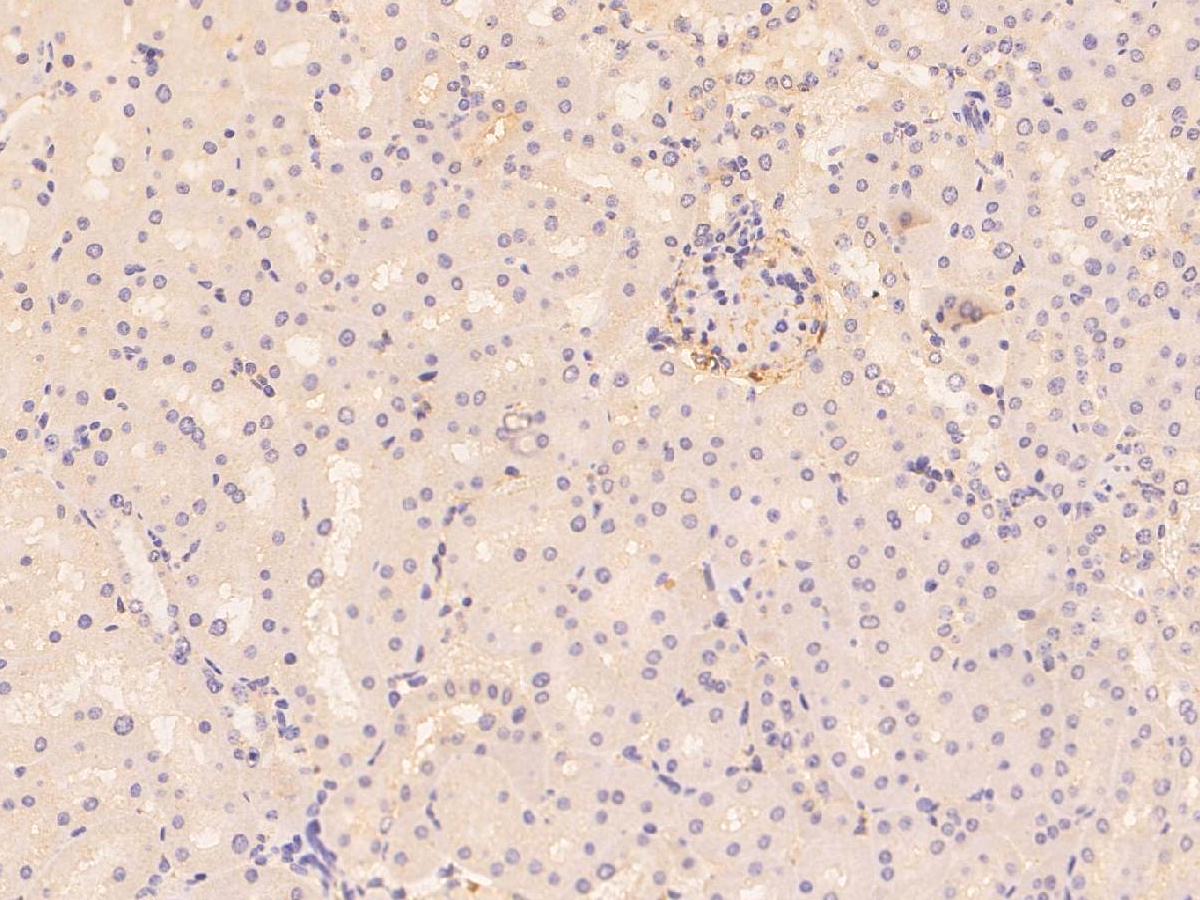

Supplement: Supplementary file 8 [file DataSheet7.zip › original images of figure 8/图8H-2-4.jpg]

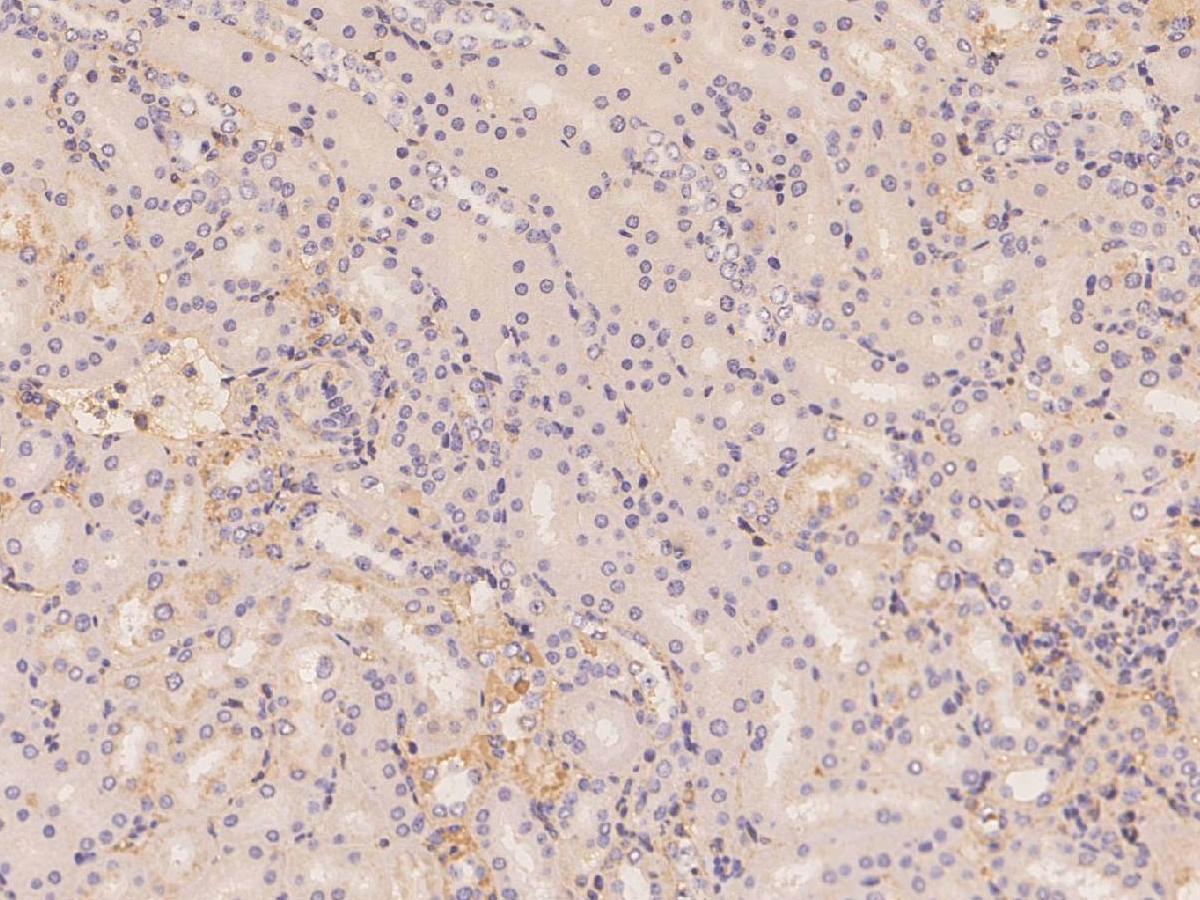

Supplement: Supplementary file 8 [file DataSheet7.zip › original images of figure 8/图8H-2-5.jpg]

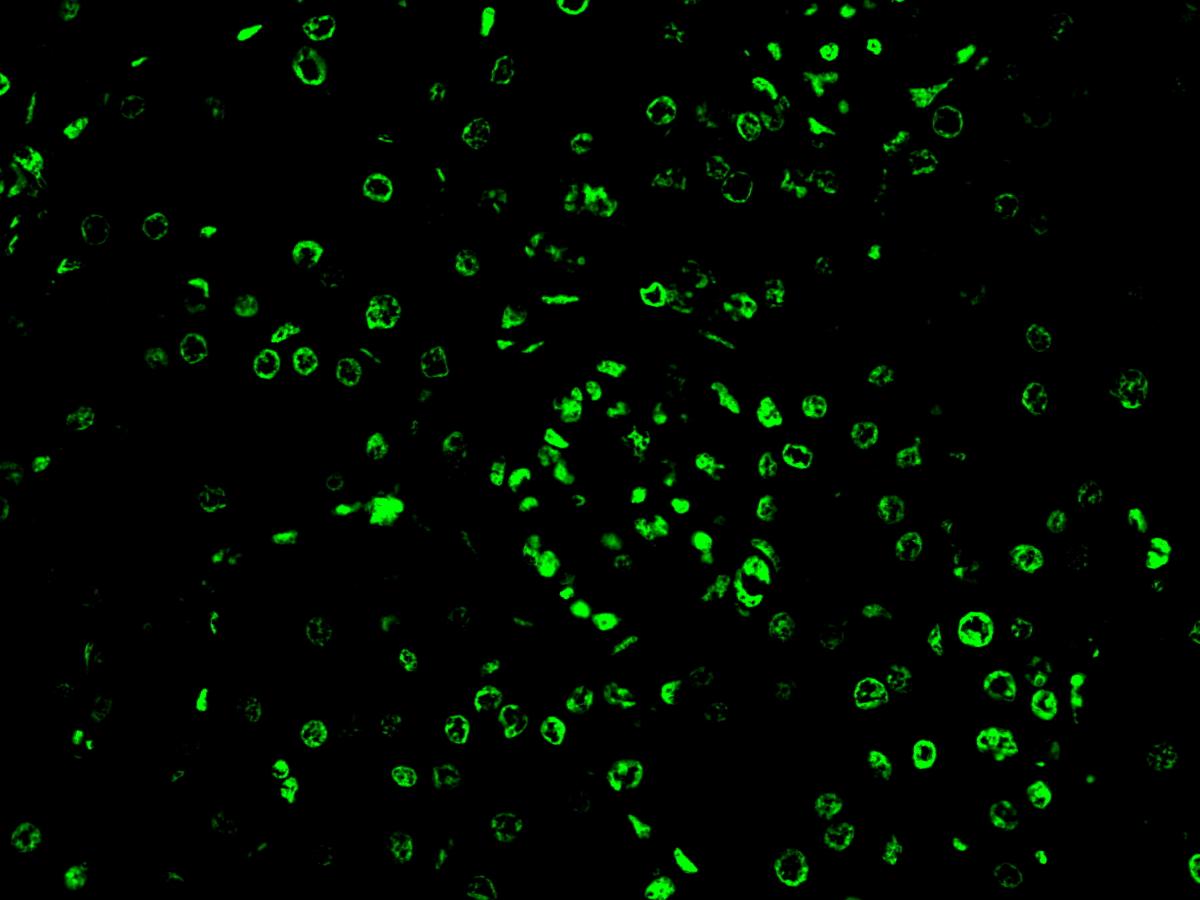

Supplement: Supplementary file 8 [file DataSheet7.zip › original images of figure 8/图8I-1-1(PARP1).jpg]

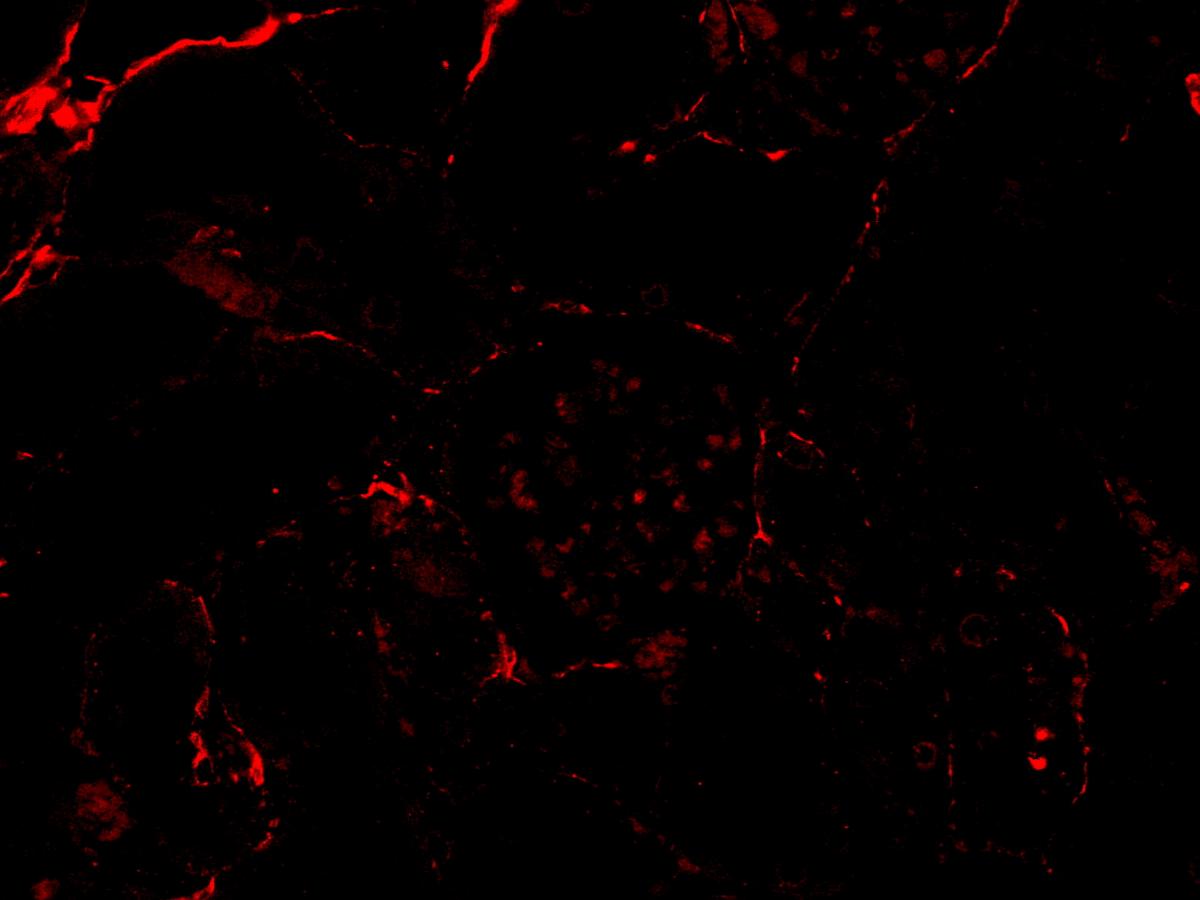

Supplement: Supplementary file 8 [file DataSheet7.zip › original images of figure 8/图8I-1-2(Synaptopodin).jpg]

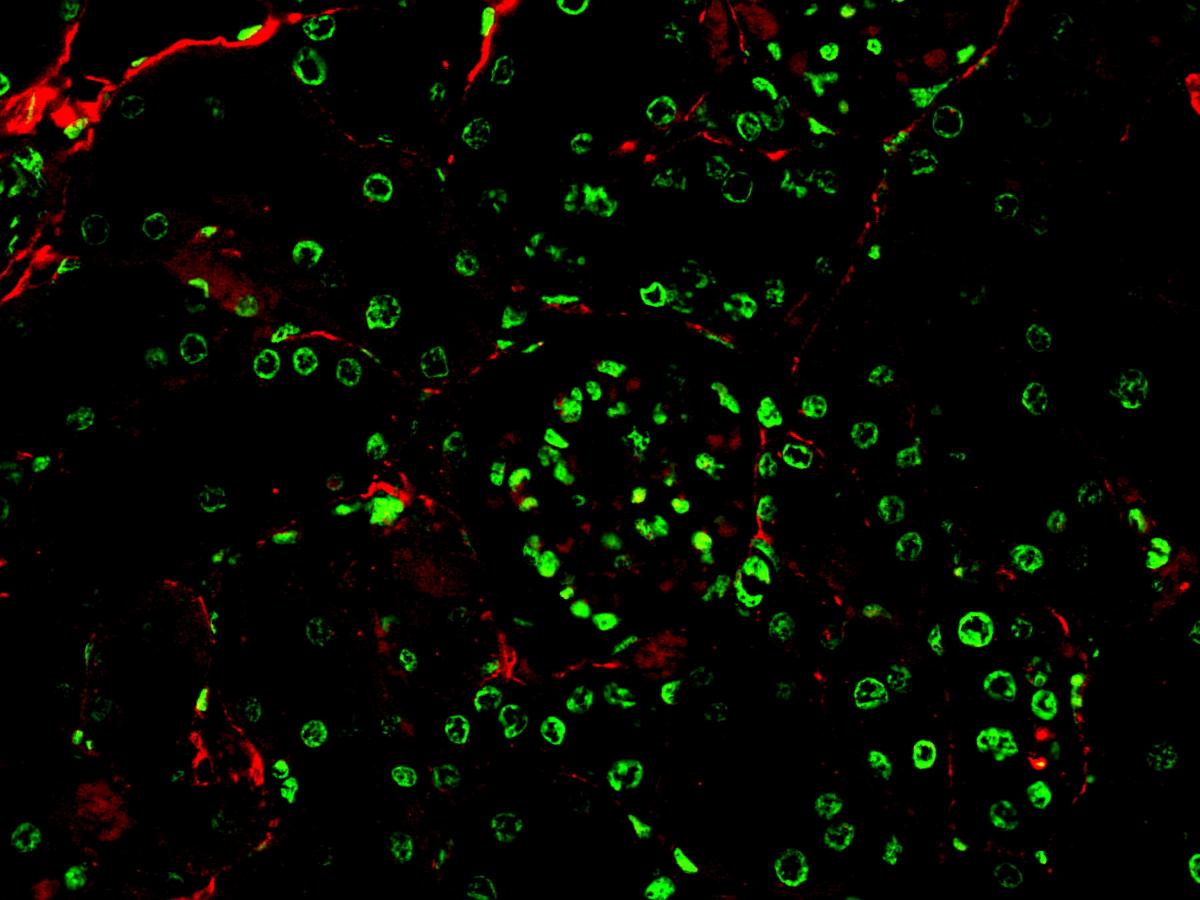

Supplement: Supplementary file 8 [file DataSheet7.zip › original images of figure 8/图8I-1-3(Merge).jpg]

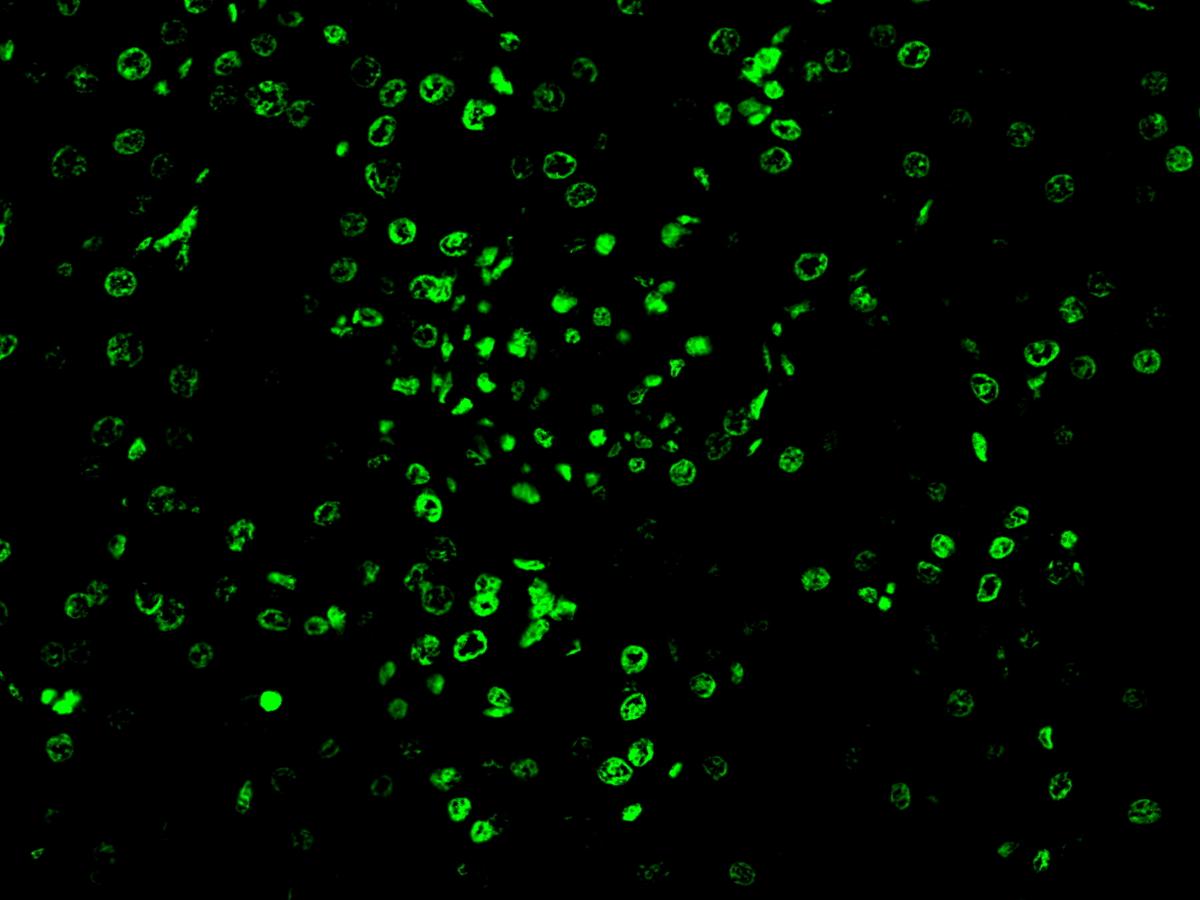

Supplement: Supplementary file 8 [file DataSheet7.zip › original images of figure 8/图8I-2-1(PARP1).jpg]

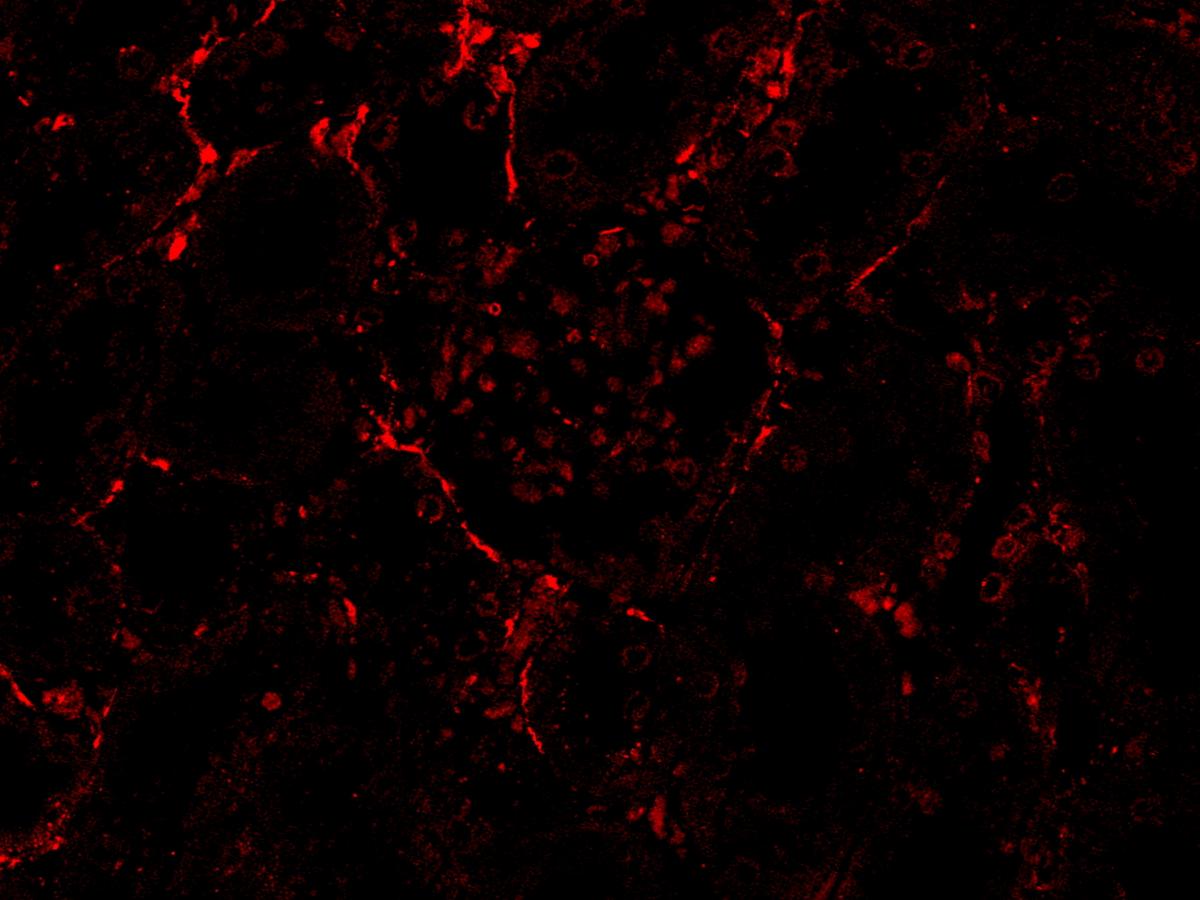

Supplement: Supplementary file 8 [file DataSheet7.zip › original images of figure 8/图8I-2-2(Synaptopodin).jpg]

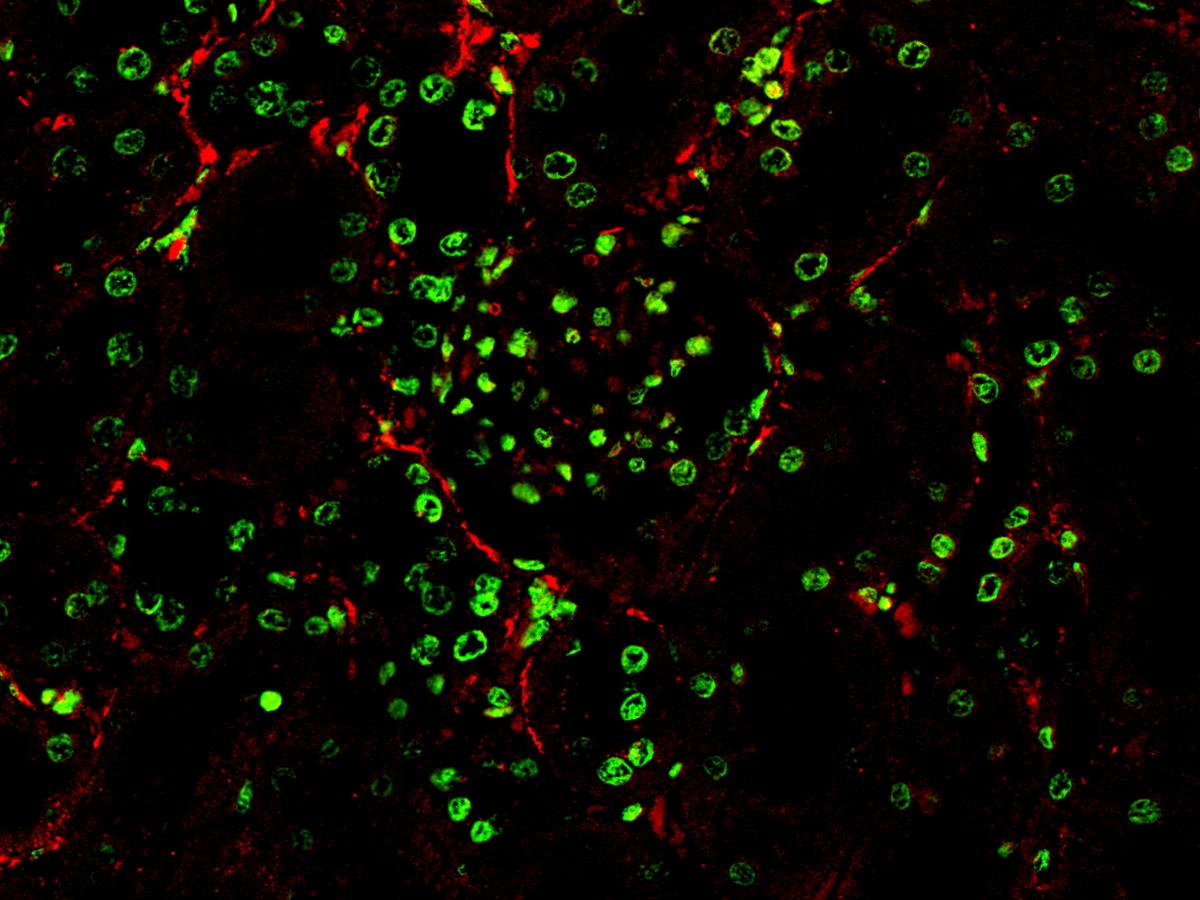

Supplement: Supplementary file 8 [file DataSheet7.zip › original images of figure 8/图8I-2-3(Merge).jpg]

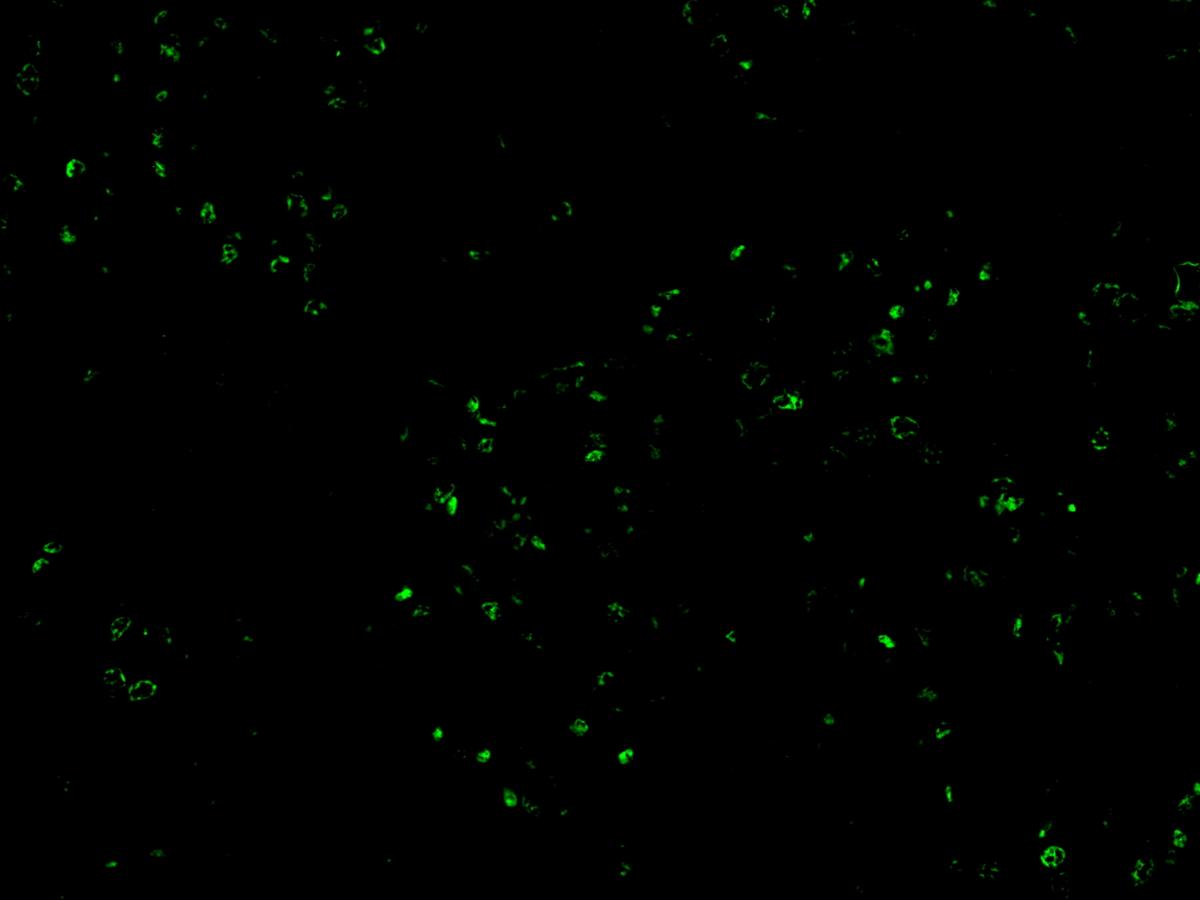

Supplement: Supplementary file 8 [file DataSheet7.zip › original images of figure 8/图8I-3-1(PARP1).jpg]

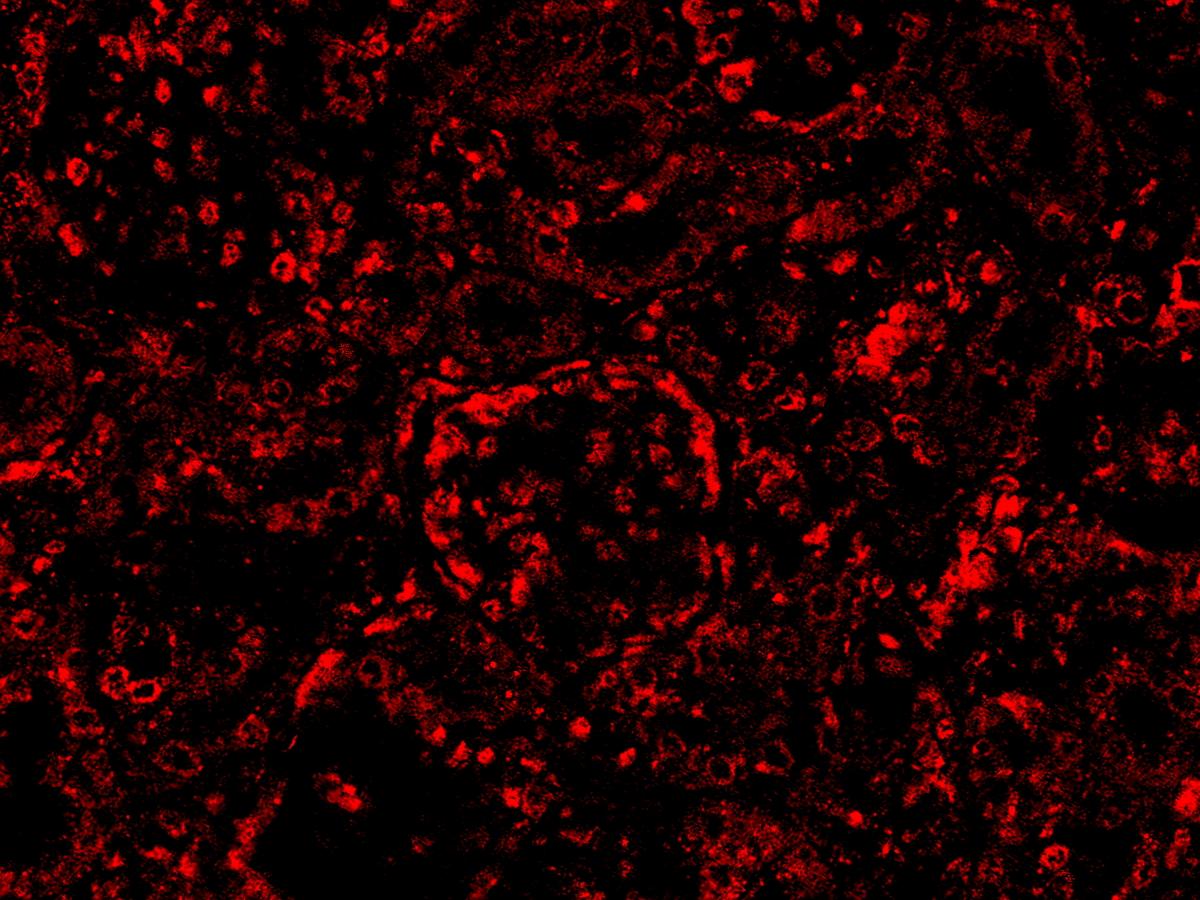

Supplement: Supplementary file 8 [file DataSheet7.zip › original images of figure 8/图8I-3-2(Synaptopodin).jpg]

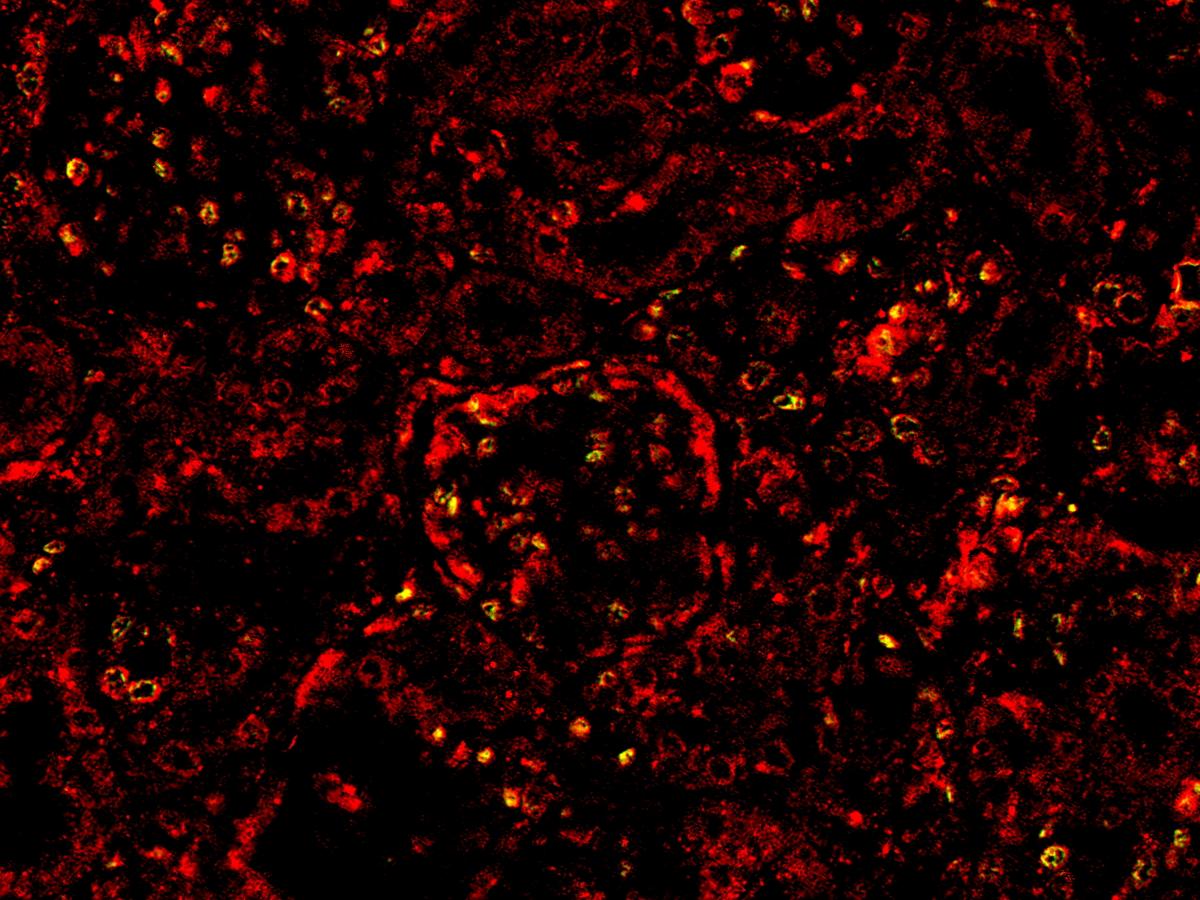

Supplement: Supplementary file 8 [file DataSheet7.zip › original images of figure 8/图8I-3-3(Merge).jpg]

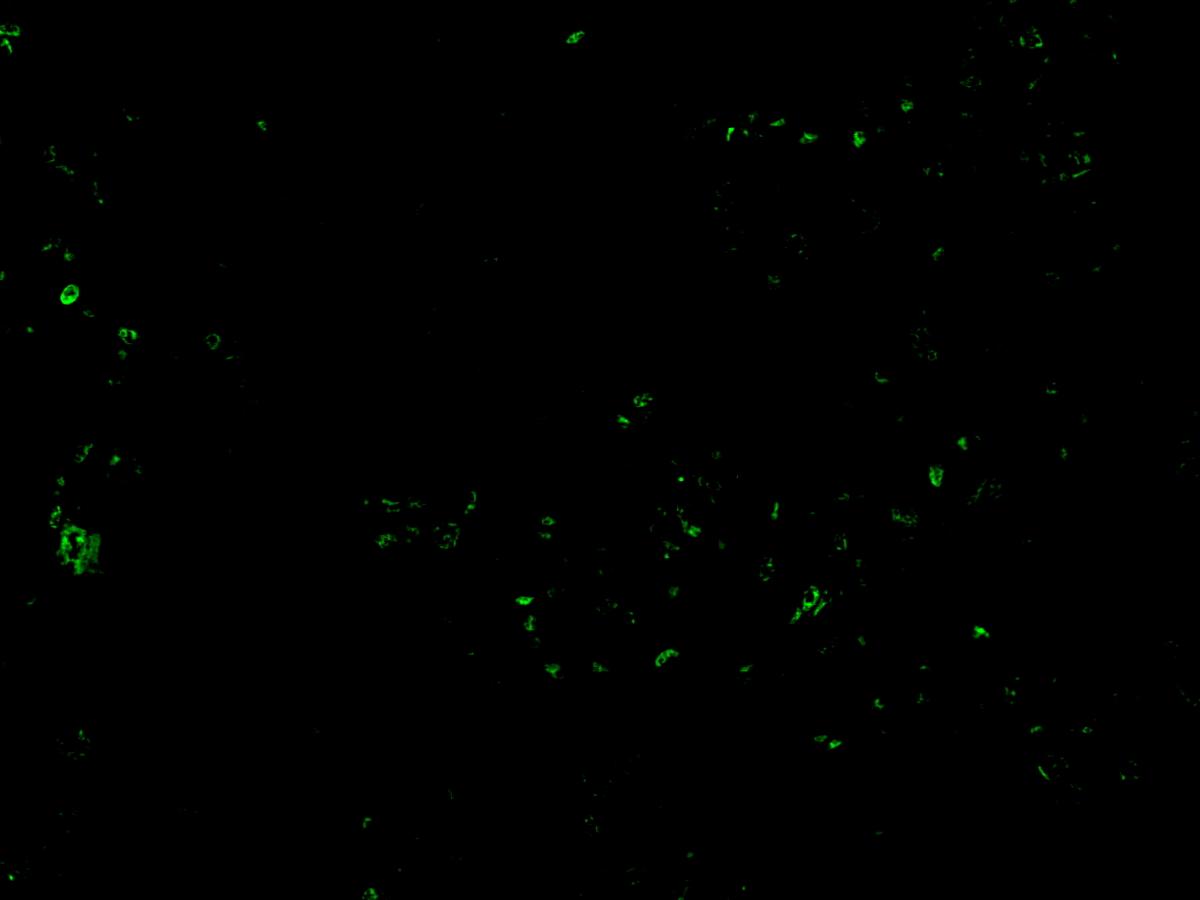

Supplement: Supplementary file 8 [file DataSheet7.zip › original images of figure 8/图8I-4-1(PARP1).jpg]

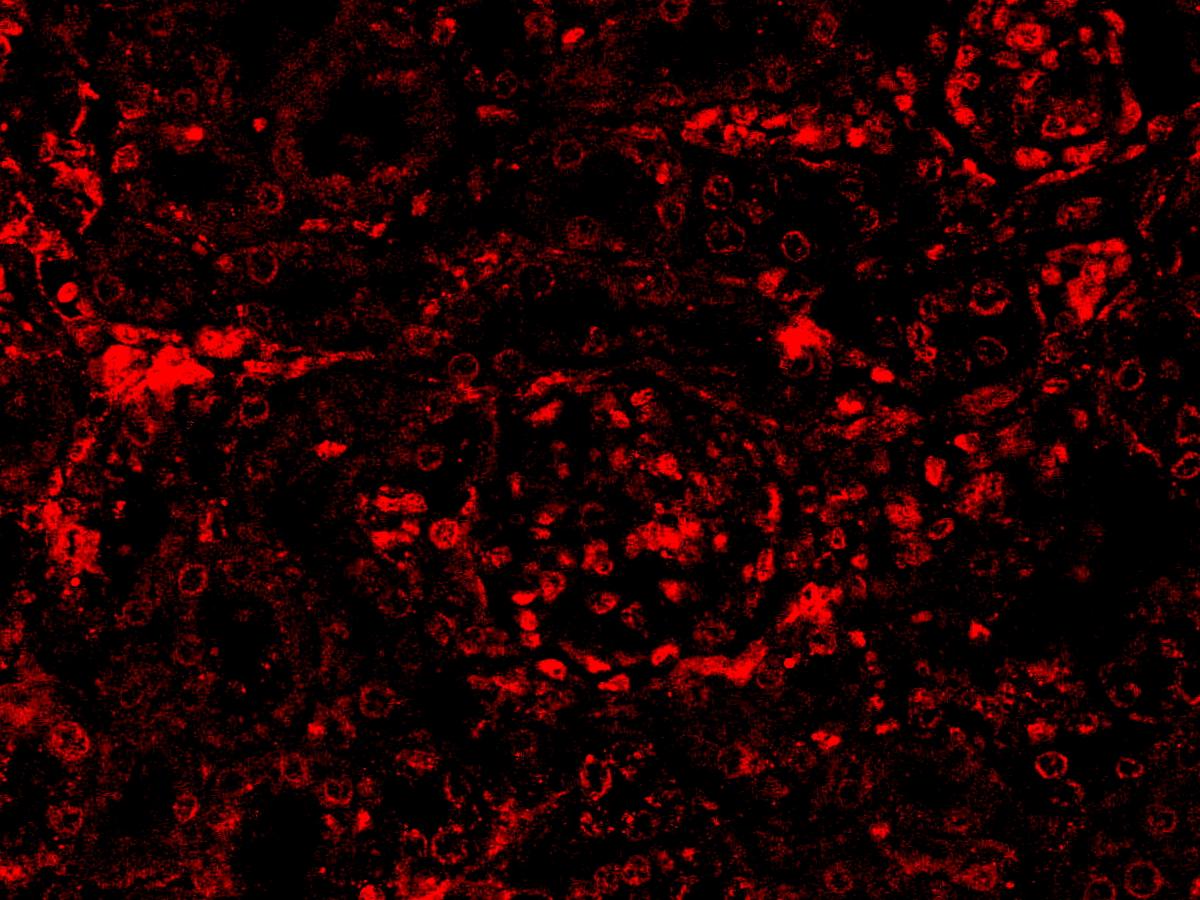

Supplement: Supplementary file 8 [file DataSheet7.zip › original images of figure 8/图8I-4-2(Synaptopodin).jpg]

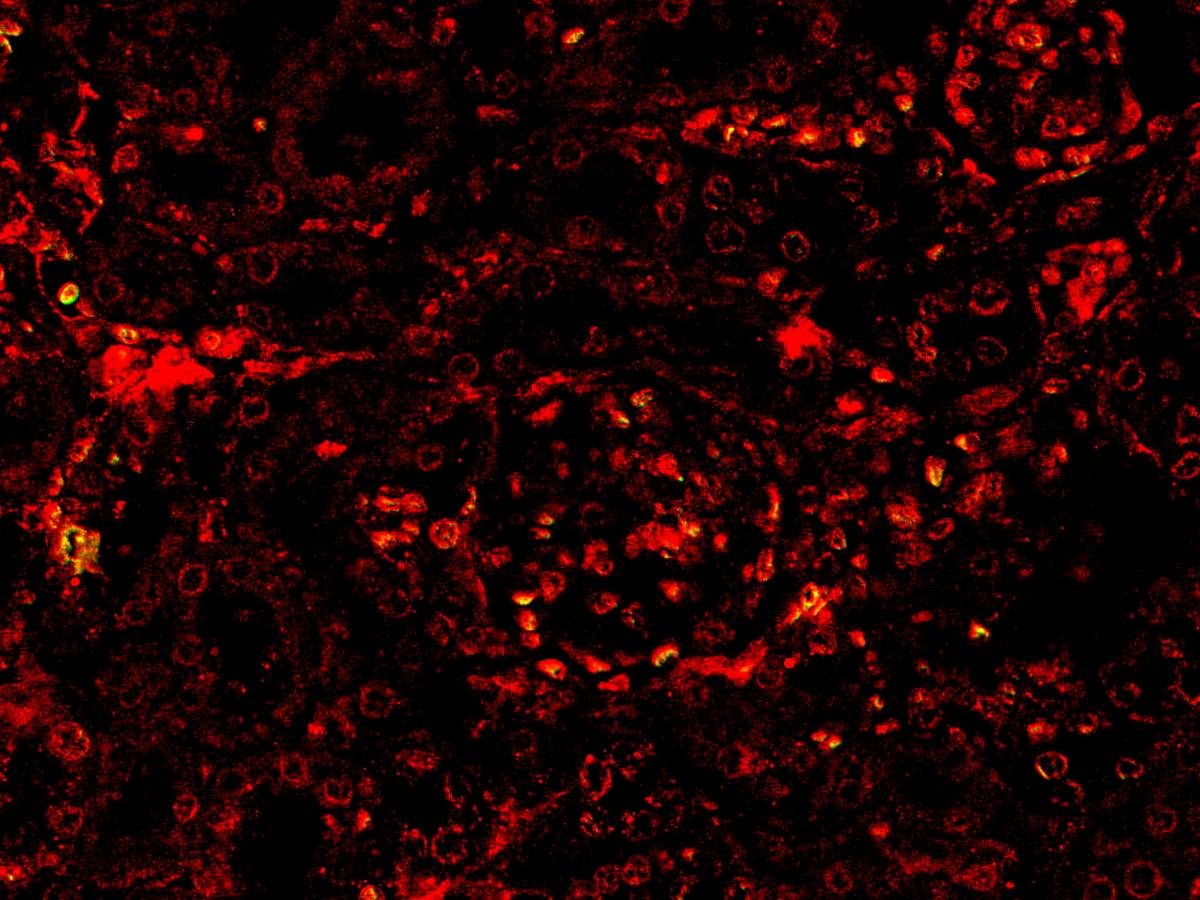

Supplement: Supplementary file 8 [file DataSheet7.zip › original images of figure 8/图8I-4-3(Merge).jpg]

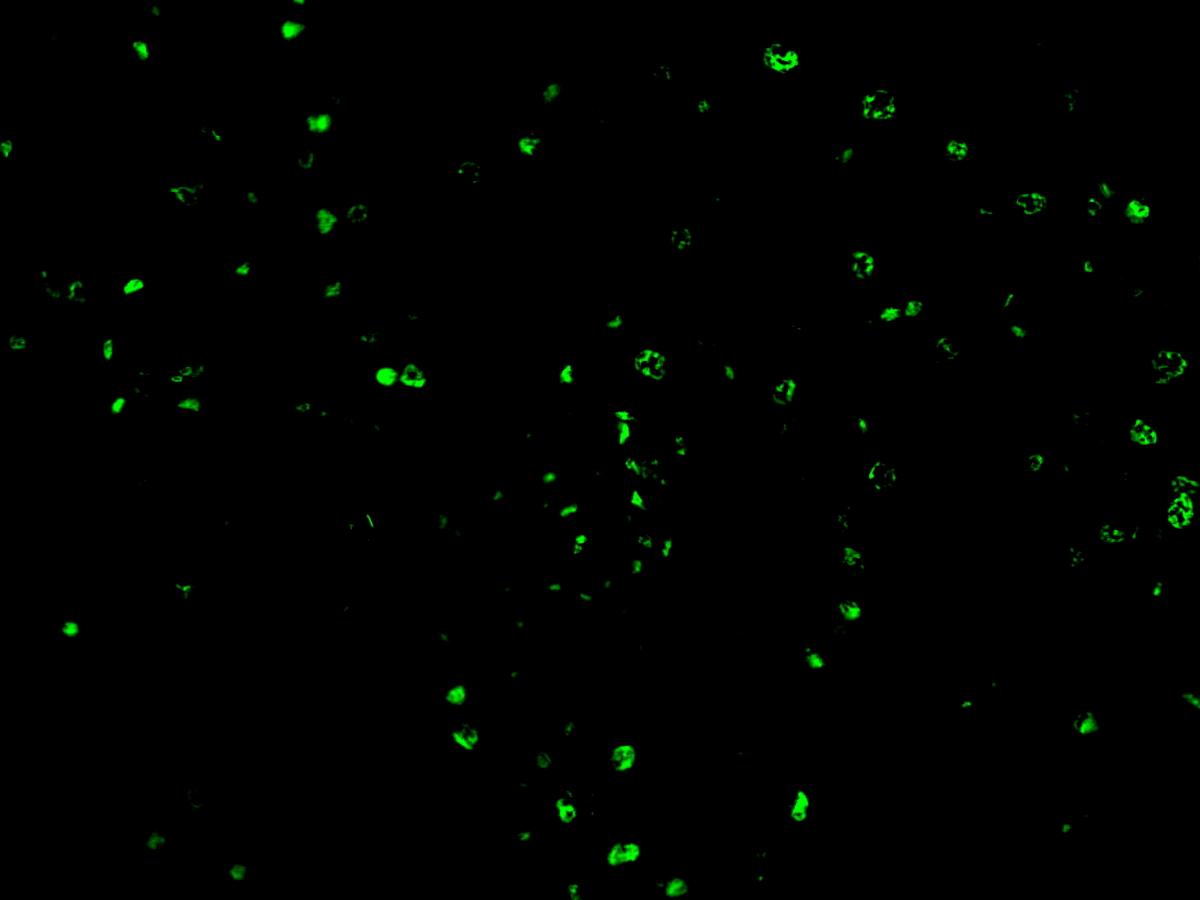

Supplement: Supplementary file 8 [file DataSheet7.zip › original images of figure 8/图8I-5-1(PARP1).jpg]

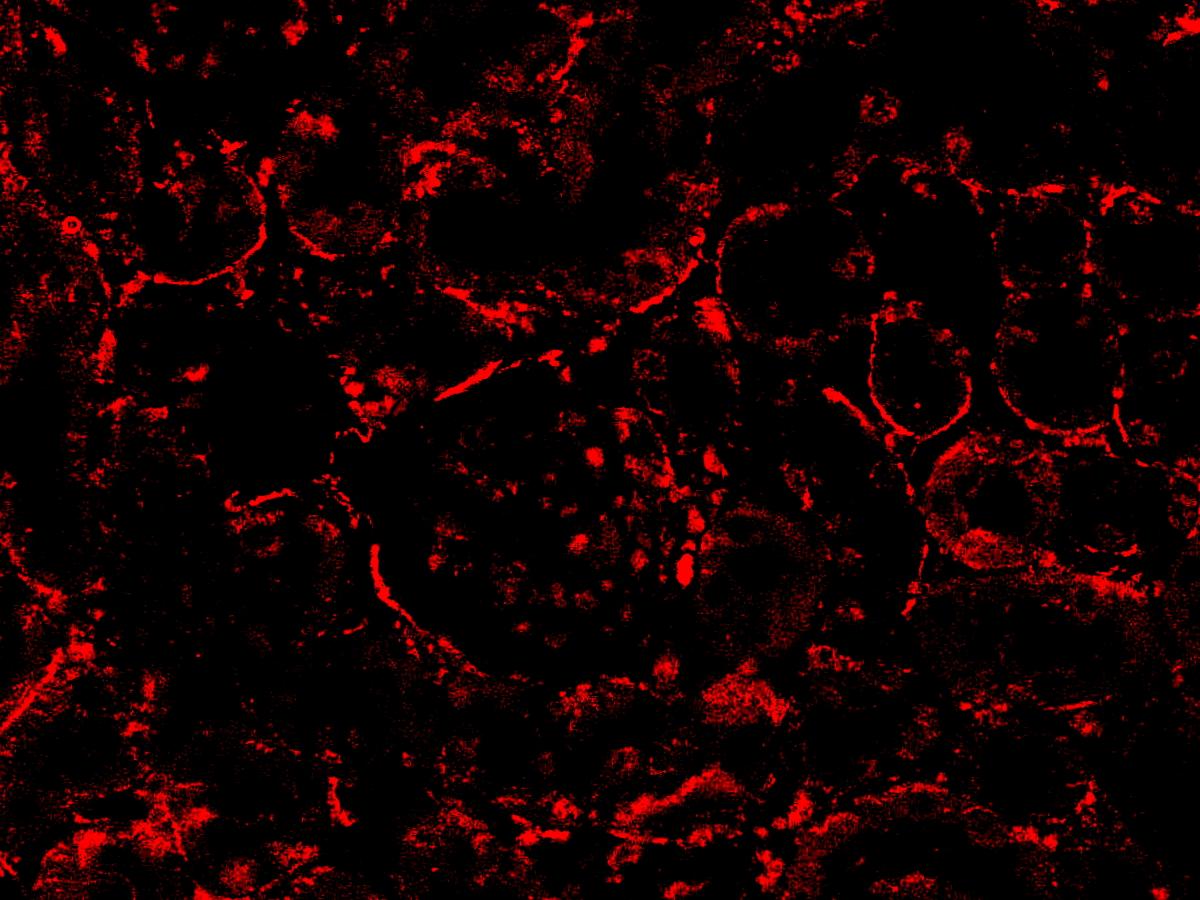

Supplement: Supplementary file 8 [file DataSheet7.zip › original images of figure 8/图8I-5-2(Synaptopodin).jpg]

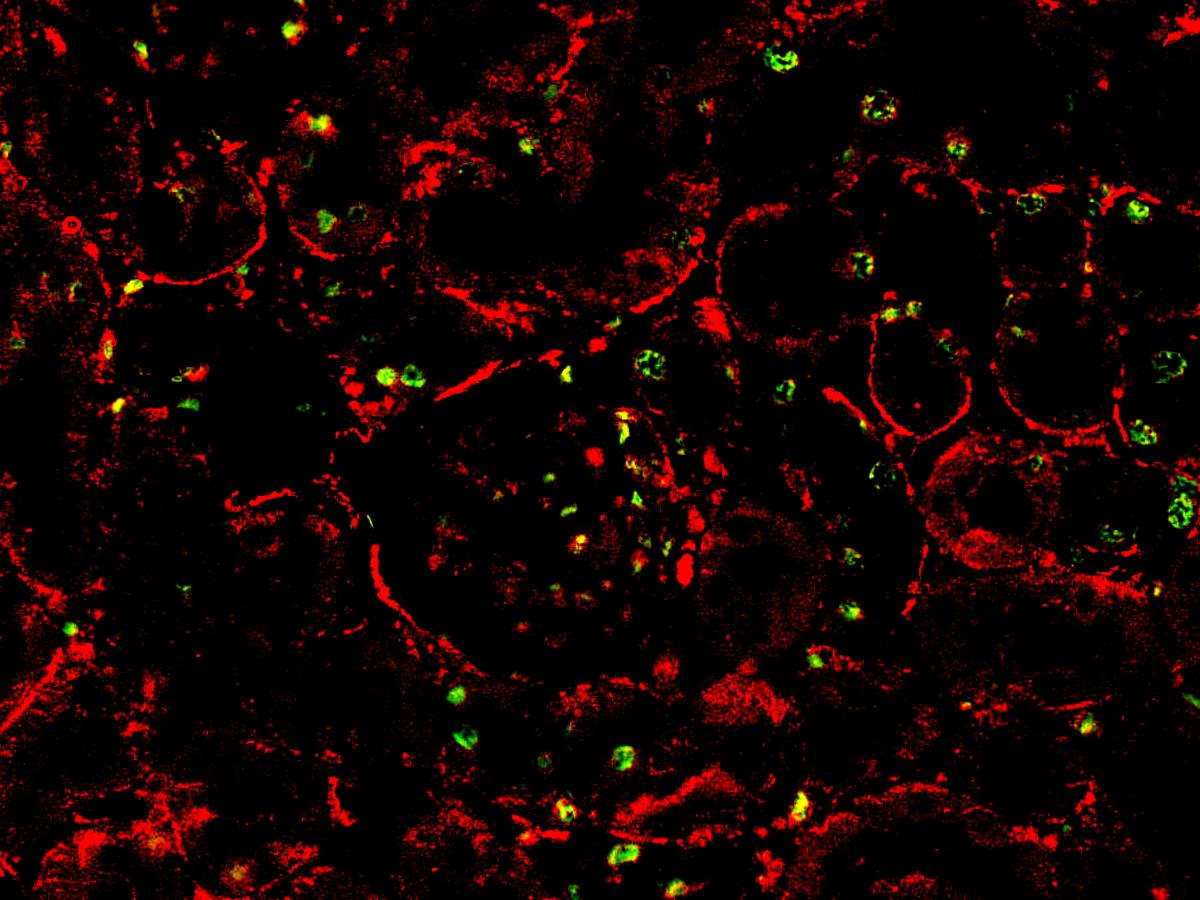

Supplement: Supplementary file 8 [file DataSheet7.zip › original images of figure 8/图8I-5-3(Merge).jpg]

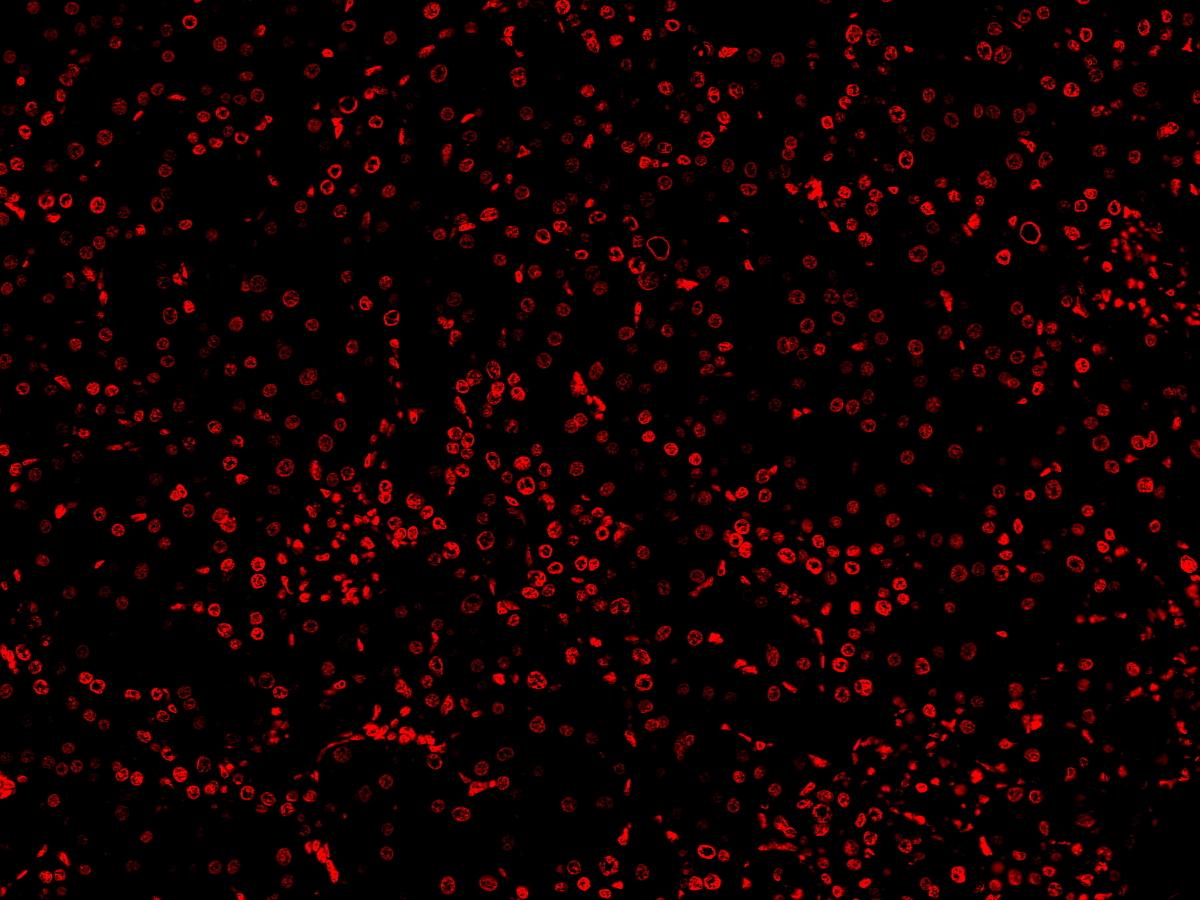

Supplement: Supplementary file 8 [file DataSheet7.zip › original images of figure 8/图8J-1-1(Tunel).jpg]

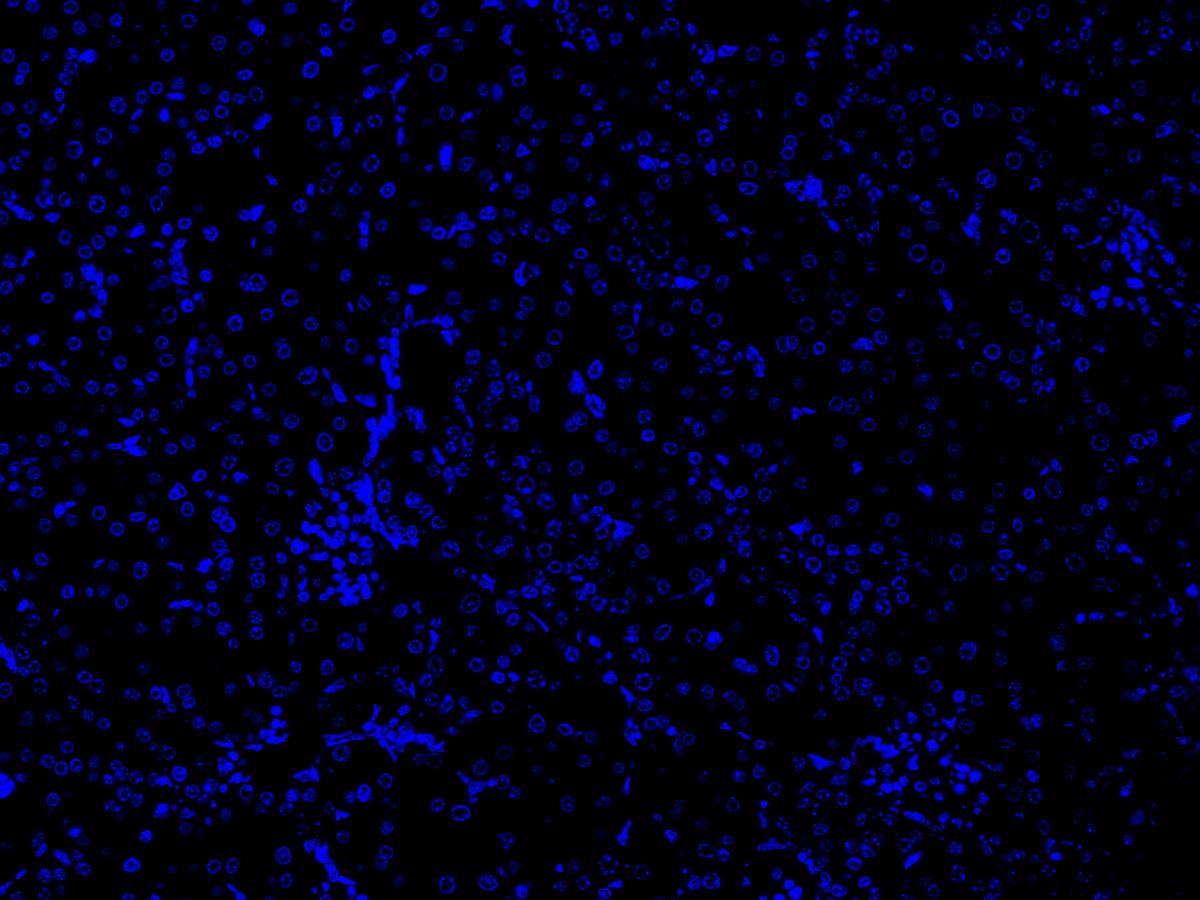

Supplement: Supplementary file 8 [file DataSheet7.zip › original images of figure 8/图8J-1-2(DAPI).jpg]

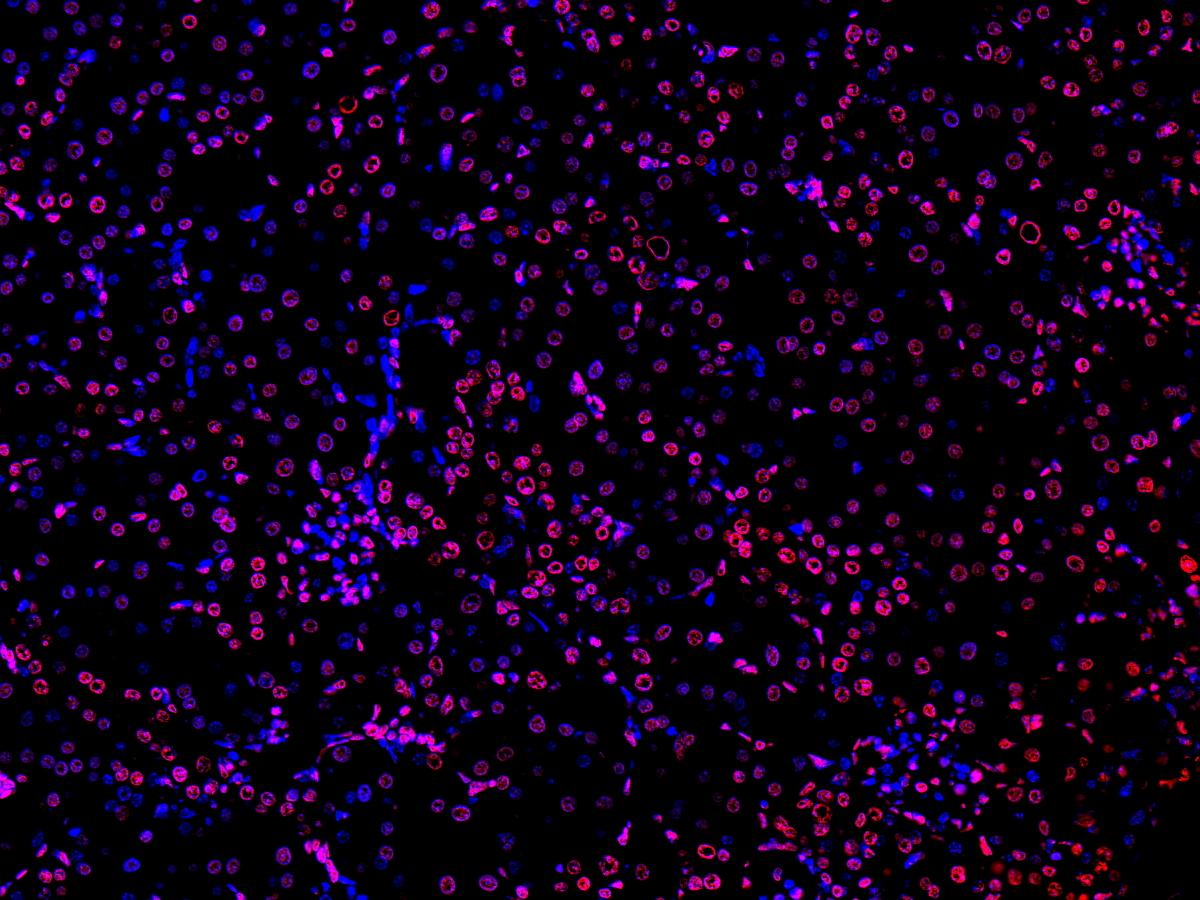

Supplement: Supplementary file 8 [file DataSheet7.zip › original images of figure 8/图8J-1-3(Merge).jpg]

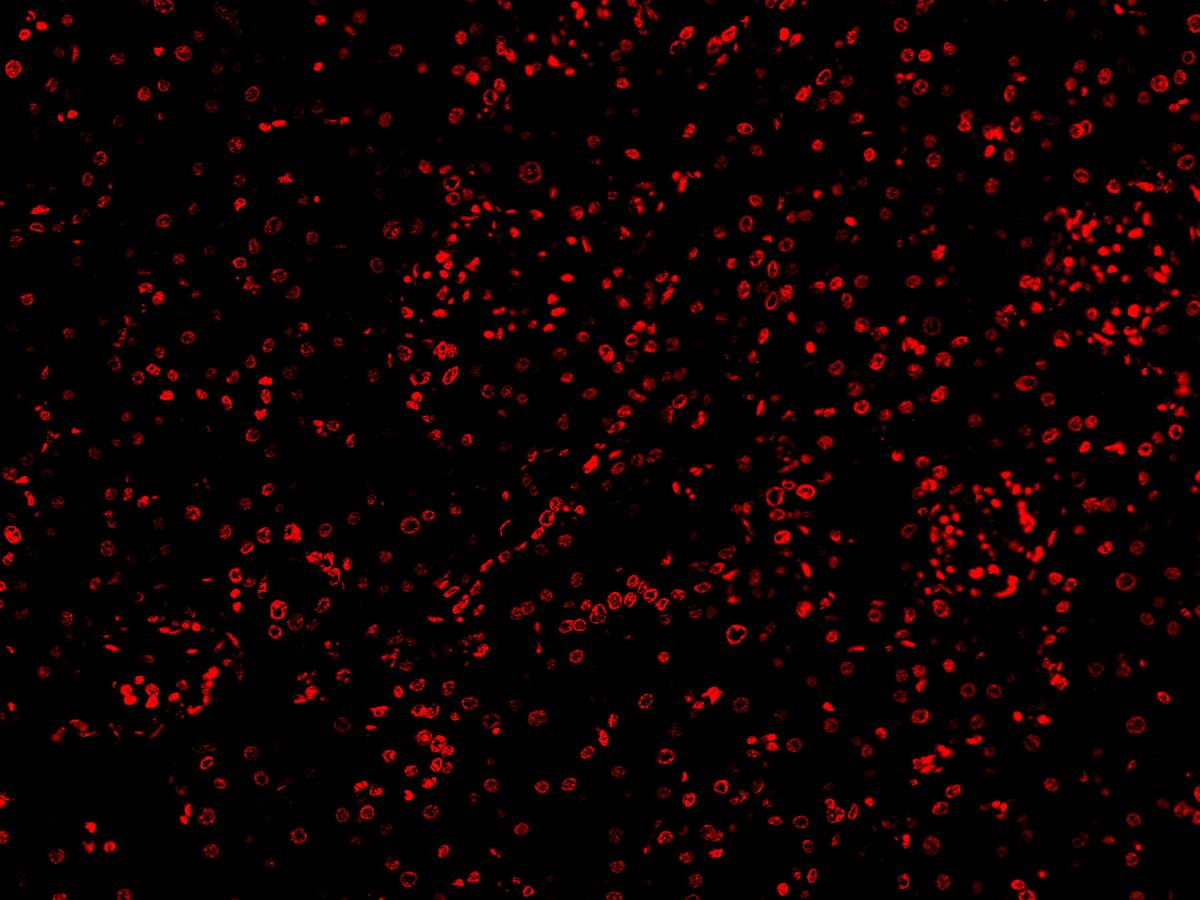

Supplement: Supplementary file 8 [file DataSheet7.zip › original images of figure 8/图8J-2-1(Tunel).jpg]

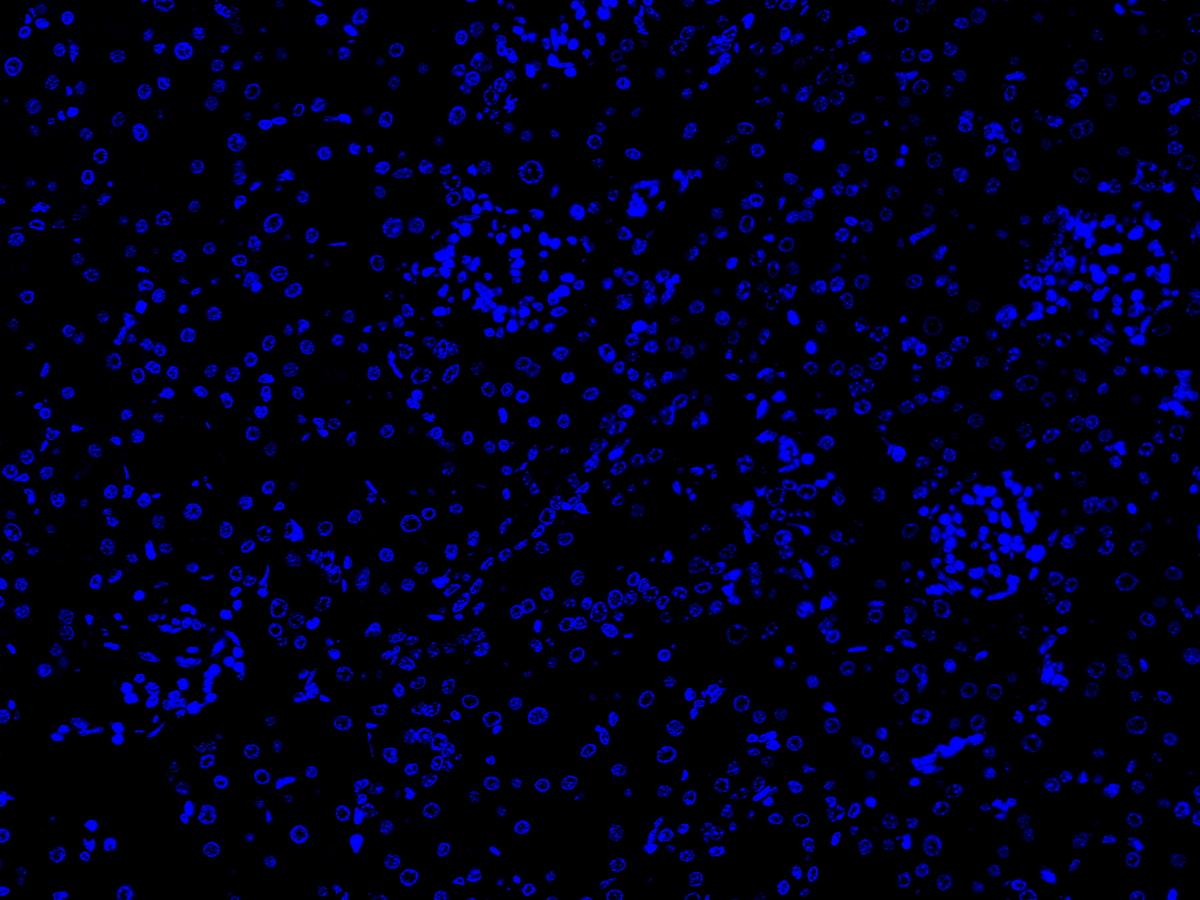

Supplement: Supplementary file 8 [file DataSheet7.zip › original images of figure 8/图8J-2-2(DAPI).jpg]

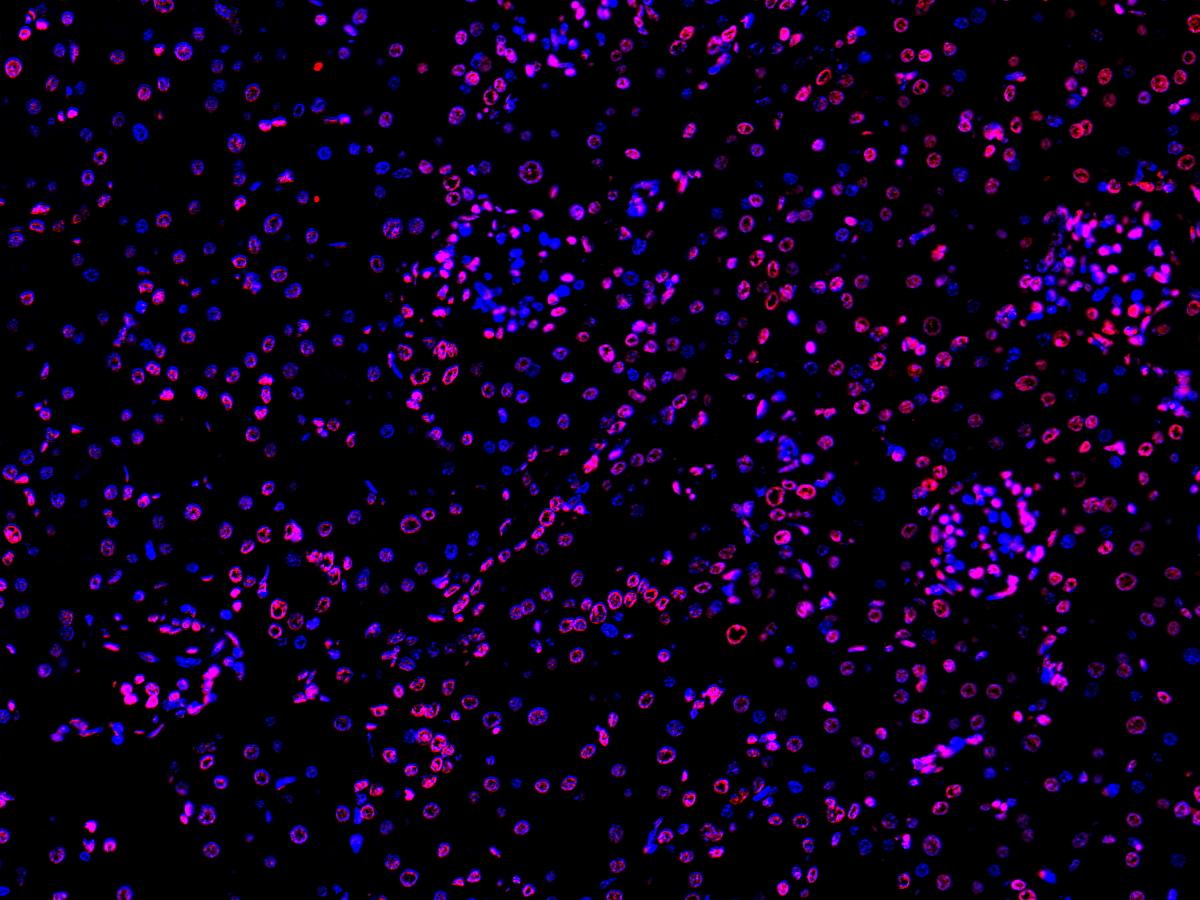

Supplement: Supplementary file 8 [file DataSheet7.zip › original images of figure 8/图8J-2-3(Merge).jpg]

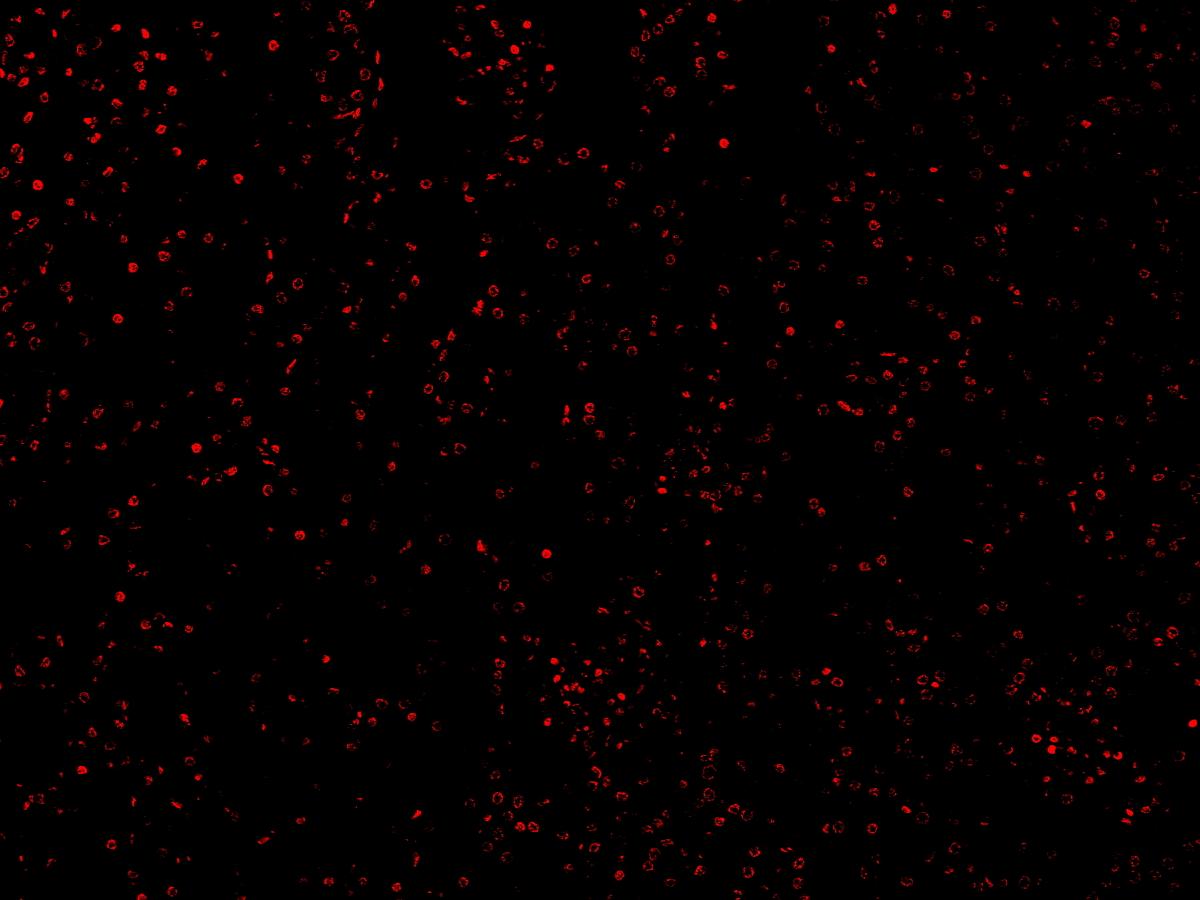

Supplement: Supplementary file 8 [file DataSheet7.zip › original images of figure 8/图8J-3-1(Tunel).jpg]

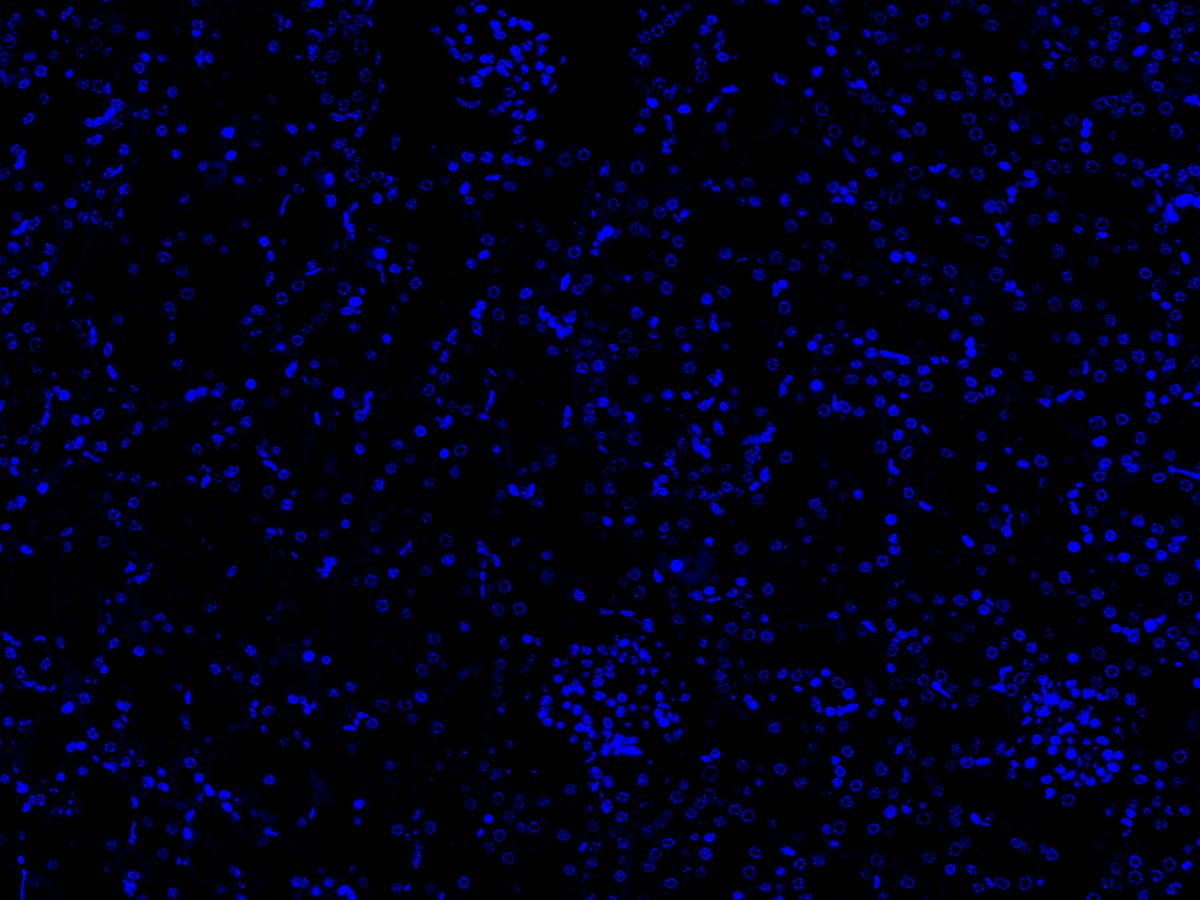

Supplement: Supplementary file 8 [file DataSheet7.zip › original images of figure 8/图8J-3-2(DAPI).jpg]

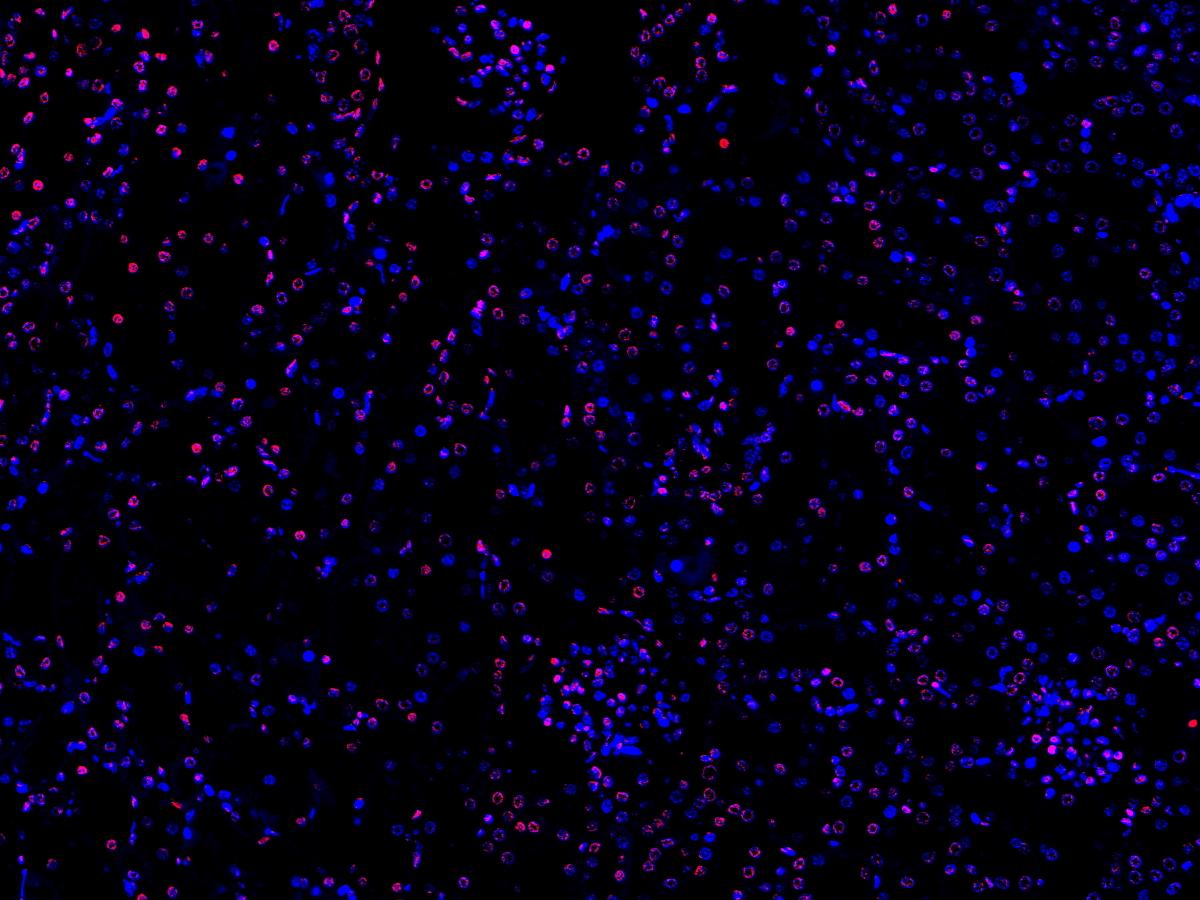

Supplement: Supplementary file 8 [file DataSheet7.zip › original images of figure 8/图8J-3-3(Merge).jpg]

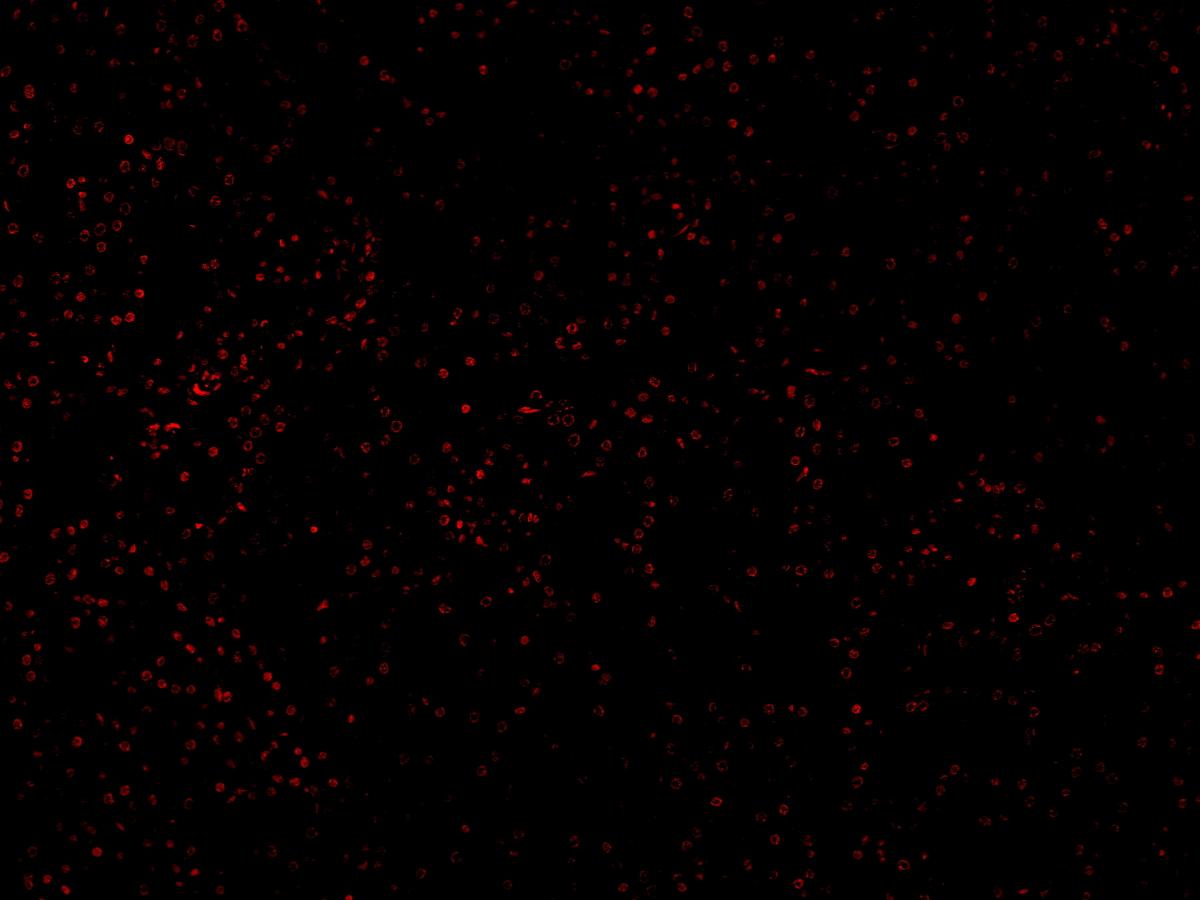

Supplement: Supplementary file 8 [file DataSheet7.zip › original images of figure 8/图8J-4-1(Tunel).jpg]

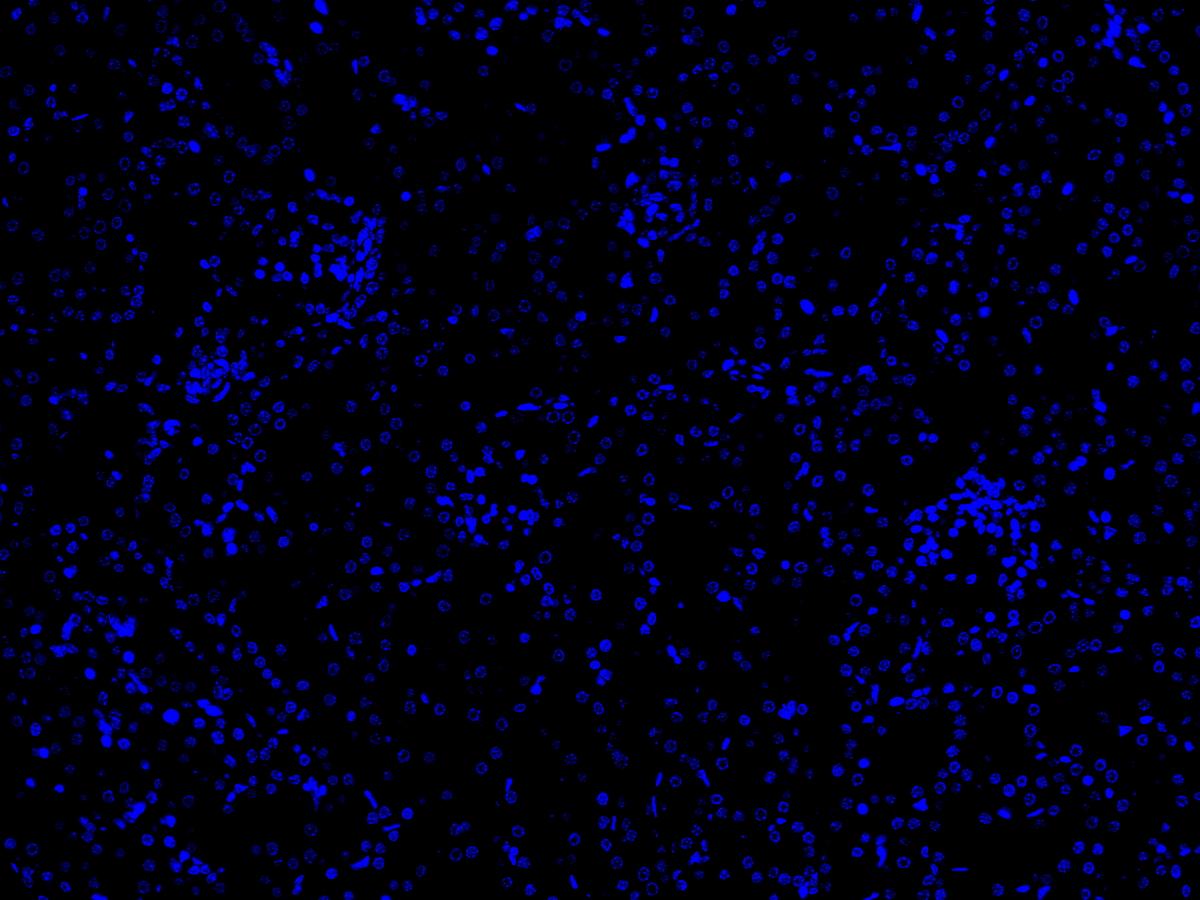

Supplement: Supplementary file 8 [file DataSheet7.zip › original images of figure 8/图8J-4-2(DAPI).jpg]

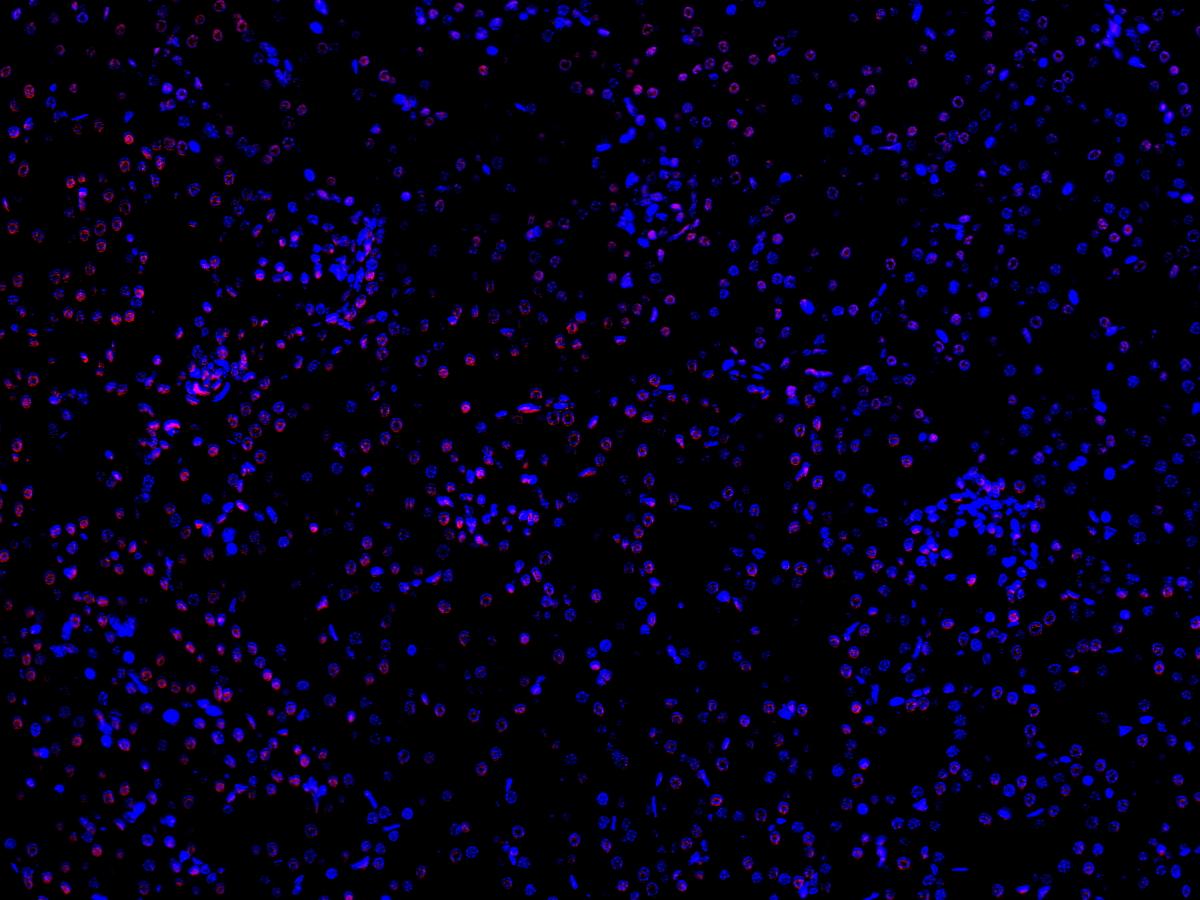

Supplement: Supplementary file 8 [file DataSheet7.zip › original images of figure 8/图8J-4-3(Merge).jpg]

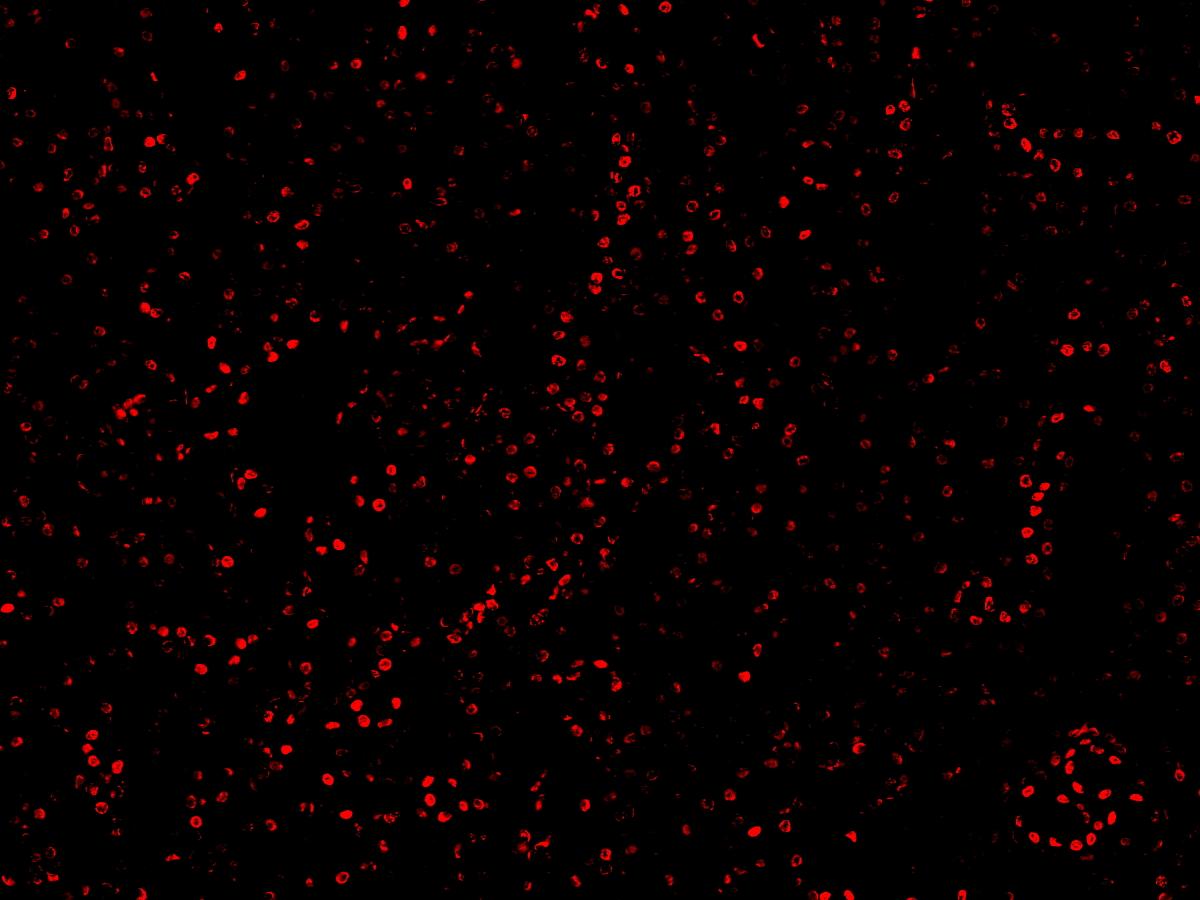

Supplement: Supplementary file 8 [file DataSheet7.zip › original images of figure 8/图8J-5-1(Tunel).jpg]

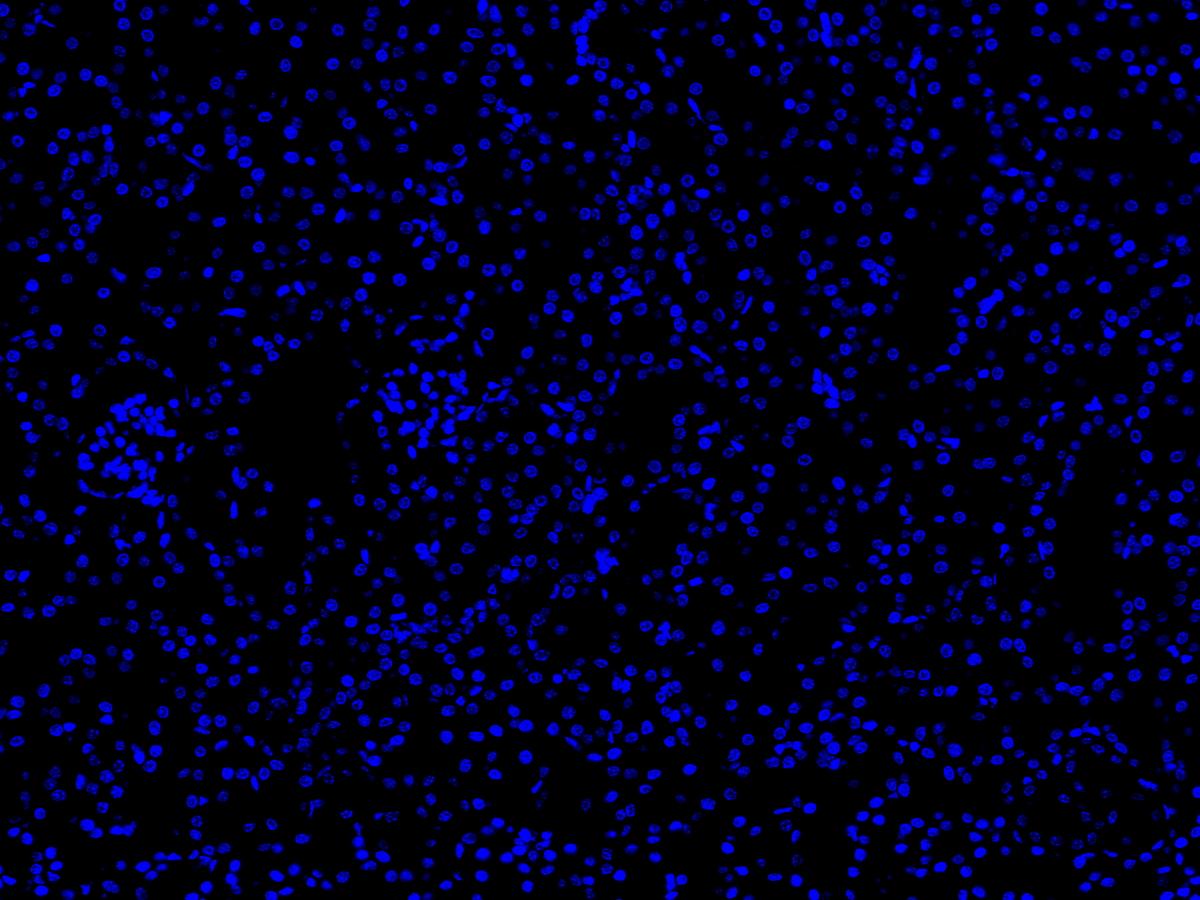

Supplement: Supplementary file 8 [file DataSheet7.zip › original images of figure 8/图8J-5-2(DAPI).jpg]

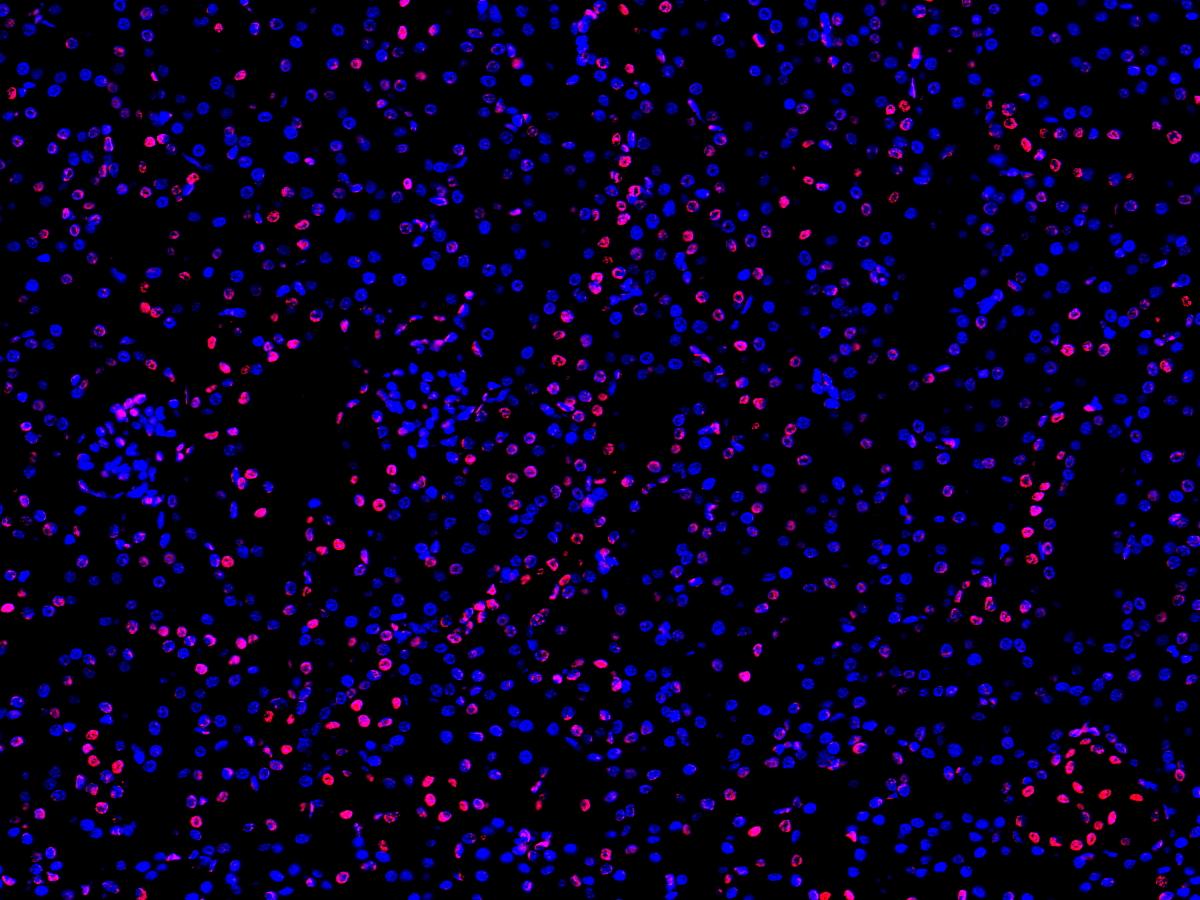

Supplement: Supplementary file 8 [file DataSheet7.zip › original images of figure 8/图8J-5-3(Merge).jpg]
